# Supplementary material for: Retinal Proteomic Analysis in a Mouse Model of Endotoxin-Induced Uveitis Using Data-Independent Acquisition-Based Mass Spectrometry
Source: Int J Mol Sci. 2022 Jun 9;23(12):6464. doi: 10.3390/ijms23126464 (PMC9223489; doi:10.3390/ijms23126464)
Supplement: Supplementary file 1 [file ijms-23-06464-s001.zip › supplementary pdf/Table S2.pdf]

Table S2. All differentially expressed proteins identified (Fold change>1.4 or <-1.4, $p<0.05$, unpaired t-test).

No.	Uniprot ID	Gene names	Protein names	Fold change	*p*-value
1	B2RWH3	Hist2h2aa1	Histone H2A	13.450	0.003
2	Q3UBS3	Hp	Haptoglobin	11.649	0.000
3	Q91X72	Hpx	Hemopexin	4.584	0.000
4	P43274	H14	Histone H1.4 (H1 VAR.2) (H1e)	4.519	0.019
5	Q8CGP2	Hist1h2bp	Histone H2B type 1-P	4.344	0.010
6	Q3UER8	Fgg	Fibrinogen gamma chain	4.177	0.000
7	E9PV24	Fga	Fibrinogen alpha chain [Cleaved into: Fibrinopeptide A; Fibrinogen alpha chain]	3.690	0.000
8	Q64525	Hist2h2bb	Histone H2B type 2-B (H2b 616)	3.307	0.013
9	Q9D0J8	Ptms	Parathymosin	3.082	0.007
10	Q1WWK3	H1f5	Hist1h1b protein (Fragment)	2.537	0.023
11	B2RTM0	Hist2h4	Histone H4	2.366	0.037
12	D3YTR5	Mbd3	Methyl-CpG-binding domain protein 3	2.191	0.001
13	P11531	Dmd	Dystrophin	2.050	0.003
14	Q9CZU7	Lamp2	Uncharacterized protein	2.042	0.000
15	Q8VC72	Ndufs8	Complex I-23kD (NADH dehydrogenase [ubiquinone] iron-sulfur protein 8, mitochondrial) (NADH-ubiquinone oxidoreductase 23 kDa subunit)	2.019	0.000
16	P62274	Rps29	40S ribosomal protein S29	1.980	0.000
17	Q52L78	Cryab	Alpha(B)-crystallin (Alpha-crystallin B chain)	1.914	0.029

18	Q5XK38	Hmgn2	Hmgn2 protein (Non-histone chromosomal protein HMG-17)	1.865	0.007
19	B9EHY2	Zmpste24	CAAX prenyl protease (EC 3.4.24.84)	1.843	0.016
20	D3Z383	Mest	Mesoderm-specific transcript protein	1.810	0.001
21	E9QLB2	Lyplal1	Lysophospholipase-like protein 1	1.749	0.019
22	Q8BHB9	Clic6	Chloride intracellular channel protein 6	1.741	0.000
23	Q9D2D1	Ctsa	Carboxypeptidase (EC 3.4.16.-)	1.739	0.005
24	O08599	Stxbp1	Syntaxin-binding protein 1 (Protein unc-18 homolog 1) (Unc18-1) (Protein unc-18 homolog A) (Unc-18A)	1.727	0.000
25	Q5NC81	Nme1	Nucleoside diphosphate kinase (EC 2.7.4.6)	1.715	0.000
26	Q9ES97-3	Rtn3	Reticulon-3	1.710	0.000
27	Q9D323	Stat1	Signal transducer and activator of transcription	1.707	0.007
28	P39053	Dnm1	Dynamin-1 (EC 3.6.5.5)	1.705	0.001
29	Q8CCG5	Ralb	Small monomeric GTPase (EC 3.6.5.2)	1.694	0.004
30	Q545Q2	Surf4	Surfeit locus protein 4	1.667	0.000
31	Q3U0T9	Rab35	Rab35	1.645	0.003
32	A0A087WNP6	Cdv3	Protein CDV3	1.640	0.002
33	P83870	Phf5a	PHD finger-like domain-containing protein 5A (PHD finger-like domain protein 5A) (Splicing factor 3B-associated 14 kDa protein) (SF3b14b)	1.636	0.000
34	Q6PGH1	Bud31	Protein BUD31 homolog (Protein G10 homolog)	1.635	0.001
35	Q3UMA3	Hgs	Hepatocyte growth factor-regulated tyrosine kinase substrate	1.611	0.000
36	A0A087WSR7	Agfg1	Arf-GAP domain and FG repeat-containing protein 1	1.596	0.040

37	Q571E2	Atp6ap1	MFLJ00383 protein (Fragment)	1.588	0.000
38	Q8C8H5	Gabrg2	Uncharacterized protein	1.587	0.000
39	Q9CPU4	Mgst3	Microsomal glutathione S-transferase 3 (Microsomal GST-3) (Glutathione peroxidase MGST3) (EC 1.11.1.-) (Microsomal glutathione S-transferase III) (Microsomal GST-III)	1.581	0.000
40	Q8BLF1	Nceh1	Neutral cholesterol ester hydrolase 1 (NCEH) (EC 3.1.1.-) (Arylacetamide deacetylase-like 1) (Chlorpyrifos oxon-binding protein) (CPO-BP)	1.577	0.000
41	Q52KP0	Rpl38	60S ribosomal protein L38	1.571	0.002
42	Q7TNR6	Igsf21	Immunoglobulin superfamily member 21 (IgSF21)	1.559	0.000
43	Q3UJ76	Ap2s1	AP complex subunit sigma	1.550	0.002
44	Q61838	Pzp	Pregnancy zone protein (Alpha-2-macroglobulin) (Alpha-2-M) [Cleaved into: Alpha-2-macroglobulin 165 kDa subunit; Alpha-2-macroglobulin 35 kDa subunit]	1.534	0.039
45	Q8BG13	Rbm3	RNA-binding protein 3 (Rbm3 protein)	1.530	0.000
46	Q4KML7	Ezr	Ezrin	1.525	0.013
47	Q7TN25	Sf3a2	Sf3a2 protein	1.521	0.000
48	A0A0A0MQM0	Eif5a	Eukaryotic translation initiation factor 5A (eIF-5A) (Fragment)	1.504	0.000
49	B2RXS4	Plxnb2	Plexin-B2	1.491	0.000
50	Q8BU20	Ndufb5	Complex I-SGDH (NADH dehydrogenase [ubiquinone] 1 beta subcomplex subunit 5, mitochondrial) (NADH-ubiquinone oxidoreductase SGDH subunit)	1.487	0.010

51	Q3U9X2	Lsm2	U6 snRNA-associated Sm-like protein LSm2	1.484	0.002
52	D3Z7P3	Gls	Glutaminase kidney isoform, mitochondrial (GLS) (EC 3.5.1.2) [Cleaved into: Glutaminase kidney isoform, mitochondrial 68 kDa chain; Glutaminase kidney isoform, mitochondrial 65 kDa chain]	1.481	0.019
53	Q9DAK9	Phpt1	14 kDa phosphohistidine phosphatase (EC 3.9.1.3) (Phosphohistidine phosphatase 1) (PHPT1) (Protein histidine phosphatase) (PHP)	1.480	0.004
54	P05132	Prkaca	cAMP-dependent protein kinase catalytic subunit alpha (PKA C-alpha) (EC 2.7.11.11)	1.470	0.006
55	Q8C8Y5	Samd7	Sterile alpha motif domain-containing protein 7 (SAM domain-containing protein 7)	1.470	0.022
56	P62071	Rras2	Ras-related protein R-Ras2 (EC 3.6.5.-)	1.467	0.040
57	F6WDS8	Rgs6	Regulator of G-protein-signaling 6	1.462	0.002
58	P27661	H2ax	Histone H2AX (H2a/x) (Histone H2A.X)	1.459	0.005
59	Q69ZK9	Nlgn2	Neuroligin-2	1.455	0.029
60	Q7TND9	Dbt	Dihydrolipoamide acetyltransferase component of pyruvate dehydrogenase complex (EC 2.3.1.-)	1.448	0.016
61	Q9D0L7	Armc10	Armadillo repeat-containing protein 10	1.444	0.025
62	A0A0R4J0I1	Serpina3k	Serine protease inhibitor A3K	1.439	0.028
63	Q63844	Mapk3	Mitogen-activated protein kinase 3 (MAP kinase 3) (MAPK 3) (EC 2.7.11.24) (ERT2) (Extracellular signal-regulated kinase 1) (ERK-1) (Insulin-stimulated MAP2 kinase) (MAP kinase isoform p44) (p44-MAPK) (MNK1) (Microtubule-associated protein 2 kinase) (p44-ERK1)	1.439	0.001

64	Q8C2N9	Msh6	DNA mismatch repair protein	1.437	0.035
65	Q5M8Q0	Rpl15	Ribosomal protein L15	1.429	0.000
66	Q3TF81	Rpp30	Uncharacterized protein (Fragment)	1.426	0.006
67	Q8C7S2	Lima1	LIM zinc-binding domain-containing protein	1.423	0.026
68	Q8BFY6	Pef1	Peflin (PEF protein with a long N-terminal hydrophobic domain) (Penta-EF hand domain-containing protein 1)	1.414	0.000
69	Q3V424	Snrpg	Small nuclear ribonucleoprotein G (snRNP-G)	1.412	0.000
70	Q91VM5	Rbmxl1	RNA binding motif protein, X-linked-like-1 (Heterogeneous nuclear ribonucleoprotein G-like 1) (RNA binding motif protein, X chromosome retrogene)	1.412	0.009
71	Q80ZX0	Sec24b	Sec24-related gene family, member B (S. cerevisiae)	1.412	0.011
72	Q3UUU4	Septin6	Septin	1.407	0.025
73	Q9Z0W3	Nup160	Nuclear pore complex protein Nup160 (160 kDa nucleoporin) (Gene trap locus 1-13 protein) (GTL-13) (Nucleoporin Nup160)	1.406	0.003
74	Q9D7P1	Rps24	40S ribosomal protein S24	1.406	0.000
75	D3Z645	Vps29	Vacuolar protein sorting-associated protein 29 (Vesicle protein sorting 29)	1.405	0.001
76	Q9MD68	mtCo1	Cytochrome c oxidase subunit 1 (EC 7.1.1.9)	1.403	0.003
77	P15105	Glul	Glutamine synthetase (GS) (EC 6.3.1.2) (Glutamate--ammonia ligase) (Palmitoyltransferase GLUL) (EC 2.3.1.225)	1.403	0.001

78	Q99LR1	Abhd12	Lysophosphatidylserine lipase ABHD12 (EC 3.1.1.-) (2-arachidonoylglycerol hydrolase ABHD12) (Abhydrolase domain-containing protein 12) (Monoacylglycerol lipase ABHD12) (EC 3.1.1.23) (Oxidized phosphatidylserine lipase ABHD12) (EC 3.1.-.-)	1.401	0.000
79	B0QZN5	Vamp2	Synaptobrevin-2 (Vesicle-associated membrane protein 2)	1.400	0.000
80	P56375	Acyp2	Acylphosphatase-2 (EC 3.6.1.7) (Acylphosphatase, muscle type isozyme) (Acylphosphate phosphohydrolase 2)	-1.403	0.034
81	Q8R2Y8	Ptrh2	Peptidyl-tRNA hydrolase 2, mitochondrial (PTH 2) (EC 3.1.1.29)	-1.406	0.001
82	Q4FZH7	Erh	Enhancer of rudimentary homolog	-1.410	0.022
83	Q69ZS5	Epn2	MKIAA1065 protein (Fragment)	-1.413	0.001
84	O70251	Eef1b	Elongation factor 1-beta (EF-1-beta)	-1.415	0.014
85	Q548W7	Dbi	Diazepam binding inhibitor	-1.417	0.008
86	P10639	Txn	Thioredoxin (Trx) (ATL-derived factor) (ADF)	-1.419	0.006
87	P62869	Elob	Elongin-B (EloB) (Elongin 18 kDa subunit) (RNA polymerase II transcription factor SIII subunit B) (SIII p18) (Transcription elongation factor B polypeptide 2)	-1.423	0.005
88	Q6PGG8	Tdrkh	Tdrkh protein	-1.435	0.046
89	Q61081	Cdc37	Hsp90 co-chaperone Cdc37 (Hsp90 chaperone protein kinase-targeting subunit) (p50Cdc37) [Cleaved into: Hsp90 co-chaperone Cdc37, N-terminally processed]	-1.437	0.002
90	Q3TN35	Sgta	SGTA_dimer domain-containing protein	-1.445	0.005

91	Q8R1W8	Impg1	Interphotoreceptor matrix proteoglycan 1 (Interphotoreceptor matrix proteoglycan of 150 kDa) (IPM-150) (Sialoprotein associated with cones and rods)	-1.446	0.001
92	Q3UL54	Rsl1d1	Uncharacterized protein	-1.446	0.005
93	Q3USR8	Ctcf	Uncharacterized protein (Fragment)	-1.448	0.033
94	Q5HZY7	Atp6v1g1	V-type proton ATPase subunit G	-1.453	0.002
95	S4R1M2	Safb	Scaffold attachment factor B1	-1.453	0.018
96	Q3UHE1-2	Pitpnm3	Membrane-associated phosphatidylinositol transfer protein 3 (Phosphatidylinositol transfer protein, membrane-associated 3) (PITPnm 3) (Pyk2 N-terminal domain-interacting receptor 1) (NIR-1)	-1.466	0.039
97	P20612	Gnat1	Guanine nucleotide-binding protein G(t) subunit alpha-1 (Transducin alpha-1 chain)	-1.470	0.000
98	P60840	Ensa	Alpha-endosulfine (ARPP-19e)	-1.478	0.000
99	Q3TX06	Gorasp2	Uncharacterized protein	-1.489	0.002
100	Q9D1L0	Chchd2	Coiled-coil-helix-coiled-coil-helix domain-containing protein 2	-1.490	0.000
101	Q8BR63	Fam177a1	Protein FAM177A1	-1.497	0.023
102	Q9CPX4	Ftl1	Ferritin	-1.506	0.017
103	Q3ULK8	Cnbp	Uncharacterized protein	-1.507	0.000
104	Q4FJN2	Fkbp5	Peptidylprolyl isomerase (EC 5.2.1.8)	-1.510	0.001
105	Q7TN29	Smap2	Stromal membrane-associated protein 2 (Stromal membrane-associated protein 1-like)	-1.512	0.007
106	Q5M9J2	Hint2	Histidine triad nucleotide binding protein 2	-1.527	0.004

107	Q9CQ60	Pgls	6-phosphogluconolactonase (6PGL) (EC 3.1.1.31)	-1.527	0.003
108	Q80YD1	Supv3l1	ATP-dependent RNA helicase SUPV3L1, mitochondrial (EC 3.6.4.13) (Suppressor of var1 3-like protein 1) (SUV3-like protein 1)	-1.528	0.016
109	Q545F0	Mif	Macrophage migration inhibitory factor	-1.539	0.004
110	Q564F6	Pdcd5	Programmed cell death 5	-1.549	0.000
111	Q91ZZ3	Sncb	Beta-synuclein	-1.555	0.001
112	P62627	Dynlrb1	Dynein light chain roadblock-type 1 (Dynein light chain 2A, cytoplasmic)	-1.579	0.000
113	Q5M9K1	Ttr	Transthyretin	-1.582	0.010
114	Q9D0J2	Atp5d	ATP synthase F1 subunit delta	-1.584	0.000
115	Q3U9A8	Sh3bgrl	SH3 domain-binding glutamic acid-rich-like protein	-1.585	0.002
116	A0A7N9VR94	Ahnak2	AHNAK nucleoprotein 2	-1.590	0.002
117	Q3V3N5	Gnl2	Nucleolar GTP-binding protein 2 (Fragment)	-1.605	0.012
118	P84075	Hpca	Neuron-specific calcium-binding protein hippocalcin	-1.606	0.014
119	P62835	Rap1a	Ras-related protein Rap-1A (EC 3.6.5.2) (Ras-related protein Krev-1)	-1.606	0.015
120	Q8K124	Plekho2	Pleckstrin homology domain-containing family O member 2 (PH domain-containing family O member 2) (Pleckstrin homology domain-containing family Q member 1) (PH domain-containing family Q member 1)	-1.611	0.002

121	Q9JKB3	Ybx3	Y-box-binding protein 3 (Cold shock domain-containing protein A) (DNA-binding protein A) (Y-box protein 3)	-1.620	0.000
122	P62075	Timm13	Mitochondrial import inner membrane translocase subunit Tim13	-1.640	0.010
123	Q3UM23	Rnh1	Ribonuclease inhibitor (Ribonuclease/angiogenin inhibitor 1)	-1.649	0.000
124	P27546-2	Map4	Microtubule-associated protein 4 (MAP-4)	-1.650	0.001
125	B2RSH2	Gnai1	Guanine nucleotide-binding protein G(i) subunit alpha-1 (Adenylate cyclase-inhibiting G alpha protein)	-1.659	0.023
126	Q8R5L1	C1qbp	Complement component 1 Q subcomponent-binding protein, mitochondrial	-1.663	0.003
127	Q3TXU8	Pgrmc1	Cytochrome b5 heme-binding domain-containing protein	-1.669	0.004
128	Q8VD33	Sgtb	Small glutamine-rich tetratricopeptide repeat-containing protein beta (Beta-SGT)	-1.676	0.001
129	B2RTP7	Krt2	Krt2 protein	-1.692	0.035
130	Q71M36	Cspg5	Chondroitin sulfate proteoglycan 5 (Acidic leucine-rich EGF-like domain-containing brain protein) (Neuroglycan C)	-1.715	0.001
131	A0A0N4SUH8	Nfu1	NFU1 iron-sulfur cluster scaffold homolog, mitochondrial	-1.718	0.000
132	Q80XH2	Impg2	Interphotoreceptor matrix proteoglycan 2 (Sialoprotein associated with cones and rods proteoglycan) (Spacrcan)	-1.745	0.001
133	Q5SS40	Ywhae	14_3_3 domain-containing protein	-1.759	0.001

134	Q08331	Calb2	Calretinin (CR)	-1.801	0.000
135	P34022	Ranbp1	Ran-specific GTPase-activating protein (HpaII tiny fragments locus 9a protein) (Ran-binding protein 1) (RANBP1)	-1.813	0.003
136	Q9CV53	Gcsh	Glycine cleavage system H protein (Fragment)	-1.815	0.006
137	Q99MR0	Actl6b	Actin-like protein 6B (53 kDa BRG1-associated factor B) (Actin-related protein Baf53b) (ArpN-alpha) (ArpNa) (BRG1-associated factor 53B) (BAF53B)	-1.839	0.028
138	D3YXH0	Gm49948	Predicted gene, 49948	-1.842	0.001
139	Q14C38	Tceal5	Transcription elongation factor A (SII)-like 5	-1.844	0.000
140	Q4V9T8	Eif1	Eukaryotic translation initiation factor 1	-1.853	0.001
141	P43081	Guca1a	Guanylyl cyclase-activating protein 1 (GCAP 1) (Guanylate cyclase activator 1A)	-1.873	0.000
142	Q9DBZ5	Eif3k	Eukaryotic translation initiation factor 3 subunit K (eIF3k) (Eukaryotic translation initiation factor 3 subunit 12) (eIF-3 p25)	-1.874	0.000
143	A0A0A1HAM8	Marcks	80K protein	-1.926	0.000
144	Q9D1J2	Cope	Coatomer subunit epsilon	-1.978	0.003
145	P51863	Atp6v0d1	V-type proton ATPase subunit d 1 (V-ATPase subunit d 1) (P39) (Physophilin) (V-ATPase 40 kDa accessory protein) (V-ATPase AC39 subunit) (Vacuolar proton pump subunit d 1)	-1.982	0.006
146	Q6IRU5	Cltb	Clathrin light chain B (Lcb)	-2.039	0.028
147	A0A087WQE6	Eloc	Elongin-C (Fragment)	-2.053	0.000

148	P99028	Uqcrh	Cytochrome b-c1 complex subunit 6, mitochondrial (Complex III subunit 6) (Complex III subunit VIII) (Cytochrome c1 non-heme 11 kDa protein) (Mitochondrial hinge protein) (Ubiquinol-cytochrome c reductase complex 11 kDa protein)	-2.066	0.008
149	Q642K0	Myl6	Myl6 protein (Myosin, light polypeptide 6, alkali, smooth muscle and non-muscle)	-2.172	0.002
150	P06837	Gap43	Neuromodulin (Axonal membrane protein GAP-43) (Calmodulin-binding protein P-57) (Growth-associated protein 43)	-2.180	0.000
151	F8WIA1	Clip1	CAP-Gly domain-containing linker protein 1	-2.192	0.020
152	Q9JKB1	Uchl3	Ubiquitin carboxyl-terminal hydrolase isozyme L3 (UCH-L3) (EC 3.4.19.12) (Ubiquitin thioesterase L3)	-2.205	0.003
153	P12658	Calb1	Calbindin (Calbindin D28) (D-28K) (PCD-29) (Spot 35 protein) (Vitamin D-dependent calcium-binding protein, avian-type)	-2.274	0.007
154	Q9D1K2	Atp6v1f	V-type proton ATPase subunit F (V-ATPase subunit F) (V-ATPase 14 kDa subunit) (Vacuolar proton pump subunit F)	-2.335	0.000
155	B1AWD9	Clta	Clathrin light chain	-2.340	0.012
156	P99027	Rplp2	60S acidic ribosomal protein P2	-2.378	0.003
157	G3UZY2	Txn2	Thioredoxin, mitochondrial (Fragment)	-2.458	0.000
158	Q80WM3	Cplx4	Complexin-4 (Complexin IV) (CPX IV)	-2.534	0.002
159	Q61012	Gngt1	Guanine nucleotide-binding protein G(T) subunit gamma-T1 (Transducin gamma chain)	-2.565	0.025

160	Q6W8Q3	Pcp4l1	Purkinje cell protein 4-like protein 1 (PCP4-like protein 1)	-2.568	0.000
161	Q3TE95	Rcn2	Uncharacterized protein	-2.569	0.000
162	Q05186	Rcn1	Reticulocalbin-1	-2.642	0.000
163	P60879	Snap25	Synaptosomal-associated protein 25 (SNAP-25) (Super protein) (SUP) (Synaptosomal-associated 25 kDa protein)	-2.695	0.000
164	Q3U6E4	Ptma	Uncharacterized protein	-2.933	0.000
165	A0A087WPA0	Lbhd1	LBH domain-containing 1	-3.062	0.000
166	Q3TL58	Skp1	S-phase kinase-associated protein 1	-3.117	0.000
167	Q542N8	Palm	Uncharacterized protein	-3.287	0.000
168	P0DP28	Calm3	Calmodulin-3	-4.184	0.004
169	Q91XV3	Basp1	Brain acid soluble protein 1 (22 kDa neuronal tissue-enriched acidic protein) (Neuronal axonal membrane protein NAP-22)	-4.443	0.000
