# Supplementary material for: Retinal Proteomic Analysis in a Mouse Model of Endotoxin-Induced Uveitis Using Data-Independent Acquisition-Based Mass Spectrometry
Source: Int J Mol Sci. 2022 Jun 9;23(12):6464. doi: 10.3390/ijms23126464 (PMC9223489; doi:10.3390/ijms23126464)
Supplement: Supplementary file 1 [file ijms-23-06464-s001.zip › supplementary pdf/Table S3.pdf]

Table S3. Enriched KEGG pathways identified by STRING

# Term ID	Term description	Observed gene count	Background gene count	Strength	FDR	Matching proteins in your network (labels)
mmu04744	Phototransduction	4	25	1.32	0.006	Gnat1,Calm3,Gngt1,Guca1a
mmu04721	Synaptic vesicle cycle	8	72	1.17	0.000	Atp6v1f,Atp6v0d1,Vamp2,Cplx4, Atp6v1g1,Ap2s1,Dnm1,Stxbp1
mmu04966	Collecting duct acid secretion	3	27	1.17	0.044	Atp6v1f,Atp6v0d1,Atp6v1g1
mmu00190	Oxidative phosphorylation	9	130	0.96	0.000	Atp6v1f,Atp6v0d1,Atp6ap1, Atp6v1g1,Ndufs8,Uqcrh,mt-Co1,Atp5d,Ndufb5
mmu04727	GABAergic synapse	6	86	0.96	0.006	Prkaca,Gngt1,Gabrg2,Gnai1,Glul,Gls
mmu04961	Endocrine and other factor-regulated calcium reabsorption	4	58	0.96	0.040	Prkaca,Calb1,Ap2s1,Dnm1
mmu04720	Long-term potentiation	4	63	0.92	0.046	Prkaca,Calm3,Mapk3,Rap1a
mmu05211	Renal cell carcinoma	4	66	0.9	0.047	Mapk3,Tceb2,Rap1a,Tceb1
mmu04971	Gastric acid secretion	4	70	0.88	0.049	Prkaca,Calm3,Ezr,Gnai1
mmu05034	Alcoholism	8	147	0.86	0.003	Prkaca,Calm3,Gngt1,H2afx,Mapk3, Gnai1,Hist1h2bp,Hist2h2bb
mmu04724	Glutamatergic synapse	6	111	0.85	0.015	Prkaca,Gngt1,Mapk3,Gnai1,Glul,Gls
mmu04713	Circadian entrainment	5	93	0.85	0.034	Prkaca,Calm3,Gngt1,Mapk3,Gnai1
mmu04611	Platelet activation	6	120	0.82	0.020	Prkaca,Fgg,Mapk3,Gnai1,Rap1a,Fga
mmu04723	Retrograde endocannabinoid signaling	7	143	0.81	0.009	Prkaca,Gngt1,Mapk3,Gabrg2,Gnai1, Ndufs8,Ndufb5

mmu04371	Apelin signaling pathway	6	133	0.77	0.030	Prkaca,Calm3,Gngt1,Mapk3,Rras2, Gnai1
mmu04114	Oocyte meiosis	5	115	0.76	0.047	Prkaca,Calm3,Skp1a,Mapk3,Ywhae
mmu05012	Parkinson disease	10	239	0.74	0.003	Txn2,Prkaca,Calm3,Txn1,Gnai1, Ndufs8,Uqcrh,mt-Co1,Atp5d,Ndufb5
mmu05203	Viral carcinogenesis	7	194	0.68	0.034	Prkaca,Atp6v0d1,Mapk3,Ywhae, Hist1h2bp,Ranbp1,Hist2h2bb
mmu04014	Ras signaling pathway	7	224	0.62	0.047	Ralb,Prkaca,Calm3,Gngt1,Mapk3, Rras2,Rap1a
mmu05170	Human immunodeficiency virus 1 infection	7	222	0.62	0.047	Calm3,Gngt1,Skp1a,Mapk3,Tceb2, Gnai1,Tceb1
mmu04714	Thermogenesis	7	225	0.61	0.047	Prkaca,Ndufs8,Uqcrh,mt-Co1,Atp5d,Ndufb5,Actl6b
mmu05200	Pathways in cancer	12	528	0.48	0.034	Ralb,Msh6,Prkaca,Calm3,Mgst3, Gngt1,Skp1a,Mapk3,Tceb2,Stat1, Gnai1,Tceb1
