# Supplementary material for: Retinal Proteomic Analysis in a Mouse Model of Endotoxin-Induced Uveitis Using Data-Independent Acquisition-Based Mass Spectrometry
Source: Int J Mol Sci. 2022 Jun 9;23(12):6464. doi: 10.3390/ijms23126464 (PMC9223489; doi:10.3390/ijms23126464)
Supplement: Supplementary file 1 [file ijms-23-06464-s001.zip › supplementary pdf/Table S1.pdf]

Table S1. The full list of 3397 identified unique proteins at global 1% FDR (%Cov: coverage of proteins; %Cov(95) : protein sequence coverage in which the peptides were identified at 95% confidence; Peptide (95%): number of peptides at 95% confidence)

No.	Unused score	Total score	%Cov	%Cov(95)	Accession	Name	Species	Peptides (95%)
1	400.03	400.03	54.74	48.08	sp|Q9JHU4|DYHC1_MOUSE	Cytoplasmic dynein 1 heavy chain 1 OS=Mus musculus OX=10090 GN=Dync1h1 PE=1 SV=2	MOUSE	218
2	320.95	320.92	72.98	68.21	tr|A3KGU5|A3KGU5_MOUSE	Spectrin alpha chain, non-erythrocytic 1 OS=Mus musculus OX=10090 GN=Sptan1 PE=1 SV=1	MOUSE	170
3	236.32	236.32	65.04	60.47	sp|Q62261|SPTB2_MOUSE	Spectrin beta chain, non-erythrocytic 1 OS=Mus musculus OX=10090 GN=Sptbn1 PE=1 SV=2	MOUSE	129
4	197.04	197.04	65.75	63.49	tr|Q5SXR6|Q5SXR6_MOUSE	Clathrin heavy chain OS=Mus musculus OX=10090 GN=Cltc PE=1 SV=1	MOUSE	139
5	164.18	164.17	55.52	50.28	sp|P14873|MAP1B_MOUSE	Microtubule-associated protein 1B OS=Mus musculus OX=10090 GN=Map1b PE=1 SV=2	MOUSE	89
6	157.33	157.33	52.61	44.75	sp|Q8CJ40|CROCC_MOUSE	Rootletin OS=Mus musculus OX=10090 GN=Crocc PE=1 SV=2	MOUSE	87
7	137.13	137.13	70.24	63.87	tr|B6ZHD0|B6ZHD0_MOUSE	Erythrocyte protein band 4.1-like 2 OS=Mus musculus OX=10090 GN=Epb41l2 PE=2 SV=1	MOUSE	84
8	133.66	133.64	93.32	92.40	tr|Q5FW97|Q5FW97_MOUSE	2-phospho-D-glycerate hydro-lyase OS=Mus musculus OX=10090 GN=EG433182 PE=1 SV=1	MOUSE	167
9	129.03	129.39	85.69	82.86	sp|P52480|KPYM_MOUSE	Pyruvate kinase PKM OS=Mus musculus OX=10090 GN=Pkm PE=1 SV=4	MOUSE	122
10	127.18	127.18	58.48	56.24	tr|A0A0G2JGX4|A0A0G2JGX4_MOUSE	Sodium/potassium-transporting ATPase subunit alpha OS=Mus musculus OX=10090 GN=Atp1a3 PE=1 SV=1	MOUSE	94
11	121.53	121.53	75.40	68.68	sp|P46460|NSF_MOUSE	Vesicle-fusing ATPase OS=Mus musculus OX=10090 GN=Nsf PE=1 SV=2	MOUSE	66
12	119.88	119.88	92.56	92.31	tr|Q3UPX6|Q3UPX6_MOUSE	Arrestin_C domain-containing protein OS=Mus musculus OX=10090 GN=Sag PE=2 SV=1	MOUSE	126
13	116.78	116.89	39.87	30.84	sp|Q99PV0|PRP8_MOUSE	Pre-mRNA-processing-splicing factor 8 OS=Mus musculus OX=10090 GN=Prpf8 PE=1 SV=2	MOUSE	60
14	115.65	115.71	43.59	37.03	sp|Q6P4T2|U520_MOUSE	U5 small nuclear ribonucleoprotein 200 kDa helicase OS=Mus musculus OX=10090 GN=Snrnp200 PE=1 SV=1	MOUSE	59

15	115.61	115.61	64.53	61.51	tr|Q8K0A8|Q8K0A8_MOUSE	Phosphodiesterase OS=Mus musculus OX=10090 GN=Pde6a PE=1 SV=1	MOUSE	69
16	111.82	111.82	64.94	62.89	tr|Q80Y52|Q80Y52_MOUSE	Heat shock protein 90, alpha (Cytosolic), class A member 1 OS=Mus musculus OX=10090 GN=Hsp90aa1 PE=1 SV=2	MOUSE	68
17	111.5	111.53	38.87	32.38	sp|O35600|ABCA4_MOUSE	Retinal-specific phospholipid-transporting ATPase ABCA4 OS=Mus musculus OX=10090 GN=Abca4 PE=2 SV=1	MOUSE	57
18	110.03	110.03	63.73	59.37	tr|Q6GQU1|Q6GQU1_MOUSE	Hexokinase OS=Mus musculus OX=10090 GN=Hk1 PE=2 SV=1	MOUSE	59
19	109.2	109.22	76.09	72.39	sp|O08599|STXB1_MOUSE	Syntaxin-binding protein 1 OS=Mus musculus OX=10090 GN=Stxbp1 PE=1 SV=2	MOUSE	71
20	108.11	108.12	28.91	19.03	tr|S4R2F3|S4R2F3_MOUSE	Ankyrin-2 OS=Mus musculus OX=10090 GN=Ank2 PE=1 SV=3	MOUSE	51
21	107.15	107.15	42.49	31.75	sp|P19096|FAS_MOUSE	Fatty acid synthase OS=Mus musculus OX=10090 GN=Fasn PE=1 SV=2	MOUSE	55
22	106.7	106.69	61.72	60.96	tr|B9EHN0|B9EHN0_MOUSE	Ubiquitin-activating enzyme E1 OS=Mus musculus OX=10090 GN=Uba1 PE=1 SV=1	MOUSE	67
23	106.11	106.12	83.26	80.47	tr|Q5FWJ3|Q5FWJ3_MOUSE	Vimentin OS=Mus musculus OX=10090 GN=Vim PE=1 SV=1	MOUSE	66
24	105	105.03	73.64	69.90	sp|P14733|LMNB1_MOUSE	Lamin-B1 OS=Mus musculus OX=10090 GN=Lmnb1 PE=1 SV=3	MOUSE	62
25	104.66	105.05	42.52	31.99	tr|Q3UH59|Q3UH59_MOUSE	Myosin-10 OS=Mus musculus OX=10090 GN=Myh10 PE=1 SV=1	MOUSE	51
26	103.32	103.32	61.35	60.45	tr|A0A0R4J0H3|A0A0R4J0H3_MOUSE	Retinol-binding protein 3 OS=Mus musculus OX=10090 GN=Rbp3 PE=1 SV=1	MOUSE	57
27	103.22	103.22	74.14	70.52	sp|Q03265|ATPA_MOUSE	ATP synthase subunit alpha, mitochondrial OS=Mus musculus OX=10090 GN=Atp5f1a PE=1 SV=1	MOUSE	75
28	102.42	102.46	55.22	47.15	sp|Q80TJ1-4|CAPS1_MOUSE	Isoform 4 of Calcium-dependent secretion activator 1 OS=Mus musculus OX=10090 GN=Cadps	MOUSE	53
29	101.57	102.16	46.34	39.95	sp|Q64511|TOP2B_MOUSE	DNA topoisomerase 2-beta OS=Mus musculus OX=10090 GN=Top2b PE=1 SV=2	MOUSE	53
30	101.45	101.46	47.18	45.09	tr|E9QNN1|E9QNN1_MOUSE	DEAH box protein 9 OS=Mus musculus OX=10090 GN=Dhx9 PE=1 SV=1	MOUSE	55
31	100.64	100.66	65.63	63.31	tr|A0A0J9YUN4|A0A0J9YUN4_MOUSE	Dynamin GTPase OS=Mus musculus OX=10090 GN=Dnm1 PE=1 SV=1	MOUSE	61

32	99.48	99.48	70.47	67.12	sp|Q01853|TERA_MOUSE	Transitional endoplasmic reticulum ATPase OS=Mus musculus OX=10090 GN=Vcp PE=1 SV=4	MOUSE	62
33	98.33	98.34	84.38	84.38	tr|D2KHZ9|D2KHZ9_MOUSE	Glyceraldehyde-3-phosphate dehydrogenase OS=Mus musculus OX=10090 GN=Gapdh PE=2 SV=1	MOUSE	144
34	95.71	95.71	87.42	86.74	sp|P68372|TBB4B_MOUSE	Tubulin beta-4B chain OS=Mus musculus OX=10090 GN=Tubb4b PE=1 SV=1	MOUSE	131
35	95.01	95.02	98.08	94.24	sp|P09411|PGK1_MOUSE	Phosphoglycerate kinase 1 OS=Mus musculus OX=10090 GN=Pgk1 PE=1 SV=4	MOUSE	78
36	93.02	93.02	96.67	96.01	sp|P68369|TBA1A_MOUSE	Tubulin alpha-1A chain OS=Mus musculus OX=10090 GN=Tuba1a PE=1 SV=1	MOUSE	115
37	89.48	89.48	69.81	68.27	tr|Q3TQ13|Q3TQ13_MOUSE	Uncharacterized protein OS=Mus musculus OX=10090 GN=Hspa8 PE=2 SV=1	MOUSE	75
38	89.39	89.4	85.25	83.95	sp|P50516|VATA_MOUSE	V-type proton ATPase catalytic subunit A OS=Mus musculus OX=10090 GN=Atp6v1a PE=1 SV=2	MOUSE	50
39	88.42	88.44	51.39	50.13	sp|P13595|NCAM1_MOUSE	Neural cell adhesion molecule 1 OS=Mus musculus OX=10090 GN=Ncam1 PE=1 SV=3	MOUSE	67
40	87.22	87.22	64.92	57.11	tr|Q3UMI7|Q3UMI7_MOUSE	Tr-type G domain-containing protein OS=Mus musculus OX=10090 GN=Eef2 PE=2 SV=1	MOUSE	47
41	87.21	87.22	71.03	63.85	sp|Q99KI0|ACON_MOUSE	Aconitate hydratase, mitochondrial OS=Mus musculus OX=10090 GN=Aco2 PE=1 SV=1	MOUSE	45
42	86.47	86.48	67.10	61.40	tr|Q571M2|Q571M2_MOUSE	Heat shock 70 kDa protein 4 (Fragment) OS=Mus musculus OX=10090 GN=Hspa4 PE=2 SV=1	MOUSE	46
43	85.29	85.29	64.22	54.88	tr|Q8CC13|Q8CC13_MOUSE	AP complex subunit beta OS=Mus musculus OX=10090 GN=Ap1b1 PE=2 SV=1	MOUSE	50
44	85.11	85.11	53.95	50.68	tr|Q3V117|Q3V117_MOUSE	ATP-citrate synthase OS=Mus musculus OX=10090 GN=Acly PE=1 SV=1	MOUSE	44
45	84.8	84.9	52.13	44.10	sp|G5E829|AT2B1_MOUSE	Plasma membrane calcium-transporting ATPase 1 OS=Mus musculus OX=10090 GN=Atp2b1 PE=1 SV=1	MOUSE	45
46	83.78	83.79	91.48	90.38	tr|Q5FWB7|Q5FWB7_MOUSE	Fructose-bisphosphate aldolase OS=Mus musculus OX=10090 GN=Aldoa PE=1 SV=1	MOUSE	91
47	82.92	82.93	49.51	44.55	sp|Q6ZQ38|CAND1_MOUSE	Cullin-associated NEDD8-dissociated protein 1 OS=Mus musculus OX=10090 GN=Cand1 PE=1 SV=2	MOUSE	47
48	82.67	82.71	89.16	87.35	tr|Q564E2|Q564E2_MOUSE	L-lactate dehydrogenase OS=Mus musculus OX=10090 GN=Ldha PE=1 SV=1	MOUSE	63

49	80.82	80.84	41.11	25.45	tr|E9PUM4|E9PUM4_MOUSE	Talin-2 OS=Mus musculus OX=10090 GN=Tln2 PE=1 SV=1	MOUSE	43
50	80.55	80.55	80.42	76.57	sp|P40142|TKT_MOUSE	Transketolase OS=Mus musculus OX=10090 GN=Tkt PE=1 SV=1	MOUSE	55
51	79.12	84.72	71.00	64.80	sp|P48722|HS74L_MOUSE	Heat shock 70 kDa protein 4L OS=Mus musculus OX=10090 GN=Hspa4l PE=1 SV=2	MOUSE	48
52	76.54	76.54	53.47	44.61	sp|Q60597-3|ODO1_MOUSE	Isoform 3 of 2-oxoglutarate dehydrogenase, mitochondrial OS=Mus musculus OX=10090 GN=Ogdh	MOUSE	38
53	76.16	76.16	58.08	51.07	sp|Q9WUB3|PYGM_MOUSE	Glycogen phosphorylase, muscle form OS=Mus musculus OX=10090 GN=Pygm PE=1 SV=3	MOUSE	37
54	75.62	75.62	80.97	77.75	tr|E9PWE8|E9PWE8_MOUSE	Dihydropyrimidinase-related protein 3 OS=Mus musculus OX=10090 GN=Dpysl3 PE=1 SV=1	MOUSE	46
55	75.01	75.04	56.61	51.17	tr|Q6PEE6|Q6PEE6_MOUSE	AP-2 complex subunit alpha OS=Mus musculus OX=10090 GN=Ap2a2 PE=2 SV=1	MOUSE	39
56	74.39	74.41	73.19	67.11	tr|Q546G4|Q546G4_MOUSE	Serum albumin OS=Mus musculus OX=10090 GN=Alb PE=1 SV=1	MOUSE	39
57	74.3	74.3	57.38	52.50	tr|Q3TVV6|Q3TVV6_MOUSE	Uncharacterized protein OS=Mus musculus OX=10090 GN=Hnrnpu PE=2 SV=1	MOUSE	55
58	74.25	74.27	64.16	52.68	tr|A0A1L1SV25|A0A1L1SV25_MOUSE	Alpha-actinin-4 OS=Mus musculus OX=10090 GN=Actn4 PE=1 SV=1	MOUSE	40
59	73.28	73.3	77.85	76.06	sp|P50396|GDIA_MOUSE	Rab GDP dissociation inhibitor alpha OS=Mus musculus OX=10090 GN=Gdi1 PE=1 SV=3	MOUSE	44
60	71.86	71.86	68.32	52.54	sp|Q7TSJ2|MAP6_MOUSE	Microtubule-associated protein 6 OS=Mus musculus OX=10090 GN=Map6 PE=1 SV=2	MOUSE	36
61	71.84	72.86	85.83	83.73	sp|Q04447|KCRB_MOUSE	Creatine kinase B-type OS=Mus musculus OX=10090 GN=Ckb PE=1 SV=1	MOUSE	95
62	71.7	71.72	54.76	52.38	tr|A1A4T2|A1A4T2_MOUSE	Alpha glucosidase 2 alpha neutral subunit OS=Mus musculus OX=10090 GN=Ganab PE=2 SV=1	MOUSE	41
63	71.47	71.49	43.25	41.93	sp|Q3U1J4|DDB1_MOUSE	DNA damage-binding protein 1 OS=Mus musculus OX=10090 GN=Ddb1 PE=1 SV=2	MOUSE	39
64	71.38	71.43	47.13	33.31	tr|F7BQW7|F7BQW7_MOUSE	Phosphoinositide 5-phosphatase (Fragment) OS=Mus musculus OX=10090 GN=Synj1 PE=1 SV=1	MOUSE	34
65	71.36	71.37	75.22	68.06	sp|P63038|CH60_MOUSE	60 kDa heat shock protein, mitochondrial OS=Mus musculus OX=10090 GN=Hspd1 PE=1 SV=1	MOUSE	40

66	71.35	71.38	87.20	86.40	tr|Q3UAF7|Q3UAF7_MOUSE	Uncharacterized protein OS=Mus musculus OX=10090 GN=Actb PE=2 SV=1	MOUSE	88
67	71.19	71.22	85.25	82.31	sp|P15105|GLNA_MOUSE	Glutamine synthetase OS=Mus musculus OX=10090 GN=Glul PE=1 SV=6	MOUSE	94
68	71.11	71.17	56.59	52.68	tr|Q8BTS0|Q8BTS0_MOUSE	DEAD box protein 5 OS=Mus musculus OX=10090 GN=Ddx5 PE=1 SV=1	MOUSE	44
69	70.74	70.84	63.51	53.51	sp|Q91VR5|DDX1_MOUSE	ATP-dependent RNA helicase DDX1 OS=Mus musculus OX=10090 GN=Ddx1 PE=1 SV=1	MOUSE	36
70	69.64	69.64	53.80	46.63	sp|Q11011|PSA_MOUSE	Puromycin-sensitive aminopeptidase OS=Mus musculus OX=10090 GN=Npepps PE=1 SV=2	MOUSE	38
71	69.47	69.55	16.27	5.90	tr|E9PVY8|E9PVY8_MOUSE	Microtubule-actin cross-linking factor 1 OS=Mus musculus OX=10090 GN=Macf1 PE=1 SV=2	MOUSE	32
72	69.33	69.33	54.86	46.88	tr|Q91V38|Q91V38_MOUSE	Heat shock protein 90, beta (Grp94), member 1 OS=Mus musculus OX=10090 GN=Hsp90b1 PE=2 SV=1	MOUSE	36
73	69.17	69.18	56.38	52.93	sp|Q9WUA3|PFKAP_MOUSE	ATP-dependent 6-phosphofructokinase, platelet type OS=Mus musculus OX=10090 GN=Pfkp PE=1 SV=1	MOUSE	38
74	68.66	68.67	60.66	57.63	sp|Q91VD9|NDUS1_MOUSE	NADH-ubiquinone oxidoreductase 75 kDa subunit, mitochondrial OS=Mus musculus OX=10090 GN=Ndufs1 PE=1 SV=2	MOUSE	36
75	68.5	68.55	34.03	22.87	tr|D3YZ62|D3YZ62_MOUSE	Unconventional myosin-Va OS=Mus musculus OX=10090 GN=Myo5a PE=1 SV=1	MOUSE	36
76	67.51	67.51	83.29	80.87	sp|P05201|AATC_MOUSE	Aspartate aminotransferase, cytoplasmic OS=Mus musculus OX=10090 GN=Got1 PE=1 SV=3	MOUSE	50
77	67.45	73.48	71.57	66.91	tr|Q6P1J1|Q6P1J1_MOUSE	Crmp1 protein OS=Mus musculus OX=10090 GN=Crmp1 PE=1 SV=1	MOUSE	42
78	66.81	66.81	46.13	44.72	tr|Q6ZQ61|Q6ZQ61_MOUSE	MKIAA0723 protein (Fragment) OS=Mus musculus OX=10090 GN=Matr3 PE=2 SV=1	MOUSE	37
79	66.79	66.8	65.77	62.01	sp|P06745|G6PI_MOUSE	Glucose-6-phosphate isomerase OS=Mus musculus OX=10090 GN=Gpi PE=1 SV=4	MOUSE	52
80	66.66	66.72	49.92	35.36	tr|E9Q3M3|E9Q3M3_MOUSE	Dynactin subunit 1 OS=Mus musculus OX=10090 GN=Dctn1 PE=1 SV=1	MOUSE	34
81	65.21	78.34	52.24	50.49	sp|O08528|HXK2_MOUSE	Hexokinase-2 OS=Mus musculus OX=10090 GN=Hk2 PE=1 SV=1	MOUSE	44

82	65.14	94.03	57.61	52.34	tr|A2VDG2|A2VDG2_MOUSE	Phosphodiesterase (Fragment) OS=Mus musculus OX=10090 GN=Pde6b PE=2 SV=1	MOUSE	61
83	65.03	65.05	51.00	44.10	tr|G5E866|G5E866_MOUSE	Splicing factor 3B subunit 1 OS=Mus musculus OX=10090 GN=Sf3b1 PE=1 SV=1	MOUSE	37
84	64.83	64.84	69.13	64.11	sp|Q8BH59|CMC1_MOUSE	Calcium-binding mitochondrial carrier protein Aralar1 OS=Mus musculus OX=10090 GN=Slc25a12 PE=1 SV=1	MOUSE	35
85	64.8	64.86	69.71	68.86	sp|P20612|GNAT1_MOUSE	Guanine nucleotide-binding protein G(t) subunit alpha-1 OS=Mus musculus OX=10090 GN=Gnat1 PE=1 SV=3	MOUSE	55
86	64.66	64.71	84.70	69.97	sp|O88569|ROA2_MOUSE	Heterogeneous nuclear ribonucleoproteins A2/B1 OS=Mus musculus OX=10090 GN=Hnrnpa2b1 PE=1 SV=2	MOUSE	49
87	64.64	64.7	68.28	51.11	tr|Q5F2D0|Q5F2D0_MOUSE	Uncharacterized protein OS=Mus musculus OX=10090 GN=Acsl6 PE=2 SV=1	MOUSE	33
88	64.08	64.14	74.24	65.08	tr|Q66JR7|Q66JR7_MOUSE	Pgm2 protein (Fragment) OS=Mus musculus OX=10090 GN=Pgm1 PE=2 SV=1	MOUSE	36
89	63.83	63.94	77.44	75.35	sp|P05202|AATM_MOUSE	Aspartate aminotransferase, mitochondrial OS=Mus musculus OX=10090 GN=Got2 PE=1 SV=1	MOUSE	51
90	63.72	63.89	66.19	57.49	sp|P56546-2|CTBP2_MOUSE	Isoform 2 of C-terminal-binding protein 2 OS=Mus musculus OX=10090 GN=Ctbp2	MOUSE	37
91	63.65	63.7	76.75	73.16	sp|P56480|ATPB_MOUSE	ATP synthase subunit beta, mitochondrial OS=Mus musculus OX=10090 GN=Atp5f1b PE=1 SV=2	MOUSE	61
92	63.61	63.67	45.22	36.82	sp|P52785|GUC2E_MOUSE	Retinal guanylyl cyclase 1 OS=Mus musculus OX=10090 GN=Gucy2e PE=1 SV=2	MOUSE	31
93	63.54	63.66	70.13	68.12	sp|P48962|ADT1_MOUSE	ADP/ATP translocase 1 OS=Mus musculus OX=10090 GN=Slc25a4 PE=1 SV=4	MOUSE	54
94	63.24	63.47	51.65	45.57	sp|Q8CAQ8-5|MIC60_MOUSE	Isoform 5 of MICOS complex subunit Mic60 OS=Mus musculus OX=10090 GN=Immt	MOUSE	32
95	61.85	61.85	25.41	13.85	tr|B0V2P5|B0V2P5_MOUSE	DmX-like protein 2 OS=Mus musculus OX=10090 GN=Dmxl2 PE=1 SV=1	MOUSE	30
96	61.6	71.7	82.34	81.12	sp|O08553|DPYL2_MOUSE	Dihydropyrimidinase-related protein 2 OS=Mus musculus OX=10090 GN=Dpysl2 PE=1 SV=2	MOUSE	66
97	61.5	66.22	59.24	54.81	tr|Q3U9G2|Q3U9G2_MOUSE	78 kDa glucose-regulated protein OS=Mus musculus OX=10090 GN=Hspa5 PE=2 SV=1	MOUSE	40

98	60.52	60.53	59.08	54.65	tr|A0A3B2WCD8|A0A3B2WCD8_MOUSE	Far upstream element-binding protein 2 OS=Mus musculus OX=10090 GN=Khsrp PE=1 SV=1	MOUSE	49
99	60.51	61.18	42.88	34.01	tr|V9GXM4|V9GXM4_MOUSE	Unconventional myosin-6 OS=Mus musculus OX=10090 GN=Myo6 PE=1 SV=1	MOUSE	32
100	60.23	60.23	72.90	71.40	tr|Q542X7|Q542X7_MOUSE	CCT-beta OS=Mus musculus OX=10090 GN=Cct2 PE=1 SV=1	MOUSE	34
101	60.11	60.11	68.03	65.66	sp|P61979|HNRPK_MOUSE	Heterogeneous nuclear ribonucleoprotein K OS=Mus musculus OX=10090 GN=Hnrnpk PE=1 SV=1	MOUSE	38
102	59.91	59.96	42.95	35.57	sp|Q6P5F9|XPO1_MOUSE	Exportin-1 OS=Mus musculus OX=10090 GN=Xpo1 PE=1 SV=1	MOUSE	29
103	59.89	59.9	35.08	34.37	tr|Q8CD23|Q8CD23_MOUSE	Nucleolin OS=Mus musculus OX=10090 GN=Ncl PE=2 SV=1	MOUSE	32
104	59.65	59.66	63.67	63.33	sp|Q76MZ3|2AAA_MOUSE	Serine/threonine-protein phosphatase 2A 65 kDa regulatory subunit A alpha isoform OS=Mus musculus OX=10090 GN=Ppp2r1a PE=1 SV=3	MOUSE	36
105	59.62	59.67	54.99	41.57	sp|Q99P72|RTN4_MOUSE	Reticulon-4 OS=Mus musculus OX=10090 GN=Rtn4 PE=1 SV=2	MOUSE	34
106	59.19	59.19	36.54	36.42	tr|Q6NXX6|Q6NXX6_MOUSE	V-type proton ATPase subunit a OS=Mus musculus OX=10090 GN=Atp6v0a1 PE=2 SV=1	MOUSE	33
107	59.14	59.16	58.43	56.06	tr|H6RXZ1|H6RXZ1_MOUSE	Synaptotagmin OS=Mus musculus OX=10090 GN=Syt1 PE=1 SV=1	MOUSE	33
108	59.09	73.64	93.94	87.05	sp|P05063|ALDOC_MOUSE	Fructose-bisphosphate aldolase C OS=Mus musculus OX=10090 GN=Aldoc PE=1 SV=4	MOUSE	59
109	58.62	58.7	83.17	67.51	sp|P62814|VATB2_MOUSE	V-type proton ATPase subunit B, brain isoform OS=Mus musculus OX=10090 GN=Atp6v1b2 PE=1 SV=1	MOUSE	38
110	58.49	58.5	48.55	43.52	tr|Q3UKJ6|Q3UKJ6_MOUSE	RNA helicase OS=Mus musculus OX=10090 GN=Dhx15 PE=1 SV=1	MOUSE	33
111	58.46	58.47	65.47	62.05	sp|P11983|TCPA_MOUSE	T-complex protein 1 subunit alpha OS=Mus musculus OX=10090 GN=Tcp1 PE=1 SV=3	MOUSE	34
112	58.41	58.46	65.57	55.42	sp|Q9D0E1|HNRPM_MOUSE	Heterogeneous nuclear ribonucleoprotein M OS=Mus musculus OX=10090 GN=Hnrnpm PE=1 SV=3	MOUSE	34
113	58.33	58.45	32.34	18.61	tr|Q5DTP7|Q5DTP7_MOUSE	DNA helicase (Fragment) OS=Mus musculus OX=10090 GN=Chd4 PE=2 SV=1	MOUSE	31

114	58	58.09	50.25	39.93	sp|P48193-2|EPB41_MOUSE	Isoform 2 of Protein 4.1 OS=Mus musculus OX=10090 GN=Epb41	MOUSE	30
115	57.86	57.89	64.36	59.04	tr|G3X905|G3X905_MOUSE	G protein-coupled receptor kinase OS=Mus musculus OX=10090 GN=Grk1 PE=1 SV=1	MOUSE	31
116	57.77	57.82	49.93	48.93	sp|Q8VIJ6|SFPQ_MOUSE	Splicing factor, proline- and glutamine-rich OS=Mus musculus OX=10090 GN=Sfpq PE=1 SV=1	MOUSE	36
117	57.7	57.71	42.63	36.20	sp|Q5SUR0|PUR4_MOUSE	Phosphoribosylformylglycinamidine synthase OS=Mus musculus OX=10090 GN=Pfas PE=1 SV=1	MOUSE	34
118	57.45	57.63	73.75	63.96	sp|P80316|TCPE_MOUSE	T-complex protein 1 subunit epsilon OS=Mus musculus OX=10090 GN=Cct5 PE=1 SV=1	MOUSE	32
119	57.38	57.41	66.97	55.47	tr|Q3UL22|Q3UL22_MOUSE	CCT-theta OS=Mus musculus OX=10090 GN=Cct8 PE=1 SV=1	MOUSE	32
120	56.92	56.93	91.76	91.76	tr|Q3TQ70|Q3TQ70_MOUSE	Beta1 subnuit of GTP-binding protein OS=Mus musculus OX=10090 GN=Gnb1 PE=1 SV=1	MOUSE	72
121	56.75	56.76	71.30	69.82	sp|P08249|MDHM_MOUSE	Malate dehydrogenase, mitochondrial OS=Mus musculus OX=10090 GN=Mdh2 PE=1 SV=3	MOUSE	35
122	56.69	56.71	77.20	74.80	sp|P61922|GABT_MOUSE	4-aminobutyrate aminotransferase, mitochondrial OS=Mus musculus OX=10090 GN=Abat PE=1 SV=1	MOUSE	34
123	56.54	56.57	35.32	26.32	sp|Q8CGC7|SYEP_MOUSE	Bifunctional glutamate/proline--tRNA ligase OS=Mus musculus OX=10090 GN=Eprs1 PE=1 SV=4	MOUSE	30
124	56.27	56.3	51.81	43.62	sp|Q00PI9|HNRL2_MOUSE	Heterogeneous nuclear ribonucleoprotein U-like protein 2 OS=Mus musculus OX=10090 GN=Hnrnpul2 PE=1 SV=2	MOUSE	32
125	56.08	56.14	54.96	49.78	sp|Q8K0U4|HS12A_MOUSE	Heat shock 70 kDa protein 12A OS=Mus musculus OX=10090 GN=Hspa12a PE=1 SV=1	MOUSE	28
126	55.78	55.79	52.67	48.99	tr|Q3THQ5|Q3THQ5_MOUSE	Uncharacterized protein OS=Mus musculus OX=10090 GN=Stip1 PE=2 SV=1	MOUSE	30
127	55.72	55.75	75.00	69.15	sp|Q9EQF6|DPYL5_MOUSE	Dihydropyrimidinase-related protein 5 OS=Mus musculus OX=10090 GN=Dpysl5 PE=1 SV=1	MOUSE	29
128	55.67	55.68	68.62	60.92	tr|Q3U4U6|Q3U4U6_MOUSE	T-complex protein 1 subunit gamma OS=Mus musculus OX=10090 GN=Cct3 PE=1 SV=1	MOUSE	31
129	55.18	75.87	64.19	55.27	sp|Q8BZ98|DYN3_MOUSE	Dynamin-3 OS=Mus musculus OX=10090 GN=Dnm3 PE=1 SV=1	MOUSE	43
130	54.74	74.06	33.06	24.85	sp|Q8VDD5|MYH9_MOUSE	Myosin-9 OS=Mus musculus OX=10090 GN=Myh9 PE=1 SV=4	MOUSE	38

131	54.72	69.6	48.72	46.37	sp|P17426|AP2A1_MOUSE	AP-2 complex subunit alpha-1 OS=Mus musculus OX=10090 GN=Ap2a1 PE=1 SV=1	MOUSE	36
132	54.54	64.34	61.93	54.76	tr|Q3TU85|Q3TU85_MOUSE	Hspa1b protein OS=Mus musculus OX=10090 GN=Hspa1b PE=1 SV=1	MOUSE	45
133	54.4	87.29	51.81	45.65	sp|Q8VDN2|AT1A1_MOUSE	Sodium/potassium-transporting ATPase subunit alpha-1 OS=Mus musculus OX=10090 GN=Atp1a1 PE=1 SV=1	MOUSE	66
134	54.35	54.39	63.08	55.20	sp|P26443|DHE3_MOUSE	Glutamate dehydrogenase 1, mitochondrial OS=Mus musculus OX=10090 GN=Glud1 PE=1 SV=1	MOUSE	30
135	53.79	72.34	57.06	50.89	tr|Q3V3U0|Q3V3U0_MOUSE	Alpha-1,4 glucan phosphorylase OS=Mus musculus OX=10090 GN=Pygb PE=2 SV=1	MOUSE	37
136	53.67	53.7	36.66	29.69	tr|Q5DTI2|Q5DTI2_MOUSE	P-type Ca(2+) transporter (Fragment) OS=Mus musculus OX=10090 GN=Atp2a2 PE=2 SV=1	MOUSE	25
137	53.57	60.6	52.56	48.46	sp|P12382|PFKAL_MOUSE	ATP-dependent 6-phosphofructokinase, liver type OS=Mus musculus OX=10090 GN=Pfkl PE=1 SV=4	MOUSE	34
138	53.54	59.52	85.66	79.02	tr|Q3TMU8|Q3TMU8_MOUSE	Dihydropyrimidinase-like 4 OS=Mus musculus OX=10090 GN=Dpysl4 PE=1 SV=1	MOUSE	35
139	53.49	53.5	35.43	28.79	tr|A0A076FSX1|A0A076FSX1_MOUSE	KCC2b variant 2 OS=Mus musculus OX=10090 GN=Slc12a5 PE=2 SV=1	MOUSE	29
140	53.33	53.36	60.53	51.48	sp|D3Z7P3|GLSK_MOUSE	Glutaminase kidney isoform, mitochondrial OS=Mus musculus OX=10090 GN=Gls PE=1 SV=1	MOUSE	32
141	52.94	52.94	61.52	58.36	tr|F7DEU6|F7DEU6_MOUSE	Inosine-5'-monophosphate dehydrogenase OS=Mus musculus OX=10090 GN=Impdh1 PE=1 SV=2	MOUSE	29
142	52.87	52.89	64.81	61.08	tr|Q5SUC3|Q5SUC3_MOUSE	Uncharacterized protein OS=Mus musculus OX=10090 GN=Canx PE=1 SV=1	MOUSE	33
143	52.82	52.82	56.91	56.91	tr|G5E924|G5E924_MOUSE	Heterogeneous nuclear ribonucleoprotein L (Fragment) OS=Mus musculus OX=10090 GN=Hnrnpl PE=1 SV=1	MOUSE	36
144	52.75	52.74	83.14	80.39	tr|Q5SS40|Q5SS40_MOUSE	14_3_3 domain-containing protein OS=Mus musculus OX=10090 GN=Ywhae PE=1 SV=1	MOUSE	31
145	52.73	52.8	91.97	89.16	sp|P17751|TPIS_MOUSE	Triosephosphate isomerase OS=Mus musculus OX=10090 GN=Tpi1 PE=1 SV=5	MOUSE	43
146	52.26	52.35	47.31	39.67	sp|Q8BGQ7|SYAC_MOUSE	Alanine--tRNA ligase, cytoplasmic OS=Mus musculus OX=10090 GN=Aars1 PE=1 SV=1	MOUSE	27
147	52.16	58.27	55.94	50.92	tr|Q3UZG3|Q3UZG3_MOUSE	Uncharacterized protein OS=Mus musculus OX=10090 GN=Hnrnpa3 PE=2 SV=1	MOUSE	39

148	52.09	71.25	91.47	85.25	tr|Q545V3|Q545V3_MOUSE	2-phospho-D-glycerate hydro-lyase OS=Mus musculus OX=10090 GN=Eno2 PE=1 SV=1	MOUSE	63
149	51.81	51.86	46.00	37.63	sp|P27546-2|MAP4_MOUSE	Isoform 2 of Microtubule-associated protein 4 OS=Mus musculus OX=10090 GN=Map4	MOUSE	27
150	51.44	51.44	89.05	89.05	tr|Q3THL7|Q3THL7_MOUSE	Voltage-dependent anion-selective channel protein 1 OS=Mus musculus OX=10090 GN=Vdac1 PE=2 SV=1	MOUSE	52
151	51.41	51.47	39.19	24.90	tr|Q1HL32|Q1HL32_MOUSE	Structural maintenance of chromosomes protein OS=Mus musculus OX=10090 GN=Smc3 PE=1 SV=1	MOUSE	26
152	51.38	51.87	41.89	33.30	tr|G3X9T8|G3X9T8_MOUSE	Ceruloplasmin OS=Mus musculus OX=10090 GN=Cp PE=1 SV=1	MOUSE	26
153	51.31	51.33	59.60	55.45	sp|P27773|PDIA3_MOUSE	Protein disulfide-isomerase A3 OS=Mus musculus OX=10090 GN=Pdia3 PE=1 SV=2	MOUSE	30
154	50.83	52.9	53.17	50.22	sp|P38647|GRP75_MOUSE	Stress-70 protein, mitochondrial OS=Mus musculus OX=10090 GN=Hspa9 PE=1 SV=3	MOUSE	31
155	50.63	50.63	65.67	60.08	sp|P24549|AL1A1_MOUSE	Retinal dehydrogenase 1 OS=Mus musculus OX=10090 GN=Aldh1a1 PE=1 SV=5	MOUSE	28
156	50.4	50.41	64.01	59.66	sp|O88844|IDHC_MOUSE	Isocitrate dehydrogenase [NADP] cytoplasmic OS=Mus musculus OX=10090 GN=Idh1 PE=1 SV=2	MOUSE	29
157	50.03	50.04	76.62	66.67	tr|Q58E64|Q58E64_MOUSE	Elongation factor 1-alpha OS=Mus musculus OX=10090 GN=Eef1a1 PE=1 SV=1	MOUSE	39
158	49.99	50	79.81	78.55	tr|Q544Y3|Q544Y3_MOUSE	CRAL-TRIO domain-containing protein OS=Mus musculus OX=10090 GN=Rlbp1 PE=1 SV=1	MOUSE	30
159	49.84	49.85	55.99	51.47	tr|Q3URP6|Q3URP6_MOUSE	Protein disulfide-isomerase OS=Mus musculus OX=10090 GN=P4hb PE=2 SV=1	MOUSE	28
160	49.79	49.86	43.50	36.67	tr|A0A1L1STE4|A0A1L1STE4_MOUSE	Interleukin enhancer-binding factor 3 OS=Mus musculus OX=10090 GN=Ilf3 PE=1 SV=1	MOUSE	28
161	49.72	49.74	47.11	44.04	tr|Z4YKB8|Z4YKB8_MOUSE	Heterochromatin protein 1-binding protein 3 OS=Mus musculus OX=10090 GN=Hp1bp3 PE=1 SV=1	MOUSE	29
162	49.67	49.74	50.99	41.72	tr|Q545R0|Q545R0_MOUSE	Catenin (Cadherin associated protein), alpha 1 OS=Mus musculus OX=10090 GN=Ctnna1 PE=1 SV=1	MOUSE	27
163	49.67	49.69	55.19	50.19	tr|Q3UZT7|Q3UZT7_MOUSE	Uncharacterized protein OS=Mus musculus OX=10090 GN=Ctnnb1 PE=2 SV=1	MOUSE	25
164	49.38	49.48	36.95	31.44	sp|O54774|AP3D1_MOUSE	AP-3 complex subunit delta-1 OS=Mus musculus OX=10090 GN=Ap3d1 PE=1 SV=1	MOUSE	26

165	49.36	68.06	72.07	66.99	tr|Q3UPA3|Q3UPA3_MOUSE	Rab GDP dissociation inhibitor (Fragment) OS=Mus musculus OX=10090 GN=Gdi2 PE=2 SV=1	MOUSE	37
166	49.07	49.09	32.23	30.38	sp|Q9ERK4|XPO2_MOUSE	Exportin-2 OS=Mus musculus OX=10090 GN=Cse1l PE=1 SV=1	MOUSE	25
167	48.9	48.93	53.68	48.77	sp|P24527|LKHA4_MOUSE	Leukotriene A-4 hydrolase OS=Mus musculus OX=10090 GN=Lta4h PE=1 SV=4	MOUSE	24
168	48.68	48.71	76.38	76.38	tr|Q3U7Z6|Q3U7Z6_MOUSE	Phosphoglycerate mutase OS=Mus musculus OX=10090 GN=Pgam1 PE=1 SV=1	MOUSE	43
169	48.56	48.63	55.82	53.14	tr|Q8BN32|Q8BN32_MOUSE	Polyadenylate-binding protein OS=Mus musculus OX=10090 GN=Pabpc1 PE=2 SV=1	MOUSE	31
170	48.24	48.24	38.07	36.07	sp|Q61548|AP180_MOUSE	Clathrin coat assembly protein AP180 OS=Mus musculus OX=10090 GN=Snap91 PE=1 SV=1	MOUSE	29
171	48.18	53.23	67.72	64.19	tr|Q564F4|Q564F4_MOUSE	T-complex protein 1 subunit delta OS=Mus musculus OX=10090 GN=Cct4 PE=1 SV=1	MOUSE	35
172	48.04	48.06	59.08	55.09	sp|Q9Z2D6-2|MECP2_MOUSE	Isoform B of Methyl-CpG-binding protein 2 OS=Mus musculus OX=10090 GN=Mecp2	MOUSE	24
173	48.02	48.14	61.58	52.39	tr|Q3TIJ7|Q3TIJ7_MOUSE	T-complex protein 1 subunit eta OS=Mus musculus OX=10090 GN=Cct7 PE=1 SV=1	MOUSE	24
174	48.01	48.08	10.45	7.04	tr|A0A0R4J0I9|A0A0R4J0I9_MOUSE	Prolow-density lipoprotein receptor-related protein 1 OS=Mus musculus OX=10090 GN=Lrp1 PE=1 SV=1	MOUSE	25
175	47.94	48.11	34.23	22.87	sp|Q9CU62|SMC1A_MOUSE	Structural maintenance of chromosomes protein 1A OS=Mus musculus OX=10090 GN=Smc1a PE=1 SV=4	MOUSE	25
176	47.78	50.18	13.98	8.43	tr|Q4JG03|Q4JG03_MOUSE	HECT-type E3 ubiquitin transferase OS=Mus musculus OX=10090 GN=Huwe1 PE=2 SV=1	MOUSE	24
177	47.63	47.67	57.41	46.67	sp|Q9QYC0|ADDA_MOUSE	Alpha-adducin OS=Mus musculus OX=10090 GN=Add1 PE=1 SV=2	MOUSE	27
178	47.54	47.56	71.84	61.55	tr|Q790Y8|Q790Y8_MOUSE	Glucose-6-phosphate 1-dehydrogenase OS=Mus musculus OX=10090 GN=G6pdx PE=1 SV=1	MOUSE	27
179	47.51	49.94	54.00	36.48	tr|Q6A0E3|Q6A0E3_MOUSE	116 kDa U5 small nuclear ribonucleoprotein component (Fragment) OS=Mus musculus OX=10090 GN=Eftud2 PE=2 SV=1	MOUSE	27
180	47.47	47.49	67.62	56.78	sp|Q8K2B3|SDHA_MOUSE	Succinate dehydrogenase [ubiquinone] flavoprotein subunit, mitochondrial OS=Mus musculus OX=10090 GN=Sdha PE=1 SV=1	MOUSE	25

181	47.34	47.34	87.62	83.66	tr|Q2TB46|Q2TB46_MOUSE	Recoverin OS=Mus musculus OX=10090 GN=Rcvrn PE=2 SV=1	MOUSE	26
182	47.02	47.06	41.52	32.57	tr|A0A571BEW4|A0A571BEW4_MOUSE	AP-3 complex subunit beta OS=Mus musculus OX=10090 GN=Ap3b2 PE=1 SV=1	MOUSE	25
183	46.95	46.97	57.91	42.88	tr|Q3U8W9|Q3U8W9_MOUSE	Uncharacterized protein OS=Mus musculus OX=10090 GN=Hnrnpr PE=2 SV=1	MOUSE	25
184	46.89	48.71	63.49	54.65	sp|P03995|GFAP_MOUSE	Glial fibrillary acidic protein OS=Mus musculus OX=10090 GN=Gfap PE=1 SV=4	MOUSE	26
185	46.82	46.88	29.53	24.52	sp|Q8BYI9|TENR_MOUSE	Tenascin-R OS=Mus musculus OX=10090 GN=Tnr PE=1 SV=2	MOUSE	24
186	46.56	46.62	37.84	32.45	sp|P12960|CNTN1_MOUSE	Contactin-1 OS=Mus musculus OX=10090 GN=Cntn1 PE=1 SV=1	MOUSE	24
187	46.05	46.07	51.36	41.75	sp|Q921I1|TRFE_MOUSE	Serotransferrin OS=Mus musculus OX=10090 GN=Tf PE=1 SV=1	MOUSE	25
188	46.05	46.05	65.05	63.19	tr|Q3TF14|Q3TF14_MOUSE	Adenosylhomocysteinase OS=Mus musculus OX=10090 GN=Ahcy PE=1 SV=1	MOUSE	24
189	45.88	47.96	48.72	45.11	sp|P48678|LMNA_MOUSE	Prelamin-A/C OS=Mus musculus OX=10090 GN=Lmna PE=1 SV=2	MOUSE	26
190	45.78	87.78	66.85	62.29	tr|Q71LX8|Q71LX8_MOUSE	Heat shock protein 84b OS=Mus musculus OX=10090 GN=Hsp90ab1 PE=1 SV=1	MOUSE	52
191	45.76	60.05	60.23	54.70	sp|P21619|LMNB2_MOUSE	Lamin-B2 OS=Mus musculus OX=10090 GN=Lmnb2 PE=1 SV=2	MOUSE	31
192	45.74	45.77	52.84	42.80	sp|Q99JF8|PSIP1_MOUSE	PC4 and SFRS1-interacting protein OS=Mus musculus OX=10090 GN=Psip1 PE=1 SV=1	MOUSE	32
193	45.66	60.08	31.57	17.46	tr|Q68FG2|Q68FG2_MOUSE	Spectrin beta chain OS=Mus musculus OX=10090 GN=Sptbn2 PE=1 SV=1	MOUSE	31
194	45.64	57.96	54.26	40.38	tr|A0A5F8MPR1|A0A5F8MPR1_MOUSE	Band 4.1-like protein 3 OS=Mus musculus OX=10090 GN=Epb41l3 PE=1 SV=1	MOUSE	31
195	45.59	45.61	31.88	23.17	tr|B2RSV4|B2RSV4_MOUSE	Splicing factor 3b, subunit 3 OS=Mus musculus OX=10090 GN=Sf3b3 PE=2 SV=1	MOUSE	24
196	45.55	45.59	61.56	60.48	sp|Q9QZQ8|H2AY_MOUSE	Core histone macro-H2A.1 OS=Mus musculus OX=10090 GN=Macroh2a1 PE=1 SV=3	MOUSE	27
197	45.54	45.55	51.31	49.40	tr|Q564E8|Q564E8_MOUSE	60S ribosomal protein L4 OS=Mus musculus OX=10090 GN=Rpl4 PE=1 SV=1	MOUSE	31

198	45.52	45.55	81.31	77.85	tr|Q4FK49|Q4FK49_MOUSE	Inorganic diphosphatase OS=Mus musculus OX=10090 GN=Ppa1 PE=1 SV=1	MOUSE	24
199	45.44	45.45	42.91	39.88	sp|Q8CJG0|AGO2_MOUSE	Protein argonaute-2 OS=Mus musculus OX=10090 GN=Ago2 PE=1 SV=3	MOUSE	22
200	45.43	45.48	67.98	57.30	tr|Q8C7C4|Q8C7C4_MOUSE	Uncharacterized protein (Fragment) OS=Mus musculus OX=10090 GN=Hmgb1 PE=2 SV=1	MOUSE	35
201	45.27	45.31	55.98	50.36	sp|Q9WV34|MPP2_MOUSE	MAGUK p55 subfamily member 2 OS=Mus musculus OX=10090 GN=Mpp2 PE=1 SV=1	MOUSE	23
202	45.11	45.14	37.89	25.74	sp|O35954|PITM1_MOUSE	Membrane-associated phosphatidylinositol transfer protein 1 OS=Mus musculus OX=10090 GN=Pitpnm1 PE=1 SV=1	MOUSE	22
203	45.09	45.16	38.31	29.46	sp|Q8K4Z5|SF3A1_MOUSE	Splicing factor 3A subunit 1 OS=Mus musculus OX=10090 GN=Sf3a1 PE=1 SV=1	MOUSE	22
204	44.91	45.05	50.57	37.02	tr|Q3UJB0|Q3UJB0_MOUSE	Splicing factor 3b, subunit 2 OS=Mus musculus OX=10090 GN=Sf3b2 PE=1 SV=1	MOUSE	22
205	44.63	44.7	58.87	46.28	tr|Q5EBP9|Q5EBP9_MOUSE	Tripartite motif-containing 28 OS=Mus musculus OX=10090 GN=Trim28 PE=2 SV=1	MOUSE	26
206	44.6	49.11	58.01	47.89	tr|Q3TQX5|Q3TQX5_MOUSE	RNA helicase OS=Mus musculus OX=10090 GN=Ddx3x PE=1 SV=1	MOUSE	26
207	44.58	44.69	21.13	13.75	tr|Q7M739|Q7M739_MOUSE	Nucleoprotein TPR OS=Mus musculus OX=10090 GN=Tpr PE=1 SV=1	MOUSE	24
208	44.39	44.42	58.71	50.56	tr|E9PUD2|E9PUD2_MOUSE	Dynamin-1-like protein OS=Mus musculus OX=10090 GN=Dnm1l PE=1 SV=1	MOUSE	24
209	44.32	44.36	32.82	22.74	sp|Q810U3|NFASC_MOUSE	Neurofascin OS=Mus musculus OX=10090 GN=Nfasc PE=1 SV=1	MOUSE	24
210	44.26	46.26	41.93	28.14	tr|F8WHL2|F8WHL2_MOUSE	Coatomer subunit alpha OS=Mus musculus OX=10090 GN=Copa PE=1 SV=1	MOUSE	25
211	44.23	44.39	59.04	40.07	tr|A2A7S7|A2A7S7_MOUSE	Tyrosine--tRNA ligase OS=Mus musculus OX=10090 GN=Yars PE=1 SV=1	MOUSE	21
212	44.16	44.18	40.45	33.42	tr|Q3TRJ1|Q3TRJ1_MOUSE	Vacuolar protein sorting-associated protein 35 OS=Mus musculus OX=10090 GN=Vps35 PE=1 SV=1	MOUSE	22
213	44.12	84.09	57.62	55.21	tr|Q5SWR1|Q5SWR1_MOUSE	AP complex subunit beta OS=Mus musculus OX=10090 GN=Ap2b1 PE=2 SV=1	MOUSE	50

214	44.01	44.42	39.19	27.03	tr|Q3UHU8|Q3UHU8_MOUSE	General transcription factor II-I OS=Mus musculus OX=10090 GN=Gtf2i PE=1 SV=1	MOUSE	22
215	43.99	44.05	42.16	33.64	sp|Q61768|KINH_MOUSE	Kinesin-1 heavy chain OS=Mus musculus OX=10090 GN=Kif5b PE=1 SV=3	MOUSE	23
216	43.99	44.04	23.13	15.33	tr|A0A668KLC6|A0A668KLC6_MOUSE	Microtubule-associated protein OS=Mus musculus OX=10090 GN=Map2 PE=1 SV=1	MOUSE	24
217	43.82	43.85	64.82	57.96	sp|Q8BFR5|EFTU_MOUSE	Elongation factor Tu, mitochondrial OS=Mus musculus OX=10090 GN=Tufm PE=1 SV=1	MOUSE	23
218	43.81	43.85	76.96	61.78	sp|Q9Z1G3|VATC1_MOUSE	V-type proton ATPase subunit C 1 OS=Mus musculus OX=10090 GN=Atp6v1c1 PE=1 SV=4	MOUSE	21
219	43.57	43.6	64.69	57.76	tr|Q3TJY2|Q3TJY2_MOUSE	WD repeat domain 1 OS=Mus musculus OX=10090 GN=Wdr1 PE=1 SV=1	MOUSE	26
220	43.55	43.65	53.14	43.96	sp|Q8CAA7|PGM2L_MOUSE	Glucose 1,6-bisphosphate synthase OS=Mus musculus OX=10090 GN=Pgm2l1 PE=1 SV=1	MOUSE	23
221	43.44	43.5	64.67	61.88	sp|P97807-2|FUMH_MOUSE	Isoform Cytoplasmic of Fumarate hydratase, mitochondrial OS=Mus musculus OX=10090 GN=Fh	MOUSE	23
222	43.41	43.57	88.35	72.82	tr|B2RTM0|B2RTM0_MOUSE	Histone H4 OS=Mus musculus OX=10090 GN=Hist2h4 PE=1 SV=1	MOUSE	74
223	43.22	43.24	60.91	48.87	sp|O88935|SYN1_MOUSE	Synapsin-1 OS=Mus musculus OX=10090 GN=Syn1 PE=1 SV=2	MOUSE	35
224	43.2	43.29	39.43	33.10	sp|Q8BIJ6|SYIM_MOUSE	Isoleucine--tRNA ligase, mitochondrial OS=Mus musculus OX=10090 GN=Iars2 PE=1 SV=1	MOUSE	21
225	43.04	43.18	66.38	53.06	sp|P30416|FKBP4_MOUSE	Peptidyl-prolyl cis-trans isomerase FKBP4 OS=Mus musculus OX=10090 GN=Fkbp4 PE=1 SV=5	MOUSE	26
226	42.9	43.04	40.39	27.14	sp|Q9EPU0|RENT1_MOUSE	Regulator of nonsense transcripts 1 OS=Mus musculus OX=10090 GN=Upf1 PE=1 SV=2	MOUSE	21
227	42.83	42.84	42.51	39.76	tr|Q3U4W8|Q3U4W8_MOUSE	Ubiquitin carboxyl-terminal hydrolase OS=Mus musculus OX=10090 GN=Usp5 PE=1 SV=1	MOUSE	24
228	42.78	42.86	61.56	60.83	sp|Q91VC3|IF4A3_MOUSE	Eukaryotic initiation factor 4A-III OS=Mus musculus OX=10090 GN=Eif4a3 PE=1 SV=3	MOUSE	25
229	42.78	42.84	53.21	44.69	sp|Q8BMS1|ECHA_MOUSE	Trifunctional enzyme subunit alpha, mitochondrial OS=Mus musculus OX=10090 GN=Hadha PE=1 SV=1	MOUSE	23
230	42.59	44.79	50.57	43.96	sp|Q80SW1|SAHH2_MOUSE	S-adenosylhomocysteine hydrolase-like protein 1 OS=Mus musculus OX=10090 GN=Ahcyl1 PE=1 SV=1	MOUSE	27

231	42.48	42.54	41.97	41.69	sp|Q60668|HNRPD_MOUSE	Heterogeneous nuclear ribonucleoprotein D0 OS=Mus musculus OX=10090 GN=Hnrnpd PE=1 SV=2	MOUSE	28
232	42.25	43.12	65.31	58.73	tr|Q543Y7|Q543Y7_MOUSE	Protein kinase C and casein kinase substrate in neurons protein 1 OS=Mus musculus OX=10090 GN=Pacsin1 PE=1 SV=1	MOUSE	23
233	42.22	42.28	61.68	48.90	tr|Q3TMC5|Q3TMC5_MOUSE	Aspartate--tRNA ligase, cytoplasmic OS=Mus musculus OX=10090 GN=Dars PE=2 SV=1	MOUSE	22
234	42.18	42.27	53.89	52.69	sp|P14152|MDHC_MOUSE	Malate dehydrogenase, cytoplasmic OS=Mus musculus OX=10090 GN=Mdh1 PE=1 SV=3	MOUSE	30
235	42.15	42.22	56.14	53.52	sp|Q9CZW5|TOM70_MOUSE	Mitochondrial import receptor subunit TOM70 OS=Mus musculus OX=10090 GN=Tomm70 PE=1 SV=2	MOUSE	29
236	42.08	63.96	51.23	47.70	tr|Q3U741|Q3U741_MOUSE	RNA helicase OS=Mus musculus OX=10090 GN=Ddx17 PE=1 SV=1	MOUSE	33
237	41.8	41.82	37.26	37.26	tr|Q5SZA3|Q5SZA3_MOUSE	Histone cluster 1, H1c OS=Mus musculus OX=10090 GN=H1f2 PE=1 SV=1	MOUSE	40
238	41.75	42.22	32.76	20.04	sp|Q6PB66|LPPRC_MOUSE	Leucine-rich PPR motif-containing protein, mitochondrial OS=Mus musculus OX=10090 GN=Lrpprc PE=1 SV=2	MOUSE	21
239	41.74	41.75	76.54	76.54	sp|P00920|CAH2_MOUSE	Carbonic anhydrase 2 OS=Mus musculus OX=10090 GN=Ca2 PE=1 SV=4	MOUSE	57
240	41.51	41.67	47.99	36.55	tr|Q3UI56|Q3UI56_MOUSE	Annexin OS=Mus musculus OX=10090 GN=Anxa6 PE=2 SV=1	MOUSE	20
241	41.44	41.48	64.72	59.11	sp|Q9Z1N5|DX39B_MOUSE	Spliceosome RNA helicase Ddx39b OS=Mus musculus OX=10090 GN=Ddx39b PE=1 SV=1	MOUSE	35
242	41.35	41.58	57.99	52.51	sp|Q9Z1F9|SAE2_MOUSE	SUMO-activating enzyme subunit 2 OS=Mus musculus OX=10090 GN=Uba2 PE=1 SV=1	MOUSE	25
243	41.15	41.28	56.98	54.92	tr|E9Q1G8|E9Q1G8_MOUSE	Septin OS=Mus musculus OX=10090 GN=Septin7 PE=1 SV=2	MOUSE	24
244	41.1	41.13	49.74	45.29	tr|A0A1B0GR11|A0A1B0GR11_MOUSE	Transaldolase OS=Mus musculus OX=10090 GN=Taldo1 PE=1 SV=1	MOUSE	23
245	41.05	41.11	46.74	34.11	sp|Q9JLV5|CUL3_MOUSE	Cullin-3 OS=Mus musculus OX=10090 GN=Cul3 PE=1 SV=1	MOUSE	20
246	40.98	41.04	68.64	59.96	sp|P17563|SBP1_MOUSE	Methanethiol oxidase OS=Mus musculus OX=10090 GN=Selenbp1 PE=1 SV=2	MOUSE	24

247	40.76	40.78	51.57	44.48	sp|E9Q912|GDS1_MOUSE	Rap1 GTPase-GDP dissociation stimulator 1 OS=Mus musculus OX=10090 GN=Rap1gds1 PE=2 SV=1	MOUSE	23
248	40.7	40.72	52.35	47.27	tr|Q52KG9|Q52KG9_MOUSE	Chaperonin containing Tcp1, subunit 6a (Zeta) OS=Mus musculus OX=10090 GN=Cct6a PE=2 SV=1	MOUSE	22
249	40.69	40.7	65.71	61.32	tr|D3YUM1|D3YUM1_MOUSE	NADH dehydrogenase [ubiquinone] flavoprotein 1, mitochondrial OS=Mus musculus OX=10090 GN=Ndufv1 PE=1 SV=1	MOUSE	22
250	40.5	42.36	38.86	31.51	sp|Q8BFY9|TNPO1_MOUSE	Transportin-1 OS=Mus musculus OX=10090 GN=Tnpo1 PE=1 SV=2	MOUSE	22
251	40.48	40.56	36.78	31.61	tr|Q3TXV1|Q3TXV1_MOUSE	26S proteasome non-ATPase regulatory subunit 2 OS=Mus musculus OX=10090 GN=Psmd2 PE=2 SV=1	MOUSE	22
252	40.42	40.45	68.63	65.33	tr|Q3TQP7|Q3TQP7_MOUSE	Uncharacterized protein OS=Mus musculus OX=10090 GN=Acat1 PE=2 SV=1	MOUSE	21
253	40.25	41.94	70.22	63.01	sp|P48036|ANXA5_MOUSE	Annexin A5 OS=Mus musculus OX=10090 GN=Anxa5 PE=1 SV=1	MOUSE	21
254	40.24	40.27	48.79	42.16	sp|Q9DB77|QCR2_MOUSE	Cytochrome b-c1 complex subunit 2, mitochondrial OS=Mus musculus OX=10090 GN=Uqcrc2 PE=1 SV=1	MOUSE	22
255	39.92	46.83	46.03	39.87	sp|P47857|PFKAM_MOUSE	ATP-dependent 6-phosphofructokinase, muscle type OS=Mus musculus OX=10090 GN=Pfkm PE=1 SV=3	MOUSE	25
256	39.89	40.02	37.44	29.91	tr|Q7TSZ6|Q7TSZ6_MOUSE	Karyopherin (Importin) beta 1 OS=Mus musculus OX=10090 GN=Kpnb1 PE=2 SV=1	MOUSE	21
257	39.89	39.9	56.44	47.33	sp|Q99LF4|RTCB_MOUSE	RNA-splicing ligase RtcB homolog OS=Mus musculus OX=10090 GN=Rtcb PE=1 SV=1	MOUSE	22
258	39.88	40.84	65.38	58.06	tr|A0A0A6YX18|A0A0A6YX18_MOUSE	V-type proton ATPase subunit H OS=Mus musculus OX=10090 GN=Atp6v1h PE=1 SV=1	MOUSE	21
259	39.84	39.91	56.84	55.16	sp|Q9D1A2|CNDP2_MOUSE	Cytosolic non-specific dipeptidase OS=Mus musculus OX=10090 GN=Cndp2 PE=1 SV=1	MOUSE	22
260	39.82	41.79	31.83	21.06	tr|Q790I0|Q790I0_MOUSE	Valyl-tRNA synthetase OS=Mus musculus OX=10090 GN=Vars PE=1 SV=1	MOUSE	20
261	39.8	39.82	62.08	60.00	sp|Q9CZ13|QCR1_MOUSE	Cytochrome b-c1 complex subunit 1, mitochondrial OS=Mus musculus OX=10090 GN=Uqcrc1 PE=1 SV=2	MOUSE	28
262	39.6	51.86	56.03	51.21	tr|Q5EBP8|Q5EBP8_MOUSE	Helix-destabilizing protein OS=Mus musculus OX=10090 GN=Hnrnpa1 PE=1 SV=1	MOUSE	30

263	39.6	43.68	77.14	65.31	sp|P63101|1433Z_MOUSE	14-3-3 protein zeta/delta OS=Mus musculus OX=10090 GN=Ywhaz PE=1 SV=1	MOUSE	27
264	39.53	39.56	49.17	42.98	tr|Q3UUR0|Q3UUR0_MOUSE	Uncharacterized protein OS=Mus musculus OX=10090 GN=Rgs9 PE=2 SV=1	MOUSE	22
265	39.38	39.45	47.83	41.39	sp|Q8BMK4|CKAP4_MOUSE	Cytoskeleton-associated protein 4 OS=Mus musculus OX=10090 GN=Ckap4 PE=1 SV=2	MOUSE	21
266	39.38	39.37	73.83	73.15	sp|P28663|SNAB_MOUSE	Beta-soluble NSF attachment protein OS=Mus musculus OX=10090 GN=Napb PE=1 SV=2	MOUSE	22
267	39.36	43.53	64.27	53.70	tr|Q4FK11|Q4FK11_MOUSE	Nono protein OS=Mus musculus OX=10090 GN=Nono PE=2 SV=1	MOUSE	24
268	39.33	40.08	43.90	37.21	tr|Q05C56|Q05C56_MOUSE	Add3 protein (Fragment) OS=Mus musculus OX=10090 GN=Add3 PE=2 SV=1	MOUSE	22
269	39.32	39.54	29.69	23.58	tr|Q3TCQ3|Q3TCQ3_MOUSE	Pyruvate carboxylase OS=Mus musculus OX=10090 GN=Pcx PE=2 SV=1	MOUSE	20
270	38.99	39.02	71.15	59.13	tr|B2MWM9|B2MWM9_MOUSE	Calreticulin OS=Mus musculus OX=10090 GN=Calr PE=1 SV=1	MOUSE	21
271	38.92	38.98	43.55	32.84	sp|Q3TXS7|PSMD1_MOUSE	26S proteasome non-ATPase regulatory subunit 1 OS=Mus musculus OX=10090 GN=Psmd1 PE=1 SV=1	MOUSE	22
272	38.73	38.86	52.36	36.65	tr|A0A338P6I6|A0A338P6I6_MOUSE	Disks large homolog 4 OS=Mus musculus OX=10090 GN=Dlg4 PE=1 SV=1	MOUSE	21
273	38.71	38.85	40.63	26.67	sp|P58281|OPA1_MOUSE	Dynamin-like 120 kDa protein, mitochondrial OS=Mus musculus OX=10090 GN=Opa1 PE=1 SV=1	MOUSE	19
274	38.44	38.52	62.59	54.44	sp|Q80TB8|VAT1L_MOUSE	Synaptic vesicle membrane protein VAT-1 homolog-like OS=Mus musculus OX=10090 GN=Vat1l PE=1 SV=2	MOUSE	21
275	38.37	38.38	50.78	39.72	sp|Q8BMF4|ODP2_MOUSE	Dihydrolipoyllysine-residue acetyltransferase component of pyruvate dehydrogenase complex, mitochondrial OS=Mus musculus OX=10090 GN=Dlat PE=1 SV=2	MOUSE	20
276	38.24	38.55	35.67	24.33	tr|G3X914|G3X914_MOUSE	Cullin-5 OS=Mus musculus OX=10090 GN=Cul5 PE=1 SV=1	MOUSE	20
277	38.24	38.29	29.65	20.97	tr|Q3UID0|Q3UID0_MOUSE	SWI/SNF complex subunit SMARCC2 OS=Mus musculus OX=10090 GN=Smarcc2 PE=1 SV=1	MOUSE	21
278	38.2	38.28	51.94	46.77	sp|Q9CZU6|CISY_MOUSE	Citrate synthase, mitochondrial OS=Mus musculus OX=10090 GN=Cs PE=1 SV=1	MOUSE	24

279	38.14	38.17	79.40	75.88	sp|P35700|PRDX1_MOUSE	Peroxiredoxin-1 OS=Mus musculus OX=10090 GN=Prdx1 PE=1 SV=1	MOUSE	26
280	38.1	38.12	58.97	56.67	tr|Q3UFJ3|Q3UFJ3_MOUSE	Pyruvate dehydrogenase E1 component subunit alpha OS=Mus musculus OX=10090 GN=Pdha1 PE=1 SV=1	MOUSE	20
281	37.96	38	81.07	74.27	sp|P60879|SNP25_MOUSE	Synaptosomal-associated protein 25 OS=Mus musculus OX=10090 GN=Snap25 PE=1 SV=1	MOUSE	24
282	37.74	37.8	38.50	32.97	sp|Q8VDP4|CCAR2_MOUSE	Cell cycle and apoptosis regulator protein 2 OS=Mus musculus OX=10090 GN=Ccar2 PE=1 SV=2	MOUSE	22
283	37.65	41.69	51.97	51.23	tr|Q5F2A7|Q5F2A7_MOUSE	RNA helicase OS=Mus musculus OX=10090 GN=Eif4a1 PE=1 SV=1	MOUSE	25
284	37.62	37.68	23.59	15.16	tr|A2AGR0|A2AGR0_MOUSE	MAP kinase-activating death domain protein OS=Mus musculus OX=10090 GN=Madd PE=1 SV=1	MOUSE	19
285	37.6	37.7	33.57	22.07	tr|Q08EB6|Q08EB6_MOUSE	CLIP-associating protein 2 OS=Mus musculus OX=10090 GN=Clasp2 PE=1 SV=1	MOUSE	20
286	37.36	37.37	43.60	39.73	tr|Q4VA93|Q4VA93_MOUSE	Protein kinase C OS=Mus musculus OX=10090 GN=Prkca PE=1 SV=1	MOUSE	22
287	37.28	37.33	57.38	57.38	sp|Q3URD3-4|SLMAP_MOUSE	Isoform 4 of Sarcolemmal membrane-associated protein OS=Mus musculus OX=10090 GN=Slmap	MOUSE	25
288	37.22	37.27	89.68	83.33	sp|Q8CGP2|H2B1P_MOUSE	Histone H2B type 1-P OS=Mus musculus OX=10090 GN=Hist1h2bp PE=1 SV=3	MOUSE	63
289	37.2	41.41	68.08	62.71	tr|Q543S2|Q543S2_MOUSE	Uncharacterized protein OS=Mus musculus OX=10090 GN=Gnao1 PE=2 SV=1	MOUSE	24
290	37.18	37.22	56.35	45.01	sp|Q8C854|MYEF2_MOUSE	Myelin expression factor 2 OS=Mus musculus OX=10090 GN=Myef2 PE=1 SV=1	MOUSE	19
291	37.06	37.17	38.16	28.05	sp|Q3UHJ0|AAK1_MOUSE	AP2-associated protein kinase 1 OS=Mus musculus OX=10090 GN=Aak1 PE=1 SV=2	MOUSE	22
292	37.06	37.08	57.42	51.54	tr|Q3U995|Q3U995_MOUSE	Phosphate carrier protein, mitochondrial OS=Mus musculus OX=10090 GN=Slc25a3 PE=2 SV=1	MOUSE	21
293	36.81	36.86	46.88	44.48	tr|Q3U590|Q3U590_MOUSE	SMP_C2CD2L domain-containing protein OS=Mus musculus OX=10090 GN=C2cd2l PE=2 SV=1	MOUSE	23
294	36.79	36.82	81.40	73.05	sp|Q9QYG0|NDRG2_MOUSE	Protein NDRG2 OS=Mus musculus OX=10090 GN=Ndrg2 PE=1 SV=1	MOUSE	21
295	36.64	36.65	58.30	51.91	tr|Q3UCC6|Q3UCC6_MOUSE	Succinate-CoA ligase subunit beta (Fragment) OS=Mus musculus OX=10090 GN=Sucla2 PE=2 SV=1	MOUSE	23

296	36.54	36.6	50.52	41.21	sp|Q9D6Z1|NOP56_MOUSE	Nucleolar protein 56 OS=Mus musculus OX=10090 GN=Nop56 PE=1 SV=2	MOUSE	20
297	36.51	36.73	47.64	39.03	sp|Q9CZW4|ACSL3_MOUSE	Long-chain-fatty-acid--CoA ligase 3 OS=Mus musculus OX=10090 GN=Acsl3 PE=1 SV=2	MOUSE	19
298	36.45	36.49	73.25	68.31	tr|Q5YLW3|Q5YLW3_MOUSE	40S ribosomal protein S3 OS=Mus musculus OX=10090 GN=Rps3 PE=1 SV=1	MOUSE	19
299	36.44	40.29	42.06	42.06	tr|Q3TUE1|Q3TUE1_MOUSE	Far upstream element-binding protein 1 OS=Mus musculus OX=10090 GN=Fubp1 PE=1 SV=1	MOUSE	21
300	36.36	39.43	62.57	53.59	sp|P16125|LDHB_MOUSE	L-lactate dehydrogenase B chain OS=Mus musculus OX=10090 GN=Ldhb PE=1 SV=2	MOUSE	22
301	36.3	37.16	62.44	54.73	tr|Q8K1M3|Q8K1M3_MOUSE	Protein kinase, cAMP dependent regulatory, type II alpha OS=Mus musculus OX=10090 GN=Prkar2a PE=1 SV=1	MOUSE	20
302	36.29	36.32	85.37	85.37	tr|Q5SVY2|Q5SVY2_MOUSE	Peptidyl-prolyl cis-trans isomerase OS=Mus musculus OX=10090 GN=Ppia PE=1 SV=1	MOUSE	38
303	36.24	36.27	53.82	52.08	sp|P61264|STX1B_MOUSE	Syntaxin-1B OS=Mus musculus OX=10090 GN=Stx1b PE=1 SV=1	MOUSE	18
304	36.12	36.21	31.81	21.60	sp|Q8BKC5|IPO5_MOUSE	Importin-5 OS=Mus musculus OX=10090 GN=Ipo5 PE=1 SV=3	MOUSE	20
305	36.12	36.16	57.52	46.68	sp|P06801|MAOX_MOUSE	NADP-dependent malic enzyme OS=Mus musculus OX=10090 GN=Me1 PE=1 SV=2	MOUSE	19
306	36.04	37.96	45.78	37.05	tr|D3Z041|D3Z041_MOUSE	Long-chain-fatty-acid--CoA ligase 1 OS=Mus musculus OX=10090 GN=Acsl1 PE=1 SV=1	MOUSE	22
307	35.93	36	54.02	50.80	tr|Q5FWI9|Q5FWI9_MOUSE	AP-2 complex subunit mu OS=Mus musculus OX=10090 GN=Ap2m1 PE=1 SV=1	MOUSE	18
308	35.78	35.82	76.17	62.09	tr|B2RXY7|B2RXY7_MOUSE	Carbonyl reductase 1 OS=Mus musculus OX=10090 GN=Cbr1 PE=1 SV=1	MOUSE	18
309	35.63	35.7	37.37	29.48	tr|Q8VDC3|Q8VDC3_MOUSE	Citrate hydro-lyase OS=Mus musculus OX=10090 GN=aco1 PE=3 SV=1	MOUSE	20
310	35.53	35.55	95.24	95.24	tr|A8DUK4|A8DUK4_MOUSE	Beta-globin OS=Mus musculus OX=10090 GN=Hbb-bs PE=1 SV=1	MOUSE	24
311	35.48	35.52	60.11	52.46	sp|Q9D6R2|IDH3A_MOUSE	Isocitrate dehydrogenase [NAD] subunit alpha, mitochondrial OS=Mus musculus OX=10090 GN=Idh3a PE=1 SV=1	MOUSE	21

312	35.45	35.47	94.65	93.05	tr|Q5EBQ2|Q5EBQ2_MOUSE	Phosphatidylethanolamine binding protein 1 OS=Mus musculus OX=10090 GN=Pebp1 PE=1 SV=1	MOUSE	29
313	35.43	35.52	65.47	42.11	tr|Q8BVQ9|Q8BVQ9_MOUSE	26S proteasome AAA-ATPase subunit RPT1 OS=Mus musculus OX=10090 GN=Psmc2 PE=1 SV=1	MOUSE	17
314	35.36	35.44	60.06	47.49	sp|P63085|MK01_MOUSE	Mitogen-activated protein kinase 1 OS=Mus musculus OX=10090 GN=Mapk1 PE=1 SV=3	MOUSE	19
315	35.36	35.38	81.36	78.18	tr|Q0PD63|Q0PD63_MOUSE	Ras-related protein Rab-3 OS=Mus musculus OX=10090 GN=Rab3a PE=1 SV=1	MOUSE	22
316	35.19	35.3	33.54	21.76	tr|F8VPX1|F8VPX1_MOUSE	Ubiquitin carboxyl-terminal hydrolase 7 OS=Mus musculus OX=10090 GN=Usp7 PE=1 SV=1	MOUSE	17
317	35.17	35.36	18.21	11.13	sp|Q5SWU9|ACACA_MOUSE	Acetyl-CoA carboxylase 1 OS=Mus musculus OX=10090 GN=Acaca PE=1 SV=1	MOUSE	17
318	35.17	35.21	51.08	45.19	sp|O08749|DLDH_MOUSE	Dihydrolipoyl dehydrogenase, mitochondrial OS=Mus musculus OX=10090 GN=Dld PE=1 SV=2	MOUSE	20
319	35.16	35.29	24.18	16.67	sp|P23116|EIF3A_MOUSE	Eukaryotic translation initiation factor 3 subunit A OS=Mus musculus OX=10090 GN=Eif3a PE=1 SV=5	MOUSE	20
320	35.16	35.2	58.11	53.24	tr|Q543K5|Q543K5_MOUSE	Phosphoserine aminotransferase OS=Mus musculus OX=10090 GN=Psat1 PE=1 SV=1	MOUSE	18
321	35.15	35.19	29.27	26.46	tr|A0A668KM31|A0A668KM31_MOUSE	Amphiphysin OS=Mus musculus OX=10090 GN=Amph PE=1 SV=1	MOUSE	19
322	35.03	35.07	85.71	85.71	tr|Q6GT24|Q6GT24_MOUSE	Peroxiredoxin-6 OS=Mus musculus OX=10090 GN=Prdx6 PE=1 SV=1	MOUSE	24
323	34.94	35.02	65.93	64.60	sp|P50518|VATE1_MOUSE	V-type proton ATPase subunit E 1 OS=Mus musculus OX=10090 GN=Atp6v1e1 PE=1 SV=2	MOUSE	20
324	34.77	34.91	41.83	27.15	sp|Q9D0R2|SYTC_MOUSE	Threonine--tRNA ligase 1, cytoplasmic OS=Mus musculus OX=10090 GN=Tars1 PE=1 SV=2	MOUSE	16
325	34.74	34.84	54.33	46.55	tr|Q768S5|Q768S5_MOUSE	Rabphilin OS=Mus musculus OX=10090 GN=Rph3a PE=2 SV=1	MOUSE	19
326	34.68	34.72	55.33	53.74	tr|F6RJV6|F6RJV6_MOUSE	LanC-like protein 2 (Fragment) OS=Mus musculus OX=10090 GN=Lancl2 PE=1 SV=1	MOUSE	18
327	34.6	34.64	40.28	34.79	tr|Q543B9|Q543B9_MOUSE	Prolyl endopeptidase OS=Mus musculus OX=10090 GN=Prep PE=1 SV=1	MOUSE	18
328	34.46	34.52	63.29	52.95	tr|Q3UVJ2|Q3UVJ2_MOUSE	Adenylyl cyclase-associated protein OS=Mus musculus OX=10090 GN=Cap1 PE=2 SV=1	MOUSE	18

329	34.45	34.49	47.80	41.87	tr|Q3TWG5|Q3TWG5_MOUSE	Dynein light intermediate chain OS=Mus musculus OX=10090 GN=Dync1li1 PE=1 SV=1	MOUSE	17
330	34.43	39.32	62.44	56.70	tr|Q545N7|Q545N7_MOUSE	Creatine kinase OS=Mus musculus OX=10090 GN=Ckmt1 PE=1 SV=1	MOUSE	21
331	34.37	34.42	58.82	44.65	tr|Q6P5I3|Q6P5I3_MOUSE	S-(hydroxymethyl)glutathione dehydrogenase OS=Mus musculus OX=10090 GN=Adh5 PE=1 SV=2	MOUSE	18
332	34.33	36.08	43.25	38.29	sp|O35841|API5_MOUSE	Apoptosis inhibitor 5 OS=Mus musculus OX=10090 GN=Api5 PE=1 SV=2	MOUSE	20
333	34.31	34.35	50.20	47.85	tr|Q3U6F6|Q3U6F6_MOUSE	Seryl-tRNA synthetase OS=Mus musculus OX=10090 GN=Sars PE=2 SV=1	MOUSE	20
334	34.28	34.42	58.18	53.62	tr|Q91V28|Q91V28_MOUSE	6-phosphogluconate dehydrogenase, decarboxylating OS=Mus musculus OX=10090 GN=Pgd PE=2 SV=1	MOUSE	19
335	34.24	34.32	47.12	42.86	sp|Q9D394|RUFY3_MOUSE	Protein RUFY3 OS=Mus musculus OX=10090 GN=Rufy3 PE=1 SV=1	MOUSE	19
336	34.17	34.25	64.66	50.94	tr|Q4FJZ4|Q4FJZ4_MOUSE	T1-TrpRS OS=Mus musculus OX=10090 GN=Wars PE=2 SV=1	MOUSE	19
337	33.97	34	63.27	60.86	tr|A0A0R3P9C8|A0A0R3P9C8_MOUSE	NADH dehydrogenase [ubiquinone] 1 alpha subcomplex subunit 9, mitochondrial OS=Mus musculus OX=10090 GN=Ndufa9 PE=1 SV=1	MOUSE	17
338	33.96	34.41	42.47	33.33	tr|A0A498WGK2|A0A498WGK2_MOUSE	Asparagine--tRNA ligase OS=Mus musculus OX=10090 GN=Nars PE=1 SV=1	MOUSE	17
339	33.95	33.97	86.41	85.33	tr|A5GZX3|A5GZX3_MOUSE	Lactoylglutathione lyase OS=Mus musculus OX=10090 GN=Glo1 PE=1 SV=1	MOUSE	31
340	33.79	48.67	46.63	38.12	tr|B2RXT3|B2RXT3_MOUSE	Oxoglutarate dehydrogenase (succinyl-transferring) OS=Mus musculus OX=10090 GN=Ogdhl PE=1 SV=1	MOUSE	26
341	33.72	33.74	65.61	61.15	tr|Q5SX53|Q5SX53_MOUSE	Uncharacterized protein OS=Mus musculus OX=10090 GN=Slc25a11 PE=1 SV=1	MOUSE	20
342	33.71	33.76	78.55	76.03	sp|P68040|RACK1_MOUSE	Receptor of activated protein C kinase 1 OS=Mus musculus OX=10090 GN=Rack1 PE=1 SV=3	MOUSE	20
343	33.51	33.52	26.95	21.97	sp|Q9JIS5|SV2A_MOUSE	Synaptic vesicle glycoprotein 2A OS=Mus musculus OX=10090 GN=Sv2a PE=1 SV=1	MOUSE	22
344	33.36	38.69	14.00	10.98	tr|A2ARP8|A2ARP8_MOUSE	Microtubule-associated protein 1A OS=Mus musculus OX=10090 GN=Map1a PE=1 SV=1	MOUSE	22

345	33.31	33.73	26.45	17.70	sp|Q91ZW3|SMCA5_MOUSE	SWI/SNF-related matrix-associated actin-dependent regulator of chromatin subfamily A member 5 OS=Mus musculus OX=10090 GN=Smarca5 PE=1 SV=1	MOUSE	17
346	33.15	33.17	47.03	46.61	tr|Q8C2Q7|Q8C2Q7_MOUSE	Heterogeneous nuclear ribonucleoprotein H OS=Mus musculus OX=10090 GN=Hnrnph1 PE=1 SV=1	MOUSE	22
347	33.06	33.17	26.16	19.96	sp|Q9JIX8|ACINU_MOUSE	Apoptotic chromatin condensation inducer in the nucleus OS=Mus musculus OX=10090 GN=Acin1 PE=1 SV=3	MOUSE	21
348	32.97	32.99	64.54	51.25	sp|Q3UM45|PP1R7_MOUSE	Protein phosphatase 1 regulatory subunit 7 OS=Mus musculus OX=10090 GN=Ppp1r7 PE=1 SV=2	MOUSE	16
349	32.86	32.88	50.54	45.14	sp|Q91WD5|NDUS2_MOUSE	NADH dehydrogenase [ubiquinone] iron-sulfur protein 2, mitochondrial OS=Mus musculus OX=10090 GN=Ndufs2 PE=1 SV=1	MOUSE	18
350	32.74	32.79	56.54	47.88	sp|Q9D0K2|SCOT1_MOUSE	Succinyl-CoA:3-ketoacid coenzyme A transferase 1, mitochondrial OS=Mus musculus OX=10090 GN=Oxct1 PE=1 SV=1	MOUSE	18
351	32.54	32.56	27.43	22.74	sp|Q99MR6|SRRT_MOUSE	Serrate RNA effector molecule homolog OS=Mus musculus OX=10090 GN=Srrt PE=1 SV=1	MOUSE	17
352	32.52	32.53	37.71	34.29	tr|Q49S98|Q49S98_MOUSE	Vasicular inhibitory amino acid transporter 10D OS=Mus musculus OX=10090 GN=Slc32a1 PE=2 SV=1	MOUSE	18
353	32.47	32.5	51.15	46.55	sp|Q9WV02|RBMX_MOUSE	RNA-binding motif protein, X chromosome OS=Mus musculus OX=10090 GN=Rbmx PE=1 SV=1	MOUSE	18
354	32.43	32.46	81.74	80.06	sp|P60335|PCBP1_MOUSE	Poly(rC)-binding protein 1 OS=Mus musculus OX=10090 GN=Pcbp1 PE=1 SV=1	MOUSE	24
355	32.38	32.39	74.49	56.49	tr|Q3TG75|Q3TG75_MOUSE	Ornithine--oxo-acid aminotransferase OS=Mus musculus OX=10090 GN=Oat PE=1 SV=1	MOUSE	18
356	32.35	32.35	40.49	40.49	sp|O35381|AN32A_MOUSE	Acidic leucine-rich nuclear phosphoprotein 32 family member A OS=Mus musculus OX=10090 GN=Anp32a PE=1 SV=1	MOUSE	18
357	32.29	32.37	29.35	17.56	sp|Q3UPL0|SC31A_MOUSE	Protein transport protein Sec31A OS=Mus musculus OX=10090 GN=Sec31a PE=1 SV=2	MOUSE	16
358	32.26	32.26	66.80	66.80	sp|Q9QW08|PHOS_MOUSE	Phosducin OS=Mus musculus OX=10090 GN=Pdc PE=2 SV=1	MOUSE	21
359	32.22	32.23	56.61	47.11	tr|Q3TMB8|Q3TMB8_MOUSE	Adenylosuccinate lyase OS=Mus musculus OX=10090 GN=Adsl PE=2 SV=1	MOUSE	16

360	32.2	32.22	62.23	61.97	sp|Q8R5C5|ACTY_MOUSE	Beta-centractin OS=Mus musculus OX=10090 GN=Actr1b PE=1 SV=1	MOUSE	19
361	32.19	32.23	38.83	35.90	sp|Q9D517|PLCC_MOUSE	1-acyl-sn-glycerol-3-phosphate acyltransferase gamma OS=Mus musculus OX=10090 GN=Agpat3 PE=1 SV=2	MOUSE	19
362	32.1	32.34	37.36	27.70	sp|Q9JIF7|COPB_MOUSE	Coatomer subunit beta OS=Mus musculus OX=10090 GN=Copb1 PE=1 SV=1	MOUSE	20
363	32.05	32.11	28.76	20.10	tr|Q8CIJ3|Q8CIJ3_MOUSE	Eukaryotic translation initiation factor 3 subunit B OS=Mus musculus OX=10090 GN=Eif3b PE=2 SV=1	MOUSE	18
364	32.04	32.07	47.33	43.62	tr|A2ALV3|A2ALV3_MOUSE	Endophilin-A1 OS=Mus musculus OX=10090 GN=Sh3gl2 PE=1 SV=1	MOUSE	19
365	32	32	58.59	58.59	tr|Q5I0W0|Q5I0W0_MOUSE	ATP synthase subunit b OS=Mus musculus OX=10090 GN=Atp5pb PE=1 SV=1	MOUSE	17
366	31.97	31.99	61.02	60.00	sp|Q60930|VDAC2_MOUSE	Voltage-dependent anion-selective channel protein 2 OS=Mus musculus OX=10090 GN=Vdac2 PE=1 SV=2	MOUSE	25
367	31.9	32.04	32.71	19.48	sp|Q920I9|WDR7_MOUSE	WD repeat-containing protein 7 OS=Mus musculus OX=10090 GN=Wdr7 PE=1 SV=3	MOUSE	17
368	31.86	31.99	38.25	27.61	sp|Q8R0Y6|AL1L1_MOUSE	Cytosolic 10-formyltetrahydrofolate dehydrogenase OS=Mus musculus OX=10090 GN=Aldh1l1 PE=1 SV=1	MOUSE	20
369	31.85	31.88	43.13	36.87	tr|Q564E6|Q564E6_MOUSE	Ssb protein OS=Mus musculus OX=10090 GN=Ssb PE=1 SV=1	MOUSE	18
370	31.81	32.47	72.41	57.14	tr|Q14AI7|Q14AI7_MOUSE	COP9 signalosome complex subunit 4 OS=Mus musculus OX=10090 GN=Cops4 PE=1 SV=1	MOUSE	17
371	31.73	31.8	28.75	24.24	tr|B2RSC8|B2RSC8_MOUSE	E3 ubiquitin-protein ligase OS=Mus musculus OX=10090 GN=Nedd4 PE=1 SV=1	MOUSE	17
372	31.7	31.78	20.63	14.51	sp|Q6P5E4|UGGG1_MOUSE	UDP-glucose:glycoprotein glucosyltransferase 1 OS=Mus musculus OX=10090 GN=Uggt1 PE=1 SV=4	MOUSE	16
373	31.67	31.8	35.59	25.55	sp|Q04750|TOP1_MOUSE	DNA topoisomerase 1 OS=Mus musculus OX=10090 GN=Top1 PE=1 SV=2	MOUSE	19
374	31.61	32.01	50.26	27.56	sp|P26041|MOES_MOUSE	Moesin OS=Mus musculus OX=10090 GN=Msn PE=1 SV=3	MOUSE	16
375	31.58	31.64	64.64	59.70	tr|Q545X8|Q545X8_MOUSE	40S ribosomal protein S4 OS=Mus musculus OX=10090 GN=Rps4x PE=1 SV=1	MOUSE	18
376	31.48	33.62	31.96	25.42	tr|A0A571BEK3|A0A571BEK3_MOUSE	Disks large homolog 2 OS=Mus musculus OX=10090 GN=Dlg2 PE=1 SV=1	MOUSE	18

377	31.47	31.65	31.35	24.71	sp|Q9WU78-3|PDC6I_MOUSE	Isoform 3 of Programmed cell death 6-interacting protein OS=Mus musculus OX=10090 GN=Pdcd6ip	MOUSE	18
378	31.34	31.36	53.70	45.14	tr|A0A0R4J0B4|A0A0R4J0B4_MOUSE	CMP-N-acetylneuraminic acid synthase OS=Mus musculus OX=10090 GN=Cmas PE=1 SV=1	MOUSE	16
379	31.28	31.81	29.79	17.43	sp|Q8BU30|SYIC_MOUSE	Isoleucine--tRNA ligase, cytoplasmic OS=Mus musculus OX=10090 GN=Iars1 PE=1 SV=2	MOUSE	20
380	31.23	31.54	69.25	64.68	sp|Q99KP6|PRP19_MOUSE	Pre-mRNA-processing factor 19 OS=Mus musculus OX=10090 GN=Prpf19 PE=1 SV=1	MOUSE	18
381	31.22	31.26	78.26	78.26	tr|Q4FJQ0|Q4FJQ0_MOUSE	Rab7 OS=Mus musculus OX=10090 GN=Rab7 PE=1 SV=1	MOUSE	17
382	31.11	31.21	27.46	20.52	sp|Q9EPL8|IPO7_MOUSE	Importin-7 OS=Mus musculus OX=10090 GN=Ipo7 PE=1 SV=2	MOUSE	15
383	31.02	31.04	59.74	50.77	tr|Q3UXI9|Q3UXI9_MOUSE	DZF domain-containing protein OS=Mus musculus OX=10090 GN=Ilf2 PE=2 SV=1	MOUSE	17
384	30.96	35.08	61.63	56.73	tr|A3KML3|A3KML3_MOUSE	14-3-3 protein theta OS=Mus musculus OX=10090 GN=Ywhaq PE=2 SV=1	MOUSE	20
385	30.95	31.08	57.49	50.48	sp|Q9CZC8|SCRN1_MOUSE	Secernin-1 OS=Mus musculus OX=10090 GN=Scrn1 PE=1 SV=1	MOUSE	18
386	30.94	30.99	45.34	40.81	tr|Q3TA40|Q3TA40_MOUSE	RIKEN cDNA 6430548M08 gene OS=Mus musculus OX=10090 GN=6430548M08Rik PE=1 SV=1	MOUSE	15
387	30.91	41.71	22.50	12.71	tr|Q3UGY4|Q3UGY4_MOUSE	Spectrin beta chain OS=Mus musculus OX=10090 GN=Sptb PE=2 SV=1	MOUSE	21
388	30.88	33.08	49.12	42.26	sp|P54071|IDHP_MOUSE	Isocitrate dehydrogenase [NADP], mitochondrial OS=Mus musculus OX=10090 GN=Idh2 PE=1 SV=3	MOUSE	18
389	30.86	30.93	60.47	51.38	tr|H7BX95|H7BX95_MOUSE	Serine/arginine-rich splicing factor 1 OS=Mus musculus OX=10090 GN=Srsf1 PE=1 SV=1	MOUSE	17
390	30.85	30.86	45.41	40.82	tr|Q9CSH0|Q9CSH0_MOUSE	Uncharacterized protein (Fragment) OS=Mus musculus OX=10090 GN=Hnrnpll PE=2 SV=1	MOUSE	16
391	30.76	30.79	88.82	80.12	sp|Q9DCX2|ATP5H_MOUSE	ATP synthase subunit d, mitochondrial OS=Mus musculus OX=10090 GN=Atp5pd PE=1 SV=3	MOUSE	19
392	30.72	30.74	42.36	38.67	sp|Q9Z273|TULP1_MOUSE	Tubby-related protein 1 OS=Mus musculus OX=10090 GN=Tulp1 PE=1 SV=1	MOUSE	18
393	30.62	31.09	13.02	5.31	sp|Q9QXS1|PLEC_MOUSE	Plectin OS=Mus musculus OX=10090 GN=Plec PE=1 SV=3	MOUSE	17

394	30.62	30.65	65.26	55.79	sp|Q9CWS0|DDAH1_MOUSE	N(G),N(G)-dimethylarginine dimethylaminohydrolase 1 OS=Mus musculus OX=10090 GN=Ddah1 PE=1 SV=3	MOUSE	16
395	30.55	30.66	36.19	28.39	tr|A0A0R4J0Z1|A0A0R4J0Z1_MOUSE	Protein disulfide-isomerase A4 OS=Mus musculus OX=10090 GN=Pdia4 PE=1 SV=1	MOUSE	17
396	30.5	30.57	34.30	27.25	sp|Q4KMM3|OXR1_MOUSE	Oxidation resistance protein 1 OS=Mus musculus OX=10090 GN=Oxr1 PE=1 SV=3	MOUSE	16
397	30.48	30.54	57.14	50.83	sp|Q5XG69|F169A_MOUSE	Soluble lamin-associated protein of 75 kDa OS=Mus musculus OX=10090 GN=Fam169a PE=1 SV=3	MOUSE	19
398	30.47	30.58	36.30	20.17	sp|Q64727|VINC_MOUSE	Vinculin OS=Mus musculus OX=10090 GN=Vcl PE=1 SV=4	MOUSE	15
399	30.45	30.62	27.22	18.11	sp|Q8R1B4|EIF3C_MOUSE	Eukaryotic translation initiation factor 3 subunit C OS=Mus musculus OX=10090 GN=Eif3c PE=1 SV=1	MOUSE	19
400	30.35	30.35	64.94	59.41	sp|Q08331|CALB2_MOUSE	Calretinin OS=Mus musculus OX=10090 GN=Calb2 PE=1 SV=3	MOUSE	18
401	30.31	34.09	28.57	23.58	sp|Q91ZQ1|PDE6C_MOUSE	Cone cGMP-specific 3',5'-cyclic phosphodiesterase subunit alpha' OS=Mus musculus OX=10090 GN=Pde6c PE=2 SV=1	MOUSE	20
402	30.31	30.36	58.85	48.33	tr|Q3TGW0|Q3TGW0_MOUSE	Actin-like protein 3 OS=Mus musculus OX=10090 GN=Actr3 PE=2 SV=1	MOUSE	15
403	30.29	70.1	77.11	74.89	sp|Q9ERD7|TBB3_MOUSE	Tubulin beta-3 chain OS=Mus musculus OX=10090 GN=Tubb3 PE=1 SV=1	MOUSE	77
404	30.26	30.33	13.78	7.87	tr|Q4FE56|Q4FE56_MOUSE	Ubiquitinyl hydrolase 1 OS=Mus musculus OX=10090 GN=Usp9x PE=1 SV=1	MOUSE	18
405	30.23	30.27	48.55	47.40	tr|Q3UWK3|Q3UWK3_MOUSE	Uncharacterized protein OS=Mus musculus OX=10090 GN=Prph2 PE=2 SV=1	MOUSE	26
406	30.2	30.21	62.12	56.06	tr|Q564F3|Q564F3_MOUSE	40S ribosomal protein S3a OS=Mus musculus OX=10090 GN=Rps3a1 PE=1 SV=1	MOUSE	15
407	30.12	30.16	33.70	27.58	tr|E9Q1S3|E9Q1S3_MOUSE	Protein transport protein SEC23 OS=Mus musculus OX=10090 GN=Sec23a PE=1 SV=1	MOUSE	14
408	30.1	30.21	25.89	13.58	tr|E1AZ71|E1AZ71_MOUSE	Cyclic nucleotide-gated channel beta 1 OS=Mus musculus OX=10090 GN=Cngb1 PE=1 SV=1	MOUSE	14
409	30.05	30.13	50.79	44.64	sp|Q8BJY1|PSMD5_MOUSE	26S proteasome non-ATPase regulatory subunit 5 OS=Mus musculus OX=10090 GN=Psmd5 PE=1 SV=4	MOUSE	17

410	30.03	30.08	64.71	64.09	tr|A0A1B0GRV0|A0A1B0GRV0_MOUSE	3'(2'),5'-bisphosphate nucleotidase 1 OS=Mus musculus OX=10090 GN=Bpnt1 PE=1 SV=1	MOUSE	16
411	29.88	29.97	51.42	43.06	sp|Q69Z99|ZN512_MOUSE	Zinc finger protein 512 OS=Mus musculus OX=10090 GN=Znf512 PE=2 SV=2	MOUSE	16
412	29.86	34.24	52.42	50.27	sp|Q8CCK0|H2AW_MOUSE	Core histone macro-H2A.2 OS=Mus musculus OX=10090 GN=Macroh2a2 PE=1 SV=3	MOUSE	18
413	29.84	30.01	31.06	16.43	tr|Q53WR6|Q53WR6_MOUSE	Golgi apparatus protein 1 OS=Mus musculus OX=10090 GN=Glg1 PE=1 SV=1	MOUSE	15
414	29.83	29.92	46.40	30.24	sp|Q8JZN5|ACAD9_MOUSE	Complex I assembly factor ACAD9, mitochondrial OS=Mus musculus OX=10090 GN=Acad9 PE=1 SV=2	MOUSE	18
415	29.83	29.89	57.19	54.52	tr|Q3V235|Q3V235_MOUSE	Prohibitin OS=Mus musculus OX=10090 GN=Phb2 PE=1 SV=1	MOUSE	15
416	29.81	29.91	64.68	50.75	tr|Q3TPZ5|Q3TPZ5_MOUSE	Uncharacterized protein OS=Mus musculus OX=10090 GN=Dctn2 PE=1 SV=1	MOUSE	17
417	29.81	29.86	64.09	61.41	sp|Q9CPV4|GLOD4_MOUSE	Glyoxalase domain-containing protein 4 OS=Mus musculus OX=10090 GN=Glod4 PE=1 SV=1	MOUSE	16
418	29.77	30.15	38.57	27.36	tr|E9QB02|E9QB02_MOUSE	Methionine--tRNA ligase, cytoplasmic OS=Mus musculus OX=10090 GN=Mars1 PE=1 SV=1	MOUSE	18
419	29.72	30	64.45	41.91	tr|Q4FJV4|Q4FJV4_MOUSE	Annexin OS=Mus musculus OX=10090 GN=Anxa1 PE=1 SV=1	MOUSE	17
420	29.66	29.79	48.75	46.01	sp|Q5FWK3|RHG01_MOUSE	Rho GTPase-activating protein 1 OS=Mus musculus OX=10090 GN=Arhgap1 PE=1 SV=1	MOUSE	16
421	29.65	29.71	54.56	45.23	sp|Q61553|FSCN1_MOUSE	Fascin OS=Mus musculus OX=10090 GN=Fscn1 PE=1 SV=4	MOUSE	18
422	29.63	29.81	36.56	29.25	tr|Q3TG12|Q3TG12_MOUSE	Phenylalanyl-tRNA synthetase beta subunit (Fragment) OS=Mus musculus OX=10090 GN=Farsb PE=2 SV=1	MOUSE	16
423	29.6	29.7	49.01	37.66	tr|Q8BMR3|Q8BMR3_MOUSE	Dolichyl-diphosphooligosaccharide--protein glycosyltransferase subunit 1 OS=Mus musculus OX=10090 GN=Rpn1 PE=2 SV=1	MOUSE	16
424	29.6	29.64	55.84	53.94	tr|Q544Z7|Q544Z7_MOUSE	DNA-(apurinic or apyrimidinic site) endonuclease OS=Mus musculus OX=10090 GN=Apex1 PE=1 SV=1	MOUSE	19
425	29.57	29.71	16.53	13.15	sp|Q61191|HCFC1_MOUSE	Host cell factor 1 OS=Mus musculus OX=10090 GN=Hcfc1 PE=1 SV=2	MOUSE	19

426	29.56	29.61	49.04	41.15	sp|Q8BGH2|SAM50_MOUSE	Sorting and assembly machinery component 50 homolog OS=Mus musculus OX=10090 GN=Samm50 PE=1 SV=1	MOUSE	18
427	29.44	29.58	37.36	34.48	sp|P15409|OPSD_MOUSE	Rhodopsin OS=Mus musculus OX=10090 GN=Rho PE=1 SV=2	MOUSE	34
428	29.42	29.56	37.63	29.81	sp|Q9ERL9|GCYA1_MOUSE	Guanylate cyclase soluble subunit alpha-1 OS=Mus musculus OX=10090 GN=Gucy1a1 PE=1 SV=2	MOUSE	14
429	29.35	47.73	55.73	46.09	tr|E9PZ43|E9PZ43_MOUSE	Microtubule-associated protein OS=Mus musculus OX=10090 GN=Map4 PE=1 SV=3	MOUSE	25
430	29.34	29.44	54.77	48.41	tr|Q542I9|Q542I9_MOUSE	AAA domain-containing protein OS=Mus musculus OX=10090 GN=Psmc1 PE=1 SV=1	MOUSE	16
431	29.32	31.19	51.35	39.54	sp|P54823|DDX6_MOUSE	Probable ATP-dependent RNA helicase DDX6 OS=Mus musculus OX=10090 GN=Ddx6 PE=1 SV=1	MOUSE	15
432	29.3	30.48	37.02	22.21	sp|Q9Z1Z0|USO1_MOUSE	General vesicular transport factor p115 OS=Mus musculus OX=10090 GN=Uso1 PE=1 SV=2	MOUSE	16
433	29.25	29.38	50.30	48.70	sp|Q3UEB3-3|PUF60_MOUSE	Isoform 3 of Poly(U)-binding-splicing factor PUF60 OS=Mus musculus OX=10090 GN=Puf60	MOUSE	17
434	29.24	30.33	51.97	37.34	tr|A0A0G2JGS4|A0A0G2JGS4_MOUSE	Calcium/calmodulin-dependent protein kinase OS=Mus musculus OX=10090 GN=Camk2d PE=1 SV=1	MOUSE	18
435	29.2	29.34	45.55	35.63	tr|Q3THE7|Q3THE7_MOUSE	Aldedh domain-containing protein OS=Mus musculus OX=10090 GN=Aldh9a1 PE=2 SV=1	MOUSE	16
436	29.16	29.22	42.33	32.49	tr|Q4FZK2|Q4FZK2_MOUSE	Elongation factor 1-gamma OS=Mus musculus OX=10090 GN=Eef1g PE=1 SV=1	MOUSE	14
437	29.1	29.19	44.14	36.67	sp|Q99KE1|MAOM_MOUSE	NAD-dependent malic enzyme, mitochondrial OS=Mus musculus OX=10090 GN=Me2 PE=1 SV=1	MOUSE	15
438	29.07	29.1	77.29	77.29	tr|E9Q7K1|E9Q7K1_MOUSE	Guanylate kinase OS=Mus musculus OX=10090 GN=Guk1 PE=1 SV=1	MOUSE	17
439	29.06	49.12	30.60	12.75	tr|K3W4R2|K3W4R2_MOUSE	Myosin-14 OS=Mus musculus OX=10090 GN=Myh14 PE=1 SV=1	MOUSE	22
440	29.06	29.21	51.38	42.13	sp|Q8C0C7|SYFA_MOUSE	Phenylalanine--tRNA ligase alpha subunit OS=Mus musculus OX=10090 GN=Farsa PE=1 SV=1	MOUSE	14
441	29.02	29.1	31.62	25.79	sp|Q03717|KCNB1_MOUSE	Potassium voltage-gated channel subfamily B member 1 OS=Mus musculus OX=10090 GN=Kcnb1 PE=1 SV=2	MOUSE	16
442	28.93	31.31	70.37	65.74	sp|O35295|PURB_MOUSE	Transcriptional activator protein Pur-beta OS=Mus musculus OX=10090 GN=Purb PE=1 SV=3	MOUSE	19

443	28.88	28.92	64.92	47.69	sp|Q9JII6|AK1A1_MOUSE	Aldo-keto reductase family 1 member A1 OS=Mus musculus OX=10090 GN=Akr1a1 PE=1 SV=3	MOUSE	16
444	28.8	28.91	65.37	54.88	tr|Q5SW18|Q5SW18_MOUSE	Platelet-activating factor acetylhydrolase IB subunit alpha OS=Mus musculus OX=10090 GN=Pafah1b1 PE=2 SV=1	MOUSE	15
445	28.79	28.81	63.41	55.43	sp|Q91WD9|SEGN_MOUSE	Secretagogin OS=Mus musculus OX=10090 GN=Scgn PE=2 SV=1	MOUSE	15
446	28.76	28.83	52.45	52.15	tr|Q8BM84|Q8BM84_MOUSE	ELAV-like protein OS=Mus musculus OX=10090 GN=Elavl1 PE=2 SV=1	MOUSE	19
447	28.72	28.85	57.14	55.43	sp|Q9R1T2|SAE1_MOUSE	SUMO-activating enzyme subunit 1 OS=Mus musculus OX=10090 GN=Sae1 PE=1 SV=1	MOUSE	17
448	28.71	32.33	39.19	39.19	tr|Q1WWK3|Q1WWK3_MOUSE	Hist1h1b protein (Fragment) OS=Mus musculus OX=10090 GN=H1f5 PE=2 SV=1	MOUSE	25
449	28.71	30.57	29.69	24.33	sp|Q8VDM6|HNRL1_MOUSE	Heterogeneous nuclear ribonucleoprotein U-like protein 1 OS=Mus musculus OX=10090 GN=Hnrnpul1 PE=1 SV=1	MOUSE	16
450	28.7	28.76	46.44	45.06	tr|G3UZM4|G3UZM4_MOUSE	Cell adhesion molecule 2 (Fragment) OS=Mus musculus OX=10090 GN=Cadm2 PE=1 SV=1	MOUSE	19
451	28.62	28.65	58.20	58.20	sp|Q9D6M3|GHC1_MOUSE	Mitochondrial glutamate carrier 1 OS=Mus musculus OX=10090 GN=Slc25a22 PE=1 SV=1	MOUSE	16
452	28.57	28.65	65.37	62.99	sp|Q99L13|3HIDH_MOUSE	3-hydroxyisobutyrate dehydrogenase, mitochondrial OS=Mus musculus OX=10090 GN=Hibadh PE=1 SV=1	MOUSE	17
453	28.49	28.51	76.19	71.43	sp|Q99LX0|PARK7_MOUSE	Parkinson disease protein 7 homolog OS=Mus musculus OX=10090 GN=Park7 PE=1 SV=1	MOUSE	18
454	28.44	28.48	43.91	41.62	tr|Q3UMW2|Q3UMW2_MOUSE	Peptidase_M24 domain-containing protein OS=Mus musculus OX=10090 GN=Pa2g4 PE=2 SV=1	MOUSE	16
455	28.41	28.51	19.33	16.49	sp|Q3TES0|IQEC3_MOUSE	IQ motif and SEC7 domain-containing protein 3 OS=Mus musculus OX=10090 GN=Iqsec3 PE=1 SV=1	MOUSE	16
456	28.37	28.57	58.99	54.97	tr|Q8CAJ7|Q8CAJ7_MOUSE	Aspartyl aminopeptidase OS=Mus musculus OX=10090 GN=Dnpep PE=2 SV=1	MOUSE	18
457	28.36	28.51	58.36	56.09	tr|Q3UIG8|Q3UIG8_MOUSE	Methyltransf_25 domain-containing protein OS=Mus musculus OX=10090 GN=Prmt1 PE=1 SV=1	MOUSE	17
458	28.29	28.43	34.33	25.36	tr|F6ZHD8|F6ZHD8_MOUSE	1,4-alpha-glucan branching enzyme OS=Mus musculus OX=10090 GN=Gbe1 PE=1 SV=2	MOUSE	14
459	28.28	28.41	34.68	25.57	sp|Q3UYV9|NCBP1_MOUSE	Nuclear cap-binding protein subunit 1 OS=Mus musculus OX=10090 GN=Ncbp1 PE=1 SV=2	MOUSE	15

460	28.28	28.29	64.24	49.70	sp|Q91VM9|IPYR2_MOUSE	Inorganic pyrophosphatase 2, mitochondrial OS=Mus musculus OX=10090 GN=Ppa2 PE=1 SV=1	MOUSE	14
461	28.24	28.45	20.80	12.67	tr|O08847|O08847_MOUSE	DNA-directed RNA polymerase subunit OS=Mus musculus OX=10090 GN=Polr2a PE=2 SV=1	MOUSE	16
462	28.2	28.2	66.33	66.33	tr|Q3UWS9|Q3UWS9_MOUSE	Peroxiredoxin-5 (Fragment) OS=Mus musculus OX=10090 GN=Prdx5 PE=2 SV=1	MOUSE	19
463	28.19	28.2	43.16	35.26	tr|Q61833|Q61833_MOUSE	Dolichyl-diphosphooligosaccharide--protein glycosyltransferase subunit 2 OS=Mus musculus OX=10090 GN=Rpn2 PE=2 SV=1	MOUSE	15
464	28.16	28.23	45.81	41.62	tr|Q3U6P5|Q3U6P5_MOUSE	RRM domain-containing protein OS=Mus musculus OX=10090 GN=Hnrnpc PE=2 SV=1	MOUSE	14
465	28.16	28.2	60.55	52.29	sp|P62141|PP1B_MOUSE	Serine/threonine-protein phosphatase PP1-beta catalytic subunit OS=Mus musculus OX=10090 GN=Ppp1cb PE=1 SV=3	MOUSE	15
466	28.13	28.24	19.56	15.77	tr|Q3V1M8|Q3V1M8_MOUSE	Uncharacterized protein OS=Mus musculus OX=10090 GN=Hdlbp PE=2 SV=1	MOUSE	15
467	28.11	28.14	62.94	59.42	tr|Q3UPX0|Q3UPX0_MOUSE	Ketimine reductase mu-crystallin OS=Mus musculus OX=10090 GN=Crym PE=1 SV=1	MOUSE	17
468	28.1	28.35	59.24	41.23	tr|Q5BKQ9|Q5BKQ9_MOUSE	Proteasome (Prosome, macropain) 26S subunit, non-ATPase, 11 OS=Mus musculus OX=10090 GN=Psmd11 PE=1 SV=1	MOUSE	17
469	28.08	28.21	56.51	53.02	sp|Q6ZWX6|IF2A_MOUSE	Eukaryotic translation initiation factor 2 subunit 1 OS=Mus musculus OX=10090 GN=Eif2s1 PE=1 SV=3	MOUSE	15
470	28.06	30.2	54.72	45.47	tr|Q544B1|Q544B1_MOUSE	Aldedh domain-containing protein OS=Mus musculus OX=10090 GN=Aldh2 PE=1 SV=1	MOUSE	17
471	28.06	28.26	32.03	23.62	sp|Q9JKR6|HYOU1_MOUSE	Hypoxia up-regulated protein 1 OS=Mus musculus OX=10090 GN=Hyou1 PE=1 SV=1	MOUSE	16
472	28.05	28.1	81.93	80.12	tr|Q544Y7|Q544Y7_MOUSE	Cofilin, non-muscle isoform OS=Mus musculus OX=10090 GN=Cfl1 PE=2 SV=1	MOUSE	15
473	28.02	28.16	41.78	34.38	tr|B1AU25|B1AU25_MOUSE	Apoptosis-inducing factor 1, mitochondrial OS=Mus musculus OX=10090 GN=Aifm1 PE=1 SV=1	MOUSE	14
474	28.01	28.04	64.06	56.58	tr|Q2UZW7|Q2UZW7_MOUSE	Microtubule-associated protein OS=Mus musculus OX=10090 GN=Mapre3 PE=1 SV=1	MOUSE	16

475	27.89	27.96	34.04	22.55	sp|Q8CGK3|LONM_MOUSE	Lon protease homolog, mitochondrial OS=Mus musculus OX=10090 GN=Lonp1 PE=1 SV=2	MOUSE	15
476	27.88	38.42	82.03	81.02	sp|Q9DB05|SNAA_MOUSE	Alpha-soluble NSF attachment protein OS=Mus musculus OX=10090 GN=Napa PE=1 SV=1	MOUSE	22
477	27.82	27.85	66.54	66.54	tr|Q3TS44|Q3TS44_MOUSE	Proteasome subunit alpha type OS=Mus musculus OX=10090 GN=Psma1 PE=1 SV=1	MOUSE	15
478	27.79	27.88	58.76	52.73	sp|Q91Z31|PTBP2_MOUSE	Polypyrimidine tract-binding protein 2 OS=Mus musculus OX=10090 GN=Ptbp2 PE=1 SV=2	MOUSE	18
479	27.76	27.87	38.96	27.43	sp|Q9CZD3|GARS_MOUSE	Glycine--tRNA ligase OS=Mus musculus OX=10090 GN=Gars1 PE=1 SV=1	MOUSE	16
480	27.73	27.78	35.62	25.52	tr|A0JNY3|A0JNY3_MOUSE	Molybdopterin molybdenumtransferase OS=Mus musculus OX=10090 GN=Gphn PE=1 SV=1	MOUSE	17
481	27.71	27.73	53.16	46.52	sp|Q8BYK4|RDH12_MOUSE	Retinol dehydrogenase 12 OS=Mus musculus OX=10090 GN=Rdh12 PE=2 SV=1	MOUSE	15
482	27.66	27.74	26.44	19.92	sp|Q8R1W8|IMPG1_MOUSE	Interphotoreceptor matrix proteoglycan 1 OS=Mus musculus OX=10090 GN=Impg1 PE=1 SV=1	MOUSE	17
483	27.58	27.67	63.24	53.24	sp|Q8BFZ9|ERLN2_MOUSE	Erlin-2 OS=Mus musculus OX=10090 GN=Erlin2 PE=1 SV=1	MOUSE	15
484	27.57	27.59	44.60	39.72	tr|Q8CAN1|Q8CAN1_MOUSE	t-SNARE coiled-coil homology domain-containing protein OS=Mus musculus OX=10090 GN=Stx3 PE=2 SV=1	MOUSE	15
485	27.55	27.59	40.49	38.02	sp|P10852|4F2_MOUSE	4F2 cell-surface antigen heavy chain OS=Mus musculus OX=10090 GN=Slc3a2 PE=1 SV=1	MOUSE	14
486	27.53	29.8	53.41	49.55	tr|B7ZC46|B7ZC46_MOUSE	Septin OS=Mus musculus OX=10090 GN=Septin8 PE=1 SV=1	MOUSE	17
487	27.44	27.46	45.80	43.36	sp|Q9Z2Q6|SEPT5_MOUSE	Septin-5 OS=Mus musculus OX=10090 GN=Septin5 PE=1 SV=2	MOUSE	15
488	27.41	27.44	97.40	97.40	sp|P08228|SODC_MOUSE	Superoxide dismutase [Cu-Zn] OS=Mus musculus OX=10090 GN=Sod1 PE=1 SV=2	MOUSE	15
489	27.39	27.51	32.49	26.26	tr|Q99PC9|Q99PC9_MOUSE	Serine/threonine-protein phosphatase 2A 56 kDa regulatory subunit OS=Mus musculus OX=10090 GN=Ppp2r5d PE=2 SV=1	MOUSE	15
490	27.36	30.52	65.38	58.46	tr|B2RWH3|B2RWH3_MOUSE	Histone H2A OS=Mus musculus OX=10090 GN=Hist2h2aa1 PE=2 SV=1	MOUSE	47

491	27.34	27.49	46.26	44.90	tr|Q3UG68|Q3UG68_MOUSE	Dolichyl-diphosphooligosaccharide--protein glycosyltransferase 48 kDa subunit OS=Mus musculus OX=10090 GN=Ddost PE=2 SV=1	MOUSE	15
492	27.34	27.44	64.38	56.85	tr|Q5SQB7|Q5SQB7_MOUSE	Nucleophosmin OS=Mus musculus OX=10090 GN=Npm1 PE=1 SV=1	MOUSE	16
493	27.34	27.37	52.15	48.10	sp|P62881|GNB5_MOUSE	Guanine nucleotide-binding protein subunit beta-5 OS=Mus musculus OX=10090 GN=Gnb5 PE=1 SV=1	MOUSE	16
494	27.32	27.4	54.37	45.15	tr|Q3U8Y7|Q3U8Y7_MOUSE	AAA domain-containing protein OS=Mus musculus OX=10090 GN=Psmc3 PE=2 SV=1	MOUSE	16
495	27.28	27.47	25.81	17.12	sp|Q8CFI7|RPB2_MOUSE	DNA-directed RNA polymerase II subunit RPB2 OS=Mus musculus OX=10090 GN=Polr2b PE=1 SV=2	MOUSE	13
496	27.22	27.33	26.66	16.03	sp|Q924C1|XPO5_MOUSE	Exportin-5 OS=Mus musculus OX=10090 GN=Xpo5 PE=1 SV=1	MOUSE	13
497	27.17	27.2	45.62	41.12	tr|Q8C243|Q8C243_MOUSE	Peptidase A1 domain-containing protein OS=Mus musculus OX=10090 GN=Ctsd PE=2 SV=1	MOUSE	16
498	27.14	27.29	10.55	5.56	sp|O88737|BSN_MOUSE	Protein bassoon OS=Mus musculus OX=10090 GN=Bsn PE=1 SV=4	MOUSE	15
499	27.01	29.25	77.35	73.82	tr|Q54AE3|Q54AE3_MOUSE	GTP-binding protein beta3 subunit OS=Mus musculus OX=10090 GN=Gnb3 PE=3 SV=1	MOUSE	19
500	27.01	27.14	26.46	18.34	tr|A2AS98|A2AS98_MOUSE	Nck-associated protein 1 OS=Mus musculus OX=10090 GN=Nckap1 PE=1 SV=1	MOUSE	18
501	27	27.03	66.67	58.33	tr|Q642L7|Q642L7_MOUSE	40S ribosomal protein S27a OS=Mus musculus OX=10090 GN=Rps27a PE=2 SV=1	MOUSE	22
502	26.98	27.13	35.96	23.67	tr|Q7TQD5|Q7TQD5_MOUSE	Carnitine O-palmitoyltransferase OS=Mus musculus OX=10090 GN=Cpt1a PE=1 SV=1	MOUSE	16
503	26.98	27.05	73.16	66.54	sp|P67778|PHB_MOUSE	Prohibitin OS=Mus musculus OX=10090 GN=Phb PE=1 SV=1	MOUSE	15
504	26.97	55.24	34.56	29.05	tr|Q3UHJ3|Q3UHJ3_MOUSE	Calcium-transporting ATPase OS=Mus musculus OX=10090 GN=Atp2b2 PE=1 SV=1	MOUSE	27
505	26.95	26.98	48.38	46.93	tr|Q9D066|Q9D066_MOUSE	Inositol-1-monophosphatase OS=Mus musculus OX=10090 GN=Impa1 PE=2 SV=1	MOUSE	13
506	26.9	26.97	51.60	43.91	tr|Q8C1T5|Q8C1T5_MOUSE	Uncharacterized protein OS=Mus musculus OX=10090 GN=Napg PE=2 SV=1	MOUSE	16

507	26.89	26.99	51.48	43.33	tr|Q80UT7|Q80UT7_MOUSE	60S ribosomal protein L7a (Fragment) OS=Mus musculus OX=10090 GN=Rpl7a PE=2 SV=1	MOUSE	18
508	26.88	36.33	69.01	59.72	sp|P08752|GNAI2_MOUSE	Guanine nucleotide-binding protein G(i) subunit alpha-2 OS=Mus musculus OX=10090 GN=Gnai2 PE=1 SV=5	MOUSE	18
509	26.87	26.94	44.17	41.49	tr|B2RS41|B2RS41_MOUSE	Succinate-semialdehyde dehydrogenase OS=Mus musculus OX=10090 GN=Aldh5a1 PE=1 SV=1	MOUSE	14
510	26.77	60.23	66.44	64.43	tr|Q545A2|Q545A2_MOUSE	ADP/ATP translocase OS=Mus musculus OX=10090 GN=Slc25a5 PE=1 SV=1	MOUSE	48
511	26.74	26.79	48.45	48.19	sp|Q8BVQ5|PPME1_MOUSE	Protein phosphatase methylesterase 1 OS=Mus musculus OX=10090 GN=Ppme1 PE=1 SV=5	MOUSE	15
512	26.73	26.83	50.11	38.53	sp|Q99JY0|ECHB_MOUSE	Trifunctional enzyme subunit beta, mitochondrial OS=Mus musculus OX=10090 GN=Hadhb PE=1 SV=1	MOUSE	15
513	26.59	27.73	34.34	24.76	sp|Q810A7|DDX42_MOUSE	ATP-dependent RNA helicase DDX42 OS=Mus musculus OX=10090 GN=Ddx42 PE=1 SV=3	MOUSE	15
514	26.59	26.69	33.66	22.63	tr|G3XA17|G3XA17_MOUSE	Eukaryotic translation initiation factor 4 gamma 2 OS=Mus musculus OX=10090 GN=Eif4g2 PE=1 SV=1	MOUSE	14
515	26.58	26.8	51.52	39.53	sp|Q9CWJ9|PUR9_MOUSE	Bifunctional purine biosynthesis protein ATIC OS=Mus musculus OX=10090 GN=Atic PE=1 SV=2	MOUSE	16
516	26.58	26.66	43.97	39.70	sp|Q9DBF1|AL7A1_MOUSE	Alpha-aminoadipic semialdehyde dehydrogenase OS=Mus musculus OX=10090 GN=Aldh7a1 PE=1 SV=4	MOUSE	15
517	26.55	26.86	18.66	7.00	tr|Z4YL78|Z4YL78_MOUSE	Cytoskeleton-associated protein 5 OS=Mus musculus OX=10090 GN=Ckap5 PE=1 SV=1	MOUSE	12
518	26.53	26.61	58.59	49.86	sp|Q99LC3|NDUAA_MOUSE	NADH dehydrogenase [ubiquinone] 1 alpha subcomplex subunit 10, mitochondrial OS=Mus musculus OX=10090 GN=Ndufa10 PE=1 SV=1	MOUSE	13
519	26.48	26.53	57.46	39.47	tr|Q3UJN2|Q3UJN2_MOUSE	RuvB-like helicase OS=Mus musculus OX=10090 GN=Ruvbl1 PE=2 SV=1	MOUSE	15
520	26.44	28.77	37.24	32.99	sp|O08539|BIN1_MOUSE	Myc box-dependent-interacting protein 1 OS=Mus musculus OX=10090 GN=Bin1 PE=1 SV=1	MOUSE	14
521	26.41	26.46	47.69	47.22	tr|Q3ULW0|Q3ULW0_MOUSE	GTP-binding nuclear protein Ran OS=Mus musculus OX=10090 GN=Ran PE=2 SV=1	MOUSE	16
522	26.39	26.4	42.76	38.28	tr|Q3UR55|Q3UR55_MOUSE	Sodium/potassium-transporting ATPase subunit beta OS=Mus musculus OX=10090 GN=Atp1b2 PE=1 SV=1	MOUSE	18

523	26.37	26.6	25.55	14.26	tr|Q7TSZ3|Q7TSZ3_MOUSE	Leucyl-tRNA synthetase OS=Mus musculus OX=10090 GN=Lars PE=2 SV=1	MOUSE	12
524	26.37	26.38	27.96	23.43	sp|Q8BG39|SV2B_MOUSE	Synaptic vesicle glycoprotein 2B OS=Mus musculus OX=10090 GN=Sv2b PE=1 SV=1	MOUSE	14
525	26.34	26.39	39.74	32.40	tr|Q3UXP2|Q3UXP2_MOUSE	RuvB-like helicase OS=Mus musculus OX=10090 GN=Ruvbl2 PE=2 SV=1	MOUSE	14
526	26.25	26.32	77.74	67.81	sp|P60487|PLPP_MOUSE	Chronophin OS=Mus musculus OX=10090 GN=Pdxp PE=1 SV=1	MOUSE	13
527	26.21	34.2	73.58	64.63	tr|A2A5N2|A2A5N2_MOUSE	Tyrosine 3-monooxygenase/tryptophan 5-monooxygenase activation protein, beta polypeptide OS=Mus musculus OX=10090 GN=Ywhab PE=2 SV=1	MOUSE	18
528	26.04	26.17	34.24	22.62	sp|Q8BG51-2|MIRO1_MOUSE	Isoform 2 of Mitochondrial Rho GTPase 1 OS=Mus musculus OX=10090 GN=Rhot1	MOUSE	12
529	26.03	26.12	19.39	12.45	sp|Q5SQX6|CYFP2_MOUSE	Cytoplasmic FMR1-interacting protein 2 OS=Mus musculus OX=10090 GN=Cyfip2 PE=1 SV=2	MOUSE	14
530	25.96	31.08	65.77	64.15	sp|P57722|PCBP3_MOUSE	Poly(rC)-binding protein 3 OS=Mus musculus OX=10090 GN=Pcbp3 PE=1 SV=3	MOUSE	20
531	25.92	25.94	71.27	67.96	tr|Q3U344|Q3U344_MOUSE	Uncharacterized protein OS=Mus musculus OX=10090 GN=Arf3 PE=2 SV=1	MOUSE	14
532	25.88	26.18	34.84	20.66	tr|Q3UZI3|Q3UZI3_MOUSE	Staphylococcal nuclease domain-containing protein OS=Mus musculus OX=10090 GN=Snd1 PE=2 SV=1	MOUSE	15
533	25.86	26.75	43.76	20.88	sp|O55029|COPB2_MOUSE	Coatomer subunit beta' OS=Mus musculus OX=10090 GN=Copb2 PE=1 SV=2	MOUSE	12
534	25.81	25.91	36.36	26.06	sp|Q9D0I9|SYRC_MOUSE	Arginine--tRNA ligase, cytoplasmic OS=Mus musculus OX=10090 GN=Rars1 PE=1 SV=2	MOUSE	15
535	25.79	30.45	45.51	38.32	sp|P46660|AINX_MOUSE	Alpha-internexin OS=Mus musculus OX=10090 GN=Ina PE=1 SV=3	MOUSE	18
536	25.76	25.8	31.33	30.37	tr|Q8CFQ9|Q8CFQ9_MOUSE	Fusion, derived from t(1216) malignant liposarcoma (Human) OS=Mus musculus OX=10090 GN=Fus PE=1 SV=1	MOUSE	15
537	25.74	25.77	69.70	69.70	sp|Q61171|PRDX2_MOUSE	Peroxiredoxin-2 OS=Mus musculus OX=10090 GN=Prdx2 PE=1 SV=3	MOUSE	15
538	25.56	25.58	64.79	61.50	tr|Q3TF25|Q3TF25_MOUSE	ATP synthase peripheral stalk subunit OSCP OS=Mus musculus OX=10090 GN=Atp5o PE=2 SV=1	MOUSE	15

539	25.5	25.65	65.30	53.31	tr|Q5M8R8|Q5M8R8_MOUSE	60S acidic ribosomal protein P0 OS=Mus musculus OX=10090 GN=Rplp0 PE=2 SV=1	MOUSE	16
540	25.49	25.54	64.50	60.36	tr|A2RSV8|A2RSV8_MOUSE	Cytochrome c oxidase subunit 4 OS=Mus musculus OX=10090 GN=Cox4i1 PE=1 SV=1	MOUSE	15
541	25.46	25.87	29.75	17.05	sp|Q569Z5|DDX46_MOUSE	Probable ATP-dependent RNA helicase DDX46 OS=Mus musculus OX=10090 GN=Ddx46 PE=1 SV=2	MOUSE	16
542	25.38	25.49	40.90	21.69	tr|A0A0R4IZY0|A0A0R4IZY0_MOUSE	Thimet oligopeptidase OS=Mus musculus OX=10090 GN=Thop1 PE=1 SV=1	MOUSE	12
543	25.38	25.42	50.14	50.14	sp|Q9D051|ODPB_MOUSE	Pyruvate dehydrogenase E1 component subunit beta, mitochondrial OS=Mus musculus OX=10090 GN=Pdhb PE=1 SV=1	MOUSE	16
544	25.29	25.36	45.69	42.64	tr|Q5SW83|Q5SW83_MOUSE	Uncharacterized protein OS=Mus musculus OX=10090 GN=Actr2 PE=1 SV=1	MOUSE	15
545	25.26	25.33	29.92	28.03	tr|Q3U9V1|Q3U9V1_MOUSE	TPR_REGION domain-containing protein OS=Mus musculus OX=10090 GN=St13 PE=2 SV=1	MOUSE	13
546	25.24	25.33	54.42	44.73	sp|P51863|VA0D1_MOUSE	V-type proton ATPase subunit d 1 OS=Mus musculus OX=10090 GN=Atp6v0d1 PE=1 SV=2	MOUSE	14
547	25.22	25.29	36.97	34.74	tr|Q3UB90|Q3UB90_MOUSE	Uncharacterized protein OS=Mus musculus OX=10090 GN=Rpl3 PE=2 SV=1	MOUSE	14
548	25.19	25.27	38.96	34.17	tr|Q3UXT6|Q3UXT6_MOUSE	Uncharacterized protein (Fragment) OS=Mus musculus OX=10090 GN=Rbm39 PE=2 SV=1	MOUSE	14
549	25.14	25.32	39.23	17.80	sp|Q80TZ3|AUXI_MOUSE	Putative tyrosine-protein phosphatase auxilin OS=Mus musculus OX=10090 GN=Dnajc6 PE=1 SV=2	MOUSE	14
550	25.09	25.16	45.42	38.60	sp|Q3UKJ7|SMU1_MOUSE	WD40 repeat-containing protein SMU1 OS=Mus musculus OX=10090 GN=Smu1 PE=2 SV=2	MOUSE	14
551	25.05	25.57	35.81	28.88	sp|Q8VEH5|EPMIP_MOUSE	EPM2A-interacting protein 1 OS=Mus musculus OX=10090 GN=Epm2aip1 PE=1 SV=1	MOUSE	13
552	25.03	25.51	48.73	43.04	tr|Q5U415|Q5U415_MOUSE	Aldo-keto reductase family 1, member B3 (Aldose reductase) OS=Mus musculus OX=10090 GN=Akr1b3 PE=2 SV=1	MOUSE	13
553	25	25.02	41.27	33.47	tr|Q3MIA8|Q3MIA8_MOUSE	COP9 signalosome complex subunit 1 OS=Mus musculus OX=10090 GN=Gps1 PE=1 SV=1	MOUSE	15
554	24.92	25.04	48.94	39.62	tr|Q3UIJ2|Q3UIJ2_MOUSE	Protein-synthesizing GTPase OS=Mus musculus OX=10090 GN=Eif2s3x PE=2 SV=1	MOUSE	14

555	24.9	25.11	48.98	37.99	sp|Q99L88|SNTB1_MOUSE	Beta-1-syntrophin OS=Mus musculus OX=10090 GN=Sntb1 PE=1 SV=4	MOUSE	14
556	24.89	25.1	24.43	12.91	tr|F8WHU5|F8WHU5_MOUSE	Sister chromatid cohesion protein PDS5 homolog B OS=Mus musculus OX=10090 GN=Pds5b PE=1 SV=1	MOUSE	14
557	24.87	24.91	74.53	71.91	tr|E9PZF0|E9PZF0_MOUSE	Nucleoside diphosphate kinase OS=Mus musculus OX=10090 GN=Gm20390 PE=3 SV=1	MOUSE	15
558	24.81	24.89	48.47	43.45	tr|Q3UHH5|Q3UHH5_MOUSE	Uncharacterized protein OS=Mus musculus OX=10090 GN=Gnaq PE=1 SV=1	MOUSE	13
559	24.81	24.82	41.03	37.30	sp|Q80VD1|FA98B_MOUSE	Protein FAM98B OS=Mus musculus OX=10090 GN=Fam98b PE=1 SV=1	MOUSE	13
560	24.75	24.88	47.82	31.31	tr|Q3UJ34|Q3UJ34_MOUSE	Argininosuccinate synthase OS=Mus musculus OX=10090 GN=Ass1 PE=1 SV=1	MOUSE	12
561	24.73	24.8	43.91	35.93	tr|Q58E59|Q58E59_MOUSE	Splicing factor 3a, subunit 3 OS=Mus musculus OX=10090 GN=Sf3a3 PE=1 SV=1	MOUSE	15
562	24.67	40.09	72.79	69.11	sp|P62631|EF1A2_MOUSE	Elongation factor 1-alpha 2 OS=Mus musculus OX=10090 GN=Eef1a2 PE=1 SV=1	MOUSE	27
563	24.65	24.67	22.22	18.42	sp|P29974|CNGA1_MOUSE	cGMP-gated cation channel alpha-1 OS=Mus musculus OX=10090 GN=Cnga1 PE=2 SV=2	MOUSE	12
564	24.64	24.66	59.64	48.33	tr|Q14AQ1|Q14AQ1_MOUSE	Proteasome (Prosome, macropain) 26S subunit, ATPase, 6 OS=Mus musculus OX=10090 GN=Psmc6 PE=1 SV=1	MOUSE	13
565	24.61	24.63	26.60	14.85	tr|F8VQC7|F8VQC7_MOUSE	Kinectin OS=Mus musculus OX=10090 GN=Ktn1 PE=1 SV=1	MOUSE	12
566	24.6	26.63	56.34	51.76	tr|Q5EBQ0|Q5EBQ0_MOUSE	Voltage-dependent anion-selective channel protein 3 OS=Mus musculus OX=10090 GN=Vdac3 PE=2 SV=1	MOUSE	13
567	24.58	24.62	49.27	43.80	tr|Q8C2Q8|Q8C2Q8_MOUSE	ATP synthase subunit gamma OS=Mus musculus OX=10090 GN=Atp5c1 PE=1 SV=1	MOUSE	13
568	24.58	24.6	60.13	53.82	tr|Q3UWX7|Q3UWX7_MOUSE	Uncharacterized protein OS=Mus musculus OX=10090 GN=Tcea1 PE=2 SV=1	MOUSE	17
569	24.54	25	15.49	9.81	tr|H3BLH0|H3BLH0_MOUSE	Probable global transcription activator SNF2L2 OS=Mus musculus OX=10090 GN=Smarca2 PE=1 SV=1	MOUSE	15
570	24.47	26.69	55.53	45.67	tr|A0A1S6GWH1|A0A1S6GWH1_MOUSE	AAA domain-containing protein OS=Mus musculus OX=10090 GN=Psmc5 PE=2 SV=1	MOUSE	15

571	24.4	24.47	47.87	36.97	tr|B2RT97|B2RT97_MOUSE	26S proteasome non-ATPase regulatory subunit 13 OS=Mus musculus OX=10090 GN=Psmd13 PE=1 SV=1	MOUSE	13
572	24.39	24.47	34.88	24.55	sp|Q9R190|MTA2_MOUSE	Metastasis-associated protein MTA2 OS=Mus musculus OX=10090 GN=Mta2 PE=1 SV=1	MOUSE	15
573	24.38	24.61	30.24	22.90	tr|S4R1M2|S4R1M2_MOUSE	Scaffold attachment factor B1 OS=Mus musculus OX=10090 GN=Safb PE=1 SV=1	MOUSE	13
574	24.33	24.38	59.50	57.32	sp|Q91V61|SFXN3_MOUSE	Sideroflexin-3 OS=Mus musculus OX=10090 GN=Sfxn3 PE=1 SV=1	MOUSE	13
575	24.3	28.45	81.86	77.21	tr|Q50HX4|Q50HX4_MOUSE	Ras-related protein Rab-14 OS=Mus musculus OX=10090 GN=Rab14 PE=1 SV=1	MOUSE	16
576	24.3	24.33	58.98	48.81	tr|B2CY77|B2CY77_MOUSE	Laminin receptor (Fragment) OS=Mus musculus OX=10090 GN=Rpsa PE=2 SV=1	MOUSE	14
577	24.27	29.54	52.96	51.71	sp|P42669|PURA_MOUSE	Transcriptional activator protein Pur-alpha OS=Mus musculus OX=10090 GN=Pura PE=1 SV=1	MOUSE	20
578	24.25	24.26	65.45	59.47	tr|Q8BUM1|Q8BUM1_MOUSE	TAR DNA-binding protein 43 OS=Mus musculus OX=10090 GN=Tardbp PE=2 SV=1	MOUSE	13
579	24.23	24.26	71.76	59.14	sp|Q9DCN2|NB5R3_MOUSE	NADH-cytochrome b5 reductase 3 OS=Mus musculus OX=10090 GN=Cyb5r3 PE=1 SV=3	MOUSE	13
580	24.22	24.33	27.79	17.99	sp|Q6A0A9|F120A_MOUSE	Constitutive coactivator of PPAR-gamma-like protein 1 OS=Mus musculus OX=10090 GN=FAM120A PE=1 SV=2	MOUSE	13
581	24.19	24.25	61.88	51.76	sp|Q60972|RBBP4_MOUSE	Histone-binding protein RBBP4 OS=Mus musculus OX=10090 GN=Rbbp4 PE=1 SV=5	MOUSE	13
582	24.16	24.2	66.97	60.06	sp|Q99LC5|ETFA_MOUSE	Electron transfer flavoprotein subunit alpha, mitochondrial OS=Mus musculus OX=10090 GN=Etfa PE=1 SV=2	MOUSE	12
583	24.15	24.25	22.69	12.30	sp|Q8CH18|CCAR1_MOUSE	Cell division cycle and apoptosis regulator protein 1 OS=Mus musculus OX=10090 GN=Ccar1 PE=1 SV=1	MOUSE	11
584	24.14	24.73	38.81	35.38	tr|Q3UQ71|Q3UQ71_MOUSE	AB hydrolase-1 domain-containing protein OS=Mus musculus OX=10090 GN=Ephx2 PE=2 SV=1	MOUSE	15
585	24.09	24.26	45.32	45.32	sp|Q8R5M8-4|CADM1_MOUSE	Isoform 4 of Cell adhesion molecule 1 OS=Mus musculus OX=10090 GN=Cadm1	MOUSE	18
586	24.08	24.26	63.55	39.50	sp|Q8CIN4|PAK2_MOUSE	Serine/threonine-protein kinase PAK 2 OS=Mus musculus OX=10090 GN=Pak2 PE=1 SV=1	MOUSE	13

587	24.05	24.1	29.63	21.21	sp|Q9Z0E0-2|NCDN_MOUSE	Isoform 2 of Neurochondrin OS=Mus musculus OX=10090 GN=Ncdn	MOUSE	13
588	24.04	24.1	41.48	32.21	sp|Q3V3N7|BBS1_MOUSE	Bardet-Biedl syndrome 1 protein homolog OS=Mus musculus OX=10090 GN=Bbs1 PE=1 SV=1	MOUSE	13
589	24.04	24.05	47.60	47.16	tr|Q3U125|Q3U125_MOUSE	Peroxiredoxin-like 2 activated in M-CSF stimulated monocytes OS=Mus musculus OX=10090 GN=Prxl2a PE=1 SV=1	MOUSE	13
590	24.03	24.1	37.11	29.47	sp|Q7TNV0|DEK_MOUSE	Protein DEK OS=Mus musculus OX=10090 GN=Dek PE=1 SV=1	MOUSE	13
591	24	24	63.33	63.33	tr|Q3V299|Q3V299_MOUSE	Protein unc-119 homolog A OS=Mus musculus OX=10090 GN=Unc119 PE=1 SV=1	MOUSE	12
592	23.88	23.92	77.52	77.06	tr|Q99KF5|Q99KF5_MOUSE	Hypoxanthine phosphoribosyltransferase OS=Mus musculus OX=10090 GN=Hprt PE=2 SV=1	MOUSE	14
593	23.87	23.91	40.36	35.22	sp|P18572|BASI_MOUSE	Basigin OS=Mus musculus OX=10090 GN=Bsg PE=1 SV=2	MOUSE	14
594	23.86	34.08	59.05	55.71	sp|P30681|HMGB2_MOUSE	High mobility group protein B2 OS=Mus musculus OX=10090 GN=Hmgb2 PE=1 SV=3	MOUSE	26
595	23.82	24.04	63.38	46.71	tr|Q3UM23|Q3UM23_MOUSE	Ribonuclease inhibitor OS=Mus musculus OX=10090 GN=Rnh1 PE=2 SV=1	MOUSE	13
596	23.8	23.94	33.98	32.81	sp|Q3UHL1|CAMKV_MOUSE	CaM kinase-like vesicle-associated protein OS=Mus musculus OX=10090 GN=Camkv PE=1 SV=2	MOUSE	14
597	23.8	23.84	63.91	49.11	sp|Q9DAR7|DCPS_MOUSE	m7GpppX diphosphatase OS=Mus musculus OX=10090 GN=Dcps PE=1 SV=1	MOUSE	12
598	23.78	23.78	64.57	64.57	sp|Q9R0P9|UCHL1_MOUSE	Ubiquitin carboxyl-terminal hydrolase isozyme L1 OS=Mus musculus OX=10090 GN=Uchl1 PE=1 SV=1	MOUSE	14
599	23.72	23.93	23.68	16.63	sp|O88508|DNM3A_MOUSE	DNA (cytosine-5)-methyltransferase 3A OS=Mus musculus OX=10090 GN=Dnmt3a PE=1 SV=2	MOUSE	12
600	23.69	23.98	40.94	29.25	sp|P14685|PSMD3_MOUSE	26S proteasome non-ATPase regulatory subunit 3 OS=Mus musculus OX=10090 GN=Psmd3 PE=1 SV=3	MOUSE	15
601	23.68	23.71	65.41	53.77	tr|A0A498WGD8|A0A498WGD8_MOUSE	Thioredoxin-like protein 1 OS=Mus musculus OX=10090 GN=Txnl1 PE=1 SV=1	MOUSE	13
602	23.68	23.71	42.25	41.83	tr|Q80XR5|Q80XR5_MOUSE	U2 snRNP auxiliary factor large subunit OS=Mus musculus OX=10090 GN=U2af2 PE=1 SV=1	MOUSE	17

603	23.67	23.71	44.16	43.02	sp|P32958|ROM1_MOUSE	Rod outer segment membrane protein 1 OS=Mus musculus OX=10090 GN=Rom1 PE=1 SV=1	MOUSE	18
604	23.58	23.89	55.41	44.32	tr|Q14BL1|Q14BL1_MOUSE	NAD(+) kinase (Fragment) OS=Mus musculus OX=10090 GN=Nadk2 PE=2 SV=2	MOUSE	13
605	23.58	23.69	51.31	37.77	tr|Q91WS8|Q91WS8_MOUSE	Medium-chain specific acyl-CoA dehydrogenase, mitochondrial OS=Mus musculus OX=10090 GN=Acadm PE=2 SV=1	MOUSE	12
606	23.55	23.68	11.73	6.99	sp|Q8BTI8|SRRM2_MOUSE	Serine/arginine repetitive matrix protein 2 OS=Mus musculus OX=10090 GN=Srrm2 PE=1 SV=3	MOUSE	12
607	23.55	23.59	34.14	31.16	sp|Q6DFW4|NOP58_MOUSE	Nucleolar protein 58 OS=Mus musculus OX=10090 GN=Nop58 PE=1 SV=1	MOUSE	12
608	23.52	23.66	60.29	52.21	sp|P84244|H33_MOUSE	Histone H3.3 OS=Mus musculus OX=10090 GN=H3-3a PE=1 SV=2	MOUSE	27
609	23.49	23.57	61.85	53.23	tr|A0A0R4J195|A0A0R4J195_MOUSE	Regulation of nuclear pre-mRNA domain-containing protein 1B OS=Mus musculus OX=10090 GN=Rprd1b PE=1 SV=1	MOUSE	13
610	23.41	23.52	41.62	39.64	sp|Q5SRX1|TM1L2_MOUSE	TOM1-like protein 2 OS=Mus musculus OX=10090 GN=Tom1l2 PE=1 SV=1	MOUSE	13
611	23.4	23.62	50.38	34.81	sp|Q8BK67|RCC2_MOUSE	Protein RCC2 OS=Mus musculus OX=10090 GN=Rcc2 PE=1 SV=1	MOUSE	13
612	23.38	23.46	39.12	38.68	sp|Q8R016|BLMH_MOUSE	Bleomycin hydrolase OS=Mus musculus OX=10090 GN=Blmh PE=1 SV=1	MOUSE	12
613	23.34	23.6	32.77	25.42	sp|Q8BML9|SYQ_MOUSE	Glutamine--tRNA ligase OS=Mus musculus OX=10090 GN=Qars1 PE=1 SV=1	MOUSE	14
614	23.33	23.73	11.70	5.82	tr|E9Q3Y4|E9Q3Y4_MOUSE	Lipopolysaccharide-responsive and beige-like anchor protein OS=Mus musculus OX=10090 GN=Lrba PE=1 SV=1	MOUSE	12
615	23.31	23.47	40.75	27.10	sp|Q9QXY6|EHD3_MOUSE	EH domain-containing protein 3 OS=Mus musculus OX=10090 GN=Ehd3 PE=1 SV=2	MOUSE	15
616	23.29	23.67	53.82	27.83	tr|D3Z0G8|D3Z0G8_MOUSE	MAGUK p55 subfamily member 4 OS=Mus musculus OX=10090 GN=Mpp4 PE=1 SV=2	MOUSE	12
617	23.27	23.31	34.54	30.59	tr|Q545P0|Q545P0_MOUSE	Sodium/potassium-transporting ATPase subunit beta OS=Mus musculus OX=10090 GN=Atp1b1 PE=1 SV=1	MOUSE	13

618	23.26	23.44	55.88	50.74	tr|Q923G3|Q923G3_MOUSE	F-actin-capping protein subunit beta OS=Mus musculus OX=10090 GN=Capzb PE=1 SV=1	MOUSE	15
619	23.24	23.27	43.94	40.28	tr|Q542R8|Q542R8_MOUSE	Uncharacterized protein OS=Mus musculus OX=10090 GN=Gnaz PE=1 SV=1	MOUSE	13
620	23.23	23.31	55.80	54.02	sp|Q9Z1L4|XLRS1_MOUSE	Retinoschisin OS=Mus musculus OX=10090 GN=Rs1 PE=1 SV=1	MOUSE	15
621	23.23	23.29	49.87	47.33	sp|P70404|IDHG1_MOUSE	Isocitrate dehydrogenase [NAD] subunit gamma 1, mitochondrial OS=Mus musculus OX=10090 GN=Idh3g PE=1 SV=1	MOUSE	15
622	23.2	23.28	55.32	50.59	tr|Q3V100|Q3V100_MOUSE	Mannose-6-phosphate isomerase OS=Mus musculus OX=10090 GN=Mpi PE=1 SV=1	MOUSE	12
623	23.07	23.09	62.84	62.84	tr|Q9DCC5|Q9DCC5_MOUSE	Cbx3 protein OS=Mus musculus OX=10090 GN=Cbx3 PE=1 SV=1	MOUSE	12
624	23.04	23.21	38.94	35.48	sp|Q8K1J6|TRNT1_MOUSE	CCA tRNA nucleotidyltransferase 1, mitochondrial OS=Mus musculus OX=10090 GN=Trnt1 PE=1 SV=1	MOUSE	13
625	23.02	23.05	68.30	53.13	sp|P48774|GSTM5_MOUSE	Glutathione S-transferase Mu 5 OS=Mus musculus OX=10090 GN=Gstm5 PE=1 SV=1	MOUSE	12
626	23.01	23.25	67.16	52.77	tr|Q5ND42|Q5ND42_MOUSE	Uncharacterized protein OS=Mus musculus OX=10090 GN=Pitpna PE=1 SV=1	MOUSE	14
627	22.94	23.2	33.52	23.72	tr|A0PJL3|A0PJL3_MOUSE	NAD(+) hydrolase SARM1 (Fragment) OS=Mus musculus OX=10090 GN=Sarm1 PE=2 SV=1	MOUSE	13
628	22.89	22.94	59.17	59.17	tr|Q78ZJ8|Q78ZJ8_MOUSE	RAB11B protein OS=Mus musculus OX=10090 GN=Rab11b PE=1 SV=1	MOUSE	13
629	22.87	23.06	25.74	11.36	tr|Q3UHE7|Q3UHE7_MOUSE	Uncharacterized protein OS=Mus musculus OX=10090 GN=Kif21a PE=2 SV=1	MOUSE	12
630	22.87	23.03	40.48	29.56	sp|P14576|SRP54_MOUSE	Signal recognition particle 54 kDa protein OS=Mus musculus OX=10090 GN=Srp54 PE=1 SV=2	MOUSE	11
631	22.87	22.96	22.39	22.22	tr|A0A1B0GRU0|A0A1B0GRU0_MOUSE	Vesicular glutamate transporter 1 OS=Mus musculus OX=10090 GN=Slc17a7 PE=1 SV=1	MOUSE	12
632	22.83	22.98	19.12	13.87	tr|Q80X48|Q80X48_MOUSE	Nucleoporin 155 OS=Mus musculus OX=10090 GN=Nup155 PE=2 SV=1	MOUSE	12
633	22.81	22.93	50.38	40.15	tr|Q61177|Q61177_MOUSE	Casein kinase II alpha subunit OS=Mus musculus OX=10090 GN=Csnk2a1 PE=1 SV=1	MOUSE	15

634	22.79	22.85	42.37	35.84	tr|D3YTT4|D3YTT4_MOUSE	Isobutyryl-CoA dehydrogenase, mitochondrial OS=Mus musculus OX=10090 GN=Acad8 PE=1 SV=1	MOUSE	12
635	22.79	22.85	52.42	45.70	tr|Q3UVN5|Q3UVN5_MOUSE	NSFL1 cofactor p47 OS=Mus musculus OX=10090 GN=Nsfl1c PE=2 SV=1	MOUSE	15
636	22.72	32.38	29.69	21.28	tr|Q8CHF1|Q8CHF1_MOUSE	Kinesin-like protein (Fragment) OS=Mus musculus OX=10090 GN=mKIAA0531 PE=2 SV=1	MOUSE	16
637	22.71	22.82	21.77	15.67	sp|Q569Z6|TR150_MOUSE	Thyroid hormone receptor-associated protein 3 OS=Mus musculus OX=10090 GN=Thrap3 PE=1 SV=1	MOUSE	12
638	22.69	22.76	65.98	59.75	sp|Q8BVI4|DHPR_MOUSE	Dihydropteridine reductase OS=Mus musculus OX=10090 GN=Qdpr PE=1 SV=2	MOUSE	11
639	22.69	22.74	51.32	46.93	tr|Q545E6|Q545E6_MOUSE	Component 3 of promoter of RISC OS=Mus musculus OX=10090 GN=Tsn PE=1 SV=1	MOUSE	12
640	22.67	22.73	41.55	39.53	tr|Q3UCH0|Q3UCH0_MOUSE	60S ribosomal protein L6 OS=Mus musculus OX=10090 GN=Rpl6 PE=1 SV=1	MOUSE	13
641	22.65	22.86	36.52	32.75	sp|P97300|NPTN_MOUSE	Neuroplastin OS=Mus musculus OX=10090 GN=Nptn PE=1 SV=3	MOUSE	13
642	22.64	38.65	11.63	6.02	tr|W6PPR4|W6PPR4_MOUSE	480-kDa ankyrinG OS=Mus musculus OX=10090 GN=Ank3 PE=4 SV=1	MOUSE	22
643	22.61	22.68	45.45	40.40	tr|Q58EU6|Q58EU6_MOUSE	Ribosomal protein L5 OS=Mus musculus OX=10090 GN=Rpl5 PE=1 SV=1	MOUSE	13
644	22.59	37.37	48.84	43.32	tr|A0A0R4J259|A0A0R4J259_MOUSE	Heterogeneous nuclear ribonucleoprotein Q OS=Mus musculus OX=10090 GN=Syncrip PE=1 SV=1	MOUSE	19
645	22.59	23.38	44.79	37.29	sp|Q8BH44|COR2B_MOUSE	Coronin-2B OS=Mus musculus OX=10090 GN=Coro2b PE=1 SV=2	MOUSE	12
646	22.54	24.69	40.58	38.04	tr|Q9D6G1|Q9D6G1_MOUSE	Uncharacterized protein OS=Mus musculus OX=10090 GN=Hnrnpab PE=2 SV=1	MOUSE	15
647	22.52	22.65	55.58	43.38	sp|Q8QZS1|HIBCH_MOUSE	3-hydroxyisobutyryl-CoA hydrolase, mitochondrial OS=Mus musculus OX=10090 GN=Hibch PE=1 SV=1	MOUSE	14
648	22.49	22.51	98.67	85.84	sp|Q91XV3|BASP1_MOUSE	Brain acid soluble protein 1 OS=Mus musculus OX=10090 GN=Basp1 PE=1 SV=3	MOUSE	12
649	22.48	24.59	62.46	58.25	sp|Q99LD8|DDAH2_MOUSE	N(G),N(G)-dimethylarginine dimethylaminohydrolase 2 OS=Mus musculus OX=10090 GN=Ddah2 PE=1 SV=1	MOUSE	14
650	22.46	29.42	59.35	55.69	sp|P68510|1433F_MOUSE	14-3-3 protein eta OS=Mus musculus OX=10090 GN=Ywhah PE=1 SV=2	MOUSE	16

651	22.46	22.67	41.31	34.58	sp|Q9EQ20|MMSA_MOUSE	Methylmalonate-semialdehyde dehydrogenase [acylating], mitochondrial OS=Mus musculus OX=10090 GN=Aldh6a1 PE=1 SV=1	MOUSE	13
652	22.46	22.63	42.02	28.72	sp|Q9R1C6|DGKE_MOUSE	Diacylglycerol kinase epsilon OS=Mus musculus OX=10090 GN=Dgke PE=1 SV=1	MOUSE	13
653	22.44	22.58	23.25	16.25	tr|Q80U83|Q80U83_MOUSE	MKIAA0079 protein (Fragment) OS=Mus musculus OX=10090 GN=Sec24c PE=2 SV=1	MOUSE	12
654	22.39	22.5	43.78	34.44	tr|Q3V3N6|Q3V3N6_MOUSE	COP9 signalosome complex subunit 2 OS=Mus musculus OX=10090 GN=Cops2 PE=2 SV=1	MOUSE	11
655	22.37	22.43	77.39	68.20	tr|Q99N15|Q99N15_MOUSE	17beta-hydroxysteroid dehydrogenase type 10/short chain L-3-hydroxyacyl-CoA dehydrogenase OS=Mus musculus OX=10090 GN=Hsd17b10 PE=1 SV=1	MOUSE	12
656	22.35	22.61	42.24	25.74	tr|Q3UTI4|Q3UTI4_MOUSE	Guanylate cyclase (Fragment) OS=Mus musculus OX=10090 GN=Gucy1b1 PE=2 SV=1	MOUSE	12
657	22.29	26.62	52.53	49.64	sp|Q9Z2X1|HNRPF_MOUSE	Heterogeneous nuclear ribonucleoprotein F OS=Mus musculus OX=10090 GN=Hnrnpf PE=1 SV=3	MOUSE	15
658	22.27	22.33	53.87	50.83	sp|Q61474|MSI1H_MOUSE	RNA-binding protein Musashi homolog 1 OS=Mus musculus OX=10090 GN=Msi1 PE=1 SV=1	MOUSE	12
659	22.21	22.44	29.12	18.66	sp|P11103|PARP1_MOUSE	Poly [ADP-ribose] polymerase 1 OS=Mus musculus OX=10090 GN=Parp1 PE=1 SV=3	MOUSE	16
660	22.2	22.21	67.88	59.59	tr|Q4VAE6|Q4VAE6_MOUSE	Ras family member A OS=Mus musculus OX=10090 GN=Rhoa PE=1 SV=1	MOUSE	12
661	22.19	26.25	49.22	34.12	tr|Q05DD2|Q05DD2_MOUSE	Neurofilament light polypeptide (Fragment) OS=Mus musculus OX=10090 GN=Nefl PE=2 SV=1	MOUSE	14
662	22.18	22.19	66.83	56.78	sp|Q9R1Q8|TAGL3_MOUSE	Transgelin-3 OS=Mus musculus OX=10090 GN=Tagln3 PE=1 SV=1	MOUSE	14
663	22.17	22.18	43.34	39.93	tr|Q58EU3|Q58EU3_MOUSE	40S ribosomal protein S2 OS=Mus musculus OX=10090 GN=Rps2 PE=1 SV=1	MOUSE	13
664	22.15	24.46	83.64	83.03	tr|Q4FK36|Q4FK36_MOUSE	Actin-depolymerizing factor OS=Mus musculus OX=10090 GN=Dstn PE=1 SV=1	MOUSE	16
665	22.03	22.1	61.29	51.21	tr|Q542H2|Q542H2_MOUSE	Proteasome subunit alpha type OS=Mus musculus OX=10090 GN=Psma7 PE=1 SV=1	MOUSE	12

666	22.03	22.07	51.54	49.23	sp|P97822|AN32E_MOUSE	Acidic leucine-rich nuclear phosphoprotein 32 family member E OS=Mus musculus OX=10090 GN=Anp32e PE=1 SV=2	MOUSE	14
667	22	22	58.25	55.34	sp|P63330|PP2AA_MOUSE	Serine/threonine-protein phosphatase 2A catalytic subunit alpha isoform OS=Mus musculus OX=10090 GN=Ppp2ca PE=1 SV=1	MOUSE	16
668	22	22	44.90	41.40	sp|Q80XN0|BDH_MOUSE	D-beta-hydroxybutyrate dehydrogenase, mitochondrial OS=Mus musculus OX=10090 GN=Bdh1 PE=1 SV=2	MOUSE	12
669	22	22	55.28	52.17	tr|Q810K5|Q810K5_MOUSE	Y box protein 1 OS=Mus musculus OX=10090 GN=Ybx1 PE=2 SV=1	MOUSE	12
670	22	22	76.97	76.97	tr|A2RTT4|A2RTT4_MOUSE	Ubiquitin-conjugating enzyme E2N OS=Mus musculus OX=10090 GN=Ube2n PE=1 SV=1	MOUSE	12
671	21.98	22.05	18.82	9.90	tr|Q3U2W2|Q3U2W2_MOUSE	Uncharacterized protein OS=Mus musculus OX=10090 GN=Mybbp1a PE=1 SV=1	MOUSE	11
672	21.97	22.12	31.16	24.46	tr|D3YYT1|D3YYT1_MOUSE	Glyoxylate reductase 1 homolog OS=Mus musculus OX=10090 GN=Glyr1 PE=1 SV=1	MOUSE	11
673	21.97	22.09	59.85	52.27	sp|O55234|PSB5_MOUSE	Proteasome subunit beta type-5 OS=Mus musculus OX=10090 GN=Psmb5 PE=1 SV=3	MOUSE	13
674	21.97	22.05	56.70	48.33	tr|Q8BKU2|Q8BKU2_MOUSE	26S proteasome AAA-ATPase subunit RPT3 OS=Mus musculus OX=10090 GN=Psmc4 PE=2 SV=1	MOUSE	12
675	21.92	22.1	24.77	18.48	tr|Q542D9|Q542D9_MOUSE	Transferrin receptor protein 1 OS=Mus musculus OX=10090 GN=Tfrc PE=1 SV=1	MOUSE	12
676	21.89	22.07	40.88	27.45	sp|Q60676|PPP5_MOUSE	Serine/threonine-protein phosphatase 5 OS=Mus musculus OX=10090 GN=Ppp5c PE=1 SV=3	MOUSE	13
677	21.85	21.91	56.61	50.51	tr|H3BKH6|H3BKH6_MOUSE	S-formylglutathione hydrolase OS=Mus musculus OX=10090 GN=Esd PE=1 SV=1	MOUSE	12
678	21.83	21.88	39.74	38.78	sp|Q8K183|PDXK_MOUSE	Pyridoxal kinase OS=Mus musculus OX=10090 GN=Pdxk PE=1 SV=1	MOUSE	11
679	21.8	21.9	40.57	29.14	sp|Q99J36|THUM1_MOUSE	THUMP domain-containing protein 1 OS=Mus musculus OX=10090 GN=Thumpd1 PE=1 SV=1	MOUSE	10
680	21.75	22.11	11.80	9.01	tr|B9EJ54|B9EJ54_MOUSE	Nucleoporin 205 OS=Mus musculus OX=10090 GN=Nup205 PE=1 SV=1	MOUSE	14
681	21.73	21.85	18.27	13.78	sp|Q9CSH3|RRP44_MOUSE	Exosome complex exonuclease RRP44 OS=Mus musculus OX=10090 GN=Dis3 PE=1 SV=4	MOUSE	11

682	21.72	21.93	47.73	40.00	sp|Q8BGT8|PHIPL_MOUSE	Phytanoyl-CoA hydroxylase-interacting protein-like OS=Mus musculus OX=10090 GN=Phyhipl PE=1 SV=1	MOUSE	14
683	21.71	21.78	36.29	34.22	tr|Q52L97|Q52L97_MOUSE	Importin subunit alpha OS=Mus musculus OX=10090 GN=Kpna2 PE=1 SV=1	MOUSE	11
684	21.68	21.75	62.50	50.46	tr|Q9DCY1|Q9DCY1_MOUSE	Peptidyl-prolyl cis-trans isomerase OS=Mus musculus OX=10090 GN=Ppib PE=1 SV=1	MOUSE	14
685	21.63	21.71	58.20	47.54	tr|Q3TIJ4|Q3TIJ4_MOUSE	RNA 3'-terminal phosphate cyclase OS=Mus musculus OX=10090 GN=Rtca PE=2 SV=1	MOUSE	11
686	21.54	21.59	44.19	38.64	sp|P49442|INPP_MOUSE	Inositol polyphosphate 1-phosphatase OS=Mus musculus OX=10090 GN=Inpp1 PE=1 SV=2	MOUSE	11
687	21.52	25.46	83.66	68.32	tr|Q5SW88|Q5SW88_MOUSE	RAB1A, member RAS oncogene family OS=Mus musculus OX=10090 GN=Rab1a PE=1 SV=1	MOUSE	13
688	21.47	21.6	37.82	28.08	tr|Q4FJL2|Q4FJL2_MOUSE	Reticulon OS=Mus musculus OX=10090 GN=Rtn1 PE=1 SV=1	MOUSE	12
689	21.45	21.66	21.82	11.91	tr|Q3TH57|Q3TH57_MOUSE	Uncharacterized protein OS=Mus musculus OX=10090 GN=Tcerg1 PE=2 SV=1	MOUSE	12
690	21.45	21.49	69.05	57.14	sp|P19157|GSTP1_MOUSE	Glutathione S-transferase P 1 OS=Mus musculus OX=10090 GN=Gstp1 PE=1 SV=2	MOUSE	20
691	21.44	21.49	40.67	26.52	tr|Q3UIG0|Q3UIG0_MOUSE	Eukaryotic translation initiation factor 3 subunit E OS=Mus musculus OX=10090 GN=Eif3e PE=1 SV=1	MOUSE	11
692	21.41	21.43	64.80	62.76	sp|Q9DBP5|KCY_MOUSE	UMP-CMP kinase OS=Mus musculus OX=10090 GN=Cmpk1 PE=1 SV=1	MOUSE	11
693	21.37	21.44	48.44	48.12	sp|Q9DCJ9|NPL_MOUSE	N-acetylneuraminate lyase OS=Mus musculus OX=10090 GN=Npl PE=1 SV=1	MOUSE	12
694	21.35	21.53	19.19	17.28	sp|Q810U4|NRCAM_MOUSE	Neuronal cell adhesion molecule OS=Mus musculus OX=10090 GN=Nrcam PE=1 SV=2	MOUSE	14
695	21.34	21.41	43.75	38.28	tr|E9PYH2|E9PYH2_MOUSE	Cytosolic acyl coenzyme A thioester hydrolase OS=Mus musculus OX=10090 GN=Acot7 PE=1 SV=1	MOUSE	12
696	21.32	21.43	56.83	53.14	sp|Q7TQI3|OTUB1_MOUSE	Ubiquitin thioesterase OTUB1 OS=Mus musculus OX=10090 GN=Otub1 PE=1 SV=2	MOUSE	15
697	21.32	21.35	40.71	36.71	sp|Q9DCL9|PUR6_MOUSE	Multifunctional protein ADE2 OS=Mus musculus OX=10090 GN=Paics PE=1 SV=4	MOUSE	15
698	21.3	21.75	55.56	45.93	tr|Q5M9N8|Q5M9N8_MOUSE	Ribosomal protein L7 OS=Mus musculus OX=10090 GN=Rpl7 PE=1 SV=1	MOUSE	14

699	21.28	21.68	27.58	12.37	tr|Q8CGB9|Q8CGB9_MOUSE	Insulin-degrading enzyme OS=Mus musculus OX=10090 GN=Ide PE=1 SV=1	MOUSE	11
700	21.28	21.48	42.55	25.13	sp|P35235-1|PTN11_MOUSE	Isoform 2 of Tyrosine-protein phosphatase non-receptor type 11 OS=Mus musculus OX=10090 GN=Ptpn11	MOUSE	11
701	21.22	21.35	29.40	20.28	sp|Q8R050|ERF3A_MOUSE	Eukaryotic peptide chain release factor GTP-binding subunit ERF3A OS=Mus musculus OX=10090 GN=Gspt1 PE=1 SV=2	MOUSE	12
702	21.21	21.32	31.42	22.91	tr|A0A0R4J107|A0A0R4J107_MOUSE	Acyl-peptide hydrolase (Fragment) OS=Mus musculus OX=10090 GN=Apeh PE=1 SV=1	MOUSE	11
703	21.18	23.46	37.04	30.71	tr|B2RRX2|B2RRX2_MOUSE	Serine/threonine-protein phosphatase OS=Mus musculus OX=10090 GN=Ppp3ca PE=2 SV=1	MOUSE	14
704	21.17	21.24	68.39	56.99	tr|Q3TTY6|Q3TTY6_MOUSE	Uncharacterized protein (Fragment) OS=Mus musculus OX=10090 GN=Lin7c PE=2 SV=1	MOUSE	12
705	21.15	21.53	67.45	66.98	tr|Q0PD65|Q0PD65_MOUSE	Rab2A OS=Mus musculus OX=10090 GN=Rab2a PE=1 SV=1	MOUSE	15
706	21.12	21.42	31.74	22.87	sp|Q8QZY1|EIF3L_MOUSE	Eukaryotic translation initiation factor 3 subunit L OS=Mus musculus OX=10090 GN=Eif3l PE=1 SV=1	MOUSE	11
707	21.12	21.15	37.45	30.04	tr|Q3TDA7|Q3TDA7_MOUSE	Uncharacterized protein OS=Mus musculus OX=10090 GN=Pacsin2 PE=1 SV=1	MOUSE	12
708	21.08	21.15	23.52	20.55	tr|Q8R056|Q8R056_MOUSE	Prom1 protein OS=Mus musculus OX=10090 GN=Prom1 PE=1 SV=1	MOUSE	15
709	21.07	24.71	78.90	58.23	sp|P51859|HDGF_MOUSE	Hepatoma-derived growth factor OS=Mus musculus OX=10090 GN=Hdgf PE=1 SV=2	MOUSE	15
710	21.05	21.86	30.03	20.28	tr|Q3U6A2|Q3U6A2_MOUSE	Uncharacterized protein OS=Mus musculus OX=10090 GN=Rbm14 PE=2 SV=1	MOUSE	12
711	21.04	21.3	25.07	12.94	tr|B7ZNJ3|B7ZNJ3_MOUSE	Caskin1 protein OS=Mus musculus OX=10090 GN=Caskin1 PE=2 SV=1	MOUSE	11
712	21.04	21.25	29.07	21.17	tr|Q05DR5|Q05DR5_MOUSE	FACT complex subunit SSRP1 (Fragment) OS=Mus musculus OX=10090 GN=Ssrp1 PE=2 SV=1	MOUSE	12
713	21	21.13	63.16	48.68	tr|Q5ND51|Q5ND51_MOUSE	V-crk sarcoma virus CT10 oncogene-like protein OS=Mus musculus OX=10090 GN=Crk PE=2 SV=1	MOUSE	11
714	20.98	21.32	43.40	35.64	tr|Q99NF7|Q99NF7_MOUSE	Protein-serine/threonine phosphatase OS=Mus musculus OX=10090 GN=Ppm1b PE=1 SV=1	MOUSE	12

715	20.97	21.03	57.66	54.05	tr|Q4FJX9|Q4FJX9_MOUSE	Superoxide dismutase OS=Mus musculus OX=10090 GN=Sod2 PE=1 SV=1	MOUSE	16
716	20.96	21.01	45.49	39.75	tr|A2AE89|A2AE89_MOUSE	Glutathione transferase OS=Mus musculus OX=10090 GN=Gstm1 PE=1 SV=2	MOUSE	11
717	20.95	21.05	42.92	25.19	tr|F8VQC1|F8VQC1_MOUSE	Signal recognition particle subunit SRP72 OS=Mus musculus OX=10090 GN=Srp72 PE=1 SV=1	MOUSE	11
718	20.95	21.02	38.21	38.21	sp|Q921H8|THIKA_MOUSE	3-ketoacyl-CoA thiolase A, peroxisomal OS=Mus musculus OX=10090 GN=Acaa1a PE=1 SV=1	MOUSE	12
719	20.93	21.01	55.06	45.34	tr|Q3UJH5|Q3UJH5_MOUSE	V-type proton ATPase subunit D OS=Mus musculus OX=10090 GN=Atp6v1d PE=2 SV=1	MOUSE	10
720	20.91	21.07	54.26	40.78	tr|Q3TE45|Q3TE45_MOUSE	Succinate dehydrogenase [ubiquinone] iron-sulfur subunit, mitochondrial OS=Mus musculus OX=10090 GN=Sdhb PE=2 SV=1	MOUSE	12
721	20.86	21.07	34.52	24.01	sp|Q9CZN7|GLYM_MOUSE	Serine hydroxymethyltransferase, mitochondrial OS=Mus musculus OX=10090 GN=Shmt2 PE=1 SV=1	MOUSE	11
722	20.86	20.93	33.63	32.30	tr|Q3UR33|Q3UR33_MOUSE	Uncharacterized protein OS=Mus musculus OX=10090 GN=Tmpo PE=2 SV=1	MOUSE	13
723	20.82	20.93	42.55	31.75	tr|Q3U4K3|Q3U4K3_MOUSE	Complex I-9kD (Fragment) OS=Mus musculus OX=10090 GN=Ndufv3 PE=2 SV=1	MOUSE	11
724	20.81	20.96	29.95	25.20	tr|Q6NXZ0|Q6NXZ0_MOUSE	Dipeptidyl peptidase 3 OS=Mus musculus OX=10090 GN=Dpp3 PE=2 SV=1	MOUSE	13
725	20.79	20.95	79.31	64.37	tr|Q5XJZ8|Q5XJZ8_MOUSE	Glutathione peroxidase (Fragment) OS=Mus musculus OX=10090 GN=Gpx4 PE=2 SV=3	MOUSE	14
726	20.74	20.88	25.72	13.07	sp|Q91YR7|PRP6_MOUSE	Pre-mRNA-processing factor 6 OS=Mus musculus OX=10090 GN=Prpf6 PE=1 SV=1	MOUSE	11
727	20.69	20.79	30.49	20.88	sp|P50544|ACADV_MOUSE	Very long-chain specific acyl-CoA dehydrogenase, mitochondrial OS=Mus musculus OX=10090 GN=Acadvl PE=1 SV=3	MOUSE	10
728	20.65	20.81	32.53	20.51	tr|Q8R2P8|Q8R2P8_MOUSE	Lysine--tRNA ligase OS=Mus musculus OX=10090 GN=Kars PE=1 SV=1	MOUSE	11
729	20.63	20.76	65.65	62.60	tr|Q91XH5|Q91XH5_MOUSE	Sepiapterin reductase OS=Mus musculus OX=10090 GN=Spr PE=1 SV=1	MOUSE	12
730	20.51	20.59	48.39	41.01	tr|Q5XJF6|Q5XJF6_MOUSE	Ribosomal protein OS=Mus musculus OX=10090 GN=Rpl10a PE=1 SV=1	MOUSE	11

731	20.48	20.5	61.72	52.07	sp|Q9QZE7|TSNAX_MOUSE	Translin-associated protein X OS=Mus musculus OX=10090 GN=Tsnax PE=1 SV=1	MOUSE	12
732	20.46	20.94	29.94	15.99	sp|O55201|SPT5H_MOUSE	Transcription elongation factor SPT5 OS=Mus musculus OX=10090 GN=Supt5h PE=1 SV=1	MOUSE	11
733	20.45	20.51	83.80	83.80	tr|Q9CY10|Q9CY10_MOUSE	GLOBIN domain-containing protein OS=Mus musculus OX=10090 PE=2 SV=1	MOUSE	16
734	20.43	20.55	67.95	41.03	tr|Q0VGQ1|Q0VGQ1_MOUSE	Hydroxysteroid (17-beta) dehydrogenase 12 OS=Mus musculus OX=10090 GN=Hsd17b12 PE=2 SV=1	MOUSE	10
735	20.4	20.77	26.23	21.61	tr|F8VQ42|F8VQ42_MOUSE	Kinesin-like protein (Fragment) OS=Mus musculus OX=10090 GN=Kif2a PE=1 SV=1	MOUSE	12
736	20.4	20.46	34.63	31.76	tr|A0A0R4J008|A0A0R4J008_MOUSE	Histone deacetylase 2 OS=Mus musculus OX=10090 GN=Hdac2 PE=1 SV=1	MOUSE	11
737	20.36	20.77	48.62	45.57	tr|Q3UJS2|Q3UJS2_MOUSE	Uncharacterized protein OS=Mus musculus OX=10090 GN=Fbl PE=2 SV=1	MOUSE	11
738	20.33	94.31	86.74	86.74	tr|B2RSN3|B2RSN3_MOUSE	Tubulin beta chain OS=Mus musculus OX=10090 GN=Tubb2b PE=1 SV=1	MOUSE	129
739	20.33	20.4	30.30	26.58	tr|Q80W12|Q80W12_MOUSE	TROVE domain family, member 2 OS=Mus musculus OX=10090 GN=Ro60 PE=2 SV=1	MOUSE	11
740	20.32	20.51	24.90	20.78	tr|Q3TYK4|Q3TYK4_MOUSE	Uncharacterized protein OS=Mus musculus OX=10090 GN=Prkar1a PE=2 SV=1	MOUSE	11
741	20.3	20.64	29.67	15.75	sp|Q8BJ71|NUP93_MOUSE	Nuclear pore complex protein Nup93 OS=Mus musculus OX=10090 GN=Nup93 PE=1 SV=1	MOUSE	10
742	20.3	20.31	53.41	45.65	sp|Q8VCA8|SCRN2_MOUSE	Secernin-2 OS=Mus musculus OX=10090 GN=Scrn2 PE=1 SV=1	MOUSE	10
743	20.25	20.44	40.25	37.97	tr|Q543C7|Q543C7_MOUSE	Uncharacterized protein OS=Mus musculus OX=10090 GN=Nr2e3 PE=2 SV=1	MOUSE	12
744	20.2	20.42	15.55	9.97	tr|B9EHJ3|B9EHJ3_MOUSE	Tight junction protein ZO-1 OS=Mus musculus OX=10090 GN=Tjp1 PE=1 SV=1	MOUSE	13
745	20.2	20.32	55.69	43.41	sp|O35864|CSN5_MOUSE	COP9 signalosome complex subunit 5 OS=Mus musculus OX=10090 GN=Cops5 PE=1 SV=3	MOUSE	11
746	20.17	20.18	63.81	60.00	tr|Q56A15|Q56A15_MOUSE	Cytochrome c OS=Mus musculus OX=10090 GN=Cycs PE=1 SV=1	MOUSE	14
747	20.15	20.32	55.51	50.61	tr|G5E8R0|G5E8R0_MOUSE	Tropomyosin alpha-1 chain OS=Mus musculus OX=10090 GN=Tpm1 PE=1 SV=1	MOUSE	11

748	20.13	23.79	60.89	54.46	sp|Q9JMG7|HDGR3_MOUSE	Hepatoma-derived growth factor-related protein 3 OS=Mus musculus OX=10090 GN=Hdgfl3 PE=1 SV=2	MOUSE	23
749	20.12	20.16	53.02	52.56	sp|O08547|SC22B_MOUSE	Vesicle-trafficking protein SEC22b OS=Mus musculus OX=10090 GN=Sec22b PE=1 SV=3	MOUSE	11
750	20.08	20.08	52.28	48.73	tr|Q545R3|Q545R3_MOUSE	N-myc downstream-regulated gene 1 protein OS=Mus musculus OX=10090 GN=Ndrg1 PE=1 SV=1	MOUSE	14
751	20.06	20.7	23.38	16.12	sp|Q8BGC0|HTSF1_MOUSE	HIV Tat-specific factor 1 homolog OS=Mus musculus OX=10090 GN=Htatsf1 PE=1 SV=1	MOUSE	12
752	20.06	20.06	24.85	22.10	tr|Q6NVA3|Q6NVA3_MOUSE	Ewing sarcoma breakpoint region 1 OS=Mus musculus OX=10090 GN=Ewsr1 PE=2 SV=1	MOUSE	13
753	20.04	20.07	43.13	43.13	sp|P47963|RL13_MOUSE	60S ribosomal protein L13 OS=Mus musculus OX=10090 GN=Rpl13 PE=1 SV=3	MOUSE	11
754	20.02	20.21	21.78	10.46	tr|E9Q683|E9Q683_MOUSE	Voltage-dependent calcium channel subunit alpha-2/delta-2 OS=Mus musculus OX=10090 GN=Cacna2d2 PE=1 SV=2	MOUSE	10
755	20.01	20.27	40.09	27.37	tr|Q3UUH0|Q3UUH0_MOUSE	Uncharacterized protein OS=Mus musculus OX=10090 GN=Asl PE=2 SV=1	MOUSE	10
756	20.01	20.1	44.73	32.90	sp|Q99JI4|PSMD6_MOUSE	26S proteasome non-ATPase regulatory subunit 6 OS=Mus musculus OX=10090 GN=Psmd6 PE=1 SV=1	MOUSE	12
757	20.01	20.01	63.40	59.28	sp|Q9R0Y5|KAD1_MOUSE	Adenylate kinase isoenzyme 1 OS=Mus musculus OX=10090 GN=Ak1 PE=1 SV=1	MOUSE	11
758	20	20	60.08	55.65	sp|Q9D6J6|NDUV2_MOUSE	NADH dehydrogenase [ubiquinone] flavoprotein 2, mitochondrial OS=Mus musculus OX=10090 GN=Ndufv2 PE=1 SV=2	MOUSE	11
759	19.98	20.03	48.24	39.22	sp|O08583|THOC4_MOUSE	THO complex subunit 4 OS=Mus musculus OX=10090 GN=Alyref PE=1 SV=3	MOUSE	14
760	19.94	20.09	66.27	54.82	tr|Q9DB79|Q9DB79_MOUSE	40S ribosomal protein S11 OS=Mus musculus OX=10090 GN=Rps11 PE=2 SV=1	MOUSE	11
761	19.93	19.97	66.96	63.00	tr|Q545L9|Q545L9_MOUSE	Protein-L-isoaspartate O-methyltransferase OS=Mus musculus OX=10090 GN=Pcmt1 PE=2 SV=1	MOUSE	11
762	19.86	19.9	46.44	40.25	tr|Q8C0E1|Q8C0E1_MOUSE	Serine/threonine-protein phosphatase 2A activator OS=Mus musculus OX=10090 GN=Ptpa PE=2 SV=1	MOUSE	10
763	19.85	19.97	53.31	51.36	tr|Q3UJS0|Q3UJS0_MOUSE	60S ribosomal protein L8 OS=Mus musculus OX=10090 GN=Rpl8 PE=2 SV=1	MOUSE	12

764	19.83	20.42	41.08	28.39	sp|Q9D0L8|MCES_MOUSE	mRNA cap guanine-N7 methyltransferase OS=Mus musculus OX=10090 GN=Rnmt PE=1 SV=1	MOUSE	10
765	19.83	19.96	41.56	27.22	tr|Q5PPQ7|Q5PPQ7_MOUSE	Coronin OS=Mus musculus OX=10090 GN=Coro1c PE=2 SV=1	MOUSE	10
766	19.83	19.89	75.65	60.62	sp|O88848-2|ARL6_MOUSE	Isoform 2 of ADP-ribosylation factor-like protein 6 OS=Mus musculus OX=10090 GN=Arl6	MOUSE	10
767	19.82	19.88	57.81	57.81	tr|Q8BPG5|Q8BPG5_MOUSE	Uncharacterized protein OS=Mus musculus OX=10090 GN=Rac1 PE=2 SV=1	MOUSE	12
768	19.8	48	52.09	37.20	tr|A1BN54|A1BN54_MOUSE	Alpha actinin 1a OS=Mus musculus OX=10090 GN=Actn1 PE=1 SV=1	MOUSE	28
769	19.79	19.89	47.17	37.74	tr|Q3TI27|Q3TI27_MOUSE	Ribose-phosphate diphosphokinase OS=Mus musculus OX=10090 GN=Prps1 PE=2 SV=1	MOUSE	10
770	19.75	20.09	47.95	33.98	sp|Q9DBE8|ALG2_MOUSE	Alpha-1,3/1,6-mannosyltransferase ALG2 OS=Mus musculus OX=10090 GN=Alg2 PE=1 SV=2	MOUSE	10
771	19.75	19.82	50.20	44.71	sp|Q9DCW4|ETFB_MOUSE	Electron transfer flavoprotein subunit beta OS=Mus musculus OX=10090 GN=Etfb PE=1 SV=3	MOUSE	11
772	19.69	19.79	55.67	55.67	sp|Q9CVB6|ARPC2_MOUSE	Actin-related protein 2/3 complex subunit 2 OS=Mus musculus OX=10090 GN=Arpc2 PE=1 SV=3	MOUSE	12
773	19.69	19.74	66.08	60.79	sp|Q9CQF3|CPSF5_MOUSE	Cleavage and polyadenylation specificity factor subunit 5 OS=Mus musculus OX=10090 GN=Nudt21 PE=1 SV=1	MOUSE	11
774	19.64	19.69	42.24	36.02	tr|Q3TXK9|Q3TXK9_MOUSE	Glutathione reductase (Fragment) OS=Mus musculus OX=10090 GN=Gsr PE=2 SV=1	MOUSE	11
775	19.6	19.72	45.53	43.63	tr|Q8BK37|Q8BK37_MOUSE	Pribosyltran_N domain-containing protein OS=Mus musculus OX=10090 GN=Prpsap2 PE=2 SV=1	MOUSE	11
776	19.58	19.63	34.35	31.58	sp|P55264|ADK_MOUSE	Adenosine kinase OS=Mus musculus OX=10090 GN=Adk PE=1 SV=2	MOUSE	10
777	19.57	19.74	48.48	21.36	tr|B2RRH9|B2RRH9_MOUSE	Glutamine amidotransferase OS=Mus musculus OX=10090 GN=Gmps PE=1 SV=1	MOUSE	9
778	19.55	19.75	38.50	34.75	sp|Q9JMH6|TRXR1_MOUSE	Thioredoxin reductase 1, cytoplasmic OS=Mus musculus OX=10090 GN=Txnrd1 PE=1 SV=3	MOUSE	13
779	19.43	78.77	89.31	80.08	tr|A0A0A0MQA5|A0A0A0MQA5_MOUSE	Tubulin alpha chain (Fragment) OS=Mus musculus OX=10090 GN=Tuba4a PE=1 SV=1	MOUSE	90

780	19.42	19.61	41.88	28.18	sp|Q5XJY5|COPD_MOUSE	Coatomer subunit delta OS=Mus musculus OX=10090 GN=Arcn1 PE=1 SV=2	MOUSE	11
781	19.33	21.57	43.23	22.08	tr|B9EHZ5|B9EHZ5_MOUSE	Membrane protein, palmitoylated 6 (MAGUK p55 subfamily member 6) OS=Mus musculus OX=10090 GN=Mpp6 PE=2 SV=1	MOUSE	10
782	19.3	19.36	69.57	57.61	tr|Q5XK38|Q5XK38_MOUSE	Hmgn2 protein OS=Mus musculus OX=10090 GN=Hmgn2 PE=1 SV=1	MOUSE	57
783	19.26	19.71	45.15	38.83	tr|Q3TFF0|Q3TFF0_MOUSE	Uncharacterized protein OS=Mus musculus OX=10090 GN=Dnaja2 PE=2 SV=1	MOUSE	11
784	19.23	19.37	80.39	78.43	tr|Q4KL76|Q4KL76_MOUSE	10 kDa heat shock protein, mitochondrial OS=Mus musculus OX=10090 GN=Hspe1 PE=1 SV=1	MOUSE	11
785	19.23	19.32	32.13	22.41	sp|Q9QZM0|UBQL2_MOUSE	Ubiquilin-2 OS=Mus musculus OX=10090 GN=Ubqln2 PE=1 SV=2	MOUSE	10
786	19.21	21.57	20.55	13.99	tr|A0A1D5RM83|A0A1D5RM83_MOUSE	IQ motif and SEC7 domain-containing protein 1 OS=Mus musculus OX=10090 GN=Iqsec1 PE=1 SV=1	MOUSE	12
787	19.14	19.36	17.63	12.27	sp|Q6GQT9|NOMO1_MOUSE	Nodal modulator 1 OS=Mus musculus OX=10090 GN=Nomo1 PE=1 SV=1	MOUSE	11
788	19.14	19.21	37.86	32.82	tr|Q3UBP0|Q3UBP0_MOUSE	Adenylosuccinate synthetase isozyme 1 OS=Mus musculus OX=10090 GN=Adssl1 PE=2 SV=1	MOUSE	10
789	19.13	19.2	59.39	54.24	sp|Q9DAW9|CNN3_MOUSE	Calponin-3 OS=Mus musculus OX=10090 GN=Cnn3 PE=1 SV=1	MOUSE	11
790	19.12	19.19	28.92	28.92	tr|Q8BT09|Q8BT09_MOUSE	40S ribosomal protein S6 OS=Mus musculus OX=10090 PE=2 SV=1	MOUSE	12
791	19.12	19.19	33.71	33.43	tr|Q4FJN9|Q4FJN9_MOUSE	Fdps protein OS=Mus musculus OX=10090 GN=Fdps PE=1 SV=1	MOUSE	10
792	19.1	35.48	51.11	50.37	tr|Q52KC1|Q52KC1_MOUSE	RNA helicase OS=Mus musculus OX=10090 GN=Eif4a2 PE=2 SV=1	MOUSE	19
793	19.08	19.48	18.86	7.59	sp|E9Q7G0|NUMA1_MOUSE	Nuclear mitotic apparatus protein 1 OS=Mus musculus OX=10090 GN=Numa1 PE=1 SV=1	MOUSE	10
794	19.08	19.17	72.93	59.83	sp|Q9CXW3|CYBP_MOUSE	Calcyclin-binding protein OS=Mus musculus OX=10090 GN=Cacybp PE=1 SV=1	MOUSE	10
795	19.05	19.15	56.74	40.66	tr|Q3UQL2|Q3UQL2_MOUSE	COP9 signalosome complex subunit 3 OS=Mus musculus OX=10090 GN=Cops3 PE=1 SV=1	MOUSE	10

796	18.99	19.1	49.07	47.22	tr|A0A140LHA2|A0A140LHA2_MOUSE	Mitotic checkpoint protein BUB3 OS=Mus musculus OX=10090 GN=Bub3 PE=1 SV=1	MOUSE	12
797	18.98	19.09	29.06	23.33	sp|Q8R326|PSPC1_MOUSE	Paraspeckle component 1 OS=Mus musculus OX=10090 GN=Pspc1 PE=1 SV=1	MOUSE	10
798	18.98	19.01	35.81	29.77	tr|A0A0R4J083|A0A0R4J083_MOUSE	Long-chain specific acyl-CoA dehydrogenase, mitochondrial OS=Mus musculus OX=10090 GN=Acadl PE=1 SV=1	MOUSE	11
799	18.97	19.1	26.20	13.28	tr|Q6PCP0|Q6PCP0_MOUSE	RNA helicase OS=Mus musculus OX=10090 GN=Ddx21 PE=2 SV=1	MOUSE	9
800	18.93	19.06	57.57	43.24	sp|Q9R0Q6|ARC1A_MOUSE	Actin-related protein 2/3 complex subunit 1A OS=Mus musculus OX=10090 GN=Arpc1a PE=1 SV=1	MOUSE	11
801	18.92	18.94	27.99	26.64	tr|V9GX43|V9GX43_MOUSE	CUGBP Elav-like family member 2 (Fragment) OS=Mus musculus OX=10090 GN=Celf2 PE=1 SV=1	MOUSE	13
802	18.9	18.99	71.33	56.99	tr|Q5DQJ3|Q5DQJ3_MOUSE	F-actin-capping protein subunit alpha OS=Mus musculus OX=10090 GN=Capza2 PE=1 SV=1	MOUSE	11
803	18.89	18.97	71.28	56.38	tr|Q5J7N1|Q5J7N1_MOUSE	Kras protein OS=Mus musculus OX=10090 GN=Kras PE=2 SV=1	MOUSE	11
804	18.85	18.99	17.39	13.52	tr|B9EJ23|B9EJ23_MOUSE	Cask protein OS=Mus musculus OX=10090 GN=Cask PE=2 SV=1	MOUSE	10
805	18.85	18.97	15.85	9.29	sp|Q6P9K9|NRX3A_MOUSE	Neurexin-3 OS=Mus musculus OX=10090 GN=Nrxn3 PE=1 SV=2	MOUSE	11
806	18.79	18.92	40.97	29.17	tr|Q0VEE0|Q0VEE0_MOUSE	Ecto-5'-nucleotidase OS=Mus musculus OX=10090 GN=Nt5e PE=1 SV=1	MOUSE	11
807	18.79	18.86	69.62	39.78	tr|Q547J4|Q547J4_MOUSE	Microtubule-associated protein OS=Mus musculus OX=10090 GN=Mapt PE=2 SV=1	MOUSE	19
808	18.78	18.94	35.32	29.59	tr|B1AT36|B1AT36_MOUSE	26S proteasome non-ATPase regulatory subunit 12 OS=Mus musculus OX=10090 GN=Psmd12 PE=1 SV=1	MOUSE	10
809	18.77	20.25	53.00	45.50	tr|Q544R9|Q544R9_MOUSE	High mobility group box 3 OS=Mus musculus OX=10090 GN=Hmgb3 PE=1 SV=1	MOUSE	13
810	18.76	18.89	39.72	37.50	tr|Q3TPJ8|Q3TPJ8_MOUSE	Cytoplasmic dynein 1 intermediate chain 2 OS=Mus musculus OX=10090 GN=Dync1i2 PE=1 SV=1	MOUSE	12
811	18.72	18.91	39.64	28.70	tr|Q3TYE5|Q3TYE5_MOUSE	Limbic system-associated membrane protein OS=Mus musculus OX=10090 GN=Lsamp PE=1 SV=1	MOUSE	11

812	18.72	18.76	69.80	69.80	tr|A0A0A0MQM0|A0A0A0MQM0_MOUSE	Eukaryotic translation initiation factor 5A (Fragment) OS=Mus musculus OX=10090 GN=Eif5a PE=1 SV=1	MOUSE	11
813	18.63	18.79	21.60	18.37	sp|Q80YV4-2|PANK4_MOUSE	Isoform 2 of 4'-phosphopantetheine phosphatase OS=Mus musculus OX=10090 GN=Pank4	MOUSE	11
814	18.61	18.67	59.76	37.20	tr|Q3T9Z2|Q3T9Z2_MOUSE	Uncharacterized protein OS=Mus musculus OX=10090 GN=Grhpr PE=1 SV=1	MOUSE	9
815	18.59	20.87	33.79	19.03	sp|Q9QYB8|ADDB_MOUSE	Beta-adducin OS=Mus musculus OX=10090 GN=Add2 PE=1 SV=4	MOUSE	11
816	18.58	18.7	12.95	9.74	sp|Q80XH2|IMPG2_MOUSE	Interphotoreceptor matrix proteoglycan 2 OS=Mus musculus OX=10090 GN=Impg2 PE=1 SV=1	MOUSE	10
817	18.58	18.64	22.04	21.70	tr|Q6PCX2|Q6PCX2_MOUSE	Transporter OS=Mus musculus OX=10090 GN=Slc6a1 PE=2 SV=1	MOUSE	14
818	18.52	18.64	26.06	21.97	sp|P57776-3|EF1D_MOUSE	Isoform 3 of Elongation factor 1-delta OS=Mus musculus OX=10090 GN=Eef1d	MOUSE	11
819	18.5	18.72	42.15	39.24	tr|Q8BG33|Q8BG33_MOUSE	Neurotrimin OS=Mus musculus OX=10090 GN=Ntm PE=1 SV=1	MOUSE	10
820	18.5	18.55	87.86	83.57	tr|Q5SX50|Q5SX50_MOUSE	Profilin OS=Mus musculus OX=10090 GN=Pfn1 PE=1 SV=1	MOUSE	11
821	18.44	18.52	58.27	51.50	sp|Q9D172|GAL3A_MOUSE	Glutamine amidotransferase-like class 1 domain-containing protein 3A, mitochondrial OS=Mus musculus OX=10090 GN=Gatd3a PE=1 SV=1	MOUSE	9
822	18.42	18.53	81.18	65.59	tr|Q3TF84|Q3TF84_MOUSE	Uncharacterized protein OS=Mus musculus OX=10090 GN=Lrrc59 PE=2 SV=1	MOUSE	15
823	18.36	18.42	21.77	14.26	tr|B2RUG7|B2RUG7_MOUSE	Zinc finger RNA binding protein OS=Mus musculus OX=10090 GN=Zfr PE=2 SV=1	MOUSE	11
824	18.33	18.45	43.04	28.61	sp|Q9EQP6|ARRC_MOUSE	Arrestin-C OS=Mus musculus OX=10090 GN=Arr3 PE=1 SV=1	MOUSE	10
825	18.32	18.46	47.07	35.90	sp|Q91WU5|AS3MT_MOUSE	Arsenite methyltransferase OS=Mus musculus OX=10090 GN=As3mt PE=1 SV=2	MOUSE	10
826	18.3	18.36	50.66	50.66	tr|Q8BRQ9|Q8BRQ9_MOUSE	Sidoreflexin OS=Mus musculus OX=10090 GN=Sfxn5 PE=1 SV=1	MOUSE	10
827	18.29	18.34	55.50	53.50	tr|Q14C38|Q14C38_MOUSE	Transcription elongation factor A (SII)-like 5 OS=Mus musculus OX=10090 GN=Tceal5 PE=1 SV=1	MOUSE	13

828	18.24	18.37	22.40	15.02	sp|Q3TCH7|CUL4A_MOUSE	Cullin-4A OS=Mus musculus OX=10090 GN=Cul4a PE=1 SV=1	MOUSE	10
829	18.22	18.25	64.32	56.43	tr|Q3TUI9|Q3TUI9_MOUSE	Proteasome subunit alpha type OS=Mus musculus OX=10090 GN=Psma5 PE=2 SV=1	MOUSE	9
830	18.21	18.26	26.98	25.90	tr|Q542P2|Q542P2_MOUSE	Uncharacterized protein OS=Mus musculus OX=10090 GN=Gpm6a PE=1 SV=1	MOUSE	12
831	18.2	18.24	47.93	36.21	sp|Q8BH95|ECHM_MOUSE	Enoyl-CoA hydratase, mitochondrial OS=Mus musculus OX=10090 GN=Echs1 PE=1 SV=1	MOUSE	9
832	18.18	18.8	50.81	34.59	sp|Q9CR16|PPID_MOUSE	Peptidyl-prolyl cis-trans isomerase D OS=Mus musculus OX=10090 GN=Ppid PE=1 SV=3	MOUSE	13
833	18.17	18.24	44.96	23.51	tr|A0A0B4J1E2|A0A0B4J1E2_MOUSE	SNW domain-containing protein 1 OS=Mus musculus OX=10090 GN=Snw1 PE=1 SV=1	MOUSE	8
834	18.14	18.15	48.37	46.34	sp|Q9QUM9|PSA6_MOUSE	Proteasome subunit alpha type-6 OS=Mus musculus OX=10090 GN=Psma6 PE=1 SV=1	MOUSE	13
835	18.13	18.24	44.35	29.71	sp|Q61655|DD19A_MOUSE	ATP-dependent RNA helicase DDX19A OS=Mus musculus OX=10090 GN=Ddx19a PE=1 SV=2	MOUSE	11
836	18.12	18.22	32.66	32.66	tr|Q9CXX6|Q9CXX6_MOUSE	Branched-chain-amino-acid aminotransferase OS=Mus musculus OX=10090 GN=Bcat1 PE=2 SV=1	MOUSE	11
837	18.12	18.16	57.53	51.37	tr|A4FUS1|A4FUS1_MOUSE	Rps16 protein OS=Mus musculus OX=10090 GN=Rps16 PE=1 SV=1	MOUSE	11
838	18.08	22.33	51.80	46.23	sp|Q9CX86|ROA0_MOUSE	Heterogeneous nuclear ribonucleoprotein A0 OS=Mus musculus OX=10090 GN=Hnrnpa0 PE=1 SV=1	MOUSE	13
839	18.07	22.58	57.76	50.93	sp|Q99JR1|SFXN1_MOUSE	Sideroflexin-1 OS=Mus musculus OX=10090 GN=Sfxn1 PE=1 SV=3	MOUSE	13
840	18.07	18.16	35.50	27.99	sp|Q9JMA1|UBP14_MOUSE	Ubiquitin carboxyl-terminal hydrolase 14 OS=Mus musculus OX=10090 GN=Usp14 PE=1 SV=3	MOUSE	10
841	18.06	18.12	45.30	36.59	sp|Q62189|SNRPA_MOUSE	U1 small nuclear ribonucleoprotein A OS=Mus musculus OX=10090 GN=Snrpa PE=1 SV=3	MOUSE	12
842	18.04	30.96	62.65	61.18	tr|Q3U9V4|Q3U9V4_MOUSE	WD_REPEATS_REGION domain-containing protein OS=Mus musculus OX=10090 GN=Gnb2 PE=1 SV=1	MOUSE	22
843	18.04	18.14	32.36	31.46	tr|Q3TML0|Q3TML0_MOUSE	Protein disulfide-isomerase A6 OS=Mus musculus OX=10090 GN=Pdia6 PE=1 SV=1	MOUSE	10
844	18.03	18.1	79.12	63.74	tr|Q543P7|Q543P7_MOUSE	Uncharacterized protein OS=Mus musculus OX=10090 GN=Arl3 PE=1 SV=1	MOUSE	13

845	18.02	18.64	33.79	23.38	tr|Q9D094|Q9D094_MOUSE	Histidine--tRNA ligase, cytoplasmic OS=Mus musculus OX=10090 GN=Hars PE=2 SV=1	MOUSE	11
846	18.01	18.08	44.93	43.01	tr|K3W4M4|K3W4M4_MOUSE	ATP-dependent (S)-NAD(P)H-hydrate dehydratase OS=Mus musculus OX=10090 GN=Naxd PE=1 SV=1	MOUSE	12
847	18	18	88.98	88.98	tr|Q3UNI8|Q3UNI8_MOUSE	D-dopachrome tautomerase OS=Mus musculus OX=10090 GN=Ddt PE=1 SV=1	MOUSE	9
848	18	18	16.43	16.43	sp|P31650|S6A11_MOUSE	Sodium- and chloride-dependent GABA transporter 3 OS=Mus musculus OX=10090 GN=Slc6a11 PE=1 SV=2	MOUSE	10
849	17.97	18.15	18.46	9.92	tr|Q8C943|Q8C943_MOUSE	Anion exchange protein (Fragment) OS=Mus musculus OX=10090 GN=Slc4a10 PE=2 SV=2	MOUSE	10
850	17.95	17.95	65.10	64.58	tr|Q5EBQ6|Q5EBQ6_MOUSE	60S ribosomal protein L9 OS=Mus musculus OX=10090 GN=Rpl9 PE=1 SV=1	MOUSE	10
851	17.92	18.3	19.29	7.27	tr|A0A1S6GWJ7|A0A1S6GWJ7_MOUSE	Uncharacterized protein OS=Mus musculus OX=10090 GN=Pi4ka PE=2 SV=1	MOUSE	11
852	17.85	91.26	87.61	86.94	sp|Q9D6F9|TBB4A_MOUSE	Tubulin beta-4A chain OS=Mus musculus OX=10090 GN=Tubb4a PE=1 SV=3	MOUSE	118
853	17.85	18.13	14.63	4.59	sp|Q9JLN9|MTOR_MOUSE	Serine/threonine-protein kinase mTOR OS=Mus musculus OX=10090 GN=Mtor PE=1 SV=2	MOUSE	9
854	17.84	28.27	81.38	69.64	tr|A8IP69|A8IP69_MOUSE	14-3-3 protein gamma subtype OS=Mus musculus OX=10090 GN=Ywhag PE=1 SV=1	MOUSE	16
855	17.83	18.04	27.95	20.82	tr|F8WHW6|F8WHW6_MOUSE	Phosphatidylinositol 4-phosphate 5-kinase type-1 gamma OS=Mus musculus OX=10090 GN=Pip5k1c PE=1 SV=1	MOUSE	11
856	17.8	17.9	26.33	23.13	tr|D3YU17|D3YU17_MOUSE	Nicalin OS=Mus musculus OX=10090 GN=Ncln PE=1 SV=1	MOUSE	10
857	17.76	17.86	46.84	42.24	sp|O54984|GET3_MOUSE	ATPase GET3 OS=Mus musculus OX=10090 GN=Get3 PE=1 SV=2	MOUSE	10
858	17.76	17.82	38.12	32.92	tr|A2AWI7|A2AWI7_MOUSE	Endophilin-B2 OS=Mus musculus OX=10090 GN=Sh3glb2 PE=1 SV=1	MOUSE	9
859	17.75	27.52	41.31	38.03	sp|P43275|H11_MOUSE	Histone H1.1 OS=Mus musculus OX=10090 GN=H1-1 PE=1 SV=2	MOUSE	29
860	17.75	17.78	57.77	52.43	tr|Q0PD38|Q0PD38_MOUSE	Ras-related protein Rab-18 OS=Mus musculus OX=10090 GN=Rab18 PE=1 SV=1	MOUSE	10
861	17.72	17.77	42.68	32.93	sp|Q924K1|AIPL1_MOUSE	Aryl-hydrocarbon-interacting protein-like 1 OS=Mus musculus OX=10090 GN=Aipl1 PE=2 SV=2	MOUSE	9

862	17.71	17.79	40.75	32.60	sp|Q9D2G2|ODO2_MOUSE	Dihydrolipoyllysine-residue succinyltransferase component of 2-oxoglutarate dehydrogenase complex, mitochondrial OS=Mus musculus OX=10090 GN=Dlst PE=1 SV=1	MOUSE	10
863	17.7	26.57	36.34	27.78	tr|Q5SVJ0|Q5SVJ0_MOUSE	Calcium/calmodulin-dependent protein kinase OS=Mus musculus OX=10090 GN=Camk2b PE=1 SV=1	MOUSE	20
864	17.69	23.61	16.24	11.47	sp|Q6R0H7|GNAS1_MOUSE	Guanine nucleotide-binding protein G(s) subunit alpha isoforms XLas OS=Mus musculus OX=10090 GN=Gnas PE=1 SV=1	MOUSE	15
865	17.69	17.8	36.64	36.13	tr|Q9JJE1|Q9JJE1_MOUSE	Brain cDNA, clone MNCb-1208, similar to Mus musculus mitogen activated protein kinase kinase 1 (Map2k1), mRNA OS=Mus musculus OX=10090 GN=Map2k1 PE=2 SV=1	MOUSE	11
866	17.68	17.78	53.37	49.52	tr|Q497E9|Q497E9_MOUSE	40S ribosomal protein S8 OS=Mus musculus OX=10090 GN=Rps8 PE=1 SV=1	MOUSE	10
867	17.66	17.69	49.83	44.55	tr|Q3UA17|Q3UA17_MOUSE	Uncharacterized protein OS=Mus musculus OX=10090 GN=Mtch2 PE=2 SV=1	MOUSE	9
868	17.64	17.7	48.03	47.24	sp|P11031|TCP4_MOUSE	Activated RNA polymerase II transcriptional coactivator p15 OS=Mus musculus OX=10090 GN=Sub1 PE=1 SV=3	MOUSE	9
869	17.62	18.73	41.36	41.36	tr|Q3UIT2|Q3UIT2_MOUSE	COP9 signalosome complex subunit 6 OS=Mus musculus OX=10090 GN=Cops6 PE=1 SV=1	MOUSE	10
870	17.6	17.65	46.90	37.97	sp|Q99J08|S14L2_MOUSE	SEC14-like protein 2 OS=Mus musculus OX=10090 GN=Sec14l2 PE=1 SV=1	MOUSE	9
871	17.6	17.64	41.75	33.51	sp|Q6ZWN5|RS9_MOUSE	40S ribosomal protein S9 OS=Mus musculus OX=10090 GN=Rps9 PE=1 SV=3	MOUSE	10
872	17.59	17.86	20.79	10.60	sp|Q9JLI8|SART3_MOUSE	Squamous cell carcinoma antigen recognized by T-cells 3 OS=Mus musculus OX=10090 GN=Sart3 PE=1 SV=1	MOUSE	9
873	17.52	17.64	31.86	20.54	tr|Q6NZD2|Q6NZD2_MOUSE	Sorting nexin-1 OS=Mus musculus OX=10090 GN=Snx1 PE=1 SV=1	MOUSE	10
874	17.49	17.72	52.05	28.85	sp|Q3UYC0|PPM1H_MOUSE	Protein phosphatase 1H OS=Mus musculus OX=10090 GN=Ppm1h PE=1 SV=1	MOUSE	9
875	17.49	17.61	45.30	26.21	sp|P68181|KAPCB_MOUSE	cAMP-dependent protein kinase catalytic subunit beta OS=Mus musculus OX=10090 GN=Prkacb PE=1 SV=2	MOUSE	11

876	17.48	17.83	24.47	14.63	sp|Q8CGY8|OGT1_MOUSE	UDP-N-acetylglucosamine--peptide N-acetylglucosaminyltransferase 110 kDa subunit OS=Mus musculus OX=10090 GN=Ogt PE=1 SV=2	MOUSE	11
877	17.48	17.7	21.32	14.95	sp|Q8C050|KS6A5_MOUSE	Ribosomal protein S6 kinase alpha-5 OS=Mus musculus OX=10090 GN=Rps6ka5 PE=1 SV=2	MOUSE	10
878	17.42	21.74	38.39	24.52	sp|Q8JZN7|MIRO2_MOUSE	Mitochondrial Rho GTPase 2 OS=Mus musculus OX=10090 GN=Rhot2 PE=1 SV=1	MOUSE	11
879	17.42	17.51	23.47	22.07	tr|Q3UK67|Q3UK67_MOUSE	Splicing factor 1 OS=Mus musculus OX=10090 GN=Sf1 PE=2 SV=1	MOUSE	11
880	17.37	17.45	84.15	76.83	sp|Q5M8N0|CNRP1_MOUSE	CB1 cannabinoid receptor-interacting protein 1 OS=Mus musculus OX=10090 GN=Cnrip1 PE=1 SV=1	MOUSE	10
881	17.36	17.77	30.80	27.90	tr|A2RS68|A2RS68_MOUSE	U1 small nuclear ribonucleoprotein 70 kDa OS=Mus musculus OX=10090 GN=Snrnp70 PE=2 SV=1	MOUSE	10
882	17.35	17.62	37.88	26.84	sp|Q8C878|UBA3_MOUSE	NEDD8-activating enzyme E1 catalytic subunit OS=Mus musculus OX=10090 GN=Uba3 PE=1 SV=2	MOUSE	9
883	17.28	17.44	24.44	17.31	tr|Q3TEX7|Q3TEX7_MOUSE	Dynamin-type G domain-containing protein OS=Mus musculus OX=10090 GN=Mfn2 PE=2 SV=1	MOUSE	10
884	17.28	17.36	53.21	44.53	sp|Q78IK4|MIC27_MOUSE	MICOS complex subunit Mic27 OS=Mus musculus OX=10090 GN=Apool PE=1 SV=1	MOUSE	9
885	17.27	17.31	44.68	44.68	tr|D3Z4T6|D3Z4T6_MOUSE	Neuronal growth regulator 1 OS=Mus musculus OX=10090 GN=Negr1 PE=1 SV=1	MOUSE	10
886	17.26	17.31	62.37	54.12	tr|Q4FZE6|Q4FZE6_MOUSE	40S ribosomal protein S7 OS=Mus musculus OX=10090 GN=Rps7 PE=2 SV=1	MOUSE	9
887	17.23	17.28	23.51	18.76	tr|Q8BSI9|Q8BSI9_MOUSE	Cadherin-2 OS=Mus musculus OX=10090 GN=Cdh2 PE=2 SV=1	MOUSE	10
888	17.23	17.26	51.41	51.00	sp|Q9WV55|VAPA_MOUSE	Vesicle-associated membrane protein-associated protein A OS=Mus musculus OX=10090 GN=Vapa PE=1 SV=2	MOUSE	11
889	17.21	17.36	58.44	48.87	sp|Q8CAY6|THIC_MOUSE	Acetyl-CoA acetyltransferase, cytosolic OS=Mus musculus OX=10090 GN=Acat2 PE=1 SV=2	MOUSE	9
890	17.21	17.28	49.34	44.74	tr|Q561N5|Q561N5_MOUSE	40S ribosomal protein S18 OS=Mus musculus OX=10090 GN=Rps18 PE=2 SV=1	MOUSE	11
891	17.2	17.31	73.24	64.79	tr|Q544F6|Q544F6_MOUSE	Cotl1 protein OS=Mus musculus OX=10090 GN=Cotl1 PE=1 SV=1	MOUSE	12

892	17.18	25.34	41.35	41.35	sp|P31324|KAP3_MOUSE	cAMP-dependent protein kinase type II-beta regulatory subunit OS=Mus musculus OX=10090 GN=Prkar2b PE=1 SV=3	MOUSE	13
893	17.18	17.26	66.67	37.22	tr|A0A0A1HAM8|A0A0A1HAM8_MOUSE	80K protein OS=Mus musculus OX=10090 GN=Marcks PE=2 SV=1	MOUSE	11
894	17.13	17.25	40.21	31.44	sp|Q08288|LYAR_MOUSE	Cell growth-regulating nucleolar protein OS=Mus musculus OX=10090 GN=Lyar PE=1 SV=2	MOUSE	9
895	17.13	17.23	37.84	27.52	sp|Q62418|DBNL_MOUSE	Drebrin-like protein OS=Mus musculus OX=10090 GN=Dbnl PE=1 SV=2	MOUSE	9
896	17.09	17.2	48.90	32.87	tr|Q3U5N4|Q3U5N4_MOUSE	Uncharacterized protein OS=Mus musculus OX=10090 GN=Adprh PE=1 SV=1	MOUSE	8
897	17.07	17.19	43.20	33.33	tr|D3YXC2|D3YXC2_MOUSE	Mitochondrial carrier homolog 1 (Fragment) OS=Mus musculus OX=10090 GN=Mtch1 PE=1 SV=2	MOUSE	10
898	17.03	17.08	40.80	39.88	sp|Q8C0M9|ASGL1_MOUSE	Isoaspartyl peptidase/L-asparaginase OS=Mus musculus OX=10090 GN=Asrgl1 PE=1 SV=1	MOUSE	11
899	17.01	17.1	38.82	32.94	sp|P57784|RU2A_MOUSE	U2 small nuclear ribonucleoprotein A' OS=Mus musculus OX=10090 GN=Snrpa1 PE=1 SV=2	MOUSE	11
900	17	17.03	63.52	54.10	tr|Q4VA29|Q4VA29_MOUSE	RNA transcription, translation and transport factor protein OS=Mus musculus OX=10090 GN=Rtraf PE=1 SV=1	MOUSE	9
901	16.97	17.21	24.07	15.40	tr|Q91WD8|Q91WD8_MOUSE	Solute carrier family 24 (Sodium/potassium/calcium exchanger), member 1 OS=Mus musculus OX=10090 GN=Slc24a1 PE=2 SV=1	MOUSE	10
902	16.93	17.17	55.59	40.78	sp|Q6PE01|SNR40_MOUSE	U5 small nuclear ribonucleoprotein 40 kDa protein OS=Mus musculus OX=10090 GN=Snrnp40 PE=1 SV=1	MOUSE	8
903	16.93	17.05	37.59	28.67	sp|Q9D7N9|APMAP_MOUSE	Adipocyte plasma membrane-associated protein OS=Mus musculus OX=10090 GN=Apmap PE=1 SV=1	MOUSE	9
904	16.88	17.07	34.59	24.91	tr|A0A3B2WCL5|A0A3B2WCL5_MOUSE	Ran-binding protein 3 OS=Mus musculus OX=10090 GN=Ranbp3 PE=1 SV=1	MOUSE	11
905	16.88	17.05	36.38	16.92	sp|O08582|GTPB1_MOUSE	GTP-binding protein 1 OS=Mus musculus OX=10090 GN=Gtpbp1 PE=1 SV=2	MOUSE	10
906	16.82	28.89	41.50	25.60	sp|Q61301-3|CTNA2_MOUSE	Isoform 3 of Catenin alpha-2 OS=Mus musculus OX=10090 GN=Ctnna2	MOUSE	16

907	16.81	16.9	67.68	50.84	sp|Q91VH6|MEMO1_MOUSE	Protein MEMO1 OS=Mus musculus OX=10090 GN=Memo1 PE=1 SV=1	MOUSE	9
908	16.81	16.89	46.62	39.53	tr|Q3U3F6|Q3U3F6_MOUSE	RRM domain-containing protein OS=Mus musculus OX=10090 GN=Raly PE=2 SV=1	MOUSE	9
909	16.74	17	24.87	15.31	tr|A2AWT5|A2AWT5_MOUSE	Nucleolar transcription factor 1 OS=Mus musculus OX=10090 GN=Ubtf PE=1 SV=1	MOUSE	12
910	16.71	16.83	64.86	58.56	tr|Q9CQB4|Q9CQB4_MOUSE	Cytochrome b-c1 complex subunit 7 OS=Mus musculus OX=10090 GN=Uqcrb PE=1 SV=1	MOUSE	10
911	16.66	16.73	30.69	22.77	tr|Q571F9|Q571F9_MOUSE	MKIAA4115 protein (Fragment) OS=Mus musculus OX=10090 GN=G3bp1 PE=2 SV=1	MOUSE	10
912	16.63	28.27	80.89	70.64	tr|B2M1R7|B2M1R7_MOUSE	Poly(rC)-binding protein 2 OS=Mus musculus OX=10090 GN=Pcbp2 PE=1 SV=1	MOUSE	18
913	16.63	16.66	46.90	35.86	sp|Q9CZX8|RS19_MOUSE	40S ribosomal protein S19 OS=Mus musculus OX=10090 GN=Rps19 PE=1 SV=3	MOUSE	8
914	16.6	16.8	51.18	41.41	sp|Q6PE15|ABHDA_MOUSE	Palmitoyl-protein thioesterase ABHD10, mitochondrial OS=Mus musculus OX=10090 GN=Abhd10 PE=1 SV=1	MOUSE	12
915	16.58	16.64	67.41	66.67	tr|Q58EU7|Q58EU7_MOUSE	Rbp1 protein OS=Mus musculus OX=10090 GN=Rbp1 PE=1 SV=1	MOUSE	12
916	16.57	18.92	20.50	14.84	tr|Q3UDQ7|Q3UDQ7_MOUSE	AAA domain-containing protein OS=Mus musculus OX=10090 GN=Afg3l2 PE=2 SV=1	MOUSE	11
917	16.56	16.69	82.71	46.62	sp|Q91ZZ3|SYUB_MOUSE	Beta-synuclein OS=Mus musculus OX=10090 GN=Sncb PE=1 SV=1	MOUSE	11
918	16.56	16.58	47.67	44.77	sp|P60824|CIRBP_MOUSE	Cold-inducible RNA-binding protein OS=Mus musculus OX=10090 GN=Cirbp PE=1 SV=1	MOUSE	9
919	16.55	16.79	18.67	12.48	tr|Q8CBB7|Q8CBB7_MOUSE	AP-1 complex subunit gamma OS=Mus musculus OX=10090 GN=Ap1g1 PE=1 SV=1	MOUSE	9
920	16.53	16.61	74.19	69.35	tr|Q80YU9|Q80YU9_MOUSE	Retinoblastoma binding protein 9 OS=Mus musculus OX=10090 GN=Rbbp9 PE=2 SV=1	MOUSE	10
921	16.51	16.59	31.53	20.91	tr|Q548L6|Q548L6_MOUSE	Glutamate decarboxylase OS=Mus musculus OX=10090 GN=Gad1 PE=1 SV=1	MOUSE	10
922	16.48	16.67	20.16	10.57	tr|V9GXQ9|V9GXQ9_MOUSE	Phosphoinositide phospholipase C OS=Mus musculus OX=10090 GN=Plcb4 PE=1 SV=1	MOUSE	9
923	16.48	16.61	22.19	14.70	sp|Q9CWF6|BBS2_MOUSE	Bardet-Biedl syndrome 2 protein homolog OS=Mus musculus OX=10090 GN=Bbs2 PE=1 SV=1	MOUSE	9

924	16.48	16.55	57.09	41.87	tr|Q543K9|Q543K9_MOUSE	Purine nucleoside phosphorylase OS=Mus musculus OX=10090 GN=Pnp PE=1 SV=1	MOUSE	8
925	16.48	16.5	55.23	51.74	sp|Q9DCJ5|NDUA8_MOUSE	NADH dehydrogenase [ubiquinone] 1 alpha subcomplex subunit 8 OS=Mus musculus OX=10090 GN=Ndufa8 PE=1 SV=3	MOUSE	9
926	16.47	16.49	22.45	19.56	tr|H3BJW3|H3BJW3_MOUSE	Cleavage and polyadenylation specificity factor subunit 6 OS=Mus musculus OX=10090 GN=Cpsf6 PE=1 SV=1	MOUSE	8
927	16.46	16.58	38.60	25.80	tr|Q9JKY7|Q9JKY7_MOUSE	Cytochrome P450 CYP2D22 OS=Mus musculus OX=10090 GN=Cyp2d22 PE=1 SV=1	MOUSE	9
928	16.44	16.73	32.01	17.61	sp|O08795-2|GLU2B_MOUSE	Isoform 2 of Glucosidase 2 subunit beta OS=Mus musculus OX=10090 GN=Prkcsh	MOUSE	9
929	16.44	16.56	41.05	30.53	sp|Q9JK81|MYG1_MOUSE	MYG1 exonuclease OS=Mus musculus OX=10090 GN=Myg1 PE=1 SV=1	MOUSE	10
930	16.43	16.52	42.12	35.96	tr|Q543F6|Q543F6_MOUSE	Uncharacterized protein OS=Mus musculus OX=10090 GN=Cdk5 PE=1 SV=1	MOUSE	9
931	16.43	16.5	23.66	16.83	sp|Q64737|PUR2_MOUSE	Trifunctional purine biosynthetic protein adenosine-3 OS=Mus musculus OX=10090 GN=Gart PE=1 SV=3	MOUSE	9
932	16.42	16.44	46.69	44.75	sp|P20108|PRDX3_MOUSE	Thioredoxin-dependent peroxide reductase, mitochondrial OS=Mus musculus OX=10090 GN=Prdx3 PE=1 SV=1	MOUSE	10
933	16.39	16.55	23.29	8.31	sp|Q8K010|OPLA_MOUSE	5-oxoprolinase OS=Mus musculus OX=10090 GN=Oplah PE=1 SV=1	MOUSE	7
934	16.38	22.66	53.02	43.72	sp|O88712-2|CTBP1_MOUSE	Isoform 2 of C-terminal-binding protein 1 OS=Mus musculus OX=10090 GN=Ctbp1	MOUSE	12
935	16.38	18.43	51.32	38.16	tr|Q91Z50|Q91Z50_MOUSE	Flap endonuclease 1 OS=Mus musculus OX=10090 GN=Fen1 PE=1 SV=1	MOUSE	9
936	16.36	18.49	51.77	46.45	sp|Q920Q6-3|MSI2H_MOUSE	Isoform 3 of RNA-binding protein Musashi homolog 2 OS=Mus musculus OX=10090 GN=Msi2	MOUSE	10
937	16.32	16.39	47.81	38.32	sp|Q9CR68|UCRI_MOUSE	Cytochrome b-c1 complex subunit Rieske, mitochondrial OS=Mus musculus OX=10090 GN=Uqcrfs1 PE=1 SV=1	MOUSE	9
938	16.3	32.43	20.85	15.97	sp|Q5SDA5|GUC2F_MOUSE	Retinal guanylyl cyclase 2 OS=Mus musculus OX=10090 GN=Gucy2f PE=1 SV=1	MOUSE	16
939	16.3	20.59	48.17	38.21	sp|Q9Z130|HNRDL_MOUSE	Heterogeneous nuclear ribonucleoprotein D-like OS=Mus musculus OX=10090 GN=Hnrnpdl PE=1 SV=1	MOUSE	12

940	16.27	16.36	34.41	28.93	tr|Q5RKP0|Q5RKP0_MOUSE	Vat1 protein (Fragment) OS=Mus musculus OX=10090 GN=Vat1 PE=2 SV=1	MOUSE	9
941	16.23	16.32	85.35	63.13	tr|Q60I30|Q60I30_MOUSE	Inosine triphosphate pyrophosphatase OS=Mus musculus OX=10090 GN=Itpa PE=1 SV=1	MOUSE	9
942	16.21	18.54	29.10	13.29	tr|Q8BTX6|Q8BTX6_MOUSE	Uncharacterized protein (Fragment) OS=Mus musculus OX=10090 GN=Mtrex PE=2 SV=1	MOUSE	9
943	16.21	16.66	8.42	3.93	sp|Q9ERU9|RBP2_MOUSE	E3 SUMO-protein ligase RanBP2 OS=Mus musculus OX=10090 GN=Ranbp2 PE=1 SV=2	MOUSE	10
944	16.2	16.26	61.67	52.08	tr|Q6RI64|Q6RI64_MOUSE	Proteasome subunit beta OS=Mus musculus OX=10090 GN=Psmb1 PE=1 SV=1	MOUSE	9
945	16.17	16.26	37.30	29.60	tr|Q505L1|Q505L1_MOUSE	Actl6a protein OS=Mus musculus OX=10090 GN=Actl6a PE=1 SV=1	MOUSE	8
946	16.15	16.21	66.88	47.13	sp|Q61425|HCDH_MOUSE	Hydroxyacyl-coenzyme A dehydrogenase, mitochondrial OS=Mus musculus OX=10090 GN=Hadh PE=1 SV=2	MOUSE	8
947	16.14	16.23	32.05	15.56	tr|Q05DV1|Q05DV1_MOUSE	NADPH--hemoprotein reductase OS=Mus musculus OX=10090 GN=Por PE=1 SV=1	MOUSE	9
948	16.14	16.18	78.57	78.57	sp|P70349|HINT1_MOUSE	Histidine triad nucleotide-binding protein 1 OS=Mus musculus OX=10090 GN=Hint1 PE=1 SV=3	MOUSE	9
949	16.12	18.4	60.58	51.92	tr|Q0PD53|Q0PD53_MOUSE	Rab6B OS=Mus musculus OX=10090 GN=Rab6b PE=1 SV=1	MOUSE	10
950	16.09	16.25	25.43	18.52	tr|A0A5F8MPZ2|A0A5F8MPZ2_MOUSE	Kinesin light chain OS=Mus musculus OX=10090 GN=Klc1 PE=1 SV=1	MOUSE	9
951	16.05	16.06	24.91	24.91	sp|Q8BGN8-2|SYNPR_MOUSE	Isoform 2 of Synaptoporin OS=Mus musculus OX=10090 GN=Synpr	MOUSE	9
952	16.02	16.13	29.11	17.84	sp|Q3UDE2|TTL12_MOUSE	Tubulin--tyrosine ligase-like protein 12 OS=Mus musculus OX=10090 GN=Ttll12 PE=1 SV=1	MOUSE	8
953	16.02	16.03	18.23	18.23	tr|Q8C7W8|Q8C7W8_MOUSE	Amino acid transporter OS=Mus musculus OX=10090 GN=Slc1a3 PE=2 SV=1	MOUSE	12
954	16	18	50.26	49.74	sp|P60766|CDC42_MOUSE	Cell division control protein 42 homolog OS=Mus musculus OX=10090 GN=Cdc42 PE=1 SV=2	MOUSE	10
955	15.94	16.19	25.12	14.06	sp|Q8CH09|SUGP2_MOUSE	SURP and G-patch domain-containing protein 2 OS=Mus musculus OX=10090 GN=Sugp2 PE=1 SV=2	MOUSE	9
956	15.93	24.81	34.82	22.96	tr|Q3UAT9|Q3UAT9_MOUSE	Inosine-5'-monophosphate dehydrogenase OS=Mus musculus OX=10090 GN=Impdh2 PE=1 SV=1	MOUSE	12

957	15.92	16.28	31.48	16.90	tr|Q7TQD1|Q7TQD1_MOUSE	Ubiquitinyl hydrolase 1 OS=Mus musculus OX=10090 GN=Tnfaip3 PE=2 SV=1	MOUSE	10
958	15.84	15.92	27.18	23.12	tr|Q8C2E6|Q8C2E6_MOUSE	Monocarboxylate transporter 1 OS=Mus musculus OX=10090 GN=Slc16a1 PE=2 SV=1	MOUSE	8
959	15.84	15.92	44.07	24.41	sp|Q9CY64|BIEA_MOUSE	Biliverdin reductase A OS=Mus musculus OX=10090 GN=Blvra PE=1 SV=1	MOUSE	7
960	15.81	21.49	46.76	32.64	tr|A0A0J9YUL3|A0A0J9YUL3_MOUSE	Septin OS=Mus musculus OX=10090 GN=Septin11 PE=1 SV=1	MOUSE	11
961	15.78	16.26	27.40	23.12	tr|Q3URK9|Q3URK9_MOUSE	Uncharacterized protein OS=Mus musculus OX=10090 GN=Trim2 PE=2 SV=1	MOUSE	13
962	15.75	15.78	52.85	47.91	tr|Q543N3|Q543N3_MOUSE	LIM and SH3 domain protein 1 OS=Mus musculus OX=10090 GN=Lasp1 PE=1 SV=1	MOUSE	12
963	15.74	15.87	15.79	5.80	sp|A2A5R2|BIG2_MOUSE	Brefeldin A-inhibited guanine nucleotide-exchange protein 2 OS=Mus musculus OX=10090 GN=Arfgef2 PE=1 SV=1	MOUSE	8
964	15.74	15.78	54.63	54.63	sp|Q91WS0|CISD1_MOUSE	CDGSH iron-sulfur domain-containing protein 1 OS=Mus musculus OX=10090 GN=Cisd1 PE=1 SV=1	MOUSE	8
965	15.72	16.07	13.46	7.93	sp|B2RXS4|PLXB2_MOUSE	Plexin-B2 OS=Mus musculus OX=10090 GN=Plxnb2 PE=1 SV=1	MOUSE	9
966	15.71	15.82	29.87	18.12	tr|Q3TMW7|Q3TMW7_MOUSE	Uncharacterized protein OS=Mus musculus OX=10090 GN=Glmn PE=2 SV=1	MOUSE	10
967	15.67	15.75	41.62	31.62	tr|Q9CY29|Q9CY29_MOUSE	26S proteasome non-ATPase regulatory subunit 4 OS=Mus musculus OX=10090 GN=Psmd4 PE=2 SV=1	MOUSE	9
968	15.66	15.8	23.71	14.43	tr|A3KG93|A3KG93_MOUSE	Lysine-specific histone demethylase OS=Mus musculus OX=10090 GN=Kdm1a PE=1 SV=1	MOUSE	9
969	15.66	15.75	34.31	26.64	sp|O70194|EIF3D_MOUSE	Eukaryotic translation initiation factor 3 subunit D OS=Mus musculus OX=10090 GN=Eif3d PE=1 SV=2	MOUSE	11
970	15.63	15.73	45.71	31.43	tr|B2RUC7|B2RUC7_MOUSE	Serine/threonine kinase receptor associated protein OS=Mus musculus OX=10090 GN=Strap PE=1 SV=1	MOUSE	8
971	15.62	15.71	16.64	14.63	sp|P32020|SCP2_MOUSE	Sterol carrier protein 2 OS=Mus musculus OX=10090 GN=Scp2 PE=1 SV=3	MOUSE	10
972	15.58	15.74	49.28	43.54	tr|B7ZWI2|B7ZWI2_MOUSE	1110005A23Rik protein OS=Mus musculus OX=10090 GN=Sarnp PE=2 SV=1	MOUSE	9

973	15.56	15.66	45.70	41.72	tr|O70569|O70569_MOUSE	Ribosomal protein S14 OS=Mus musculus OX=10090 GN=rps14 PE=3 SV=1	MOUSE	8
974	15.53	15.61	70.63	66.87	sp|Q80WM3|CPLX4_MOUSE	Complexin-4 OS=Mus musculus OX=10090 GN=Cplx4 PE=1 SV=2	MOUSE	8
975	15.52	15.57	54.02	34.10	sp|Q9R1P0|PSA4_MOUSE	Proteasome subunit alpha type-4 OS=Mus musculus OX=10090 GN=Psma4 PE=1 SV=1	MOUSE	8
976	15.51	15.6	33.26	25.05	tr|Q3TKP3|Q3TKP3_MOUSE	Ataxin-10 OS=Mus musculus OX=10090 GN=Atxn10 PE=2 SV=1	MOUSE	9
977	15.49	15.9	21.27	16.26	tr|D3YWN7|D3YWN7_MOUSE	Ensconsin OS=Mus musculus OX=10090 GN=Map7 PE=1 SV=2	MOUSE	9
978	15.48	15.58	65.36	65.36	sp|Q9CQJ8|NDUB9_MOUSE	NADH dehydrogenase [ubiquinone] 1 beta subcomplex subunit 9 OS=Mus musculus OX=10090 GN=Ndufb9 PE=1 SV=3	MOUSE	10
979	15.47	15.54	47.65	43.68	sp|Q9EQU5-2|SET_MOUSE	Isoform 2 of Protein SET OS=Mus musculus OX=10090 GN=Set	MOUSE	9
980	15.46	15.65	68.67	36.14	tr|Q99M52|Q99M52_MOUSE	Sorting nexin 12 OS=Mus musculus OX=10090 GN=Snx12 PE=2 SV=1	MOUSE	8
981	15.44	15.54	55.83	50.31	tr|B0QZN5|B0QZN5_MOUSE	Synaptobrevin-2 OS=Mus musculus OX=10090 GN=Vamp2 PE=1 SV=1	MOUSE	12
982	15.42	17.04	37.33	24.89	tr|Q8R3X4|Q8R3X4_MOUSE	Timm44 protein (Fragment) OS=Mus musculus OX=10090 GN=Timm44 PE=2 SV=1	MOUSE	11
983	15.41	15.51	41.41	26.52	sp|Q9CZ30|OLA1_MOUSE	Obg-like ATPase 1 OS=Mus musculus OX=10090 GN=Ola1 PE=1 SV=1	MOUSE	8
984	15.37	15.39	36.20	32.94	sp|Q9CQM9|GLRX3_MOUSE	Glutaredoxin-3 OS=Mus musculus OX=10090 GN=Glrx3 PE=1 SV=1	MOUSE	9
985	15.31	15.56	19.81	9.71	tr|B1AS46|B1AS46_MOUSE	Zinc finger protein castor homolog 1 OS=Mus musculus OX=10090 GN=Casz1 PE=1 SV=1	MOUSE	9
986	15.31	15.41	50.72	45.65	tr|Q5D098|Q5D098_MOUSE	Proteasome subunit beta (Fragment) OS=Mus musculus OX=10090 GN=Psmb7 PE=2 SV=1	MOUSE	11
987	15.27	15.35	55.30	38.11	sp|Q9JM96|BORG4_MOUSE	Cdc42 effector protein 4 OS=Mus musculus OX=10090 GN=Cdc42ep4 PE=1 SV=1	MOUSE	9
988	15.27	15.32	38.94	33.04	tr|Q3TVS6|Q3TVS6_MOUSE	Cathepsin B OS=Mus musculus OX=10090 GN=Ctsb PE=2 SV=1	MOUSE	9

989	15.26	15.46	22.64	11.27	tr|G3X956|G3X956_MOUSE	FACT complex subunit OS=Mus musculus OX=10090 GN=Supt16 PE=1 SV=1	MOUSE	10
990	15.26	15.31	32.48	26.75	tr|A0A0R4J023|A0A0R4J023_MOUSE	Methylglutaconyl-CoA hydratase, mitochondrial OS=Mus musculus OX=10090 GN=Auh PE=1 SV=1	MOUSE	9
991	15.2	15.39	31.88	12.08	tr|Q80VM5|Q80VM5_MOUSE	Dipeptidyl aminopeptidase-like protein 6 OS=Mus musculus OX=10090 GN=Dpp6 PE=1 SV=1	MOUSE	7
992	15.16	15.2	81.63	63.95	tr|Q8BME2|Q8BME2_MOUSE	NADH dehydrogenase [ubiquinone] 1 alpha subcomplex subunit 12 OS=Mus musculus OX=10090 GN=Ndufa12 PE=2 SV=1	MOUSE	8
993	15.13	15.16	26.24	24.03	sp|Q8K2C9|HACD3_MOUSE	Very-long-chain (3R)-3-hydroxyacyl-CoA dehydratase 3 OS=Mus musculus OX=10090 GN=Hacd3 PE=1 SV=2	MOUSE	10
994	15.13	15.16	35.52	28.21	sp|Q8BWT1|THIM_MOUSE	3-ketoacyl-CoA thiolase, mitochondrial OS=Mus musculus OX=10090 GN=Acaa2 PE=1 SV=3	MOUSE	8
995	15.12	15.2	36.87	33.41	tr|Q6PFB2|Q6PFB2_MOUSE	Rcc1 protein OS=Mus musculus OX=10090 GN=Rcc1 PE=1 SV=1	MOUSE	8
996	15.02	15.08	47.94	36.08	tr|Q8VCV2|Q8VCV2_MOUSE	N-myc downstream-regulated gene 3 protein OS=Mus musculus OX=10090 GN=Ndrg3 PE=1 SV=1	MOUSE	8
997	14.92	15.02	31.66	30.34	sp|Q61081|CDC37_MOUSE	Hsp90 co-chaperone Cdc37 OS=Mus musculus OX=10090 GN=Cdc37 PE=1 SV=1	MOUSE	9
998	14.92	14.94	51.20	51.20	tr|Q9DAB3|Q9DAB3_MOUSE	Uncharacterized protein OS=Mus musculus OX=10090 GN=Arl8b PE=2 SV=1	MOUSE	8
999	14.88	15.02	28.71	18.39	sp|Q8K3H0|DP13A_MOUSE	DCC-interacting protein 13-alpha OS=Mus musculus OX=10090 GN=Appl1 PE=1 SV=1	MOUSE	8
1000	14.86	14.9	17.28	14.84	tr|Q3U2J2|Q3U2J2_MOUSE	Glucose transporter type 1, erythrocyte/brain OS=Mus musculus OX=10090 GN=Slc2a1 PE=2 SV=1	MOUSE	10
1001	14.84	15.43	37.27	30.34	tr|Q3UK27|Q3UK27_MOUSE	NEDD8-activating enzyme E1 regulatory subunit OS=Mus musculus OX=10090 GN=Nae1 PE=1 SV=1	MOUSE	10
1002	14.84	14.89	15.51	12.13	tr|Q8JZN2|Q8JZN2_MOUSE	Cold shock domain-containing protein E1 OS=Mus musculus OX=10090 GN=Csde1 PE=1 SV=1	MOUSE	8
1003	14.82	14.9	20.37	16.50	tr|Q542V3|Q542V3_MOUSE	Serine/arginine-rich-splicing factor 4 OS=Mus musculus OX=10090 GN=Srsf4 PE=1 SV=1	MOUSE	9
1004	14.81	14.95	75.39	54.45	tr|Q4W4C9|Q4W4C9_MOUSE	Visinin-like protein 1 OS=Mus musculus OX=10090 GN=Vsnl1 PE=1 SV=1	MOUSE	9

1005	14.77	14.88	34.16	25.69	tr|Q3UMP4|Q3UMP4_MOUSE	Plasminogen activator inhibitor 1 RNA-binding protein OS=Mus musculus OX=10090 GN=Serbp1 PE=1 SV=1	MOUSE	7
1006	14.76	15.33	75.00	65.95	tr|Q3TJC2|Q3TJC2_MOUSE	SGNH_hydro domain-containing protein OS=Mus musculus OX=10090 GN=Pafah1b3 PE=1 SV=1	MOUSE	10
1007	14.76	15.31	22.18	12.19	tr|B7ZW94|B7ZW94_MOUSE	Family with sequence similarity 120, member C OS=Mus musculus OX=10090 GN=Fam120c PE=2 SV=1	MOUSE	9
1008	14.74	15.46	16.92	13.30	sp|O88704|HCN1_MOUSE	Potassium/sodium hyperpolarization-activated cyclic nucleotide-gated channel 1 OS=Mus musculus OX=10090 GN=Hcn1 PE=1 SV=1	MOUSE	8
1009	14.72	14.79	22.05	15.41	tr|Q8C7F9|Q8C7F9_MOUSE	Transmembrane 9 superfamily member OS=Mus musculus OX=10090 GN=Tm9sf2 PE=2 SV=1	MOUSE	8
1010	14.68	14.73	50.35	50.35	tr|Q8BT90|Q8BT90_MOUSE	40S ribosomal protein S17 (Fragment) OS=Mus musculus OX=10090 GN=Rps17 PE=2 SV=1	MOUSE	9
1011	14.64	14.68	27.60	24.68	sp|Q9CY27|TECR_MOUSE	Very-long-chain enoyl-CoA reductase OS=Mus musculus OX=10090 GN=Tecr PE=1 SV=1	MOUSE	8
1012	14.61	14.68	33.81	31.67	tr|Q7TMG8|Q7TMG8_MOUSE	Glioblastoma amplified sequence OS=Mus musculus OX=10090 GN=Nipsnap2 PE=1 SV=1	MOUSE	8
1013	14.61	14.64	39.55	32.77	tr|C9K0Z7|C9K0Z7_MOUSE	Peptidylprolyl isomerase OS=Mus musculus OX=10090 GN=Fkbp8 PE=2 SV=1	MOUSE	7
1014	14.59	14.61	41.67	33.33	tr|Q8K0E9|Q8K0E9_MOUSE	Dhdh protein (Fragment) OS=Mus musculus OX=10090 GN=Dhdh PE=2 SV=1	MOUSE	8
1015	14.56	14.7	39.38	26.64	sp|Q91WN1|DNJC9_MOUSE	DnaJ homolog subfamily C member 9 OS=Mus musculus OX=10090 GN=Dnajc9 PE=1 SV=2	MOUSE	8
1016	14.55	14.57	62.57	43.27	tr|F7CDT0|F7CDT0_MOUSE	NEDD8-conjugating enzyme Ubc12 (Fragment) OS=Mus musculus OX=10090 GN=Ube2m PE=1 SV=1	MOUSE	7
1017	14.47	14.72	18.69	11.20	tr|A2AFF6|A2AFF6_MOUSE	Cohesin subunit SA-2 OS=Mus musculus OX=10090 GN=Stag2 PE=1 SV=1	MOUSE	11
1018	14.46	14.52	44.30	35.23	sp|Q8VD12|Z385A_MOUSE	Zinc finger protein 385A OS=Mus musculus OX=10090 GN=Znf385a PE=1 SV=2	MOUSE	8
1019	14.45	14.82	26.09	11.67	sp|Q9QZE5|COPG1_MOUSE	Coatomer subunit gamma-1 OS=Mus musculus OX=10090 GN=Copg1 PE=1 SV=1	MOUSE	7
1020	14.44	14.55	28.57	16.96	tr|B2KGP3|B2KGP3_MOUSE	Protein phosphatase 1E (PP2C domain containing) OS=Mus musculus OX=10090 GN=Ppm1e PE=1 SV=1	MOUSE	9

1021	14.43	14.49	33.86	24.83	sp|P36552|HEM6_MOUSE	Oxygen-dependent coproporphyrinogen-III oxidase, mitochondrial OS=Mus musculus OX=10090 GN=Cpox PE=1 SV=2	MOUSE	8
1022	14.41	14.49	26.29	17.21	tr|B1AX58|B1AX58_MOUSE	Plastin-3 OS=Mus musculus OX=10090 GN=Pls3 PE=1 SV=1	MOUSE	8
1023	14.35	14.36	52.32	39.74	tr|Q5BLJ7|Q5BLJ7_MOUSE	40S ribosomal protein S13 OS=Mus musculus OX=10090 GN=Rps13 PE=1 SV=1	MOUSE	10
1024	14.34	14.49	22.86	18.64	tr|Q3UHW2|Q3UHW2_MOUSE	Uncharacterized protein OS=Mus musculus OX=10090 GN=Hsd17b4 PE=2 SV=1	MOUSE	9
1025	14.33	14.51	30.39	15.59	sp|Q61550|RAD21_MOUSE	Double-strand-break repair protein rad21 homolog OS=Mus musculus OX=10090 GN=Rad21 PE=1 SV=3	MOUSE	8
1026	14.32	24.7	70.48	64.32	tr|Q542T7|Q542T7_MOUSE	Ras-related protein Rab-3 OS=Mus musculus OX=10090 GN=Rab3c PE=1 SV=1	MOUSE	16
1027	14.32	16.56	45.66	35.55	tr|D3Z3N4|D3Z3N4_MOUSE	Heterogeneous nuclear ribonucleoprotein H3 OS=Mus musculus OX=10090 GN=Hnrnph3 PE=1 SV=1	MOUSE	10
1028	14.3	14.46	26.98	22.34	sp|Q60900|ELAV3_MOUSE	ELAV-like protein 3 OS=Mus musculus OX=10090 GN=Elavl3 PE=1 SV=1	MOUSE	8
1029	14.3	14.31	47.95	42.47	sp|Q9D1D4|TMEDA_MOUSE	Transmembrane emp24 domain-containing protein 10 OS=Mus musculus OX=10090 GN=Tmed10 PE=1 SV=1	MOUSE	9
1030	14.26	14.39	66.83	42.93	sp|P62696|CRBB2_MOUSE	Beta-crystallin B2 OS=Mus musculus OX=10090 GN=Crybb2 PE=1 SV=2	MOUSE	10
1031	14.23	14.34	24.14	19.38	tr|A0A5F8MPK9|A0A5F8MPK9_MOUSE	Phosphatidylinositol-3-phosphatase SAC1 OS=Mus musculus OX=10090 GN=Sacm1l PE=1 SV=1	MOUSE	9
1032	14.23	14.29	30.86	25.68	sp|Q99L43|CDS2_MOUSE	Phosphatidate cytidylyltransferase 2 OS=Mus musculus OX=10090 GN=Cds2 PE=1 SV=1	MOUSE	8
1033	14.18	14.37	30.50	14.86	tr|Q6GTL7|Q6GTL7_MOUSE	6-phosphofructo-2-kinase OS=Mus musculus OX=10090 GN=Pfkfb2 PE=1 SV=1	MOUSE	9
1034	14.18	14.23	44.24	34.85	sp|Q8BTX9|HSDL1_MOUSE	Inactive hydroxysteroid dehydrogenase-like protein 1 OS=Mus musculus OX=10090 GN=Hsdl1 PE=1 SV=1	MOUSE	8
1035	14.13	14.32	26.92	21.63	tr|Q3UJ95|Q3UJ95_MOUSE	Uncharacterized protein OS=Mus musculus OX=10090 GN=Pip4k2b PE=1 SV=1	MOUSE	7
1036	14.13	14.13	31.21	29.48	sp|Q9WUM5|SUCA_MOUSE	Succinate--CoA ligase [ADP/GDP-forming] subunit alpha, mitochondrial OS=Mus musculus OX=10090 GN=Suclg1 PE=1 SV=4	MOUSE	8

1037	14.1	14.22	35.97	32.70	sp|Q8CG76|ARK72_MOUSE	Aflatoxin B1 aldehyde reductase member 2 OS=Mus musculus OX=10090 GN=Akr7a2 PE=1 SV=3	MOUSE	8
1038	14.1	14.19	54.91	36.16	tr|Q3UBU9|Q3UBU9_MOUSE	Peptidylprolyl isomerase OS=Mus musculus OX=10090 GN=Fkbp3 PE=1 SV=1	MOUSE	8
1039	14.09	14.13	45.81	35.20	tr|Q8C1Y3|Q8C1Y3_MOUSE	H15 domain-containing protein OS=Mus musculus OX=10090 GN=H1f0 PE=2 SV=1	MOUSE	9
1040	14.07	14.23	41.31	33.50	tr|Q9Z1A1|Q9Z1A1_MOUSE	TFG protein OS=Mus musculus OX=10090 GN=Tfg PE=1 SV=1	MOUSE	11
1041	14.07	14.12	58.33	46.08	sp|Q99PT1|GDIR1_MOUSE	Rho GDP-dissociation inhibitor 1 OS=Mus musculus OX=10090 GN=Arhgdia PE=1 SV=3	MOUSE	10
1042	14.06	39.8	22.70	20.48	tr|H2BL43|H2BL43_MOUSE	Calcium-transporting ATPase OS=Mus musculus OX=10090 GN=Atp2b4 PE=2 SV=1	MOUSE	21
1043	14.06	14.06	56.43	56.43	sp|Q9JJV2|PROF2_MOUSE	Profilin-2 OS=Mus musculus OX=10090 GN=Pfn2 PE=1 SV=3	MOUSE	10
1044	14.03	14.03	30.67	24.37	tr|Q3THA6|Q3THA6_MOUSE	Uncharacterized protein OS=Mus musculus OX=10090 GN=Srsf7 PE=2 SV=1	MOUSE	7
1045	14.02	14.02	34.26	26.54	sp|Q921M7|CYRIB_MOUSE	CYFIP-related Rac1 interactor B OS=Mus musculus OX=10090 GN=Cyrib PE=1 SV=1	MOUSE	8
1046	14.01	14.09	20.80	15.21	tr|Q3UYK6|Q3UYK6_MOUSE	Amino acid transporter OS=Mus musculus OX=10090 GN=Slc1a2 PE=2 SV=1	MOUSE	8
1047	14.01	14.04	57.54	48.81	sp|Q9CXS4|CENPV_MOUSE	Centromere protein V OS=Mus musculus OX=10090 GN=Cenpv PE=1 SV=2	MOUSE	8
1048	14	32.16	65.31	56.85	tr|Q9D7B3|Q9D7B3_MOUSE	Uncharacterized protein (Fragment) OS=Mus musculus OX=10090 GN=Gnat2 PE=2 SV=2	MOUSE	18
1049	14	14	56.07	55.49	tr|Q569M7|Q569M7_MOUSE	Cryaa protein OS=Mus musculus OX=10090 GN=Cryaa PE=2 SV=1	MOUSE	7
1050	13.98	14.13	28.83	18.75	tr|Q3UK24|Q3UK24_MOUSE	WD_REPEATS_REGION domain-containing protein OS=Mus musculus OX=10090 GN=Wdr37 PE=2 SV=1	MOUSE	7
1051	13.98	14.02	54.24	44.07	tr|Q5HZY7|Q5HZY7_MOUSE	V-type proton ATPase subunit G OS=Mus musculus OX=10090 GN=Atp6v1g1 PE=1 SV=1	MOUSE	7
1052	13.96	14.01	28.40	25.05	tr|Q99PH4|Q99PH4_MOUSE	Aldehyde dehydrogenase OS=Mus musculus OX=10090 GN=Aldh3a2 PE=3 SV=1	MOUSE	9
1053	13.95	14.09	37.23	33.58	tr|Q8BQL7|Q8BQL7_MOUSE	CUE domain-containing protein OS=Mus musculus OX=10090 GN=Tollip PE=2 SV=1	MOUSE	9

1054	13.95	14.07	36.53	16.83	tr|Q3UCF2|Q3UCF2_MOUSE	Uncharacterized protein OS=Mus musculus OX=10090 GN=Cdc5l PE=1 SV=1	MOUSE	7
1055	13.94	41.82	44.75	41.10	sp|P43274|H14_MOUSE	Histone H1.4 OS=Mus musculus OX=10090 GN=H1-4 PE=1 SV=2	MOUSE	40
1056	13.94	13.95	67.41	62.22	sp|P52760|RIDA_MOUSE	2-iminobutanoate/2-iminopropanoate deaminase OS=Mus musculus OX=10090 GN=Rida PE=1 SV=3	MOUSE	7
1057	13.91	13.94	38.19	34.63	tr|G5E8T9|G5E8T9_MOUSE	Hydroxyacylglutathione hydrolase, mitochondrial OS=Mus musculus OX=10090 GN=Hagh PE=1 SV=1	MOUSE	7
1058	13.9	14.05	26.42	20.57	sp|Q3TIX9|SNUT2_MOUSE	U4/U6.U5 tri-snRNP-associated protein 2 OS=Mus musculus OX=10090 GN=Usp39 PE=1 SV=2	MOUSE	8
1059	13.9	13.94	60.11	42.55	tr|F8WJG3|F8WJG3_MOUSE	Transformer-2 protein homolog beta OS=Mus musculus OX=10090 GN=Tra2b PE=1 SV=1	MOUSE	7
1060	13.89	13.92	29.56	26.60	tr|Q3UGB5|Q3UGB5_MOUSE	DAZ-associated protein 1 OS=Mus musculus OX=10090 GN=Dazap1 PE=1 SV=1	MOUSE	8
1061	13.87	13.94	28.59	14.23	sp|Q61102|ABCB7_MOUSE	Iron-sulfur clusters transporter ABCB7, mitochondrial OS=Mus musculus OX=10090 GN=Abcb7 PE=1 SV=3	MOUSE	7
1062	13.87	13.9	23.90	22.43	sp|Q5SUF2-3|LC7L3_MOUSE	Isoform 3 of Luc7-like protein 3 OS=Mus musculus OX=10090 GN=Luc7l3	MOUSE	7
1063	13.85	14.88	69.83	69.83	sp|Q9CPP6|NDUA5_MOUSE	NADH dehydrogenase [ubiquinone] 1 alpha subcomplex subunit 5 OS=Mus musculus OX=10090 GN=Ndufa5 PE=1 SV=3	MOUSE	8
1064	13.85	14.43	35.71	29.29	tr|Q5KU03|Q5KU03_MOUSE	Glycogen synthase kinase-3 beta OS=Mus musculus OX=10090 GN=Gsk3b PE=1 SV=1	MOUSE	8
1065	13.83	14.14	22.22	12.90	sp|Q9DBC3|CMTR1_MOUSE	Cap-specific mRNA (nucleoside-2'-O-)-methyltransferase 1 OS=Mus musculus OX=10090 GN=Cmtr1 PE=1 SV=1	MOUSE	9
1066	13.82	14.22	42.95	38.26	tr|Q91YK6|Q91YK6_MOUSE	Rpl23a protein (Fragment) OS=Mus musculus OX=10090 GN=Rpl23a PE=2 SV=1	MOUSE	8
1067	13.82	13.93	52.40	44.73	tr|A0A0R4J094|A0A0R4J094_MOUSE	Fumarylacetoacetate hydrolase domain-containing 2A OS=Mus musculus OX=10090 GN=Fahd2a PE=1 SV=1	MOUSE	7
1068	13.77	13.98	19.81	11.74	sp|Q9EQQ9-3|OGA_MOUSE	Isoform 3 of Protein O-GlcNAcase OS=Mus musculus OX=10090 GN=Oga	MOUSE	9
1069	13.77	13.8	53.59	53.59	sp|Q8VBV7|CSN8_MOUSE	COP9 signalosome complex subunit 8 OS=Mus musculus OX=10090 GN=Cops8 PE=1 SV=1	MOUSE	7

1070	13.76	13.85	51.28	35.38	tr|Q3TXU8|Q3TXU8_MOUSE	Cytochrome b5 heme-binding domain-containing protein OS=Mus musculus OX=10090 GN=Pgrmc1 PE=1 SV=1	MOUSE	7
1071	13.73	13.89	31.96	16.96	sp|Q91X72|HEMO_MOUSE	Hemopexin OS=Mus musculus OX=10090 GN=Hpx PE=1 SV=2	MOUSE	8
1072	13.73	13.75	73.48	65.91	tr|Q6ZWZ6|Q6ZWZ6_MOUSE	40S ribosomal protein S12 OS=Mus musculus OX=10090 GN=Rps12 PE=1 SV=1	MOUSE	8
1073	13.69	13.74	39.89	35.11	tr|Q642K1|Q642K1_MOUSE	Ribosomal protein L18 OS=Mus musculus OX=10090 GN=Rpl18 PE=2 SV=1	MOUSE	8
1074	13.67	13.78	50.77	39.03	sp|Q8BH69|SPS1_MOUSE	Selenide, water dikinase 1 OS=Mus musculus OX=10090 GN=Sephs1 PE=1 SV=1	MOUSE	8
1075	13.65	13.71	29.91	20.24	tr|Q3ULL5|Q3ULL5_MOUSE	Eif2s2 protein OS=Mus musculus OX=10090 GN=Eif2s2 PE=1 SV=1	MOUSE	7
1076	13.62	13.64	35.80	35.50	tr|Q3TD71|Q3TD71_MOUSE	Secretory carrier-associated membrane protein OS=Mus musculus OX=10090 GN=Scamp1 PE=1 SV=1	MOUSE	8
1077	13.61	26.01	25.30	21.58	sp|Q91WM1|STRBP_MOUSE	Spermatid perinuclear RNA-binding protein OS=Mus musculus OX=10090 GN=Strbp PE=1 SV=1	MOUSE	14
1078	13.61	21.82	60.39	56.51	sp|Q9JKB3|YBOX3_MOUSE	Y-box-binding protein 3 OS=Mus musculus OX=10090 GN=Ybx3 PE=1 SV=2	MOUSE	12
1079	13.59	13.89	19.44	9.13	sp|Q6NV83|SR140_MOUSE	U2 snRNP-associated SURP motif-containing protein OS=Mus musculus OX=10090 GN=U2surp PE=1 SV=3	MOUSE	7
1080	13.59	13.64	52.57	34.14	sp|P47199|QOR_MOUSE	Quinone oxidoreductase OS=Mus musculus OX=10090 GN=Cryz PE=1 SV=1	MOUSE	7
1081	13.54	13.62	15.17	9.86	tr|Q8CHG1|Q8CHG1_MOUSE	MKIAA0369 protein (Fragment) OS=Mus musculus OX=10090 GN=Dclk1 PE=2 SV=1	MOUSE	7
1082	13.52	15.7	41.53	37.21	sp|Q9DCT1|AKCL2_MOUSE	1,5-anhydro-D-fructose reductase OS=Mus musculus OX=10090 GN=Akr1e2 PE=1 SV=1	MOUSE	9
1083	13.51	13.56	45.89	41.13	sp|P27048|RSMB_MOUSE	Small nuclear ribonucleoprotein-associated protein B OS=Mus musculus OX=10090 GN=Snrpb PE=1 SV=1	MOUSE	12
1084	13.5	13.67	57.34	37.12	sp|Q9QYA2|TOM40_MOUSE	Mitochondrial import receptor subunit TOM40 homolog OS=Mus musculus OX=10090 GN=Tomm40 PE=1 SV=3	MOUSE	8
1085	13.5	13.66	29.70	15.25	tr|Q3UKF5|Q3UKF5_MOUSE	X-prolyl aminopeptidase OS=Mus musculus OX=10090 GN=Xpnpep1 PE=2 SV=1	MOUSE	7

1086	13.49	13.65	59.77	54.60	tr|A0A087WRH2|A0A087WRH2_MOUSE	Fas apoptotic inhibitory molecule 1 (Fragment) OS=Mus musculus OX=10090 GN=Faim PE=1 SV=6	MOUSE	8
1087	13.49	13.55	18.04	13.70	tr|Q69ZI4|Q69ZI4_MOUSE	MKIAA1480 protein (Fragment) OS=Mus musculus OX=10090 GN=mKIAA1480 PE=2 SV=1	MOUSE	8
1088	13.48	13.65	11.58	4.54	tr|A0A0N4SVL0|A0A0N4SVL0_MOUSE	Eukaryotic translation initiation factor 4 gamma 3 OS=Mus musculus OX=10090 GN=Eif4g3 PE=1 SV=2	MOUSE	7
1089	13.44	13.65	18.04	15.24	tr|Q3V165|Q3V165_MOUSE	Bardet-Biedl syndrome 7 protein homolog OS=Mus musculus OX=10090 GN=Bbs7 PE=2 SV=1	MOUSE	9
1090	13.43	13.65	52.79	39.02	tr|A0MNP4|A0MNP4_MOUSE	CDW13/WDR61 OS=Mus musculus OX=10090 GN=Wdr61 PE=1 SV=1	MOUSE	8
1091	13.42	13.64	24.87	13.45	tr|Q3UZH3|Q3UZH3_MOUSE	C-1-tetrahydrofolate synthase, cytoplasmic (Fragment) OS=Mus musculus OX=10090 GN=Mthfd1 PE=2 SV=1	MOUSE	8
1092	13.42	13.46	61.74	61.74	tr|Q58DZ3|Q58DZ3_MOUSE	60S ribosomal protein L30 OS=Mus musculus OX=10090 GN=Rpl30 PE=1 SV=1	MOUSE	7
1093	13.39	13.64	38.46	33.78	tr|Q3TDN8|Q3TDN8_MOUSE	AB hydrolase-1 domain-containing protein OS=Mus musculus OX=10090 GN=Bphl PE=2 SV=1	MOUSE	7
1094	13.38	13.44	62.31	56.15	tr|Q5M9M4|Q5M9M4_MOUSE	40S ribosomal protein S15a OS=Mus musculus OX=10090 GN=Rps15a PE=1 SV=1	MOUSE	7
1095	13.34	14.31	47.84	40.29	tr|Q544Q7|Q544Q7_MOUSE	Sodium/potassium-transporting ATPase subunit beta OS=Mus musculus OX=10090 GN=Atp1b3 PE=1 SV=1	MOUSE	9
1096	13.31	13.51	13.49	9.61	tr|Q8C8Y2|Q8C8Y2_MOUSE	WD repeat domain 17 OS=Mus musculus OX=10090 GN=Wdr17 PE=1 SV=1	MOUSE	9
1097	13.26	13.3	33.41	22.99	tr|Q8C1W9|Q8C1W9_MOUSE	Uncharacterized protein OS=Mus musculus OX=10090 GN=Nap1l4 PE=2 SV=1	MOUSE	7
1098	13.22	13.26	20.32	19.07	tr|A2A513|A2A513_MOUSE	Keratin, type I cytoskeletal 10 OS=Mus musculus OX=10090 GN=Krt10 PE=1 SV=1	MOUSE	9
1099	13.21	17.74	32.34	20.77	tr|Q570Z8|Q570Z8_MOUSE	MKIAA4114 protein (Fragment) OS=Mus musculus OX=10090 GN=Picalm PE=2 SV=1	MOUSE	12
1100	13.2	13.46	44.41	36.42	tr|B2RXQ8|B2RXQ8_MOUSE	WD repeat domain containing 82 OS=Mus musculus OX=10090 GN=Wdr82 PE=1 SV=1	MOUSE	7
1101	13.19	13.28	46.58	46.15	tr|Q8C266|Q8C266_MOUSE	Ras-related protein Rab-5C OS=Mus musculus OX=10090 GN=Rab5c PE=1 SV=1	MOUSE	9

1102	13.18	13.22	41.22	33.11	sp|P14115|RL27A_MOUSE	60S ribosomal protein L27a OS=Mus musculus OX=10090 GN=Rpl27a PE=1 SV=5	MOUSE	7
1103	13.17	15.52	40.18	22.00	tr|Q3U6B1|Q3U6B1_MOUSE	Uncharacterized protein (Fragment) OS=Mus musculus OX=10090 GN=G3bp2 PE=2 SV=1	MOUSE	10
1104	13.17	13.32	46.39	22.89	sp|O35685|NUDC_MOUSE	Nuclear migration protein nudC OS=Mus musculus OX=10090 GN=Nudc PE=1 SV=1	MOUSE	8
1105	13.15	13.2	26.09	24.45	tr|G3UYZ1|G3UYZ1_MOUSE	Immunoglobulin superfamily member 8 OS=Mus musculus OX=10090 GN=Igsf8 PE=1 SV=1	MOUSE	8
1106	13.13	15.23	67.57	54.05	sp|P83917|CBX1_MOUSE	Chromobox protein homolog 1 OS=Mus musculus OX=10090 GN=Cbx1 PE=1 SV=1	MOUSE	8
1107	13.1	13.21	47.92	21.73	sp|Q8C0E2|VP26B_MOUSE	Vacuolar protein sorting-associated protein 26B OS=Mus musculus OX=10090 GN=Vps26b PE=1 SV=1	MOUSE	7
1108	13.08	18.8	6.91	3.99	sp|Q9EPN1|NBEA_MOUSE	Neurobeachin OS=Mus musculus OX=10090 GN=Nbea PE=1 SV=1	MOUSE	10
1109	13.02	13.07	38.78	34.98	sp|Q9DCT2|NDUS3_MOUSE	NADH dehydrogenase [ubiquinone] iron-sulfur protein 3, mitochondrial OS=Mus musculus OX=10090 GN=Ndufs3 PE=1 SV=2	MOUSE	8
1110	13	13.1	30.40	20.10	sp|Q99LR1|ABD12_MOUSE	Lysophosphatidylserine lipase ABHD12 OS=Mus musculus OX=10090 GN=Abhd12 PE=1 SV=2	MOUSE	6
1111	13	13.1	25.66	19.96	tr|F6WDS8|F6WDS8_MOUSE	Regulator of G-protein-signaling 6 OS=Mus musculus OX=10090 GN=Rgs6 PE=1 SV=1	MOUSE	7
1112	13	13.07	47.44	41.45	sp|P49722|PSA2_MOUSE	Proteasome subunit alpha type-2 OS=Mus musculus OX=10090 GN=Psma2 PE=1 SV=3	MOUSE	7
1113	12.99	28.57	49.67	49.22	sp|P70333|HNRH2_MOUSE	Heterogeneous nuclear ribonucleoprotein H2 OS=Mus musculus OX=10090 GN=Hnrnph2 PE=1 SV=1	MOUSE	19
1114	12.99	13.04	22.54	17.46	tr|Q3TE95|Q3TE95_MOUSE	Uncharacterized protein OS=Mus musculus OX=10090 GN=Rcn2 PE=2 SV=1	MOUSE	7
1115	12.95	12.97	32.35	32.35	tr|Q91V55|Q91V55_MOUSE	40S ribosomal protein S5 OS=Mus musculus OX=10090 GN=Rps5 PE=1 SV=1	MOUSE	7
1116	12.93	13.1	43.62	26.62	sp|Q3U5Q7|CMPK2_MOUSE	UMP-CMP kinase 2, mitochondrial OS=Mus musculus OX=10090 GN=Cmpk2 PE=1 SV=2	MOUSE	7
1117	12.89	13.25	23.20	10.38	tr|D3Z0M9|D3Z0M9_MOUSE	RNA helicase OS=Mus musculus OX=10090 GN=Ddx23 PE=1 SV=1	MOUSE	7

1118	12.88	13.05	45.49	35.69	tr|Q9DCD8|Q9DCD8_MOUSE	Proteasome subunit alpha type OS=Mus musculus OX=10090 GN=Psma3 PE=2 SV=1	MOUSE	9
1119	12.86	12.95	72.17	65.22	tr|Q545F0|Q545F0_MOUSE	Macrophage migration inhibitory factor OS=Mus musculus OX=10090 GN=Mif PE=1 SV=1	MOUSE	11
1120	12.82	12.98	29.47	23.38	tr|Q3TM67|Q3TM67_MOUSE	PUA domain-containing protein OS=Mus musculus OX=10090 GN=Dkc1 PE=2 SV=1	MOUSE	7
1121	12.82	12.98	91.53	58.47	sp|P62869|ELOB_MOUSE	Elongin-B OS=Mus musculus OX=10090 GN=Elob PE=1 SV=1	MOUSE	7
1122	12.82	12.97	20.94	14.37	tr|Q571E2|Q571E2_MOUSE	MFLJ00383 protein (Fragment) OS=Mus musculus OX=10090 GN=Atp6ap1 PE=2 SV=1	MOUSE	6
1123	12.81	12.91	73.39	67.74	sp|Q9DAK9|PHP14_MOUSE	14 kDa phosphohistidine phosphatase OS=Mus musculus OX=10090 GN=Phpt1 PE=1 SV=1	MOUSE	9
1124	12.81	12.88	21.32	20.81	tr|Q78P93|Q78P93_MOUSE	Ceramidase OS=Mus musculus OX=10090 GN=Asah1 PE=1 SV=1	MOUSE	7
1125	12.8	12.87	52.43	52.43	tr|B1ARA3|B1ARA3_MOUSE	60S ribosomal protein L26 (Fragment) OS=Mus musculus OX=10090 GN=Rpl26 PE=1 SV=1	MOUSE	7
1126	12.79	12.91	14.64	8.47	tr|G3X9J4|G3X9J4_MOUSE	RAB11-binding protein RELCH OS=Mus musculus OX=10090 GN=Relch PE=1 SV=2	MOUSE	7
1127	12.79	12.88	15.96	11.85	sp|P28571-1|SC6A9_MOUSE	Isoform GlyT-1A of Sodium- and chloride-dependent glycine transporter 1 OS=Mus musculus OX=10090 GN=Slc6a9	MOUSE	7
1128	12.76	12.96	27.32	11.60	sp|Q9WTX6|CUL1_MOUSE	Cullin-1 OS=Mus musculus OX=10090 GN=Cul1 PE=1 SV=1	MOUSE	6
1129	12.7	14.75	41.85	39.08	sp|Q9D0M3|CY1_MOUSE	Cytochrome c1, heme protein, mitochondrial OS=Mus musculus OX=10090 GN=Cyc1 PE=1 SV=1	MOUSE	9
1130	12.68	12.82	45.42	25.73	sp|Q922V4|PLRG1_MOUSE	Pleiotropic regulator 1 OS=Mus musculus OX=10090 GN=Plrg1 PE=1 SV=1	MOUSE	7
1131	12.67	12.84	42.47	27.42	sp|Q3TDN2|FAF2_MOUSE	FAS-associated factor 2 OS=Mus musculus OX=10090 GN=Faf2 PE=1 SV=2	MOUSE	7
1132	12.66	12.84	41.83	31.30	sp|P42208|SEPT2_MOUSE	Septin-2 OS=Mus musculus OX=10090 GN=Septin2 PE=1 SV=2	MOUSE	9
1133	12.65	15.07	34.15	21.95	sp|Q6PDL0|DC1L2_MOUSE	Cytoplasmic dynein 1 light intermediate chain 2 OS=Mus musculus OX=10090 GN=Dync1li2 PE=1 SV=2	MOUSE	8

1134	12.64	15.68	69.02	57.07	sp|P62835|RAP1A_MOUSE	Ras-related protein Rap-1A OS=Mus musculus OX=10090 GN=Rap1a PE=1 SV=1	MOUSE	9
1135	12.64	12.76	37.38	34.58	tr|A1L3B8|A1L3B8_MOUSE	Proteasome (Prosome, macropain) 26S subunit, non-ATPase, 7 OS=Mus musculus OX=10090 GN=Psmd7 PE=1 SV=1	MOUSE	9
1136	12.64	12.73	52.02	39.52	tr|Q564E5|Q564E5_MOUSE	Eukaryotic translation initiation factor 4H OS=Mus musculus OX=10090 GN=Eif4h PE=2 SV=1	MOUSE	7
1137	12.64	12.72	55.88	47.06	tr|Q5BLJ9|Q5BLJ9_MOUSE	60S ribosomal protein L27 OS=Mus musculus OX=10090 GN=Rpl27 PE=1 SV=1	MOUSE	9
1138	12.59	12.7	44.00	44.00	sp|O70251|EF1B_MOUSE	Elongation factor 1-beta OS=Mus musculus OX=10090 GN=Eef1b PE=1 SV=5	MOUSE	9
1139	12.59	12.67	52.53	49.42	sp|Q9CQ60|6PGL_MOUSE	6-phosphogluconolactonase OS=Mus musculus OX=10090 GN=Pgls PE=1 SV=1	MOUSE	8
1140	12.58	12.69	42.37	28.81	sp|Q9D710|TMX2_MOUSE	Thioredoxin-related transmembrane protein 2 OS=Mus musculus OX=10090 GN=Tmx2 PE=1 SV=1	MOUSE	8
1141	12.57	12.64	51.70	43.54	tr|Q569Y6|Q569Y6_MOUSE	Ubiquitin-conjugating enzyme E2 variant 1 OS=Mus musculus OX=10090 GN=Ube2v1 PE=2 SV=1	MOUSE	7
1142	12.55	28.85	39.42	21.84	tr|Q4KML7|Q4KML7_MOUSE	Ezrin OS=Mus musculus OX=10090 GN=Ezr PE=1 SV=1	MOUSE	15
1143	12.54	13.03	41.29	35.32	tr|I7HLV2|I7HLV2_MOUSE	60S ribosomal protein L10 (Fragment) OS=Mus musculus OX=10090 GN=Rpl10 PE=1 SV=1	MOUSE	7
1144	12.54	12.62	20.96	16.77	sp|Q8BKZ9|ODPX_MOUSE	Pyruvate dehydrogenase protein X component, mitochondrial OS=Mus musculus OX=10090 GN=Pdhx PE=1 SV=1	MOUSE	6
1145	12.51	12.58	56.52	38.04	sp|Q9CRC9|GNPI2_MOUSE	Glucosamine-6-phosphate isomerase 2 OS=Mus musculus OX=10090 GN=Gnpda2 PE=1 SV=1	MOUSE	8
1146	12.49	12.62	31.14	20.25	tr|Q99J57|Q99J57_MOUSE	S-adenosylmethionine synthase OS=Mus musculus OX=10090 GN=Mat2a PE=1 SV=1	MOUSE	7
1147	12.46	12.49	28.09	16.50	tr|Q571J7|Q571J7_MOUSE	Serine/threonine-protein phosphatase 2A 55 kDa regulatory subunit B (Fragment) OS=Mus musculus OX=10090 GN=Ppp2r2a PE=2 SV=1	MOUSE	6
1148	12.45	12.62	34.56	30.39	sp|Q8BLF1|NCEH1_MOUSE	Neutral cholesterol ester hydrolase 1 OS=Mus musculus OX=10090 GN=Nceh1 PE=1 SV=1	MOUSE	8

1149	12.45	12.47	25.00	16.27	sp|Q9JHI5|IVD_MOUSE	Isovaleryl-CoA dehydrogenase, mitochondrial OS=Mus musculus OX=10090 GN=Ivd PE=1 SV=1	MOUSE	6
1150	12.43	12.57	25.30	14.26	tr|Q91YS2|Q91YS2_MOUSE	Rangap1 protein OS=Mus musculus OX=10090 GN=Rangap1 PE=2 SV=1	MOUSE	7
1151	12.41	12.44	29.98	14.72	tr|Q8C2T9|Q8C2T9_MOUSE	Uncharacterized protein OS=Mus musculus OX=10090 GN=Ik PE=2 SV=1	MOUSE	7
1152	12.39	12.53	24.63	24.04	sp|Q9WVT6|CAH14_MOUSE	Carbonic anhydrase 14 OS=Mus musculus OX=10090 GN=Ca14 PE=1 SV=1	MOUSE	7
1153	12.38	12.44	44.84	27.34	sp|Q9QZB7|ARP10_MOUSE	Actin-related protein 10 OS=Mus musculus OX=10090 GN=Actr10 PE=1 SV=2	MOUSE	6
1154	12.37	12.49	37.24	33.47	tr|G3UW94|G3UW94_MOUSE	Splicing factor U2AF 35 kDa subunit OS=Mus musculus OX=10090 GN=U2af1 PE=1 SV=1	MOUSE	10
1155	12.37	12.45	38.28	22.97	sp|Q8R2R9|AP3M2_MOUSE	AP-3 complex subunit mu-2 OS=Mus musculus OX=10090 GN=Ap3m2 PE=1 SV=1	MOUSE	6
1156	12.35	12.45	31.82	29.09	tr|Q9DD05|Q9DD05_MOUSE	Delta-aminolevulinic acid dehydratase OS=Mus musculus OX=10090 GN=Alad PE=2 SV=1	MOUSE	7
1157	12.33	12.44	13.63	11.00	tr|E9Q8K8|E9Q8K8_MOUSE	Zinc finger CCCH domain-containing protein 4 OS=Mus musculus OX=10090 GN=Zc3h4 PE=1 SV=1	MOUSE	8
1158	12.3	14.47	39.19	34.68	tr|Q0PD35|Q0PD35_MOUSE	Ras-related protein Rab-21 OS=Mus musculus OX=10090 GN=Rab21 PE=1 SV=1	MOUSE	8
1159	12.3	12.49	20.55	8.13	tr|A0A0A0MQD1|A0A0A0MQD1_MOUSE	Tudor domain-containing protein 7 OS=Mus musculus OX=10090 GN=Tdrd7 PE=1 SV=1	MOUSE	7
1160	12.29	12.48	16.74	10.40	sp|P98203|ARVC_MOUSE	Armadillo repeat protein deleted in velo-cardio-facial syndrome homolog OS=Mus musculus OX=10090 GN=Arvcf PE=1 SV=2	MOUSE	8
1161	12.28	12.48	36.73	29.65	sp|Q91YJ3|THYN1_MOUSE	Thymocyte nuclear protein 1 OS=Mus musculus OX=10090 GN=Thyn1 PE=1 SV=1	MOUSE	7
1162	12.27	13.07	45.87	23.54	sp|Q3THG9|AASD1_MOUSE	Alanyl-tRNA editing protein Aarsd1 OS=Mus musculus OX=10090 GN=Aarsd1 PE=1 SV=2	MOUSE	7
1163	12.27	12.66	28.53	20.16	tr|Q3UZ17|Q3UZ17_MOUSE	Uncharacterized protein OS=Mus musculus OX=10090 GN=Luc7l2 PE=2 SV=1	MOUSE	6
1164	12.25	12.46	15.01	6.95	sp|Q9QY81|PO210_MOUSE	Nuclear pore membrane glycoprotein 210 OS=Mus musculus OX=10090 GN=Nup210 PE=1 SV=2	MOUSE	8

1165	12.25	12.3	34.26	29.76	tr|Q9DBN7|Q9DBN7_MOUSE	Uncharacterized protein OS=Mus musculus OX=10090 GN=Eci1 PE=2 SV=1	MOUSE	7
1166	12.24	12.25	59.66	42.05	sp|Q9DCS9|NDUBA_MOUSE	NADH dehydrogenase [ubiquinone] 1 beta subcomplex subunit 10 OS=Mus musculus OX=10090 GN=Ndufb10 PE=1 SV=3	MOUSE	8
1167	12.22	12.39	32.51	26.11	tr|Q5M9M0|Q5M9M0_MOUSE	60S ribosomal protein L13a OS=Mus musculus OX=10090 GN=Rpl13a PE=1 SV=1	MOUSE	7
1168	12.2	12.34	27.29	20.26	sp|A2ASZ8|SCMC2_MOUSE	Calcium-binding mitochondrial carrier protein SCaMC-2 OS=Mus musculus OX=10090 GN=Slc25a25 PE=1 SV=1	MOUSE	8
1169	12.19	12.53	22.73	13.08	tr|Q3TBF1|Q3TBF1_MOUSE	Protein-arginine deiminase OS=Mus musculus OX=10090 GN=Padi2 PE=2 SV=1	MOUSE	8
1170	12.19	12.29	36.52	29.78	sp|Q9JM76|ARPC3_MOUSE	Actin-related protein 2/3 complex subunit 3 OS=Mus musculus OX=10090 GN=Arpc3 PE=1 SV=3	MOUSE	7
1171	12.18	12.28	30.00	27.71	sp|Q91YR1|TWF1_MOUSE	Twinfilin-1 OS=Mus musculus OX=10090 GN=Twf1 PE=1 SV=2	MOUSE	7
1172	12.17	12.34	19.00	11.93	tr|A0A0R4J049|A0A0R4J049_MOUSE	Protein arginine N-methyltransferase 5 OS=Mus musculus OX=10090 GN=Prmt5 PE=1 SV=1	MOUSE	6
1173	12.14	13.22	10.72	5.46	tr|M0QWP1|M0QWP1_MOUSE	Agrin OS=Mus musculus OX=10090 GN=Agrn PE=1 SV=1	MOUSE	9
1174	12.14	12.15	30.67	23.47	tr|Q921W2|Q921W2_MOUSE	Nucleolysin TIAR OS=Mus musculus OX=10090 GN=Tial1 PE=1 SV=1	MOUSE	6
1175	12.08	12.19	36.21	23.96	sp|Q99J77|SIAS_MOUSE	Sialic acid synthase OS=Mus musculus OX=10090 GN=Nans PE=1 SV=1	MOUSE	8
1176	12.06	12.14	49.04	23.37	tr|Q544A1|Q544A1_MOUSE	WW domain binding protein 2 OS=Mus musculus OX=10090 GN=Wbp2 PE=1 SV=1	MOUSE	7
1177	12.02	13.06	44.93	44.93	tr|Q7JCZ1|Q7JCZ1_MOUSE	Cytochrome c oxidase subunit 2 OS=Mus musculus OX=10090 GN=mt-Co2 PE=1 SV=1	MOUSE	7
1178	12.01	20.44	41.18	33.88	sp|Q60973|RBBP7_MOUSE	Histone-binding protein RBBP7 OS=Mus musculus OX=10090 GN=Rbbp7 PE=1 SV=1	MOUSE	10
1179	12.01	12.15	37.14	26.00	sp|Q3UMR5|MCU_MOUSE	Calcium uniporter protein, mitochondrial OS=Mus musculus OX=10090 GN=Mcu PE=1 SV=2	MOUSE	7
1180	12.01	12.01	44.04	38.07	tr|Q3URG1|Q3URG1_MOUSE	Uncharacterized protein OS=Mus musculus OX=10090 GN=Tppp PE=1 SV=1	MOUSE	6

1181	12	12	61.73	61.73	tr|Q8C1L7|Q8C1L7_MOUSE	40S ribosomal protein S21 OS=Mus musculus OX=10090 PE=2 SV=1	MOUSE	7
1182	12	12	26.21	26.21	tr|Q9CXY0|Q9CXY0_MOUSE	Small monomeric GTPase OS=Mus musculus OX=10090 GN=Rala PE=2 SV=1	MOUSE	6
1183	12	12	54.55	54.55	tr|Q5BLK0|Q5BLK0_MOUSE	60S ribosomal protein L12 OS=Mus musculus OX=10090 GN=Rpl12 PE=1 SV=1	MOUSE	6
1184	12	12	31.75	31.75	sp|Q9DCC4|P5CR3_MOUSE	Pyrroline-5-carboxylate reductase 3 OS=Mus musculus OX=10090 GN=Pycr3 PE=1 SV=2	MOUSE	8
1185	11.99	12.44	42.78	36.26	sp|Q99JB2|STML2_MOUSE	Stomatin-like protein 2, mitochondrial OS=Mus musculus OX=10090 GN=Stoml2 PE=1 SV=1	MOUSE	7
1186	11.98	12.04	36.52	36.17	sp|Q8K4Z3|NNRE_MOUSE	NAD(P)H-hydrate epimerase OS=Mus musculus OX=10090 GN=Naxe PE=1 SV=1	MOUSE	7
1187	11.95	12.01	43.18	33.52	tr|Q3THJ0|Q3THJ0_MOUSE	60S ribosomal protein L18a OS=Mus musculus OX=10090 GN=Rpl18a PE=2 SV=1	MOUSE	6
1188	11.93	12.08	18.13	8.28	tr|Q3UF95|Q3UF95_MOUSE	BCL2-associated athanogene 6 OS=Mus musculus OX=10090 GN=Bag6 PE=1 SV=1	MOUSE	7
1189	11.92	12.13	32.62	21.51	tr|Q3UG16|Q3UG16_MOUSE	Uncharacterized protein OS=Mus musculus OX=10090 GN=Ap1m1 PE=1 SV=1	MOUSE	7
1190	11.92	12.09	22.24	14.02	tr|Q542C3|Q542C3_MOUSE	Uncharacterized protein OS=Mus musculus OX=10090 GN=Pcif1 PE=1 SV=1	MOUSE	7
1191	11.9	12.4	30.40	12.16	tr|Q3UN55|Q3UN55_MOUSE	Carn_acyltransf domain-containing protein OS=Mus musculus OX=10090 GN=Cpt2 PE=2 SV=1	MOUSE	7
1192	11.9	11.97	36.36	36.36	sp|Q6P069|SORCN_MOUSE	Sorcin OS=Mus musculus OX=10090 GN=Sri PE=1 SV=1	MOUSE	7
1193	11.88	12.1	13.69	7.13	tr|Q3U4M7|Q3U4M7_MOUSE	Tripeptidyl-peptidase 2 OS=Mus musculus OX=10090 GN=Tpp2 PE=2 SV=1	MOUSE	7
1194	11.88	12.04	21.64	18.04	sp|Q8CCF0|PRP31_MOUSE	U4/U6 small nuclear ribonucleoprotein Prp31 OS=Mus musculus OX=10090 GN=Prpf31 PE=1 SV=3	MOUSE	7
1195	11.84	11.94	41.48	40.61	sp|Q61206|PA1B2_MOUSE	Platelet-activating factor acetylhydrolase IB subunit alpha2 OS=Mus musculus OX=10090 GN=Pafah1b2 PE=1 SV=2	MOUSE	7
1196	11.83	11.89	43.22	38.19	tr|Q3UWU7|Q3UWU7_MOUSE	Uncharacterized protein OS=Mus musculus OX=10090 GN=Diras2 PE=1 SV=1	MOUSE	6

1197	11.81	13.02	48.97	37.46	tr|Q3UFX6|Q3UFX6_MOUSE	Hydrolase_4 domain-containing protein OS=Mus musculus OX=10090 GN=Mgll PE=2 SV=1	MOUSE	8
1198	11.8	11.96	32.09	25.86	sp|Q8CHP8|PGP_MOUSE	Glycerol-3-phosphate phosphatase OS=Mus musculus OX=10090 GN=Pgp PE=1 SV=1	MOUSE	7
1199	11.78	12.04	27.04	17.60	sp|Q8BMA6|SRP68_MOUSE	Signal recognition particle subunit SRP68 OS=Mus musculus OX=10090 GN=Srp68 PE=1 SV=2	MOUSE	8
1200	11.78	11.81	45.12	37.67	tr|N0E4C0|N0E4C0_MOUSE	Casein kinase II subunit beta OS=Mus musculus OX=10090 GN=Csnk2b PE=2 SV=1	MOUSE	6
1201	11.76	11.81	59.84	50.39	sp|Q9D023|MPC2_MOUSE	Mitochondrial pyruvate carrier 2 OS=Mus musculus OX=10090 GN=Mpc2 PE=1 SV=1	MOUSE	6
1202	11.73	11.82	60.71	60.71	tr|Q9D0J2|Q9D0J2_MOUSE	ATP synthase F1 subunit delta OS=Mus musculus OX=10090 GN=Atp5d PE=2 SV=1	MOUSE	13
1203	11.73	11.8	61.86	49.15	tr|Q14AF6|Q14AF6_MOUSE	Small nuclear ribonucleoprotein Sm D2 OS=Mus musculus OX=10090 GN=Snrpd2 PE=1 SV=1	MOUSE	8
1204	11.72	11.81	38.02	31.14	sp|Q99LB6|MAT2B_MOUSE	Methionine adenosyltransferase 2 subunit beta OS=Mus musculus OX=10090 GN=Mat2b PE=1 SV=1	MOUSE	7
1205	11.7	11.86	75.93	63.89	tr|Q5I0U7|Q5I0U7_MOUSE	Tubulin-specific chaperone A OS=Mus musculus OX=10090 GN=Tbca PE=1 SV=1	MOUSE	7
1206	11.69	14.87	46.00	29.43	tr|Q545V8|Q545V8_MOUSE	Protein kinase domain-containing protein OS=Mus musculus OX=10090 GN=Csnk2a2 PE=1 SV=1	MOUSE	10
1207	11.69	11.77	25.56	22.78	sp|Q80SY6|ADAL_MOUSE	Adenosine deaminase-like protein OS=Mus musculus OX=10090 GN=Adal PE=2 SV=1	MOUSE	6
1208	11.67	14.41	32.97	21.98	tr|Q922I7|Q922I7_MOUSE	Polypyrimidine tract-binding protein 1 OS=Mus musculus OX=10090 GN=Ptbp1 PE=1 SV=1	MOUSE	10
1209	11.67	11.72	31.28	28.19	tr|Q9CWK0|Q9CWK0_MOUSE	60S ribosomal protein L14 OS=Mus musculus OX=10090 GN=Rpl14 PE=2 SV=1	MOUSE	7
1210	11.66	11.85	23.93	10.62	sp|Q3UNH4|GRIN1_MOUSE	G protein-regulated inducer of neurite outgrowth 1 OS=Mus musculus OX=10090 GN=Gprin1 PE=1 SV=2	MOUSE	7
1211	11.66	11.75	37.62	27.33	tr|Q6GTX3|Q6GTX3_MOUSE	Apoe protein OS=Mus musculus OX=10090 GN=Apoe PE=2 SV=1	MOUSE	7
1212	11.65	11.75	31.54	14.23	sp|Q99K28|ARFG2_MOUSE	ADP-ribosylation factor GTPase-activating protein 2 OS=Mus musculus OX=10090 GN=Arfgap2 PE=1 SV=1	MOUSE	5

1213	11.65	11.7	11.91	7.78	tr|A0A0R4J0D3|A0A0R4J0D3_MOUSE	Dolichyl-diphosphooligosaccharide--protein glycotransferase OS=Mus musculus OX=10090 GN=Stt3b PE=1 SV=1	MOUSE	6
1214	11.64	11.66	40.80	40.30	sp|Q8VBV8|GUC1B_MOUSE	Guanylyl cyclase-activating protein 2 OS=Mus musculus OX=10090 GN=Guca1b PE=1 SV=3	MOUSE	6
1215	11.63	11.84	7.26	4.99	tr|E9QAI5|E9QAI5_MOUSE	Aspartate carbamoyltransferase OS=Mus musculus OX=10090 GN=Cad PE=1 SV=1	MOUSE	8
1216	11.63	11.75	51.82	42.34	sp|Q9ER00|STX12_MOUSE	Syntaxin-12 OS=Mus musculus OX=10090 GN=Stx12 PE=1 SV=1	MOUSE	7
1217	11.63	11.75	30.03	23.21	sp|Q8BGF6|ELMD2_MOUSE	ELMO domain-containing protein 2 OS=Mus musculus OX=10090 GN=Elmod2 PE=1 SV=1	MOUSE	8
1218	11.62	11.82	25.82	22.35	sp|Q9CPY7|AMPL_MOUSE	Cytosol aminopeptidase OS=Mus musculus OX=10090 GN=Lap3 PE=1 SV=3	MOUSE	8
1219	11.6	11.64	33.04	32.60	sp|Q7TNE3|SPAG7_MOUSE	Sperm-associated antigen 7 OS=Mus musculus OX=10090 GN=Spag7 PE=1 SV=1	MOUSE	6
1220	11.59	11.86	23.36	17.03	tr|Q3UQN3|Q3UQN3_MOUSE	UV excision repair protein RAD23 OS=Mus musculus OX=10090 GN=Rad23b PE=2 SV=1	MOUSE	6
1221	11.57	11.62	41.60	41.60	tr|Q9CY93|Q9CY93_MOUSE	60S ribosomal protein L31 OS=Mus musculus OX=10090 GN=Rpl31 PE=2 SV=1	MOUSE	6
1222	11.56	11.74	38.75	31.83	tr|Q9D375|Q9D375_MOUSE	AB hydrolase-1 domain-containing protein OS=Mus musculus OX=10090 GN=Abhd6 PE=2 SV=1	MOUSE	7
1223	11.55	11.6	64.04	64.04	sp|Q9D0M5|DYL2_MOUSE	Dynein light chain 2, cytoplasmic OS=Mus musculus OX=10090 GN=Dynll2 PE=1 SV=1	MOUSE	8
1224	11.54	13.74	47.93	45.16	sp|Q80UU9|PGRC2_MOUSE	Membrane-associated progesterone receptor component 2 OS=Mus musculus OX=10090 GN=Pgrmc2 PE=1 SV=2	MOUSE	7
1225	11.53	11.63	31.55	28.71	tr|Q8R5C0|Q8R5C0_MOUSE	Metaxin OS=Mus musculus OX=10090 GN=Mtx1 PE=1 SV=1	MOUSE	7
1226	11.52	11.73	32.79	28.37	tr|K4DI58|K4DI58_MOUSE	Cell adhesion molecule 3 OS=Mus musculus OX=10090 GN=Cadm3 PE=1 SV=1	MOUSE	6
1227	11.5	11.55	16.04	14.06	sp|Q9DCP2|S38A3_MOUSE	Sodium-coupled neutral amino acid transporter 3 OS=Mus musculus OX=10090 GN=Slc38a3 PE=1 SV=1	MOUSE	7
1228	11.49	11.62	58.33	41.67	sp|Q9ERS2|NDUAD_MOUSE	NADH dehydrogenase [ubiquinone] 1 alpha subcomplex subunit 13 OS=Mus musculus OX=10090 GN=Ndufa13 PE=1 SV=3	MOUSE	6

1229	11.47	11.54	43.78	31.84	sp|Q9R1P3|PSB2_MOUSE	Proteasome subunit beta type-2 OS=Mus musculus OX=10090 GN=Psmb2 PE=1 SV=1	MOUSE	9
1230	11.46	11.56	50.78	39.90	sp|P84075|HPCA_MOUSE	Neuron-specific calcium-binding protein hippocalcin OS=Mus musculus OX=10090 GN=Hpca PE=1 SV=2	MOUSE	7
1231	11.46	11.52	72.09	53.49	sp|P56391|CX6B1_MOUSE	Cytochrome c oxidase subunit 6B1 OS=Mus musculus OX=10090 GN=Cox6b1 PE=1 SV=2	MOUSE	7
1232	11.45	11.52	29.82	29.82	sp|Q7TNV1|TLC3B_MOUSE	Ceramide synthase OS=Mus musculus OX=10090 GN=Tlcd3b PE=1 SV=2	MOUSE	7
1233	11.44	11.58	44.53	26.04	sp|Q8BWM0|PGES2_MOUSE	Prostaglandin E synthase 2 OS=Mus musculus OX=10090 GN=Ptges2 PE=1 SV=3	MOUSE	6
1234	11.41	11.56	36.07	23.93	sp|Q9DB73|NB5R1_MOUSE	NADH-cytochrome b5 reductase 1 OS=Mus musculus OX=10090 GN=Cyb5r1 PE=1 SV=1	MOUSE	6
1235	11.4	11.51	14.40	11.46	tr|Q3UGC0|Q3UGC0_MOUSE	RRM domain-containing protein OS=Mus musculus OX=10090 GN=Eif4b PE=2 SV=1	MOUSE	6
1236	11.4	11.48	65.05	55.34	sp|Q9CPQ8|ATP5L_MOUSE	ATP synthase subunit g, mitochondrial OS=Mus musculus OX=10090 GN=Atp5mg PE=1 SV=1	MOUSE	8
1237	11.38	11.5	39.40	28.53	sp|Q8C163|EXOG_MOUSE	Nuclease EXOG, mitochondrial OS=Mus musculus OX=10090 GN=Exog PE=1 SV=1	MOUSE	6
1238	11.37	11.54	21.09	11.29	sp|Q9Z315|SNUT1_MOUSE	U4/U6.U5 tri-snRNP-associated protein 1 OS=Mus musculus OX=10090 GN=Sart1 PE=1 SV=1	MOUSE	7
1239	11.37	11.43	35.92	31.39	sp|Q8WTY4|CPIN1_MOUSE	Anamorsin OS=Mus musculus OX=10090 GN=Ciapin1 PE=1 SV=1	MOUSE	7
1240	11.36	11.72	58.78	58.78	sp|Q9CQZ5|NDUA6_MOUSE	NADH dehydrogenase [ubiquinone] 1 alpha subcomplex subunit 6 OS=Mus musculus OX=10090 GN=Ndufa6 PE=1 SV=1	MOUSE	8
1241	11.33	11.62	20.65	7.22	sp|Q6ZPJ3|UBE2O_MOUSE	(E3-independent) E2 ubiquitin-conjugating enzyme UBE2O OS=Mus musculus OX=10090 GN=Ube2o PE=1 SV=3	MOUSE	6
1242	11.33	11.38	18.26	16.17	sp|Q80VP1|EPN1_MOUSE	Epsin-1 OS=Mus musculus OX=10090 GN=Epn1 PE=1 SV=3	MOUSE	6
1243	11.3	11.4	32.92	27.08	tr|Q9DCY6|Q9DCY6_MOUSE	Glutathione transferase OS=Mus musculus OX=10090 GN=Gstt1 PE=2 SV=1	MOUSE	6
1244	11.3	11.34	38.22	15.61	sp|Q62277|SYPH_MOUSE	Synaptophysin OS=Mus musculus OX=10090 GN=Syp PE=1 SV=2	MOUSE	6

1245	11.29	95.6	87.61	86.94	sp|P99024|TBB5_MOUSE	Tubulin beta-5 chain OS=Mus musculus OX=10090 GN=Tubb5 PE=1 SV=1	MOUSE	133
1246	11.28	11.36	37.22	28.16	sp|Q9CR26|VTA1_MOUSE	Vacuolar protein sorting-associated protein VTA1 homolog OS=Mus musculus OX=10090 GN=Vta1 PE=1 SV=1	MOUSE	6
1247	11.19	11.25	42.01	32.34	tr|Q9CZL0|Q9CZL0_MOUSE	GOLD domain-containing protein OS=Mus musculus OX=10090 GN=Tmed9 PE=2 SV=1	MOUSE	7
1248	11.18	13.42	54.48	37.31	tr|Q3U4H0|Q3U4H0_MOUSE	APC-binding protein EB1 OS=Mus musculus OX=10090 GN=Mapre1 PE=1 SV=1	MOUSE	8
1249	11.17	14.8	30.07	15.73	sp|Q61699|HS105_MOUSE	Heat shock protein 105 kDa OS=Mus musculus OX=10090 GN=Hsph1 PE=1 SV=2	MOUSE	9
1250	11.16	11.19	51.32	51.32	sp|Q9CPQ1|COX6C_MOUSE	Cytochrome c oxidase subunit 6C OS=Mus musculus OX=10090 GN=Cox6c PE=1 SV=3	MOUSE	8
1251	11.14	11.2	58.28	41.72	tr|Q642K0|Q642K0_MOUSE	Myl6 protein OS=Mus musculus OX=10090 GN=Myl6 PE=2 SV=1	MOUSE	5
1252	11.13	11.27	43.32	28.97	tr|Q5NTY0|Q5NTY0_MOUSE	DnaJ (Hsp40) homolog, subfamily A, member 1 OS=Mus musculus OX=10090 GN=Dnaja1 PE=1 SV=1	MOUSE	7
1253	11.13	11.22	33.81	30.58	tr|A2RTC8|A2RTC8_MOUSE	Membrane-associated ring finger (C3HC4) 5 OS=Mus musculus OX=10090 GN=Marchf5 PE=1 SV=1	MOUSE	6
1254	11.13	11.18	33.90	29.30	tr|A0A0R4J0X5|A0A0R4J0X5_MOUSE	Alpha-1-antitrypsin 1-3 OS=Mus musculus OX=10090 GN=Serpina1c PE=1 SV=1	MOUSE	7
1255	11.12	11.24	36.85	13.95	sp|Q5U458|DJC11_MOUSE	DnaJ homolog subfamily C member 11 OS=Mus musculus OX=10090 GN=Dnajc11 PE=1 SV=2	MOUSE	5
1256	11.12	11.22	38.05	31.99	sp|Q99J99|THTM_MOUSE	3-mercaptopyruvate sulfurtransferase OS=Mus musculus OX=10090 GN=Mpst PE=1 SV=4	MOUSE	6
1257	11.1	11.4	21.44	7.70	sp|Q8K212|PACS1_MOUSE	Phosphofurin acidic cluster sorting protein 1 OS=Mus musculus OX=10090 GN=Pacs1 PE=1 SV=2	MOUSE	6
1258	11.08	11.23	41.27	34.29	tr|Q544R7|Q544R7_MOUSE	Heme oxygenase OS=Mus musculus OX=10090 GN=Hmox2 PE=1 SV=1	MOUSE	7
1259	11.07	11.45	30.81	24.59	sp|Q8CG72|ADPRS_MOUSE	ADP-ribose glycohydrolase ARH3 OS=Mus musculus OX=10090 GN=Adprs PE=1 SV=1	MOUSE	6
1260	11.07	11.21	64.94	64.94	tr|Q561N4|Q561N4_MOUSE	Ubiquitin-conjugating enzyme E2L 3 OS=Mus musculus OX=10090 GN=Ube2l3 PE=1 SV=1	MOUSE	8

1261	11.07	11.19	31.15	21.90	sp|Q60749|KHDR1_MOUSE	KH domain-containing, RNA-binding, signal transduction-associated protein 1 OS=Mus musculus OX=10090 GN=Khdrbs1 PE=1 SV=2	MOUSE	7
1262	11.06	11.14	42.34	31.98	sp|P24472|GSTA4_MOUSE	Glutathione S-transferase A4 OS=Mus musculus OX=10090 GN=Gsta4 PE=1 SV=3	MOUSE	7
1263	11.06	11.13	81.61	70.11	tr|Q548W7|Q548W7_MOUSE	Diazepam binding inhibitor OS=Mus musculus OX=10090 GN=Dbi PE=1 SV=1	MOUSE	7
1264	11.04	11.18	32.42	21.16	sp|Q5M8N4|D39U1_MOUSE	Epimerase family protein SDR39U1 OS=Mus musculus OX=10090 GN=Sdr39u1 PE=1 SV=2	MOUSE	5
1265	11.04	11.14	33.02	22.12	tr|Q5HZI6|Q5HZI6_MOUSE	GDP-4-keto-6-deoxy-D-mannose-3,5-epimerase-4-reductase OS=Mus musculus OX=10090 GN=Gfus PE=1 SV=1	MOUSE	6
1266	11.03	12.16	19.52	8.47	tr|Q14BH8|Q14BH8_MOUSE	Cacna2d1 protein OS=Mus musculus OX=10090 GN=Cacna2d1 PE=2 SV=1	MOUSE	8
1267	11.03	11.07	65.49	63.72	tr|Q4V9T8|Q4V9T8_MOUSE	Eukaryotic translation initiation factor 1 OS=Mus musculus OX=10090 GN=Eif1 PE=1 SV=1	MOUSE	7
1268	11.02	11.14	32.84	27.46	tr|A0A0R4J1Y3|A0A0R4J1Y3_MOUSE	Probable tRNA N6-adenosine threonylcarbamoyltransferase OS=Mus musculus OX=10090 GN=Osgep PE=1 SV=1	MOUSE	6
1269	11.02	11.09	15.53	15.53	sp|Q9JKF6|NECT1_MOUSE	Nectin-1 OS=Mus musculus OX=10090 GN=Nectin1 PE=1 SV=3	MOUSE	7
1270	11	11.43	58.05	39.51	tr|Q545G0|Q545G0_MOUSE	Proteasome subunit beta OS=Mus musculus OX=10090 GN=Psmb3 PE=1 SV=1	MOUSE	6
1271	10.96	13.19	42.15	29.12	sp|P12658|CALB1_MOUSE	Calbindin OS=Mus musculus OX=10090 GN=Calb1 PE=1 SV=2	MOUSE	7
1272	10.95	11.07	23.37	9.60	tr|Q3TET2|Q3TET2_MOUSE	Glycerol kinase OS=Mus musculus OX=10090 GN=Gk PE=2 SV=1	MOUSE	5
1273	10.95	11	40.20	30.39	tr|Q5M8Q0|Q5M8Q0_MOUSE	Ribosomal protein L15 OS=Mus musculus OX=10090 GN=Rpl15 PE=2 SV=1	MOUSE	6
1274	10.93	11.08	44.49	35.24	sp|Q9CRB9|MIC19_MOUSE	MICOS complex subunit Mic19 OS=Mus musculus OX=10090 GN=Chchd3 PE=1 SV=1	MOUSE	6
1275	10.93	10.98	38.21	26.57	tr|Q4FJK0|Q4FJK0_MOUSE	Decr1 protein OS=Mus musculus OX=10090 GN=Decr1 PE=1 SV=1	MOUSE	6

1276	10.92	11.12	12.59	4.63	tr|A2AM56|A2AM56_MOUSE	ATP-binding cassette, sub-family A (ABC1), member 8b OS=Mus musculus OX=10090 GN=Abca8b PE=2 SV=1	MOUSE	6
1277	10.92	11.11	25.76	17.24	tr|A0A2I3BRL0|A0A2I3BRL0_MOUSE	5'-nucleotidase domain-containing 2 OS=Mus musculus OX=10090 GN=Nt5dc2 PE=1 SV=1	MOUSE	6
1278	10.92	11.02	21.80	19.89	tr|Q8CHR8|Q8CHR8_MOUSE	Developmentally regulated GTP binding protein 1 OS=Mus musculus OX=10090 GN=Drg1 PE=2 SV=1	MOUSE	7
1279	10.91	10.99	16.89	13.83	sp|Q6R891|NEB2_MOUSE	Neurabin-2 OS=Mus musculus OX=10090 GN=Ppp1r9b PE=1 SV=1	MOUSE	6
1280	10.9	11.02	22.58	18.55	tr|Q3UJC3|Q3UJC3_MOUSE	Glycylpeptide N-tetradecanoyltransferase OS=Mus musculus OX=10090 GN=Nmt1 PE=1 SV=1	MOUSE	8
1281	10.88	10.93	35.51	17.23	tr|Q8BSH9|Q8BSH9_MOUSE	Nucleosome assembly protein 1-like 1 OS=Mus musculus OX=10090 GN=Nap1l1 PE=1 SV=1	MOUSE	8
1282	10.85	11	22.00	17.16	tr|Q3UKQ3|Q3UKQ3_MOUSE	Uncharacterized protein OS=Mus musculus OX=10090 GN=Wbp11 PE=2 SV=1	MOUSE	6
1283	10.85	10.89	39.29	39.29	sp|P62830|RL23_MOUSE	60S ribosomal protein L23 OS=Mus musculus OX=10090 GN=Rpl23 PE=1 SV=1	MOUSE	6
1284	10.84	10.95	32.75	27.87	tr|A0A3B2W864|A0A3B2W864_MOUSE	Predicted gene, 49804 OS=Mus musculus OX=10090 GN=Gm49804 PE=3 SV=1	MOUSE	6
1285	10.82	10.97	17.40	7.65	sp|Q8C7X2-2|EMC1_MOUSE	Isoform 2 of ER membrane protein complex subunit 1 OS=Mus musculus OX=10090 GN=Emc1	MOUSE	5
1286	10.79	10.84	26.77	20.87	tr|Q8C671|Q8C671_MOUSE	Serine/arginine-rich splicing factor 2 OS=Mus musculus OX=10090 GN=Srsf2 PE=2 SV=1	MOUSE	6
1287	10.78	10.85	40.00	39.05	tr|Q6ZWZ4|Q6ZWZ4_MOUSE	60S ribosomal protein L36 OS=Mus musculus OX=10090 GN=Rpl36 PE=1 SV=1	MOUSE	7
1288	10.77	11.16	18.95	7.27	sp|Q9EPK7|XPO7_MOUSE	Exportin-7 OS=Mus musculus OX=10090 GN=Xpo7 PE=1 SV=3	MOUSE	6
1289	10.75	11.86	23.12	14.73	sp|Q8K3K8|OPTN_MOUSE	Optineurin OS=Mus musculus OX=10090 GN=Optn PE=1 SV=1	MOUSE	7
1290	10.75	11.3	25.49	13.39	tr|Q922A2|Q922A2_MOUSE	Annexin OS=Mus musculus OX=10090 GN=Anxa7 PE=2 SV=1	MOUSE	5
1291	10.75	10.95	45.73	18.48	tr|Q8BX12|Q8BX12_MOUSE	Uncharacterized protein (Fragment) OS=Mus musculus OX=10090 GN=Ncoa5 PE=2 SV=1	MOUSE	8
1292	10.74	15.2	30.87	18.67	tr|Q3TM70|Q3TM70_MOUSE	Uncharacterized protein OS=Mus musculus OX=10090 GN=Ehd4 PE=1 SV=1	MOUSE	8

1293	10.73	10.82	34.21	26.56	sp|Q3TMH2|SCRN3_MOUSE	Secernin-3 OS=Mus musculus OX=10090 GN=Scrn3 PE=1 SV=1	MOUSE	6
1294	10.72	10.91	29.58	21.73	sp|Q8VE88|F1142_MOUSE	Protein FAM114A2 OS=Mus musculus OX=10090 GN=Fam114a2 PE=1 SV=2	MOUSE	6
1295	10.71	17.5	36.01	23.55	sp|Q64332|SYN2_MOUSE	Synapsin-2 OS=Mus musculus OX=10090 GN=Syn2 PE=1 SV=2	MOUSE	11
1296	10.71	10.82	21.58	15.45	tr|Q3UYP2|Q3UYP2_MOUSE	Prenylcys_lyase domain-containing protein OS=Mus musculus OX=10090 GN=Pcyox1 PE=2 SV=1	MOUSE	6
1297	10.7	10.86	34.35	22.44	sp|Q9D5T0|ATAD1_MOUSE	Outer mitochondrial transmembrane helix translocase OS=Mus musculus OX=10090 GN=Atad1 PE=1 SV=1	MOUSE	7
1298	10.7	10.84	48.26	48.26	sp|Q3THE2|ML12B_MOUSE	Myosin regulatory light chain 12B OS=Mus musculus OX=10090 GN=Myl12b PE=1 SV=2	MOUSE	7
1299	10.69	11.01	14.48	7.84	tr|B2RQC7|B2RQC7_MOUSE	DIP2 disco-interacting protein 2 homolog B (Drosophila) OS=Mus musculus OX=10090 GN=Dip2b PE=1 SV=1	MOUSE	7
1300	10.65	16.16	39.71	29.43	sp|Q8BWG8|ARRB1_MOUSE	Beta-arrestin-1 OS=Mus musculus OX=10090 GN=Arrb1 PE=1 SV=1	MOUSE	9
1301	10.63	10.8	29.43	22.49	tr|Q3U944|Q3U944_MOUSE	L-type lectin-like domain-containing protein OS=Mus musculus OX=10090 GN=Lman1 PE=2 SV=1	MOUSE	9
1302	10.61	10.79	19.37	9.81	tr|Q3TWN8|Q3TWN8_MOUSE	Delta-1-pyrroline-5-carboxylate synthase OS=Mus musculus OX=10090 GN=Aldh18a1 PE=2 SV=1	MOUSE	6
1303	10.6	12.81	57.32	36.59	tr|Q9D6W4|Q9D6W4_MOUSE	RRM domain-containing protein OS=Mus musculus OX=10090 GN=Srsf3 PE=2 SV=1	MOUSE	12
1304	10.6	10.76	65.66	54.55	tr|Q99JZ4|Q99JZ4_MOUSE	GTP-binding protein SAR1a OS=Mus musculus OX=10090 GN=Sar1a PE=1 SV=1	MOUSE	6
1305	10.59	12.81	23.78	23.48	tr|A2AEG6|A2AEG6_MOUSE	Neuronal membrane glycoprotein M6-b OS=Mus musculus OX=10090 GN=Gpm6b PE=1 SV=1	MOUSE	10
1306	10.58	27.95	50.26	47.89	sp|Q63844|MK03_MOUSE	Mitogen-activated protein kinase 3 OS=Mus musculus OX=10090 GN=Mapk3 PE=1 SV=5	MOUSE	15
1307	10.55	10.66	72.48	63.76	sp|P0DP28|CALM3_MOUSE	Calmodulin-3 OS=Mus musculus OX=10090 GN=Calm3 PE=1 SV=1	MOUSE	10
1308	10.54	10.66	26.51	14.26	sp|Q9CZX0|ELP3_MOUSE	Elongator complex protein 3 OS=Mus musculus OX=10090 GN=Elp3 PE=1 SV=1	MOUSE	6
1309	10.52	10.73	16.71	10.14	sp|B2RY56|RBM25_MOUSE	RNA-binding protein 25 OS=Mus musculus OX=10090 GN=Rbm25 PE=1 SV=2	MOUSE	7

1310	10.51	11.95	13.31	2.61	sp|Q6ZQ08|CNOT1_MOUSE	CCR4-NOT transcription complex subunit 1 OS=Mus musculus OX=10090 GN=Cnot1 PE=1 SV=2	MOUSE	6
1311	10.49	10.78	10.04	4.70	sp|B1AZI6|THOC2_MOUSE	THO complex subunit 2 OS=Mus musculus OX=10090 GN=Thoc2 PE=1 SV=1	MOUSE	6
1312	10.48	15.56	13.88	4.52	tr|Q80TM2|Q80TM2_MOUSE	MKIAA1027 protein (Fragment) OS=Mus musculus OX=10090 GN=Tln1 PE=2 SV=4	MOUSE	7
1313	10.43	10.46	43.63	33.98	sp|Q3UGR5|HDHD2_MOUSE	Haloacid dehalogenase-like hydrolase domain-containing protein 2 OS=Mus musculus OX=10090 GN=Hdhd2 PE=1 SV=2	MOUSE	6
1314	10.42	10.88	17.51	6.29	sp|Q9Z277|BAZ1B_MOUSE	Tyrosine-protein kinase BAZ1B OS=Mus musculus OX=10090 GN=Baz1b PE=1 SV=2	MOUSE	7
1315	10.42	10.63	14.77	5.14	tr|B2RQQ7|B2RQQ7_MOUSE	Non-specific serine/threonine protein kinase OS=Mus musculus OX=10090 GN=Cdc42bpb PE=2 SV=1	MOUSE	5
1316	10.42	10.53	22.99	16.90	tr|Q6P6I8|Q6P6I8_MOUSE	Signal-regulatory protein alpha OS=Mus musculus OX=10090 GN=Sirpa PE=1 SV=1	MOUSE	6
1317	10.42	10.53	33.01	20.71	sp|Q9CYG7|TOM34_MOUSE	Mitochondrial import receptor subunit TOM34 OS=Mus musculus OX=10090 GN=Tomm34 PE=1 SV=1	MOUSE	5
1318	10.42	10.45	54.76	53.17	tr|Q91VM2|Q91VM2_MOUSE	Small nuclear ribonucleoprotein Sm D3 OS=Mus musculus OX=10090 GN=Snrpd3 PE=2 SV=1	MOUSE	5
1319	10.4	14.51	53.91	53.91	tr|B2RVP5|B2RVP5_MOUSE	Histone H2A OS=Mus musculus OX=10090 GN=H2az2 PE=2 SV=1	MOUSE	11
1320	10.39	10.49	44.62	31.54	sp|O70152|DPM1_MOUSE	Dolichol-phosphate mannosyltransferase subunit 1 OS=Mus musculus OX=10090 GN=Dpm1 PE=1 SV=1	MOUSE	7
1321	10.38	10.57	45.42	29.58	tr|Q8C845|Q8C845_MOUSE	EF-hand domain-containing protein D2 OS=Mus musculus OX=10090 GN=Efhd2 PE=1 SV=1	MOUSE	7
1322	10.37	10.41	34.76	18.45	sp|Q99JX4|EIF3M_MOUSE	Eukaryotic translation initiation factor 3 subunit M OS=Mus musculus OX=10090 GN=Eif3m PE=1 SV=1	MOUSE	5
1323	10.36	10.6	41.75	24.92	sp|Q8K274|KT3K_MOUSE	Ketosamine-3-kinase OS=Mus musculus OX=10090 GN=Fn3krp PE=1 SV=2	MOUSE	6
1324	10.35	10.81	9.92	2.94	sp|Q6P5D8|SMHD1_MOUSE	Structural maintenance of chromosomes flexible hinge domain-containing protein 1 OS=Mus musculus OX=10090 GN=Smchd1 PE=1 SV=2	MOUSE	5
1325	10.35	10.49	40.96	26.51	tr|Q3UMJ4|Q3UMJ4_MOUSE	General transcription factor IIF subunit 2 OS=Mus musculus OX=10090 GN=Gtf2f2 PE=1 SV=1	MOUSE	6

1326	10.33	10.49	12.27	6.05	tr|A0A3B2WCN9|A0A3B2WCN9_MOUSE	Tight junction protein ZO-2 OS=Mus musculus OX=10090 GN=Tjp2 PE=1 SV=1	MOUSE	6
1327	10.31	10.4	41.49	41.49	tr|E9Q7G1|E9Q7G1_MOUSE	Transmembrane p24-trafficking protein 7 OS=Mus musculus OX=10090 GN=Tmed7 PE=1 SV=1	MOUSE	7
1328	10.29	11.87	16.24	8.22	tr|D3Z061|D3Z061_MOUSE	E1 ubiquitin-activating enzyme OS=Mus musculus OX=10090 GN=Uba6 PE=1 SV=1	MOUSE	7
1329	10.27	11.42	43.38	25.87	sp|Q99KQ4|NAMPT_MOUSE	Nicotinamide phosphoribosyltransferase OS=Mus musculus OX=10090 GN=Nampt PE=1 SV=1	MOUSE	7
1330	10.25	10.55	20.29	7.99	tr|Q6ZQ47|Q6ZQ47_MOUSE	MKIAA0784 protein (Fragment) OS=Mus musculus OX=10090 GN=Adnp PE=2 SV=1	MOUSE	6
1331	10.24	10.41	21.41	11.52	tr|Q9DBS6|Q9DBS6_MOUSE	Phosphodiesterase OS=Mus musculus OX=10090 GN=Pde1b PE=2 SV=1	MOUSE	5
1332	10.24	10.26	71.14	59.73	sp|Q9JKC6|CEND_MOUSE	Cell cycle exit and neuronal differentiation protein 1 OS=Mus musculus OX=10090 GN=Cend1 PE=1 SV=1	MOUSE	5
1333	10.22	10.34	12.17	8.76	tr|A0A571BE69|A0A571BE69_MOUSE	Neuronal-specific septin-3 OS=Mus musculus OX=10090 GN=Septin3 PE=1 SV=1	MOUSE	9
1334	10.21	10.44	34.25	26.48	tr|A0A7N9VR94|A0A7N9VR94_MOUSE	AHNAK nucleoprotein 2 OS=Mus musculus OX=10090 GN=Ahnak2 PE=4 SV=1	MOUSE	5
1335	10.2	10.28	40.22	34.06	sp|Q9JHW2|NIT2_MOUSE	Omega-amidase NIT2 OS=Mus musculus OX=10090 GN=Nit2 PE=1 SV=1	MOUSE	6
1336	10.18	10.27	21.61	19.11	tr|Q8BTW2|Q8BTW2_MOUSE	Eukaryotic translation initiation factor 3 subunit F OS=Mus musculus OX=10090 GN=Eif3f PE=2 SV=1	MOUSE	6
1337	10.17	10.76	57.04	42.96	sp|Q9DC07|LNEBL_MOUSE	LIM zinc-binding domain-containing Nebulette OS=Mus musculus OX=10090 GN=Nebl PE=1 SV=1	MOUSE	8
1338	10.16	10.27	39.69	34.54	tr|Q80V08|Q80V08_MOUSE	60S ribosomal protein L17 (Fragment) OS=Mus musculus OX=10090 GN=Rpl17 PE=2 SV=1	MOUSE	7
1339	10.16	10.18	31.91	29.18	sp|Q8BGB7|ENOPH_MOUSE	Enolase-phosphatase E1 OS=Mus musculus OX=10090 GN=Enoph1 PE=1 SV=1	MOUSE	6
1340	10.15	10.22	39.54	39.54	tr|Q3TI40|Q3TI40_MOUSE	Uncharacterized protein OS=Mus musculus OX=10090 GN=Mtx2 PE=2 SV=1	MOUSE	6
1341	10.15	10.16	27.39	17.52	tr|Q3TN35|Q3TN35_MOUSE	Uncharacterized protein OS=Mus musculus OX=10090 GN=Sgta PE=2 SV=1	MOUSE	7

1342	10.14	10.27	36.13	36.13	tr|A3KMJ8|A3KMJ8_MOUSE	Vbp1 protein (Fragment) OS=Mus musculus OX=10090 GN=Vbp1 PE=2 SV=1	MOUSE	6
1343	10.13	14.38	52.76	45.23	sp|Q9WVA4|TAGL2_MOUSE	Transgelin-2 OS=Mus musculus OX=10090 GN=Tagln2 PE=1 SV=4	MOUSE	7
1344	10.13	10.15	36.36	34.34	sp|Q9DCZ4|MIC26_MOUSE	MICOS complex subunit Mic26 OS=Mus musculus OX=10090 GN=Apoo PE=1 SV=2	MOUSE	5
1345	10.11	29.88	58.51	58.24	sp|P61164|ACTZ_MOUSE	Alpha-centractin OS=Mus musculus OX=10090 GN=Actr1a PE=1 SV=1	MOUSE	18
1346	10.1	10.24	59.12	36.50	sp|P62965|RABP1_MOUSE	Cellular retinoic acid-binding protein 1 OS=Mus musculus OX=10090 GN=Crabp1 PE=1 SV=2	MOUSE	6
1347	10.09	10.18	29.43	22.34	sp|P70697|DCUP_MOUSE	Uroporphyrinogen decarboxylase OS=Mus musculus OX=10090 GN=Urod PE=1 SV=2	MOUSE	6
1348	10.09	10.1	37.66	29.87	sp|Q8BHC4|DCAKD_MOUSE	Dephospho-CoA kinase domain-containing protein OS=Mus musculus OX=10090 GN=Dcakd PE=1 SV=1	MOUSE	5
1349	10.08	10.28	20.06	16.17	sp|Q91VE0|S27A4_MOUSE	Long-chain fatty acid transport protein 4 OS=Mus musculus OX=10090 GN=Slc27a4 PE=1 SV=1	MOUSE	8
1350	10.07	10.19	50.53	46.84	tr|Q546K1|Q546K1_MOUSE	Cytoglobin OS=Mus musculus OX=10090 GN=Cygb PE=1 SV=1	MOUSE	6
1351	10.06	10.06	58.39	48.91	sp|Q9CR61|NDUB7_MOUSE	NADH dehydrogenase [ubiquinone] 1 beta subcomplex subunit 7 OS=Mus musculus OX=10090 GN=Ndufb7 PE=1 SV=3	MOUSE	6
1352	10.05	10.13	18.44	15.37	tr|Q3UMC8|Q3UMC8_MOUSE	Uncharacterized protein OS=Mus musculus OX=10090 GN=Stam PE=2 SV=1	MOUSE	7
1353	10.05	10.05	36.92	31.90	tr|Q8R5L1|Q8R5L1_MOUSE	Complement component 1 Q subcomponent-binding protein, mitochondrial OS=Mus musculus OX=10090 GN=C1qbp PE=1 SV=1	MOUSE	5
1354	10.04	10.04	4.30	3.20	sp|P70670|NACAM_MOUSE	Nascent polypeptide-associated complex subunit alpha, muscle-specific form OS=Mus musculus OX=10090 GN=Naca PE=1 SV=2	MOUSE	6
1355	10.04	10.04	41.43	35.71	tr|B1AQF4|B1AQF4_MOUSE	Dual specificity protein phosphatase OS=Mus musculus OX=10090 GN=Dusp3 PE=1 SV=1	MOUSE	6
1356	10.02	10.23	47.04	27.61	tr|Q3TG37|Q3TG37_MOUSE	Na(+)/H(+) exchange regulatory cofactor NHE-RF OS=Mus musculus OX=10090 GN=Slc9a3r1 PE=2 SV=1	MOUSE	6

1357	10.02	10.02	41.82	22.18	sp|Q9CZ04|CSN7A_MOUSE	COP9 signalosome complex subunit 7a OS=Mus musculus OX=10090 GN=Cops7a PE=1 SV=2	MOUSE	5
1358	10.01	10.26	41.36	33.33	tr|Q923F9|Q923F9_MOUSE	NADH dehydrogenase [ubiquinone] iron-sulfur protein 4, mitochondrial OS=Mus musculus OX=10090 GN=Ndufs4 PE=2 SV=1	MOUSE	6
1359	10.01	10.01	33.33	33.33	tr|Q53YX2|Q53YX2_MOUSE	Thy-1 antigen OS=Mus musculus OX=10090 GN=Thy1 PE=2 SV=1	MOUSE	5
1360	10	10.1	14.88	8.76	tr|A0A0R4J0S4|A0A0R4J0S4_MOUSE	Lethal(2) giant larvae protein homolog 1 OS=Mus musculus OX=10090 GN=Llgl1 PE=1 SV=1	MOUSE	6
1361	10	10.02	49.11	38.46	sp|Q9CZB0|C560_MOUSE	Succinate dehydrogenase cytochrome b560 subunit, mitochondrial OS=Mus musculus OX=10090 GN=Sdhc PE=1 SV=1	MOUSE	5
1362	10	10	36.84	36.18	sp|Q9CQ92|FIS1_MOUSE	Mitochondrial fission 1 protein OS=Mus musculus OX=10090 GN=Fis1 PE=1 SV=1	MOUSE	6
1363	10	10	32.84	32.84	sp|Q9JM62|REEP6_MOUSE	Receptor expression-enhancing protein 6 OS=Mus musculus OX=10090 GN=Reep6 PE=1 SV=1	MOUSE	5
1364	10	10	22.54	22.54	sp|P51491|OPSB_MOUSE	Short-wave-sensitive opsin 1 OS=Mus musculus OX=10090 GN=Opn1sw PE=1 SV=1	MOUSE	6
1365	9.98	11.16	14.24	9.10	tr|C7G3P2|C7G3P2_MOUSE	PRP4 pre-mRNA-processing factor 4 homolog (Fragment) OS=Mus musculus OX=10090 GN=Prpf4b PE=2 SV=1	MOUSE	8
1366	9.97	10.18	17.96	10.21	tr|A0A0G2JEP0|A0A0G2JEP0_MOUSE	Fragile X mental retardation syndrome-related protein 1 OS=Mus musculus OX=10090 GN=Fxr1 PE=1 SV=1	MOUSE	5
1367	9.96	10.09	20.85	14.58	tr|F8SLQ5|F8SLQ5_MOUSE	Peroxisomal biogenesis factor 5-like protein transcript variant 11 OS=Mus musculus OX=10090 GN=Pex5l PE=2 SV=1	MOUSE	6
1368	9.95	10.13	50.00	23.87	sp|O35593|PSDE_MOUSE	26S proteasome non-ATPase regulatory subunit 14 OS=Mus musculus OX=10090 GN=Psmd14 PE=1 SV=2	MOUSE	5
1369	9.95	10.1	29.81	16.16	sp|Q8BHD8|PCMD2_MOUSE	Protein-L-isoaspartate O-methyltransferase domain-containing protein 2 OS=Mus musculus OX=10090 GN=Pcmtd2 PE=2 SV=1	MOUSE	6
1370	9.92	10.34	25.94	14.27	sp|A2ALS5-3|RPGP1_MOUSE	Isoform 3 of Rap1 GTPase-activating protein 1 OS=Mus musculus OX=10090 GN=Rap1gap	MOUSE	6
1371	9.91	10.14	14.83	8.57	sp|Q3UVL4|VPS51_MOUSE	Vacuolar protein sorting-associated protein 51 homolog OS=Mus musculus OX=10090 GN=Vps51 PE=1 SV=2	MOUSE	6

1372	9.89	10.03	57.04	56.30	tr|Q497I3|Q497I3_MOUSE	Fatty acid binding protein 5, epidermal OS=Mus musculus OX=10090 GN=Fabp5 PE=1 SV=1	MOUSE	5
1373	9.89	10	33.81	21.94	tr|Q3UYX6|Q3UYX6_MOUSE	RRM domain-containing protein OS=Mus musculus OX=10090 GN=Srsf10 PE=2 SV=1	MOUSE	5
1374	9.89	9.92	23.05	23.05	sp|O88271|CFDP1_MOUSE	Craniofacial development protein 1 OS=Mus musculus OX=10090 GN=Cfdp1 PE=1 SV=1	MOUSE	5
1375	9.88	9.99	21.06	13.91	tr|G5E8R8|G5E8R8_MOUSE	UBX domain-containing protein 7 OS=Mus musculus OX=10090 GN=Ubxn7 PE=1 SV=1	MOUSE	5
1376	9.88	9.95	24.30	18.72	sp|Q99KV1|DJB11_MOUSE	DnaJ homolog subfamily B member 11 OS=Mus musculus OX=10090 GN=Dnajb11 PE=1 SV=1	MOUSE	5
1377	9.86	9.94	55.15	55.15	tr|Q3UTI7|Q3UTI7_MOUSE	Peptidyl-prolyl cis-trans isomerase OS=Mus musculus OX=10090 GN=Pin1 PE=2 SV=1	MOUSE	8
1378	9.85	9.92	52.59	52.59	sp|P52503|NDUS6_MOUSE	NADH dehydrogenase [ubiquinone] iron-sulfur protein 6, mitochondrial OS=Mus musculus OX=10090 GN=Ndufs6 PE=1 SV=2	MOUSE	6
1379	9.84	15.84	30.62	14.68	tr|Q3UP61|Q3UP61_MOUSE	Uncharacterized protein OS=Mus musculus OX=10090 GN=Dlg1 PE=2 SV=1	MOUSE	9
1380	9.84	9.91	18.65	11.37	tr|Q3UJX2|Q3UJX2_MOUSE	DUF1716 domain-containing protein OS=Mus musculus OX=10090 GN=Ctnnbl1 PE=2 SV=1	MOUSE	5
1381	9.84	9.9	44.37	39.44	tr|Q3UJ76|Q3UJ76_MOUSE	AP complex subunit sigma OS=Mus musculus OX=10090 GN=Ap2s1 PE=1 SV=1	MOUSE	5
1382	9.82	10.77	28.33	20.94	tr|Q3URM4|Q3URM4_MOUSE	Thioredoxin domain-containing protein OS=Mus musculus OX=10090 GN=Erp44 PE=2 SV=1	MOUSE	6
1383	9.82	10.02	7.09	2.93	tr|S4R2J9|S4R2J9_MOUSE	Protein PRRC2C OS=Mus musculus OX=10090 GN=Prrc2c PE=1 SV=1	MOUSE	6
1384	9.82	9.97	18.28	10.04	sp|O35136|NCAM2_MOUSE	Neural cell adhesion molecule 2 OS=Mus musculus OX=10090 GN=Ncam2 PE=1 SV=1	MOUSE	5
1385	9.79	9.91	47.09	26.98	tr|Q8BU20|Q8BU20_MOUSE	Complex I-SGDH OS=Mus musculus OX=10090 GN=Ndufb5 PE=2 SV=1	MOUSE	6
1386	9.78	11.11	24.71	17.09	tr|Q3UZJ4|Q3UZJ4_MOUSE	Serine/threonine-protein phosphatase 2A 56 kDa regulatory subunit OS=Mus musculus OX=10090 GN=Ppp2r5e PE=1 SV=1	MOUSE	6

1387	9.78	9.89	25.78	20.40	sp|Q9D880|TIM50_MOUSE	Mitochondrial import inner membrane translocase subunit TIM50 OS=Mus musculus OX=10090 GN=Timm50 PE=1 SV=1	MOUSE	5
1388	9.76	10.08	25.94	21.46	tr|Q05CI8|Q05CI8_MOUSE	Ccbl1 protein OS=Mus musculus OX=10090 GN=Kyat1 PE=1 SV=1	MOUSE	7
1389	9.76	9.93	14.49	3.18	tr|Q3UH16|Q3UH16_MOUSE	Uncharacterized protein OS=Mus musculus OX=10090 GN=Kif1a PE=2 SV=1	MOUSE	5
1390	9.74	10.01	37.72	28.74	sp|P61965|WDR5_MOUSE	WD repeat-containing protein 5 OS=Mus musculus OX=10090 GN=Wdr5 PE=1 SV=1	MOUSE	6
1391	9.7	10.11	40.25	28.30	sp|O55091|IMPCT_MOUSE	Protein IMPACT OS=Mus musculus OX=10090 GN=Impact PE=1 SV=2	MOUSE	6
1392	9.7	9.88	35.48	34.19	sp|Q9D6U8|F162A_MOUSE	Protein FAM162A OS=Mus musculus OX=10090 GN=Fam162a PE=1 SV=1	MOUSE	6
1393	9.7	9.85	33.21	31.43	tr|Q571F3|Q571F3_MOUSE	MFLJ00061 protein (Fragment) OS=Mus musculus OX=10090 GN=Necap1 PE=2 SV=1	MOUSE	6
1394	9.68	9.91	22.96	17.92	sp|Q8BGR9|UBCP1_MOUSE	Ubiquitin-like domain-containing CTD phosphatase 1 OS=Mus musculus OX=10090 GN=Ublcp1 PE=1 SV=1	MOUSE	6
1395	9.68	9.86	23.47	14.24	sp|Q8BRF7|SCFD1_MOUSE	Sec1 family domain-containing protein 1 OS=Mus musculus OX=10090 GN=Scfd1 PE=1 SV=1	MOUSE	6
1396	9.68	9.72	45.35	40.12	sp|Q8VED9|LEGL_MOUSE	Galectin-related protein OS=Mus musculus OX=10090 GN=Lgalsl PE=1 SV=1	MOUSE	5
1397	9.67	9.85	51.60	31.05	tr|Q544R8|Q544R8_MOUSE	Trafficking protein particle complex subunit OS=Mus musculus OX=10090 GN=Trappc4 PE=1 SV=1	MOUSE	6
1398	9.66	9.91	48.88	32.74	tr|Q3U489|Q3U489_MOUSE	Adenylate kinase 4, mitochondrial OS=Mus musculus OX=10090 GN=Ak4 PE=1 SV=1	MOUSE	6
1399	9.63	9.96	42.86	30.31	sp|Q3TDK6|ROGDI_MOUSE	Protein rogdi homolog OS=Mus musculus OX=10090 GN=Rogdi PE=1 SV=2	MOUSE	6
1400	9.62	9.74	11.89	7.03	sp|Q8K400|STXB5_MOUSE	Syntaxin-binding protein 5 OS=Mus musculus OX=10090 GN=Stxbp5 PE=1 SV=3	MOUSE	5
1401	9.6	13.25	49.24	49.24	tr|Q9CU14|Q9CU14_MOUSE	PX domain-containing protein (Fragment) OS=Mus musculus OX=10090 GN=Snx3 PE=2 SV=1	MOUSE	8
1402	9.58	14.05	14.73	11.33	tr|A0A5F8MP96|A0A5F8MP96_MOUSE	Septin-4 OS=Mus musculus OX=10090 GN=Septin4 PE=1 SV=1	MOUSE	9

1403	9.52	9.78	43.62	31.38	sp|Q9EQ80|NIF3L_MOUSE	NIF3-like protein 1 OS=Mus musculus OX=10090 GN=Nif3l1 PE=1 SV=4	MOUSE	6
1404	9.52	9.57	57.75	56.34	tr|Q5EBI8|Q5EBI8_MOUSE	ATP synthase subunit e, mitochondrial OS=Mus musculus OX=10090 GN=Atp5k PE=2 SV=1	MOUSE	5
1405	9.5	9.77	27.35	16.57	tr|Q3UH04|Q3UH04_MOUSE	Protein kinase domain-containing protein OS=Mus musculus OX=10090 GN=Camk1d PE=2 SV=1	MOUSE	6
1406	9.5	9.66	51.45	41.49	tr|Q542F1|Q542F1_MOUSE	Chloride intracellular channel protein OS=Mus musculus OX=10090 GN=Clic1 PE=1 SV=1	MOUSE	6
1407	9.5	9.56	45.65	39.86	tr|Q9D181|Q9D181_MOUSE	U6 snRNA-associated Sm-like protein LSm4 OS=Mus musculus OX=10090 GN=Lsm4 PE=2 SV=1	MOUSE	5
1408	9.49	9.74	18.46	8.13	tr|Q3TUQ5|Q3TUQ5_MOUSE	Pinin OS=Mus musculus OX=10090 GN=Pnn PE=1 SV=1	MOUSE	5
1409	9.49	9.6	35.71	29.46	sp|Q9DC70|NDUS7_MOUSE	NADH dehydrogenase [ubiquinone] iron-sulfur protein 7, mitochondrial OS=Mus musculus OX=10090 GN=Ndufs7 PE=1 SV=1	MOUSE	6
1410	9.49	9.53	19.21	16.59	tr|Q80V84|Q80V84_MOUSE	HtrA serine peptidase 2 OS=Mus musculus OX=10090 GN=Htra2 PE=2 SV=1	MOUSE	6
1411	9.48	9.65	22.59	19.25	tr|Q921L6|Q921L6_MOUSE	Cttn protein OS=Mus musculus OX=10090 GN=Cttn PE=1 SV=1	MOUSE	7
1412	9.48	9.61	34.62	28.40	tr|Q3UTV3|Q3UTV3_MOUSE	Uncharacterized protein (Fragment) OS=Mus musculus OX=10090 GN=Gdap1 PE=2 SV=1	MOUSE	6
1413	9.47	15.82	51.00	43.00	tr|Q4FJL0|Q4FJL0_MOUSE	Rab10 OS=Mus musculus OX=10090 GN=Rab10 PE=1 SV=1	MOUSE	8
1414	9.47	9.57	22.51	22.51	sp|Q99J09|MEP50_MOUSE	Methylosome protein 50 OS=Mus musculus OX=10090 GN=Wdr77 PE=1 SV=1	MOUSE	6
1415	9.47	9.52	30.04	26.18	sp|Q9D6Y7|MSRA_MOUSE	Mitochondrial peptide methionine sulfoxide reductase OS=Mus musculus OX=10090 GN=Msra PE=1 SV=1	MOUSE	5
1416	9.44	9.58	61.86	58.47	tr|Q54A87|Q54A87_MOUSE	V-type proton ATPase subunit G OS=Mus musculus OX=10090 GN=Atp6v1g2 PE=1 SV=1	MOUSE	6
1417	9.43	9.67	31.26	26.77	sp|Q8CBY8|DCTN4_MOUSE	Dynactin subunit 4 OS=Mus musculus OX=10090 GN=Dctn4 PE=1 SV=1	MOUSE	10
1418	9.43	9.63	40.98	26.50	sp|P97355|SPSY_MOUSE	Spermine synthase OS=Mus musculus OX=10090 GN=Sms PE=1 SV=1	MOUSE	6

1419	9.43	9.5	48.08	47.12	tr|Q4FZH7|Q4FZH7_MOUSE	Enhancer of rudimentary homolog OS=Mus musculus OX=10090 GN=Erh PE=1 SV=1	MOUSE	5
1420	9.42	29.83	55.04	43.33	tr|Q3T9L0|Q3T9L0_MOUSE	RNA helicase OS=Mus musculus OX=10090 GN=Ddx39a PE=2 SV=1	MOUSE	17
1421	9.42	9.68	17.43	13.03	tr|Q69ZY2|Q69ZY2_MOUSE	MKIAA0830 protein (Fragment) OS=Mus musculus OX=10090 GN=Endod1 PE=2 SV=1	MOUSE	6
1422	9.41	9.68	35.50	18.49	tr|Q68FH8|Q68FH8_MOUSE	Smarcd1 protein OS=Mus musculus OX=10090 GN=Smarcd1 PE=2 SV=2	MOUSE	7
1423	9.41	9.45	31.82	28.79	sp|P99026|PSB4_MOUSE	Proteasome subunit beta type-4 OS=Mus musculus OX=10090 GN=Psmb4 PE=1 SV=1	MOUSE	6
1424	9.38	9.52	65.83	38.33	sp|Q9CQ54|NDUC2_MOUSE	NADH dehydrogenase [ubiquinone] 1 subunit C2 OS=Mus musculus OX=10090 GN=Ndufc2 PE=1 SV=1	MOUSE	5
1425	9.36	9.48	7.73	4.42	tr|Q8C2N9|Q8C2N9_MOUSE	DNA mismatch repair protein OS=Mus musculus OX=10090 GN=Msh6 PE=2 SV=1	MOUSE	5
1426	9.34	9.51	19.27	11.71	sp|P27612|PLAP_MOUSE	Phospholipase A-2-activating protein OS=Mus musculus OX=10090 GN=Plaa PE=1 SV=4	MOUSE	7
1427	9.34	9.47	26.32	20.05	sp|O89112|LANC1_MOUSE	Glutathione S-transferase LANCL1 OS=Mus musculus OX=10090 GN=Lancl1 PE=1 SV=1	MOUSE	7
1428	9.34	9.41	46.82	28.64	tr|A0A0J9YV85|A0A0J9YV85_MOUSE	Ras-related protein Rab-28 OS=Mus musculus OX=10090 GN=Rab28 PE=1 SV=1	MOUSE	5
1429	9.33	9.97	6.14	1.47	sp|A2AN08|UBR4_MOUSE	E3 ubiquitin-protein ligase UBR4 OS=Mus musculus OX=10090 GN=Ubr4 PE=1 SV=1	MOUSE	5
1430	9.33	9.41	22.99	11.88	tr|Q3UHZ7|Q3UHZ7_MOUSE	UDP-N-acetylhexosamine pyrophosphorylase OS=Mus musculus OX=10090 GN=Uap1 PE=1 SV=1	MOUSE	7
1431	9.32	9.79	33.05	18.46	tr|Q3USR8|Q3USR8_MOUSE	Uncharacterized protein (Fragment) OS=Mus musculus OX=10090 GN=Ctcf PE=2 SV=1	MOUSE	5
1432	9.3	9.51	8.06	5.94	tr|K3W4L0|K3W4L0_MOUSE	Unconventional myosin-XVIIIa OS=Mus musculus OX=10090 GN=Myo18a PE=1 SV=1	MOUSE	7
1433	9.28	13.06	48.56	44.03	tr|Q8BH80|Q8BH80_MOUSE	Vesicle-associated membrane protein, associated protein B and C OS=Mus musculus OX=10090 GN=Vapb PE=1 SV=1	MOUSE	10
1434	9.26	9.38	32.26	25.81	tr|Q3UVR2|Q3UVR2_MOUSE	Uncharacterized protein OS=Mus musculus OX=10090 GN=Cntfr PE=2 SV=1	MOUSE	6
1435	9.26	9.37	38.77	29.52	sp|Q9WTP7|KAD3_MOUSE	GTP:AMP phosphotransferase AK3, mitochondrial OS=Mus musculus OX=10090 GN=Ak3 PE=1 SV=3	MOUSE	6

1436	9.26	9.34	43.29	41.13	sp|Q9WTL7|LYPA2_MOUSE	Acyl-protein thioesterase 2 OS=Mus musculus OX=10090 GN=Lypla2 PE=1 SV=1	MOUSE	6
1437	9.25	9.35	45.24	45.24	tr|Q564F6|Q564F6_MOUSE	Programmed cell death 5 OS=Mus musculus OX=10090 GN=Pdcd5 PE=1 SV=1	MOUSE	5
1438	9.22	9.46	28.51	19.22	sp|P21956|MFGM_MOUSE	Lactadherin OS=Mus musculus OX=10090 GN=Mfge8 PE=1 SV=3	MOUSE	6
1439	9.22	9.37	36.88	32.50	tr|Q3TJG6|Q3TJG6_MOUSE	CS domain-containing protein OS=Mus musculus OX=10090 GN=Ptges3 PE=2 SV=1	MOUSE	5
1440	9.21	9.41	28.57	18.94	tr|Q7TPW6|Q7TPW6_MOUSE	Solute carrier family 25 (Mitochondrial carnitine/acylcarnitine translocase), member 20 OS=Mus musculus OX=10090 GN=Slc25a20 PE=2 SV=1	MOUSE	6
1441	9.2	9.33	30.15	27.48	sp|P57759|ERP29_MOUSE	Endoplasmic reticulum resident protein 29 OS=Mus musculus OX=10090 GN=Erp29 PE=1 SV=2	MOUSE	6
1442	9.19	9.36	29.77	29.10	tr|Q543C9|Q543C9_MOUSE	Homeobox domain-containing protein OS=Mus musculus OX=10090 GN=Crx PE=2 SV=1	MOUSE	8
1443	9.18	14.26	44.06	44.06	tr|Q5RKN9|Q5RKN9_MOUSE	F-actin-capping protein subunit alpha OS=Mus musculus OX=10090 GN=Capza1 PE=1 SV=1	MOUSE	8
1444	9.18	9.35	19.28	12.85	sp|P36916|GNL1_MOUSE	Guanine nucleotide-binding protein-like 1 OS=Mus musculus OX=10090 GN=Gnl1 PE=1 SV=4	MOUSE	6
1445	9.1	20.98	19.13	10.27	sp|Q91W97|HKDC1_MOUSE	Hexokinase HKDC1 OS=Mus musculus OX=10090 GN=Hkdc1 PE=2 SV=1	MOUSE	10
1446	9.1	9.22	23.68	17.24	tr|A0A0J9YVG0|A0A0J9YVG0_MOUSE	Protein phosphatase 1G OS=Mus musculus OX=10090 GN=Ppm1g PE=1 SV=1	MOUSE	5
1447	9.1	9.18	15.52	10.52	tr|Q32P04|Q32P04_MOUSE	Keratin 5 OS=Mus musculus OX=10090 GN=Krt5 PE=1 SV=2	MOUSE	6
1448	9.09	18.36	68.67	55.36	sp|Q8JZS0|LIN7A_MOUSE	Protein lin-7 homolog A OS=Mus musculus OX=10090 GN=Lin7a PE=1 SV=2	MOUSE	11
1449	9.09	10.46	79.67	69.11	tr|Q5GQ64|Q5GQ64_MOUSE	Gamma-synuclein (Fragment) OS=Mus musculus OX=10090 GN=Sncg PE=1 SV=1	MOUSE	6
1450	9.08	9.32	26.04	13.60	tr|E9PV22|E9PV22_MOUSE	Leucine-rich repeat-containing protein 47 OS=Mus musculus OX=10090 GN=Lrrc47 PE=1 SV=1	MOUSE	6
1451	9.06	9.19	26.47	16.04	tr|Q3V199|Q3V199_MOUSE	W2 domain-containing protein (Fragment) OS=Mus musculus OX=10090 GN=Bzw2 PE=2 SV=1	MOUSE	6

1452	9.05	9.08	55.56	45.83	tr|D3Z645|D3Z645_MOUSE	Vacuolar protein sorting-associated protein 29 OS=Mus musculus OX=10090 GN=Vps29 PE=1 SV=1	MOUSE	5
1453	9.04	11.38	30.36	23.93	tr|A0A0N4SVC2|A0A0N4SVC2_MOUSE	Transformer-2 protein homolog alpha OS=Mus musculus OX=10090 GN=Tra2a PE=1 SV=1	MOUSE	6
1454	9.02	9.07	28.71	25.25	sp|P43081|GUC1A_MOUSE	Guanylyl cyclase-activating protein 1 OS=Mus musculus OX=10090 GN=Guca1a PE=1 SV=3	MOUSE	5
1455	9.01	9.24	15.57	9.86	tr|Q3U3A8|Q3U3A8_MOUSE	Calcium-activated neutral proteinase 2 OS=Mus musculus OX=10090 GN=Capn2 PE=2 SV=1	MOUSE	5
1456	9	10.17	28.39	22.25	tr|Q3UCW0|Q3UCW0_MOUSE	Uncharacterized protein OS=Mus musculus OX=10090 GN=Tsg101 PE=1 SV=1	MOUSE	6
1457	8.98	9.13	38.02	23.64	sp|Q99L04|DHRS1_MOUSE	Dehydrogenase/reductase SDR family member 1 OS=Mus musculus OX=10090 GN=Dhrs1 PE=1 SV=1	MOUSE	6
1458	8.98	9.02	70.59	70.59	sp|Q9CQX8|RT36_MOUSE	28S ribosomal protein S36, mitochondrial OS=Mus musculus OX=10090 GN=Mrps36 PE=1 SV=1	MOUSE	5
1459	8.96	9.21	67.72	42.41	sp|Q9D289|TPC6B_MOUSE	Trafficking protein particle complex subunit 6B OS=Mus musculus OX=10090 GN=Trappc6b PE=1 SV=1	MOUSE	6
1460	8.95	11.93	12.04	6.78	tr|J9RV59|J9RV59_MOUSE	Anion exchange protein OS=Mus musculus OX=10090 GN=Slc4a7 PE=1 SV=1	MOUSE	6
1461	8.95	9.1	25.67	12.17	tr|Q3UJ53|Q3UJ53_MOUSE	Amine oxidase OS=Mus musculus OX=10090 GN=Maoa PE=2 SV=1	MOUSE	5
1462	8.94	9.04	23.75	21.46	tr|Q91ZH2|Q91ZH2_MOUSE	Proliferating cell nuclear antigen OS=Mus musculus OX=10090 GN=Pcna PE=2 SV=1	MOUSE	5
1463	8.92	9.59	24.67	17.67	tr|Q3V0T8|Q3V0T8_MOUSE	Uncharacterized protein OS=Mus musculus OX=10090 GN=Ggps1 PE=1 SV=1	MOUSE	5
1464	8.92	8.98	41.44	41.44	tr|Q3U6E4|Q3U6E4_MOUSE	Uncharacterized protein OS=Mus musculus OX=10090 GN=Ptma PE=2 SV=1	MOUSE	6
1465	8.91	9.06	30.50	12.87	tr|Q3UYS0|Q3UYS0_MOUSE	Uncharacterized protein OS=Mus musculus OX=10090 GN=Pccb PE=2 SV=1	MOUSE	5
1466	8.91	8.98	43.28	26.89	sp|Q60692|PSB6_MOUSE	Proteasome subunit beta type-6 OS=Mus musculus OX=10090 GN=Psmb6 PE=1 SV=3	MOUSE	5
1467	8.9	9.08	22.48	9.53	tr|Q3UYN2|Q3UYN2_MOUSE	Copine-6 OS=Mus musculus OX=10090 GN=Cpne6 PE=1 SV=1	MOUSE	6
1468	8.88	9.87	17.19	9.60	tr|Q3UDL9|Q3UDL9_MOUSE	Uncharacterized protein (Fragment) OS=Mus musculus OX=10090 GN=Bclaf1 PE=2 SV=1	MOUSE	6

1469	8.88	9.05	13.47	7.89	tr|Q8BWY6|Q8BWY6_MOUSE	Alpha-mannosidase OS=Mus musculus OX=10090 GN=Man2c1 PE=2 SV=1	MOUSE	5
1470	8.88	8.98	22.80	17.03	sp|Q9QXB9|DRG2_MOUSE	Developmentally-regulated GTP-binding protein 2 OS=Mus musculus OX=10090 GN=Drg2 PE=1 SV=1	MOUSE	5
1471	8.87	8.98	18.18	9.57	tr|Q5RL55|Q5RL55_MOUSE	ATP-binding cassette, sub-family F (GCN20), member 1 OS=Mus musculus OX=10090 GN=Abcf1 PE=2 SV=1	MOUSE	5
1472	8.86	9.14	31.39	13.38	sp|O54941|SMCE1_MOUSE	SWI/SNF-related matrix-associated actin-dependent regulator of chromatin subfamily E member 1 OS=Mus musculus OX=10090 GN=Smarce1 PE=1 SV=1	MOUSE	5
1473	8.86	8.91	18.29	10.66	sp|Q8BI72|CARF_MOUSE	CDKN2A-interacting protein OS=Mus musculus OX=10090 GN=Cdkn2aip PE=1 SV=1	MOUSE	5
1474	8.85	9.64	21.01	11.83	sp|Q922Q1|MARC2_MOUSE	Mitochondrial amidoxime reducing component 2 OS=Mus musculus OX=10090 GN=Mtarc2 PE=1 SV=1	MOUSE	5
1475	8.84	9.04	11.90	4.94	sp|Q8VDC1|FYCO1_MOUSE	FYVE and coiled-coil domain-containing protein 1 OS=Mus musculus OX=10090 GN=Fyco1 PE=1 SV=1	MOUSE	5
1476	8.82	8.86	7.52	5.24	tr|H3BL37|H3BL37_MOUSE	Treacle protein OS=Mus musculus OX=10090 GN=Tcof1 PE=1 SV=1	MOUSE	5
1477	8.81	9.01	12.62	4.21	tr|B9EKP5|B9EKP5_MOUSE	Filamin, alpha OS=Mus musculus OX=10090 GN=Flna PE=2 SV=1	MOUSE	6
1478	8.78	16.76	36.10	31.33	tr|Q58E49|Q58E49_MOUSE	Histone deacetylase OS=Mus musculus OX=10090 GN=Hdac1 PE=2 SV=1	MOUSE	10
1479	8.76	8.89	20.72	7.57	tr|Q8C7S2|Q8C7S2_MOUSE	LIM zinc-binding domain-containing protein OS=Mus musculus OX=10090 GN=Lima1 PE=2 SV=1	MOUSE	4
1480	8.75	8.88	41.01	26.27	tr|Q3U5I5|Q3U5I5_MOUSE	Uncharacterized protein OS=Mus musculus OX=10090 GN=Grb2 PE=2 SV=1	MOUSE	6
1481	8.75	8.79	20.51	17.95	tr|B2RTE3|B2RTE3_MOUSE	Pre-mRNA-splicing factor 38A OS=Mus musculus OX=10090 GN=Prpf38a PE=2 SV=1	MOUSE	5
1482	8.74	8.78	52.98	46.36	tr|Q4VBC9|Q4VBC9_MOUSE	Complex I-ESSS (Fragment) OS=Mus musculus OX=10090 GN=Ndufb11 PE=2 SV=1	MOUSE	5
1483	8.71	15.13	36.39	23.30	sp|P49443|PPM1A_MOUSE	Protein phosphatase 1A OS=Mus musculus OX=10090 GN=Ppm1a PE=1 SV=1	MOUSE	7
1484	8.71	8.84	45.79	23.23	tr|Q3USR5|Q3USR5_MOUSE	Uncharacterized protein OS=Mus musculus OX=10090 GN=Fbxo2 PE=1 SV=1	MOUSE	5

1485	8.71	8.82	50.20	29.72	tr|Q5HZK3|Q5HZK3_MOUSE	Proteasome (Prosome, macropain) 28 subunit, alpha OS=Mus musculus OX=10090 GN=Psme1 PE=1 SV=1	MOUSE	5
1486	8.71	8.77	40.00	20.00	tr|Q3UW40|Q3UW40_MOUSE	TRASH domain-containing protein OS=Mus musculus OX=10090 GN=Rpl24 PE=2 SV=1	MOUSE	5
1487	8.71	8.77	34.18	22.45	tr|Q5I0T8|Q5I0T8_MOUSE	Ribosomal protein L19 OS=Mus musculus OX=10090 GN=Rpl19 PE=1 SV=1	MOUSE	6
1488	8.7	8.83	18.09	7.36	sp|Q9DBR1|XRN2_MOUSE	5'-3' exoribonuclease 2 OS=Mus musculus OX=10090 GN=Xrn2 PE=1 SV=1	MOUSE	5
1489	8.7	8.75	25.90	21.64	sp|Q99M28|RNPS1_MOUSE	RNA-binding protein with serine-rich domain 1 OS=Mus musculus OX=10090 GN=Rnps1 PE=1 SV=1	MOUSE	6
1490	8.69	8.73	76.84	67.37	tr|Q3U9X2|Q3U9X2_MOUSE	U6 snRNA-associated Sm-like protein LSm2 OS=Mus musculus OX=10090 GN=Lsm2 PE=1 SV=1	MOUSE	5
1491	8.68	10.92	42.59	22.22	tr|Q9D8S5|Q9D8S5_MOUSE	Serine/arginine-rich-splicing factor 5 OS=Mus musculus OX=10090 GN=Srsf5 PE=1 SV=1	MOUSE	6
1492	8.68	8.9	42.72	26.93	tr|Q3ULN6|Q3ULN6_MOUSE	Sacchrp_dh_NADP domain-containing protein OS=Mus musculus OX=10090 GN=Sccpdh PE=2 SV=1	MOUSE	6
1493	8.66	27.18	29.91	20.69	tr|Q3U111|Q3U111_MOUSE	FERM domain-containing protein (Fragment) OS=Mus musculus OX=10090 GN=Rdx PE=2 SV=1	MOUSE	14
1494	8.64	9.02	5.71	1.79	sp|P11531|DMD_MOUSE	Dystrophin OS=Mus musculus OX=10090 GN=Dmd PE=1 SV=3	MOUSE	6
1495	8.64	8.74	34.03	34.03	sp|Q8BMJ3|IF1AX_MOUSE	Eukaryotic translation initiation factor 1A, X-chromosomal OS=Mus musculus OX=10090 GN=Eif1ax PE=2 SV=3	MOUSE	5
1496	8.64	8.72	14.99	11.53	tr|Q9MD82|Q9MD82_MOUSE	NADH-ubiquinone oxidoreductase chain 5 OS=Mus musculus OX=10090 GN=mt-Nd5 PE=1 SV=1	MOUSE	5
1497	8.62	8.83	57.81	40.63	sp|Q9D0T1|NH2L1_MOUSE	NHP2-like protein 1 OS=Mus musculus OX=10090 GN=Snu13 PE=1 SV=4	MOUSE	6
1498	8.59	8.62	18.81	18.81	sp|Q9D0J8|PTMS_MOUSE	Parathymosin OS=Mus musculus OX=10090 GN=Ptms PE=1 SV=3	MOUSE	5
1499	8.57	8.74	44.97	20.12	tr|Q3TL79|Q3TL79_MOUSE	Aha1_N domain-containing protein OS=Mus musculus OX=10090 GN=Ahsa1 PE=2 SV=1	MOUSE	5
1500	8.57	8.6	33.33	22.62	tr|Q9D3C4|Q9D3C4_MOUSE	Actin-related protein 2/3 complex subunit 4 OS=Mus musculus OX=10090 GN=Arpc4 PE=2 SV=1	MOUSE	5
1501	8.55	8.75	27.62	22.14	tr|Q3U8V1|Q3U8V1_MOUSE	RRM domain-containing protein OS=Mus musculus OX=10090 GN=Poldip3 PE=2 SV=1	MOUSE	6

1502	8.53	8.65	37.35	33.13	tr|Q3U0J1|Q3U0J1_MOUSE	Peptidyl-prolyl cis-trans isomerase OS=Mus musculus OX=10090 GN=Ppil1 PE=2 SV=1	MOUSE	6
1503	8.52	8.61	41.90	31.90	tr|E9QN99|E9QN99_MOUSE	Protein ABHD14B OS=Mus musculus OX=10090 GN=Abhd14b PE=1 SV=1	MOUSE	6
1504	8.51	8.64	51.04	38.54	sp|P18608|HMGN1_MOUSE	Non-histone chromosomal protein HMG-14 OS=Mus musculus OX=10090 GN=Hmgn1 PE=1 SV=2	MOUSE	13
1505	8.5	8.66	52.86	50.00	tr|Q52KP0|Q52KP0_MOUSE	60S ribosomal protein L38 OS=Mus musculus OX=10090 GN=Rpl38 PE=1 SV=1	MOUSE	6
1506	8.48	8.54	24.24	21.55	sp|Q91V64|ISOC1_MOUSE	Isochorismatase domain-containing protein 1 OS=Mus musculus OX=10090 GN=Isoc1 PE=1 SV=1	MOUSE	5
1507	8.45	8.49	38.40	28.27	sp|Q148R9|R9BP_MOUSE	Regulator of G-protein signaling 9-binding protein OS=Mus musculus OX=10090 GN=Rgs9bp PE=1 SV=2	MOUSE	5
1508	8.44	8.68	23.86	14.22	tr|Q3U6T2|Q3U6T2_MOUSE	Alpha-galactosidase OS=Mus musculus OX=10090 GN=Naga PE=2 SV=1	MOUSE	5
1509	8.43	8.71	12.90	9.14	sp|Q9Z1Q2|ABHGA_MOUSE	Phosphatidylserine lipase ABHD16A OS=Mus musculus OX=10090 GN=Abhd16a PE=1 SV=3	MOUSE	5
1510	8.42	17.35	44.44	24.50	sp|P05132|KAPCA_MOUSE	cAMP-dependent protein kinase catalytic subunit alpha OS=Mus musculus OX=10090 GN=Prkaca PE=1 SV=3	MOUSE	10
1511	8.42	8.52	54.46	54.46	tr|Q8CFZ0|Q8CFZ0_MOUSE	SUMO-conjugating enzyme UBC9 OS=Mus musculus OX=10090 GN=Ube2i PE=1 SV=1	MOUSE	5
1512	8.41	8.55	29.93	14.19	tr|Q3TX06|Q3TX06_MOUSE	Uncharacterized protein OS=Mus musculus OX=10090 GN=Gorasp2 PE=2 SV=1	MOUSE	4
1513	8.4	8.54	24.30	11.59	sp|P05480|SRC_MOUSE	Neuronal proto-oncogene tyrosine-protein kinase Src OS=Mus musculus OX=10090 GN=Src PE=1 SV=5	MOUSE	5
1514	8.4	8.47	27.40	10.71	tr|Q80UJ8|Q80UJ8_MOUSE	Lactb protein OS=Mus musculus OX=10090 GN=Lactb PE=2 SV=1	MOUSE	4
1515	8.38	8.44	21.84	17.41	tr|Q9R1R9|Q9R1R9_MOUSE	UBE-1a OS=Mus musculus OX=10090 GN=Rdh11 PE=2 SV=1	MOUSE	4
1516	8.36	8.59	31.45	21.96	tr|A0A1B0GS63|A0A1B0GS63_MOUSE	Arfaptin-2 OS=Mus musculus OX=10090 GN=Arfip2 PE=1 SV=1	MOUSE	5
1517	8.35	8.43	11.92	7.32	tr|Q4G0C0|Q4G0C0_MOUSE	Sec23 interacting protein OS=Mus musculus OX=10090 GN=Sec23ip PE=2 SV=1	MOUSE	4

1518	8.34	8.53	18.01	6.65	sp|Q3V3R1|C1TM_MOUSE	Monofunctional C1-tetrahydrofolate synthase, mitochondrial OS=Mus musculus OX=10090 GN=Mthfd1l PE=1 SV=2	MOUSE	5
1519	8.33	48.18	41.19	27.01	tr|Q6ZQ49|Q6ZQ49_MOUSE	Sodium/potassium-transporting ATPase subunit alpha (Fragment) OS=Mus musculus OX=10090 GN=Atp1a2 PE=2 SV=1	MOUSE	45
1520	8.33	8.54	12.11	3.38	tr|Q7TSJ0|Q7TSJ0_MOUSE	DNA (cytosine-5)-methyltransferase OS=Mus musculus OX=10090 GN=Dnmt1 PE=2 SV=1	MOUSE	5
1521	8.32	8.34	39.89	30.90	tr|Q3UCC5|Q3UCC5_MOUSE	PEST proteolytic signal-containing nuclear protein OS=Mus musculus OX=10090 GN=Pcnp PE=1 SV=1	MOUSE	4
1522	8.31	8.43	49.10	28.38	sp|Q9CR00|PSMD9_MOUSE	26S proteasome non-ATPase regulatory subunit 9 OS=Mus musculus OX=10090 GN=Psmd9 PE=1 SV=1	MOUSE	5
1523	8.31	8.41	60.10	32.51	sp|P34022|RANG_MOUSE	Ran-specific GTPase-activating protein OS=Mus musculus OX=10090 GN=Ranbp1 PE=1 SV=2	MOUSE	4
1524	8.31	8.4	17.51	6.55	tr|Q571A2|Q571A2_MOUSE	MKIAA4106 protein (Fragment) OS=Mus musculus OX=10090 GN=Cul2 PE=2 SV=1	MOUSE	4
1525	8.31	8.33	32.03	21.88	tr|A0A0N4SUH8|A0A0N4SUH8_MOUSE	NFU1 iron-sulfur cluster scaffold homolog, mitochondrial OS=Mus musculus OX=10090 GN=Nfu1 PE=1 SV=1	MOUSE	4
1526	8.29	8.91	34.16	31.37	tr|Q8K239|Q8K239_MOUSE	Tsfm protein (Fragment) OS=Mus musculus OX=10090 GN=Tsfm PE=2 SV=1	MOUSE	5
1527	8.28	8.34	25.38	15.11	sp|Q9JL56|GDE1_MOUSE	Glycerophosphodiester phosphodiesterase 1 OS=Mus musculus OX=10090 GN=Gde1 PE=1 SV=1	MOUSE	4
1528	8.27	8.41	24.27	15.06	tr|Q58E65|Q58E65_MOUSE	Methionine aminopeptidase 2 OS=Mus musculus OX=10090 GN=Metap2 PE=1 SV=1	MOUSE	5
1529	8.27	8.35	38.20	24.89	tr|Q2VPR3|Q2VPR3_MOUSE	2500003M10Rik protein (Fragment) OS=Mus musculus OX=10090 GN=Chtop PE=2 SV=1	MOUSE	5
1530	8.27	8.33	36.36	21.82	tr|Q9DC85|Q9DC85_MOUSE	60S ribosomal protein L35a OS=Mus musculus OX=10090 GN=Rpl35a PE=2 SV=1	MOUSE	4
1531	8.25	8.31	32.00	21.71	sp|P04344|CRGB_MOUSE	Gamma-crystallin B OS=Mus musculus OX=10090 GN=Crygb PE=2 SV=4	MOUSE	5
1532	8.23	8.46	16.27	10.43	sp|Q3B7Z2|OSBP1_MOUSE	Oxysterol-binding protein 1 OS=Mus musculus OX=10090 GN=Osbp PE=1 SV=3	MOUSE	6
1533	8.21	8.44	14.15	6.38	sp|O35551|RABE1_MOUSE	Rab GTPase-binding effector protein 1 OS=Mus musculus OX=10090 GN=Rabep1 PE=1 SV=2	MOUSE	4

1534	8.21	8.28	19.20	11.46	tr|Q544D7|Q544D7_MOUSE	AMP-binding domain-containing protein OS=Mus musculus OX=10090 GN=Slc27a1 PE=1 SV=1	MOUSE	4
1535	8.2	8.29	38.38	33.84	sp|Q9CQW1|YKT6_MOUSE	Synaptobrevin homolog YKT6 OS=Mus musculus OX=10090 GN=Ykt6 PE=1 SV=1	MOUSE	5
1536	8.19	8.3	49.75	29.65	sp|Q62393-3|TPD52_MOUSE	Isoform 3 of Tumor protein D52 OS=Mus musculus OX=10090 GN=Tpd52	MOUSE	6
1537	8.19	8.29	28.40	25.93	tr|Q3TSW9|Q3TSW9_MOUSE	Uncharacterized protein OS=Mus musculus OX=10090 GN=Tfam PE=2 SV=1	MOUSE	5
1538	8.18	8.24	22.94	16.18	sp|Q61249|IGBP1_MOUSE	Immunoglobulin-binding protein 1 OS=Mus musculus OX=10090 GN=Igbp1 PE=1 SV=1	MOUSE	4
1539	8.17	10.46	4.46	1.99	sp|Q9QYX7|PCLO_MOUSE	Protein piccolo OS=Mus musculus OX=10090 GN=Pclo PE=1 SV=4	MOUSE	6
1540	8.16	8.24	24.46	17.27	tr|Q3TEE8|Q3TEE8_MOUSE	Uncharacterized protein OS=Mus musculus OX=10090 GN=Txndc5 PE=1 SV=1	MOUSE	5
1541	8.15	8.26	20.07	12.34	sp|Q3TIU4|PDE12_MOUSE	2',5'-phosphodiesterase 12 OS=Mus musculus OX=10090 GN=Pde12 PE=1 SV=2	MOUSE	5
1542	8.15	8.17	19.96	15.07	sp|Q8BTV2|CPSF7_MOUSE	Cleavage and polyadenylation specificity factor subunit 7 OS=Mus musculus OX=10090 GN=Cpsf7 PE=1 SV=2	MOUSE	5
1543	8.14	12.46	33.15	30.62	tr|Q8BK29|Q8BK29_MOUSE	Pribosyltran_N domain-containing protein OS=Mus musculus OX=10090 GN=Prpsap1 PE=2 SV=1	MOUSE	8
1544	8.14	8.22	30.61	17.27	sp|O08915|AIP_MOUSE	AH receptor-interacting protein OS=Mus musculus OX=10090 GN=Aip PE=1 SV=1	MOUSE	5
1545	8.14	8.19	31.67	27.60	sp|Q9D1C8|VPS28_MOUSE	Vacuolar protein sorting-associated protein 28 homolog OS=Mus musculus OX=10090 GN=Vps28 PE=1 SV=1	MOUSE	4
1546	8.13	8.74	46.41	32.04	tr|Q14AC7|Q14AC7_MOUSE	ADP-ribosylation factor-like 1 OS=Mus musculus OX=10090 GN=Arl1 PE=1 SV=1	MOUSE	5
1547	8.13	8.31	39.32	15.38	sp|Q3ULJ0|GPD1L_MOUSE	Glycerol-3-phosphate dehydrogenase 1-like protein OS=Mus musculus OX=10090 GN=Gpd1l PE=1 SV=2	MOUSE	5
1548	8.13	8.28	20.71	15.95	tr|Q3UKN6|Q3UKN6_MOUSE	Nucleobindin-2 OS=Mus musculus OX=10090 GN=Nucb2 PE=1 SV=1	MOUSE	5
1549	8.13	8.2	47.27	47.27	sp|P16254|SRP14_MOUSE	Signal recognition particle 14 kDa protein OS=Mus musculus OX=10090 GN=Srp14 PE=1 SV=1	MOUSE	6
1550	8.12	8.24	27.87	27.87	tr|F6TQL3|F6TQL3_MOUSE	DnaJ homolog subfamily C member 8 (Fragment) OS=Mus musculus OX=10090 GN=Dnajc8 PE=1 SV=1	MOUSE	5

1551	8.12	8.2	42.91	29.15	sp|Q9CQE1|NPS3B_MOUSE	Protein NipSnap homolog 3B OS=Mus musculus OX=10090 GN=Nipsnap3b PE=1 SV=1	MOUSE	5
1552	8.12	8.19	31.35	31.35	tr|A0A0U1RP13|A0A0U1RP13_MOUSE	CDP-diacylglycerol--inositol 3-phosphatidyltransferase OS=Mus musculus OX=10090 GN=Cdipt PE=1 SV=1	MOUSE	6
1553	8.12	8.13	59.67	40.88	sp|Q9DB27|MCTS1_MOUSE	Malignant T-cell-amplified sequence 1 OS=Mus musculus OX=10090 GN=Mcts1 PE=1 SV=1	MOUSE	4
1554	8.12	8.13	59.20	47.70	sp|Q9CWZ3|RBM8A_MOUSE	RNA-binding protein 8A OS=Mus musculus OX=10090 GN=Rbm8a PE=1 SV=4	MOUSE	4
1555	8.1	8.23	12.05	4.92	tr|H3BIW6|H3BIW6_MOUSE	Phosphoinositide phospholipase C OS=Mus musculus OX=10090 GN=Plch2 PE=1 SV=1	MOUSE	4
1556	8.09	8.15	53.71	30.29	tr|Q3U0D7|Q3U0D7_MOUSE	ADP-ribosylation factor 6 OS=Mus musculus OX=10090 GN=Arf6 PE=1 SV=1	MOUSE	4
1557	8.07	10.61	18.44	14.18	tr|Q922Z3|Q922Z3_MOUSE	Trap1 protein (Fragment) OS=Mus musculus OX=10090 GN=Trap1 PE=2 SV=1	MOUSE	7
1558	8.06	8.07	27.52	22.87	tr|Q9DA45|Q9DA45_MOUSE	Uncharacterized protein OS=Mus musculus OX=10090 GN=Chchd6 PE=2 SV=1	MOUSE	7
1559	8.06	8.07	43.90	42.68	tr|A0A0G2JDW7|A0A0G2JDW7_MOUSE	40S ribosomal protein S27 (Fragment) OS=Mus musculus OX=10090 GN=Rps27 PE=1 SV=1	MOUSE	5
1560	8.06	8.07	21.40	21.40	sp|Q8R191|SNG3_MOUSE	Synaptogyrin-3 OS=Mus musculus OX=10090 GN=Syngr3 PE=1 SV=1	MOUSE	6
1561	8.05	8.15	29.28	22.65	tr|B2RTB0|B2RTB0_MOUSE	PDGFA associated protein 1 OS=Mus musculus OX=10090 GN=Pdap1 PE=1 SV=1	MOUSE	6
1562	8.04	8.15	24.90	12.35	sp|Q8BGW1|FTO_MOUSE	Alpha-ketoglutarate-dependent dioxygenase FTO OS=Mus musculus OX=10090 GN=Fto PE=1 SV=1	MOUSE	6
1563	8.04	8.07	50.87	24.35	sp|P97823|LYPA1_MOUSE	Acyl-protein thioesterase 1 OS=Mus musculus OX=10090 GN=Lypla1 PE=1 SV=1	MOUSE	4
1564	8.03	8.12	10.44	7.99	tr|Q3UMA3|Q3UMA3_MOUSE	Hepatocyte growth factor-regulated tyrosine kinase substrate OS=Mus musculus OX=10090 GN=Hgs PE=1 SV=1	MOUSE	5
1565	8.03	8.08	45.96	37.37	sp|P60904|DNJC5_MOUSE	DnaJ homolog subfamily C member 5 OS=Mus musculus OX=10090 GN=Dnajc5 PE=1 SV=1	MOUSE	5
1566	8.02	8.2	8.15	4.89	tr|Q91VT0|Q91VT0_MOUSE	DNA polymerase OS=Mus musculus OX=10090 GN=Pold1 PE=2 SV=1	MOUSE	6

1567	8.02	8.16	17.06	11.69	sp|Q8BNW9|KBTBB_MOUSE	Kelch repeat and BTB domain-containing protein 11 OS=Mus musculus OX=10090 GN=Kbtbd11 PE=1 SV=3	MOUSE	5
1568	8.02	8.03	52.41	33.13	tr|Q3TI65|Q3TI65_MOUSE	Mitochondrial 18 kDa protein OS=Mus musculus OX=10090 GN=Mtfp1 PE=2 SV=1	MOUSE	4
1569	8.02	8.02	38.07	32.99	sp|P46414|CDN1B_MOUSE	Cyclin-dependent kinase inhibitor 1B OS=Mus musculus OX=10090 GN=Cdkn1b PE=1 SV=2	MOUSE	4
1570	8.01	22.74	34.73	28.05	tr|E0CZ78|E0CZ78_MOUSE	Serine/threonine-protein phosphatase OS=Mus musculus OX=10090 GN=Ppp3cb PE=1 SV=1	MOUSE	13
1571	8.01	19.92	33.29	15.06	tr|Q91WP9|Q91WP9_MOUSE	Alpha-1,4 glucan phosphorylase OS=Mus musculus OX=10090 GN=Pygl PE=2 SV=1	MOUSE	11
1572	8.01	10.06	51.90	32.07	sp|Q9ES97-3|RTN3_MOUSE	Isoform 3 of Reticulon-3 OS=Mus musculus OX=10090 GN=Rtn3	MOUSE	5
1573	8	127.35	85.12	83.43	sp|P52480-2|KPYM_MOUSE	Isoform M1 of Pyruvate kinase PKM OS=Mus musculus OX=10090 GN=Pkm	MOUSE	118
1574	8	9.49	21.88	20.31	tr|Q69ZP5|Q69ZP5_MOUSE	MKIAA1181 protein (Fragment) OS=Mus musculus OX=10090 GN=Ergic1 PE=2 SV=1	MOUSE	5
1575	8	8.76	40.40	40.40	sp|Q9CQ75|NDUA2_MOUSE	NADH dehydrogenase [ubiquinone] 1 alpha subcomplex subunit 2 OS=Mus musculus OX=10090 GN=Ndufa2 PE=1 SV=3	MOUSE	5
1576	8	8	42.06	41.12	tr|Q566K0|Q566K0_MOUSE	High mobility group AT-hook protein 1 OS=Mus musculus OX=10090 GN=Hmga1 PE=2 SV=1	MOUSE	4
1577	8	8	27.85	25.32	tr|Q4VAI2|Q4VAI2_MOUSE	Low molecular weight cytosolic acid phosphatase OS=Mus musculus OX=10090 GN=Acp1 PE=2 SV=1	MOUSE	4
1578	8	8	46.67	46.67	tr|Q8C073|Q8C073_MOUSE	SH3 domain-binding glutamic acid-rich-like protein OS=Mus musculus OX=10090 GN=Sh3bgrl2 PE=2 SV=1	MOUSE	4
1579	8	8	38.35	38.35	tr|B2RY90|B2RY90_MOUSE	Isoc2a protein OS=Mus musculus OX=10090 GN=Isoc2a PE=2 SV=1	MOUSE	4
1580	8	8	54.62	54.62	sp|P62315|SMD1_MOUSE	Small nuclear ribonucleoprotein Sm D1 OS=Mus musculus OX=10090 GN=Snrpd1 PE=1 SV=1	MOUSE	8
1581	7.99	8.22	27.55	11.02	tr|Q3UHY8|Q3UHY8_MOUSE	Uncharacterized protein OS=Mus musculus OX=10090 GN=Abce1 PE=1 SV=1	MOUSE	5
1582	7.98	8.09	11.72	10.16	tr|Q3UQM7|Q3UQM7_MOUSE	Uncharacterized protein OS=Mus musculus OX=10090 GN=Slc7a5 PE=2 SV=1	MOUSE	4

1583	7.98	8.07	30.00	18.75	tr|Q3TMZ1|Q3TMZ1_MOUSE	Pyrroline-5-carboxylate reductase OS=Mus musculus OX=10090 GN=Pycr2 PE=1 SV=1	MOUSE	5
1584	7.98	8.02	30.71	17.72	tr|Q4FK54|Q4FK54_MOUSE	Proteaseome (Prosome, macropain) 28 subunit, 3 OS=Mus musculus OX=10090 GN=Psme3 PE=1 SV=1	MOUSE	5
1585	7.96	8.01	56.70	43.30	tr|A2AP32|A2AP32_MOUSE	Complex I-B17 OS=Mus musculus OX=10090 GN=Ndufb6 PE=1 SV=1	MOUSE	4
1586	7.94	8.02	58.22	58.22	sp|Q9CQX2|CYB5B_MOUSE	Cytochrome b5 type B OS=Mus musculus OX=10090 GN=Cyb5b PE=1 SV=1	MOUSE	5
1587	7.89	8.18	36.56	11.48	tr|Q8BKS6|Q8BKS6_MOUSE	Prolyl endopeptidase (Fragment) OS=Mus musculus OX=10090 GN=Prepl PE=2 SV=1	MOUSE	5
1588	7.88	8.03	16.84	11.58	tr|Q3THX4|Q3THX4_MOUSE	Uncharacterized protein OS=Mus musculus OX=10090 GN=Vps45 PE=2 SV=1	MOUSE	5
1589	7.87	8.04	43.77	19.15	sp|Q9WUP7|UCHL5_MOUSE	Ubiquitin carboxyl-terminal hydrolase isozyme L5 OS=Mus musculus OX=10090 GN=Uchl5 PE=1 SV=2	MOUSE	6
1590	7.86	7.93	15.45	10.45	tr|Q3TRK3|Q3TRK3_MOUSE	ADF-H domain-containing protein OS=Mus musculus OX=10090 GN=Dbn1 PE=2 SV=1	MOUSE	4
1591	7.86	7.9	50.93	41.67	sp|P26883|FKB1A_MOUSE	Peptidyl-prolyl cis-trans isomerase FKBP1A OS=Mus musculus OX=10090 GN=Fkbp1a PE=1 SV=2	MOUSE	4
1592	7.86	7.89	30.31	30.31	sp|Q9DCM0|ETHE1_MOUSE	Persulfide dioxygenase ETHE1, mitochondrial OS=Mus musculus OX=10090 GN=Ethe1 PE=1 SV=2	MOUSE	5
1593	7.83	8.07	13.02	7.56	tr|A0A1Y7VL44|A0A1Y7VL44_MOUSE	Tetratricopeptide repeat protein 7B OS=Mus musculus OX=10090 GN=Ttc7b PE=1 SV=1	MOUSE	6
1594	7.82	15.74	59.44	54.44	tr|Q14BR4|Q14BR4_MOUSE	ADP-ribosylation factor 4 OS=Mus musculus OX=10090 GN=Arf4 PE=1 SV=1	MOUSE	8
1595	7.82	8.05	31.01	19.71	tr|Q3UWH3|Q3UWH3_MOUSE	Uncharacterized protein OS=Mus musculus OX=10090 GN=Vrk1 PE=2 SV=1	MOUSE	5
1596	7.81	8.04	31.83	20.19	sp|Q9ESW4|AGK_MOUSE	Acylglycerol kinase, mitochondrial OS=Mus musculus OX=10090 GN=Agk PE=1 SV=1	MOUSE	5
1597	7.79	8.7	13.86	11.78	tr|Q8CCV5|Q8CCV5_MOUSE	Uncharacterized protein OS=Mus musculus OX=10090 GN=Esrrb PE=2 SV=1	MOUSE	5
1598	7.76	7.9	42.77	25.23	tr|Q3T9Y8|Q3T9Y8_MOUSE	Eukaryotic translation initiation factor 3 subunit I OS=Mus musculus OX=10090 GN=Eif3i PE=2 SV=1	MOUSE	5
1599	7.75	7.86	52.45	41.26	tr|G5E814|G5E814_MOUSE	Complex I-B14.7 OS=Mus musculus OX=10090 GN=Ndufa11 PE=1 SV=1	MOUSE	4

1600	7.74	7.84	27.01	14.44	tr|E9PWB2|E9PWB2_MOUSE	Casein kinase I isoform alpha OS=Mus musculus OX=10090 GN=Csnk1a1 PE=1 SV=1	MOUSE	4
1601	7.74	7.84	39.26	39.26	sp|P16045|LEG1_MOUSE	Galectin-1 OS=Mus musculus OX=10090 GN=Lgals1 PE=1 SV=3	MOUSE	4
1602	7.73	7.8	37.26	28.77	tr|Q8VC72|Q8VC72_MOUSE	Complex I-23kD OS=Mus musculus OX=10090 GN=Ndufs8 PE=2 SV=2	MOUSE	5
1603	7.71	7.76	24.54	17.84	tr|Q545Q2|Q545Q2_MOUSE	Surfeit locus protein 4 OS=Mus musculus OX=10090 GN=Surf4 PE=1 SV=1	MOUSE	4
1604	7.7	13.6	25.24	9.55	sp|P08553|NFM_MOUSE	Neurofilament medium polypeptide OS=Mus musculus OX=10090 GN=Nefm PE=1 SV=4	MOUSE	7
1605	7.7	13.1	40.17	39.74	tr|Q0PD56|Q0PD56_MOUSE	Rab5B OS=Mus musculus OX=10090 GN=Rab5b PE=2 SV=1	MOUSE	7
1606	7.68	7.9	20.64	9.65	sp|Q925I1|ATAD3_MOUSE	ATPase family AAA domain-containing protein 3 OS=Mus musculus OX=10090 GN=Atad3 PE=1 SV=1	MOUSE	5
1607	7.68	7.76	40.00	36.88	tr|Q9CQM8|Q9CQM8_MOUSE	60S ribosomal protein L21 OS=Mus musculus OX=10090 GN=Rpl21 PE=1 SV=1	MOUSE	7
1608	7.68	7.72	39.29	39.29	tr|Q3TND1|Q3TND1_MOUSE	Peptidylprolyl isomerase OS=Mus musculus OX=10090 GN=Fkbp2 PE=1 SV=1	MOUSE	4
1609	7.66	87.6	54.64	53.54	tr|A0A0A6YY91|A0A0A6YY91_MOUSE	Neural cell adhesion molecule 1 (Fragment) OS=Mus musculus OX=10090 GN=Ncam1 PE=1 SV=1	MOUSE	67
1610	7.66	7.8	20.38	14.50	sp|Q50H33|KCTD8_MOUSE	BTB/POZ domain-containing protein KCTD8 OS=Mus musculus OX=10090 GN=Kctd8 PE=1 SV=1	MOUSE	6
1611	7.66	7.74	31.77	18.06	tr|D3YU12|D3YU12_MOUSE	NmrA-like family domain-containing protein 1 OS=Mus musculus OX=10090 GN=Nmral1 PE=1 SV=1	MOUSE	4
1612	7.66	7.73	33.99	33.99	sp|Q9CPU4|MGST3_MOUSE	Microsomal glutathione S-transferase 3 OS=Mus musculus OX=10090 GN=Mgst3 PE=1 SV=1	MOUSE	4
1613	7.65	7.83	14.95	12.71	tr|Q8R3X7|Q8R3X7_MOUSE	Phosphoenolpyruvate carboxykinase (GTP) (Fragment) OS=Mus musculus OX=10090 GN=Pck2 PE=2 SV=1	MOUSE	5
1614	7.65	7.69	15.16	11.09	tr|Q9CY49|Q9CY49_MOUSE	WD_REPEATS_REGION domain-containing protein OS=Mus musculus OX=10090 GN=Tbl2 PE=2 SV=1	MOUSE	4
1615	7.64	7.75	36.68	20.46	tr|A0A068BEQ2|A0A068BEQ2_MOUSE	H2-K region protein 6 OS=Mus musculus OX=10090 GN=H2-Ke6 PE=2 SV=1	MOUSE	4
1616	7.63	7.75	50.00	41.55	tr|A7VJ98|A7VJ98_MOUSE	Glia maturation factor OS=Mus musculus OX=10090 GN=Gmfb PE=2 SV=1	MOUSE	5

1617	7.63	7.72	18.28	7.84	tr|Q8BH30|Q8BH30_MOUSE	Importin subunit alpha OS=Mus musculus OX=10090 GN=Kpna6 PE=1 SV=1	MOUSE	4
1618	7.62	7.79	59.91	42.73	sp|P06837|NEUM_MOUSE	Neuromodulin OS=Mus musculus OX=10090 GN=Gap43 PE=1 SV=1	MOUSE	5
1619	7.62	7.76	23.54	15.61	tr|Q3TCD4|Q3TCD4_MOUSE	Enoyl-CoA delta isomerase 2 OS=Mus musculus OX=10090 GN=Eci2 PE=1 SV=1	MOUSE	4
1620	7.61	9.32	10.07	3.95	tr|F8WGE3|F8WGE3_MOUSE	ABC-type organic anion transporter ABCA8A OS=Mus musculus OX=10090 GN=Abca8a PE=1 SV=1	MOUSE	5
1621	7.61	7.75	55.81	48.84	tr|Q497K3|Q497K3_MOUSE	Sm protein F OS=Mus musculus OX=10090 GN=Snrpf PE=1 SV=1	MOUSE	5
1622	7.59	7.83	46.10	23.05	sp|P47758|SRPRB_MOUSE	Signal recognition particle receptor subunit beta OS=Mus musculus OX=10090 GN=Srprb PE=1 SV=1	MOUSE	6
1623	7.59	7.71	49.16	36.87	tr|Q3TMX5|Q3TMX5_MOUSE	Mesencephalic astrocyte-derived neurotrophic factor OS=Mus musculus OX=10090 GN=Manf PE=1 SV=1	MOUSE	5
1624	7.59	7.64	40.69	40.69	tr|Q3UK70|Q3UK70_MOUSE	40S ribosomal protein S15 OS=Mus musculus OX=10090 GN=Rps15 PE=2 SV=1	MOUSE	5
1625	7.57	7.96	22.07	8.83	tr|Q543M7|Q543M7_MOUSE	Importin subunit alpha OS=Mus musculus OX=10090 GN=Kpna3 PE=1 SV=1	MOUSE	4
1626	7.56	7.62	10.60	7.34	sp|P70699|LYAG_MOUSE	Lysosomal alpha-glucosidase OS=Mus musculus OX=10090 GN=Gaa PE=1 SV=2	MOUSE	4
1627	7.51	7.57	48.15	37.04	sp|Q9CQB5|CISD2_MOUSE	CDGSH iron-sulfur domain-containing protein 2 OS=Mus musculus OX=10090 GN=Cisd2 PE=1 SV=1	MOUSE	5
1628	7.49	7.64	23.26	16.01	sp|Q9D1P4|CHRD1_MOUSE	Cysteine and histidine-rich domain-containing protein 1 OS=Mus musculus OX=10090 GN=Chordc1 PE=1 SV=1	MOUSE	4
1629	7.49	7.54	58.82	53.59	sp|Q9D898|ARP5L_MOUSE	Actin-related protein 2/3 complex subunit 5-like protein OS=Mus musculus OX=10090 GN=Arpc5l PE=1 SV=1	MOUSE	4
1630	7.47	7.57	16.06	11.50	tr|Q8BME1|Q8BME1_MOUSE	Phosphoacetylglucosamine mutase OS=Mus musculus OX=10090 GN=Pgm3 PE=2 SV=1	MOUSE	5
1631	7.46	7.63	43.61	38.35	tr|Q5EBJ0|Q5EBJ0_MOUSE	Fatty acid binding protein 3, muscle and heart OS=Mus musculus OX=10090 GN=Fabp3 PE=1 SV=1	MOUSE	5
1632	7.46	7.6	19.61	12.30	sp|Q3UUG6|TBC24_MOUSE	TBC1 domain family member 24 OS=Mus musculus OX=10090 GN=Tbc1d24 PE=1 SV=2	MOUSE	5
1633	7.46	7.6	25.67	22.09	tr|Q0P5W2|Q0P5W2_MOUSE	Thioredoxin domain containing 13 OS=Mus musculus OX=10090 GN=Tmx4 PE=1 SV=1	MOUSE	5

1634	7.46	7.49	33.33	33.33	tr|Q3URU9|Q3URU9_MOUSE	Retinal rod rhodopsin-sensitive cGMP 3',5'-cyclic phosphodiesterase subunit delta OS=Mus musculus OX=10090 GN=Pde6d PE=2 SV=1	MOUSE	4
1635	7.45	19.26	33.14	16.71	tr|Q8BJP2|Q8BJP2_MOUSE	Uncharacterized protein OS=Mus musculus OX=10090 GN=Ago1 PE=2 SV=1	MOUSE	9
1636	7.45	7.97	20.00	15.56	sp|Q9D3A9|TTYH1_MOUSE	Protein tweety homolog 1 OS=Mus musculus OX=10090 GN=Ttyh1 PE=1 SV=1	MOUSE	5
1637	7.43	7.67	31.94	14.54	sp|Q8BIW1|PRUN1_MOUSE	Exopolyphosphatase PRUNE1 OS=Mus musculus OX=10090 GN=Prune1 PE=1 SV=1	MOUSE	4
1638	7.43	7.52	33.90	24.58	sp|Q91VT4|CBR4_MOUSE	3-oxoacyl-[acyl-carrier-protein] reductase OS=Mus musculus OX=10090 GN=Cbr4 PE=1 SV=2	MOUSE	4
1639	7.42	7.6	27.69	10.74	tr|F6Y616|F6Y616_MOUSE	Polyadenylate-binding protein-interacting protein 1 OS=Mus musculus OX=10090 GN=Paip1 PE=1 SV=2	MOUSE	4
1640	7.41	7.58	23.61	14.81	tr|Q7TMY2|Q7TMY2_MOUSE	Acyl-Coenzyme A dehydrogenase, short/branched chain OS=Mus musculus OX=10090 GN=Acadsb PE=2 SV=1	MOUSE	5
1641	7.4	15.3	35.13	20.84	tr|Q3UUU4|Q3UUU4_MOUSE	Septin OS=Mus musculus OX=10090 GN=Septin6 PE=2 SV=1	MOUSE	10
1642	7.4	7.68	16.11	7.73	tr|Q5DTH1|Q5DTH1_MOUSE	MKIAA4216 protein (Fragment) OS=Mus musculus OX=10090 GN=Ube3a PE=2 SV=1	MOUSE	5
1643	7.38	8.15	13.04	7.97	sp|P63154|CRNL1_MOUSE	Crooked neck-like protein 1 OS=Mus musculus OX=10090 GN=Crnkl1 PE=1 SV=1	MOUSE	5
1644	7.38	7.93	28.40	13.23	tr|Q3UIH7|Q3UIH7_MOUSE	Geranylgeranyl transferase type-2 subunit alpha OS=Mus musculus OX=10090 GN=Rabggta PE=2 SV=1	MOUSE	6
1645	7.38	7.74	19.65	8.24	tr|A0A0R4J034|A0A0R4J034_MOUSE	Pyridoxal-dependent decarboxylase domain-containing protein 1 OS=Mus musculus OX=10090 GN=Pdxdc1 PE=1 SV=1	MOUSE	5
1646	7.38	7.56	10.54	6.96	sp|Q8CGF6|WDR47_MOUSE	WD repeat-containing protein 47 OS=Mus musculus OX=10090 GN=Wdr47 PE=1 SV=2	MOUSE	5
1647	7.37	7.56	45.97	27.01	sp|Q9JJU9|CRBB3_MOUSE	Beta-crystallin B3 OS=Mus musculus OX=10090 GN=Crybb3 PE=2 SV=3	MOUSE	4
1648	7.35	7.49	22.04	12.69	sp|Q9WU79|PROD_MOUSE	Proline dehydrogenase 1, mitochondrial OS=Mus musculus OX=10090 GN=Prodh PE=1 SV=2	MOUSE	4
1649	7.34	11.66	50.25	41.79	tr|Q3U0T9|Q3U0T9_MOUSE	Rab35 OS=Mus musculus OX=10090 GN=Rab35 PE=1 SV=1	MOUSE	6

1650	7.34	7.57	38.61	27.08	sp|Q9DCS3|MECR_MOUSE	Enoyl-[acyl-carrier-protein] reductase, mitochondrial OS=Mus musculus OX=10090 GN=Mecr PE=1 SV=2	MOUSE	6
1651	7.32	7.42	11.83	9.17	sp|Q9QY42|GPR37_MOUSE	Prosaposin receptor GPR37 OS=Mus musculus OX=10090 GN=Gpr37 PE=2 SV=1	MOUSE	4
1652	7.3	14.01	44.49	33.82	tr|Q8JZZ5|Q8JZZ5_MOUSE	Phosphatidylinositol transfer protein beta isoform OS=Mus musculus OX=10090 GN=Pitpnb PE=1 SV=1	MOUSE	8
1653	7.3	7.36	28.25	25.28	tr|Q3UGW4|Q3UGW4_MOUSE	ATP-dependent Clp protease proteolytic subunit (Fragment) OS=Mus musculus OX=10090 GN=Clpp PE=2 SV=1	MOUSE	6
1654	7.29	7.34	29.78	21.32	tr|Q8C2U7|Q8C2U7_MOUSE	tRNA-binding domain-containing protein OS=Mus musculus OX=10090 GN=Aimp1 PE=2 SV=1	MOUSE	4
1655	7.28	7.66	20.02	6.43	tr|A2AE27|A2AE27_MOUSE	AMP deaminase OS=Mus musculus OX=10090 GN=Ampd2 PE=1 SV=1	MOUSE	5
1656	7.28	7.43	14.66	9.09	tr|A0A0R4J124|A0A0R4J124_MOUSE	SRSF protein kinase 2 OS=Mus musculus OX=10090 GN=Srpk2 PE=1 SV=1	MOUSE	5
1657	7.28	7.34	9.40	9.40	tr|D3YXH0|D3YXH0_MOUSE	Predicted gene, 49948 OS=Mus musculus OX=10090 GN=Gm49948 PE=4 SV=1	MOUSE	4
1658	7.27	7.35	21.05	11.44	tr|Q3TDX2|Q3TDX2_MOUSE	Vesicle-fusing ATPase OS=Mus musculus OX=10090 GN=Vps4a PE=2 SV=1	MOUSE	4
1659	7.24	7.29	17.27	16.91	sp|Q8VBT0|TMX1_MOUSE	Thioredoxin-related transmembrane protein 1 OS=Mus musculus OX=10090 GN=Tmx1 PE=1 SV=1	MOUSE	6
1660	7.22	7.33	12.91	10.33	sp|Q9D824|FIP1_MOUSE	Pre-mRNA 3'-end-processing factor FIP1 OS=Mus musculus OX=10090 GN=Fip1l1 PE=1 SV=1	MOUSE	4
1661	7.2	7.31	37.45	35.74	sp|Q9JKD3|SCAM5_MOUSE	Secretory carrier-associated membrane protein 5 OS=Mus musculus OX=10090 GN=Scamp5 PE=1 SV=1	MOUSE	5
1662	7.2	7.27	28.16	19.18	tr|Q3U9U5|Q3U9U5_MOUSE	B-cell receptor-associated protein OS=Mus musculus OX=10090 GN=Bcap31 PE=2 SV=1	MOUSE	4
1663	7.17	7.58	48.71	30.26	sp|Q8BH58|TIPRL_MOUSE	TIP41-like protein OS=Mus musculus OX=10090 GN=Tiprl PE=1 SV=1	MOUSE	4
1664	7.16	7.29	48.90	48.35	tr|Q78IG7|Q78IG7_MOUSE	Transcription factor BTF3 (Fragment) OS=Mus musculus OX=10090 GN=Btf3l4 PE=2 SV=1	MOUSE	5
1665	7.14	7.42	27.16	12.95	tr|Q6PF96|Q6PF96_MOUSE	Electron transfer flavoprotein-ubiquinone oxidoreductase OS=Mus musculus OX=10090 GN=Etfdh PE=1 SV=1	MOUSE	6

1666	7.12	7.61	12.60	10.77	tr|Q561M4|Q561M4_MOUSE	Target of myb1 homolog (Chicken) OS=Mus musculus OX=10090 GN=Tom1 PE=2 SV=1	MOUSE	4
1667	7.11	7.18	33.99	21.57	sp|Q9D0L7|ARM10_MOUSE	Armadillo repeat-containing protein 10 OS=Mus musculus OX=10090 GN=Armc10 PE=1 SV=1	MOUSE	4
1668	7.09	10.6	14.95	12.20	sp|Q69ZK9|NLGN2_MOUSE	Neuroligin-2 OS=Mus musculus OX=10090 GN=Nlgn2 PE=1 SV=2	MOUSE	7
1669	7.09	7.26	23.49	16.55	tr|Q8BUU7|Q8BUU7_MOUSE	N-acetylgalactosamine kinase OS=Mus musculus OX=10090 GN=Galk2 PE=1 SV=1	MOUSE	4
1670	7.09	7.19	31.12	17.34	sp|Q91XU3|PI42C_MOUSE	Phosphatidylinositol 5-phosphate 4-kinase type-2 gamma OS=Mus musculus OX=10090 GN=Pip4k2c PE=1 SV=1	MOUSE	5
1671	7.09	7.14	8.35	4.25	tr|E9QKH8|E9QKH8_MOUSE	Catenin delta-2 OS=Mus musculus OX=10090 GN=Ctnnd2 PE=1 SV=1	MOUSE	4
1672	7.08	7.21	17.05	6.71	tr|A2AQR0|A2AQR0_MOUSE	Glycerol-3-phosphate dehydrogenase OS=Mus musculus OX=10090 GN=Gpd2 PE=1 SV=1	MOUSE	4
1673	7.08	7.14	42.28	30.87	tr|Q545B6|Q545B6_MOUSE	Stathmin OS=Mus musculus OX=10090 GN=Stmn1 PE=1 SV=1	MOUSE	4
1674	7.08	7.14	58.41	38.05	tr|A0A068BGR9|A0A068BGR9_MOUSE	Complex I-B14.5a OS=Mus musculus OX=10090 GN=Ndufa7 PE=2 SV=1	MOUSE	4
1675	7.07	7.22	18.20	11.92	sp|O35114|SCRB2_MOUSE	Lysosome membrane protein 2 OS=Mus musculus OX=10090 GN=Scarb2 PE=1 SV=3	MOUSE	4
1676	7.06	7.36	3.32	0.85	sp|Q6ZWR6|SYNE1_MOUSE	Nesprin-1 OS=Mus musculus OX=10090 GN=Syne1 PE=1 SV=2	MOUSE	5
1677	7.06	7.24	27.48	14.45	sp|Q9CX56|PSMD8_MOUSE	26S proteasome non-ATPase regulatory subunit 8 OS=Mus musculus OX=10090 GN=Psmd8 PE=1 SV=2	MOUSE	5
1678	7.05	7.45	18.39	13.84	tr|A2RS22|A2RS22_MOUSE	Coronin OS=Mus musculus OX=10090 GN=Coro1b PE=1 SV=1	MOUSE	4
1679	7.05	7.18	16.43	7.72	tr|B2RQS1|B2RQS1_MOUSE	Striatin-3 OS=Mus musculus OX=10090 GN=Strn3 PE=1 SV=1	MOUSE	4
1680	7	9.36	45.07	29.58	tr|Q4V9W8|Q4V9W8_MOUSE	Ras-related protein Rab-4 OS=Mus musculus OX=10090 GN=Rab4b PE=1 SV=1	MOUSE	5
1681	7	7.12	59.02	32.79	tr|Q9CPX4|Q9CPX4_MOUSE	Ferritin OS=Mus musculus OX=10090 GN=Ftl1 PE=1 SV=1	MOUSE	4
1682	6.99	17.81	69.23	38.46	tr|D3Z6I8|D3Z6I8_MOUSE	Tropomyosin alpha-3 chain OS=Mus musculus OX=10090 GN=Tpm3 PE=1 SV=1	MOUSE	9

1683	6.99	7.24	25.68	11.03	tr|Q3TVI6|Q3TVI6_MOUSE	Uncharacterized protein OS=Mus musculus OX=10090 GN=Appl2 PE=1 SV=1	MOUSE	5
1684	6.99	7.2	20.79	9.08	sp|Q922U1|PRPF3_MOUSE	U4/U6 small nuclear ribonucleoprotein Prp3 OS=Mus musculus OX=10090 GN=Prpf3 PE=1 SV=1	MOUSE	4
1685	6.96	12.06	7.91	5.15	tr|E9Q9E1|E9Q9E1_MOUSE	Eukaryotic translation initiation factor 4 gamma 1 OS=Mus musculus OX=10090 GN=Eif4g1 PE=1 SV=1	MOUSE	7
1686	6.96	8.81	16.84	8.59	sp|Q811G0|PTHB1_MOUSE	Protein PTHB1 OS=Mus musculus OX=10090 GN=Bbs9 PE=1 SV=2	MOUSE	6
1687	6.96	8.59	28.51	14.06	tr|D3Z710|D3Z710_MOUSE	Kinesin light chain OS=Mus musculus OX=10090 GN=Klc3 PE=1 SV=1	MOUSE	5
1688	6.96	7.03	58.31	20.38	tr|G3UY42|G3UY42_MOUSE	Polyadenylate-binding protein 2 OS=Mus musculus OX=10090 GN=Pabpn1 PE=1 SV=1	MOUSE	5
1689	6.95	7.12	13.82	11.32	tr|Q8BTJ0|Q8BTJ0_MOUSE	WD_REPEATS_REGION domain-containing protein OS=Mus musculus OX=10090 GN=Prpf4 PE=2 SV=1	MOUSE	4
1690	6.91	7.08	23.32	14.71	tr|Q543R4|Q543R4_MOUSE	Carboxypeptidase E OS=Mus musculus OX=10090 GN=Cpe PE=1 SV=1	MOUSE	4
1691	6.9	7.04	32.45	25.28	sp|O88587|COMT_MOUSE	Catechol O-methyltransferase OS=Mus musculus OX=10090 GN=Comt PE=1 SV=2	MOUSE	4
1692	6.9	6.95	44.93	40.58	tr|Q059I1|Q059I1_MOUSE	40S ribosomal protein S28 OS=Mus musculus OX=10090 GN=Rps28 PE=1 SV=1	MOUSE	4
1693	6.89	8.44	35.56	20.77	tr|Q5SVF7|Q5SVF7_MOUSE	Uncharacterized protein OS=Mus musculus OX=10090 GN=Nipsnap1 PE=1 SV=1	MOUSE	5
1694	6.88	7.03	11.67	9.73	sp|Q63943|MEF2D_MOUSE	Myocyte-specific enhancer factor 2D OS=Mus musculus OX=10090 GN=Mef2d PE=1 SV=2	MOUSE	4
1695	6.87	6.94	28.49	13.39	tr|Q3UJK6|Q3UJK6_MOUSE	NAD-dependent protein deacetylase OS=Mus musculus OX=10090 GN=Sirt2 PE=2 SV=1	MOUSE	3
1696	6.86	7.07	32.07	20.65	tr|Q9ESR1|Q9ESR1_MOUSE	Ras homolog OS=Mus musculus OX=10090 GN=Rheb PE=2 SV=1	MOUSE	4
1697	6.85	7.03	16.46	8.31	sp|Q8VCT3|AMPB_MOUSE	Aminopeptidase B OS=Mus musculus OX=10090 GN=Rnpep PE=1 SV=2	MOUSE	4
1698	6.84	7.08	40.15	26.28	tr|Q5M9N5|Q5M9N5_MOUSE	60S ribosomal protein L28 OS=Mus musculus OX=10090 GN=Rpl28 PE=2 SV=1	MOUSE	4
1699	6.82	7.54	52.50	31.00	tr|Q6ZWQ6|Q6ZWQ6_MOUSE	E2 ubiquitin-conjugating enzyme OS=Mus musculus OX=10090 GN=Ube2k PE=1 SV=1	MOUSE	5

1700	6.81	7.66	8.11	2.74	tr|Q6DFZ1|Q6DFZ1_MOUSE	Golgi-specific brefeldin A-resistance factor 1 OS=Mus musculus OX=10090 GN=Gbf1 PE=1 SV=1	MOUSE	4
1701	6.81	6.89	14.15	5.06	sp|Q80VP0|TCPR1_MOUSE	Tectonin beta-propeller repeat-containing protein 1 OS=Mus musculus OX=10090 GN=Tecpr1 PE=1 SV=1	MOUSE	3
1702	6.81	6.85	21.95	16.26	tr|A0A0R4J1Z3|A0A0R4J1Z3_MOUSE	Transmembrane protein 33 OS=Mus musculus OX=10090 GN=Tmem33 PE=1 SV=1	MOUSE	4
1703	6.8	11.37	27.17	11.75	tr|Q3U5L3|Q3U5L3_MOUSE	Sorting nexin-2 OS=Mus musculus OX=10090 GN=Snx2 PE=2 SV=1	MOUSE	6
1704	6.79	6.86	35.56	27.41	tr|Q5PR09|Q5PR09_MOUSE	Ribosomal protein L32 OS=Mus musculus OX=10090 GN=Rpl32 PE=2 SV=1	MOUSE	3
1705	6.77	6.93	34.93	24.89	sp|Q6IRU5|CLCB_MOUSE	Clathrin light chain B OS=Mus musculus OX=10090 GN=Cltb PE=1 SV=1	MOUSE	4
1706	6.74	6.95	15.53	7.70	sp|P52479|UBP10_MOUSE	Ubiquitin carboxyl-terminal hydrolase 10 OS=Mus musculus OX=10090 GN=Usp10 PE=1 SV=3	MOUSE	4
1707	6.73	6.93	19.50	13.93	sp|O70299|MB211_MOUSE	Putative nucleotidyltransferase MAB21L1 OS=Mus musculus OX=10090 GN=Mab21l1 PE=2 SV=1	MOUSE	5
1708	6.71	7	9.15	3.33	tr|G3UY75|G3UY75_MOUSE	Protein-tyrosine-phosphatase OS=Mus musculus OX=10090 GN=Ptprd PE=1 SV=1	MOUSE	5
1709	6.71	6.8	13.64	11.48	tr|A0A1W2P799|A0A1W2P799_MOUSE	Predicted gene, 49358 (Fragment) OS=Mus musculus OX=10090 GN=Gm49358 PE=3 SV=1	MOUSE	4
1710	6.7	12.52	63.26	35.81	sp|Q9CQD1|RAB5A_MOUSE	Ras-related protein Rab-5A OS=Mus musculus OX=10090 GN=Rab5a PE=1 SV=1	MOUSE	6
1711	6.7	6.83	8.63	6.32	sp|Q91XL9|OSBL1_MOUSE	Oxysterol-binding protein-related protein 1 OS=Mus musculus OX=10090 GN=Osbpl1a PE=1 SV=2	MOUSE	4
1712	6.67	6.81	20.78	9.04	sp|Q8VBZ3|CLPT1_MOUSE	Cleft lip and palate transmembrane protein 1 homolog OS=Mus musculus OX=10090 GN=Clptm1 PE=1 SV=1	MOUSE	5
1713	6.66	6.84	24.73	16.25	tr|A2AN37|A2AN37_MOUSE	Uridine-cytidine kinase OS=Mus musculus OX=10090 GN=Uck1 PE=1 SV=1	MOUSE	4
1714	6.66	6.8	24.34	17.76	sp|Q8BXZ1|TMX3_MOUSE	Protein disulfide-isomerase TMX3 OS=Mus musculus OX=10090 GN=Tmx3 PE=1 SV=2	MOUSE	5
1715	6.66	6.73	12.83	5.75	tr|Q8VED0|Q8VED0_MOUSE	Methylmalonyl-CoA isomerase OS=Mus musculus OX=10090 GN=Mmut PE=2 SV=1	MOUSE	5
1716	6.66	6.69	11.32	11.32	tr|Q4JFN6|Q4JFN6_MOUSE	NADH-ubiquinone oxidoreductase chain 1 OS=Mus musculus OX=10090 GN=mt-Nd1 PE=3 SV=1	MOUSE	4

1717	6.65	6.8	7.14	4.53	tr|Q3TGG2|Q3TGG2_MOUSE	Ataxin-2-like protein OS=Mus musculus OX=10090 GN=Atxn2l PE=1 SV=1	MOUSE	4
1718	6.65	6.76	42.26	20.50	sp|Q9WTP6|KAD2_MOUSE	Adenylate kinase 2, mitochondrial OS=Mus musculus OX=10090 GN=Ak2 PE=1 SV=5	MOUSE	4
1719	6.65	6.75	30.08	29.27	sp|Q6ZWV7|RL35_MOUSE	60S ribosomal protein L35 OS=Mus musculus OX=10090 GN=Rpl35 PE=1 SV=1	MOUSE	4
1720	6.65	6.71	7.56	3.07	sp|Q6PB44|PTN23_MOUSE	Tyrosine-protein phosphatase non-receptor type 23 OS=Mus musculus OX=10090 GN=Ptpn23 PE=1 SV=2	MOUSE	3
1721	6.65	6.69	18.08	11.83	tr|Q549A5|Q549A5_MOUSE	Clusterin OS=Mus musculus OX=10090 GN=Clu PE=1 SV=1	MOUSE	4
1722	6.64	6.67	17.65	13.00	tr|Q8C094|Q8C094_MOUSE	Mitogen-activated protein kinase (Fragment) OS=Mus musculus OX=10090 GN=Mapk9 PE=2 SV=1	MOUSE	4
1723	6.63	6.72	18.00	12.44	tr|Q6AXD3|Q6AXD3_MOUSE	Abi1 protein OS=Mus musculus OX=10090 GN=Abi1 PE=2 SV=1	MOUSE	4
1724	6.62	6.78	18.55	5.21	sp|Q9D2V7|CORO7_MOUSE	Coronin-7 OS=Mus musculus OX=10090 GN=Coro7 PE=1 SV=2	MOUSE	4
1725	6.62	6.73	26.01	12.38	tr|A0A0R4J0Z3|A0A0R4J0Z3_MOUSE	Aquaporin-4 OS=Mus musculus OX=10090 GN=Aqp4 PE=1 SV=1	MOUSE	4
1726	6.61	6.64	51.30	44.35	tr|Q497N1|Q497N1_MOUSE	40S ribosomal protein S26 OS=Mus musculus OX=10090 GN=Rps26 PE=1 SV=1	MOUSE	4
1727	6.6	9.21	21.58	7.00	sp|Q9QXK3|COPG2_MOUSE	Coatomer subunit gamma-2 OS=Mus musculus OX=10090 GN=Copg2 PE=1 SV=1	MOUSE	5
1728	6.6	8.99	28.32	19.08	sp|Q9R226|KHDR3_MOUSE	KH domain-containing, RNA-binding, signal transduction-associated protein 3 OS=Mus musculus OX=10090 GN=Khdrbs3 PE=1 SV=1	MOUSE	6
1729	6.6	8.65	15.42	11.30	tr|Q3TA75|Q3TA75_MOUSE	Uncharacterized protein (Fragment) OS=Mus musculus OX=10090 GN=Fxr2 PE=2 SV=1	MOUSE	5
1730	6.6	6.72	12.87	7.59	sp|Q8C8N2|SCAI_MOUSE	Protein SCAI OS=Mus musculus OX=10090 GN=Scai PE=1 SV=2	MOUSE	4
1731	6.59	6.68	10.34	6.57	tr|Q812D5|Q812D5_MOUSE	Lingerer protein-2a OS=Mus musculus OX=10090 GN=lig-2a PE=2 SV=1	MOUSE	4
1732	6.59	6.63	18.73	8.07	sp|Q8VD37|SGIP1_MOUSE	SH3-containing GRB2-like protein 3-interacting protein 1 OS=Mus musculus OX=10090 GN=Sgip1 PE=1 SV=1	MOUSE	3

1733	6.56	6.9	26.69	12.02	sp|Q06138|CAB39_MOUSE	Calcium-binding protein 39 OS=Mus musculus OX=10090 GN=Cab39 PE=1 SV=2	MOUSE	4
1734	6.56	6.79	55.00	42.50	tr|Q542U7|Q542U7_MOUSE	LSM6 homolog, U6 small nuclear RNA associated (S. cerevisiae) OS=Mus musculus OX=10090 GN=Lsm6 PE=1 SV=1	MOUSE	4
1735	6.56	6.65	31.17	23.38	tr|Q3TKG5|Q3TKG5_MOUSE	H/ACA ribonucleoprotein complex subunit OS=Mus musculus OX=10090 GN=Gar1 PE=2 SV=1	MOUSE	4
1736	6.53	6.61	22.01	19.31	tr|Q3TMV8|Q3TMV8_MOUSE	DCN1-like protein OS=Mus musculus OX=10090 PE=2 SV=1	MOUSE	4
1737	6.52	6.63	29.39	15.27	tr|Q545Q8|Q545Q8_MOUSE	Phosphomannomutase OS=Mus musculus OX=10090 GN=Pmm1 PE=1 SV=1	MOUSE	3
1738	6.51	6.89	20.43	6.94	tr|Q9D323|Q9D323_MOUSE	Signal transducer and activator of transcription OS=Mus musculus OX=10090 GN=Stat1 PE=2 SV=1	MOUSE	5
1739	6.51	6.54	36.02	21.43	sp|Q9D1M0|SEC13_MOUSE	Protein SEC13 homolog OS=Mus musculus OX=10090 GN=Sec13 PE=1 SV=3	MOUSE	6
1740	6.5	10.42	10.36	5.25	tr|E9QPI5|E9QPI5_MOUSE	Sister chromatid cohesion protein PDS5 homolog A OS=Mus musculus OX=10090 GN=Pds5a PE=1 SV=1	MOUSE	5
1741	6.5	6.6	17.77	12.65	tr|A2RTH5|A2RTH5_MOUSE	Leucine carboxyl methyltransferase 1 OS=Mus musculus OX=10090 GN=Lcmt1 PE=1 SV=1	MOUSE	4
1742	6.49	10.84	33.16	31.12	tr|Q4FJM5|Q4FJM5_MOUSE	Ras homolog gene family, member B OS=Mus musculus OX=10090 GN=Rhob PE=1 SV=1	MOUSE	6
1743	6.49	6.61	54.50	19.91	sp|Q8BWR2|PITH1_MOUSE	PITH domain-containing protein 1 OS=Mus musculus OX=10090 GN=Pithd1 PE=1 SV=1	MOUSE	4
1744	6.48	6.61	9.38	2.24	tr|A0A0U1RPD8|A0A0U1RPD8_MOUSE	General transcription factor 3C polypeptide 1 OS=Mus musculus OX=10090 GN=Gtf3c1 PE=1 SV=1	MOUSE	4
1745	6.47	6.5	28.91	17.01	sp|Q8BR90|CE051_MOUSE	UPF0600 protein C5orf51 homolog OS=Mus musculus OX=10090 PE=1 SV=1	MOUSE	4
1746	6.46	27.58	30.72	26.47	tr|F8WGT1|F8WGT1_MOUSE	Putative adenosylhomocysteinase 3 OS=Mus musculus OX=10090 GN=Ahcyl2 PE=1 SV=1	MOUSE	15
1747	6.46	6.52	20.62	15.59	tr|Q3UAP1|Q3UAP1_MOUSE	Uncharacterized protein OS=Mus musculus OX=10090 GN=Preb PE=1 SV=1	MOUSE	4
1748	6.46	6.49	16.52	10.13	tr|Q6HAA1|Q6HAA1_MOUSE	GA-binding protein alpha-subunit OS=Mus musculus OX=10090 GN=Gabpa PE=3 SV=1	MOUSE	3

1749	6.45	24.86	56.06	41.82	tr|Q3U8W0|Q3U8W0_MOUSE	Serine/threonine-protein phosphatase PP1-alpha catalytic subunit OS=Mus musculus OX=10090 GN=Ppp1ca PE=2 SV=1	MOUSE	13
1750	6.45	15.95	34.82	30.92	tr|Q3UPA1|Q3UPA1_MOUSE	Uncharacterized protein OS=Mus musculus OX=10090 GN=Gna11 PE=1 SV=1	MOUSE	9
1751	6.45	6.6	49.75	31.34	sp|P11352|GPX1_MOUSE	Glutathione peroxidase 1 OS=Mus musculus OX=10090 GN=Gpx1 PE=1 SV=2	MOUSE	4
1752	6.45	6.57	9.83	4.48	sp|Q61838|PZP_MOUSE	Pregnancy zone protein OS=Mus musculus OX=10090 GN=Pzp PE=1 SV=3	MOUSE	4
1753	6.45	6.55	14.74	11.85	sp|Q91VC9|GHITM_MOUSE	Growth hormone-inducible transmembrane protein OS=Mus musculus OX=10090 GN=Ghitm PE=1 SV=1	MOUSE	4
1754	6.44	6.56	22.73	12.78	tr|Q8BTX5|Q8BTX5_MOUSE	Eukaryotic translation initiation factor 3 subunit H OS=Mus musculus OX=10090 GN=Eif3h PE=2 SV=1	MOUSE	4
1755	6.44	6.47	20.06	14.45	tr|Q542N8|Q542N8_MOUSE	Uncharacterized protein OS=Mus musculus OX=10090 GN=Palm PE=2 SV=1	MOUSE	3
1756	6.44	6.47	17.13	11.53	tr|A0A2R8VK70|A0A2R8VK70_MOUSE	Integrin-associated protein OS=Mus musculus OX=10090 GN=Cd47 PE=1 SV=1	MOUSE	3
1757	6.43	6.76	19.35	12.20	sp|Q9CQJ4|RING2_MOUSE	E3 ubiquitin-protein ligase RING2 OS=Mus musculus OX=10090 GN=Rnf2 PE=1 SV=1	MOUSE	3
1758	6.42	6.62	18.43	10.24	tr|Q5SVF9|Q5SVF9_MOUSE	THO complex subunit 5 homolog OS=Mus musculus OX=10090 GN=Thoc5 PE=1 SV=1	MOUSE	4
1759	6.42	6.58	10.68	3.01	tr|E9Q166|E9Q166_MOUSE	ATPase family, AAA domain-containing 2B OS=Mus musculus OX=10090 GN=Atad2b PE=1 SV=1	MOUSE	4
1760	6.42	6.55	15.05	8.58	tr|Q8BP60|Q8BP60_MOUSE	Uncharacterized protein OS=Mus musculus OX=10090 GN=Nxf1 PE=2 SV=1	MOUSE	4
1761	6.42	6.53	5.99	3.91	tr|Q8CGC3|Q8CGC3_MOUSE	Hdac6 protein OS=Mus musculus OX=10090 GN=Hdac6 PE=1 SV=1	MOUSE	4
1762	6.41	6.44	72.92	60.42	sp|P62627|DLRB1_MOUSE	Dynein light chain roadblock-type 1 OS=Mus musculus OX=10090 GN=Dynlrb1 PE=1 SV=3	MOUSE	4
1763	6.38	6.67	32.58	11.46	tr|Q3UKV0|Q3UKV0_MOUSE	Eukaryotic translation initiation factor 2B, subunit 3 OS=Mus musculus OX=10090 GN=Eif2b3 PE=1 SV=1	MOUSE	4
1764	6.38	6.41	44.83	44.83	sp|Q78IK2|ATPMK_MOUSE	ATP synthase membrane subunit K, mitochondrial OS=Mus musculus OX=10090 GN=Atp5mk PE=1 SV=1	MOUSE	5

1765	6.38	6.4	24.47	20.74	sp|Q8R5J9|PRAF3_MOUSE	PRA1 family protein 3 OS=Mus musculus OX=10090 GN=Arl6ip5 PE=1 SV=2	MOUSE	3
1766	6.35	6.38	12.53	10.31	sp|O35599|OPSG_MOUSE	Medium-wave-sensitive opsin 1 OS=Mus musculus OX=10090 GN=Opn1mw PE=1 SV=1	MOUSE	3
1767	6.35	6.37	22.77	15.63	sp|Q9D8B3|CHM4B_MOUSE	Charged multivesicular body protein 4b OS=Mus musculus OX=10090 GN=Chmp4b PE=1 SV=2	MOUSE	3
1768	6.34	6.49	50.92	20.86	tr|Q3TL58|Q3TL58_MOUSE	S-phase kinase-associated protein 1 OS=Mus musculus OX=10090 GN=Skp1 PE=2 SV=1	MOUSE	4
1769	6.34	6.37	58.46	38.46	tr|G3UZY2|G3UZY2_MOUSE	Thioredoxin, mitochondrial (Fragment) OS=Mus musculus OX=10090 GN=Txn2 PE=1 SV=1	MOUSE	3
1770	6.33	6.36	39.46	34.69	tr|Q5M9K1|Q5M9K1_MOUSE	Transthyretin OS=Mus musculus OX=10090 GN=Ttr PE=1 SV=1	MOUSE	3
1771	6.33	6.35	14.16	9.09	tr|Q8C446|Q8C446_MOUSE	Gamma-aminobutyric acid receptor subunit beta-3 OS=Mus musculus OX=10090 GN=Gabrb3 PE=1 SV=1	MOUSE	3
1772	6.31	6.49	10.58	6.04	sp|Q8BIL5|HOOK1_MOUSE	Protein Hook homolog 1 OS=Mus musculus OX=10090 GN=Hook1 PE=1 SV=2	MOUSE	4
1773	6.3	21.18	13.65	9.98	sp|Q7TMB8|CYFP1_MOUSE	Cytoplasmic FMR1-interacting protein 1 OS=Mus musculus OX=10090 GN=Cyfip1 PE=1 SV=1	MOUSE	12
1774	6.3	6.37	18.10	16.38	sp|Q921W4|QORL1_MOUSE	Quinone oxidoreductase-like protein 1 OS=Mus musculus OX=10090 GN=Cryzl1 PE=1 SV=1	MOUSE	4
1775	6.29	6.47	19.16	16.22	tr|A0A0A6YVU8|A0A0A6YVU8_MOUSE	Predicted pseudogene 9774 OS=Mus musculus OX=10090 GN=Gm9774 PE=3 SV=1	MOUSE	5
1776	6.29	6.31	29.11	17.00	tr|A0A0A6YWB0|A0A0A6YWB0_MOUSE	Muscleblind-like protein 1 OS=Mus musculus OX=10090 GN=Mbnl1 PE=1 SV=1	MOUSE	3
1777	6.27	6.39	21.58	11.15	tr|A2AQL0|A2AQL0_MOUSE	Non-specific serine/threonine protein kinase OS=Mus musculus OX=10090 GN=Stk39 PE=1 SV=1	MOUSE	4
1778	6.27	6.37	22.61	17.31	tr|A0A0N4SVL9|A0A0N4SVL9_MOUSE	Serine/threonine-protein phosphatase OS=Mus musculus OX=10090 GN=Ppp6c PE=1 SV=1	MOUSE	4
1779	6.26	8.27	10.49	10.49	tr|Q99K73|Q99K73_MOUSE	Nrf1 protein OS=Mus musculus OX=10090 GN=Nrf1 PE=1 SV=1	MOUSE	5

1780	6.25	6.28	84.31	30.72	sp|Q9D1L0|CHCH2_MOUSE	Coiled-coil-helix-coiled-coil-helix domain-containing protein 2 OS=Mus musculus OX=10090 GN=Chchd2 PE=2 SV=1	MOUSE	5
1781	6.22	6.24	32.98	25.13	tr|Q8C5M4|Q8C5M4_MOUSE	Uncharacterized protein OS=Mus musculus OX=10090 GN=Pdcd6 PE=2 SV=1	MOUSE	3
1782	6.21	6.41	33.94	21.17	tr|Q544R1|Q544R1_MOUSE	Pyridoxal phosphate homeostasis protein OS=Mus musculus OX=10090 GN=Plpbp PE=1 SV=1	MOUSE	4
1783	6.21	6.3	20.91	16.38	tr|Q5D0A4|Q5D0A4_MOUSE	Stx1a protein (Fragment) OS=Mus musculus OX=10090 GN=Stx1a PE=2 SV=1	MOUSE	4
1784	6.2	6.22	13.96	10.47	tr|A0A0R4J0T5|A0A0R4J0T5_MOUSE	CUGBP Elav-like family member 1 OS=Mus musculus OX=10090 GN=Celf1 PE=1 SV=1	MOUSE	4
1785	6.2	6.22	21.54	14.46	tr|Q3UMP2|Q3UMP2_MOUSE	3-hydroxy-3-methylglutarate-CoA lyase OS=Mus musculus OX=10090 GN=Hmgcl PE=2 SV=1	MOUSE	3
1786	6.19	8.23	9.42	6.44	sp|P04104|K2C1_MOUSE	Keratin, type II cytoskeletal 1 OS=Mus musculus OX=10090 GN=Krt1 PE=1 SV=4	MOUSE	5
1787	6.19	6.26	20.82	20.48	tr|Q3TKQ3|Q3TKQ3_MOUSE	Ribosomal RNA-processing protein 4 OS=Mus musculus OX=10090 GN=Exosc2 PE=1 SV=1	MOUSE	5
1788	6.19	6.21	33.05	16.24	sp|Q9JKK7|TMOD2_MOUSE	Tropomodulin-2 OS=Mus musculus OX=10090 GN=Tmod2 PE=1 SV=2	MOUSE	3
1789	6.19	6.2	57.89	48.42	tr|A0A087WQE6|A0A087WQE6_MOUSE	Elongin-C (Fragment) OS=Mus musculus OX=10090 GN=Eloc PE=1 SV=1	MOUSE	3
1790	6.19	6.2	51.18	44.88	tr|Q3U8H8|Q3U8H8_MOUSE	NTF2 domain-containing protein OS=Mus musculus OX=10090 PE=2 SV=1	MOUSE	5
1791	6.18	8.46	13.53	10.42	tr|A0A087WQS2|A0A087WQS2_MOUSE	Basic leucine zipper and W2 domain-containing protein 1 OS=Mus musculus OX=10090 GN=Bzw1 PE=1 SV=1	MOUSE	5
1792	6.18	6.2	35.06	25.97	tr|Q8BG13|Q8BG13_MOUSE	RNA-binding protein 3 OS=Mus musculus OX=10090 GN=Rbm3 PE=1 SV=1	MOUSE	4
1793	6.17	6.31	11.13	5.70	tr|Q3UJA5|Q3UJA5_MOUSE	Uncharacterized protein OS=Mus musculus OX=10090 GN=Asap1 PE=2 SV=1	MOUSE	4
1794	6.16	6.17	39.65	26.87	sp|Q8R0F8|FAHD1_MOUSE	Acylpyruvase FAHD1, mitochondrial OS=Mus musculus OX=10090 GN=Fahd1 PE=1 SV=2	MOUSE	3
1795	6.15	6.36	9.38	4.83	tr|E9Q3K3|E9Q3K3_MOUSE	Arf-GAP with GTPase, ANK repeat and PH domain-containing protein 1 OS=Mus musculus OX=10090 GN=Agap1 PE=1 SV=3	MOUSE	4

1796	6.15	6.32	15.97	12.39	tr|E9QM06|E9QM06_MOUSE	Telomeric repeat-binding factor 2 OS=Mus musculus OX=10090 GN=Terf2 PE=1 SV=1	MOUSE	4
1797	6.14	6.2	11.45	4.42	tr|Q3TMP1|Q3TMP1_MOUSE	General transcription factor IIIC, polypeptide 3 OS=Mus musculus OX=10090 GN=Gtf3c3 PE=1 SV=1	MOUSE	3
1798	6.12	19.76	44.92	31.36	sp|B2RSH2|GNAI1_MOUSE	Guanine nucleotide-binding protein G(i) subunit alpha-1 OS=Mus musculus OX=10090 GN=Gnai1 PE=1 SV=1	MOUSE	12
1799	6.12	6.13	39.84	39.84	tr|Q4VAG4|Q4VAG4_MOUSE	Ribosomal protein L22 OS=Mus musculus OX=10090 GN=Rpl22 PE=1 SV=1	MOUSE	4
1800	6.11	6.29	38.04	23.91	sp|Q8C570|RAE1L_MOUSE	mRNA export factor OS=Mus musculus OX=10090 GN=Rae1 PE=1 SV=1	MOUSE	5
1801	6.11	6.22	42.15	29.12	sp|Q3UGC7|EI3JA_MOUSE	Eukaryotic translation initiation factor 3 subunit J-A OS=Mus musculus OX=10090 GN=Eif3j1 PE=2 SV=1	MOUSE	5
1802	6.1	6.27	22.40	12.30	tr|D3Z6W3|D3Z6W3_MOUSE	Retinol dehydrogenase OS=Mus musculus OX=10090 GN=Rdh8 PE=3 SV=1	MOUSE	4
1803	6.1	6.18	26.18	13.82	tr|Q9DB22|Q9DB22_MOUSE	RPOLD domain-containing protein OS=Mus musculus OX=10090 PE=2 SV=1	MOUSE	3
1804	6.1	6.13	20.96	7.69	tr|A6H669|A6H669_MOUSE	Bardet-Biedl syndrome 4 (Human) OS=Mus musculus OX=10090 GN=Bbs4 PE=2 SV=1	MOUSE	3
1805	6.08	6.29	22.49	22.49	tr|Q9CSK4|Q9CSK4_MOUSE	Uncharacterized protein (Fragment) OS=Mus musculus OX=10090 GN=Lrrc57 PE=2 SV=1	MOUSE	6
1806	6.08	6.09	17.31	12.14	tr|Q3U6V5|Q3U6V5_MOUSE	Eukaryotic peptide chain release factor subunit 1 OS=Mus musculus OX=10090 GN=Etf1 PE=2 SV=1	MOUSE	4
1807	6.08	6.09	35.37	25.85	tr|Q3V1C8|Q3V1C8_MOUSE	Bis(5'-nucleosyl)-tetraphosphatase [asymmetrical] OS=Mus musculus OX=10090 GN=Nudt2 PE=2 SV=1	MOUSE	3
1808	6.07	6.09	13.51	6.98	tr|Q9DBZ6|Q9DBZ6_MOUSE	Uncharacterized protein OS=Mus musculus OX=10090 GN=Abcd3 PE=2 SV=1	MOUSE	3
1809	6.06	8.86	40.93	31.61	sp|Q91X97|NCALD_MOUSE	Neurocalcin-delta OS=Mus musculus OX=10090 GN=Ncald PE=1 SV=4	MOUSE	7
1810	6.06	6.08	28.00	24.00	tr|Q58EA6|Q58EA6_MOUSE	40S ribosomal protein S25 OS=Mus musculus OX=10090 GN=Rps25 PE=1 SV=1	MOUSE	3
1811	6.06	6.07	55.39	33.82	sp|Q64152|BTF3_MOUSE	Transcription factor BTF3 OS=Mus musculus OX=10090 GN=Btf3 PE=1 SV=3	MOUSE	3

1812	6.06	6.07	20.94	15.94	sp|Q8R010|AIMP2_MOUSE	Aminoacyl tRNA synthase complex-interacting multifunctional protein 2 OS=Mus musculus OX=10090 GN=Aimp2 PE=1 SV=2	MOUSE	3
1813	6.05	6.15	69.57	59.13	sp|P99027|RLA2_MOUSE	60S acidic ribosomal protein P2 OS=Mus musculus OX=10090 GN=Rplp2 PE=1 SV=3	MOUSE	3
1814	6.05	6.14	43.48	19.32	tr|Q8C988|Q8C988_MOUSE	MPN domain-containing protein OS=Mus musculus OX=10090 PE=2 SV=1	MOUSE	3
1815	6.05	6.13	18.17	6.48	tr|D3YVV7|D3YVV7_MOUSE	NOVA alternative-splicing regulator 2 OS=Mus musculus OX=10090 GN=Nova2 PE=1 SV=3	MOUSE	3
1816	6.04	6.16	15.68	11.35	tr|Q9CQG4|Q9CQG4_MOUSE	Receptor expression-enhancing protein OS=Mus musculus OX=10090 GN=Reep5 PE=2 SV=1	MOUSE	4
1817	6.04	6.04	33.87	13.42	sp|Q9WTX2|PRKRA_MOUSE	Interferon-inducible double-stranded RNA-dependent protein kinase activator A OS=Mus musculus OX=10090 GN=Prkra PE=1 SV=1	MOUSE	3
1818	6.03	6.2	12.40	7.85	tr|Q3TEF1|Q3TEF1_MOUSE	Glutamate--cysteine ligase OS=Mus musculus OX=10090 GN=Gclc PE=1 SV=1	MOUSE	5
1819	6.02	43.84	71.35	60.48	tr|Q3TG92|Q3TG92_MOUSE	Uncharacterized protein OS=Mus musculus OX=10090 GN=Actc1 PE=2 SV=1	MOUSE	32
1820	6.02	6.07	25.70	17.32	tr|Q4FJR0|Q4FJR0_MOUSE	Nudt4 protein OS=Mus musculus OX=10090 GN=Nudt4 PE=1 SV=1	MOUSE	3
1821	6.02	6.02	20.33	20.33	tr|Q792Z1|Q792Z1_MOUSE	Trypsin 10 OS=Mus musculus OX=10090 GN=Try10 PE=1 SV=1	MOUSE	9
1822	6.02	6.02	15.80	14.39	sp|Q8QZY9|SF3B4_MOUSE	Splicing factor 3B subunit 4 OS=Mus musculus OX=10090 GN=Sf3b4 PE=1 SV=1	MOUSE	4
1823	6.01	6.74	39.89	39.33	sp|Q9CPT5|NOP16_MOUSE	Nucleolar protein 16 OS=Mus musculus OX=10090 GN=Nop16 PE=1 SV=1	MOUSE	5
1824	6.01	6.15	19.32	11.79	tr|Q548L4|Q548L4_MOUSE	Glutamate decarboxylase OS=Mus musculus OX=10090 GN=Gad2 PE=1 SV=1	MOUSE	4
1825	6.01	6.09	18.33	4.75	tr|B9EI57|B9EI57_MOUSE	SAFB-like, transcription modulator OS=Mus musculus OX=10090 GN=Sltm PE=2 SV=1	MOUSE	3
1826	6.01	6.02	47.06	31.62	tr|A0A1W2P729|A0A1W2P729_MOUSE	Protein canopy homolog 2 (Fragment) OS=Mus musculus OX=10090 GN=Cnpy2 PE=1 SV=1	MOUSE	4
1827	6.01	6.01	31.34	23.88	tr|Q4VAF0|Q4VAF0_MOUSE	Acylphosphatase (Fragment) OS=Mus musculus OX=10090 GN=Acyp1 PE=2 SV=1	MOUSE	3

1828	6.01	6.01	19.84	13.77	tr|Q8R0J2|Q8R0J2_MOUSE	Mannose-P-dolichol utilization defect 1 protein OS=Mus musculus OX=10090 GN=Mpdu1 PE=1 SV=1	MOUSE	3
1829	6.01	6.01	32.89	23.03	tr|Q3UG43|Q3UG43_MOUSE	Macro domain-containing protein OS=Mus musculus OX=10090 GN=Oard1 PE=2 SV=1	MOUSE	4
1830	6.01	6.01	23.50	19.50	tr|B1AZ39|B1AZ39_MOUSE	Chromatin modifying protein 6 OS=Mus musculus OX=10090 GN=Chmp6 PE=1 SV=1	MOUSE	3
1831	6.01	6.01	33.64	26.36	sp|P83870|PHF5A_MOUSE	PHD finger-like domain-containing protein 5A OS=Mus musculus OX=10090 GN=Phf5a PE=1 SV=1	MOUSE	3
1832	6.01	6.01	42.39	29.35	sp|P62305|RUXE_MOUSE	Small nuclear ribonucleoprotein E OS=Mus musculus OX=10090 GN=Snrpe PE=1 SV=1	MOUSE	4
1833	6	22.84	79.61	79.61	tr|Q5NC81|Q5NC81_MOUSE	Nucleoside diphosphate kinase OS=Mus musculus OX=10090 GN=Nme1 PE=1 SV=1	MOUSE	14
1834	6	10	35.00	35.00	sp|O55042|SYUA_MOUSE	Alpha-synuclein OS=Mus musculus OX=10090 GN=Snca PE=1 SV=2	MOUSE	5
1835	6	8.21	28.84	16.71	sp|Q9CYI4|LUC7L_MOUSE	Putative RNA-binding protein Luc7-like 1 OS=Mus musculus OX=10090 GN=Luc7l PE=1 SV=2	MOUSE	6
1836	6	6.03	17.23	13.51	tr|A2AEX8|A2AEX8_MOUSE	Four and a half LIM domains protein 1 OS=Mus musculus OX=10090 GN=Fhl1 PE=1 SV=1	MOUSE	3
1837	6	6	23.62	13.39	tr|Q9QYT9|Q9QYT9_MOUSE	Major prion protein OS=Mus musculus OX=10090 GN=Prnpb PE=3 SV=1	MOUSE	3
1838	6	6	34.48	34.48	tr|Q9CZI5|Q9CZI5_MOUSE	40S ribosomal protein S23 OS=Mus musculus OX=10090 GN=Rps23 PE=2 SV=1	MOUSE	4
1839	6	6	21.63	21.63	tr|Q7TQJ3|Q7TQJ3_MOUSE	Protein-ribulosamine 3-kinase OS=Mus musculus OX=10090 GN=Fn3k PE=1 SV=1	MOUSE	3
1840	6	6	27.54	27.54	tr|Q3UWG5|Q3UWG5_MOUSE	Tetraspanin OS=Mus musculus OX=10090 GN=Cd81 PE=2 SV=1	MOUSE	4
1841	6	6	22.47	22.47	sp|Q9JIG8|PRAF2_MOUSE	PRA1 family protein 2 OS=Mus musculus OX=10090 GN=Praf2 PE=1 SV=1	MOUSE	3
1842	6	6	40.46	40.46	sp|Q9CWW6|PIN4_MOUSE	Peptidyl-prolyl cis-trans isomerase NIMA-interacting 4 OS=Mus musculus OX=10090 GN=Pin4 PE=1 SV=1	MOUSE	3
1843	6	6	27.72	27.72	sp|P63166|SUMO1_MOUSE	Small ubiquitin-related modifier 1 OS=Mus musculus OX=10090 GN=Sumo1 PE=1 SV=1	MOUSE	3

1844	5.99	6.07	53.37	19.69	sp|Q66GT5|PTPM1_MOUSE	Phosphatidylglycerophosphatase and protein-tyrosine phosphatase 1 OS=Mus musculus OX=10090 GN=Ptpmt1 PE=1 SV=1	MOUSE	4
1845	5.98	25.29	61.54	48.95	sp|P27661|H2AX_MOUSE	Histone H2AX OS=Mus musculus OX=10090 GN=H2ax PE=1 SV=2	MOUSE	39
1846	5.98	6.08	39.13	15.65	sp|Q9JKB1|UCHL3_MOUSE	Ubiquitin carboxyl-terminal hydrolase isozyme L3 OS=Mus musculus OX=10090 GN=Uchl3 PE=1 SV=2	MOUSE	3
1847	5.98	6.07	15.19	10.34	tr|Q9D2D1|Q9D2D1_MOUSE	Carboxypeptidase OS=Mus musculus OX=10090 GN=Ctsa PE=2 SV=1	MOUSE	3
1848	5.98	6.04	9.38	4.61	tr|Q571B0|Q571B0_MOUSE	Transmembrane 9 superfamily member (Fragment) OS=Mus musculus OX=10090 GN=Tm9sf3 PE=2 SV=1	MOUSE	3
1849	5.97	6.04	18.14	14.64	tr|Q7TN25|Q7TN25_MOUSE	Sf3a2 protein OS=Mus musculus OX=10090 GN=Sf3a2 PE=2 SV=1	MOUSE	5
1850	5.95	19.43	36.57	23.64	tr|Q6ZWS7|Q6ZWS7_MOUSE	Calcium/calmodulin-dependent protein kinase OS=Mus musculus OX=10090 GN=Camk2g PE=2 SV=1	MOUSE	14
1851	5.95	6.05	24.93	11.76	sp|Q64442|DHSO_MOUSE	Sorbitol dehydrogenase OS=Mus musculus OX=10090 GN=Sord PE=1 SV=3	MOUSE	3
1852	5.95	6.04	21.46	21.46	sp|Q9D7S9|CHMP5_MOUSE	Charged multivesicular body protein 5 OS=Mus musculus OX=10090 GN=Chmp5 PE=1 SV=1	MOUSE	4
1853	5.94	6.02	12.84	8.21	tr|B9EHY2|B9EHY2_MOUSE	CAAX prenyl protease OS=Mus musculus OX=10090 GN=Zmpste24 PE=1 SV=1	MOUSE	3
1854	5.94	5.98	14.12	9.92	tr|Q3UD03|Q3UD03_MOUSE	CYTOSOL_AP domain-containing protein OS=Mus musculus OX=10090 GN=Npepl1 PE=2 SV=1	MOUSE	4
1855	5.93	5.97	61.73	55.56	tr|Q3UI46|Q3UI46_MOUSE	NEDD8 OS=Mus musculus OX=10090 GN=Nedd8 PE=1 SV=1	MOUSE	3
1856	5.92	6	38.99	30.82	tr|Q8C1Z9|Q8C1Z9_MOUSE	GOLD domain-containing protein OS=Mus musculus OX=10090 GN=Tmed2 PE=2 SV=1	MOUSE	3
1857	5.91	6.13	22.05	9.94	tr|Q5SWN2|Q5SWN2_MOUSE	Replication protein A subunit OS=Mus musculus OX=10090 GN=Rpa1 PE=1 SV=2	MOUSE	5
1858	5.91	6.01	21.83	15.63	tr|A0A158SIT3|A0A158SIT3_MOUSE	Ortholog of family with sequence similarity 50, member A FAM50A (Homo sapiens) OS=Mus musculus OX=10090 GN=Fam50a PE=4 SV=1	MOUSE	4
1859	5.91	6	36.89	21.78	sp|Q99LS3|SERB_MOUSE	Phosphoserine phosphatase OS=Mus musculus OX=10090 GN=Psph PE=1 SV=1	MOUSE	3

1860	5.9	6.03	39.53	39.53	sp|P62488|RPB7_MOUSE	DNA-directed RNA polymerase II subunit RPB7 OS=Mus musculus OX=10090 GN=Polr2g PE=1 SV=1	MOUSE	5
1861	5.89	5.96	23.26	23.26	sp|P63028|TCTP_MOUSE	Translationally-controlled tumor protein OS=Mus musculus OX=10090 GN=Tpt1 PE=1 SV=1	MOUSE	3
1862	5.87	6.09	19.09	12.40	sp|Q3THK3|T2FA_MOUSE	General transcription factor IIF subunit 1 OS=Mus musculus OX=10090 GN=Gtf2f1 PE=1 SV=2	MOUSE	5
1863	5.87	6.02	44.55	44.55	tr|F7CTF8|F7CTF8_MOUSE	Mitochondrial fission factor (Fragment) OS=Mus musculus OX=10090 GN=Mff PE=1 SV=1	MOUSE	4
1864	5.86	6.16	17.42	10.48	sp|Q7TSV4|PGM2_MOUSE	Phosphoglucomutase-2 OS=Mus musculus OX=10090 GN=Pgm2 PE=1 SV=1	MOUSE	5
1865	5.86	6	11.31	3.66	tr|Z4YJU8|Z4YJU8_MOUSE	Golgin subfamily A member 2 OS=Mus musculus OX=10090 GN=Golga2 PE=1 SV=3	MOUSE	3
1866	5.86	5.93	43.81	32.38	sp|P10639|THIO_MOUSE	Thioredoxin OS=Mus musculus OX=10090 GN=Txn PE=1 SV=3	MOUSE	3
1867	5.86	5.89	24.43	12.05	tr|Q9CWQ7|Q9CWQ7_MOUSE	Uncharacterized protein OS=Mus musculus OX=10090 GN=Ufd1 PE=2 SV=1	MOUSE	3
1868	5.85	8	35.60	28.27	sp|Q61686|CBX5_MOUSE	Chromobox protein homolog 5 OS=Mus musculus OX=10090 GN=Cbx5 PE=1 SV=1	MOUSE	4
1869	5.85	5.94	13.54	5.25	tr|A0A0R4J087|A0A0R4J087_MOUSE	Transporter OS=Mus musculus OX=10090 GN=Slc6a17 PE=1 SV=1	MOUSE	3
1870	5.85	5.93	56.04	48.35	tr|B1ARW4|B1ARW4_MOUSE	Complex I-15 kDa (Fragment) OS=Mus musculus OX=10090 GN=Ndufs5 PE=1 SV=1	MOUSE	4
1871	5.85	5.89	19.66	19.66	sp|Q9CXW4|RL11_MOUSE	60S ribosomal protein L11 OS=Mus musculus OX=10090 GN=Rpl11 PE=1 SV=4	MOUSE	3
1872	5.84	5.96	44.97	17.46	tr|Q3TP62|Q3TP62_MOUSE	PH domain-containing protein OS=Mus musculus OX=10090 GN=Plekhb1 PE=2 SV=1	MOUSE	3
1873	5.84	5.9	13.23	10.31	tr|Q3U3M5|Q3U3M5_MOUSE	Cyclin-K OS=Mus musculus OX=10090 GN=Ccnk PE=1 SV=1	MOUSE	3
1874	5.82	5.89	13.82	11.64	sp|Q8BFY6|PEF1_MOUSE	Peflin OS=Mus musculus OX=10090 GN=Pef1 PE=1 SV=1	MOUSE	3
1875	5.81	5.95	7.85	3.57	sp|Q9Z0W3|NU160_MOUSE	Nuclear pore complex protein Nup160 OS=Mus musculus OX=10090 GN=Nup160 PE=1 SV=2	MOUSE	3
1876	5.8	5.88	13.32	8.03	sp|Q91YL3|UCKL1_MOUSE	Uridine-cytidine kinase-like 1 OS=Mus musculus OX=10090 GN=Uckl1 PE=1 SV=1	MOUSE	3

1877	5.79	9.83	31.45	22.58	tr|Q3UW32|Q3UW32_MOUSE	LRRcap domain-containing protein (Fragment) OS=Mus musculus OX=10090 GN=Anp32b PE=2 SV=1	MOUSE	6
1878	5.78	6.01	16.01	9.80	sp|Q9DC50|OCTC_MOUSE	Peroxisomal carnitine O-octanoyltransferase OS=Mus musculus OX=10090 GN=Crot PE=1 SV=1	MOUSE	4
1879	5.78	5.88	34.45	28.57	tr|Q5BLK2|Q5BLK2_MOUSE	40S ribosomal protein S20 OS=Mus musculus OX=10090 GN=Rps20 PE=1 SV=1	MOUSE	4
1880	5.78	5.87	19.57	12.04	tr|Q3UL54|Q3UL54_MOUSE	Uncharacterized protein OS=Mus musculus OX=10090 GN=Rsl1d1 PE=2 SV=1	MOUSE	3
1881	5.75	5.91	9.69	2.15	tr|A0A1B0GSX7|A0A1B0GSX7_MOUSE	Nuclear pore complex protein Nup96 OS=Mus musculus OX=10090 GN=Nup98 PE=1 SV=1	MOUSE	3
1882	5.75	5.89	51.15	37.40	tr|Q4VAE8|Q4VAE8_MOUSE	Complex I-B15 (Fragment) OS=Mus musculus OX=10090 GN=Ndufb4 PE=2 SV=1	MOUSE	3
1883	5.75	5.83	27.13	26.60	tr|Q80ZM5|Q80ZM5_MOUSE	H1 histone family, member X OS=Mus musculus OX=10090 GN=H1f10 PE=1 SV=1	MOUSE	6
1884	5.74	6.08	8.43	4.68	tr|F8VPK5|F8VPK5_MOUSE	Rho-associated protein kinase OS=Mus musculus OX=10090 GN=Rock2 PE=1 SV=1	MOUSE	5
1885	5.73	5.95	10.06	4.97	tr|G3X8Y3|G3X8Y3_MOUSE	N-alpha-acetyltransferase 15, NatA auxiliary subunit OS=Mus musculus OX=10090 GN=Naa15 PE=1 SV=1	MOUSE	5
1886	5.72	7.82	36.21	26.44	tr|A2RTH4|A2RTH4_MOUSE	Crystallin, gamma E OS=Mus musculus OX=10090 GN=Cryge PE=2 SV=1	MOUSE	5
1887	5.72	5.88	12.04	4.95	tr|Q6A0C5|Q6A0C5_MOUSE	MKIAA0126 protein (Fragment) OS=Mus musculus OX=10090 GN=Ube4a PE=2 SV=1	MOUSE	3
1888	5.72	5.88	32.45	20.06	sp|Q8BTG7-2|NDRG4_MOUSE	Isoform 2 of Protein NDRG4 OS=Mus musculus OX=10090 GN=Ndrg4	MOUSE	5
1889	5.72	5.86	8.31	3.99	tr|G5E863|G5E863_MOUSE	Glutamate receptor OS=Mus musculus OX=10090 GN=Gria4 PE=1 SV=1	MOUSE	3
1890	5.71	5.95	13.62	7.77	sp|Q8BX80|ENASE_MOUSE	Cytosolic endo-beta-N-acetylglucosaminidase OS=Mus musculus OX=10090 GN=Engase PE=1 SV=1	MOUSE	5
1891	5.71	5.86	20.40	13.79	tr|Q3TXY3|Q3TXY3_MOUSE	Uncharacterized protein OS=Mus musculus OX=10090 GN=Tmem201 PE=2 SV=1	MOUSE	4
1892	5.71	5.82	37.21	30.81	tr|E9Q317|E9Q317_MOUSE	18 kDa Sin3-associated polypeptide OS=Mus musculus OX=10090 GN=Sap18 PE=1 SV=1	MOUSE	4
1893	5.71	5.77	17.69	9.13	tr|A0A087WSR7|A0A087WSR7_MOUSE	Arf-GAP domain and FG repeat-containing protein 1 OS=Mus musculus OX=10090 GN=Agfg1 PE=1 SV=1	MOUSE	3

1894	5.7	7.97	17.42	11.69	sp|O70572|NSMA_MOUSE	Sphingomyelin phosphodiesterase 2 OS=Mus musculus OX=10090 GN=Smpd2 PE=1 SV=1	MOUSE	5
1895	5.7	5.77	14.65	14.65	sp|Q9JJE4|PAQR4_MOUSE	Progestin and adipoQ receptor family member 4 OS=Mus musculus OX=10090 GN=Paqr4 PE=2 SV=1	MOUSE	3
1896	5.69	5.83	35.78	16.51	tr|F6VG18|F6VG18_MOUSE	Leucine zipper transcription factor-like protein 1 (Fragment) OS=Mus musculus OX=10090 GN=Lztfl1 PE=1 SV=1	MOUSE	3
1897	5.69	5.8	25.93	16.20	tr|A0A0R4J069|A0A0R4J069_MOUSE	Selenocysteine lyase OS=Mus musculus OX=10090 GN=Scly PE=1 SV=1	MOUSE	4
1898	5.68	5.86	38.92	25.75	tr|Q9CWA5|Q9CWA5_MOUSE	Nucleoside diphosphate kinase (Fragment) OS=Mus musculus OX=10090 GN=Nme3 PE=2 SV=1	MOUSE	4
1899	5.68	5.86	18.42	18.42	sp|Q8VD33|SGTB_MOUSE	Small glutamine-rich tetratricopeptide repeat-containing protein beta OS=Mus musculus OX=10090 GN=Sgtb PE=1 SV=1	MOUSE	4
1900	5.68	5.74	14.86	14.86	sp|Q922B1|MACD1_MOUSE	ADP-ribose glycohydrolase MACROD1 OS=Mus musculus OX=10090 GN=Macrod1 PE=1 SV=2	MOUSE	3
1901	5.67	14.08	17.04	6.22	tr|E9QNY9|E9QNY9_MOUSE	CLIP-associating protein 1 OS=Mus musculus OX=10090 GN=Clasp1 PE=1 SV=2	MOUSE	7
1902	5.66	12.64	36.06	30.29	tr|Q7M6W1|Q7M6W1_MOUSE	Reticulon OS=Mus musculus OX=10090 GN=Rtn1 PE=1 SV=1	MOUSE	7
1903	5.66	5.76	18.37	11.48	tr|Q80UL3|Q80UL3_MOUSE	Galactokinase 1 OS=Mus musculus OX=10090 GN=Galk1 PE=2 SV=1	MOUSE	4
1904	5.66	5.72	13.42	10.74	sp|Q8VD62|CK068_MOUSE	UPF0696 protein C11orf68 homolog OS=Mus musculus OX=10090 GN=Bles03 PE=1 SV=2	MOUSE	3
1905	5.65	5.72	10.54	4.82	tr|Q27PY9|Q27PY9_MOUSE	Na(+)/Ca(2+)-exchange protein 1 OS=Mus musculus OX=10090 GN=Slc8a1 PE=2 SV=1	MOUSE	3
1906	5.64	5.86	43.58	18.72	sp|Q9DBH5|LMAN2_MOUSE	Vesicular integral-membrane protein VIP36 OS=Mus musculus OX=10090 GN=Lman2 PE=1 SV=2	MOUSE	4
1907	5.64	5.74	58.95	42.11	sp|P62075|TIM13_MOUSE	Mitochondrial import inner membrane translocase subunit Tim13 OS=Mus musculus OX=10090 GN=Timm13 PE=1 SV=1	MOUSE	3
1908	5.64	5.72	46.49	35.96	tr|Q3U9A8|Q3U9A8_MOUSE	SH3 domain-binding glutamic acid-rich-like protein OS=Mus musculus OX=10090 GN=Sh3bgrl PE=2 SV=1	MOUSE	3

1909	5.64	5.7	19.08	9.66	sp|Q9Z2E1|MBD2_MOUSE	Methyl-CpG-binding domain protein 2 OS=Mus musculus OX=10090 GN=Mbd2 PE=2 SV=2	MOUSE	3
1910	5.63	9.03	14.53	9.54	sp|P98191|CDS1_MOUSE	Phosphatidate cytidylyltransferase 1 OS=Mus musculus OX=10090 GN=Cds1 PE=1 SV=2	MOUSE	5
1911	5.63	5.76	21.59	21.59	sp|Q6TEK5|VKORL_MOUSE	Vitamin K epoxide reductase complex subunit 1-like protein 1 OS=Mus musculus OX=10090 GN=Vkorc1l1 PE=1 SV=1	MOUSE	4
1912	5.63	5.69	26.70	14.44	tr|Q3USC7|Q3USC7_MOUSE	Ganglioside-induced differentiation-associated protein 1-like 1 OS=Mus musculus OX=10090 GN=Gdap1l1 PE=1 SV=1	MOUSE	3
1913	5.62	5.95	19.77	9.45	tr|E9PW14|E9PW14_MOUSE	Nuclear protein MDM1 OS=Mus musculus OX=10090 GN=Mdm1 PE=1 SV=1	MOUSE	5
1914	5.61	6.23	26.73	17.49	tr|A2RS58|A2RS58_MOUSE	V-crk sarcoma virus CT10 oncogene homolog (Avian)-like OS=Mus musculus OX=10090 GN=Crkl PE=1 SV=1	MOUSE	5
1915	5.61	5.88	6.80	4.68	tr|E9QKA4|E9QKA4_MOUSE	Serine/arginine repetitive matrix protein 1 OS=Mus musculus OX=10090 GN=Srrm1 PE=1 SV=1	MOUSE	3
1916	5.61	5.76	27.00	13.50	tr|Q8K295|Q8K295_MOUSE	Lrpap1 protein (Fragment) OS=Mus musculus OX=10090 GN=Lrpap1 PE=2 SV=1	MOUSE	4
1917	5.6	5.92	14.20	4.84	tr|Q3U2V0|Q3U2V0_MOUSE	Uncharacterized protein OS=Mus musculus OX=10090 GN=Sympk PE=2 SV=1	MOUSE	5
1918	5.6	5.68	8.71	4.71	tr|B2KFW6|B2KFW6_MOUSE	Polyhomeotic-like 2 (Drosophila) OS=Mus musculus OX=10090 GN=Phc2 PE=4 SV=1	MOUSE	3
1919	5.59	5.64	21.10	13.31	tr|Q0VGN4|Q0VGN4_MOUSE	Stx16 protein (Fragment) OS=Mus musculus OX=10090 GN=Stx16 PE=2 SV=1	MOUSE	3
1920	5.58	9.86	49.30	27.70	tr|Q0PD14|Q0PD14_MOUSE	Rab39B OS=Mus musculus OX=10090 GN=Rab39b PE=1 SV=1	MOUSE	5
1921	5.58	5.73	40.37	39.45	tr|Q3UX28|Q3UX28_MOUSE	Mitochondrial pyruvate carrier OS=Mus musculus OX=10090 GN=Mpc1 PE=1 SV=1	MOUSE	4
1922	5.58	5.7	24.18	23.53	sp|Q9CRB2|NHP2_MOUSE	H/ACA ribonucleoprotein complex subunit 2 OS=Mus musculus OX=10090 GN=Nhp2 PE=1 SV=1	MOUSE	3
1923	5.57	5.82	15.80	10.92	sp|Q921T2|TOIP1_MOUSE	Torsin-1A-interacting protein 1 OS=Mus musculus OX=10090 GN=Tor1aip1 PE=1 SV=3	MOUSE	4
1924	5.57	5.8	19.72	19.72	sp|Q9DBZ5|EIF3K_MOUSE	Eukaryotic translation initiation factor 3 subunit K OS=Mus musculus OX=10090 GN=Eif3k PE=1 SV=1	MOUSE	4

1925	5.57	5.68	17.66	12.73	tr|A0A0R4J1H4|A0A0R4J1H4_MOUSE	Nuclear receptor ROR-beta OS=Mus musculus OX=10090 GN=Rorb PE=1 SV=1	MOUSE	5
1926	5.57	5.64	11.65	8.19	tr|A0A0R4J030|A0A0R4J030_MOUSE	APC membrane recruitment protein 2 OS=Mus musculus OX=10090 GN=Amer2 PE=1 SV=1	MOUSE	3
1927	5.56	14.61	22.79	19.51	tr|Q80TN1|Q80TN1_MOUSE	Calcium/calmodulin-dependent protein kinase (Fragment) OS=Mus musculus OX=10090 GN=Camk2a PE=2 SV=3	MOUSE	12
1928	5.52	5.66	35.69	10.29	sp|Q8JZU2|TXTP_MOUSE	Tricarboxylate transport protein, mitochondrial OS=Mus musculus OX=10090 GN=Slc25a1 PE=1 SV=1	MOUSE	3
1929	5.52	5.62	8.54	5.49	sp|Q8R480|NUP85_MOUSE	Nuclear pore complex protein Nup85 OS=Mus musculus OX=10090 GN=Nup85 PE=1 SV=1	MOUSE	3
1930	5.5	5.73	12.55	3.92	tr|Q80ZX0|Q80ZX0_MOUSE	Sec24-related gene family, member B (S. cerevisiae) OS=Mus musculus OX=10090 GN=Sec24b PE=1 SV=1	MOUSE	3
1931	5.5	5.73	13.28	5.53	sp|Q80SY5|PR38B_MOUSE	Pre-mRNA-splicing factor 38B OS=Mus musculus OX=10090 GN=Prpf38b PE=1 SV=1	MOUSE	3
1932	5.5	5.64	6.53	2.85	tr|A4FUW1|A4FUW1_MOUSE	4932417H02Rik protein (Fragment) OS=Mus musculus OX=10090 GN=Rptor PE=2 SV=1	MOUSE	3
1933	5.49	5.65	9.89	6.89	sp|Q71M36|CSPG5_MOUSE	Chondroitin sulfate proteoglycan 5 OS=Mus musculus OX=10090 GN=Cspg5 PE=1 SV=2	MOUSE	3
1934	5.49	5.56	48.21	46.43	sp|P62274|RS29_MOUSE	40S ribosomal protein S29 OS=Mus musculus OX=10090 GN=Rps29 PE=1 SV=2	MOUSE	3
1935	5.48	5.73	8.42	2.83	sp|Q6PDI5|ECM29_MOUSE	Proteasome adapter and scaffold protein ECM29 OS=Mus musculus OX=10090 GN=Ecpas PE=1 SV=3	MOUSE	3
1936	5.48	5.62	5.81	2.43	sp|Q60520|SIN3A_MOUSE	Paired amphipathic helix protein Sin3a OS=Mus musculus OX=10090 GN=Sin3a PE=1 SV=3	MOUSE	3
1937	5.47	5.62	11.89	6.06	tr|E9QK48|E9QK48_MOUSE	Echinoderm microtubule-associated protein-like 2 OS=Mus musculus OX=10090 GN=Eml2 PE=1 SV=1	MOUSE	4
1938	5.46	7.25	21.97	8.65	sp|Q3U186|SYRM_MOUSE	Probable arginine--tRNA ligase, mitochondrial OS=Mus musculus OX=10090 GN=Rars2 PE=1 SV=1	MOUSE	4
1939	5.46	5.62	25.46	22.88	sp|Q8VHC5|CABP4_MOUSE	Calcium-binding protein 4 OS=Mus musculus OX=10090 GN=Cabp4 PE=1 SV=1	MOUSE	4
1940	5.46	5.56	42.31	42.31	sp|Q62048|PEA15_MOUSE	Astrocytic phosphoprotein PEA-15 OS=Mus musculus OX=10090 GN=Pea15 PE=1 SV=1	MOUSE	4

1941	5.44	5.65	84.87	68.07	sp|Q8R404|MIC13_MOUSE	MICOS complex subunit MIC13 OS=Mus musculus OX=10090 GN=Micos13 PE=1 SV=1	MOUSE	4
1942	5.44	5.54	36.59	26.83	sp|Q9CQ69|QCR8_MOUSE	Cytochrome b-c1 complex subunit 8 OS=Mus musculus OX=10090 GN=Uqcrq PE=1 SV=3	MOUSE	3
1943	5.44	5.53	19.46	10.86	sp|Q99PL6|UBXN6_MOUSE	UBX domain-containing protein 6 OS=Mus musculus OX=10090 GN=Ubxn6 PE=1 SV=1	MOUSE	4
1944	5.43	10.62	34.66	26.71	sp|Q8K354|CBR3_MOUSE	Carbonyl reductase [NADPH] 3 OS=Mus musculus OX=10090 GN=Cbr3 PE=1 SV=1	MOUSE	6
1945	5.42	7.77	22.45	8.93	tr|Q3TDT0|Q3TDT0_MOUSE	Tripartite motif-containing protein 3 OS=Mus musculus OX=10090 GN=Trim3 PE=1 SV=1	MOUSE	5
1946	5.42	5.48	17.80	17.80	tr|Q3U6D7|Q3U6D7_MOUSE	MARVEL domain-containing protein OS=Mus musculus OX=10090 GN=Syngr1 PE=2 SV=1	MOUSE	3
1947	5.41	5.48	58.75	58.75	tr|V9GXV0|V9GXV0_MOUSE	Isocitrate dehydrogenase 3 (NAD+) beta (Fragment) OS=Mus musculus OX=10090 GN=Idh3b PE=1 SV=1	MOUSE	5
1948	5.4	32.66	25.06	15.86	tr|A2ALL9|A2ALL9_MOUSE	Calcium-transporting ATPase OS=Mus musculus OX=10090 GN=Atp2b3 PE=1 SV=1	MOUSE	18
1949	5.4	5.54	14.43	5.90	tr|Q921M8|Q921M8_MOUSE	Ubiquitin carboxyl-terminal hydrolase OS=Mus musculus OX=10090 GN=Usp4 PE=2 SV=1	MOUSE	4
1950	5.39	5.57	26.01	9.12	tr|A0A1L1SS90|A0A1L1SS90_MOUSE	N-acyl-L-amino-acid amidohydrolase OS=Mus musculus OX=10090 GN=Acy1 PE=1 SV=1	MOUSE	3
1951	5.38	5.57	29.91	12.39	sp|Q4VAE3|TMM65_MOUSE	Transmembrane protein 65 OS=Mus musculus OX=10090 GN=Tmem65 PE=1 SV=1	MOUSE	3
1952	5.38	5.52	10.58	10.58	tr|Q3V386|Q3V386_MOUSE	Wiskott-Aldrich syndrome protein family member OS=Mus musculus OX=10090 GN=Wasf3 PE=2 SV=1	MOUSE	4
1953	5.38	5.44	31.61	31.61	tr|Q8C1D9|Q8C1D9_MOUSE	V-type proton ATPase proteolipid subunit OS=Mus musculus OX=10090 GN=Atp6v0c PE=2 SV=1	MOUSE	3
1954	5.37	5.42	26.79	26.32	sp|Q9DD18|DTD1_MOUSE	D-aminoacyl-tRNA deacylase 1 OS=Mus musculus OX=10090 GN=Dtd1 PE=1 SV=2	MOUSE	3
1955	5.36	5.84	14.84	7.98	sp|Q3UHD6|SNX27_MOUSE	Sorting nexin-27 OS=Mus musculus OX=10090 GN=Snx27 PE=1 SV=2	MOUSE	4
1956	5.36	5.45	12.28	6.57	tr|A1L3S7|A1L3S7_MOUSE	Gatad2b protein OS=Mus musculus OX=10090 GN=Gatad2b PE=1 SV=1	MOUSE	3
1957	5.35	5.44	36.05	25.00	tr|Q684Q9|Q684Q9_MOUSE	Ubiquitin-like protein 4A (Fragment) OS=Mus musculus OX=10090 PE=2 SV=1	MOUSE	3

1958	5.33	7.97	41.41	33.04	sp|Q8R1V4|TMED4_MOUSE	Transmembrane emp24 domain-containing protein 4 OS=Mus musculus OX=10090 GN=Tmed4 PE=1 SV=1	MOUSE	7
1959	5.32	13.38	70.37	48.15	sp|Q61411|RASH_MOUSE	GTPase HRas OS=Mus musculus OX=10090 GN=Hras PE=1 SV=2	MOUSE	9
1960	5.32	5.56	33.33	25.37	sp|Q9DB60|PXL2B_MOUSE	Prostamide/prostaglandin F synthase OS=Mus musculus OX=10090 GN=Prxl2b PE=1 SV=1	MOUSE	5
1961	5.32	5.41	14.44	6.34	sp|Q91XU0-2|WRIP1_MOUSE	Isoform 2 of ATPase WRNIP1 OS=Mus musculus OX=10090 GN=Wrnip1	MOUSE	3
1962	5.32	5.38	14.32	10.68	tr|Q6PDD6|Q6PDD6_MOUSE	Stard10 protein (Fragment) OS=Mus musculus OX=10090 GN=Stard10 PE=2 SV=1	MOUSE	4
1963	5.31	5.46	10.60	7.60	tr|S4R2L4|S4R2L4_MOUSE	Arginine/serine-rich coiled-coil protein 2 OS=Mus musculus OX=10090 GN=Rsrc2 PE=1 SV=1	MOUSE	3
1964	5.3	5.4	14.76	10.69	tr|S4R225|S4R225_MOUSE	WD repeat-containing protein 13 OS=Mus musculus OX=10090 GN=Wdr13 PE=1 SV=1	MOUSE	3
1965	5.29	5.37	18.11	7.45	sp|Q922D4-4|PP6R3_MOUSE	Isoform 4 of Serine/threonine-protein phosphatase 6 regulatory subunit 3 OS=Mus musculus OX=10090 GN=Ppp6r3	MOUSE	3
1966	5.28	16.98	51.44	46.15	tr|Q3U4W5|Q3U4W5_MOUSE	Uncharacterized protein OS=Mus musculus OX=10090 GN=Rab6a PE=2 SV=1	MOUSE	9
1967	5.28	7.16	18.00	7.59	tr|A0A0A6YX71|A0A0A6YX71_MOUSE	Serine/threonine-protein kinase DCLK2 OS=Mus musculus OX=10090 GN=Dclk2 PE=1 SV=1	MOUSE	4
1968	5.28	6.97	19.44	17.24	tr|Q7TMN7|Q7TMN7_MOUSE	Annexin OS=Mus musculus OX=10090 GN=Anxa4 PE=2 SV=1	MOUSE	5
1969	5.27	39.51	40.72	40.27	tr|Q149Z9|Q149Z9_MOUSE	Histone cluster 1, H1d OS=Mus musculus OX=10090 GN=H1f3 PE=1 SV=1	MOUSE	36
1970	5.27	5.39	6.55	2.66	tr|Q7TQG5|Q7TQG5_MOUSE	Neogenin OS=Mus musculus OX=10090 GN=Neo1 PE=1 SV=1	MOUSE	3
1971	5.26	5.7	22.28	9.04	sp|Q60902-4|EP15R_MOUSE	Isoform 4 of Epidermal growth factor receptor substrate 15-like 1 OS=Mus musculus OX=10090 GN=Eps15l1	MOUSE	5
1972	5.26	5.59	17.01	12.91	sp|Q56A08|GPKOW_MOUSE	G-patch domain and KOW motifs-containing protein OS=Mus musculus OX=10090 GN=Gpkow PE=1 SV=2	MOUSE	4
1973	5.26	5.51	12.49	6.29	tr|E9Q640|E9Q640_MOUSE	RNA-binding protein 26 OS=Mus musculus OX=10090 GN=Rbm26 PE=1 SV=1	MOUSE	5

1974	5.26	5.34	21.17	15.77	sp|Q9DB34|CHM2A_MOUSE	Charged multivesicular body protein 2a OS=Mus musculus OX=10090 GN=Chmp2a PE=1 SV=1	MOUSE	3
1975	5.25	5.42	9.12	2.23	tr|A0A286YCX6|A0A286YCX6_MOUSE	Trafficking protein particle complex 8 OS=Mus musculus OX=10090 GN=Trappc8 PE=1 SV=2	MOUSE	3
1976	5.25	5.33	12.73	8.08	tr|Q3UDS7|Q3UDS7_MOUSE	ADP-dependent glucokinase OS=Mus musculus OX=10090 GN=Adpgk PE=1 SV=1	MOUSE	3
1977	5.25	5.32	52.90	34.36	tr|A0A087WPA0|A0A087WPA0_MOUSE	LBH domain-containing 1 OS=Mus musculus OX=10090 GN=Lbhd1 PE=4 SV=1	MOUSE	5
1978	5.23	5.4	17.00	6.68	tr|Q3UL32|Q3UL32_MOUSE	Uncharacterized protein OS=Mus musculus OX=10090 GN=Dnajc7 PE=2 SV=1	MOUSE	3
1979	5.23	5.36	35.47	20.35	sp|Q91W29|COX42_MOUSE	Cytochrome c oxidase subunit 4 isoform 2, mitochondrial OS=Mus musculus OX=10090 GN=Cox4i2 PE=1 SV=1	MOUSE	3
1980	5.22	5.36	23.95	16.17	sp|Q9CR09|UFC1_MOUSE	Ubiquitin-fold modifier-conjugating enzyme 1 OS=Mus musculus OX=10090 GN=Ufc1 PE=1 SV=1	MOUSE	3
1981	5.21	5.34	22.32	11.61	sp|Q9CX34|SGT1_MOUSE	Protein SGT1 homolog OS=Mus musculus OX=10090 GN=Sugt1 PE=1 SV=3	MOUSE	3
1982	5.2	5.72	17.12	15.56	tr|Q9MD68|Q9MD68_MOUSE	Cytochrome c oxidase subunit 1 OS=Mus musculus OX=10090 GN=mt-Co1 PE=1 SV=1	MOUSE	5
1983	5.2	5.33	28.23	9.57	tr|A0A0R4J0I1|A0A0R4J0I1_MOUSE	Serine protease inhibitor A3K OS=Mus musculus OX=10090 GN=Serpina3k PE=1 SV=1	MOUSE	3
1984	5.19	5.38	13.46	8.26	sp|Q9JHU9|INO1_MOUSE	Inositol-3-phosphate synthase 1 OS=Mus musculus OX=10090 GN=Isyna1 PE=1 SV=1	MOUSE	3
1985	5.19	5.25	13.86	8.32	tr|Q4VA41|Q4VA41_MOUSE	Tubby-like protein OS=Mus musculus OX=10090 GN=Tub PE=2 SV=1	MOUSE	4
1986	5.18	5.39	14.32	12.59	sp|Q8JZX4|SPF45_MOUSE	Splicing factor 45 OS=Mus musculus OX=10090 GN=Rbm17 PE=1 SV=1	MOUSE	4
1987	5.18	5.27	63.94	33.65	tr|Q4FJU3|Q4FJU3_MOUSE	Crip2 protein OS=Mus musculus OX=10090 GN=Crip2 PE=1 SV=1	MOUSE	3
1988	5.15	5.23	50.00	27.66	sp|Q80X32|CE024_MOUSE	UPF0461 protein C5orf24 homolog OS=Mus musculus OX=10090 PE=2 SV=1	MOUSE	3
1989	5.15	5.22	14.87	6.03	tr|Q3UPY7|Q3UPY7_MOUSE	Dolichyl-P-Glc:Glc(2)Man(9)GlcNAc(2)-PP-dolichol alpha-1,2-glucosyltransferase (Fragment) OS=Mus musculus OX=10090 GN=Alg10b PE=2 SV=1	MOUSE	3

1990	5.15	5.22	21.70	10.85	tr|A0A0R4J0M1|A0A0R4J0M1_MOUSE	Tubulin-folding cofactor C OS=Mus musculus OX=10090 GN=Tbcc PE=1 SV=1	MOUSE	3
1991	5.15	5.21	58.30	24.38	sp|Q8VI64|HUMMR_MOUSE	Protein MGARP OS=Mus musculus OX=10090 GN=Mgarp PE=1 SV=1	MOUSE	3
1992	5.13	5.22	42.31	33.65	tr|Q9D6H6|Q9D6H6_MOUSE	Complex I-B12 OS=Mus musculus OX=10090 GN=Ndufb3 PE=2 SV=1	MOUSE	3
1993	5.13	5.21	43.41	37.98	tr|Q9D881|Q9D881_MOUSE	Cytochrome c oxidase polypeptide Vb OS=Mus musculus OX=10090 GN=Cox5b-ps PE=1 SV=1	MOUSE	4
1994	5.13	5.2	17.91	14.53	tr|A0A0A0MQ70|A0A0A0MQ70_MOUSE	Solute carrier family 25 member 35 OS=Mus musculus OX=10090 GN=Slc25a35 PE=1 SV=1	MOUSE	3
1995	5.12	5.24	20.40	16.40	tr|Q8C5F7|Q8C5F7_MOUSE	Protein kinase domain-containing protein OS=Mus musculus OX=10090 PE=2 SV=1	MOUSE	3
1996	5.09	17.73	23.55	16.99	sp|P68404|KPCB_MOUSE	Protein kinase C beta type OS=Mus musculus OX=10090 GN=Prkcb PE=1 SV=4	MOUSE	11
1997	5.09	5.2	15.02	7.23	tr|Q0VDM6|Q0VDM6_MOUSE	Folh1 protein OS=Mus musculus OX=10090 GN=Folh1 PE=1 SV=1	MOUSE	3
1998	5.09	5.15	37.40	37.40	tr|A0A087WPM0|A0A087WPM0_MOUSE	Selenoprotein H OS=Mus musculus OX=10090 GN=Selenoh PE=1 SV=1	MOUSE	5
1999	5.09	5.14	27.62	27.62	sp|Q8R2Y8|PTH2_MOUSE	Peptidyl-tRNA hydrolase 2, mitochondrial OS=Mus musculus OX=10090 GN=Ptrh2 PE=1 SV=1	MOUSE	3
2000	5.08	5.17	17.34	15.17	sp|Q8VDK1|NIT1_MOUSE	Deaminated glutathione amidase OS=Mus musculus OX=10090 GN=Nit1 PE=1 SV=2	MOUSE	3
2001	5.07	5.53	49.72	31.07	tr|Q4G0C5|Q4G0C5_MOUSE	Peptidyl-prolyl cis-trans isomerase OS=Mus musculus OX=10090 GN=Ppih PE=1 SV=1	MOUSE	4
2002	5.07	5.23	20.11	7.31	tr|F8WHL1|F8WHL1_MOUSE	Solute carrier family 30 member 9 OS=Mus musculus OX=10090 GN=Slc30a9 PE=1 SV=1	MOUSE	3
2003	5.06	10.18	51.30	32.64	sp|P62748|HPCL1_MOUSE	Hippocalcin-like protein 1 OS=Mus musculus OX=10090 GN=Hpcal1 PE=1 SV=2	MOUSE	7
2004	5.05	5.15	28.57	19.78	tr|A0A1W2P7J9|A0A1W2P7J9_MOUSE	Glutathione S-transferase theta-2 (Fragment) OS=Mus musculus OX=10090 GN=Gstt2 PE=1 SV=1	MOUSE	3
2005	5.04	5.23	9.44	5.98	sp|A2AR02|PPIG_MOUSE	Peptidyl-prolyl cis-trans isomerase G OS=Mus musculus OX=10090 GN=Ppig PE=1 SV=1	MOUSE	4

2006	5.03	5.15	36.00	8.00	tr|A2ADR8|A2ADR8_MOUSE	Nuclear inhibitor of protein phosphatase 1 OS=Mus musculus OX=10090 GN=Ppp1r8 PE=1 SV=1	MOUSE	2
2007	5.03	5.09	43.28	43.28	tr|Q7JCZ0|Q7JCZ0_MOUSE	ATP synthase protein 8 OS=Mus musculus OX=10090 GN=mt-Atp8 PE=1 SV=1	MOUSE	3
2008	5.02	21.67	85.07	64.18	tr|Q0PD66|Q0PD66_MOUSE	Rab1B OS=Mus musculus OX=10090 GN=Rab1b PE=1 SV=1	MOUSE	11
2009	5.02	5.1	81.63	57.14	sp|Q62426|CYTB_MOUSE	Cystatin-B OS=Mus musculus OX=10090 GN=Cstb PE=1 SV=1	MOUSE	3
2010	5.02	5.08	21.67	21.67	sp|P08030|APT_MOUSE	Adenine phosphoribosyltransferase OS=Mus musculus OX=10090 GN=Aprt PE=1 SV=2	MOUSE	3
2011	5.01	5.16	15.66	9.25	sp|Q8C729|F126B_MOUSE	Protein FAM126B OS=Mus musculus OX=10090 GN=Fam126b PE=1 SV=1	MOUSE	3
2012	4.99	41.86	28.54	22.78	tr|Q3TCR7|Q3TCR7_MOUSE	Dynamin GTPase OS=Mus musculus OX=10090 GN=Dnm2 PE=1 SV=1	MOUSE	29
2013	4.99	5.18	43.15	33.56	sp|P61327|MGN_MOUSE	Protein mago nashi homolog OS=Mus musculus OX=10090 GN=Magoh PE=2 SV=1	MOUSE	4
2014	4.99	5.05	4.51	3.42	tr|E9QM77|E9QM77_MOUSE	Ataxin-2 OS=Mus musculus OX=10090 GN=Atxn2 PE=1 SV=1	MOUSE	3
2015	4.98	18.25	69.44	64.44	sp|P84084|ARF5_MOUSE	ADP-ribosylation factor 5 OS=Mus musculus OX=10090 GN=Arf5 PE=1 SV=2	MOUSE	11
2016	4.98	5.04	23.40	19.50	tr|A0A1L1SSF8|A0A1L1SSF8_MOUSE	RIKEN cDNA 4931406C07 gene OS=Mus musculus OX=10090 GN=4931406C07Rik PE=1 SV=1	MOUSE	3
2017	4.97	5.16	57.62	52.32	tr|Q3TKM9|Q3TKM9_MOUSE	Actin-related protein 2/3 complex subunit 5 OS=Mus musculus OX=10090 GN=Arpc5 PE=2 SV=1	MOUSE	4
2018	4.97	5.13	14.52	11.98	tr|Q3UGC5|Q3UGC5_MOUSE	Uncharacterized protein OS=Mus musculus OX=10090 GN=Bcl2l13 PE=2 SV=1	MOUSE	3
2019	4.96	5.12	20.96	13.49	tr|Q8C3V7|Q8C3V7_MOUSE	Ferrochelatase OS=Mus musculus OX=10090 GN=Fech PE=2 SV=1	MOUSE	3
2020	4.96	5.04	7.57	2.45	tr|E9QKE4|E9QKE4_MOUSE	Rab3 GTPase-activating protein non-catalytic subunit OS=Mus musculus OX=10090 GN=Rab3gap2 PE=1 SV=1	MOUSE	3
2021	4.95	5.02	79.59	42.86	tr|A0A0N4SVQ1|A0A0N4SVQ1_MOUSE	Cytochrome c oxidase subunit NDUFA4 OS=Mus musculus OX=10090 GN=Ndufa4 PE=1 SV=1	MOUSE	4
2022	4.94	5.17	5.33	1.53	sp|P70206|PLXA1_MOUSE	Plexin-A1 OS=Mus musculus OX=10090 GN=Plxna1 PE=1 SV=1	MOUSE	3

2023	4.93	5.13	14.29	4.86	tr|V9GXJ1|V9GXJ1_MOUSE	Predicted gene, 28036 OS=Mus musculus OX=10090 GN=Gm28036 PE=4 SV=1	MOUSE	3
2024	4.93	4.99	30.81	30.81	tr|Q6P5I1|Q6P5I1_MOUSE	Stromal cell derived factor 2 OS=Mus musculus OX=10090 GN=Sdf2 PE=2 SV=2	MOUSE	4
2025	4.91	5.11	20.43	12.13	sp|Q3UJU9|RMD3_MOUSE	Regulator of microtubule dynamics protein 3 OS=Mus musculus OX=10090 GN=Rmdn3 PE=1 SV=2	MOUSE	4
2026	4.9	5.06	9.72	5.69	sp|Q7TMC8|FCSK_MOUSE	L-fucose kinase OS=Mus musculus OX=10090 GN=Fcsk PE=1 SV=1	MOUSE	3
2027	4.9	4.96	29.55	29.55	tr|Q5NDA4|Q5NDA4_MOUSE	Fatty acid binding protein 7, brain OS=Mus musculus OX=10090 GN=Fabp7 PE=2 SV=1	MOUSE	3
2028	4.88	7.1	19.36	9.79	tr|Q80XD3|Q80XD3_MOUSE	Regulator of G-protein-signaling 7 OS=Mus musculus OX=10090 GN=Rgs7 PE=1 SV=1	MOUSE	4
2029	4.88	5.49	18.65	10.88	sp|Q80WJ7|LYRIC_MOUSE	Protein LYRIC OS=Mus musculus OX=10090 GN=Mtdh PE=1 SV=1	MOUSE	5
2030	4.88	5.04	44.56	27.98	tr|Q4FJX4|Q4FJX4_MOUSE	Csrp1 protein OS=Mus musculus OX=10090 GN=Csrp1 PE=1 SV=1	MOUSE	3
2031	4.88	4.97	18.31	10.12	tr|Q4FJR4|Q4FJR4_MOUSE	Protein-serine/threonine kinase OS=Mus musculus OX=10090 GN=Pdk3 PE=1 SV=1	MOUSE	3
2032	4.87	4.93	32.95	28.98	tr|Q3UMQ2|Q3UMQ2_MOUSE	Down-regulator of transcription 1 OS=Mus musculus OX=10090 GN=Dr1 PE=2 SV=1	MOUSE	3
2033	4.86	5.73	14.75	8.81	tr|G5E8N7|G5E8N7_MOUSE	Bifunctional polynucleotide phosphatase/kinase OS=Mus musculus OX=10090 GN=Pnkp PE=1 SV=1	MOUSE	4
2034	4.86	5	33.01	11.65	sp|Q8VD72|TTC8_MOUSE	Tetratricopeptide repeat protein 8 OS=Mus musculus OX=10090 GN=Ttc8 PE=1 SV=1	MOUSE	3
2035	4.86	5	15.38	7.31	tr|Q542S9|Q542S9_MOUSE	Syntrophin, basic 2 OS=Mus musculus OX=10090 GN=Sntb2 PE=1 SV=1	MOUSE	3
2036	4.86	4.92	5.61	3.79	tr|F8VPN4|F8VPN4_MOUSE	4-alpha-glucanotransferase OS=Mus musculus OX=10090 GN=Agl PE=1 SV=1	MOUSE	3
2037	4.85	6.06	37.67	33.56	sp|P12787|COX5A_MOUSE	Cytochrome c oxidase subunit 5A, mitochondrial OS=Mus musculus OX=10090 GN=Cox5a PE=1 SV=2	MOUSE	6
2038	4.85	4.95	10.69	2.43	sp|Q6NZL0|SOGA3_MOUSE	Protein SOGA3 OS=Mus musculus OX=10090 GN=Soga3 PE=1 SV=2	MOUSE	2
2039	4.85	4.94	13.95	13.95	sp|Q9D832|DNJB4_MOUSE	DnaJ homolog subfamily B member 4 OS=Mus musculus OX=10090 GN=Dnajb4 PE=1 SV=1	MOUSE	4

2040	4.84	5.07	28.06	28.06	tr|D3Z442|D3Z442_MOUSE	Mth938 domain-containing protein OS=Mus musculus OX=10090 GN=Aamdc PE=1 SV=1	MOUSE	3
2041	4.84	4.91	7.24	3.44	sp|B2RXC1|TPC11_MOUSE	Trafficking protein particle complex subunit 11 OS=Mus musculus OX=10090 GN=Trappc11 PE=1 SV=1	MOUSE	3
2042	4.83	6.96	25.58	22.26	tr|Q3UZN6|Q3UZN6_MOUSE	Phosducin domain-containing protein OS=Mus musculus OX=10090 GN=Pdcl PE=2 SV=1	MOUSE	4
2043	4.83	4.89	8.76	8.76	tr|Q920W8|Q920W8_MOUSE	40S ribosomal protein S30 (Fragment) OS=Mus musculus OX=10090 GN=fau PE=3 SV=1	MOUSE	4
2044	4.82	98.42	68.28	62.98	sp|P39053|DYN1_MOUSE	Dynamin-1 OS=Mus musculus OX=10090 GN=Dnm1 PE=1 SV=2	MOUSE	60
2045	4.82	4.97	10.94	6.57	tr|Q543P6|Q543P6_MOUSE	Uncharacterized protein OS=Mus musculus OX=10090 GN=Acot11 PE=1 SV=1	MOUSE	3
2046	4.82	4.93	25.42	8.02	sp|Q9R0H0-2|ACOX1_MOUSE	Isoform 2 of Peroxisomal acyl-coenzyme A oxidase 1 OS=Mus musculus OX=10090 GN=Acox1	MOUSE	3
2047	4.82	4.88	34.88	34.88	tr|Q5EAT0|Q5EAT0_MOUSE	Signal recognition particle 9 kDa protein OS=Mus musculus OX=10090 GN=Srp9 PE=1 SV=1	MOUSE	3
2048	4.81	5.1	28.16	11.71	tr|Q8BIV6|Q8BIV6_MOUSE	Aldo_ket_red domain-containing protein OS=Mus musculus OX=10090 GN=Akr1b10 PE=2 SV=1	MOUSE	3
2049	4.81	5.07	58.79	30.77	sp|P09528|FRIH_MOUSE	Ferritin heavy chain OS=Mus musculus OX=10090 GN=Fth1 PE=1 SV=2	MOUSE	4
2050	4.78	4.95	6.28	1.80	tr|F8WIE5|F8WIE5_MOUSE	HECT-type E3 ubiquitin transferase OS=Mus musculus OX=10090 GN=Hectd1 PE=1 SV=1	MOUSE	3
2051	4.78	4.86	10.43	6.59	tr|Q3UME9|Q3UME9_MOUSE	Uncharacterized protein OS=Mus musculus OX=10090 GN=Mogs PE=2 SV=1	MOUSE	3
2052	4.78	4.84	25.18	14.39	tr|Q3UKQ5|Q3UKQ5_MOUSE	Uncharacterized protein OS=Mus musculus OX=10090 GN=M6pr PE=1 SV=1	MOUSE	4
2053	4.77	7.45	11.18	6.36	tr|B1AQ77|B1AQ77_MOUSE	Keratin, type I cytoskeletal 15 OS=Mus musculus OX=10090 GN=Krt15 PE=1 SV=1	MOUSE	5
2054	4.76	4.95	7.72	6.61	tr|Q3V3N5|Q3V3N5_MOUSE	Nucleolar GTP-binding protein 2 (Fragment) OS=Mus musculus OX=10090 GN=Gnl2 PE=2 SV=1	MOUSE	3
2055	4.76	4.94	9.64	4.04	tr|B9EKP8|B9EKP8_MOUSE	Sorbin and SH3 domain-containing protein 2 OS=Mus musculus OX=10090 GN=Sorbs2 PE=1 SV=1	MOUSE	3
2056	4.75	5	7.30	2.94	sp|P54763|EPHB2_MOUSE	Ephrin type-B receptor 2 OS=Mus musculus OX=10090 GN=Ephb2 PE=1 SV=3	MOUSE	3

2057	4.74	5	7.41	5.04	tr|B2RRY4|B2RRY4_MOUSE	MAGUK p55 subfamily member 5 OS=Mus musculus OX=10090 GN=Mpp5 PE=1 SV=1	MOUSE	3
2058	4.74	4.87	29.07	20.93	tr|A2A4K0|A2A4K0_MOUSE	ESCRT-II complex subunit VPS25 OS=Mus musculus OX=10090 GN=Vps25 PE=1 SV=1	MOUSE	3
2059	4.73	5.09	20.71	14.05	tr|Q3TZT4|Q3TZT4_MOUSE	Inositol-polyphosphate 5-phosphatase OS=Mus musculus OX=10090 GN=Inpp5a PE=1 SV=1	MOUSE	5
2060	4.73	5.04	19.46	11.08	sp|Q9JL35|HMGN5_MOUSE	High mobility group nucleosome-binding domain-containing protein 5 OS=Mus musculus OX=10090 GN=Hmgn5 PE=1 SV=2	MOUSE	3
2061	4.73	4.92	30.38	11.54	tr|Q3UIB5|Q3UIB5_MOUSE	Uncharacterized protein OS=Mus musculus OX=10090 GN=Dhrs4 PE=2 SV=1	MOUSE	3
2062	4.73	4.79	31.13	31.13	tr|Q5M9P1|Q5M9P1_MOUSE	Ribosomal protein L36A OS=Mus musculus OX=10090 GN=Rpl36a PE=1 SV=1	MOUSE	4
2063	4.72	4.96	20.41	7.91	sp|Q8R0F6|ILKAP_MOUSE	Integrin-linked kinase-associated serine/threonine phosphatase 2C OS=Mus musculus OX=10090 GN=Ilkap PE=1 SV=1	MOUSE	4
2064	4.7	4.9	25.44	14.98	sp|Q9QZD8|DIC_MOUSE	Mitochondrial dicarboxylate carrier OS=Mus musculus OX=10090 GN=Slc25a10 PE=1 SV=2	MOUSE	3
2065	4.7	4.8	25.21	25.21	sp|Q9D1K2|VATF_MOUSE	V-type proton ATPase subunit F OS=Mus musculus OX=10090 GN=Atp6v1f PE=1 SV=2	MOUSE	3
2066	4.65	4.97	17.06	6.92	tr|Q3TTV6|Q3TTV6_MOUSE	Tuftelin-interacting protein 11 OS=Mus musculus OX=10090 GN=Tfip11 PE=1 SV=1	MOUSE	4
2067	4.65	4.72	26.11	11.67	tr|Q3U9K9|Q3U9K9_MOUSE	Trafficking protein particle complex subunit OS=Mus musculus OX=10090 GN=Trappc3 PE=1 SV=1	MOUSE	2
2068	4.64	5.44	10.79	3.09	tr|Q5DTM9|Q5DTM9_MOUSE	MKIAA4112 protein (Fragment) OS=Mus musculus OX=10090 GN=Ppfia2 PE=2 SV=1	MOUSE	3
2069	4.64	4.72	20.04	3.93	sp|Q3TFD2|PCAT1_MOUSE	Lysophosphatidylcholine acyltransferase 1 OS=Mus musculus OX=10090 GN=Lpcat1 PE=1 SV=1	MOUSE	2
2070	4.64	4.71	30.25	19.14	tr|Q8R0H0|Q8R0H0_MOUSE	DNA-directed RNA polymerase II subunit E OS=Mus musculus OX=10090 GN=Polr2e PE=2 SV=1	MOUSE	2
2071	4.64	4.7	17.52	8.76	sp|Q6Q783|KMT5C_MOUSE	Histone-lysine N-methyltransferase KMT5C OS=Mus musculus OX=10090 GN=Kmt5c PE=1 SV=1	MOUSE	3
2072	4.63	4.91	15.60	12.82	sp|Q7TNR6|IGS21_MOUSE	Immunoglobulin superfamily member 21 OS=Mus musculus OX=10090 GN=Igsf21 PE=1 SV=1	MOUSE	4

2073	4.63	4.67	13.04	9.57	tr|Q9D3J1|Q9D3J1_MOUSE	Peptidyl-prolyl cis-trans isomerase OS=Mus musculus OX=10090 GN=Ppil4 PE=2 SV=1	MOUSE	3
2074	4.61	6.8	35.21	16.55	tr|E9Q6X0|E9Q6X0_MOUSE	Microtubule-associated protein RP/EB family member 2 OS=Mus musculus OX=10090 GN=Mapre2 PE=1 SV=1	MOUSE	3
2075	4.61	4.85	7.50	4.07	tr|A2AQA7|A2AQA7_MOUSE	RNA helicase aquarius OS=Mus musculus OX=10090 GN=Aqr PE=1 SV=1	MOUSE	3
2076	4.61	4.75	6.10	6.10	tr|Q3U409|Q3U409_MOUSE	Uncharacterized protein OS=Mus musculus OX=10090 GN=Ablim2 PE=2 SV=1	MOUSE	3
2077	4.61	4.7	46.34	46.34	tr|Q5M9N6|Q5M9N6_MOUSE	Rpl37a protein OS=Mus musculus OX=10090 GN=Rpl37a PE=2 SV=1	MOUSE	3
2078	4.6	4.79	12.55	6.79	sp|Q3U0L2|AN33B_MOUSE	Ankyrin repeat domain-containing protein 33B OS=Mus musculus OX=10090 GN=Ankrd33b PE=1 SV=1	MOUSE	3
2079	4.6	4.78	17.00	12.39	tr|A3KGR9|A3KGR9_MOUSE	Histocompatibility 13 (Fragment) OS=Mus musculus OX=10090 GN=H13 PE=1 SV=1	MOUSE	3
2080	4.6	4.75	19.71	10.29	tr|Q1XID4|Q1XID4_MOUSE	ATPase H(+)-transporting lysosomal accessory protein 2 OS=Mus musculus OX=10090 GN=Atp6ap2 PE=1 SV=2	MOUSE	3
2081	4.59	5.74	16.39	6.30	sp|Q9CYT6|CAP2_MOUSE	Adenylyl cyclase-associated protein 2 OS=Mus musculus OX=10090 GN=Cap2 PE=1 SV=1	MOUSE	3
2082	4.58	4.81	19.82	13.36	tr|Q3TK95|Q3TK95_MOUSE	Uncharacterized protein OS=Mus musculus OX=10090 GN=Eif4e PE=1 SV=1	MOUSE	3
2083	4.58	4.63	37.14	23.27	tr|Q545K4|Q545K4_MOUSE	Eukaryotic translation initiation factor 6 OS=Mus musculus OX=10090 GN=Eif6 PE=1 SV=1	MOUSE	3
2084	4.57	4.86	28.93	12.58	sp|O55240|RDH5_MOUSE	Retinol dehydrogenase 5 OS=Mus musculus OX=10090 GN=Rdh5 PE=1 SV=1	MOUSE	3
2085	4.57	4.71	34.97	29.45	tr|Q5M9J2|Q5M9J2_MOUSE	Histidine triad nucleotide binding protein 2 OS=Mus musculus OX=10090 GN=Hint2 PE=1 SV=1	MOUSE	3
2086	4.57	4.69	21.31	16.80	tr|Q5U452|Q5U452_MOUSE	Non-specific serine/threonine protein kinase OS=Mus musculus OX=10090 GN=Trp53rka PE=1 SV=1	MOUSE	3
2087	4.57	4.68	9.89	6.06	tr|A7MD55|A7MD55_MOUSE	Grm6 protein (Fragment) OS=Mus musculus OX=10090 GN=Grm6 PE=2 SV=1	MOUSE	3
2088	4.56	4.75	22.44	16.34	sp|E9Q4P1|WDFY1_MOUSE	WD repeat and FYVE domain-containing protein 1 OS=Mus musculus OX=10090 GN=Wdfy1 PE=1 SV=1	MOUSE	4
2089	4.56	4.6	11.82	10.68	sp|Q91VZ6|SMAP1_MOUSE	Stromal membrane-associated protein 1 OS=Mus musculus OX=10090 GN=Smap1 PE=1 SV=1	MOUSE	3

2090	4.55	4.65	17.54	11.69	sp|Q8R4R6|NUP35_MOUSE	Nucleoporin NUP35 OS=Mus musculus OX=10090 GN=Nup35 PE=1 SV=2	MOUSE	3
2091	4.54	4.58	23.05	11.08	tr|Q543Z5|Q543Z5_MOUSE	Protein kinase domain-containing protein OS=Mus musculus OX=10090 GN=Map2k6 PE=1 SV=1	MOUSE	3
2092	4.53	4.72	34.60	8.89	tr|Q3TUF3|Q3TUF3_MOUSE	Calumenin OS=Mus musculus OX=10090 GN=Calu PE=1 SV=1	MOUSE	2
2093	4.53	4.65	30.84	23.36	tr|Q3U6L3|Q3U6L3_MOUSE	Uncharacterized protein OS=Mus musculus OX=10090 GN=Glrx PE=1 SV=1	MOUSE	3
2094	4.53	4.64	14.33	14.04	tr|D3Z383|D3Z383_MOUSE	Mesoderm-specific transcript protein OS=Mus musculus OX=10090 GN=Mest PE=1 SV=2	MOUSE	3
2095	4.53	4.58	40.23	24.71	sp|Q9D1M4|MCA3_MOUSE	Eukaryotic translation elongation factor 1 epsilon-1 OS=Mus musculus OX=10090 GN=Eef1e1 PE=1 SV=1	MOUSE	4
2096	4.51	4.56	26.29	20.00	tr|Q52L78|Q52L78_MOUSE	Alpha(B)-crystallin OS=Mus musculus OX=10090 GN=Cryab PE=1 SV=1	MOUSE	3
2097	4.51	4.55	62.50	62.50	sp|Q9CQS2|NOP10_MOUSE	H/ACA ribonucleoprotein complex subunit 3 OS=Mus musculus OX=10090 GN=Nop10 PE=3 SV=1	MOUSE	3
2098	4.5	4.64	9.88	3.77	tr|Q3UYK8|Q3UYK8_MOUSE	Rab3 GTPase-activating protein catalytic subunit OS=Mus musculus OX=10090 GN=Rab3gap1 PE=2 SV=1	MOUSE	3
2099	4.5	4.64	9.05	4.03	sp|Q4VBE4|EGFLA_MOUSE	Pikachurin OS=Mus musculus OX=10090 GN=Egflam PE=1 SV=1	MOUSE	3
2100	4.5	4.53	17.94	17.56	tr|A0A498WFS2|A0A498WFS2_MOUSE	UBX domain-containing protein 1 OS=Mus musculus OX=10090 GN=Ubxn1 PE=1 SV=1	MOUSE	3
2101	4.49	4.67	15.46	7.57	tr|Q3UE06|Q3UE06_MOUSE	Uncharacterized protein OS=Mus musculus OX=10090 GN=Nploc4 PE=2 SV=1	MOUSE	4
2102	4.49	4.65	15.46	8.83	sp|Q99ME9|GTPB4_MOUSE	GTP-binding protein 4 OS=Mus musculus OX=10090 GN=Gtpbp4 PE=1 SV=3	MOUSE	4
2103	4.47	4.77	24.42	24.42	tr|Q9ESU1|Q9ESU1_MOUSE	SWI/SNF-related matrix-associated actin-dependent regulator of chromatin subfamily B member 1 OS=Mus musculus OX=10090 GN=Smarcb1 PE=2 SV=1	MOUSE	5
2104	4.46	4.7	17.85	13.03	tr|Q3UXG7|Q3UXG7_MOUSE	GDP-fucose protein O-fucosyltransferase 1 OS=Mus musculus OX=10090 GN=Pofut1 PE=1 SV=1	MOUSE	3
2105	4.46	4.62	7.69	4.58	tr|Q2M4J2|Q2M4J2_MOUSE	Phosphoinositide phospholipase C (Fragment) OS=Mus musculus OX=10090 GN=Plcb1 PE=4 SV=1	MOUSE	3

2106	4.44	4.49	30.73	15.14	tr|Q9CZ22|Q9CZ22_MOUSE	Uncharacterized protein OS=Mus musculus OX=10090 GN=Tpd52l2 PE=2 SV=1	MOUSE	2
2107	4.44	4.48	20.84	9.93	sp|Q8VE47|UBA5_MOUSE	Ubiquitin-like modifier-activating enzyme 5 OS=Mus musculus OX=10090 GN=Uba5 PE=1 SV=2	MOUSE	2
2108	4.42	4.59	13.62	7.59	tr|A0A0R4J098|A0A0R4J098_MOUSE	Zinc finger protein 326 OS=Mus musculus OX=10090 GN=Zfp326 PE=1 SV=1	MOUSE	4
2109	4.42	4.57	24.24	7.74	tr|Q6A0D1|Q6A0D1_MOUSE	ER membrane protein complex subunit 2 (Fragment) OS=Mus musculus OX=10090 GN=Emc2 PE=1 SV=1	MOUSE	2
2110	4.4	4.44	31.76	19.59	tr|A0A0G2JGL0|A0A0G2JGL0_MOUSE	Ubiquitin-conjugating enzyme E2 D3 OS=Mus musculus OX=10090 GN=Ube2d3 PE=1 SV=1	MOUSE	5
2111	4.39	4.65	12.85	6.00	tr|Q501L4|Q501L4_MOUSE	General transcription factor IIIC, polypeptide 4 OS=Mus musculus OX=10090 GN=Gtf3c4 PE=2 SV=1	MOUSE	4
2112	4.39	4.6	3.73	3.61	sp|Q3TWI9|CSCL2_MOUSE	CSC1-like protein 2 OS=Mus musculus OX=10090 GN=Tmem63b PE=1 SV=1	MOUSE	4
2113	4.38	8.29	30.98	13.32	tr|Q3U3C4|Q3U3C4_MOUSE	Uncharacterized protein OS=Mus musculus OX=10090 GN=Sh3gl1 PE=2 SV=1	MOUSE	4
2114	4.38	4.52	10.35	4.33	tr|Q3UXN3|Q3UXN3_MOUSE	Cysteinyl-tRNA synthetase OS=Mus musculus OX=10090 GN=Cars PE=2 SV=1	MOUSE	3
2115	4.38	4.51	16.61	4.75	sp|Q8BU33|HACL2_MOUSE	2-hydroxyacyl-CoA lyase 2 OS=Mus musculus OX=10090 GN=Ilvbl PE=1 SV=1	MOUSE	2
2116	4.36	4.49	19.28	14.35	tr|F7CUP3|F7CUP3_MOUSE	Amidohydrolase domain-containing protein 2 OS=Mus musculus OX=10090 GN=Amdhd2 PE=1 SV=2	MOUSE	3
2117	4.36	4.4	20.53	5.30	sp|Q9QYR9|ACOT2_MOUSE	Acyl-coenzyme A thioesterase 2, mitochondrial OS=Mus musculus OX=10090 GN=Acot2 PE=1 SV=2	MOUSE	2
2118	4.35	4.59	13.24	8.07	sp|Q8R3N6|THOC1_MOUSE	THO complex subunit 1 OS=Mus musculus OX=10090 GN=Thoc1 PE=1 SV=1	MOUSE	4
2119	4.35	4.59	25.66	12.24	sp|Q8C6E0|CFA36_MOUSE	Cilia- and flagella-associated protein 36 OS=Mus musculus OX=10090 GN=Cfap36 PE=1 SV=1	MOUSE	3
2120	4.35	4.47	10.62	5.81	sp|Q3UHB8|CC177_MOUSE	Coiled-coil domain-containing protein 177 OS=Mus musculus OX=10090 GN=Ccdc177 PE=1 SV=1	MOUSE	3
2121	4.35	4.46	23.98	11.72	sp|P49586|PCY1A_MOUSE	Choline-phosphate cytidylyltransferase A OS=Mus musculus OX=10090 GN=Pcyt1a PE=1 SV=1	MOUSE	3
2122	4.33	4.53	9.09	5.20	sp|Q8K2F8|LS14A_MOUSE	Protein LSM14 homolog A OS=Mus musculus OX=10090 GN=Lsm14a PE=1 SV=1	MOUSE	3

2123	4.33	4.51	9.80	6.08	sp|Q60770|STXB3_MOUSE	Syntaxin-binding protein 3 OS=Mus musculus OX=10090 GN=Stxbp3 PE=1 SV=1	MOUSE	3
2124	4.33	4.46	42.86	35.40	sp|Q9CQ22|LTOR1_MOUSE	Ragulator complex protein LAMTOR1 OS=Mus musculus OX=10090 GN=Lamtor1 PE=1 SV=1	MOUSE	3
2125	4.32	4.49	11.94	5.39	sp|Q80YD1|SUV3_MOUSE	ATP-dependent RNA helicase SUPV3L1, mitochondrial OS=Mus musculus OX=10090 GN=Supv3l1 PE=1 SV=1	MOUSE	3
2126	4.32	4.35	19.65	13.87	tr|Q9DC94|Q9DC94_MOUSE	Signal sequence receptor subunit delta OS=Mus musculus OX=10090 GN=Ssr4 PE=2 SV=1	MOUSE	2
2127	4.31	11.44	47.50	47.00	tr|Q9DB24|Q9DB24_MOUSE	Transcription elongation factor A (SII)-like 6 OS=Mus musculus OX=10090 GN=Tceal6 PE=1 SV=1	MOUSE	14
2128	4.3	4.57	15.43	10.57	tr|Q8R436|Q8R436_MOUSE	Glutathione synthase (Fragment) OS=Mus musculus OX=10090 PE=3 SV=1	MOUSE	4
2129	4.3	4.33	10.51	5.05	sp|Q8C7K6|PCYXL_MOUSE	Prenylcysteine oxidase-like OS=Mus musculus OX=10090 GN=Pcyox1l PE=1 SV=1	MOUSE	2
2130	4.29	25.38	65.38	58.46	tr|A2AB79|A2AB79_MOUSE	Histone H2A OS=Mus musculus OX=10090 GN=H2aw PE=2 SV=1	MOUSE	40
2131	4.29	4.77	27.71	10.19	sp|Q9CRY7|GDPD1_MOUSE	Lysophospholipase D GDPD1 OS=Mus musculus OX=10090 GN=Gdpd1 PE=1 SV=1	MOUSE	2
2132	4.28	4.4	34.87	24.37	sp|Q8BGT7|SPF30_MOUSE	Survival of motor neuron-related-splicing factor 30 OS=Mus musculus OX=10090 GN=Smndc1 PE=1 SV=1	MOUSE	3
2133	4.27	4.41	7.53	5.73	sp|Q8BH66|ATLA1_MOUSE	Atlastin-1 OS=Mus musculus OX=10090 GN=Atl1 PE=1 SV=1	MOUSE	3
2134	4.27	4.31	20.92	8.31	sp|Q05186|RCN1_MOUSE	Reticulocalbin-1 OS=Mus musculus OX=10090 GN=Rcn1 PE=1 SV=1	MOUSE	2
2135	4.27	4.3	12.62	1.92	tr|E9Q0N0|E9Q0N0_MOUSE	Intersectin-1 OS=Mus musculus OX=10090 GN=Itsn1 PE=1 SV=1	MOUSE	2
2136	4.26	4.4	14.23	4.65	sp|Q9WTU0|PHF2_MOUSE	Lysine-specific demethylase PHF2 OS=Mus musculus OX=10090 GN=Phf2 PE=1 SV=2	MOUSE	3
2137	4.23	4.34	21.32	17.05	tr|Q78P92|Q78P92_MOUSE	FSHD Region Gene 1 protein OS=Mus musculus OX=10090 GN=Frg1 PE=1 SV=1	MOUSE	3
2138	4.22	4.71	8.45	4.01	tr|Q6DI95|Q6DI95_MOUSE	Transportin 3 OS=Mus musculus OX=10090 GN=Tnpo3 PE=2 SV=1	MOUSE	2
2139	4.22	4.45	8.44	3.01	sp|G5E870|TRIPC_MOUSE	E3 ubiquitin-protein ligase TRIP12 OS=Mus musculus OX=10090 GN=Trip12 PE=1 SV=1	MOUSE	5

2140	4.22	4.24	13.73	6.47	tr|A0A286YDN1|A0A286YDN1_MOUSE	Echinoderm microtubule-associated protein-like 3 OS=Mus musculus OX=10090 GN=Eml3 PE=1 SV=1	MOUSE	3
2141	4.22	4.24	7.90	5.09	tr|I3PQW3|I3PQW3_MOUSE	La ribonucleoprotein domain family member 7 OS=Mus musculus OX=10090 GN=Larp7 PE=3 SV=1	MOUSE	2
2142	4.21	4.29	6.67	4.52	sp|Q99KG3|RBM10_MOUSE	RNA-binding protein 10 OS=Mus musculus OX=10090 GN=Rbm10 PE=1 SV=1	MOUSE	3
2143	4.21	4.24	45.45	38.38	sp|Q9DCB1|HMGN3_MOUSE	High mobility group nucleosome-binding domain-containing protein 3 OS=Mus musculus OX=10090 GN=Hmgn3 PE=1 SV=1	MOUSE	2
2144	4.21	4.23	10.38	6.40	tr|Q8C8H5|Q8C8H5_MOUSE	Uncharacterized protein OS=Mus musculus OX=10090 GN=Gabrg2 PE=2 SV=1	MOUSE	2
2145	4.18	4.74	33.62	19.66	sp|Q8VDQ1|PTGR2_MOUSE	Prostaglandin reductase 2 OS=Mus musculus OX=10090 GN=Ptgr2 PE=1 SV=2	MOUSE	5
2146	4.18	4.66	16.03	5.23	tr|E9QK41|E9QK41_MOUSE	Actin-binding LIM protein 1 OS=Mus musculus OX=10090 GN=Ablim1 PE=1 SV=1	MOUSE	3
2147	4.18	4.33	36.10	26.56	tr|Q14C26|Q14C26_MOUSE	ER membrane protein complex subunit 7 OS=Mus musculus OX=10090 GN=Emc7 PE=1 SV=1	MOUSE	4
2148	4.18	4.2	6.25	3.04	tr|V9GXD9|V9GXD9_MOUSE	Synergin gamma OS=Mus musculus OX=10090 GN=Synrg PE=1 SV=1	MOUSE	2
2149	4.17	8.25	53.01	40.96	tr|Q3UHW9|Q3UHW9_MOUSE	ADF-H domain-containing protein OS=Mus musculus OX=10090 GN=Cfl2 PE=1 SV=1	MOUSE	5
2150	4.17	4.33	21.03	13.49	tr|A0A140LJ01|A0A140LJ01_MOUSE	Microsomal signal peptidase 25 kDa subunit (Fragment) OS=Mus musculus OX=10090 GN=Spcs2 PE=1 SV=1	MOUSE	3
2151	4.16	4.18	42.14	15.71	tr|Q4VA32|Q4VA32_MOUSE	Thioesterase superfamily member 2 OS=Mus musculus OX=10090 GN=Acot13 PE=1 SV=1	MOUSE	2
2152	4.16	4.18	23.65	18.24	sp|Q9JMG1|EDF1_MOUSE	Endothelial differentiation-related factor 1 OS=Mus musculus OX=10090 GN=Edf1 PE=1 SV=1	MOUSE	2
2153	4.15	4.25	19.75	14.18	sp|Q3TSG4|ALKB5_MOUSE	RNA demethylase ALKBH5 OS=Mus musculus OX=10090 GN=Alkbh5 PE=1 SV=2	MOUSE	3
2154	4.15	4.25	12.59	12.59	tr|Q80VX2|Q80VX2_MOUSE	CD200 antigen OS=Mus musculus OX=10090 GN=Cd200 PE=1 SV=1	MOUSE	3
2155	4.15	4.16	18.77	14.08	sp|Q9QX60|DGUOK_MOUSE	Deoxyguanosine kinase, mitochondrial OS=Mus musculus OX=10090 GN=Dguok PE=1 SV=3	MOUSE	3

2156	4.14	4.49	26.76	11.71	tr|Q3TXT4|Q3TXT4_MOUSE	Uncharacterized protein OS=Mus musculus OX=10090 GN=Snx5 PE=2 SV=1	MOUSE	4
2157	4.12	8	9.46	4.77	sp|Q8JZR6|S4A8_MOUSE	Electroneutral sodium bicarbonate exchanger 1 OS=Mus musculus OX=10090 GN=Slc4a8 PE=2 SV=1	MOUSE	4
2158	4.12	4.21	8.10	8.10	tr|Q80X37|Q80X37_MOUSE	CD44 antigen OS=Mus musculus OX=10090 GN=Cd44 PE=1 SV=1	MOUSE	3
2159	4.11	4.26	18.97	6.42	tr|Q8N9U3|Q8N9U3_MOUSE	Propionate--CoA ligase OS=Mus musculus OX=10090 GN=Acss2 PE=2 SV=1	MOUSE	2
2160	4.11	4.24	22.33	17.48	tr|Q3U6G1|Q3U6G1_MOUSE	Uncharacterized protein OS=Mus musculus OX=10090 GN=Blvrb PE=1 SV=1	MOUSE	3
2161	4.1	7.24	17.99	16.81	sp|Q3TWW8|SRSF6_MOUSE	Serine/arginine-rich splicing factor 6 OS=Mus musculus OX=10090 GN=Srsf6 PE=1 SV=1	MOUSE	6
2162	4.1	6.59	20.92	11.52	tr|Q4FJX1|Q4FJX1_MOUSE	Importin subunit alpha OS=Mus musculus OX=10090 GN=Kpna4 PE=1 SV=1	MOUSE	3
2163	4.1	4.19	31.95	16.60	sp|Q9WVQ5|MTNB_MOUSE	Methylthioribulose-1-phosphate dehydratase OS=Mus musculus OX=10090 GN=Apip PE=1 SV=1	MOUSE	2
2164	4.1	4.19	9.29	5.05	sp|Q8K124|PKHO2_MOUSE	Pleckstrin homology domain-containing family O member 2 OS=Mus musculus OX=10090 GN=Plekho2 PE=1 SV=1	MOUSE	2
2165	4.1	4.13	9.74	3.20	tr|Q571J8|Q571J8_MOUSE	Non-specific serine/threonine protein kinase (Fragment) OS=Mus musculus OX=10090 GN=Mark2 PE=2 SV=1	MOUSE	2
2166	4.1	4.11	22.26	6.83	tr|Q3UFZ6|Q3UFZ6_MOUSE	Caprin-1_dimer domain-containing protein (Fragment) OS=Mus musculus OX=10090 GN=Caprin1 PE=2 SV=1	MOUSE	3
2167	4.1	4.11	9.18	3.94	sp|P21981|TGM2_MOUSE	Protein-glutamine gamma-glutamyltransferase 2 OS=Mus musculus OX=10090 GN=Tgm2 PE=1 SV=4	MOUSE	2
2168	4.09	4.1	10.71	7.14	sp|Q9D387|LAMP5_MOUSE	Lysosome-associated membrane glycoprotein 5 OS=Mus musculus OX=10090 GN=Lamp5 PE=1 SV=2	MOUSE	3
2169	4.08	4.27	8.53	4.26	tr|Q8CCX6|Q8CCX6_MOUSE	Protein VAC14 homolog OS=Mus musculus OX=10090 GN=Vac14 PE=2 SV=1	MOUSE	2
2170	4.08	4.18	33.67	24.49	sp|Q9CQU3|RER1_MOUSE	Protein RER1 OS=Mus musculus OX=10090 GN=Rer1 PE=1 SV=1	MOUSE	3
2171	4.08	4.17	7.34	2.38	tr|A2AKI5|A2AKI5_MOUSE	Integrin alpha-V OS=Mus musculus OX=10090 GN=Itgav PE=1 SV=1	MOUSE	2

2172	4.08	4.09	15.31	10.94	tr|Q8BNF3|Q8BNF3_MOUSE	Uncharacterized protein OS=Mus musculus OX=10090 PE=2 SV=1	MOUSE	2
2173	4.07	4.53	16.01	11.58	sp|Q6P8X1|SNX6_MOUSE	Sorting nexin-6 OS=Mus musculus OX=10090 GN=Snx6 PE=1 SV=2	MOUSE	3
2174	4.07	4.45	12.35	7.53	tr|Q8CII3|Q8CII3_MOUSE	3-ketodihydrosphingosine reductase OS=Mus musculus OX=10090 GN=Kdsr PE=1 SV=2	MOUSE	3
2175	4.07	4.24	17.22	10.26	tr|Q8BSZ8|Q8BSZ8_MOUSE	PABS domain-containing protein OS=Mus musculus OX=10090 GN=Srm PE=2 SV=1	MOUSE	3
2176	4.07	4.09	20.26	5.19	sp|Q6ZPU9|KBP_MOUSE	KIF-binding protein OS=Mus musculus OX=10090 GN=Kifbp PE=1 SV=2	MOUSE	2
2177	4.07	4.08	21.03	16.31	tr|Q9D927|Q9D927_MOUSE	Uncharacterized protein OS=Mus musculus OX=10090 GN=1810009N02Rik PE=2 SV=1	MOUSE	2
2178	4.06	8.92	26.06	21.36	sp|Q99MR0|ACL6B_MOUSE	Actin-like protein 6B OS=Mus musculus OX=10090 GN=Actl6b PE=1 SV=1	MOUSE	6
2179	4.06	4.07	23.93	12.82	sp|Q9D1R9|RL34_MOUSE	60S ribosomal protein L34 OS=Mus musculus OX=10090 GN=Rpl34 PE=1 SV=2	MOUSE	2
2180	4.05	30.58	53.35	44.85	sp|Q91VM5|RMXL1_MOUSE	RNA binding motif protein, X-linked-like-1 OS=Mus musculus OX=10090 GN=Rbmxl1 PE=2 SV=1	MOUSE	17
2181	4.05	4.17	17.86	6.90	tr|Q3TYV5|Q3TYV5_MOUSE	2',3'-cyclic-nucleotide 3'-phosphodiesterase OS=Mus musculus OX=10090 GN=Cnp PE=2 SV=1	MOUSE	3
2182	4.05	4.12	22.19	7.94	sp|Q9JK48|SHLB1_MOUSE	Endophilin-B1 OS=Mus musculus OX=10090 GN=Sh3glb1 PE=1 SV=1	MOUSE	2
2183	4.05	4.08	19.29	10.35	tr|D3YWK1|D3YWK1_MOUSE	WD repeat domain phosphoinositide-interacting protein 2 OS=Mus musculus OX=10090 GN=Wipi2 PE=1 SV=1	MOUSE	2
2184	4.05	4.06	11.53	3.17	sp|E9PV24|FIBA_MOUSE	Fibrinogen alpha chain OS=Mus musculus OX=10090 GN=Fga PE=1 SV=1	MOUSE	2
2185	4.05	4.05	24.47	10.13	sp|Q9D273|MMAB_MOUSE	Corrinoid adenosyltransferase OS=Mus musculus OX=10090 GN=Mmab PE=1 SV=1	MOUSE	2
2186	4.04	4.44	6.37	1.31	tr|E9Q0F0|E9Q0F0_MOUSE	Keratin 78 OS=Mus musculus OX=10090 GN=Krt78 PE=1 SV=1	MOUSE	3
2187	4.04	4.15	8.52	2.60	tr|M1H0B0|M1H0B0_MOUSE	Anoctamin OS=Mus musculus OX=10090 GN=Ano2 PE=2 SV=2	MOUSE	2
2188	4.04	4.05	26.05	11.49	tr|D3YTR5|D3YTR5_MOUSE	Methyl-CpG-binding domain protein 3 OS=Mus musculus OX=10090 GN=Mbd3 PE=1 SV=1	MOUSE	2

2189	4.04	4.05	39.77	27.27	sp|P56135|ATPK_MOUSE	ATP synthase subunit f, mitochondrial OS=Mus musculus OX=10090 GN=Atp5mf PE=1 SV=3	MOUSE	5
2190	4.04	4.05	2.85	2.28	tr|Q80X98|Q80X98_MOUSE	RNA helicase OS=Mus musculus OX=10090 GN=Dhx38 PE=1 SV=1	MOUSE	2
2191	4.04	4.04	27.04	17.41	sp|Q9D7I5|LHPP_MOUSE	Phospholysine phosphohistidine inorganic pyrophosphate phosphatase OS=Mus musculus OX=10090 GN=Lhpp PE=1 SV=1	MOUSE	2
2192	4.03	4.13	32.86	32.86	tr|Q9EPX9|Q9EPX9_MOUSE	Cystatin C OS=Mus musculus OX=10090 GN=Cst3 PE=2 SV=1	MOUSE	3
2193	4.03	4.05	7.45	4.41	tr|Q69ZS5|Q69ZS5_MOUSE	MKIAA1065 protein (Fragment) OS=Mus musculus OX=10090 GN=Epn2 PE=2 SV=1	MOUSE	2
2194	4.03	4.03	26.85	18.52	sp|Q9D2R6|COA3_MOUSE	Cytochrome c oxidase assembly factor 3 homolog, mitochondrial OS=Mus musculus OX=10090 GN=Coa3 PE=1 SV=1	MOUSE	2
2195	4.02	20.24	61.90	44.44	sp|Q64524|H2B2E_MOUSE	Histone H2B type 2-E OS=Mus musculus OX=10090 GN=H2bc21 PE=1 SV=3	MOUSE	52
2196	4.02	4.11	14.52	10.89	sp|Q91WT4|DJC17_MOUSE	DnaJ homolog subfamily C member 17 OS=Mus musculus OX=10090 GN=Dnajc17 PE=1 SV=2	MOUSE	2
2197	4.02	4.02	9.73	5.35	tr|S4R1K5|S4R1K5_MOUSE	Rho guanine nucleotide exchange factor 9 OS=Mus musculus OX=10090 GN=Arhgef9 PE=1 SV=1	MOUSE	2
2198	4.02	4.02	27.27	20.45	tr|Q9D7P1|Q9D7P1_MOUSE	40S ribosomal protein S24 OS=Mus musculus OX=10090 GN=Rps24 PE=2 SV=1	MOUSE	2
2199	4.02	4.02	17.00	13.50	tr|Q9D7S5|Q9D7S5_MOUSE	Methyltransferase-like 7A1 OS=Mus musculus OX=10090 GN=Mettl7a1 PE=1 SV=1	MOUSE	2
2200	4.01	4.06	12.39	8.93	tr|Q3UBS3|Q3UBS3_MOUSE	Haptoglobin OS=Mus musculus OX=10090 GN=Hp PE=1 SV=1	MOUSE	2
2201	4.01	4.02	17.01	10.82	sp|Q9CY97|SSU72_MOUSE	RNA polymerase II subunit A C-terminal domain phosphatase SSU72 OS=Mus musculus OX=10090 GN=Ssu72 PE=1 SV=1	MOUSE	2
2202	4.01	4.01	15.33	9.19	tr|Q9DAN5|Q9DAN5_MOUSE	J domain-containing protein OS=Mus musculus OX=10090 GN=Dnajb6 PE=2 SV=1	MOUSE	2
2203	4.01	4.01	6.86	4.29	tr|Q54AJ5|Q54AJ5_MOUSE	Activated leukocyte cell adhesion molecule OS=Mus musculus OX=10090 GN=Alcam PE=1 SV=1	MOUSE	2

2204	4.01	4.01	27.74	16.79	tr|D3YVR9|D3YVR9_MOUSE	RIKEN cDNA 1110004F10 gene OS=Mus musculus OX=10090 GN=1110004F10Rik PE=1 SV=1	MOUSE	2
2205	4.01	4.01	12.81	8.12	sp|Q3TZX3|S2533_MOUSE	Solute carrier family 25 member 33 OS=Mus musculus OX=10090 GN=Slc25a33 PE=2 SV=1	MOUSE	2
2206	4.01	4.01	50.00	20.75	sp|P56375|ACYP2_MOUSE	Acylphosphatase-2 OS=Mus musculus OX=10090 GN=Acyp2 PE=1 SV=2	MOUSE	2
2207	4.01	4.01	20.13	18.87	tr|Q569X3|Q569X3_MOUSE	U1 small nuclear ribonucleoprotein C OS=Mus musculus OX=10090 GN=Snrpc PE=1 SV=1	MOUSE	2
2208	4	92.29	81.57	81.57	sp|Q7TMM9|TBB2A_MOUSE	Tubulin beta-2A chain OS=Mus musculus OX=10090 GN=Tubb2a PE=1 SV=1	MOUSE	128
2209	4	14.59	69.52	60.43	tr|F6XXH9|F6XXH9_MOUSE	Inosine-5'-monophosphate dehydrogenase 1 (Fragment) OS=Mus musculus OX=10090 GN=Impdh1 PE=1 SV=1	MOUSE	9
2210	4	4.14	8.25	5.01	sp|Q3UHE1-2|PITM3_MOUSE	Isoform 2 of Membrane-associated phosphatidylinositol transfer protein 3 OS=Mus musculus OX=10090 GN=Pitpnm3	MOUSE	3
2211	4	4.03	15.61	10.73	tr|Q4FJW2|Q4FJW2_MOUSE	Drap1 protein OS=Mus musculus OX=10090 GN=Drap1 PE=1 SV=1	MOUSE	2
2212	4	4.03	15.14	9.51	tr|Q3U4N2|Q3U4N2_MOUSE	Protein-S-isoprenylcysteine O-methyltransferase OS=Mus musculus OX=10090 GN=Icmt PE=1 SV=1	MOUSE	2
2213	4	4.01	38.04	31.52	sp|Q9Z239|PLM_MOUSE	Phospholemman OS=Mus musculus OX=10090 GN=Fxyd1 PE=1 SV=1	MOUSE	2
2214	4	4	11.41	6.65	tr|Q5FWJ9|Q5FWJ9_MOUSE	Nucleoporin 62 OS=Mus musculus OX=10090 GN=Nup62 PE=1 SV=1	MOUSE	2
2215	4	4	8.50	8.28	tr|Q9ME04|Q9ME04_MOUSE	NADH-ubiquinone oxidoreductase chain 4 OS=Mus musculus OX=10090 GN=ND4 PE=3 SV=1	MOUSE	2
2216	4	4	39.06	37.50	tr|Q5NCJ9|Q5NCJ9_MOUSE	Uncharacterized protein OS=Mus musculus OX=10090 GN=Uqcr10 PE=1 SV=1	MOUSE	2
2217	4	4	24.14	22.99	tr|Q542R6|Q542R6_MOUSE	Rhodopsin-sensitive cGMP 3',5'-cyclic phosphodiesterase subunit gamma OS=Mus musculus OX=10090 GN=Pde6g PE=2 SV=1	MOUSE	3
2218	4	4	23.23	18.71	sp|Q9DB70|FUND1_MOUSE	FUN14 domain-containing protein 1 OS=Mus musculus OX=10090 GN=Fundc1 PE=1 SV=1	MOUSE	2
2219	4	4	16.88	16.88	sp|O70591|PFD2_MOUSE	Prefoldin subunit 2 OS=Mus musculus OX=10090 GN=Pfdn2 PE=1 SV=2	MOUSE	2

2220	4	4	29.31	29.31	tr|Q9D6M5|Q9D6M5_MOUSE	Uncharacterized protein OS=Mus musculus OX=10090 GN=Dynlt3 PE=2 SV=1	MOUSE	2
2221	4	4	30.30	30.30	tr|Q9CV53|Q9CV53_MOUSE	Glycine cleavage system H protein (Fragment) OS=Mus musculus OX=10090 GN=Gcsh PE=2 SV=1	MOUSE	2
2222	4	4	26.42	26.42	tr|Q8C297|Q8C297_MOUSE	Uncharacterized protein OS=Mus musculus OX=10090 GN=Tmem14c PE=2 SV=1	MOUSE	2
2223	4	4	6.32	6.32	tr|Q8BXX3|Q8BXX3_MOUSE	GPI-anchor transamidase OS=Mus musculus OX=10090 GN=Pigk PE=1 SV=1	MOUSE	2
2224	4	4	20.31	20.31	tr|Q3V424|Q3V424_MOUSE	Small nuclear ribonucleoprotein G OS=Mus musculus OX=10090 GN=Snrpg PE=2 SV=1	MOUSE	2
2225	4	4	22.41	22.41	tr|Q3UYI4|Q3UYI4_MOUSE	Cocaine- and amphetamine-regulated transcript protein OS=Mus musculus OX=10090 GN=Cartpt PE=2 SV=1	MOUSE	2
2226	4	4	8.88	8.88	tr|Q3TYJ0|Q3TYJ0_MOUSE	Uncharacterized protein OS=Mus musculus OX=10090 GN=Stub1 PE=2 SV=1	MOUSE	2
2227	4	4	10.41	10.41	tr|B2RWB7|B2RWB7_MOUSE	9630033F20Rik protein OS=Mus musculus OX=10090 GN=Tigar PE=1 SV=1	MOUSE	2
2228	4	4	25.74	25.74	sp|Q9CQY6|UQCC2_MOUSE	Ubiquinol-cytochrome-c reductase complex assembly factor 2 OS=Mus musculus OX=10090 GN=Uqcc2 PE=1 SV=1	MOUSE	2
2229	4	4	19.35	19.35	sp|Q8K194|SNR27_MOUSE	U4/U6.U5 small nuclear ribonucleoprotein 27 kDa protein OS=Mus musculus OX=10090 GN=Snrnp27 PE=1 SV=1	MOUSE	2
2230	4	4	16.82	16.82	sp|Q8BFQ8|GALD1_MOUSE	Glutamine amidotransferase-like class 1 domain-containing protein 1 OS=Mus musculus OX=10090 GN=Gatd1 PE=1 SV=1	MOUSE	2
2231	4	4	12.78	12.78	sp|C0HK80|ARXS2_MOUSE	Adipocyte-related X-chromosome expressed sequence 2 OS=Mus musculus OX=10090 GN=Arxes2 PE=1 SV=1	MOUSE	2
2232	3.98	4.14	9.07	1.49	sp|Q8BL66|EEA1_MOUSE	Early endosome antigen 1 OS=Mus musculus OX=10090 GN=Eea1 PE=1 SV=2	MOUSE	2
2233	3.97	4.25	17.04	6.08	tr|Q8BLF7|Q8BLF7_MOUSE	MFS domain-containing protein OS=Mus musculus OX=10090 GN=Slc2a3 PE=2 SV=1	MOUSE	3
2234	3.97	4.25	9.31	5.65	tr|Q14DS6|Q14DS6_MOUSE	Leucyl-tRNA synthetase OS=Mus musculus OX=10090 GN=Lars2 PE=2 SV=1	MOUSE	3

2235	3.97	4.02	16.74	12.55	tr|E9QLB2|E9QLB2_MOUSE	Lysophospholipase-like protein 1 OS=Mus musculus OX=10090 GN=Lyplal1 PE=1 SV=1	MOUSE	2
2236	3.96	8.06	38.03	24.41	tr|B2RRN5|B2RRN5_MOUSE	Ras-related protein Rab-4 OS=Mus musculus OX=10090 GN=Rab4a PE=2 SV=1	MOUSE	4
2237	3.96	4.12	8.13	1.91	tr|B2RXS8|B2RXS8_MOUSE	Protein-tyrosine-phosphatase OS=Mus musculus OX=10090 GN=Ptprz1 PE=1 SV=1	MOUSE	2
2238	3.96	4.04	4.18	2.13	sp|Q6DID3|SCAF8_MOUSE	SR-related and CTD-associated factor 8 OS=Mus musculus OX=10090 GN=Scaf8 PE=1 SV=1	MOUSE	2
2239	3.96	4	8.21	5.53	sp|Q6P5F7|TTYH3_MOUSE	Protein tweety homolog 3 OS=Mus musculus OX=10090 GN=Ttyh3 PE=1 SV=1	MOUSE	2
2240	3.95	4.08	11.53	6.02	tr|S4R213|S4R213_MOUSE	Lactamase, beta-like 1 OS=Mus musculus OX=10090 GN=Lactbl1 PE=4 SV=1	MOUSE	3
2241	3.95	4.04	31.33	11.39	tr|Q3UZJ3|Q3UZJ3_MOUSE	General transcription factor TFIIB OS=Mus musculus OX=10090 GN=Gtf2b PE=2 SV=1	MOUSE	2
2242	3.94	4.05	13.82	6.18	tr|Q6PET3|Q6PET3_MOUSE	Signal peptidase complex catalytic subunit SEC11 (Fragment) OS=Mus musculus OX=10090 GN=Sec11a PE=2 SV=1	MOUSE	2
2243	3.94	4.02	9.29	9.29	sp|Q9JMD0|ZN207_MOUSE	BUB3-interacting and GLEBS motif-containing protein ZNF207 OS=Mus musculus OX=10090 GN=Znf207 PE=1 SV=1	MOUSE	3
2244	3.93	8.43	14.77	7.66	tr|A0A2R8VHP3|A0A2R8VHP3_MOUSE	Predicted pseudogene 5478 OS=Mus musculus OX=10090 GN=Gm5478 PE=1 SV=1	MOUSE	7
2245	3.93	4.1	20.98	7.93	tr|Q3TQR3|Q3TQR3_MOUSE	Eukaryotic translation initiation factor 5 OS=Mus musculus OX=10090 GN=Eif5 PE=1 SV=1	MOUSE	3
2246	3.93	4.01	15.78	7.89	sp|Q99LC2|CSTF1_MOUSE	Cleavage stimulation factor subunit 1 OS=Mus musculus OX=10090 GN=Cstf1 PE=1 SV=1	MOUSE	2
2247	3.92	4.03	34.80	10.80	sp|P70122|SBDS_MOUSE	Ribosome maturation protein SBDS OS=Mus musculus OX=10090 GN=Sbds PE=1 SV=4	MOUSE	2
2248	3.92	4	22.22	15.38	sp|P60521|GBRL2_MOUSE	Gamma-aminobutyric acid receptor-associated protein-like 2 OS=Mus musculus OX=10090 GN=Gabarapl2 PE=1 SV=1	MOUSE	2
2249	3.92	4	10.69	10.69	sp|Q9DC33|HM20A_MOUSE	High mobility group protein 20A OS=Mus musculus OX=10090 GN=Hmg20a PE=1 SV=1	MOUSE	3

2250	3.92	3.96	9.81	5.97	tr|Q8BGR3|Q8BGR3_MOUSE	Calcium/calmodulin-dependent protein kinase IV OS=Mus musculus OX=10090 GN=Camk4 PE=1 SV=1	MOUSE	2
2251	3.91	4.14	8.38	3.62	tr|A0A6Q6PUT5|A0A6Q6PUT5_MOUSE	Pre-mRNA-processing factor 40 homolog A (Fragment) OS=Mus musculus OX=10090 GN=Prpf40a PE=1 SV=1	MOUSE	3
2252	3.91	4.13	22.83	12.32	tr|Q6PCW8|Q6PCW8_MOUSE	Ribosomal RNA-processing protein 43 OS=Mus musculus OX=10090 GN=Exosc8 PE=2 SV=1	MOUSE	3
2253	3.91	4.01	8.22	2.36	sp|Q5DTM8|BRE1A_MOUSE	E3 ubiquitin-protein ligase BRE1A OS=Mus musculus OX=10090 GN=Rnf20 PE=1 SV=2	MOUSE	2
2254	3.9	4.09	14.25	10.98	sp|Q7TN29|SMAP2_MOUSE	Stromal membrane-associated protein 2 OS=Mus musculus OX=10090 GN=Smap2 PE=1 SV=1	MOUSE	3
2255	3.9	3.99	15.13	10.39	tr|G5E881|G5E881_MOUSE	Phosphatidate cytidylyltransferase, mitochondrial OS=Mus musculus OX=10090 GN=Tamm41 PE=1 SV=1	MOUSE	2
2256	3.89	4.06	12.81	7.53	sp|Q8JZM7|CDC73_MOUSE	Parafibromin OS=Mus musculus OX=10090 GN=Cdc73 PE=1 SV=1	MOUSE	3
2257	3.89	3.96	8.47	5.58	tr|Q3TDA1|Q3TDA1_MOUSE	Oxysterol-binding protein OS=Mus musculus OX=10090 GN=Osbpl2 PE=2 SV=1	MOUSE	2
2258	3.89	3.96	11.83	11.83	tr|D3Z417|D3Z417_MOUSE	G/T mismatch-specific thymine DNA glycosylase (Fragment) OS=Mus musculus OX=10090 GN=Tdg PE=1 SV=1	MOUSE	2
2259	3.88	3.95	22.60	7.12	tr|Q569V4|Q569V4_MOUSE	MRG15 protein OS=Mus musculus OX=10090 GN=Morf4l1 PE=2 SV=1	MOUSE	2
2260	3.87	3.97	11.70	4.41	tr|Q3TAR6|Q3TAR6_MOUSE	Protein kinase domain-containing protein OS=Mus musculus OX=10090 GN=Vrk3 PE=1 SV=1	MOUSE	4
2261	3.86	4.01	29.89	14.02	tr|Q8K106|Q8K106_MOUSE	Beta-B1 crystallin OS=Mus musculus OX=10090 GN=Crybb1 PE=2 SV=1	MOUSE	3
2262	3.86	3.95	7.68	2.94	tr|A0A571BG20|A0A571BG20_MOUSE	SRC kinase-signaling inhibitor 1 OS=Mus musculus OX=10090 GN=Srcin1 PE=1 SV=1	MOUSE	2
2263	3.85	3.99	16.60	5.93	tr|A0A0R4J078|A0A0R4J078_MOUSE	UBX domain-containing protein 4 OS=Mus musculus OX=10090 GN=Ubxn4 PE=1 SV=1	MOUSE	2
2264	3.85	3.96	21.31	7.38	sp|Q8VHL1|SETD7_MOUSE	Histone-lysine N-methyltransferase SETD7 OS=Mus musculus OX=10090 GN=Setd7 PE=1 SV=2	MOUSE	2
2265	3.84	3.97	24.62	24.62	sp|Q9CXL3|CG050_MOUSE	Uncharacterized protein C7orf50 homolog OS=Mus musculus OX=10090 PE=1 SV=3	MOUSE	3

2266	3.82	24.98	67.07	64.66	tr|Q8CFQ6|Q8CFQ6_MOUSE	Pcbp3 protein OS=Mus musculus OX=10090 GN=Pcbp3 PE=2 SV=1	MOUSE	15
2267	3.82	4.09	8.60	1.62	sp|Q61687|ATRX_MOUSE	Transcriptional regulator ATRX OS=Mus musculus OX=10090 GN=Atrx PE=1 SV=3	MOUSE	3
2268	3.82	4.09	16.67	7.55	tr|D3Z0V2|D3Z0V2_MOUSE	Rho guanine nucleotide exchange factor 7 (Fragment) OS=Mus musculus OX=10090 GN=Arhgef7 PE=1 SV=2	MOUSE	4
2269	3.82	3.94	9.63	7.11	sp|Q9CZV8|FXL20_MOUSE	F-box/LRR-repeat protein 20 OS=Mus musculus OX=10090 GN=Fbxl20 PE=1 SV=3	MOUSE	3
2270	3.82	3.91	10.30	2.75	tr|Q8BJQ7|Q8BJQ7_MOUSE	Glycine cleavage system P protein OS=Mus musculus OX=10090 GN=Gldc PE=2 SV=1	MOUSE	2
2271	3.82	3.88	7.36	4.45	tr|Q3UNH5|Q3UNH5_MOUSE	YTH domain-containing protein OS=Mus musculus OX=10090 GN=Ythdf2 PE=2 SV=1	MOUSE	2
2272	3.81	4.01	17.48	12.58	tr|B0QZT2|B0QZT2_MOUSE	Dehydrogenase/reductase SDR family member 13 OS=Mus musculus OX=10090 GN=Dhrs13 PE=1 SV=1	MOUSE	3
2273	3.8	4.02	42.11	42.11	sp|P0DN34|NDUB1_MOUSE	NADH dehydrogenase [ubiquinone] 1 beta subcomplex subunit 1 OS=Mus musculus OX=10090 GN=Ndufb1 PE=1 SV=1	MOUSE	3
2274	3.8	3.85	22.55	22.55	tr|A0A087WNP6|A0A087WNP6_MOUSE	Protein CDV3 OS=Mus musculus OX=10090 GN=Cdv3 PE=1 SV=1	MOUSE	2
2275	3.79	4.3	21.89	5.18	sp|Q8BH57|WDR48_MOUSE	WD repeat-containing protein 48 OS=Mus musculus OX=10090 GN=Wdr48 PE=1 SV=1	MOUSE	3
2276	3.79	4.12	11.03	2.03	sp|Q7TT37|ELP1_MOUSE	Elongator complex protein 1 OS=Mus musculus OX=10090 GN=Elp1 PE=1 SV=2	MOUSE	3
2277	3.78	3.94	20.72	17.72	sp|Q9R062|GLYG_MOUSE	Glycogenin-1 OS=Mus musculus OX=10090 GN=Gyg1 PE=1 SV=3	MOUSE	4
2278	3.78	3.9	19.61	19.61	sp|P62071|RRAS2_MOUSE	Ras-related protein R-Ras2 OS=Mus musculus OX=10090 GN=Rras2 PE=1 SV=1	MOUSE	3
2279	3.78	3.87	16.06	6.48	tr|Q3TSR1|Q3TSR1_MOUSE	Vacuolar protein-sorting-associated protein 36 OS=Mus musculus OX=10090 GN=Vps36 PE=1 SV=1	MOUSE	2
2280	3.77	3.97	23.40	14.29	tr|B2FDF6|B2FDF6_MOUSE	Synaptosomal-associated protein 47 OS=Mus musculus OX=10090 GN=Snap47 PE=1 SV=1	MOUSE	3
2281	3.77	3.89	18.50	18.50	tr|Q3V4A0|Q3V4A0_MOUSE	Calcium load-activated calcium channel (Fragment) OS=Mus musculus OX=10090 GN=Tmco1 PE=2 SV=1	MOUSE	3

2282	3.77	3.87	39.16	16.27	sp|Q9CPT4|MYDGF_MOUSE	Myeloid-derived growth factor OS=Mus musculus OX=10090 GN=Mydgf PE=1 SV=1	MOUSE	2
2283	3.77	3.82	6.24	6.24	tr|J3QM95|J3QM95_MOUSE	FSD1-like protein OS=Mus musculus OX=10090 GN=Fsd1l PE=1 SV=1	MOUSE	2
2284	3.76	3.88	26.34	17.94	sp|Q8BIG7|CMTD1_MOUSE	Catechol O-methyltransferase domain-containing protein 1 OS=Mus musculus OX=10090 GN=Comtd1 PE=1 SV=1	MOUSE	3
2285	3.75	4.03	17.46	9.76	tr|G3X8U3|G3X8U3_MOUSE	Queuosine salvage protein OS=Mus musculus OX=10090 GN=2210016F16Rik PE=1 SV=1	MOUSE	3
2286	3.75	3.81	4.67	1.85	tr|F8VPK0|F8VPK0_MOUSE	Tetratricopeptide repeat domain 37 OS=Mus musculus OX=10090 GN=Ttc37 PE=1 SV=1	MOUSE	2
2287	3.74	6.08	10.61	7.50	tr|B2RTP7|B2RTP7_MOUSE	Krt2 protein OS=Mus musculus OX=10090 GN=Krt2 PE=2 SV=1	MOUSE	4
2288	3.74	3.89	21.37	21.37	sp|Q9Z199|SPT4B_MOUSE	Transcription elongation factor SPT4-B OS=Mus musculus OX=10090 GN=Supt4h1b PE=2 SV=1	MOUSE	2
2289	3.74	3.87	14.11	6.61	tr|Q6PGG8|Q6PGG8_MOUSE	Tdrkh protein OS=Mus musculus OX=10090 GN=Tdrkh PE=2 SV=1	MOUSE	3
2290	3.74	3.85	20.97	9.14	sp|Q8BHE3|ATCAY_MOUSE	Caytaxin OS=Mus musculus OX=10090 GN=Atcay PE=1 SV=1	MOUSE	3
2291	3.73	3.86	12.54	5.14	tr|A2AEW8|A2AEW8_MOUSE	GRIP1-associated protein 1 OS=Mus musculus OX=10090 GN=Gripap1 PE=1 SV=1	MOUSE	3
2292	3.72	45.7	67.37	57.92	tr|E9Q2W9|E9Q2W9_MOUSE	Alpha-actinin-4 (Fragment) OS=Mus musculus OX=10090 GN=Actn4 PE=1 SV=1	MOUSE	23
2293	3.72	4.04	6.66	3.26	sp|E9Q5K9|YTDC1_MOUSE	YTH domain-containing protein 1 OS=Mus musculus OX=10090 GN=Ythdc1 PE=1 SV=2	MOUSE	2
2294	3.72	3.83	23.85	11.72	tr|Q5SVP3|Q5SVP3_MOUSE	PA28 beta subunit OS=Mus musculus OX=10090 GN=PSME2b PE=1 SV=1	MOUSE	2
2295	3.71	3.79	16.96	13.84	sp|Q9ERE7|MESD_MOUSE	LRP chaperone MESD OS=Mus musculus OX=10090 GN=Mesd PE=1 SV=1	MOUSE	2
2296	3.7	3.94	50.70	13.02	tr|Q9QXC6|Q9QXC6_MOUSE	Beta-A3/A1 crystallin protein OS=Mus musculus OX=10090 GN=Cryba1 PE=1 SV=1	MOUSE	2
2297	3.7	3.83	17.16	6.31	tr|G3X8W7|G3X8W7_MOUSE	Cell division cycle protein 23 homolog OS=Mus musculus OX=10090 GN=Cdc23 PE=1 SV=1	MOUSE	3
2298	3.7	3.83	19.59	13.06	sp|Q6ZQI3|MLEC_MOUSE	Malectin OS=Mus musculus OX=10090 GN=Mlec PE=1 SV=2	MOUSE	3

2299	3.7	3.81	7.86	4.49	tr|Q5SUH7|Q5SUH7_MOUSE	Clathrin interactor 1 OS=Mus musculus OX=10090 GN=Clint1 PE=1 SV=1	MOUSE	2
2300	3.69	3.76	29.38	20.90	tr|Q542M2|Q542M2_MOUSE	Coatomer subunit zeta OS=Mus musculus OX=10090 GN=Copz1 PE=1 SV=1	MOUSE	2
2301	3.68	5.41	47.74	33.55	tr|F2Z3Z1|F2Z3Z1_MOUSE	Hippocalcin-like protein 4 (Fragment) OS=Mus musculus OX=10090 GN=Hpcal4 PE=1 SV=1	MOUSE	4
2302	3.68	3.85	12.65	2.73	tr|F8VPQ4|F8VPQ4_MOUSE	SLIT-ROBO Rho GTPase-activating protein 3 OS=Mus musculus OX=10090 GN=Srgap3 PE=1 SV=1	MOUSE	2
2303	3.68	3.78	15.44	8.42	tr|Q543X5|Q543X5_MOUSE	CCR4-NOT transcription complex subunit 7 OS=Mus musculus OX=10090 GN=Cnot7 PE=1 SV=1	MOUSE	2
2304	3.66	12	44.14	44.14	tr|Q4VBX4|Q4VBX4_MOUSE	Ubiquitin-conjugating enzyme E2 variant 2 OS=Mus musculus OX=10090 GN=Ube2v2 PE=2 SV=1	MOUSE	6
2305	3.66	3.85	5.62	5.62	tr|G5E811|G5E811_MOUSE	Glycine receptor subunit alpha-3 OS=Mus musculus OX=10090 GN=Glra3 PE=1 SV=1	MOUSE	3
2306	3.66	3.77	23.86	7.61	tr|Q54AG5|Q54AG5_MOUSE	Choline/ethanolamine kinase-beta OS=Mus musculus OX=10090 GN=Chkb PE=1 SV=1	MOUSE	2
2307	3.66	3.76	11.56	5.28	sp|Q99K70|RRAGC_MOUSE	Ras-related GTP-binding protein C OS=Mus musculus OX=10090 GN=Rragc PE=1 SV=1	MOUSE	2
2308	3.66	3.75	9.03	5.14	tr|Q5BKS5|Q5BKS5_MOUSE	Hook homolog 3 (Drosophila) OS=Mus musculus OX=10090 GN=Hook3 PE=2 SV=1	MOUSE	2
2309	3.65	3.81	13.29	10.40	tr|Q69ZP6|Q69ZP6_MOUSE	MKIAA1178 protein (Fragment) OS=Mus musculus OX=10090 GN=Cd2bp2 PE=2 SV=1	MOUSE	3
2310	3.65	3.78	21.00	7.21	tr|A7VMV2|A7VMV2_MOUSE	L-gulonate 3-dehydrogenase OS=Mus musculus OX=10090 GN=Cryl1 PE=1 SV=1	MOUSE	2
2311	3.65	3.76	9.80	2.34	tr|B2RY27|B2RY27_MOUSE	Taok3 protein OS=Mus musculus OX=10090 GN=Taok3 PE=2 SV=1	MOUSE	2
2312	3.64	3.81	16.79	7.36	tr|Q3UK41|Q3UK41_MOUSE	HDAg domain-containing protein OS=Mus musculus OX=10090 GN=Nelfa PE=2 SV=1	MOUSE	3
2313	3.62	3.89	20.16	9.68	tr|B1AWD9|B1AWD9_MOUSE	Clathrin light chain OS=Mus musculus OX=10090 GN=Clta PE=1 SV=1	MOUSE	3
2314	3.62	3.74	32.81	32.81	tr|Q14A47|Q14A47_MOUSE	Coiled-coil domain-containing protein 72 OS=Mus musculus OX=10090 GN=Tma7 PE=2 SV=1	MOUSE	3

2315	3.62	3.68	41.67	41.67	sp|Q9CQ91|NDUA3_MOUSE	NADH dehydrogenase [ubiquinone] 1 alpha subcomplex subunit 3 OS=Mus musculus OX=10090 GN=Ndufa3 PE=1 SV=1	MOUSE	2
2316	3.61	3.74	11.59	4.00	tr|G5E8I8|G5E8I8_MOUSE	Calcium homeostasis endoplasmic reticulum protein OS=Mus musculus OX=10090 GN=Cherp PE=1 SV=2	MOUSE	3
2317	3.6	4.03	10.97	6.20	tr|Q3TPD1|Q3TPD1_MOUSE	Uncharacterized protein (Fragment) OS=Mus musculus OX=10090 PE=2 SV=1	MOUSE	3
2318	3.6	3.68	6.00	4.18	tr|B2M0S2|B2M0S2_MOUSE	Secretory carrier-associated membrane protein OS=Mus musculus OX=10090 GN=Gm45927 PE=2 SV=1	MOUSE	2
2319	3.6	3.68	21.14	21.14	sp|Q8QZZ7|TPRKB_MOUSE	EKC/KEOPS complex subunit Tprkb OS=Mus musculus OX=10090 GN=Tprkb PE=1 SV=1	MOUSE	2
2320	3.59	3.71	33.82	19.32	sp|Q80ZW2|THEM6_MOUSE	Protein THEM6 OS=Mus musculus OX=10090 GN=Them6 PE=1 SV=1	MOUSE	3
2321	3.59	3.68	22.70	17.79	tr|Z4YJH1|Z4YJH1_MOUSE	Centrin-2 OS=Mus musculus OX=10090 GN=Cetn2 PE=1 SV=1	MOUSE	2
2322	3.59	3.66	11.39	11.39	sp|Q8R2U0|SEH1_MOUSE	Nucleoporin SEH1 OS=Mus musculus OX=10090 GN=Seh1l PE=2 SV=1	MOUSE	2
2323	3.58	3.78	2.28	1.61	tr|B2RY88|B2RY88_MOUSE	Eml5 protein OS=Mus musculus OX=10090 GN=Eml5 PE=2 SV=1	MOUSE	3
2324	3.58	3.76	12.82	11.22	sp|Q99JY8|PLPP3_MOUSE	Phospholipid phosphatase 3 OS=Mus musculus OX=10090 GN=Plpp3 PE=1 SV=1	MOUSE	3
2325	3.56	3.85	24.03	13.81	sp|Q9QXV1|CBX8_MOUSE	Chromobox protein homolog 8 OS=Mus musculus OX=10090 GN=Cbx8 PE=1 SV=1	MOUSE	3
2326	3.55	3.64	14.50	14.50	tr|Q3TLW5|Q3TLW5_MOUSE	Aquaporin-1 OS=Mus musculus OX=10090 GN=Aqp1 PE=2 SV=1	MOUSE	3
2327	3.55	3.63	16.60	16.60	sp|Q8BXV2|BRI3B_MOUSE	BRI3-binding protein OS=Mus musculus OX=10090 GN=Bri3bp PE=1 SV=1	MOUSE	4
2328	3.55	3.61	8.87	4.35	sp|Q8BH24|TM9S4_MOUSE	Transmembrane 9 superfamily member 4 OS=Mus musculus OX=10090 GN=Tm9sf4 PE=1 SV=1	MOUSE	2
2329	3.54	3.8	11.46	3.96	tr|E9Q616|E9Q616_MOUSE	AHNAK nucleoprotein (desmoyokin) OS=Mus musculus OX=10090 GN=Ahnak PE=1 SV=1	MOUSE	2
2330	3.53	3.62	26.39	11.81	sp|Q6PGH1|BUD31_MOUSE	Protein BUD31 homolog OS=Mus musculus OX=10090 GN=Bud31 PE=1 SV=2	MOUSE	2

2331	3.52	5.45	15.22	5.49	tr|A2A6U3|A2A6U3_MOUSE	Septin-9 OS=Mus musculus OX=10090 GN=Septin9 PE=1 SV=1	MOUSE	4
2332	3.52	3.59	6.11	6.11	tr|Q3TIJ0|Q3TIJ0_MOUSE	NOT2_3_5 domain-containing protein OS=Mus musculus OX=10090 GN=Cnot2 PE=2 SV=1	MOUSE	2
2333	3.51	3.59	6.39	6.39	tr|Q9ESU8|Q9ESU8_MOUSE	Neutral amino acid transporter ASCT1 OS=Mus musculus OX=10090 GN=Slc1a4 PE=2 SV=1	MOUSE	2
2334	3.5	3.61	14.91	10.55	tr|Q7TPS7|Q7TPS7_MOUSE	Nfyc protein OS=Mus musculus OX=10090 GN=Nfyc PE=2 SV=1	MOUSE	3
2335	3.5	3.61	11.97	11.97	tr|Q5U4G4|Q5U4G4_MOUSE	Ppap2c protein (Fragment) OS=Mus musculus OX=10090 GN=Plpp2 PE=2 SV=1	MOUSE	3
2336	3.5	3.58	5.59	1.66	tr|F8VPL1|F8VPL1_MOUSE	Voltage-dependent calcium channel subunit alpha-2/delta-4 OS=Mus musculus OX=10090 GN=Cacna2d4 PE=3 SV=1	MOUSE	2
2337	3.49	3.57	5.26	4.11	sp|Q05D44|IF2P_MOUSE	Eukaryotic translation initiation factor 5B OS=Mus musculus OX=10090 GN=Eif5b PE=1 SV=2	MOUSE	2
2338	3.48	3.78	11.92	4.83	sp|Q6PAV2|HERC4_MOUSE	Probable E3 ubiquitin-protein ligase HERC4 OS=Mus musculus OX=10090 GN=Herc4 PE=1 SV=2	MOUSE	4
2339	3.48	3.61	8.92	6.37	tr|Q4FJX5|Q4FJX5_MOUSE	Dhcr7 protein OS=Mus musculus OX=10090 GN=Dhcr7 PE=2 SV=1	MOUSE	2
2340	3.47	3.65	23.24	16.22	sp|Q9JKL4|NDUF3_MOUSE	NADH dehydrogenase [ubiquinone] 1 alpha subcomplex assembly factor 3 OS=Mus musculus OX=10090 GN=Ndufaf3 PE=1 SV=1	MOUSE	2
2341	3.47	3.54	27.06	13.76	sp|Q8VE95|CH082_MOUSE	UPF0598 protein C8orf82 homolog OS=Mus musculus OX=10090 PE=1 SV=1	MOUSE	2
2342	3.47	3.54	39.39	27.27	tr|Q8R472|Q8R472_MOUSE	HIG1 domain family member 1A, mitochondrial OS=Mus musculus OX=10090 GN=Higd1a PE=1 SV=1	MOUSE	2
2343	3.47	3.53	15.57	14.37	sp|Q9D328|NACHO_MOUSE	Novel acetylcholine receptor chaperone OS=Mus musculus OX=10090 GN=Tmem35a PE=1 SV=1	MOUSE	2
2344	3.46	3.57	7.49	1.81	tr|Q8R320|Q8R320_MOUSE	Nardilysin, N-arginine dibasic convertase, NRD convertase 1 OS=Mus musculus OX=10090 GN=Nrd1 PE=2 SV=1	MOUSE	2
2345	3.46	3.57	19.55	9.77	sp|A2ADY9|DDI2_MOUSE	Protein DDI1 homolog 2 OS=Mus musculus OX=10090 GN=Ddi2 PE=1 SV=1	MOUSE	2
2346	3.46	3.52	8.45	5.25	tr|B2RY30|B2RY30_MOUSE	Exosome complex component RRP45 OS=Mus musculus OX=10090 GN=Exosc9 PE=2 SV=1	MOUSE	2

2347	3.45	3.6	21.13	16.20	sp|Q9CPQ3|TOM22_MOUSE	Mitochondrial import receptor subunit TOM22 homolog OS=Mus musculus OX=10090 GN=Tomm22 PE=1 SV=3	MOUSE	2
2348	3.45	3.57	11.86	6.63	sp|P54731|FAF1_MOUSE	FAS-associated factor 1 OS=Mus musculus OX=10090 GN=Faf1 PE=1 SV=2	MOUSE	3
2349	3.45	3.56	39.53	20.00	tr|A0A1Y7VJZ2|A0A1Y7VJZ2_MOUSE	Maleylacetoacetate isomerase OS=Mus musculus OX=10090 GN=Gstz1 PE=1 SV=1	MOUSE	3
2350	3.44	3.51	19.47	19.47	sp|P61804|DAD1_MOUSE	Dolichyl-diphosphooligosaccharide--protein glycosyltransferase subunit DAD1 OS=Mus musculus OX=10090 GN=Dad1 PE=1 SV=3	MOUSE	2
2351	3.43	3.76	5.61	1.12	tr|G3X9H5|G3X9H5_MOUSE	Huntingtin OS=Mus musculus OX=10090 GN=Htt PE=1 SV=1	MOUSE	3
2352	3.43	3.69	28.36	8.21	tr|A0A0R4IZW8|A0A0R4IZW8_MOUSE	Calcium-activated neutral proteinase small subunit OS=Mus musculus OX=10090 GN=Capns1 PE=1 SV=1	MOUSE	3
2353	3.43	3.5	5.63	4.27	tr|Q8BZW3|Q8BZW3_MOUSE	Beta-galactosidase-1-like protein 2 OS=Mus musculus OX=10090 GN=Glb1l2 PE=1 SV=1	MOUSE	2
2354	3.42	3.48	6.96	6.96	tr|Q544L9|Q544L9_MOUSE	Uncharacterized protein OS=Mus musculus OX=10090 GN=Efnb1 PE=1 SV=1	MOUSE	2
2355	3.41	3.55	50.63	48.73	sp|Q8R1B5|CPLX3_MOUSE	Complexin-3 OS=Mus musculus OX=10090 GN=Cplx3 PE=1 SV=1	MOUSE	4
2356	3.4	3.51	17.31	7.31	tr|Q8K0I5|Q8K0I5_MOUSE	3-hydroxy-3-methylglutaryl coenzyme A synthase OS=Mus musculus OX=10090 GN=Hmgcs1 PE=2 SV=1	MOUSE	2
2357	3.39	3.57	15.05	4.76	tr|A0A0R4J079|A0A0R4J079_MOUSE	Golgi resident protein GCP60 OS=Mus musculus OX=10090 GN=Acbd3 PE=1 SV=1	MOUSE	2
2358	3.39	3.51	37.13	22.16	sp|Q8BHG9|CGBP1_MOUSE	CGG triplet repeat-binding protein 1 OS=Mus musculus OX=10090 GN=Cggbp1 PE=1 SV=1	MOUSE	3
2359	3.39	3.48	11.27	3.89	sp|O35405|PLD3_MOUSE	5'-3' exonuclease PLD3 OS=Mus musculus OX=10090 GN=Pld3 PE=1 SV=1	MOUSE	3
2360	3.39	3.47	8.92	6.02	tr|Q7TND9|Q7TND9_MOUSE	Dihydrolipoamide acetyltransferase component of pyruvate dehydrogenase complex OS=Mus musculus OX=10090 GN=Dbt PE=2 SV=1	MOUSE	2
2361	3.38	3.56	16.93	13.54	sp|Q3UPH1|PRRC1_MOUSE	Protein PRRC1 OS=Mus musculus OX=10090 GN=Prrc1 PE=1 SV=1	MOUSE	3
2362	3.38	3.49	20.31	15.33	tr|Q8BH40|Q8BH40_MOUSE	Syntaxin-7 OS=Mus musculus OX=10090 GN=Stx7 PE=1 SV=1	MOUSE	3

2363	3.38	3.44	29.93	23.36	sp|Q9D8S9|BOLA1_MOUSE	BolA-like protein 1 OS=Mus musculus OX=10090 GN=Bola1 PE=1 SV=1	MOUSE	2
2364	3.37	3.65	23.18	23.18	sp|Q9D6K8|FUND2_MOUSE	FUN14 domain-containing protein 2 OS=Mus musculus OX=10090 GN=Fundc2 PE=1 SV=1	MOUSE	3
2365	3.36	3.78	20.42	7.33	tr|E9PWY6|E9PWY6_MOUSE	2-amino-3-ketobutyrate coenzyme A ligase, mitochondrial OS=Mus musculus OX=10090 GN=Gcat PE=1 SV=1	MOUSE	2
2366	3.36	3.58	8.66	5.02	tr|Q8CDZ5|Q8CDZ5_MOUSE	Nucleoporin_C domain-containing protein OS=Mus musculus OX=10090 GN=Nup133 PE=2 SV=1	MOUSE	3
2367	3.36	3.49	20.58	6.03	tr|Q544K9|Q544K9_MOUSE	OMPdecase OS=Mus musculus OX=10090 GN=Umps PE=1 SV=1	MOUSE	3
2368	3.36	3.49	28.10	23.14	sp|Q91VR7|MLP3A_MOUSE	Microtubule-associated proteins 1A/1B light chain 3A OS=Mus musculus OX=10090 GN=Map1lc3a PE=1 SV=1	MOUSE	3
2369	3.33	3.55	18.13	9.36	tr|Q8BTD8|Q8BTD8_MOUSE	Uncharacterized protein (Fragment) OS=Mus musculus OX=10090 GN=Pitpnc1 PE=2 SV=1	MOUSE	3
2370	3.33	3.49	21.18	17.06	sp|Q9CQU0|TXD12_MOUSE	Thioredoxin domain-containing protein 12 OS=Mus musculus OX=10090 GN=Txndc12 PE=1 SV=1	MOUSE	2
2371	3.33	3.45	15.16	3.12	tr|G3X920|G3X920_MOUSE	Armadillo repeat-containing protein 8 OS=Mus musculus OX=10090 GN=Armc8 PE=1 SV=1	MOUSE	2
2372	3.33	3.4	9.18	9.18	tr|Q3UN47|Q3UN47_MOUSE	Endonuclease OS=Mus musculus OX=10090 GN=Endog PE=1 SV=1	MOUSE	2
2373	3.32	27.41	47.78	45.39	sp|Q9Z204-3|HNRPC_MOUSE	Isoform 3 of Heterogeneous nuclear ribonucleoproteins C1/C2 OS=Mus musculus OX=10090 GN=Hnrnpc	MOUSE	14
2374	3.29	9.58	27.88	17.95	sp|Q8VDS4|RPR1A_MOUSE	Regulation of nuclear pre-mRNA domain-containing protein 1A OS=Mus musculus OX=10090 GN=Rprd1a PE=1 SV=1	MOUSE	5
2375	3.29	3.51	27.72	8.42	tr|A0A494B9W4|A0A494B9W4_MOUSE	RNA-binding protein 4B (Fragment) OS=Mus musculus OX=10090 GN=Rbm4b PE=1 SV=1	MOUSE	2
2376	3.28	3.48	20.00	15.27	tr|Q6ZQJ2|Q6ZQJ2_MOUSE	Methionine aminopeptidase (Fragment) OS=Mus musculus OX=10090 GN=mKIAA0094 PE=3 SV=1	MOUSE	3
2377	3.28	3.47	7.34	3.75	sp|Q8R124|KLH36_MOUSE	Kelch-like protein 36 OS=Mus musculus OX=10090 GN=Klhl36 PE=1 SV=1	MOUSE	2
2378	3.28	3.4	24.29	21.05	sp|Q9CRD0|OCAD1_MOUSE	OCIA domain-containing protein 1 OS=Mus musculus OX=10090 GN=Ociad1 PE=1 SV=1	MOUSE	3

2379	3.27	3.34	4.58	4.58	tr|Q9CZU7|Q9CZU7_MOUSE	Uncharacterized protein OS=Mus musculus OX=10090 GN=Lamp2 PE=2 SV=1	MOUSE	2
2380	3.26	7.54	20.17	8.70	tr|Q3ULP9|Q3ULP9_MOUSE	Uncharacterized protein OS=Mus musculus OX=10090 GN=Fubp3 PE=2 SV=1	MOUSE	4
2381	3.26	3.48	13.30	6.12	tr|G5E896|G5E896_MOUSE	Enhancer of mRNA-decapping protein 4 OS=Mus musculus OX=10090 GN=Edc4 PE=1 SV=1	MOUSE	4
2382	3.26	3.39	20.23	10.69	sp|Q8CFE2|HPF1_MOUSE	Histone PARylation factor 1 OS=Mus musculus OX=10090 GN=Hpf1 PE=1 SV=1	MOUSE	2
2383	3.26	3.37	23.46	13.99	tr|F6V305|F6V305_MOUSE	Ubiquinol-cytochrome-c reductase complex assembly factor 1 (Fragment) OS=Mus musculus OX=10090 GN=Uqcc1 PE=1 SV=1	MOUSE	3
2384	3.25	10.36	10.20	9.26	sp|Q02257|PLAK_MOUSE	Junction plakoglobin OS=Mus musculus OX=10090 GN=Jup PE=1 SV=3	MOUSE	6
2385	3.25	3.41	21.66	12.10	sp|Q9CPX6|ATG3_MOUSE	Ubiquitin-like-conjugating enzyme ATG3 OS=Mus musculus OX=10090 GN=Atg3 PE=1 SV=1	MOUSE	3
2386	3.25	3.4	7.28	5.40	tr|A0A1S6GWG8|A0A1S6GWG8_MOUSE	Uncharacterized protein OS=Mus musculus OX=10090 GN=Dnaja4 PE=2 SV=1	MOUSE	2
2387	3.25	3.36	10.79	7.42	sp|Q8C8Y5|SAMD7_MOUSE	Sterile alpha motif domain-containing protein 7 OS=Mus musculus OX=10090 GN=Samd7 PE=2 SV=4	MOUSE	3
2388	3.25	3.31	31.65	31.65	sp|P60603|ROMO1_MOUSE	Reactive oxygen species modulator 1 OS=Mus musculus OX=10090 GN=Romo1 PE=2 SV=1	MOUSE	3
2389	3.24	3.36	14.12	4.39	tr|Q8R378|Q8R378_MOUSE	Leucine-rich repeat protein SHOC-2 OS=Mus musculus OX=10090 GN=Shoc2 PE=2 SV=1	MOUSE	2
2390	3.23	3.44	11.11	9.47	sp|Q7TSY6|CELF4_MOUSE	CUGBP Elav-like family member 4 OS=Mus musculus OX=10090 GN=Celf4 PE=1 SV=2	MOUSE	3
2391	3.23	3.33	39.19	17.57	sp|Q61012|GBG1_MOUSE	Guanine nucleotide-binding protein G(T) subunit gamma-T1 OS=Mus musculus OX=10090 GN=Gngt1 PE=1 SV=3	MOUSE	2
2392	3.23	3.29	32.35	32.35	sp|O08997|ATOX1_MOUSE	Copper transport protein ATOX1 OS=Mus musculus OX=10090 GN=Atox1 PE=1 SV=1	MOUSE	2
2393	3.21	3.35	10.23	2.78	tr|Q3UDS1|Q3UDS1_MOUSE	Uncharacterized protein OS=Mus musculus OX=10090 GN=Cpsf3 PE=2 SV=1	MOUSE	2
2394	3.18	4.23	10.23	6.86	tr|Q5CCJ9|Q5CCJ9_MOUSE	Orfx protein OS=Mus musculus OX=10090 GN=Brd3 PE=2 SV=2	MOUSE	4

2395	3.18	3.37	27.41	27.41	tr|A8Y5N8|A8Y5N8_MOUSE	Pleckstrin homology domain-containing family F member 2 (Fragment) OS=Mus musculus OX=10090 GN=Plekhf2 PE=1 SV=1	MOUSE	4
2396	3.18	3.34	6.45	1.10	tr|F8VQ05|F8VQ05_MOUSE	FRY-like transcription coactivator OS=Mus musculus OX=10090 GN=Fryl PE=1 SV=1	MOUSE	2
2397	3.18	3.24	6.13	5.92	sp|Q8CHK3|MBOA7_MOUSE	Lysophospholipid acyltransferase 7 OS=Mus musculus OX=10090 GN=Mboat7 PE=1 SV=1	MOUSE	2
2398	3.17	3.39	7.02	5.76	sp|P09055|ITB1_MOUSE	Integrin beta-1 OS=Mus musculus OX=10090 GN=Itgb1 PE=1 SV=1	MOUSE	3
2399	3.16	3.24	15.72	7.24	tr|D3Z780|D3Z780_MOUSE	Translation initiation factor eIF-2B subunit delta OS=Mus musculus OX=10090 GN=Eif2b4 PE=1 SV=1	MOUSE	2
2400	3.15	3.34	16.58	7.67	tr|Q540F5|Q540F5_MOUSE	CTP:phosphoethanolamine cytidylyltransferase OS=Mus musculus OX=10090 PE=3 SV=1	MOUSE	3
2401	3.15	3.29	11.72	11.72	tr|G3X926|G3X926_MOUSE	Ribosome production factor 2 homolog OS=Mus musculus OX=10090 GN=Rpf2 PE=1 SV=1	MOUSE	2
2402	3.15	3.28	33.60	16.00	sp|Q9JHS3|LTOR2_MOUSE	Ragulator complex protein LAMTOR2 OS=Mus musculus OX=10090 GN=Lamtor2 PE=1 SV=1	MOUSE	2
2403	3.15	3.21	22.07	14.83	sp|Q8BL95|CF298_MOUSE	Cilia- and flagella-associated protein 298 OS=Mus musculus OX=10090 GN=Cfap298 PE=1 SV=1	MOUSE	2
2404	3.15	3.21	19.23	14.29	tr|Q3UFD0|Q3UFD0_MOUSE	Cytochrome c oxidase assembly protein COX16 homolog, mitochondrial OS=Mus musculus OX=10090 PE=2 SV=1	MOUSE	2
2405	3.14	9.28	43.03	34.55	tr|Q5M9K7|Q5M9K7_MOUSE	Ribosomal protein S10 OS=Mus musculus OX=10090 GN=Rps10 PE=1 SV=1	MOUSE	5
2406	3.14	3.3	12.92	3.81	tr|Q8C027|Q8C027_MOUSE	Vacuolar protein sorting-associated protein 11 homolog OS=Mus musculus OX=10090 GN=Vps11 PE=2 SV=1	MOUSE	2
2407	3.14	3.22	25.60	16.80	sp|P59708|SF3B6_MOUSE	Splicing factor 3B subunit 6 OS=Mus musculus OX=10090 GN=Sf3b6 PE=1 SV=1	MOUSE	2
2408	3.13	3.23	15.22	12.73	tr|Q9D6D0|Q9D6D0_MOUSE	Solute carrier family 25, member 27 OS=Mus musculus OX=10090 GN=Slc25a27 PE=1 SV=1	MOUSE	3
2409	3.13	3.19	4.80	4.80	sp|Q9D4C9|CLVS1_MOUSE	Clavesin-1 OS=Mus musculus OX=10090 GN=Clvs1 PE=1 SV=1	MOUSE	2
2410	3.12	3.27	9.55	3.60	sp|Q9Z1K5|ARI1_MOUSE	E3 ubiquitin-protein ligase ARIH1 OS=Mus musculus OX=10090 GN=Arih1 PE=1 SV=3	MOUSE	2

2411	3.11	3.35	9.42	2.64	tr|Q7TQD7|Q7TQD7_MOUSE	Myo1b protein OS=Mus musculus OX=10090 GN=Myo1b PE=1 SV=1	MOUSE	2
2412	3.11	3.18	7.63	4.88	sp|Q9JJY3|NSMA2_MOUSE	Sphingomyelin phosphodiesterase 3 OS=Mus musculus OX=10090 GN=Smpd3 PE=1 SV=1	MOUSE	2
2413	3.1	34.9	92.06	74.60	sp|Q64525|H2B2B_MOUSE	Histone H2B type 2-B OS=Mus musculus OX=10090 GN=Hist2h2bb PE=1 SV=3	MOUSE	62
2414	3.1	3.24	5.99	3.38	tr|E9QP59|E9QP59_MOUSE	Inner nuclear membrane protein Man1 OS=Mus musculus OX=10090 GN=Lemd3 PE=1 SV=1	MOUSE	3
2415	3.09	3.23	11.62	6.14	tr|Q4FJN2|Q4FJN2_MOUSE	Peptidylprolyl isomerase OS=Mus musculus OX=10090 GN=Fkbp5 PE=1 SV=1	MOUSE	3
2416	3.08	3.26	8.07	1.89	tr|A0A1L1SV73|A0A1L1SV73_MOUSE	Ubiquitin carboxyl-terminal hydrolase 47 OS=Mus musculus OX=10090 GN=Usp47 PE=1 SV=1	MOUSE	2
2417	3.08	3.17	16.44	10.41	tr|Q9CT17|Q9CT17_MOUSE	RRM domain-containing protein (Fragment) OS=Mus musculus OX=10090 GN=Srsf11 PE=2 SV=1	MOUSE	2
2418	3.08	3.14	48.53	48.53	sp|Q6W8Q3|PC4L1_MOUSE	Purkinje cell protein 4-like protein 1 OS=Mus musculus OX=10090 GN=Pcp4l1 PE=1 SV=1	MOUSE	2
2419	3.07	17.95	37.12	20.16	tr|A2A5Y6|A2A5Y6_MOUSE	Microtubule-associated protein OS=Mus musculus OX=10090 GN=Mapt PE=1 SV=1	MOUSE	16
2420	3.06	3.18	5.22	1.65	tr|B9EKS2|B9EKS2_MOUSE	Jumonji domain containing 1B OS=Mus musculus OX=10090 GN=Kdm3b PE=1 SV=1	MOUSE	2
2421	3.06	3.17	8.76	7.30	sp|Q8K2Q7|BROX_MOUSE	BRO1 domain-containing protein BROX OS=Mus musculus OX=10090 GN=Brox PE=1 SV=1	MOUSE	2
2422	3.06	3.16	13.49	5.55	tr|Q91V35|Q91V35_MOUSE	Receptor-type tyrosine-protein phosphatase alpha OS=Mus musculus OX=10090 GN=Ptpra PE=1 SV=1	MOUSE	2
2423	3.06	3.15	15.78	5.54	sp|Q3TKY6|CWC27_MOUSE	Spliceosome-associated protein CWC27 homolog OS=Mus musculus OX=10090 GN=Cwc27 PE=1 SV=1	MOUSE	2
2424	3.06	3.12	40.18	40.18	tr|Q9DCW5|Q9DCW5_MOUSE	Cytochrome c oxidase subunit OS=Mus musculus OX=10090 GN=Cox6a1 PE=1 SV=1	MOUSE	2
2425	3.05	3.12	21.44	5.19	tr|Q3UER8|Q3UER8_MOUSE	Fibrinogen gamma chain OS=Mus musculus OX=10090 GN=Fgg PE=1 SV=1	MOUSE	2
2426	3.04	3.19	4.92	2.83	sp|Q9EPE9|AT131_MOUSE	Endoplasmic reticulum transmembrane helix translocase OS=Mus musculus OX=10090 GN=Atp13a1 PE=1 SV=2	MOUSE	2
2427	3.02	3.25	21.50	8.25	tr|Q3TZ02|Q3TZ02_MOUSE	Cytohesin-1 OS=Mus musculus OX=10090 GN=Cyth1 PE=1 SV=1	MOUSE	3

2428	3.02	3.22	9.56	6.04	sp|Q8BHB9|CLIC6_MOUSE	Chloride intracellular channel protein 6 OS=Mus musculus OX=10090 GN=Clic6 PE=1 SV=1	MOUSE	3
2429	3.02	3.15	25.00	5.94	tr|D3Z4U0|D3Z4U0_MOUSE	Zinc finger Ran-binding domain-containing protein 2 OS=Mus musculus OX=10090 GN=Zranb2 PE=1 SV=2	MOUSE	2
2430	3.02	3.1	15.53	7.95	sp|Q9EST4|PSMG2_MOUSE	Proteasome assembly chaperone 2 OS=Mus musculus OX=10090 GN=Psmg2 PE=1 SV=1	MOUSE	2
2431	3.02	3.09	38.06	26.87	sp|P84086|CPLX2_MOUSE	Complexin-2 OS=Mus musculus OX=10090 GN=Cplx2 PE=1 SV=1	MOUSE	2
2432	3	12.88	54.89	42.93	tr|Q52L50|Q52L50_MOUSE	RAS related protein 1b OS=Mus musculus OX=10090 GN=Rap1b PE=1 SV=1	MOUSE	8
2433	3	3.24	40.89	19.11	tr|Q3UGM5|Q3UGM5_MOUSE	Uncharacterized protein OS=Mus musculus OX=10090 GN=Mob4 PE=2 SV=1	MOUSE	3
2434	2.99	3.18	9.08	1.61	tr|I1E4X5|I1E4X5_MOUSE	VPS35 endosomal protein-sorting factor-like OS=Mus musculus OX=10090 GN=Vps35l PE=1 SV=1	MOUSE	1
2435	2.97	3.06	18.78	11.09	sp|Q9Z2L7|CRLF3_MOUSE	Cytokine receptor-like factor 3 OS=Mus musculus OX=10090 GN=Crlf3 PE=1 SV=1	MOUSE	2
2436	2.97	3.05	15.91	10.23	tr|Q8VC93|Q8VC93_MOUSE	Eri3 protein (Fragment) OS=Mus musculus OX=10090 GN=Eri3 PE=2 SV=2	MOUSE	2
2437	2.96	3.11	8.57	5.34	tr|B1AXN9|B1AXN9_MOUSE	Non-specific serine/threonine protein kinase OS=Mus musculus OX=10090 GN=Rps6ka3 PE=1 SV=1	MOUSE	3
2438	2.96	3.06	19.58	10.00	sp|O09131|GSTO1_MOUSE	Glutathione S-transferase omega-1 OS=Mus musculus OX=10090 GN=Gsto1 PE=1 SV=2	MOUSE	2
2439	2.96	3.04	19.89	14.20	tr|E9Q933|E9Q933_MOUSE	Transmembrane protein 11, mitochondrial OS=Mus musculus OX=10090 GN=Tmem11 PE=1 SV=1	MOUSE	2
2440	2.95	19.36	80.00	56.67	tr|Q58E57|Q58E57_MOUSE	High mobility group nucleosomal binding domain 2 OS=Mus musculus OX=10090 GN=Hmgn2 PE=2 SV=1	MOUSE	59
2441	2.95	11.99	13.37	5.33	tr|E9PV14|E9PV14_MOUSE	Band 4.1-like protein 1 OS=Mus musculus OX=10090 GN=Epb41l1 PE=1 SV=2	MOUSE	7
2442	2.95	3.19	6.43	1.51	sp|Q62383|SPT6H_MOUSE	Transcription elongation factor SPT6 OS=Mus musculus OX=10090 GN=Supt6h PE=1 SV=2	MOUSE	2
2443	2.95	3.01	7.63	7.63	tr|Q80ZU1|Q80ZU1_MOUSE	WD repeat domain 68 OS=Mus musculus OX=10090 GN=Dcaf7 PE=2 SV=1	MOUSE	2
2444	2.94	4.12	9.36	5.40	sp|Q5QNQ6|OSBP2_MOUSE	Oxysterol-binding protein 2 OS=Mus musculus OX=10090 GN=Osbp2 PE=1 SV=1	MOUSE	3

2445	2.93	3.06	9.15	5.35	tr|Q8C541|Q8C541_MOUSE	Fatty acid amide hydrolase OS=Mus musculus OX=10090 GN=Faah PE=2 SV=1	MOUSE	2
2446	2.92	3.36	57.53	16.67	sp|Q9Z0Y1|DCTN3_MOUSE	Dynactin subunit 3 OS=Mus musculus OX=10090 GN=Dctn3 PE=1 SV=2	MOUSE	3
2447	2.92	3.01	16.63	5.69	sp|A6H630|ARMT1_MOUSE	Damage-control phosphatase ARMT1 OS=Mus musculus OX=10090 GN=Armt1 PE=1 SV=1	MOUSE	2
2448	2.92	3.01	5.63	2.68	tr|A0A0A6YXH3|A0A0A6YXH3_MOUSE	Oxysterol-binding protein OS=Mus musculus OX=10090 GN=Osbpl9 PE=1 SV=1	MOUSE	2
2449	2.92	2.97	11.83	6.80	sp|Q8R4N0|CLYBL_MOUSE	Citramalyl-CoA lyase, mitochondrial OS=Mus musculus OX=10090 GN=Clybl PE=1 SV=2	MOUSE	2
2450	2.91	5	6.51	3.37	tr|C7G3P1|C7G3P1_MOUSE	MKIAA0654 protein (Fragment) OS=Mus musculus OX=10090 GN=Ppfia3 PE=2 SV=1	MOUSE	3
2451	2.91	2.98	16.41	12.82	sp|P62342|SELT_MOUSE	Thioredoxin reductase-like selenoprotein T OS=Mus musculus OX=10090 GN=Selenot PE=1 SV=2	MOUSE	2
2452	2.9	9.23	29.13	18.93	tr|Q8CCG5|Q8CCG5_MOUSE	Small monomeric GTPase OS=Mus musculus OX=10090 GN=Ralb PE=2 SV=1	MOUSE	5
2453	2.9	3.09	6.27	3.84	tr|G5DDB7|G5DDB7_MOUSE	Phosphoinositide phospholipase C OS=Mus musculus OX=10090 GN=Plcd1 PE=1 SV=1	MOUSE	3
2454	2.89	2.99	13.94	6.49	tr|A2AD85|A2AD85_MOUSE	Protein kinase domain-containing protein OS=Mus musculus OX=10090 GN=Stk26 PE=1 SV=1	MOUSE	2
2455	2.88	2.95	10.13	3.49	sp|Q8VHP6|CDHR1_MOUSE	Cadherin-related family member 1 OS=Mus musculus OX=10090 GN=Cdhr1 PE=1 SV=1	MOUSE	2
2456	2.88	2.95	8.02	4.61	sp|P70303|PYRG2_MOUSE	CTP synthase 2 OS=Mus musculus OX=10090 GN=Ctps2 PE=1 SV=1	MOUSE	2
2457	2.88	2.94	6.88	5.40	tr|Q9DC13|Q9DC13_MOUSE	Uncharacterized protein OS=Mus musculus OX=10090 GN=Lamp1 PE=2 SV=1	MOUSE	2
2458	2.88	2.94	25.70	25.14	sp|Q99LW6|YAF2_MOUSE	YY1-associated factor 2 OS=Mus musculus OX=10090 GN=Yaf2 PE=1 SV=1	MOUSE	2
2459	2.87	2.98	24.66	16.44	tr|A0A1Y7VK19|A0A1Y7VK19_MOUSE	Iron-sulfur cluster assembly 2 homolog, mitochondrial (Fragment) OS=Mus musculus OX=10090 GN=Isca2 PE=1 SV=1	MOUSE	2
2460	2.85	3.03	9.61	6.86	sp|Q8BTS4|NUP54_MOUSE	Nuclear pore complex protein Nup54 OS=Mus musculus OX=10090 GN=Nup54 PE=1 SV=1	MOUSE	3

2461	2.85	2.94	12.37	4.26	tr|A2RS23|A2RS23_MOUSE	Peptidase D OS=Mus musculus OX=10090 GN=Pepd PE=1 SV=1	MOUSE	2
2462	2.84	3.02	37.10	31.45	tr|Q542I7|Q542I7_MOUSE	Late endosomal/lysosomal adaptor and MAPK and MTOR activator 3 OS=Mus musculus OX=10090 GN=Lamtor3 PE=1 SV=1	MOUSE	2
2463	2.84	2.93	42.03	21.01	tr|A2A8E2|A2A8E2_MOUSE	CXXC motif containing zinc binding protein OS=Mus musculus OX=10090 GN=Czib PE=1 SV=1	MOUSE	2
2464	2.84	2.9	31.77	13.54	tr|Z4YL87|Z4YL87_MOUSE	Pre-rRNA-processing protein TSR2 homolog OS=Mus musculus OX=10090 GN=Tsr2 PE=1 SV=1	MOUSE	2
2465	2.83	3.01	44.24	11.87	tr|Q3T9H8|Q3T9H8_MOUSE	Uncharacterized protein OS=Mus musculus OX=10090 GN=Smug1 PE=2 SV=1	MOUSE	2
2466	2.82	2.99	36.49	28.38	tr|A0A158SIT5|A0A158SIT5_MOUSE	L antigen family, member 3 OS=Mus musculus OX=10090 GN=Lage3 PE=3 SV=1	MOUSE	3
2467	2.82	2.97	12.58	3.81	tr|B3V099|B3V099_MOUSE	BetaCstF-64 variant 1 OS=Mus musculus OX=10090 GN=Cstf2 PE=2 SV=1	MOUSE	2
2468	2.82	2.88	15.97	15.97	tr|A0A1Y7VMB1|A0A1Y7VMB1_MOUSE	tRNA (guanine(37)-N1)-methyltransferase (Fragment) OS=Mus musculus OX=10090 GN=Trmt5 PE=1 SV=1	MOUSE	2
2469	2.81	2.96	8.16	3.34	tr|Q9CW64|Q9CW64_MOUSE	Thioredoxin domain-containing protein (Fragment) OS=Mus musculus OX=10090 GN=Nhlrc2 PE=2 SV=1	MOUSE	2
2470	2.81	2.89	9.30	3.27	sp|Q8VCN5|CGL_MOUSE	Cystathionine gamma-lyase OS=Mus musculus OX=10090 GN=Cth PE=1 SV=1	MOUSE	1
2471	2.81	2.87	30.50	19.00	sp|Q9CRA7|ATP5S_MOUSE	ATP synthase subunit s, mitochondrial OS=Mus musculus OX=10090 GN=Dmac2l PE=1 SV=1	MOUSE	2
2472	2.8	3.1	9.20	4.93	sp|Q8BYA0|TBCD_MOUSE	Tubulin-specific chaperone D OS=Mus musculus OX=10090 GN=Tbcd PE=1 SV=1	MOUSE	5
2473	2.8	3.02	8.71	3.23	tr|Q3TI84|Q3TI84_MOUSE	TPR_REGION domain-containing protein OS=Mus musculus OX=10090 GN=Cdc16 PE=1 SV=1	MOUSE	2
2474	2.8	2.95	20.54	7.86	sp|Q9D020|5NT3A_MOUSE	Cytosolic 5'-nucleotidase 3A OS=Mus musculus OX=10090 GN=Nt5c3a PE=1 SV=4	MOUSE	2
2475	2.8	2.95	14.45	7.81	sp|Q6PDY2|AEDO_MOUSE	2-aminoethanethiol dioxygenase OS=Mus musculus OX=10090 GN=Ado PE=1 SV=2	MOUSE	2
2476	2.8	2.85	32.14	32.14	tr|E0CY07|E0CY07_MOUSE	Protein kish OS=Mus musculus OX=10090 GN=Tmem167 PE=1 SV=1	MOUSE	2

2477	2.79	2.88	6.50	1.94	tr|A0A571BG95|A0A571BG95_MOUSE	Active breakpoint cluster region-related protein OS=Mus musculus OX=10090 GN=Abr PE=1 SV=1	MOUSE	2
2478	2.79	2.86	13.78	7.40	sp|O54724|CAVN1_MOUSE	Caveolae-associated protein 1 OS=Mus musculus OX=10090 GN=Cavin1 PE=1 SV=1	MOUSE	2
2479	2.78	2.84	16.46	6.21	tr|D3YVC9|D3YVC9_MOUSE	Graves disease carrier protein homolog (Fragment) OS=Mus musculus OX=10090 GN=Slc25a16 PE=1 SV=1	MOUSE	2
2480	2.78	2.83	11.32	10.69	tr|Q9D6J9|Q9D6J9_MOUSE	Succinate dehydrogenase [ubiquinone] cytochrome b small subunit OS=Mus musculus OX=10090 GN=Sdhd PE=2 SV=1	MOUSE	2
2481	2.77	2.85	37.14	17.14	sp|Q9CZG9|PDZ11_MOUSE	PDZ domain-containing protein 11 OS=Mus musculus OX=10090 GN=Pdzd11 PE=1 SV=1	MOUSE	1
2482	2.77	2.82	4.43	4.43	sp|Q8R1T1|CHMP7_MOUSE	Charged multivesicular body protein 7 OS=Mus musculus OX=10090 GN=Chmp7 PE=1 SV=1	MOUSE	2
2483	2.76	2.85	7.36	0.97	tr|Q3TT06|Q3TT06_MOUSE	Nuclear pore complex protein OS=Mus musculus OX=10090 GN=Nup107 PE=2 SV=1	MOUSE	1
2484	2.76	2.84	24.78	8.70	sp|Q3UUI3|THEM4_MOUSE	Acyl-coenzyme A thioesterase THEM4 OS=Mus musculus OX=10090 GN=Them4 PE=1 SV=1	MOUSE	2
2485	2.74	4.87	15.64	15.64	sp|Q505D7|OPA3_MOUSE	Optic atrophy 3 protein homolog OS=Mus musculus OX=10090 GN=Opa3 PE=1 SV=1	MOUSE	3
2486	2.74	3.06	24.02	11.35	sp|Q9JHS9|CWC15_MOUSE	Spliceosome-associated protein CWC15 homolog OS=Mus musculus OX=10090 GN=Cwc15 PE=1 SV=1	MOUSE	2
2487	2.74	2.89	9.04	4.27	sp|Q8BU14|SEC62_MOUSE	Translocation protein SEC62 OS=Mus musculus OX=10090 GN=Sec62 PE=1 SV=1	MOUSE	2
2488	2.74	2.79	5.53	4.28	tr|Q3UF16|Q3UF16_MOUSE	Procollagen-proline 4-dioxygenase OS=Mus musculus OX=10090 GN=P4ha1 PE=2 SV=1	MOUSE	2
2489	2.73	10.08	24.40	15.01	tr|Q80Y51|Q80Y51_MOUSE	ELAV-like protein OS=Mus musculus OX=10090 GN=Elavl2 PE=1 SV=1	MOUSE	6
2490	2.73	8.02	17.79	7.62	sp|Q3UMU9|HDGR2_MOUSE	Hepatoma-derived growth factor-related protein 2 OS=Mus musculus OX=10090 GN=Hdgfl2 PE=1 SV=1	MOUSE	6
2491	2.73	4.84	5.85	3.61	sp|Q8C129|LCAP_MOUSE	Leucyl-cystinyl aminopeptidase OS=Mus musculus OX=10090 GN=Lnpep PE=1 SV=1	MOUSE	3
2492	2.73	2.8	28.25	15.25	tr|Q3ULK8|Q3ULK8_MOUSE	Uncharacterized protein OS=Mus musculus OX=10090 GN=Cnbp PE=1 SV=1	MOUSE	2

2493	2.71	2.99	13.75	3.66	sp|Q8CCJ3|UFL1_MOUSE	E3 UFM1-protein ligase 1 OS=Mus musculus OX=10090 GN=Ufl1 PE=1 SV=2	MOUSE	2
2494	2.71	2.78	5.04	4.04	tr|Q8C9W5|Q8C9W5_MOUSE	Uncharacterized protein (Fragment) OS=Mus musculus OX=10090 GN=Erap1 PE=2 SV=1	MOUSE	2
2495	2.71	2.76	17.91	11.34	sp|Q8K409|DPOLB_MOUSE	DNA polymerase beta OS=Mus musculus OX=10090 GN=Polb PE=1 SV=3	MOUSE	2
2496	2.7	2.77	5.73	3.34	tr|Q3UGC1|Q3UGC1_MOUSE	Uncharacterized protein OS=Mus musculus OX=10090 GN=Strip1 PE=2 SV=1	MOUSE	2
2497	2.69	3.48	25.49	25.49	sp|P62311|LSM3_MOUSE	U6 snRNA-associated Sm-like protein LSm3 OS=Mus musculus OX=10090 GN=Lsm3 PE=1 SV=2	MOUSE	3
2498	2.68	2.8	10.84	5.05	sp|Q8K2T8|PAF1_MOUSE	RNA polymerase II-associated factor 1 homolog OS=Mus musculus OX=10090 GN=Paf1 PE=1 SV=1	MOUSE	2
2499	2.68	2.75	7.85	2.99	tr|Q3TCW5|Q3TCW5_MOUSE	Protein kinase domain-containing protein OS=Mus musculus OX=10090 GN=Nrbp1 PE=2 SV=1	MOUSE	1
2500	2.67	2.81	12.13	8.52	sp|Q9DCV4|RMD1_MOUSE	Regulator of microtubule dynamics protein 1 OS=Mus musculus OX=10090 GN=Rmdn1 PE=1 SV=2	MOUSE	2
2501	2.66	3.08	15.62	6.29	tr|Q3U232|Q3U232_MOUSE	Coronin OS=Mus musculus OX=10090 GN=Coro1a PE=2 SV=1	MOUSE	1
2502	2.66	2.74	10.44	3.70	sp|O35385|PPE2_MOUSE	Serine/threonine-protein phosphatase with EF-hands 2 OS=Mus musculus OX=10090 GN=Ppef2 PE=2 SV=1	MOUSE	3
2503	2.66	2.73	15.34	6.35	tr|Q4FJQ6|Q4FJQ6_MOUSE	Serpinb6a protein OS=Mus musculus OX=10090 GN=Serpinb6a PE=1 SV=1	MOUSE	2
2504	2.66	2.71	17.57	6.71	sp|Q80X95|RRAGA_MOUSE	Ras-related GTP-binding protein A OS=Mus musculus OX=10090 GN=Rraga PE=1 SV=1	MOUSE	2
2505	2.65	2.79	10.74	9.20	sp|Q9DCJ1|LST8_MOUSE	Target of rapamycin complex subunit LST8 OS=Mus musculus OX=10090 GN=Mlst8 PE=1 SV=1	MOUSE	2
2506	2.65	2.7	28.32	17.70	sp|Q9D0A3|ARPIN_MOUSE	Arpin OS=Mus musculus OX=10090 GN=Arpin PE=1 SV=1	MOUSE	3
2507	2.64	2.84	4.73	0.93	tr|Q8CHA4|Q8CHA4_MOUSE	MKIAA1329 protein (Fragment) OS=Mus musculus OX=10090 GN=Sorcs2 PE=2 SV=1	MOUSE	1
2508	2.63	2.76	3.99	2.00	tr|A0A0N4SW93|A0A0N4SW93_MOUSE	C2 domain-containing protein 5 OS=Mus musculus OX=10090 GN=C2cd5 PE=1 SV=1	MOUSE	2
2509	2.63	2.71	14.09	4.29	tr|Q7TPT3|Q7TPT3_MOUSE	Exocyst complex component 7 OS=Mus musculus OX=10090 GN=Exoc7 PE=2 SV=1	MOUSE	2

2510	2.63	2.67	7.04	7.04	tr|Q8BQV8|Q8BQV8_MOUSE	Sulfotransferase OS=Mus musculus OX=10090 GN=Sult4a1 PE=2 SV=1	MOUSE	2
2511	2.62	2.79	17.45	12.50	sp|Q8C5W3|TBCEL_MOUSE	Tubulin-specific chaperone cofactor E-like protein OS=Mus musculus OX=10090 GN=Tbcel PE=1 SV=1	MOUSE	3
2512	2.62	2.74	18.06	10.56	sp|Q8BTZ7|GMPPB_MOUSE	Mannose-1-phosphate guanyltransferase beta OS=Mus musculus OX=10090 GN=Gmppb PE=1 SV=1	MOUSE	2
2513	2.62	2.7	3.38	2.67	tr|D3Z4I0|D3Z4I0_MOUSE	GPI ethanolamine phosphate transferase 2 OS=Mus musculus OX=10090 GN=Pigg PE=1 SV=1	MOUSE	2
2514	2.61	5.1	14.26	8.63	tr|Q3UJS6|Q3UJS6_MOUSE	Uncharacterized protein OS=Mus musculus OX=10090 GN=Cpne3 PE=2 SV=1	MOUSE	5
2515	2.61	2.67	12.76	1.51	tr|Q8K366|Q8K366_MOUSE	Exosc10 protein OS=Mus musculus OX=10090 GN=Exosc10 PE=1 SV=1	MOUSE	1
2516	2.6	107.83	78.11	71.31	sp|O08599-2|STXB1_MOUSE	Isoform 2 of Syntaxin-binding protein 1 OS=Mus musculus OX=10090 GN=Stxbp1	MOUSE	68
2517	2.6	56.5	49.02	49.02	tr|Q0VG47|Q0VG47_MOUSE	Heterogeneous nuclear ribonucleoprotein A3 OS=Mus musculus OX=10090 GN=Hnrnpa3 PE=2 SV=1	MOUSE	38
2518	2.6	2.76	8.15	5.17	sp|Q8K0C4|CP51A_MOUSE	Lanosterol 14-alpha demethylase OS=Mus musculus OX=10090 GN=Cyp51a1 PE=1 SV=1	MOUSE	2
2519	2.6	2.66	23.31	14.83	tr|A2AER8|A2AER8_MOUSE	Polyglutamine tract-binding protein 1 (Fragment) OS=Mus musculus OX=10090 GN=Pqbp1 PE=1 SV=1	MOUSE	2
2520	2.6	2.65	8.50	3.96	tr|Q8CFR5|Q8CFR5_MOUSE	Dystrobrevin OS=Mus musculus OX=10090 GN=Dtna PE=1 SV=1	MOUSE	2
2521	2.59	2.65	36.99	15.61	sp|Q9D735|TRIR_MOUSE	Telomerase RNA component interacting RNase OS=Mus musculus OX=10090 GN=Trir PE=1 SV=1	MOUSE	1
2522	2.59	2.64	5.78	1.86	sp|Q80TR8|DCAF1_MOUSE	DDB1- and CUL4-associated factor 1 OS=Mus musculus OX=10090 GN=Dcaf1 PE=1 SV=4	MOUSE	2
2523	2.59	2.63	36.36	36.36	tr|A0A0G2JFK6|A0A0G2JFK6_MOUSE	Coiled-coil domain-containing protein 12 (Fragment) OS=Mus musculus OX=10090 GN=Ccdc12 PE=1 SV=1	MOUSE	2
2524	2.58	4.78	24.40	5.57	tr|Q3UE40|Q3UE40_MOUSE	Uncharacterized protein OS=Mus musculus OX=10090 GN=Gna13 PE=2 SV=1	MOUSE	2
2525	2.58	2.76	6.60	2.04	tr|B7ZN33|B7ZN33_MOUSE	Nisch protein (Fragment) OS=Mus musculus OX=10090 GN=Nisch PE=2 SV=1	MOUSE	2
2526	2.58	2.73	18.35	13.61	sp|O70209|PDLI3_MOUSE	PDZ and LIM domain protein 3 OS=Mus musculus OX=10090 GN=Pdlim3 PE=1 SV=1	MOUSE	2

2527	2.57	2.67	8.02	6.11	tr|A0A0A6YXC8|A0A0A6YXC8_MOUSE	Protein enabled homolog OS=Mus musculus OX=10090 GN=Enah PE=1 SV=1	MOUSE	2
2528	2.56	2.66	21.39	15.03	tr|Q8K3J2|Q8K3J2_MOUSE	Bcl2-associated X protein kappa isoform OS=Mus musculus OX=10090 GN=Bax PE=2 SV=1	MOUSE	2
2529	2.55	2.74	8.91	1.96	sp|Q99PU8-3|DHX30_MOUSE	Isoform 3 of ATP-dependent RNA helicase DHX30 OS=Mus musculus OX=10090 GN=Dhx30	MOUSE	2
2530	2.55	2.59	24.74	11.05	sp|Q8K3C3|LZIC_MOUSE	Protein LZIC OS=Mus musculus OX=10090 GN=Lzic PE=1 SV=1	MOUSE	2
2531	2.54	6.32	16.53	7.35	sp|Q2NL51|GSK3A_MOUSE	Glycogen synthase kinase-3 alpha OS=Mus musculus OX=10090 GN=Gsk3a PE=1 SV=2	MOUSE	3
2532	2.54	2.76	14.12	1.40	sp|P42232|STA5B_MOUSE	Signal transducer and activator of transcription 5B OS=Mus musculus OX=10090 GN=Stat5b PE=1 SV=1	MOUSE	1
2533	2.54	2.72	9.18	2.89	tr|Q5SQ20|Q5SQ20_MOUSE	Pescadillo homolog OS=Mus musculus OX=10090 GN=Pes1 PE=1 SV=1	MOUSE	2
2534	2.54	2.67	6.69	0.62	sp|Q8C9B9|DIDO1_MOUSE	Death-inducer obliterator 1 OS=Mus musculus OX=10090 GN=Dido1 PE=1 SV=4	MOUSE	1
2535	2.54	2.58	9.92	5.37	tr|Z4YM84|Z4YM84_MOUSE	Acyl-CoA-binding domain-containing protein 5 OS=Mus musculus OX=10090 GN=Acbd5 PE=1 SV=2	MOUSE	2
2536	2.53	2.63	6.44	3.40	sp|Q8R5H6|WASF1_MOUSE	Wiskott-Aldrich syndrome protein family member 1 OS=Mus musculus OX=10090 GN=Wasf1 PE=1 SV=2	MOUSE	2
2537	2.53	2.58	14.60	6.06	tr|G5E8V8|G5E8V8_MOUSE	Phosphorylated adapter RNA export protein OS=Mus musculus OX=10090 GN=Phax PE=1 SV=1	MOUSE	2
2538	2.53	2.58	11.95	4.43	tr|Q3TY06|Q3TY06_MOUSE	Alpha-MPP (Fragment) OS=Mus musculus OX=10090 GN=Pmpca PE=2 SV=1	MOUSE	2
2539	2.53	2.57	6.95	5.52	tr|Q9DBE7|Q9DBE7_MOUSE	Double-stranded RNA-binding protein Staufen homolog 1 OS=Mus musculus OX=10090 GN=Stau1 PE=1 SV=1	MOUSE	2
2540	2.52	19.02	32.47	21.79	sp|Q61033|LAP2A_MOUSE	Lamina-associated polypeptide 2, isoforms alpha/zeta OS=Mus musculus OX=10090 GN=Tmpo PE=1 SV=4	MOUSE	13
2541	2.52	2.58	11.48	3.55	tr|Q6NXW0|Q6NXW0_MOUSE	Non-specific serine/threonine protein kinase OS=Mus musculus OX=10090 GN=Akt3 PE=2 SV=1	MOUSE	1
2542	2.52	2.55	19.57	19.57	sp|Q9CR60|GOT1B_MOUSE	Vesicle transport protein GOT1B OS=Mus musculus OX=10090 GN=Golt1b PE=1 SV=1	MOUSE	2
2543	2.51	2.63	6.52	1.50	tr|Q7TT13|Q7TT13_MOUSE	Mink1 protein OS=Mus musculus OX=10090 GN=Mink1 PE=2 SV=1	MOUSE	2

2544	2.51	2.56	14.58	8.66	sp|C0HKD9|MFA1B_MOUSE	Microfibrillar-associated protein 1B OS=Mus musculus OX=10090 GN=Mfap1b PE=1 SV=1	MOUSE	2
2545	2.51	2.55	29.81	23.08	sp|P61458|PHS_MOUSE	Pterin-4-alpha-carbinolamine dehydratase OS=Mus musculus OX=10090 GN=Pcbd1 PE=1 SV=2	MOUSE	2
2546	2.5	6.84	28.79	17.03	sp|Q8BHZ0|CYRIA_MOUSE	CYFIP-related Rac1 interactor A OS=Mus musculus OX=10090 GN=Cyria PE=1 SV=1	MOUSE	4
2547	2.49	2.61	13.60	11.03	tr|Q8BP79|Q8BP79_MOUSE	Holocytochrome c-type synthase OS=Mus musculus OX=10090 GN=Hccs PE=2 SV=1	MOUSE	2
2548	2.49	2.53	13.79	13.79	sp|Q8CHP5|PYM1_MOUSE	Partner of Y14 and mago OS=Mus musculus OX=10090 GN=Pym1 PE=1 SV=2	MOUSE	2
2549	2.49	2.52	20.00	10.27	tr|D3Z6H3|D3Z6H3_MOUSE	Dynactin subunit 6 OS=Mus musculus OX=10090 GN=Dctn6 PE=1 SV=1	MOUSE	2
2550	2.48	3.26	6.02	1.87	tr|Q5DTY3|Q5DTY3_MOUSE	Conserved oligomeric Golgi complex subunit 1 (Fragment) OS=Mus musculus OX=10090 GN=mKIAA1381 PE=3 SV=1	MOUSE	2
2551	2.48	2.83	9.49	2.28	tr|Q3UDN2|Q3UDN2_MOUSE	Vacuolar protein sorting-associated protein 53 homolog OS=Mus musculus OX=10090 GN=Vps53 PE=2 SV=1	MOUSE	2
2552	2.48	2.61	9.85	9.85	tr|I6L990|I6L990_MOUSE	Samd11 protein OS=Mus musculus OX=10090 GN=Samd11 PE=2 SV=1	MOUSE	2
2553	2.48	2.59	3.70	2.47	tr|F8VQK3|F8VQK3_MOUSE	Guanylate cyclase OS=Mus musculus OX=10090 GN=Gucy1a2 PE=1 SV=1	MOUSE	2
2554	2.47	2.58	27.57	11.21	tr|Q9CRA2|Q9CRA2_MOUSE	PDZ and LIM domain protein 5 OS=Mus musculus OX=10090 GN=Pdlim5 PE=1 SV=1	MOUSE	2
2555	2.47	2.51	8.19	3.93	tr|E9Q9M1|E9Q9M1_MOUSE	Cytosolic purine 5'-nucleotidase OS=Mus musculus OX=10090 GN=Nt5c2 PE=1 SV=1	MOUSE	2
2556	2.47	2.5	16.54	13.16	tr|Q9CVI1|Q9CVI1_MOUSE	Flavodoxin_2 domain-containing protein (Fragment) OS=Mus musculus OX=10090 GN=Nqo2 PE=2 SV=1	MOUSE	2
2557	2.46	2.6	16.59	4.23	sp|Q8BZR9|NCBP3_MOUSE	Nuclear cap-binding protein subunit 3 OS=Mus musculus OX=10090 GN=Ncbp3 PE=1 SV=1	MOUSE	2
2558	2.46	2.57	16.27	1.89	sp|Q99KC8|VMA5A_MOUSE	von Willebrand factor A domain-containing protein 5A OS=Mus musculus OX=10090 GN=Vwa5a PE=1 SV=2	MOUSE	1
2559	2.46	2.49	3.87	3.87	sp|Q8K3X4|I2BPL_MOUSE	Probable E3 ubiquitin-protein ligase IRF2BPL OS=Mus musculus OX=10090 GN=Irf2bpl PE=1 SV=1	MOUSE	2

2560	2.44	2.5	8.95	1.26	tr|Q3UZA5|Q3UZA5_MOUSE	Uncharacterized protein OS=Mus musculus OX=10090 GN=Kifap3 PE=2 SV=1	MOUSE	1
2561	2.44	2.47	13.60	13.60	tr|Q3TF81|Q3TF81_MOUSE	Uncharacterized protein (Fragment) OS=Mus musculus OX=10090 GN=Rpp30 PE=2 SV=1	MOUSE	2
2562	2.44	2.47	11.70	11.70	sp|Q9D7X8|GGCT_MOUSE	Gamma-glutamylcyclotransferase OS=Mus musculus OX=10090 GN=Ggct PE=1 SV=1	MOUSE	2
2563	2.43	2.47	32.10	19.75	tr|A0A2I3BQC3|A0A2I3BQC3_MOUSE	Small integral membrane protein 40 OS=Mus musculus OX=10090 GN=Smim40 PE=4 SV=1	MOUSE	2
2564	2.43	2.46	12.32	8.12	tr|B5TZD5|B5TZD5_MOUSE	D4 zinc and double PHD finger family 3 splice variant a OS=Mus musculus OX=10090 GN=Dpf3 PE=2 SV=1	MOUSE	2
2565	2.41	2.54	9.79	5.00	sp|Q99M87|DNJA3_MOUSE	DnaJ homolog subfamily A member 3, mitochondrial OS=Mus musculus OX=10090 GN=Dnaja3 PE=1 SV=1	MOUSE	2
2566	2.41	2.52	13.70	9.93	tr|Q14B19|Q14B19_MOUSE	Ventral anterior homeobox containing gene 2 OS=Mus musculus OX=10090 GN=Vax2 PE=2 SV=1	MOUSE	2
2567	2.41	2.44	19.10	19.10	sp|Q9D1I5|MCEE_MOUSE	Methylmalonyl-CoA epimerase, mitochondrial OS=Mus musculus OX=10090 GN=Mcee PE=1 SV=1	MOUSE	2
2568	2.4	2.5	16.08	8.48	tr|Q3TZ44|Q3TZ44_MOUSE	Aldose 1-epimerase OS=Mus musculus OX=10090 GN=Galm PE=2 SV=1	MOUSE	2
2569	2.4	2.44	13.96	11.04	tr|Q9D1J2|Q9D1J2_MOUSE	Coatomer subunit epsilon OS=Mus musculus OX=10090 GN=Cope PE=2 SV=1	MOUSE	2
2570	2.4	2.43	18.22	13.77	tr|Q3URS8|Q3URS8_MOUSE	Uncharacterized protein OS=Mus musculus OX=10090 GN=Mlf2 PE=2 SV=1	MOUSE	2
2571	2.4	2.43	13.06	3.82	sp|Q8VDN4|CCD92_MOUSE	Coiled-coil domain-containing protein 92 OS=Mus musculus OX=10090 GN=Ccdc92 PE=1 SV=1	MOUSE	1
2572	2.39	2.65	9.56	2.19	sp|P27641|XRCC5_MOUSE	X-ray repair cross-complementing protein 5 OS=Mus musculus OX=10090 GN=Xrcc5 PE=1 SV=4	MOUSE	1
2573	2.38	2.5	15.09	3.02	tr|Q4FK57|Q4FK57_MOUSE	Ero1l protein OS=Mus musculus OX=10090 GN=Ero1a PE=1 SV=1	MOUSE	1
2574	2.38	2.43	6.80	1.13	sp|Q8BY89|CTL2_MOUSE	Choline transporter-like protein 2 OS=Mus musculus OX=10090 GN=Slc44a2 PE=1 SV=2	MOUSE	1
2575	2.38	2.41	10.60	4.87	tr|Q9D0K3|Q9D0K3_MOUSE	AAA domain-containing protein OS=Mus musculus OX=10090 GN=Rfc2 PE=2 SV=1	MOUSE	1
2576	2.37	2.57	7.64	2.58	sp|Q9Z0H8|CLIP2_MOUSE	CAP-Gly domain-containing linker protein 2 OS=Mus musculus OX=10090 GN=Clip2 PE=1 SV=2	MOUSE	2

2577	2.37	2.43	6.93	2.31	tr|B2RUK5|B2RUK5_MOUSE	Methylcrotonoyl-Coenzyme A carboxylase 2 (Beta) OS=Mus musculus OX=10090 GN=Mccc2 PE=1 SV=1	MOUSE	1
2578	2.36	2.55	13.61	10.82	sp|Q8BZX4-2|SREK1_MOUSE	Isoform 2 of Splicing regulatory glutamine/lysine-rich protein 1 OS=Mus musculus OX=10090 GN=Srek1	MOUSE	4
2579	2.36	2.4	3.00	0.64	tr|Q3TSQ2|Q3TSQ2_MOUSE	Uncharacterized protein OS=Mus musculus OX=10090 GN=Pogz PE=2 SV=1	MOUSE	1
2580	2.36	2.4	7.55	2.60	sp|Q8VHL0|UT1_MOUSE	Urea transporter 1 OS=Mus musculus OX=10090 GN=Slc14a1 PE=1 SV=2	MOUSE	1
2581	2.36	2.39	12.16	3.65	sp|Q9D2E2-2|TOE1_MOUSE	Isoform 2 of Target of EGR1 protein 1 OS=Mus musculus OX=10090 GN=Toe1	MOUSE	1
2582	2.35	4.22	27.96	5.11	sp|Q99J95|CDK9_MOUSE	Cyclin-dependent kinase 9 OS=Mus musculus OX=10090 GN=Cdk9 PE=1 SV=1	MOUSE	2
2583	2.35	2.48	5.85	1.66	tr|M4TKR7|M4TKR7_MOUSE	Tyrosine-protein kinase receptor OS=Mus musculus OX=10090 GN=Insr PE=2 SV=1	MOUSE	2
2584	2.33	2.48	7.89	1.44	sp|Q6PAR5|GAPD1_MOUSE	GTPase-activating protein and VPS9 domain-containing protein 1 OS=Mus musculus OX=10090 GN=Gapvd1 PE=1 SV=2	MOUSE	2
2585	2.32	2.44	12.29	9.32	tr|G5E8W7|G5E8W7_MOUSE	Lysosomal thioesterase PPT2 (Fragment) OS=Mus musculus OX=10090 GN=Ppt2 PE=1 SV=1	MOUSE	2
2586	2.32	2.42	7.32	7.32	sp|Q9DBG5|PLIN3_MOUSE	Perilipin-3 OS=Mus musculus OX=10090 GN=Plin3 PE=1 SV=1	MOUSE	2
2587	2.32	2.34	5.25	5.25	sp|Q80TL4|PHF24_MOUSE	PHD finger protein 24 OS=Mus musculus OX=10090 GN=Phf24 PE=1 SV=2	MOUSE	2
2588	2.31	2.47	8.17	5.55	sp|Q80U56|AVL9_MOUSE	Late secretory pathway protein AVL9 homolog OS=Mus musculus OX=10090 GN=Avl9 PE=1 SV=2	MOUSE	3
2589	2.31	2.34	7.69	4.94	tr|Q9EQ66|Q9EQ66_MOUSE	Aspartly beta-hydroxylase OS=Mus musculus OX=10090 GN=ASPH PE=3 SV=1	MOUSE	1
2590	2.31	2.34	15.71	8.57	sp|Q8BIS8|CC126_MOUSE	Coiled-coil domain-containing protein 126 OS=Mus musculus OX=10090 GN=Ccdc126 PE=2 SV=1	MOUSE	1
2591	2.3	9.51	7.68	4.19	tr|Q0VGY9|Q0VGY9_MOUSE	Ank1 protein OS=Mus musculus OX=10090 GN=Ank1 PE=1 SV=1	MOUSE	7
2592	2.3	2.62	14.86	3.51	sp|Q9Z2Y3-2|HOME1_MOUSE	Isoform 2 of Homer protein homolog 1 OS=Mus musculus OX=10090 GN=Homer1	MOUSE	1

2593	2.3	2.34	26.55	11.06	sp|P40240|CD9_MOUSE	CD9 antigen OS=Mus musculus OX=10090 GN=Cd9 PE=1 SV=2	MOUSE	2
2594	2.29	318.27	71.62	66.57	tr|A3KGU9|A3KGU9_MOUSE	Spectrin alpha chain, non-erythrocytic 1 OS=Mus musculus OX=10090 GN=Sptan1 PE=1 SV=2	MOUSE	170
2595	2.29	2.44	11.70	9.04	tr|B0V2Q7|B0V2Q7_MOUSE	Lclat1 protein OS=Mus musculus OX=10090 GN=Lclat1 PE=1 SV=1	MOUSE	2
2596	2.29	2.33	10.70	2.28	tr|A2AR56|A2AR56_MOUSE	FERM domain-containing protein 5 OS=Mus musculus OX=10090 GN=Frmd5 PE=1 SV=1	MOUSE	1
2597	2.28	2.46	27.43	10.97	tr|Q3U6J4|Q3U6J4_MOUSE	Uncharacterized protein (Fragment) OS=Mus musculus OX=10090 GN=Mettl3 PE=2 SV=1	MOUSE	4
2598	2.28	2.39	6.92	2.89	sp|Q80T69|RSBN1_MOUSE	Lysine-specific demethylase 9 OS=Mus musculus OX=10090 GN=Rsbn1 PE=1 SV=3	MOUSE	2
2599	2.28	2.31	13.12	4.37	tr|A2AUC6|A2AUC6_MOUSE	Bardet-Biedl syndrome 5 protein homolog OS=Mus musculus OX=10090 GN=Bbs5 PE=1 SV=1	MOUSE	1
2600	2.28	2.31	13.39	4.91	sp|Q99M71|EPDR1_MOUSE	Mammalian ependymin-related protein 1 OS=Mus musculus OX=10090 GN=Epdr1 PE=1 SV=1	MOUSE	1
2601	2.27	4.22	10.83	7.50	tr|Q6AXH6|Q6AXH6_MOUSE	Abi2 protein OS=Mus musculus OX=10090 GN=Abi2 PE=1 SV=1	MOUSE	3
2602	2.27	2.31	22.76	4.84	tr|Q8BYP9|Q8BYP9_MOUSE	GB1/RHD3-type G domain-containing protein OS=Mus musculus OX=10090 GN=Atl2 PE=2 SV=1	MOUSE	1
2603	2.26	10.4	31.88	22.22	tr|Q0PD49|Q0PD49_MOUSE	RAB8B, member RAS oncogene family OS=Mus musculus OX=10090 GN=Rab8b PE=1 SV=1	MOUSE	5
2604	2.26	2.37	16.32	2.97	tr|D3Z132|D3Z132_MOUSE	THO complex subunit 6 homolog OS=Mus musculus OX=10090 GN=Thoc6 PE=1 SV=1	MOUSE	1
2605	2.26	2.36	9.62	9.62	tr|Q7TMX6|Q7TMX6_MOUSE	Exosome component 7 OS=Mus musculus OX=10090 GN=Exosc7 PE=2 SV=1	MOUSE	2
2606	2.26	2.29	6.19	2.06	sp|Q9JI90|RNF14_MOUSE	E3 ubiquitin-protein ligase RNF14 OS=Mus musculus OX=10090 GN=Rnf14 PE=1 SV=2	MOUSE	1
2607	2.25	2.28	13.05	7.14	sp|Q3URS9|MITOK_MOUSE	Mitochondrial potassium channel OS=Mus musculus OX=10090 GN=Ccdc51 PE=1 SV=1	MOUSE	2
2608	2.25	2.27	15.09	7.37	tr|Q91VJ9|Q91VJ9_MOUSE	1-acyl-sn-glycerol-3-phosphate acyltransferase OS=Mus musculus OX=10090 GN=Agpat1 PE=2 SV=1	MOUSE	1
2609	2.25	2.27	13.04	4.01	sp|Q9JKY0|CNOT9_MOUSE	CCR4-NOT transcription complex subunit 9 OS=Mus musculus OX=10090 GN=Cnot9 PE=1 SV=1	MOUSE	1

2610	2.24	2.27	10.96	2.30	tr|Q3UZ63|Q3UZ63_MOUSE	zf-CCCH_3 domain-containing protein (Fragment) OS=Mus musculus OX=10090 GN=Zc3h11a PE=2 SV=1	MOUSE	1
2611	2.24	2.27	11.20	3.04	tr|Q91XI2|Q91XI2_MOUSE	Catalase OS=Mus musculus OX=10090 GN=Cat PE=2 SV=1	MOUSE	1
2612	2.24	2.27	28.65	11.80	sp|P49817|CAV1_MOUSE	Caveolin-1 OS=Mus musculus OX=10090 GN=Cav1 PE=1 SV=1	MOUSE	1
2613	2.24	2.27	12.87	9.41	tr|A0A0A6YY12|A0A0A6YY12_MOUSE	Delta(14)-sterol reductase LBR (Fragment) OS=Mus musculus OX=10090 GN=Lbr PE=1 SV=1	MOUSE	1
2614	2.22	2.27	10.86	3.09	tr|F6V2U0|F6V2U0_MOUSE	Phosphatidylinositol-3,4-bisphosphate 4-phosphatase OS=Mus musculus OX=10090 GN=Inpp4a PE=1 SV=1	MOUSE	2
2615	2.22	2.24	7.21	1.84	tr|Q3UDU6|Q3UDU6_MOUSE	Rab-GAP TBC domain-containing protein OS=Mus musculus OX=10090 GN=Tbc1d17 PE=2 SV=1	MOUSE	1
2616	2.21	2.3	8.10	4.98	sp|O54851|CXD2_MOUSE	Gap junction delta-2 protein OS=Mus musculus OX=10090 GN=Gjd2 PE=1 SV=2	MOUSE	2
2617	2.21	2.24	17.19	2.81	tr|Q8BHC2|Q8BHC2_MOUSE	IST1 homolog OS=Mus musculus OX=10090 GN=Ist1 PE=2 SV=1	MOUSE	1
2618	2.21	2.23	5.34	1.01	tr|B2RRE0|B2RRE0_MOUSE	A kinase (PRKA) anchor protein (Gravin) 12 OS=Mus musculus OX=10090 GN=Akap12 PE=2 SV=1	MOUSE	1
2619	2.21	2.23	7.96	3.98	tr|Q14AZ9|Q14AZ9_MOUSE	Zinc binding alcohol dehydrogenase, domain containing 2 OS=Mus musculus OX=10090 GN=Zadh2 PE=1 SV=1	MOUSE	1
2620	2.19	8.4	65.56	38.89	tr|A0A338P6P7|A0A338P6P7_MOUSE	Protein kinase C and casein kinase substrate in neurons protein 2 (Fragment) OS=Mus musculus OX=10090 GN=Pacsin2 PE=1 SV=1	MOUSE	5
2621	2.19	2.21	19.91	3.36	sp|Q8CI32|BAG5_MOUSE	BAG family molecular chaperone regulator 5 OS=Mus musculus OX=10090 GN=Bag5 PE=1 SV=1	MOUSE	1
2622	2.19	2.21	11.23	1.60	tr|Q3TV20|Q3TV20_MOUSE	Asparagine synthetase [glutamine-hydrolyzing] OS=Mus musculus OX=10090 GN=Asns PE=2 SV=1	MOUSE	1
2623	2.18	2.31	25.12	11.11	sp|Q8BR63|F177A_MOUSE	Protein FAM177A1 OS=Mus musculus OX=10090 GN=Fam177a1 PE=1 SV=1	MOUSE	2
2624	2.18	2.2	15.92	7.35	tr|Q542B0|Q542B0_MOUSE	Uncharacterized protein OS=Mus musculus OX=10090 GN=Exosc4 PE=1 SV=1	MOUSE	1
2625	2.17	13.18	29.48	15.54	tr|Q3T9A1|Q3T9A1_MOUSE	Dynamin-type G domain-containing protein (Fragment) OS=Mus musculus OX=10090 GN=Ehd1 PE=2 SV=1	MOUSE	8

2626	2.17	2.51	26.01	8.09	sp|Q9D1H6|NDUF4_MOUSE	NADH dehydrogenase [ubiquinone] 1 alpha subcomplex assembly factor 4 OS=Mus musculus OX=10090 GN=Ndufaf4 PE=1 SV=1	MOUSE	1
2627	2.17	2.29	21.24	5.70	sp|Q8BTU1|CFA20_MOUSE	Cilia- and flagella-associated protein 20 OS=Mus musculus OX=10090 GN=Cfap20 PE=1 SV=1	MOUSE	1
2628	2.17	2.28	8.52	2.26	sp|Q8K0D5|EFGM_MOUSE	Elongation factor G, mitochondrial OS=Mus musculus OX=10090 GN=Gfm1 PE=1 SV=1	MOUSE	2
2629	2.17	2.19	18.22	10.22	tr|Q3U5M2|Q3U5M2_MOUSE	Pre-mRNA-splicing factor SPF27 OS=Mus musculus OX=10090 GN=Bcas2 PE=2 SV=1	MOUSE	1
2630	2.16	10.41	18.21	13.06	tr|Q66K07|Q66K07_MOUSE	Ubqln1 protein OS=Mus musculus OX=10090 GN=Ubqln1 PE=2 SV=1	MOUSE	5
2631	2.16	2.27	7.46	0.78	sp|P01027|CO3_MOUSE	Complement C3 OS=Mus musculus OX=10090 GN=C3 PE=1 SV=3	MOUSE	1
2632	2.16	2.18	15.14	9.96	tr|D3YY42|D3YY42_MOUSE	Protein YIF1B OS=Mus musculus OX=10090 GN=Yif1b PE=1 SV=1	MOUSE	1
2633	2.16	2.17	8.23	2.13	tr|A2AMH5|A2AMH5_MOUSE	Choline transporter-like protein 1 OS=Mus musculus OX=10090 GN=Slc44a1 PE=1 SV=1	MOUSE	1
2634	2.15	2.3	25.42	8.33	tr|A0A0R4J018|A0A0R4J018_MOUSE	Thiopurine S-methyltransferase OS=Mus musculus OX=10090 GN=Tpmt PE=1 SV=1	MOUSE	2
2635	2.15	2.16	21.20	5.07	tr|Q3UTB8|Q3UTB8_MOUSE	GrpE protein homolog OS=Mus musculus OX=10090 GN=Grpel1 PE=2 SV=1	MOUSE	1
2636	2.15	2.16	7.72	2.03	sp|Q32M02|FSCN2_MOUSE	Fascin-2 OS=Mus musculus OX=10090 GN=Fscn2 PE=2 SV=1	MOUSE	1
2637	2.14	2.26	23.49	15.10	sp|Q9EQ06|DHB11_MOUSE	Estradiol 17-beta-dehydrogenase 11 OS=Mus musculus OX=10090 GN=Hsd17b11 PE=1 SV=1	MOUSE	2
2638	2.14	2.24	13.75	3.09	sp|P28651|CAH8_MOUSE	Carbonic anhydrase-related protein OS=Mus musculus OX=10090 GN=Ca8 PE=1 SV=5	MOUSE	1
2639	2.14	2.16	6.07	1.93	sp|Q5U3K5|RABL6_MOUSE	Rab-like protein 6 OS=Mus musculus OX=10090 GN=Rabl6 PE=1 SV=2	MOUSE	1
2640	2.14	2.16	8.50	2.36	sp|Q8R0H9|GGA1_MOUSE	ADP-ribosylation factor-binding protein GGA1 OS=Mus musculus OX=10090 GN=Gga1 PE=1 SV=1	MOUSE	1
2641	2.14	2.16	4.88	2.55	sp|Q8BM72|HSP13_MOUSE	Heat shock 70 kDa protein 13 OS=Mus musculus OX=10090 GN=Hspa13 PE=1 SV=1	MOUSE	1

2642	2.13	2.36	7.37	1.65	tr|Q3TR28|Q3TR28_MOUSE	Uncharacterized protein OS=Mus musculus OX=10090 GN=Trim9 PE=2 SV=1	MOUSE	1
2643	2.13	2.15	19.50	7.45	sp|Q9D7A8|ARMC1_MOUSE	Armadillo repeat-containing protein 1 OS=Mus musculus OX=10090 GN=Armc1 PE=1 SV=1	MOUSE	1
2644	2.13	2.14	14.06	2.54	tr|E9Q3M0|E9Q3M0_MOUSE	Mothers against decapentaplegic homolog OS=Mus musculus OX=10090 GN=Smad2 PE=1 SV=1	MOUSE	1
2645	2.13	2.14	11.74	8.50	tr|Q9D7Y7|Q9D7Y7_MOUSE	Peptidase S1 domain-containing protein OS=Mus musculus OX=10090 GN=2210010C04Rik PE=2 SV=1	MOUSE	4
2646	2.12	6.19	10.31	5.42	tr|Q08EK5|Q08EK5_MOUSE	Keratin 77 OS=Mus musculus OX=10090 GN=Krt77 PE=1 SV=1	MOUSE	6
2647	2.12	5.73	13.25	7.92	sp|Q9DBS5|KLC4_MOUSE	Kinesin light chain 4 OS=Mus musculus OX=10090 GN=Klc4 PE=1 SV=1	MOUSE	3
2648	2.12	3.21	14.44	8.30	sp|P70677|CASP3_MOUSE	Caspase-3 OS=Mus musculus OX=10090 GN=Casp3 PE=1 SV=1	MOUSE	2
2649	2.12	3.01	11.16	11.16	tr|A2A6M1|A2A6M1_MOUSE	Vacuolar-sorting protein SNF8 OS=Mus musculus OX=10090 GN=Snf8 PE=1 SV=1	MOUSE	2
2650	2.12	2.21	6.80	5.44	sp|Q99JP7|GGT7_MOUSE	Glutathione hydrolase 7 OS=Mus musculus OX=10090 GN=Ggt7 PE=1 SV=2	MOUSE	2
2651	2.12	2.15	20.68	5.06	tr|Q3U7F6|Q3U7F6_MOUSE	Uncharacterized protein OS=Mus musculus OX=10090 GN=Zfp422 PE=2 SV=1	MOUSE	1
2652	2.12	2.13	45.45	16.53	sp|P60840|ENSA_MOUSE	Alpha-endosulfine OS=Mus musculus OX=10090 GN=Ensa PE=1 SV=1	MOUSE	2
2653	2.12	2.13	7.14	5.13	tr|B1AU76|B1AU76_MOUSE	Nuclear autoantigenic sperm protein OS=Mus musculus OX=10090 GN=Nasp PE=1 SV=1	MOUSE	1
2654	2.11	2.74	25.34	10.27	tr|H3BL23|H3BL23_MOUSE	Ubiquitin-conjugating enzyme E2 E2 (Fragment) OS=Mus musculus OX=10090 GN=Ube2e2 PE=1 SV=1	MOUSE	2
2655	2.11	2.58	32.76	24.14	tr|Q8BM48|Q8BM48_MOUSE	Microsomal signal peptidase 21 kDa subunit OS=Mus musculus OX=10090 GN=Sec11c PE=2 SV=1	MOUSE	2
2656	2.11	2.24	6.50	0.97	sp|Q6KCD5-2|NIPBL_MOUSE	Isoform 2 of Nipped-B-like protein OS=Mus musculus OX=10090 GN=Nipbl	MOUSE	2
2657	2.11	2.23	4.94	1.11	tr|F8WIA1|F8WIA1_MOUSE	CAP-Gly domain-containing linker protein 1 OS=Mus musculus OX=10090 GN=Clip1 PE=1 SV=1	MOUSE	1
2658	2.11	2.23	11.71	3.10	sp|Q8K268|ABCF3_MOUSE	ATP-binding cassette sub-family F member 3 OS=Mus musculus OX=10090 GN=Abcf3 PE=1 SV=1	MOUSE	1

2659	2.11	2.17	33.95	14.20	tr|D3YWI4|D3YWI4_MOUSE	Protein Mpv17 OS=Mus musculus OX=10090 GN=Mpv17 PE=1 SV=1	MOUSE	1
2660	2.11	2.12	8.23	2.50	tr|Q3UGN6|Q3UGN6_MOUSE	3-phosphoinositide-dependent protein kinase 1 OS=Mus musculus OX=10090 GN=Pdpk1 PE=2 SV=1	MOUSE	1
2661	2.1	2.52	5.94	0.91	tr|A2A485|A2A485_MOUSE	Zinc finger, MYND-type-containing 8 OS=Mus musculus OX=10090 GN=Zmynd8 PE=1 SV=1	MOUSE	1
2662	2.1	2.36	17.74	3.98	sp|P40336|VP26A_MOUSE	Vacuolar protein sorting-associated protein 26A OS=Mus musculus OX=10090 GN=Vps26a PE=1 SV=1	MOUSE	1
2663	2.1	2.26	7.78	2.38	tr|Q6PGJ3|Q6PGJ3_MOUSE	L1 cell adhesion molecule OS=Mus musculus OX=10090 GN=L1cam PE=1 SV=1	MOUSE	2
2664	2.1	2.12	6.10	0.88	sp|P10493|NID1_MOUSE	Nidogen-1 OS=Mus musculus OX=10090 GN=Nid1 PE=1 SV=2	MOUSE	1
2665	2.1	2.11	5.08	3.29	tr|Q8BMS7|Q8BMS7_MOUSE	Calcium-transporting ATPase OS=Mus musculus OX=10090 GN=Atp2c1 PE=1 SV=1	MOUSE	2
2666	2.09	12.82	16.23	8.68	sp|Q99LG2|TNPO2_MOUSE	Transportin-2 OS=Mus musculus OX=10090 GN=Tnpo2 PE=1 SV=1	MOUSE	7
2667	2.09	3.17	26.15	26.15	sp|Q9D0R8|LSM12_MOUSE	Protein LSM12 homolog OS=Mus musculus OX=10090 GN=Lsm12 PE=1 SV=1	MOUSE	3
2668	2.09	2.25	9.14	4.66	tr|Z4YJT3|Z4YJT3_MOUSE	La-related protein 1 OS=Mus musculus OX=10090 GN=Larp1 PE=1 SV=1	MOUSE	3
2669	2.09	2.24	22.03	7.34	sp|Q62086|PON2_MOUSE	Serum paraoxonase/arylesterase 2 OS=Mus musculus OX=10090 GN=Pon2 PE=1 SV=2	MOUSE	2
2670	2.09	2.11	19.83	3.03	tr|D3Z6W2|D3Z6W2_MOUSE	Tyrosine-protein phosphatase non-receptor type OS=Mus musculus OX=10090 GN=Ptpn2 PE=1 SV=1	MOUSE	1
2671	2.09	2.1	4.93	1.13	tr|B2RWW1|B2RWW1_MOUSE	Microtubule-associated protein 1S OS=Mus musculus OX=10090 GN=Map1s PE=2 SV=1	MOUSE	1
2672	2.08	13.14	56.45	50.54	sp|Q8VEH3|ARL8A_MOUSE	ADP-ribosylation factor-like protein 8A OS=Mus musculus OX=10090 GN=Arl8a PE=1 SV=1	MOUSE	8
2673	2.08	4.35	9.29	2.83	tr|Q4LG64|Q4LG64_MOUSE	Glutamate receptor OS=Mus musculus OX=10090 GN=Gria2 PE=2 SV=1	MOUSE	3
2674	2.08	2.96	9.34	2.09	tr|F8VQE9|F8VQE9_MOUSE	Arf-GAP with GTPase, ANK repeat and PH domain-containing protein 3 OS=Mus musculus OX=10090 GN=Agap3 PE=1 SV=1	MOUSE	2

2675	2.08	2.09	6.59	1.60	tr|E9PW03|E9PW03_MOUSE	Glycosylphosphatidylinositol anchor attachment 1 protein OS=Mus musculus OX=10090 GN=Gpaa1 PE=1 SV=1	MOUSE	1
2676	2.07	9.45	19.01	14.32	tr|Q9CZS7|Q9CZS7_MOUSE	PIPK domain-containing protein OS=Mus musculus OX=10090 GN=Pip4k2a PE=2 SV=1	MOUSE	6
2677	2.07	4.13	24.75	16.16	sp|Q91Z61|DIRA1_MOUSE	GTP-binding protein Di-Ras1 OS=Mus musculus OX=10090 GN=Diras1 PE=1 SV=1	MOUSE	3
2678	2.07	2.2	35.06	13.85	tr|Q9Z2P7|Q9Z2P7_MOUSE	GES30 OS=Mus musculus OX=10090 GN=Vti1b PE=2 SV=1	MOUSE	2
2679	2.07	2.19	6.01	1.91	tr|A2AVJ7|A2AVJ7_MOUSE	Ribosome-binding protein 1 OS=Mus musculus OX=10090 GN=Rrbp1 PE=1 SV=1	MOUSE	2
2680	2.07	2.18	20.45	4.87	tr|Z4YKT6|Z4YKT6_MOUSE	Dehydrogenase/reductase SDR family member 7B OS=Mus musculus OX=10090 GN=Dhrs7b PE=1 SV=1	MOUSE	1
2681	2.07	2.12	3.77	1.15	tr|Q4VA02|Q4VA02_MOUSE	Rp1 protein (Fragment) OS=Mus musculus OX=10090 GN=Rp1 PE=2 SV=1	MOUSE	1
2682	2.07	2.11	16.81	3.78	tr|A2VCP7|A2VCP7_MOUSE	Proteasome inhibitor PI31 subunit (Fragment) OS=Mus musculus OX=10090 GN=Psmf1 PE=2 SV=1	MOUSE	1
2683	2.07	2.09	13.11	5.57	tr|Q3UZR8|Q3UZR8_MOUSE	Translation initiation factor eIF-2B subunit alpha OS=Mus musculus OX=10090 GN=Eif2b1 PE=1 SV=1	MOUSE	1
2684	2.07	2.08	13.00	2.50	tr|A0A1L1STF0|A0A1L1STF0_MOUSE	Ubiquitin carboxyl-terminal hydrolase OS=Mus musculus OX=10090 GN=Mindy2 PE=1 SV=1	MOUSE	1
2685	2.07	2.08	8.06	2.02	tr|F6T1Y2|F6T1Y2_MOUSE	Rho GTPase-activating protein 44 (Fragment) OS=Mus musculus OX=10090 GN=Arhgap44 PE=1 SV=1	MOUSE	1
2686	2.07	2.08	4.69	2.96	tr|A0A1L1SS47|A0A1L1SS47_MOUSE	CCR4-NOT transcription complex subunit 10 OS=Mus musculus OX=10090 GN=Cnot10 PE=1 SV=1	MOUSE	1
2687	2.07	2.08	23.96	16.67	sp|Q6ZWM4|LSM8_MOUSE	U6 snRNA-associated Sm-like protein LSm8 OS=Mus musculus OX=10090 GN=Lsm8 PE=1 SV=3	MOUSE	1
2688	2.07	2.07	33.33	9.88	tr|A0A0R4J0K1|A0A0R4J0K1_MOUSE	Selenoprotein F OS=Mus musculus OX=10090 GN=Selenof PE=1 SV=1	MOUSE	1
2689	2.06	2.45	51.69	51.69	sp|P99028|QCR6_MOUSE	Cytochrome b-c1 complex subunit 6, mitochondrial OS=Mus musculus OX=10090 GN=Uqcrh PE=1 SV=2	MOUSE	3
2690	2.06	2.12	7.96	3.10	sp|O55222|ILK_MOUSE	Integrin-linked protein kinase OS=Mus musculus OX=10090 GN=Ilk PE=1 SV=2	MOUSE	1
2691	2.06	2.08	13.94	4.81	tr|Q922W2|Q922W2_MOUSE	Qrich1 protein OS=Mus musculus OX=10090 GN=Qrich1 PE=2 SV=1	MOUSE	1

2692	2.06	2.07	42.57	18.24	tr|Q8R2K3|Q8R2K3_MOUSE	Single-stranded DNA-binding protein, mitochondrial OS=Mus musculus OX=10090 GN=Ssbp1 PE=1 SV=1	MOUSE	2
2693	2.06	2.07	11.53	2.88	sp|Q9EPK2|XRP2_MOUSE	Protein XRP2 OS=Mus musculus OX=10090 GN=Rp2 PE=1 SV=3	MOUSE	1
2694	2.05	52.71	50.31	40.90	tr|A0A3B2WBE1|A0A3B2WBE1_MOUSE	Band 4.1-like protein 3 (Fragment) OS=Mus musculus OX=10090 GN=Epb41l3 PE=1 SV=1	MOUSE	28
2695	2.05	8.17	36.70	21.10	tr|Q6PJ91|Q6PJ91_MOUSE	Glutathione transferase OS=Mus musculus OX=10090 GN=Gstm7 PE=2 SV=1	MOUSE	4
2696	2.05	4.13	8.43	3.03	sp|Q8JZW4|CPNE5_MOUSE	Copine-5 OS=Mus musculus OX=10090 GN=Cpne5 PE=1 SV=1	MOUSE	3
2697	2.05	2.23	5.32	0.94	tr|Q3UHQ5|Q3UHQ5_MOUSE	TOG domain-containing protein OS=Mus musculus OX=10090 GN=Gcn1 PE=2 SV=1	MOUSE	2
2698	2.05	2.06	7.49	2.89	tr|D3YWR0|D3YWR0_MOUSE	Protein Aster-B OS=Mus musculus OX=10090 GN=Gramd1b PE=1 SV=2	MOUSE	1
2699	2.04	39.56	57.41	35.85	tr|V9GZG5|V9GZG5_MOUSE	Palmitoyltransferase GLUL OS=Mus musculus OX=10090 PE=3 SV=1	MOUSE	38
2700	2.04	9.66	16.54	9.66	tr|Q80XK0|Q80XK0_MOUSE	Serine/threonine-protein phosphatase (Fragment) OS=Mus musculus OX=10090 GN=Ppp3cc PE=2 SV=1	MOUSE	5
2701	2.04	2.17	14.86	2.39	sp|Q0VBL3|RBM15_MOUSE	RNA-binding protein 15 OS=Mus musculus OX=10090 GN=Rbm15 PE=1 SV=1	MOUSE	1
2702	2.04	2.15	8.91	4.93	tr|Q3U0M8|Q3U0M8_MOUSE	ElaC homolog protein 2 OS=Mus musculus OX=10090 GN=Elac2 PE=2 SV=1	MOUSE	2
2703	2.04	2.15	3.97	3.97	tr|B2RWR2|B2RWR2_MOUSE	Lpcat2 protein OS=Mus musculus OX=10090 GN=Lpcat2 PE=2 SV=1	MOUSE	2
2704	2.04	2.05	12.27	4.53	tr|Q7TT04|Q7TT04_MOUSE	Nup43 protein (Fragment) OS=Mus musculus OX=10090 GN=Nup43 PE=2 SV=2	MOUSE	1
2705	2.04	2.04	10.80	2.81	tr|A0A2R8VHX0|A0A2R8VHX0_MOUSE	Cysteine sulfinic acid decarboxylase OS=Mus musculus OX=10090 GN=Csad PE=1 SV=1	MOUSE	1
2706	2.04	2.04	7.01	1.35	tr|Q5QNU0|Q5QNU0_MOUSE	Uncharacterized protein OS=Mus musculus OX=10090 GN=Nup88 PE=2 SV=1	MOUSE	1
2707	2.03	2.11	15.85	4.61	tr|Q543U1|Q543U1_MOUSE	Hist_deacetyl domain-containing protein OS=Mus musculus OX=10090 GN=Hdac11 PE=1 SV=1	MOUSE	1
2708	2.03	2.04	14.26	2.04	sp|Q9Z0E6|GBP2_MOUSE	Guanylate-binding protein 2 OS=Mus musculus OX=10090 GN=Gbp2 PE=1 SV=1	MOUSE	1

2709	2.03	2.04	15.79	5.54	tr|Q99L41|Q99L41_MOUSE	Hydroxymethylbilane synthase OS=Mus musculus OX=10090 GN=Hmbs PE=2 SV=1	MOUSE	1
2710	2.03	2.04	7.88	3.64	tr|Q8R0M2|Q8R0M2_MOUSE	UTP--glucose-1-phosphate uridylyltransferase (Fragment) OS=Mus musculus OX=10090 GN=Ugp2 PE=2 SV=1	MOUSE	2
2711	2.03	2.03	3.97	1.02	sp|Q8VI75|IPO4_MOUSE	Importin-4 OS=Mus musculus OX=10090 GN=Ipo4 PE=1 SV=1	MOUSE	1
2712	2.03	2.03	3.57	1.13	sp|P98200|AT8A2_MOUSE	Phospholipid-transporting ATPase IB OS=Mus musculus OX=10090 GN=Atp8a2 PE=1 SV=1	MOUSE	1
2713	2.03	2.03	22.76	6.90	tr|D3YYL1|D3YYL1_MOUSE	B-cell CLL/lymphoma 7 protein family member B OS=Mus musculus OX=10090 GN=Bcl7b PE=1 SV=1	MOUSE	1
2714	2.03	2.03	6.03	3.29	tr|Q3TDP3|Q3TDP3_MOUSE	PlsC domain-containing protein OS=Mus musculus OX=10090 GN=Agpat5 PE=2 SV=1	MOUSE	1
2715	2.03	2.03	9.38	6.25	sp|Q9WV35|ABEC2_MOUSE	C->U-editing enzyme APOBEC-2 OS=Mus musculus OX=10090 GN=Apobec2 PE=1 SV=1	MOUSE	1
2716	2.02	4.69	12.16	10.86	tr|Q3TYJ3|Q3TYJ3_MOUSE	Cytoplasmic dynein 1 intermediate chain 1 OS=Mus musculus OX=10090 GN=Dync1i1 PE=1 SV=1	MOUSE	4
2717	2.02	2.11	1.79	0.68	tr|E9Q3T3|E9Q3T3_MOUSE	Kinesin-like protein KIF14 OS=Mus musculus OX=10090 GN=Kif14 PE=1 SV=1	MOUSE	1
2718	2.02	2.04	3.73	0.77	tr|E9PWR4|E9PWR4_MOUSE	Disco-interacting protein 2 homolog C OS=Mus musculus OX=10090 GN=Dip2c PE=1 SV=1	MOUSE	1
2719	2.02	2.04	4.66	2.10	tr|Q6A0A4|Q6A0A4_MOUSE	MKIAA0205 protein (Fragment) OS=Mus musculus OX=10090 GN=Lpgat1 PE=2 SV=1	MOUSE	1
2720	2.02	2.03	14.75	3.28	sp|Q9D1E6|TBCB_MOUSE	Tubulin-folding cofactor B OS=Mus musculus OX=10090 GN=Tbcb PE=1 SV=2	MOUSE	1
2721	2.02	2.03	6.28	2.76	tr|D3YWC9|D3YWC9_MOUSE	Protein MTSS 2 OS=Mus musculus OX=10090 GN=Mtss2 PE=1 SV=1	MOUSE	1
2722	2.02	2.03	8.63	3.58	tr|Q543Z1|Q543Z1_MOUSE	Carboxylic ester hydrolase OS=Mus musculus OX=10090 GN=Ache PE=2 SV=1	MOUSE	1
2723	2.02	2.03	5.18	3.75	tr|J3QNQ8|J3QNQ8_MOUSE	Leucine-rich repeat, immunoglobulin-like domain and transmembrane domain-containing protein 3 OS=Mus musculus OX=10090 GN=Lrit3 PE=4 SV=1	MOUSE	1
2724	2.02	2.02	13.79	8.97	tr|Q4KL41|Q4KL41_MOUSE	Translocase of outer mitochondrial membrane 20 homolog (Yeast) OS=Mus musculus OX=10090 GN=Tomm20 PE=1 SV=1	MOUSE	1

2725	2.02	2.02	2.34	1.45	tr|A0A2I3BQJ1|A0A2I3BQJ1_MOUSE	Serine/threonine-protein phosphatase 6 regulatory ankyrin repeat subunit A OS=Mus musculus OX=10090 GN=Ankrd28 PE=1 SV=1	MOUSE	1
2726	2.02	2.02	8.73	4.36	sp|Q9CRA5|GOLP3_MOUSE	Golgi phosphoprotein 3 OS=Mus musculus OX=10090 GN=Golph3 PE=1 SV=1	MOUSE	1
2727	2.02	2.02	14.34	9.84	sp|Q8C1S0|MED19_MOUSE	Mediator of RNA polymerase II transcription subunit 19 OS=Mus musculus OX=10090 GN=Med19 PE=1 SV=1	MOUSE	1
2728	2.01	33.85	77.67	62.62	sp|P60879-2|SNP25_MOUSE	Isoform 2 of Synaptosomal-associated protein 25 OS=Mus musculus OX=10090 GN=Snap25	MOUSE	22
2729	2.01	11.95	48.40	30.59	tr|Q0PD62|Q0PD62_MOUSE	Ras-related protein Rab-3 OS=Mus musculus OX=10090 GN=Rab3b PE=1 SV=1	MOUSE	10
2730	2.01	10.71	12.17	7.59	tr|E9PXY1|E9PXY1_MOUSE	Cullin-4B OS=Mus musculus OX=10090 GN=Cul4b PE=1 SV=2	MOUSE	6
2731	2.01	2.59	28.13	13.12	tr|M0QWC2|M0QWC2_MOUSE	Microtubule-associated proteins 1A/1B light chain 3B OS=Mus musculus OX=10090 GN=Map1lc3b PE=1 SV=1	MOUSE	2
2732	2.01	2.55	16.13	10.42	tr|Q9DB01|Q9DB01_MOUSE	Uncharacterized protein OS=Mus musculus OX=10090 GN=Pcbp4 PE=2 SV=1	MOUSE	4
2733	2.01	2.22	10.39	3.79	tr|Q3URI3|Q3URI3_MOUSE	Phosphatidylinositol-3,4-bisphosphate 4-phosphatase (Fragment) OS=Mus musculus OX=10090 GN=Inpp4b PE=1 SV=1	MOUSE	2
2734	2.01	2.08	10.99	2.82	tr|Q3V1X4|Q3V1X4_MOUSE	Uncharacterized protein OS=Mus musculus OX=10090 GN=Surf6 PE=2 SV=1	MOUSE	1
2735	2.01	2.02	8.64	3.04	tr|E0CYH4|E0CYH4_MOUSE	WD repeat-containing protein 26 (Fragment) OS=Mus musculus OX=10090 GN=Wdr26 PE=1 SV=1	MOUSE	1
2736	2.01	2.02	36.00	10.40	sp|Q9DCG9|TR112_MOUSE	Multifunctional methyltransferase subunit TRM112-like protein OS=Mus musculus OX=10090 GN=Trmt112 PE=1 SV=1	MOUSE	2
2737	2.01	2.02	5.86	1.81	sp|Q9CXJ4|MITOS_MOUSE	Mitochondrial potassium channel ATP-binding subunit OS=Mus musculus OX=10090 GN=Abcb8 PE=1 SV=1	MOUSE	1
2738	2.01	2.02	18.50	5.91	sp|Q91WE2|PIP30_MOUSE	PSME3-interacting protein OS=Mus musculus OX=10090 GN=Psme3ip1 PE=1 SV=1	MOUSE	1
2739	2.01	2.02	4.23	1.33	tr|Q61213|Q61213_MOUSE	Protease OS=Mus musculus OX=10090 GN=gag PE=2 SV=1	MOUSE	1

2740	2.01	2.02	23.53	8.24	tr|A0A1L1SU40|A0A1L1SU40_MOUSE	Choline transporter-like protein 2 (Fragment) OS=Mus musculus OX=10090 GN=Slc44a2 PE=1 SV=1	MOUSE	1
2741	2.01	2.01	6.25	2.39	tr|Q6ZQC2|Q6ZQC2_MOUSE	MKIAA0410 protein (Fragment) OS=Mus musculus OX=10090 GN=mKIAA0410 PE=2 SV=1	MOUSE	1
2742	2.01	2.01	5.77	1.75	tr|Q3UJN9|Q3UJN9_MOUSE	Coilin OS=Mus musculus OX=10090 GN=Coil PE=2 SV=1	MOUSE	1
2743	2.01	2.01	23.32	7.77	tr|Q3U8S0|Q3U8S0_MOUSE	Adaptor-related protein complex 3, sigma 1 subunit OS=Mus musculus OX=10090 GN=Ap3s1 PE=1 SV=1	MOUSE	1
2744	2.01	2.01	59.26	17.28	tr|F8WH46|F8WH46_MOUSE	RIKEN cDNA 0610010K14 gene OS=Mus musculus OX=10090 GN=0610010K14Rik PE=1 SV=1	MOUSE	1
2745	2.01	2.01	8.76	4.68	tr|Q9EQC8|Q9EQC8_MOUSE	Papillary Renal Cell carcinoma (Translocation-associated) OS=Mus musculus OX=10090 GN=Prcc PE=1 SV=1	MOUSE	1
2746	2.01	2.01	10.04	7.34	tr|Q3TAQ8|Q3TAQ8_MOUSE	Phosphoglycerate mutase OS=Mus musculus OX=10090 GN=Bpgm PE=2 SV=1	MOUSE	1
2747	2.01	2.01	40.71	10.62	tr|G3UZA7|G3UZA7_MOUSE	Small ubiquitin-related modifier OS=Mus musculus OX=10090 GN=Sumo3 PE=1 SV=1	MOUSE	1
2748	2.01	2.01	11.48	8.13	tr|D3Z7W0|D3Z7W0_MOUSE	Iron-sulfur cluster assembly enzyme ISCU, mitochondrial OS=Mus musculus OX=10090 GN=Iscu PE=1 SV=1	MOUSE	1
2749	2.01	2.01	20.22	9.55	sp|Q9CZH7|MXRA7_MOUSE	Matrix-remodeling-associated protein 7 OS=Mus musculus OX=10090 GN=Mxra7 PE=1 SV=2	MOUSE	1
2750	2.01	2.01	19.71	12.50	sp|Q8BVA2|TM222_MOUSE	Transmembrane protein 222 OS=Mus musculus OX=10090 GN=Tmem222 PE=1 SV=1	MOUSE	1
2751	2.01	2.01	8.25	3.44	sp|Q8BHE8|MAIP1_MOUSE	m-AAA protease-interacting protein 1, mitochondrial OS=Mus musculus OX=10090 GN=Maip1 PE=1 SV=1	MOUSE	1
2752	2.01	2.01	66.67	44.05	sp|Q8BGH7|C42S2_MOUSE	CDC42 small effector protein 2 OS=Mus musculus OX=10090 GN=Cdc42se2 PE=1 SV=1	MOUSE	1
2753	2	96.32	52.09	44.09	sp|Q80TJ1-2|CAPS1_MOUSE	Isoform 2 of Calcium-dependent secretion activator 1 OS=Mus musculus OX=10090 GN=Cadps	MOUSE	50
2754	2	77.47	52.59	50.35	tr|A0A0A6YY47|A0A0A6YY47_MOUSE	Neural cell adhesion molecule 1 OS=Mus musculus OX=10090 GN=Ncam1 PE=1 SV=1	MOUSE	55
2755	2	70.31	55.08	52.65	sp|Q8BHN3|GANAB_MOUSE	Neutral alpha-glucosidase AB OS=Mus musculus OX=10090 GN=Ganab PE=1 SV=1	MOUSE	41
2756	2	61.18	43.26	34.16	tr|E9Q175|E9Q175_MOUSE	Unconventional myosin-6 OS=Mus musculus OX=10090 GN=Myo6 PE=1 SV=1	MOUSE	32

2757	2	58.15	66.29	63.78	sp|P61979-3|HNRPK_MOUSE	Isoform 3 of Heterogeneous nuclear ribonucleoprotein K OS=Mus musculus OX=10090 GN=Hnrnpk	MOUSE	36
2758	2	47.14	55.56	48.76	sp|D3Z7P3-2|GLSK_MOUSE	Isoform 2 of Glutaminase kidney isoform, mitochondrial OS=Mus musculus OX=10090 GN=Gls	MOUSE	28
2759	2	35.22	54.43	44.65	tr|A2BI12|A2BI12_MOUSE	PC4 and SFRS1-interacting protein OS=Mus musculus OX=10090 GN=Psip1 PE=1 SV=1	MOUSE	20
2760	2	26.51	62.99	62.99	tr|Q3UBN8|Q3UBN8_MOUSE	Uncharacterized protein OS=Mus musculus OX=10090 GN=Bpnt1 PE=2 SV=1	MOUSE	14
2761	2	25.96	63.16	55.26	tr|D3Z6G3|D3Z6G3_MOUSE	Microtubule-associated protein RP/EB family member 3 OS=Mus musculus OX=10090 GN=Mapre3 PE=1 SV=1	MOUSE	15
2762	2	25.6	54.34	54.06	tr|Q8K3G7|Q8K3G7_MOUSE	Reticulon OS=Mus musculus OX=10090 GN=Rtn4 PE=2 SV=1	MOUSE	13
2763	2	23.8	43.03	38.70	tr|Q6ZWM8|Q6ZWM8_MOUSE	Serine/threonine-protein phosphatase OS=Mus musculus OX=10090 GN=Ppp1cc PE=2 SV=1	MOUSE	12
2764	2	22	55.99	55.34	sp|P62715|PP2AB_MOUSE	Serine/threonine-protein phosphatase 2A catalytic subunit beta isoform OS=Mus musculus OX=10090 GN=Ppp2cb PE=1 SV=1	MOUSE	15
2765	2	15.08	46.13	33.87	tr|Q544I1|Q544I1_MOUSE	N-myc downstream-regulated gene 3 protein OS=Mus musculus OX=10090 GN=Ndrg3 PE=1 SV=1	MOUSE	9
2766	2	8.94	42.77	42.77	tr|I1E4X7|I1E4X7_MOUSE	Diphosphoinositol polyphosphate phosphohydrolase 1 OS=Mus musculus OX=10090 GN=Nudt3 PE=1 SV=1	MOUSE	5
2767	2	8.26	18.10	13.47	sp|Q925E7|2ABD_MOUSE	Serine/threonine-protein phosphatase 2A 55 kDa regulatory subunit B delta isoform OS=Mus musculus OX=10090 GN=Ppp2r2d PE=1 SV=1	MOUSE	4
2768	2	7.3	10.72	8.50	tr|Q8C762|Q8C762_MOUSE	Tyrosine-protein kinase OS=Mus musculus OX=10090 GN=Yes1 PE=2 SV=1	MOUSE	4
2769	2	7.22	33.33	33.33	tr|Q4FJR7|Q4FJR7_MOUSE	Eukaryotic translation initiation factor 4C OS=Mus musculus OX=10090 GN=Eif1a PE=2 SV=1	MOUSE	4
2770	2	6	35.42	34.38	tr|Q3TVP0|Q3TVP0_MOUSE	Uncharacterized protein OS=Mus musculus OX=10090 PE=2 SV=1	MOUSE	3
2771	2	6	49.44	49.44	sp|P63168|DYL1_MOUSE	Dynein light chain 1, cytoplasmic OS=Mus musculus OX=10090 GN=Dynll1 PE=1 SV=1	MOUSE	3
2772	2	4.53	22.63	20.00	tr|Q3UX06|Q3UX06_MOUSE	Uncharacterized protein (Fragment) OS=Mus musculus OX=10090 GN=Parp1 PE=2 SV=1	MOUSE	3

2773	2	4	23.17	10.57	tr|Q9Z1R9|Q9Z1R9_MOUSE	Protease, serine 1 (trypsin 1) OS=Mus musculus OX=10090 GN=Prss1 PE=1 SV=1	MOUSE	6
2774	2	4	10.55	10.09	tr|Q549X4|Q549X4_MOUSE	Small monomeric GTPase OS=Mus musculus OX=10090 GN=Rab27b PE=1 SV=1	MOUSE	2
2775	2	3.96	3.65	3.65	tr|E9QNA7|E9QNA7_MOUSE	Sorbin and SH3 domain-containing protein 1 OS=Mus musculus OX=10090 GN=Sorbs1 PE=1 SV=1	MOUSE	2
2776	2	3.8	6.06	6.06	tr|Q3UFK0|Q3UFK0_MOUSE	Uncharacterized protein OS=Mus musculus OX=10090 GN=Emb PE=2 SV=1	MOUSE	2
2777	2	3.14	27.55	25.51	tr|D3Z4G2|D3Z4G2_MOUSE	Homeobox protein OTX2 (Fragment) OS=Mus musculus OX=10090 GN=Otx2 PE=1 SV=1	MOUSE	3
2778	2	2.07	11.05	11.05	tr|Q497V8|Q497V8_MOUSE	2310061I04Rik protein OS=Mus musculus OX=10090 GN=2310061I04Rik PE=2 SV=1	MOUSE	2
2779	2	2.03	5.97	2.10	tr|Q6P8N8|Q6P8N8_MOUSE	ATP-dependent Clp protease ATP-binding subunit clpX-like, mitochondrial OS=Mus musculus OX=10090 GN=Clpx PE=1 SV=1	MOUSE	1
2780	2	2.03	2.21	0.65	tr|B9EIX2|B9EIX2_MOUSE	AW555464 protein OS=Mus musculus OX=10090 GN=Cep170b PE=1 SV=1	MOUSE	1
2781	2	2.02	36.90	28.57	tr|A0A3B2WBC6|A0A3B2WBC6_MOUSE	DNA-directed RNA polymerase II subunit GRINL1A OS=Mus musculus OX=10090 GN=Polr2m PE=4 SV=1	MOUSE	1
2782	2	2.01	4.08	0.97	sp|Q9DBR7|MYPT1_MOUSE	Protein phosphatase 1 regulatory subunit 12A OS=Mus musculus OX=10090 GN=Ppp1r12a PE=1 SV=2	MOUSE	1
2783	2	2.01	12.65	3.34	sp|Q91WM2|HDHD5_MOUSE	Haloacid dehalogenase-like hydrolase domain-containing 5 OS=Mus musculus OX=10090 GN=Hdhd5 PE=1 SV=1	MOUSE	1
2784	2	2.01	1.67	0.43	tr|A0A5F8MPX2|A0A5F8MPX2_MOUSE	Synaptotagmin-like protein 2 OS=Mus musculus OX=10090 GN=Sytl2 PE=1 SV=1	MOUSE	1
2785	2	2.01	6.43	4.83	tr|D3Z1C5|D3Z1C5_MOUSE	LIM domain-binding protein 1 OS=Mus musculus OX=10090 GN=Ldb1 PE=1 SV=1	MOUSE	1
2786	2	2.01	34.02	27.84	tr|A0A1L1SQA2|A0A1L1SQA2_MOUSE	Predicted gene 9803 OS=Mus musculus OX=10090 GN=Gm9803 PE=1 SV=1	MOUSE	1
2787	2	2.01	6.65	3.80	sp|Q9CWI3|BCCIP_MOUSE	BRCA2 and CDKN1A-interacting protein OS=Mus musculus OX=10090 GN=Bccip PE=1 SV=1	MOUSE	1
2788	2	2.01	7.69	4.81	sp|Q8CJ61|CKLF4_MOUSE	CKLF-like MARVEL transmembrane domain-containing protein 4 OS=Mus musculus OX=10090 GN=Cmtm4 PE=1 SV=1	MOUSE	1

2789	2	2.01	16.45	9.21	sp|Q80Y14|GLRX5_MOUSE	Glutaredoxin-related protein 5, mitochondrial OS=Mus musculus OX=10090 GN=Glrx5 PE=1 SV=2	MOUSE	1
2790	2	2	7.94	4.11	tr|Q3U3B9|Q3U3B9_MOUSE	BSD domain-containing protein OS=Mus musculus OX=10090 GN=Syap1 PE=2 SV=1	MOUSE	1
2791	2	2	5.41	3.70	tr|A0A286YDV7|A0A286YDV7_MOUSE	Nucleolar and coiled-body phosphoprotein 1 (Fragment) OS=Mus musculus OX=10090 GN=Nolc1 PE=1 SV=1	MOUSE	2
2792	2	2	12.27	6.36	sp|Q9WVB0-2|RBPMS_MOUSE	Isoform 2 of RNA-binding protein with multiple splicing OS=Mus musculus OX=10090 GN=Rbpms	MOUSE	1
2793	2	2	3.56	1.43	sp|Q9DC29|ABCB6_MOUSE	ATP-binding cassette sub-family B member 6 OS=Mus musculus OX=10090 GN=Abcb6 PE=1 SV=1	MOUSE	1
2794	2	2	3.42	3.42	tr|W0SMJ3|W0SMJ3_MOUSE	Cytochrome b (Fragment) OS=Mus musculus OX=10090 GN=cytb PE=3 SV=1	MOUSE	2
2795	2	2	4.02	3.65	tr|Q99KL8|Q99KL8_MOUSE	Nqo1 protein OS=Mus musculus OX=10090 GN=Nqo1 PE=2 SV=1	MOUSE	1
2796	2	2	4.21	2.97	tr|Q05CC9|Q05CC9_MOUSE	Porcn protein (Fragment) OS=Mus musculus OX=10090 GN=Porcn PE=2 SV=1	MOUSE	1
2797	2	2	19.47	8.85	tr|E9PW43|E9PW43_MOUSE	Predicted pseudogene 10320 OS=Mus musculus OX=10090 GN=Gm10320 PE=3 SV=1	MOUSE	1
2798	2	2	13.71	6.09	tr|D3Z7S0|D3Z7S0_MOUSE	Golgi to ER traffic protein 4 homolog (Fragment) OS=Mus musculus OX=10090 GN=Get4 PE=1 SV=1	MOUSE	1
2799	2	2	5.58	3.46	tr|A2ASL8|A2ASL8_MOUSE	Endoplasmic reticulum junction formation protein lunapark OS=Mus musculus OX=10090 GN=Lnpk PE=1 SV=1	MOUSE	1
2800	2	2	20.00	19.00	tr|A0A0A6YY66|A0A0A6YY66_MOUSE	Vesicle-associated membrane protein 4 OS=Mus musculus OX=10090 GN=Vamp4 PE=1 SV=1	MOUSE	1
2801	2	2	42.31	25.64	tr|A0A0A6YW87|A0A0A6YW87_MOUSE	Protein cornichon homolog 4 OS=Mus musculus OX=10090 GN=Cnih4 PE=1 SV=1	MOUSE	1
2802	2	2	24.84	24.84	sp|Q9WVK0|ATRAP_MOUSE	Type-1 angiotensin II receptor-associated protein OS=Mus musculus OX=10090 GN=Agtrap PE=1 SV=2	MOUSE	1
2803	2	2	6.07	3.04	sp|Q9QXE7|TBL1X_MOUSE	F-box-like/WD repeat-containing protein TBL1X OS=Mus musculus OX=10090 GN=Tbl1x PE=1 SV=2	MOUSE	1
2804	2	2	8.33	5.56	sp|Q9CQU5|ZWINT_MOUSE	ZW10 interactor OS=Mus musculus OX=10090 GN=Zwint PE=1 SV=1	MOUSE	1

2805	2	2	9.05	6.58	sp|Q9CQK7|RWDD1_MOUSE	RWD domain-containing protein 1 OS=Mus musculus OX=10090 GN=Rwdd1 PE=1 SV=1	MOUSE	1
2806	2	2	5.45	2.37	sp|Q922R1|PHAF1_MOUSE	Phagosome assembly factor 1 OS=Mus musculus OX=10090 GN=Phaf1 PE=1 SV=2	MOUSE	1
2807	2	2	4.27	2.85	sp|Q8VE80|THOC3_MOUSE	THO complex subunit 3 OS=Mus musculus OX=10090 GN=Thoc3 PE=2 SV=1	MOUSE	1
2808	2	2	1.94	1.04	sp|Q05909|PTPRG_MOUSE	Receptor-type tyrosine-protein phosphatase gamma OS=Mus musculus OX=10090 GN=Ptprg PE=1 SV=1	MOUSE	1
2809	2	2	6.53	6.53	tr|Q9D1X6|Q9D1X6_MOUSE	UPAR/Ly6 domain-containing protein OS=Mus musculus OX=10090 GN=Ly6h PE=2 SV=1	MOUSE	1
2810	2	2	23.40	23.40	tr|Q9CUX0|Q9CUX0_MOUSE	Uncharacterized protein OS=Mus musculus OX=10090 GN=N6amt1 PE=2 SV=2	MOUSE	1
2811	2	2	2.76	2.76	tr|Q99LZ8|Q99LZ8_MOUSE	Single stranded DNA binding protein 4 OS=Mus musculus OX=10090 GN=Ssbp4 PE=2 SV=1	MOUSE	1
2812	2	2	13.82	13.82	tr|Q920G6|Q920G6_MOUSE	MAC-inhibitory protein OS=Mus musculus OX=10090 GN=CD59A PE=4 SV=1	MOUSE	1
2813	2	2	10.00	10.00	tr|Q8R3W2|Q8R3W2_MOUSE	RIKEN cDNA 0610009B22 gene OS=Mus musculus OX=10090 GN=0610009B22Rik PE=2 SV=1	MOUSE	1
2814	2	2	9.55	9.55	tr|Q8R0Q6|Q8R0Q6_MOUSE	Tspan7 protein OS=Mus musculus OX=10090 GN=Tspan7 PE=2 SV=1	MOUSE	1
2815	2	2	5.91	5.91	tr|Q8CAD1|Q8CAD1_MOUSE	Ras-binding protein DA-Raf OS=Mus musculus OX=10090 GN=Araf PE=1 SV=1	MOUSE	1
2816	2	2	25.35	25.35	tr|Q8BT77|Q8BT77_MOUSE	Uncharacterized protein (Fragment) OS=Mus musculus OX=10090 GN=Chchd7 PE=2 SV=2	MOUSE	1
2817	2	2	13.04	13.04	tr|Q7GIP5|Q7GIP5_MOUSE	NADH-ubiquinone oxidoreductase chain 3 OS=Mus musculus OX=10090 GN=mt-Nd3 PE=2 SV=1	MOUSE	2
2818	2	2	21.69	21.69	tr|Q545S6|Q545S6_MOUSE	Transcription elongation factor 1 homolog OS=Mus musculus OX=10090 GN=Elof1 PE=2 SV=1	MOUSE	1
2819	2	2	14.29	14.29	tr|Q545K0|Q545K0_MOUSE	Complex I-MWFE OS=Mus musculus OX=10090 GN=Ndufa1 PE=1 SV=1	MOUSE	1
2820	2	2	6.52	6.52	tr|Q4V9T7|Q4V9T7_MOUSE	Replication termination factor 2 OS=Mus musculus OX=10090 GN=Rtf2 PE=2 SV=1	MOUSE	1
2821	2	2	7.57	7.57	tr|Q4FK79|Q4FK79_MOUSE	Signal sequence receptor subunit gamma OS=Mus musculus OX=10090 GN=Ssr3 PE=2 SV=1	MOUSE	1

2822	2	2	10.28	10.28	tr|Q3V460|Q3V460_MOUSE	Gene model 561, (NCBI) OS=Mus musculus OX=10090 GN=Smim26 PE=1 SV=1	MOUSE	1
2823	2	2	3.49	3.49	tr|Q3UYC1|Q3UYC1_MOUSE	Diphosphomevalonate decarboxylase OS=Mus musculus OX=10090 GN=Mvd PE=1 SV=1	MOUSE	1
2824	2	2	35.29	35.29	tr|Q3UJV6|Q3UJV6_MOUSE	Uncharacterized protein OS=Mus musculus OX=10090 GN=Uqcr11 PE=2 SV=1	MOUSE	2
2825	2	2	4.72	4.72	tr|Q3UDP8|Q3UDP8_MOUSE	UBC core domain-containing protein OS=Mus musculus OX=10090 GN=Ube2j1 PE=2 SV=1	MOUSE	1
2826	2	2	5.52	5.52	tr|Q3U466|Q3U466_MOUSE	Ceroid-lipofuscinosis, neuronal 6 OS=Mus musculus OX=10090 GN=Cln6 PE=1 SV=1	MOUSE	1
2827	2	2	9.92	9.92	tr|Q3U3K5|Q3U3K5_MOUSE	Uncharacterized protein OS=Mus musculus OX=10090 GN=Leprotl1 PE=2 SV=1	MOUSE	1
2828	2	2	1.53	1.53	tr|Q3TR20|Q3TR20_MOUSE	Component of oligomeric Golgi complex 7 OS=Mus musculus OX=10090 GN=Cog7 PE=1 SV=1	MOUSE	1
2829	2	2	2.73	2.73	tr|Q14C53|Q14C53_MOUSE	Solute carrier family 39 (Zinc transporter), member 7 OS=Mus musculus OX=10090 GN=Slc39a7 PE=2 SV=1	MOUSE	1
2830	2	2	25.00	25.00	tr|Q14AR0|Q14AR0_MOUSE	RRM domain-containing protein OS=Mus musculus OX=10090 GN=Slirp PE=2 SV=1	MOUSE	1
2831	2	2	25.45	25.45	tr|F6XV67|F6XV67_MOUSE	RIKEN cDNA 2410004B18 gene (Fragment) OS=Mus musculus OX=10090 GN=2410004B18Rik PE=1 SV=1	MOUSE	1
2832	2	2	7.84	7.84	tr|F6VF36|F6VF36_MOUSE	Signal recognition particle receptor ('docking protein') (Fragment) OS=Mus musculus OX=10090 GN=Srpr PE=1 SV=1	MOUSE	1
2833	2	2	0.35	0.35	tr|E9Q9C6|E9Q9C6_MOUSE	Fc fragment of IgG-binding protein OS=Mus musculus OX=10090 GN=Fcgbp PE=1 SV=1	MOUSE	1
2834	2	2	16.44	16.44	tr|D3Z7P2|D3Z7P2_MOUSE	Transmembrane protein 109 (Fragment) OS=Mus musculus OX=10090 GN=Tmem109 PE=1 SV=1	MOUSE	1
2835	2	2	3.52	3.52	tr|D3Z6S7|D3Z6S7_MOUSE	Immunoglobulin superfamily member 11 OS=Mus musculus OX=10090 GN=Igsf11 PE=1 SV=1	MOUSE	1
2836	2	2	12.57	12.57	tr|D3Z0K7|D3Z0K7_MOUSE	WAS/WASL-interacting protein family member 2 (Fragment) OS=Mus musculus OX=10090 GN=Wipf2 PE=1 SV=1	MOUSE	1
2837	2	2	12.97	12.97	tr|A2A6P4|A2A6P4_MOUSE	Family with sequence similarity 104, member A OS=Mus musculus OX=10090 GN=Fam104a PE=1 SV=1	MOUSE	1

2838	2	2	14.04	14.04	tr|A0A1Y7VLP0|A0A1Y7VLP0_MOUSE	ATP synthase membrane subunit 6.8PL (Fragment) OS=Mus musculus OX=10090 GN=Atp5mpl PE=1 SV=1	MOUSE	1
2839	2	2	12.67	12.67	tr|A0A1B0GQW0|A0A1B0GQW0_MOUSE	ER lumen protein-retaining receptor OS=Mus musculus OX=10090 GN=Kdelr1 PE=1 SV=1	MOUSE	1
2840	2	2	17.46	17.46	tr|A0A0U1RNU5|A0A0U1RNU5_MOUSE	RIKEN cDNA E130218I03 gene OS=Mus musculus OX=10090 GN=E130218I03Rik PE=4 SV=1	MOUSE	1
2841	2	2	20.93	20.93	tr|A0A0N4SWI4|A0A0N4SWI4_MOUSE	Protein FAM136A OS=Mus musculus OX=10090 GN=Fam136a PE=1 SV=1	MOUSE	1
2842	2	2	4.01	4.01	sp|Q9QUN9|DKK3_MOUSE	Dickkopf-related protein 3 OS=Mus musculus OX=10090 GN=Dkk3 PE=2 SV=1	MOUSE	1
2843	2	2	2.90	2.90	sp|Q9D8B7|JAM3_MOUSE	Junctional adhesion molecule C OS=Mus musculus OX=10090 GN=Jam3 PE=1 SV=2	MOUSE	1
2844	2	2	4.45	4.45	sp|Q9D4F2|PLPP6_MOUSE	Phospholipid phosphatase 6 OS=Mus musculus OX=10090 GN=Plpp6 PE=1 SV=1	MOUSE	1
2845	2	2	6.80	6.80	sp|Q9D3P8|PLRKT_MOUSE	Plasminogen receptor (KT) OS=Mus musculus OX=10090 GN=Plgrkt PE=1 SV=1	MOUSE	1
2846	2	2	7.10	7.10	sp|Q9CR23|TMEM9_MOUSE	Proton-transporting V-type ATPase complex assembly regulator TMEM9 OS=Mus musculus OX=10090 GN=Tmem9 PE=1 SV=1	MOUSE	1
2847	2	2	4.14	4.14	sp|Q9CQJ2|PIHD1_MOUSE	PIH1 domain-containing protein 1 OS=Mus musculus OX=10090 GN=Pih1d1 PE=1 SV=1	MOUSE	1
2848	2	2	20.43	20.43	sp|Q91VW3|SH3L3_MOUSE	SH3 domain-binding glutamic acid-rich-like protein 3 OS=Mus musculus OX=10090 GN=Sh3bgrl3 PE=1 SV=1	MOUSE	1
2849	2	2	18.32	18.32	sp|Q8K273|EMC5_MOUSE	ER membrane protein complex subunit 5 OS=Mus musculus OX=10090 GN=Mmgt1 PE=1 SV=1	MOUSE	1
2850	2	2	9.17	9.17	sp|Q8CIB6|TM230_MOUSE	Transmembrane protein 230 OS=Mus musculus OX=10090 GN=Tmem230 PE=1 SV=1	MOUSE	1
2851	2	2	9.65	9.65	sp|Q8CCI5|RYBP_MOUSE	RING1 and YY1-binding protein OS=Mus musculus OX=10090 GN=Rybp PE=1 SV=1	MOUSE	1
2852	2	2	13.75	13.75	sp|Q8BTE5|CEBOS_MOUSE	Protein CEBPZOS OS=Mus musculus OX=10090 GN=Cebpzos PE=3 SV=2	MOUSE	1

2853	2	2	2.95	2.95	sp|Q8BPS4|GP180_MOUSE	Integral membrane protein GPR180 OS=Mus musculus OX=10090 GN=Gpr180 PE=1 SV=1	MOUSE	1
2854	2	2	8.05	8.05	sp|Q78XF5|OSTC_MOUSE	Oligosaccharyltransferase complex subunit OSTC OS=Mus musculus OX=10090 GN=Ostc PE=1 SV=1	MOUSE	1
2855	2	2	6.67	6.67	sp|Q6ZWQ7|SPCS3_MOUSE	Signal peptidase complex subunit 3 OS=Mus musculus OX=10090 GN=Spcs3 PE=1 SV=1	MOUSE	1
2856	2	2	24.78	24.78	sp|Q5F285|TM256_MOUSE	Transmembrane protein 256 OS=Mus musculus OX=10090 GN=Tmem256 PE=1 SV=1	MOUSE	1
2857	2	2	14.41	14.41	sp|P62774|MTPN_MOUSE	Myotrophin OS=Mus musculus OX=10090 GN=Mtpn PE=1 SV=2	MOUSE	1
2858	1.98	2.13	3.52	0.52	sp|Q8CC88|VWA8_MOUSE	von Willebrand factor A domain-containing protein 8 OS=Mus musculus OX=10090 GN=Vwa8 PE=1 SV=2	MOUSE	1
2859	1.98	2.08	16.62	4.83	sp|O54786|DFFA_MOUSE	DNA fragmentation factor subunit alpha OS=Mus musculus OX=10090 GN=Dffa PE=1 SV=2	MOUSE	1
2860	1.98	2.04	9.87	1.92	sp|Q3TDD9|PPR21_MOUSE	Protein phosphatase 1 regulatory subunit 21 OS=Mus musculus OX=10090 GN=Ppp1r21 PE=1 SV=2	MOUSE	1
2861	1.98	2.02	65.28	12.50	tr|E0CY38|E0CY38_MOUSE	Ubiquitin-like protein 5 OS=Mus musculus OX=10090 GN=Ubl5 PE=4 SV=1	MOUSE	1
2862	1.97	2.13	19.69	5.00	tr|Q544H0|Q544H0_MOUSE	Eukaryotic translation initiation factor 3 subunit G OS=Mus musculus OX=10090 GN=Eif3g PE=1 SV=1	MOUSE	1
2863	1.97	2.11	9.77	4.77	sp|Q3TPE9|ANKY2_MOUSE	Ankyrin repeat and MYND domain-containing protein 2 OS=Mus musculus OX=10090 GN=Ankmy2 PE=1 SV=1	MOUSE	2
2864	1.97	2.01	2.86	1.84	sp|Q9EPL2|CSTN1_MOUSE	Calsyntenin-1 OS=Mus musculus OX=10090 GN=Clstn1 PE=1 SV=1	MOUSE	1
2865	1.96	2.19	5.47	2.17	tr|Q7TSA7|Q7TSA7_MOUSE	Ppp1r9a protein OS=Mus musculus OX=10090 GN=Ppp1r9a PE=2 SV=1	MOUSE	1
2866	1.96	2.11	13.87	11.31	tr|Q4FJZ6|Q4FJZ6_MOUSE	GCS light chain OS=Mus musculus OX=10090 GN=Gclm PE=1 SV=1	MOUSE	2
2867	1.96	2.1	9.61	3.74	sp|Q8CFS6|KCNV2_MOUSE	Potassium voltage-gated channel subfamily V member 2 OS=Mus musculus OX=10090 GN=Kcnv2 PE=2 SV=1	MOUSE	2
2868	1.96	2.08	18.96	4.19	tr|Q7TPN5|Q7TPN5_MOUSE	Wiskott-Aldrich syndrome-like (Human) OS=Mus musculus OX=10090 GN=Wasl PE=2 SV=1	MOUSE	1
2869	1.96	2.01	4.43	2.39	tr|Q8K2R0|Q8K2R0_MOUSE	Farp1 protein (Fragment) OS=Mus musculus OX=10090 GN=Farp1 PE=2 SV=1	MOUSE	2

2870	1.95	5.31	8.89	2.45	tr|Q3UIZ0|Q3UIZ0_MOUSE	Uncharacterized protein OS=Mus musculus OX=10090 GN=Gak PE=2 SV=1	MOUSE	4
2871	1.94	2.07	7.28	2.71	tr|Q3TKH5|Q3TKH5_MOUSE	TRAMP-like complex RNA-binding factor ZCCHC8 (Fragment) OS=Mus musculus OX=10090 GN=Zcchc8 PE=2 SV=1	MOUSE	1
2872	1.94	2.02	4.02	1.28	tr|Q80T83|Q80T83_MOUSE	Ubiquitinyl hydrolase 1 (Fragment) OS=Mus musculus OX=10090 GN=Vcpip1 PE=2 SV=1	MOUSE	1
2873	1.93	7.69	18.29	9.43	sp|Q8R4E6|PURG_MOUSE	Purine-rich element-binding protein gamma OS=Mus musculus OX=10090 GN=Purg PE=1 SV=1	MOUSE	6
2874	1.93	2.03	3.66	1.16	sp|Q8BUH8|SENP7_MOUSE	Sentrin-specific protease 7 OS=Mus musculus OX=10090 GN=Senp7 PE=1 SV=1	MOUSE	1
2875	1.92	2.25	8.96	2.46	tr|H9KV00|H9KV00_MOUSE	Protein SON OS=Mus musculus OX=10090 GN=Son PE=1 SV=1	MOUSE	2
2876	1.92	2	6.48	1.44	tr|E9Q481|E9Q481_MOUSE	Serine/threonine-protein phosphatase 4 regulatory subunit 3A OS=Mus musculus OX=10090 GN=Ppp4r3a PE=1 SV=1	MOUSE	1
2877	1.91	2.11	8.33	3.72	sp|O55106|STRN_MOUSE	Striatin OS=Mus musculus OX=10090 GN=Strn PE=1 SV=2	MOUSE	2
2878	1.9	2.07	27.70	3.05	tr|Q80WF9|Q80WF9_MOUSE	Ceh-10 homeodomain-containing homolog OS=Mus musculus OX=10090 GN=Vsx2 PE=2 SV=2	MOUSE	1
2879	1.9	2.06	4.05	0.99	tr|B2RQG2|B2RQG2_MOUSE	PHD finger protein 3 OS=Mus musculus OX=10090 GN=Phf3 PE=1 SV=1	MOUSE	2
2880	1.89	2.01	5.66	1.85	sp|Q69ZF3|GBA2_MOUSE	Non-lysosomal glucosylceramidase OS=Mus musculus OX=10090 GN=Gba2 PE=1 SV=2	MOUSE	1
2881	1.89	1.98	2.18	1.09	tr|B2RY05|B2RY05_MOUSE	Non-specific serine/threonine protein kinase OS=Mus musculus OX=10090 GN=Pik3r4 PE=2 SV=1	MOUSE	1
2882	1.88	12.4	43.25	30.16	tr|G3UYJ7|G3UYJ7_MOUSE	Predicted gene 20441 (Fragment) OS=Mus musculus OX=10090 GN=Gm20441 PE=3 SV=1	MOUSE	6
2883	1.88	6.23	13.64	9.47	tr|Q3TQ93|Q3TQ93_MOUSE	Uncharacterized protein OS=Mus musculus OX=10090 GN=Rab33b PE=2 SV=1	MOUSE	3
2884	1.88	2.63	11.16	4.46	sp|Q8CHW4|EI2BE_MOUSE	Translation initiation factor eIF-2B subunit epsilon OS=Mus musculus OX=10090 GN=Eif2b5 PE=1 SV=1	MOUSE	3
2885	1.87	76.48	53.18	44.87	sp|Q60597|ODO1_MOUSE	2-oxoglutarate dehydrogenase, mitochondrial OS=Mus musculus OX=10090 GN=Ogdh PE=1 SV=3	MOUSE	38

2886	1.87	2.01	24.93	11.81	sp|Q8BIF9|ZN787_MOUSE	Zinc finger protein 787 OS=Mus musculus OX=10090 GN=Znf787 PE=2 SV=3	MOUSE	2
2887	1.87	1.99	6.55	2.56	tr|Q3UXU8|Q3UXU8_MOUSE	CST complex subunit STN1 OS=Mus musculus OX=10090 GN=Stn1 PE=1 SV=1	MOUSE	1
2888	1.87	1.94	2.93	0.72	sp|O88307|SORL_MOUSE	Sortilin-related receptor OS=Mus musculus OX=10090 GN=Sorl1 PE=1 SV=3	MOUSE	1
2889	1.87	1.94	9.88	7.19	sp|Q9R0D8|WDR54_MOUSE	WD repeat-containing protein 54 OS=Mus musculus OX=10090 GN=Wdr54 PE=1 SV=1	MOUSE	1
2890	1.86	2.05	15.96	6.49	sp|Q80ZW0|STK35_MOUSE	Serine/threonine-protein kinase 35 OS=Mus musculus OX=10090 GN=Stk35 PE=1 SV=3	MOUSE	2
2891	1.86	1.93	5.72	2.06	sp|Q8K2I1|FNTB_MOUSE	Protein farnesyltransferase subunit beta OS=Mus musculus OX=10090 GN=Fntb PE=1 SV=1	MOUSE	1
2892	1.85	71.31	87.20	86.40	tr|Q4KL81|Q4KL81_MOUSE	Actin, gamma, cytoplasmic 1 OS=Mus musculus OX=10090 GN=Actg1 PE=2 SV=1	MOUSE	88
2893	1.85	41.14	62.84	51.49	sp|O88935-1|SYN1_MOUSE	Isoform Ib of Synapsin-1 OS=Mus musculus OX=10090 GN=Syn1	MOUSE	34
2894	1.85	12.52	30.69	27.80	sp|P97300-3|NPTN_MOUSE	Isoform 3 of Neuroplastin OS=Mus musculus OX=10090 GN=Nptn	MOUSE	7
2895	1.85	4.26	9.59	4.12	tr|Q6A074|Q6A074_MOUSE	MKIAA0400 protein (Fragment) OS=Mus musculus OX=10090 GN=Asap2 PE=2 SV=1	MOUSE	3
2896	1.85	2.06	28.99	12.56	tr|B1AX95|B1AX95_MOUSE	Transformation-related protein 63-regulated-like OS=Mus musculus OX=10090 GN=Tprgl PE=1 SV=1	MOUSE	2
2897	1.85	2.03	14.85	3.33	tr|Q8C197|Q8C197_MOUSE	Glyco_transf_64 domain-containing protein OS=Mus musculus OX=10090 GN=Extl2 PE=2 SV=1	MOUSE	2
2898	1.85	1.99	4.45	4.45	sp|Q5DTY9|KCD16_MOUSE	BTB/POZ domain-containing protein KCTD16 OS=Mus musculus OX=10090 GN=Kctd16 PE=1 SV=2	MOUSE	2
2899	1.85	1.96	6.81	3.29	tr|Q3UNC0|Q3UNC0_MOUSE	NTP_transferase domain-containing protein (Fragment) OS=Mus musculus OX=10090 GN=Gmppa PE=2 SV=1	MOUSE	1
2900	1.85	1.91	7.78	3.42	tr|Q3UTZ4|Q3UTZ4_MOUSE	ATP-dependent dihydroxyacetone kinase OS=Mus musculus OX=10090 GN=Tkfc PE=1 SV=1	MOUSE	1
2901	1.84	2.02	13.45	1.42	sp|Q8C7E9|CSTFT_MOUSE	Cleavage stimulation factor subunit 2 tau variant OS=Mus musculus OX=10090 GN=Cstf2t PE=1 SV=2	MOUSE	1
2902	1.84	1.98	14.75	2.81	tr|Q684Q6|Q684Q6_MOUSE	55 kDa erythrocyte membrane protein (Fragment) OS=Mus musculus OX=10090 GN=Mpp1 PE=2 SV=1	MOUSE	1

2903	1.84	1.96	13.26	3.58	tr|D3YUE4|D3YUE4_MOUSE	Family with sequence similarity 151, member B OS=Mus musculus OX=10090 GN=Fam151b PE=1 SV=1	MOUSE	1
2904	1.84	1.96	20.57	6.38	sp|Q8WUR0|CS012_MOUSE	Protein C19orf12 homolog OS=Mus musculus OX=10090 PE=1 SV=1	MOUSE	1
2905	1.84	1.94	7.93	3.13	tr|Q9QUI3|Q9QUI3_MOUSE	Casein kinase OS=Mus musculus OX=10090 GN=Csnk1e PE=2 SV=1	MOUSE	1
2906	1.83	1.96	8.41	8.41	tr|Q7JCY9|Q7JCY9_MOUSE	ATP synthase subunit a OS=Mus musculus OX=10090 GN=ATP6 PE=1 SV=1	MOUSE	2
2907	1.83	1.94	4.32	1.96	sp|Q8BTG3|T11L1_MOUSE	T-complex protein 11-like protein 1 OS=Mus musculus OX=10090 GN=Tcp11l1 PE=1 SV=1	MOUSE	1
2908	1.82	6.19	17.32	10.24	tr|Q921L9|Q921L9_MOUSE	Protein kinase, cAMP dependent regulatory, type I beta OS=Mus musculus OX=10090 GN=Prkar1b PE=2 SV=1	MOUSE	4
2909	1.82	2.19	6.78	4.76	tr|Q3TEP0|Q3TEP0_MOUSE	WD_REPEATS_REGION domain-containing protein OS=Mus musculus OX=10090 GN=Aaas PE=2 SV=1	MOUSE	2
2910	1.82	2.05	2.65	1.71	tr|A1L349|A1L349_MOUSE	Polynucleotide adenylyltransferase OS=Mus musculus OX=10090 GN=Papolb PE=1 SV=1	MOUSE	1
2911	1.82	1.92	2.19	2.19	tr|Q2M4G8|Q2M4G8_MOUSE	Protein tyrosine phosphatase, non-receptor type 9 OS=Mus musculus OX=10090 GN=Ptpn9 PE=1 SV=1	MOUSE	1
2912	1.82	1.91	13.44	5.38	sp|Q9CY16|RT28_MOUSE	28S ribosomal protein S28, mitochondrial OS=Mus musculus OX=10090 GN=Mrps28 PE=1 SV=1	MOUSE	1
2913	1.82	1.89	8.20	8.20	tr|Q8R3T4|Q8R3T4_MOUSE	Serum amyloid A protein OS=Mus musculus OX=10090 GN=Saa2 PE=2 SV=1	MOUSE	1
2914	1.82	1.89	11.88	11.88	sp|Q78T54|VMA21_MOUSE	Vacuolar ATPase assembly integral membrane protein Vma21 OS=Mus musculus OX=10090 GN=Vma21 PE=1 SV=1	MOUSE	1
2915	1.81	1.97	29.86	5.21	sp|Q8VHN8|TIRR_MOUSE	Tudor-interacting repair regulator protein OS=Mus musculus OX=10090 GN=Nudt16l1 PE=1 SV=2	MOUSE	1
2916	1.81	1.91	7.03	3.98	tr|Q99M50|Q99M50_MOUSE	Phospholipid scramblase OS=Mus musculus OX=10090 GN=Plscr1 PE=2 SV=1	MOUSE	1
2917	1.8	2	12.06	1.75	tr|Q3UI98|Q3UI98_MOUSE	Uncharacterized protein OS=Mus musculus OX=10090 GN=Gtf3c5 PE=2 SV=1	MOUSE	1
2918	1.8	1.89	14.13	6.36	sp|Q9CQ65|MTAP_MOUSE	S-methyl-5'-thioadenosine phosphorylase OS=Mus musculus OX=10090 GN=Mtap PE=1 SV=1	MOUSE	1

2919	1.8	1.89	15.56	15.56	tr|A0A2R8VHK6|A0A2R8VHK6_MOUSE	Essential MCU regulator, mitochondrial OS=Mus musculus OX=10090 GN=Smdt1 PE=1 SV=1	MOUSE	1
2920	1.8	1.89	2.04	2.04	sp|Q3U4G3|XXLT1_MOUSE	Xyloside xylosyltransferase 1 OS=Mus musculus OX=10090 GN=Xxylt1 PE=1 SV=2	MOUSE	1
2921	1.79	1.91	16.22	7.34	tr|A2AM95|A2AM95_MOUSE	Emerin OS=Mus musculus OX=10090 GN=Emd PE=4 SV=1	MOUSE	2
2922	1.79	1.87	17.28	3.70	sp|Q9QYI5|DNJB2_MOUSE	DnaJ homolog subfamily B member 2 OS=Mus musculus OX=10090 GN=Dnajb2 PE=1 SV=3	MOUSE	1
2923	1.78	2.07	17.70	3.35	tr|Q9DBU8|Q9DBU8_MOUSE	MHD domain-containing protein OS=Mus musculus OX=10090 GN=Ap3m1 PE=2 SV=1	MOUSE	1
2924	1.78	1.94	11.31	1.08	sp|Q91WG4|ELP2_MOUSE	Elongator complex protein 2 OS=Mus musculus OX=10090 GN=Elp2 PE=1 SV=1	MOUSE	1
2925	1.78	1.92	10.79	5.40	tr|E9QMN5|E9QMN5_MOUSE	Transcriptional repressor p66 alpha OS=Mus musculus OX=10090 GN=Gatad2a PE=1 SV=1	MOUSE	2
2926	1.78	1.88	7.48	1.69	sp|Q8C0L8|COG5_MOUSE	Conserved oligomeric Golgi complex subunit 5 OS=Mus musculus OX=10090 GN=Cog5 PE=1 SV=3	MOUSE	1
2927	1.78	1.87	11.00	1.92	sp|O35218|CPSF2_MOUSE	Cleavage and polyadenylation specificity factor subunit 2 OS=Mus musculus OX=10090 GN=Cpsf2 PE=1 SV=1	MOUSE	1
2928	1.78	1.87	4.36	0.75	tr|E9PYJ7|E9PYJ7_MOUSE	Membrane-associated phosphatidylinositol transfer protein 2 OS=Mus musculus OX=10090 GN=Pitpnm2 PE=1 SV=1	MOUSE	1
2929	1.77	3	16.89	4.55	sp|Q6P9R2|OXSR1_MOUSE	Serine/threonine-protein kinase OSR1 OS=Mus musculus OX=10090 GN=Oxsr1 PE=1 SV=1	MOUSE	2
2930	1.77	1.95	17.05	6.78	tr|Q80YP1|Q80YP1_MOUSE	Arginyl-tRNA--protein transferase 1 OS=Mus musculus OX=10090 GN=Ate1 PE=2 SV=1	MOUSE	2
2931	1.77	1.88	14.29	11.16	tr|Q5FWJ7|Q5FWJ7_MOUSE	Vesicle transport through interaction with t-SNAREs homolog 1A OS=Mus musculus OX=10090 GN=Vti1a PE=1 SV=1	MOUSE	1
2932	1.77	1.87	13.01	3.76	sp|Q69ZC8|GPAM1_MOUSE	GPALPP motifs-containing protein 1 OS=Mus musculus OX=10090 GN=Gpalpp1 PE=1 SV=2	MOUSE	1
2933	1.77	1.86	18.56	8.98	tr|Q3UEX1|Q3UEX1_MOUSE	Tubby protein (Fragment) OS=Mus musculus OX=10090 GN=Tub PE=1 SV=1	MOUSE	1
2934	1.77	1.85	4.15	2.17	sp|P23953|EST1C_MOUSE	Carboxylesterase 1C OS=Mus musculus OX=10090 GN=Ces1c PE=1 SV=4	MOUSE	1

2935	1.77	1.85	20.87	20.87	tr|Q3TYE9|Q3TYE9_MOUSE	Uncharacterized protein (Fragment) OS=Mus musculus OX=10090 GN=Reep2 PE=2 SV=1	MOUSE	1
2936	1.77	1.85	8.75	8.75	tr|F6R220|F6R220_MOUSE	GDP-Man:Man(3)GlcNAc(2)-PP-Dol alpha-1,2-mannosyltransferase (Fragment) OS=Mus musculus OX=10090 GN=Alg11 PE=1 SV=1	MOUSE	1
2937	1.76	3.7	6.06	2.54	sp|Q80TJ7|PHF8_MOUSE	Histone lysine demethylase PHF8 OS=Mus musculus OX=10090 GN=Phf8 PE=1 SV=2	MOUSE	2
2938	1.76	1.89	12.05	3.96	sp|Q8BJW6|EIF2A_MOUSE	Eukaryotic translation initiation factor 2A OS=Mus musculus OX=10090 GN=Eif2a PE=1 SV=2	MOUSE	2
2939	1.76	1.88	32.32	10.37	sp|Q9D967|MGDP1_MOUSE	Magnesium-dependent phosphatase 1 OS=Mus musculus OX=10090 GN=Mdp1 PE=1 SV=1	MOUSE	1
2940	1.76	1.86	20.29	6.52	sp|Q9DAU1|CNPY3_MOUSE	Protein canopy homolog 3 OS=Mus musculus OX=10090 GN=Cnpy3 PE=1 SV=1	MOUSE	1
2941	1.76	1.85	10.56	6.11	sp|Q9CYK1|SYWM_MOUSE	Tryptophan--tRNA ligase, mitochondrial OS=Mus musculus OX=10090 GN=Wars2 PE=1 SV=2	MOUSE	1
2942	1.76	1.84	3.67	0.64	tr|Q80SX9|Q80SX9_MOUSE	Kidins220 protein (Fragment) OS=Mus musculus OX=10090 GN=Kidins220 PE=2 SV=1	MOUSE	1
2943	1.75	1.9	7.02	4.26	tr|Q3UDP9|Q3UDP9_MOUSE	MFS domain-containing protein OS=Mus musculus OX=10090 GN=Slc16a3 PE=1 SV=1	MOUSE	2
2944	1.75	1.89	13.38	4.54	tr|E9PWK1|E9PWK1_MOUSE	Epoxide hydrolase OS=Mus musculus OX=10090 GN=Ephx1 PE=1 SV=1	MOUSE	2
2945	1.75	1.88	2.01	0.44	tr|G5E893|G5E893_MOUSE	Ankyrin repeat domain 12 OS=Mus musculus OX=10090 GN=Ankrd12 PE=1 SV=1	MOUSE	1
2946	1.74	2.15	16.28	12.62	sp|Q9QZH3|PPIE_MOUSE	Peptidyl-prolyl cis-trans isomerase E OS=Mus musculus OX=10090 GN=Ppie PE=1 SV=2	MOUSE	3
2947	1.74	1.82	11.32	11.32	tr|Q58DZ5|Q58DZ5_MOUSE	Mitochondrial ribosomal protein S33 OS=Mus musculus OX=10090 GN=Mrps33 PE=1 SV=1	MOUSE	1
2948	1.73	1.9	12.44	2.39	sp|Q9CZP5|BCS1_MOUSE	Mitochondrial chaperone BCS1 OS=Mus musculus OX=10090 GN=Bcs1l PE=1 SV=1	MOUSE	1
2949	1.73	1.8	5.98	3.63	sp|P28481|CO2A1_MOUSE	Collagen alpha-1(II) chain OS=Mus musculus OX=10090 GN=Col2a1 PE=1 SV=2	MOUSE	3
2950	1.73	1.8	5.01	2.90	tr|Q8C7Z5|Q8C7Z5_MOUSE	Uncharacterized protein OS=Mus musculus OX=10090 GN=Kcnj10 PE=2 SV=1	MOUSE	1

2951	1.72	16	49.74	49.21	sp|P60766-1|CDC42_MOUSE	Isoform 1 of Cell division control protein 42 homolog OS=Mus musculus OX=10090 GN=Cdc42	MOUSE	9
2952	1.72	2.11	16.13	1.47	tr|A2A9P6|A2A9P6_MOUSE	Cyclin-dependent kinase 11B OS=Mus musculus OX=10090 GN=Cdk11b PE=1 SV=1	MOUSE	1
2953	1.72	1.87	6.24	1.91	sp|Q8K2J0|PLCD3_MOUSE	1-phosphatidylinositol 4,5-bisphosphate phosphodiesterase delta-3 OS=Mus musculus OX=10090 GN=Plcd3 PE=1 SV=2	MOUSE	1
2954	1.72	1.8	7.25	4.93	tr|M1VMU3|M1VMU3_MOUSE	NADH-ubiquinone oxidoreductase chain 2 OS=Mus musculus OX=10090 GN=ND2 PE=3 SV=1	MOUSE	1
2955	1.71	3.39	22.88	11.44	sp|Q3UL36|ARGL1_MOUSE	Arginine and glutamate-rich protein 1 OS=Mus musculus OX=10090 GN=Arglu1 PE=1 SV=2	MOUSE	3
2956	1.71	1.88	10.24	3.18	tr|Q3V3A4|Q3V3A4_MOUSE	Vacuolar protein sorting-associated protein 52 homolog OS=Mus musculus OX=10090 GN=Vps52 PE=2 SV=1	MOUSE	2
2957	1.71	1.87	28.34	17.11	tr|A0A3Q4EGL6|A0A3Q4EGL6_MOUSE	Sulfhydryl oxidase OS=Mus musculus OX=10090 GN=Gfer PE=1 SV=1	MOUSE	3
2958	1.71	1.84	8.87	5.75	tr|Q8BYY1|Q8BYY1_MOUSE	Uncharacterized protein OS=Mus musculus OX=10090 GN=Ngfr PE=2 SV=1	MOUSE	1
2959	1.7	1.89	4.61	2.16	tr|B2RRC5|B2RRC5_MOUSE	RAB GTPase activating protein 1 OS=Mus musculus OX=10090 GN=Rabgap1 PE=2 SV=1	MOUSE	2
2960	1.7	1.8	7.35	1.28	tr|Q05DK2|Q05DK2_MOUSE	Ctr9 protein (Fragment) OS=Mus musculus OX=10090 GN=Ctr9 PE=2 SV=1	MOUSE	1
2961	1.7	1.8	3.02	1.04	tr|Q6NWW3|Q6NWW3_MOUSE	Grip1 protein OS=Mus musculus OX=10090 GN=Grip1 PE=2 SV=1	MOUSE	1
2962	1.7	1.77	20.35	11.50	tr|Q99KD6|Q99KD6_MOUSE	Cox7a2l protein OS=Mus musculus OX=10090 GN=Cox7a2l PE=2 SV=1	MOUSE	1
2963	1.69	1.85	12.03	1.65	tr|Q3TW27|Q3TW27_MOUSE	Negative elongation factor D OS=Mus musculus OX=10090 GN=Nelfcd PE=1 SV=1	MOUSE	1
2964	1.69	1.8	12.29	2.50	tr|Q3TET5|Q3TET5_MOUSE	Beta-MPP (Fragment) OS=Mus musculus OX=10090 GN=Pmpcb PE=2 SV=1	MOUSE	1
2965	1.69	1.8	12.65	6.53	tr|E0CYC4|E0CYC4_MOUSE	Cyclin-dependent kinase 8 (Fragment) OS=Mus musculus OX=10090 GN=Cdk8 PE=1 SV=1	MOUSE	1
2966	1.69	1.78	5.17	1.34	tr|A0A6I8MWZ0|A0A6I8MWZ0_MOUSE	Peptidylprolyl isomerase (Fragment) OS=Mus musculus OX=10090 GN=Fkbp15 PE=1 SV=1	MOUSE	1

2967	1.68	2.47	15.93	7.08	sp|Q6IFX2|K1C42_MOUSE	Keratin, type I cytoskeletal 42 OS=Mus musculus OX=10090 GN=Krt42 PE=1 SV=1	MOUSE	3
2968	1.68	1.93	11.87	3.58	sp|Q8VCW8|ACSF2_MOUSE	Medium-chain acyl-CoA ligase ACSF2, mitochondrial OS=Mus musculus OX=10090 GN=Acsf2 PE=1 SV=1	MOUSE	2
2969	1.68	1.82	11.67	2.38	tr|Q3UGE1|Q3UGE1_MOUSE	TPR_REGION domain-containing protein OS=Mus musculus OX=10090 GN=Trappc12 PE=2 SV=1	MOUSE	1
2970	1.68	1.77	6.98	1.66	tr|A0A0R4J0W6|A0A0R4J0W6_MOUSE	Leucine-rich repeat-containing protein 40 OS=Mus musculus OX=10090 GN=Lrrc40 PE=1 SV=1	MOUSE	1
2971	1.67	1.8	13.21	3.19	sp|Q9R0X4|ACOT9_MOUSE	Acyl-coenzyme A thioesterase 9, mitochondrial OS=Mus musculus OX=10090 GN=Acot9 PE=1 SV=1	MOUSE	1
2972	1.66	64.34	61.99	54.83	sp|P17879|HS71B_MOUSE	Heat shock 70 kDa protein 1B OS=Mus musculus OX=10090 GN=Hspa1b PE=1 SV=3	MOUSE	45
2973	1.65	4.85	6.50	3.03	tr|E9Q7E9|E9Q7E9_MOUSE	Phosphofurin acidic cluster sorting protein 2 OS=Mus musculus OX=10090 GN=Pacs2 PE=1 SV=1	MOUSE	3
2974	1.65	3.02	19.72	12.84	tr|Q3U1N3|Q3U1N3_MOUSE	Harvey rat sarcoma oncogene, subgroup R OS=Mus musculus OX=10090 GN=Rras PE=1 SV=1	MOUSE	2
2975	1.65	1.88	16.87	9.81	tr|A0A2K6EDK3|A0A2K6EDK3_MOUSE	NudC domain-containing protein 1 OS=Mus musculus OX=10090 GN=Nudcd1 PE=1 SV=1	MOUSE	3
2976	1.65	1.75	9.21	4.61	tr|E9Q156|E9Q156_MOUSE	tRNA (guanine-N(7)-)-methyltransferase non-catalytic subunit WDR4 OS=Mus musculus OX=10090 GN=Wdr4 PE=1 SV=1	MOUSE	1
2977	1.64	1.76	9.42	1.19	tr|Q8BWV2|Q8BWV2_MOUSE	Vacuolar protein sorting-associated protein 16 homolog OS=Mus musculus OX=10090 GN=Vps16 PE=2 SV=1	MOUSE	1
2978	1.64	1.72	4.52	4.52	tr|Q497T6|Q497T6_MOUSE	COMM domain containing 2 OS=Mus musculus OX=10090 GN=Commd2 PE=1 SV=1	MOUSE	1
2979	1.63	1.86	37.93	7.59	tr|Q14BF8|Q14BF8_MOUSE	Trafficking protein particle complex subunit OS=Mus musculus OX=10090 GN=Trappc1 PE=1 SV=1	MOUSE	1
2980	1.63	1.81	14.06	2.67	sp|Q8CHT0|AL4A1_MOUSE	Delta-1-pyrroline-5-carboxylate dehydrogenase, mitochondrial OS=Mus musculus OX=10090 GN=Aldh4a1 PE=1 SV=3	MOUSE	1
2981	1.63	1.76	7.48	1.15	tr|Q6GR78|Q6GR78_MOUSE	Amyloid-beta A4 protein OS=Mus musculus OX=10090 GN=App PE=2 SV=1	MOUSE	1
2982	1.63	1.75	2.62	0.54	tr|B2RSW8|B2RSW8_MOUSE	Pericentriolar material 1 OS=Mus musculus OX=10090 GN=Pcm1 PE=2 SV=1	MOUSE	1

2983	1.62	4.77	22.22	15.66	tr|Q3U281|Q3U281_MOUSE	Uncharacterized protein OS=Mus musculus OX=10090 GN=Sar1b PE=2 SV=1	MOUSE	3
2984	1.62	1.71	12.54	10.10	tr|Q62347|Q62347_MOUSE	Cyclic AMP-responsive element-binding protein 1 OS=Mus musculus OX=10090 GN=Creb1 PE=1 SV=1	MOUSE	1
2985	1.62	1.7	14.01	9.34	sp|Q8BQP9|R7BP_MOUSE	Regulator of G-protein signaling 7-binding protein OS=Mus musculus OX=10090 GN=Rgs7bp PE=1 SV=1	MOUSE	2
2986	1.62	1.7	3.05	1.60	tr|E9Q1U6|E9Q1U6_MOUSE	CBP80/20-dependent translation initiation factor OS=Mus musculus OX=10090 GN=Ctif PE=1 SV=1	MOUSE	1
2987	1.61	2.32	7.44	3.86	tr|Q8C0C2|Q8C0C2_MOUSE	USP domain-containing protein OS=Mus musculus OX=10090 GN=Usp11 PE=2 SV=1	MOUSE	2
2988	1.61	1.8	12.38	4.88	sp|Q61753|SERA_MOUSE	D-3-phosphoglycerate dehydrogenase OS=Mus musculus OX=10090 GN=Phgdh PE=1 SV=3	MOUSE	2
2989	1.61	1.76	24.35	18.26	sp|Q9CQ86|MIEN1_MOUSE	Migration and invasion enhancer 1 OS=Mus musculus OX=10090 GN=Mien1 PE=1 SV=1	MOUSE	2
2990	1.6	1.76	22.03	10.13	sp|Q9CR11|YETS4_MOUSE	YEATS domain-containing protein 4 OS=Mus musculus OX=10090 GN=Yeats4 PE=2 SV=1	MOUSE	2
2991	1.6	1.72	4.24	0.56	tr|E9QML5|E9QML5_MOUSE	Zinc finger protein 638 OS=Mus musculus OX=10090 GN=Zfp638 PE=1 SV=2	MOUSE	1
2992	1.6	1.7	5.73	2.20	tr|Q80X31|Q80X31_MOUSE	Sodium/hydrogen exchanger OS=Mus musculus OX=10090 GN=Slc9a1 PE=2 SV=1	MOUSE	1
2993	1.6	1.68	3.93	3.93	sp|Q80V26|IMPA3_MOUSE	Golgi-resident adenosine 3',5'-bisphosphate 3'-phosphatase OS=Mus musculus OX=10090 GN=Bpnt2 PE=1 SV=1	MOUSE	1
2994	1.6	1.67	6.43	1.64	tr|Q8BRU5|Q8BRU5_MOUSE	RNA helicase OS=Mus musculus OX=10090 GN=Ddx50 PE=2 SV=1	MOUSE	1
2995	1.59	3.89	5.05	3.39	sp|Q7TSH6-3|SCAF4_MOUSE	Isoform 3 of SR-related and CTD-associated factor 4 OS=Mus musculus OX=10090 GN=Scaf4	MOUSE	3
2996	1.59	3.37	24.29	15.00	tr|F6T4M4|F6T4M4_MOUSE	Serine/arginine repetitive matrix protein 1 (Fragment) OS=Mus musculus OX=10090 GN=Srrm1 PE=1 SV=1	MOUSE	2
2997	1.59	2.13	12.08	2.66	tr|J3QJU5|J3QJU5_MOUSE	LLGL scribble cell polarity complex component 2 OS=Mus musculus OX=10090 GN=Llgl2 PE=1 SV=1	MOUSE	1
2998	1.59	1.74	3.49	1.01	tr|A2AA71|A2AA71_MOUSE	Protein transport protein Sec24A OS=Mus musculus OX=10090 GN=Sec24a PE=1 SV=1	MOUSE	1

2999	1.59	1.72	20.08	7.20	tr|Q3TDL0|Q3TDL0_MOUSE	PCI domain-containing protein OS=Mus musculus OX=10090 GN=Cops7b PE=2 SV=1	MOUSE	1
3000	1.59	1.69	1.89	0.73	sp|Q8K448|ABCA5_MOUSE	Cholesterol transporter ABCA5 OS=Mus musculus OX=10090 GN=Abca5 PE=1 SV=2	MOUSE	1
3001	1.58	7.05	17.25	12.66	tr|Q6ZQK4|Q6ZQK4_MOUSE	MKIAA0044 protein (Fragment) OS=Mus musculus OX=10090 GN=Ppp2r5c PE=2 SV=1	MOUSE	4
3002	1.58	1.68	13.64	3.79	tr|G3X9M0|G3X9M0_MOUSE	28S ribosomal protein S29, mitochondrial OS=Mus musculus OX=10090 GN=Dap3 PE=1 SV=1	MOUSE	1
3003	1.58	1.67	8.25	4.29	tr|Q6NSU0|Q6NSU0_MOUSE	Protein FAM3C (Fragment) OS=Mus musculus OX=10090 GN=Fam3c PE=2 SV=2	MOUSE	1
3004	1.57	1.72	3.66	1.32	tr|F6VBV0|F6VBV0_MOUSE	Regulating synaptic membrane exocytosis protein 1 OS=Mus musculus OX=10090 GN=Rims1 PE=1 SV=3	MOUSE	1
3005	1.57	1.69	15.49	2.89	tr|Q9JJQ6|Q9JJQ6_MOUSE	Factor 8-associated gene A OS=Mus musculus OX=10090 GN=F8a PE=1 SV=1	MOUSE	1
3006	1.57	1.66	5.17	2.01	tr|Z4YMT0|Z4YMT0_MOUSE	TATA box-binding protein-like 2 (Fragment) OS=Mus musculus OX=10090 GN=Tbpl2 PE=3 SV=1	MOUSE	1
3007	1.57	1.64	4.08	4.08	RRRRRsp|Q3KQJ0|TMM69_MOUSE	REVERSED Transmembrane protein 69 OS=Mus musculus OX=10090 GN=Tmem69 PE=2 SV=2	MOUSE	1
3008	1.56	1.69	16.25	5.00	tr|Q8BLQ6|Q8BLQ6_MOUSE	AP complex subunit sigma OS=Mus musculus OX=10090 GN=Ap1s2 PE=2 SV=1	MOUSE	1
3009	1.56	1.66	8.11	2.51	tr|Q9Z162|Q9Z162_MOUSE	Vascular endothelial zinc finger 1 OS=Mus musculus OX=10090 GN=Vezf1 PE=2 SV=1	MOUSE	1
3010	1.55	1.68	18.47	3.36	tr|Q8BVU9|Q8BVU9_MOUSE	Collagen-binding protein OS=Mus musculus OX=10090 GN=Serpinh1 PE=2 SV=1	MOUSE	1
3011	1.55	1.66	7.20	2.13	tr|Q3U2Z5|Q3U2Z5_MOUSE	SH3 domain-containing protein OS=Mus musculus OX=10090 GN=Eps8 PE=2 SV=1	MOUSE	1
3012	1.55	1.64	4.83	4.83	tr|Q6PB99|Q6PB99_MOUSE	Ena/VASP-like protein OS=Mus musculus OX=10090 GN=Evl PE=2 SV=1	MOUSE	1
3013	1.55	1.64	8.78	8.78	sp|Q99N92|RM27_MOUSE	39S ribosomal protein L27, mitochondrial OS=Mus musculus OX=10090 GN=Mrpl27 PE=1 SV=1	MOUSE	1
3014	1.55	1.62	5.82	3.88	tr|E9QKB2|E9QKB2_MOUSE	Intracellular hyaluronan-binding protein 4 OS=Mus musculus OX=10090 GN=Habp4 PE=1 SV=1	MOUSE	1
3015	1.54	1.71	25.00	9.72	tr|B7ZNW0|B7ZNW0_MOUSE	Pgam5 protein OS=Mus musculus OX=10090 GN=Pgam5 PE=2 SV=1	MOUSE	3

3016	1.54	1.63	10.87	6.25	sp|Q8BIG4|FBX28_MOUSE	F-box only protein 28 OS=Mus musculus OX=10090 GN=Fbxo28 PE=1 SV=1	MOUSE	1
3017	1.54	1.62	1.38	0.89	tr|A0A087WSD2|A0A087WSD2_MOUSE	Membrane-spanning 4-domains, subfamily A, member 14 OS=Mus musculus OX=10090 GN=Ms4a14 PE=3 SV=1	MOUSE	1
3018	1.54	1.62	17.92	7.51	sp|Q8C838|TARG1_MOUSE	Trafficking regulator of GLUT4 1 OS=Mus musculus OX=10090 GN=Trarg1 PE=1 SV=1	MOUSE	1
3019	1.53	1.62	11.66	4.71	sp|Q8R3E3|WIPI1_MOUSE	WD repeat domain phosphoinositide-interacting protein 1 OS=Mus musculus OX=10090 GN=Wipi1 PE=1 SV=1	MOUSE	1
3020	1.53	1.61	6.19	4.19	tr|F6SQM4|F6SQM4_MOUSE	Protein FAM234A OS=Mus musculus OX=10090 GN=Fam234a PE=1 SV=2	MOUSE	1
3021	1.52	1.7	24.76	15.53	tr|B1AUZ1|B1AUZ1_MOUSE	N-alpha-acetyltransferase 10 OS=Mus musculus OX=10090 GN=Naa10 PE=1 SV=1	MOUSE	2
3022	1.52	1.6	8.04	8.04	tr|D3Z497|D3Z497_MOUSE	Predicted gene 7075 OS=Mus musculus OX=10090 GN=Gm7075 PE=3 SV=1	MOUSE	1
3023	1.52	1.6	9.09	9.09	sp|Q9CQW0|EMC6_MOUSE	ER membrane protein complex subunit 6 OS=Mus musculus OX=10090 GN=Emc6 PE=1 SV=1	MOUSE	1
3024	1.51	1.65	11.71	2.24	tr|Q80YG4|Q80YG4_MOUSE	SEC63 OS=Mus musculus OX=10090 GN=Sec63 PE=2 SV=1	MOUSE	1
3025	1.51	1.65	15.27	8.78	tr|F6WMX7|F6WMX7_MOUSE	Calcium uptake protein 3, mitochondrial (Fragment) OS=Mus musculus OX=10090 GN=Micu3 PE=1 SV=1	MOUSE	3
3026	1.51	1.63	7.21	2.56	sp|Q8K099|LRIT1_MOUSE	Leucine-rich repeat, immunoglobulin-like domain and transmembrane domain-containing protein 1 OS=Mus musculus OX=10090 GN=Lrit1 PE=2 SV=2	MOUSE	1
3027	1.51	1.6	14.47	14.47	tr|B2RVQ7|B2RVQ7_MOUSE	Zinc finger protein 706 OS=Mus musculus OX=10090 GN=Zfp706 PE=1 SV=1	MOUSE	1
3028	1.5	1.75	7.75	2.58	tr|Q80UI9|Q80UI9_MOUSE	Wolfram syndrome 1 homolog (Human) OS=Mus musculus OX=10090 GN=Wfs1 PE=2 SV=1	MOUSE	2
3029	1.5	1.63	4.54	4.54	tr|Q3UX26|Q3UX26_MOUSE	Uncharacterized protein (Fragment) OS=Mus musculus OX=10090 GN=Pak1ip1 PE=2 SV=1	MOUSE	2
3030	1.49	1.65	7.00	1.08	sp|O54991|CNTP1_MOUSE	Contactin-associated protein 1 OS=Mus musculus OX=10090 GN=Cntnap1 PE=1 SV=2	MOUSE	1
3031	1.49	1.58	9.09	2.17	sp|Q9ET22|DPP2_MOUSE	Dipeptidyl peptidase 2 OS=Mus musculus OX=10090 GN=Dpp7 PE=1 SV=2	MOUSE	1

3032	1.48	10.3	10.56	2.71	sp|G3X9K3|BIG1_MOUSE	Brefeldin A-inhibited guanine nucleotide-exchange protein 1 OS=Mus musculus OX=10090 GN=Arfgef1 PE=1 SV=1	MOUSE	5
3033	1.48	2.14	11.28	3.76	tr|Q8BS26|Q8BS26_MOUSE	Uncharacterized protein OS=Mus musculus OX=10090 GN=Akt1 PE=2 SV=1	MOUSE	1
3034	1.48	1.62	7.75	7.75	tr|E0CY81|E0CY81_MOUSE	Predicted readthrough transcript (NMD candidate), 44505 OS=Mus musculus OX=10090 GN=Gm44505 PE=1 SV=1	MOUSE	2
3035	1.48	1.58	7.47	3.14	sp|Q99M31|HSP7E_MOUSE	Heat shock 70 kDa protein 14 OS=Mus musculus OX=10090 GN=Hspa14 PE=1 SV=2	MOUSE	1
3036	1.48	1.57	10.09	4.32	tr|Q71VB4|Q71VB4_MOUSE	PBX1b OS=Mus musculus OX=10090 GN=Pbx1 PE=2 SV=1	MOUSE	1
3037	1.48	1.57	4.29	4.29	tr|E3WH30|E3WH30_MOUSE	Meiosis protein 5 homolog OS=Mus musculus OX=10090 GN=Sfr1 PE=2 SV=1	MOUSE	1
3038	1.47	1.62	9.14	7.21	tr|Q80YC0|Q80YC0_MOUSE	Sel1l protein (Fragment) OS=Mus musculus OX=10090 GN=Sel1l PE=2 SV=1	MOUSE	2
3039	1.47	1.57	10.00	2.40	tr|A2AQJ7|A2AQJ7_MOUSE	Transmembrane protein 87A OS=Mus musculus OX=10090 GN=Tmem87a PE=1 SV=1	MOUSE	1
3040	1.47	1.56	25.90	9.35	tr|F7CCI4|F7CCI4_MOUSE	Female-lethal(2)D homolog (Fragment) OS=Mus musculus OX=10090 GN=Wtap PE=1 SV=1	MOUSE	1
3041	1.47	1.55	17.71	17.71	tr|A0A2I3BRK6|A0A2I3BRK6_MOUSE	Transcription and mRNA export factor ENY2 OS=Mus musculus OX=10090 GN=Eny2 PE=1 SV=1	MOUSE	1
3042	1.46	1.6	14.40	14.40	tr|Q80UU0|Q80UU0_MOUSE	Golgi SNAP receptor complex member 1 OS=Mus musculus OX=10090 GN=Gosr1 PE=2 SV=1	MOUSE	2
3043	1.46	1.54	55.22	40.30	tr|D3Z7Q3|D3Z7Q3_MOUSE	Guanine nucleotide-binding protein subunit gamma OS=Mus musculus OX=10090 GN=Gng5 PE=1 SV=1	MOUSE	1
3044	1.46	1.54	4.03	4.03	tr|Q4KL28|Q4KL28_MOUSE	Granzyme G OS=Mus musculus OX=10090 GN=Gzmg PE=2 SV=1	MOUSE	1
3045	1.46	1.54	13.79	13.79	tr|G3UW70|G3UW70_MOUSE	Late endosomal/lysosomal adaptor and MAPK and MTOR activator 5 OS=Mus musculus OX=10090 GN=Lamtor5 PE=1 SV=1	MOUSE	1
3046	1.45	4.92	7.49	3.91	tr|Q6AXB7|Q6AXB7_MOUSE	Synaptic functional regulator FMR1 OS=Mus musculus OX=10090 GN=Fmr1 PE=1 SV=1	MOUSE	3
3047	1.44	1.68	6.11	0.88	sp|Q6GQT1|A2MG_MOUSE	Alpha-2-macroglobulin-P OS=Mus musculus OX=10090 GN=A2m PE=2 SV=2	MOUSE	1

3048	1.44	1.62	6.04	1.98	tr|A0A0X1KG60|A0A0X1KG60_MOUSE	E3 ubiquitin protein ligase OS=Mus musculus OX=10090 GN=Rnf40 PE=1 SV=1	MOUSE	2
3049	1.44	1.59	6.71	1.19	tr|Q8BND1|Q8BND1_MOUSE	cGMP-dependent protein kinase OS=Mus musculus OX=10090 GN=Prkg1 PE=2 SV=1	MOUSE	1
3050	1.44	1.52	2.56	1.15	tr|Q5U4B1|Q5U4B1_MOUSE	Replication factor C subunit 1 OS=Mus musculus OX=10090 GN=Rfc1 PE=1 SV=1	MOUSE	1
3051	1.44	1.52	4.68	4.68	sp|Q99J39|DCMC_MOUSE	Malonyl-CoA decarboxylase, mitochondrial OS=Mus musculus OX=10090 GN=Mlycd PE=1 SV=1	MOUSE	1
3052	1.44	1.52	6.39	6.39	sp|Q3V038|TTC9A_MOUSE	Tetratricopeptide repeat protein 9A OS=Mus musculus OX=10090 GN=Ttc9 PE=1 SV=1	MOUSE	1
3053	1.43	3.63	21.50	8.14	tr|Q3UNF2|Q3UNF2_MOUSE	Serine/threonine-protein phosphatase OS=Mus musculus OX=10090 GN=Ppp4c PE=1 SV=1	MOUSE	2
3054	1.43	1.55	27.45	12.75	tr|A0A2I3BRW0|A0A2I3BRW0_MOUSE	Microsomal signal peptidase 12 kDa subunit OS=Mus musculus OX=10090 GN=Spcs1 PE=1 SV=1	MOUSE	1
3055	1.43	1.52	6.08	1.52	sp|Q3UIL6|PKHA7_MOUSE	Pleckstrin homology domain-containing family A member 7 OS=Mus musculus OX=10090 GN=Plekha7 PE=1 SV=2	MOUSE	1
3056	1.43	1.52	9.00	9.00	sp|O09114|PTGDS_MOUSE	Prostaglandin-H2 D-isomerase OS=Mus musculus OX=10090 GN=Ptgds PE=1 SV=1	MOUSE	1
3057	1.43	1.51	4.31	2.15	sp|Q91Z92|B3GT6_MOUSE	Beta-1,3-galactosyltransferase 6 OS=Mus musculus OX=10090 GN=B3galt6 PE=2 SV=1	MOUSE	1
3058	1.43	1.51	4.11	1.88	sp|Q8R3Y8|I2BP1_MOUSE	Interferon regulatory factor 2-binding protein 1 OS=Mus musculus OX=10090 GN=Irf2bp1 PE=1 SV=2	MOUSE	1
3059	1.43	1.5	3.32	2.06	tr|E9Q5M4|E9Q5M4_MOUSE	Zinc finger RNA-binding protein 2 OS=Mus musculus OX=10090 GN=Zfr2 PE=1 SV=1	MOUSE	1
3060	1.42	1.58	11.66	4.24	tr|G3XA48|G3XA48_MOUSE	Isopentenyl-diphosphate Delta-isomerase OS=Mus musculus OX=10090 GN=Idi1 PE=1 SV=1	MOUSE	1
3061	1.42	1.52	12.64	3.72	tr|Q5I940|Q5I940_MOUSE	USE1-like protein (Fragment) OS=Mus musculus OX=10090 GN=Use1 PE=2 SV=1	MOUSE	1
3062	1.42	1.51	9.07	5.01	sp|Q8BYN3|ITPK1_MOUSE	Inositol-tetrakisphosphate 1-kinase OS=Mus musculus OX=10090 GN=Itpk1 PE=2 SV=1	MOUSE	1
3063	1.41	1.55	5.36	1.28	sp|Q6ZPE2|MTMR5_MOUSE	Myotubularin-related protein 5 OS=Mus musculus OX=10090 GN=Sbf1 PE=1 SV=2	MOUSE	2

3064	1.41	1.53	8.99	4.61	tr|B9EIE9|B9EIE9_MOUSE	Adenylosuccinate synthetase isozyme 2 OS=Mus musculus OX=10090 GN=Adss PE=1 SV=1	MOUSE	1
3065	1.41	1.53	20.00	4.50	sp|Q9JM14|NT5C_MOUSE	5'(3')-deoxyribonucleotidase, cytosolic type OS=Mus musculus OX=10090 GN=Nt5c PE=1 SV=1	MOUSE	1
3066	1.4	1.56	3.39	0.97	sp|Q920R0|ALS2_MOUSE	Alsin OS=Mus musculus OX=10090 GN=Als2 PE=1 SV=3	MOUSE	1
3067	1.4	1.54	21.57	9.80	sp|Q9CPZ6|ORML3_MOUSE	ORM1-like protein 3 OS=Mus musculus OX=10090 GN=Ormdl3 PE=2 SV=1	MOUSE	1
3068	1.4	1.54	2.24	2.24	sp|Q7TMI3|UHRF2_MOUSE	E3 ubiquitin-protein ligase UHRF2 OS=Mus musculus OX=10090 GN=Uhrf2 PE=1 SV=1	MOUSE	2
3069	1.4	1.52	6.79	2.91	sp|Q6PHU5|SORT_MOUSE	Sortilin OS=Mus musculus OX=10090 GN=Sort1 PE=1 SV=1	MOUSE	2
3070	1.4	1.49	7.34	2.45	tr|A0A024QYR9|A0A024QYR9_MOUSE	Phosphatidylinositol-3,4,5-trisphosphate 3-phosphatase OS=Mus musculus OX=10090 GN=Pten PE=2 SV=1	MOUSE	1
3071	1.4	1.49	7.69	4.00	tr|A0A0A0MQF5|A0A0A0MQF5_MOUSE	Ubiquitinyl hydrolase 1 OS=Mus musculus OX=10090 GN=Otud6b PE=1 SV=1	MOUSE	1
3072	1.4	1.48	24.84	7.19	tr|Q9JIF6|Q9JIF6_MOUSE	Geranylgeranyl transferase type-2 subunit beta OS=Mus musculus OX=10090 GN=Rabggtb PE=1 SV=1	MOUSE	1
3073	1.4	1.48	2.61	1.68	tr|Q6GQW1|Q6GQW1_MOUSE	Slc4a1ap protein (Fragment) OS=Mus musculus OX=10090 GN=Slc4a1ap PE=2 SV=1	MOUSE	1
3074	1.38	1.52	13.27	1.55	sp|P97364|SPS2_MOUSE	Selenide, water dikinase 2 OS=Mus musculus OX=10090 GN=Sephs2 PE=1 SV=3	MOUSE	1
3075	1.38	1.47	3.31	2.25	tr|Q8K0E2|Q8K0E2_MOUSE	Exocyst complex component 3 OS=Mus musculus OX=10090 GN=Exoc3 PE=1 SV=1	MOUSE	1
3076	1.38	1.47	13.59	8.74	tr|Q3U0H6|Q3U0H6_MOUSE	Uncharacterized protein (Fragment) OS=Mus musculus OX=10090 GN=Tmem199 PE=2 SV=1	MOUSE	1
3077	1.38	1.46	6.24	6.24	sp|Q8K070|SAM14_MOUSE	Sterile alpha motif domain-containing protein 14 OS=Mus musculus OX=10090 GN=Samd14 PE=1 SV=1	MOUSE	3
3078	1.37	1.69	10.62	9.33	sp|Q8K0G5|EIPR1_MOUSE	EARP and GARP complex-interacting protein 1 OS=Mus musculus OX=10090 GN=Eipr1 PE=1 SV=2	MOUSE	2
3079	1.37	1.48	3.32	0.68	sp|O35126|ATN1_MOUSE	Atrophin-1 OS=Mus musculus OX=10090 GN=Atn1 PE=1 SV=1	MOUSE	1

3080	1.37	1.44	5.67	3.12	tr|Q8CJ24|Q8CJ24_MOUSE	Brain mitochondrial carrier protein short-inserted form OS=Mus musculus OX=10090 GN=Slc25a14 PE=2 SV=2	MOUSE	1
3081	1.37	1.44	4.78	4.78	tr|Q3U4R0|Q3U4R0_MOUSE	Secretory carrier-associated membrane protein OS=Mus musculus OX=10090 GN=Scamp4 PE=2 SV=1	MOUSE	1
3082	1.37	1.44	25.71	25.71	tr|H3BLR9|H3BLR9_MOUSE	Ribonuclease kappa OS=Mus musculus OX=10090 GN=Rnasek PE=4 SV=1	MOUSE	1
3083	1.37	1.44	4.67	4.67	sp|Q9EQQ2|YIPF5_MOUSE	Protein YIPF5 OS=Mus musculus OX=10090 GN=Yipf5 PE=1 SV=1	MOUSE	1
3084	1.36	1.56	10.74	2.13	tr|A0A0R4J150|A0A0R4J150_MOUSE	Oxysterol-binding protein OS=Mus musculus OX=10090 GN=Osbpl8 PE=1 SV=1	MOUSE	1
3085	1.36	1.45	13.08	2.82	sp|Q8CE96|TRM6_MOUSE	tRNA (adenine(58)-N(1))-methyltransferase non-catalytic subunit TRM6 OS=Mus musculus OX=10090 GN=Trmt6 PE=1 SV=1	MOUSE	1
3086	1.35	1.47	8.56	1.82	tr|B8X349|B8X349_MOUSE	JNK-interacting leucine zipper protein long form OS=Mus musculus OX=10090 GN=Spag9 PE=2 SV=1	MOUSE	1
3087	1.35	1.44	12.37	6.19	tr|A0A0G2JGE1|A0A0G2JGE1_MOUSE	Cysteine-S-conjugate beta-lyase 2 (Fragment) OS=Mus musculus OX=10090 GN=Kyat3 PE=1 SV=1	MOUSE	1
3088	1.35	1.43	10.08	6.98	tr|Q0VF50|Q0VF50_MOUSE	Complement component 1, q subcomponent-like 1 OS=Mus musculus OX=10090 GN=C1ql1 PE=2 SV=1	MOUSE	1
3089	1.34	36.44	40.27	32.67	tr|Q3U484|Q3U484_MOUSE	RNA helicase OS=Mus musculus OX=10090 GN=Ddx3y PE=2 SV=1	MOUSE	20
3090	1.34	16	67.59	48.15	tr|Q9DB48|Q9DB48_MOUSE	Uncharacterized protein OS=Mus musculus OX=10090 GN=Rab2b PE=2 SV=1	MOUSE	10
3091	1.34	1.49	8.47	1.32	sp|Q9QXW9|LAT2_MOUSE	Large neutral amino acids transporter small subunit 2 OS=Mus musculus OX=10090 GN=Slc7a8 PE=1 SV=1	MOUSE	1
3092	1.34	1.48	12.16	12.16	sp|Q9D142|NUD14_MOUSE	Uridine diphosphate glucose pyrophosphatase NUDT14 OS=Mus musculus OX=10090 GN=Nudt14 PE=1 SV=1	MOUSE	2
3093	1.34	1.43	7.28	4.11	tr|Q6NS54|Q6NS54_MOUSE	Na(+)/H(+) exchange regulatory cofactor NHE-RF OS=Mus musculus OX=10090 GN=Slc9a3r2 PE=1 SV=1	MOUSE	1
3094	1.34	1.43	21.51	12.79	tr|Q545H3|Q545H3_MOUSE	Mitochondrial import inner membrane translocase subunit TIM17 OS=Mus musculus OX=10090 GN=Timm17b PE=1 SV=1	MOUSE	1
3095	1.34	1.42	4.81	2.03	sp|Q9R0N6-2|SYT8_MOUSE	Isoform 2 of Synaptotagmin-8 OS=Mus musculus OX=10090 GN=Syt8	MOUSE	1

3096	1.33	1.45	15.78	3.41	sp|O35654|DPOD2_MOUSE	DNA polymerase delta subunit 2 OS=Mus musculus OX=10090 GN=Pold2 PE=1 SV=2	MOUSE	1
3097	1.33	1.41	4.40	2.78	tr|Q3U2U4|Q3U2U4_MOUSE	S5 DRBM domain-containing protein OS=Mus musculus OX=10090 GN=Mrps5 PE=2 SV=1	MOUSE	1
3098	1.32	23.13	35.55	21.96	tr|B7U582|B7U582_MOUSE	Heat shock protein 70-2 OS=Mus musculus OX=10090 PE=3 SV=1	MOUSE	23
3099	1.32	1.44	8.33	3.41	tr|A0A0R4J215|A0A0R4J215_MOUSE	Coiled-coil domain-containing protein 75 OS=Mus musculus OX=10090 GN=Gpatch11 PE=1 SV=1	MOUSE	1
3100	1.32	1.42	2.63	1.39	tr|E9QMK9|E9QMK9_MOUSE	D-glutamate cyclase, mitochondrial OS=Mus musculus OX=10090 GN=Dglucy PE=1 SV=1	MOUSE	1
3101	1.31	18.79	45.51	21.16	tr|B2RTL5|B2RTL5_MOUSE	Aldehyde dehydrogenase family 1, subfamily A7 OS=Mus musculus OX=10090 GN=Aldh1a7 PE=1 SV=1	MOUSE	11
3102	1.31	1.45	4.94	2.66	sp|Q920A7|AFG31_MOUSE	AFG3-like protein 1 OS=Mus musculus OX=10090 GN=Afg3l1 PE=1 SV=2	MOUSE	2
3103	1.3	1.38	5.99	5.99	tr|Q8C521|Q8C521_MOUSE	RING-type E3 ubiquitin transferase OS=Mus musculus OX=10090 GN=Rnf114 PE=2 SV=1	MOUSE	1
3104	1.3	1.37	5.64	1.59	tr|G3UZF7|G3UZF7_MOUSE	(E2-independent) E3 ubiquitin-conjugating enzyme FATS OS=Mus musculus OX=10090 GN=D7Ertd443e PE=1 SV=1	MOUSE	1
3105	1.29	5.56	17.24	11.33	tr|A0A068BFS4|A0A068BFS4_MOUSE	E3 ubiquitin-protein ligase RING1 OS=Mus musculus OX=10090 GN=Ring1 PE=2 SV=1	MOUSE	3
3106	1.29	1.48	24.53	4.35	sp|Q9D9V3|ECHD1_MOUSE	Ethylmalonyl-CoA decarboxylase OS=Mus musculus OX=10090 GN=Echdc1 PE=1 SV=2	MOUSE	1
3107	1.29	1.48	7.35	3.99	sp|Q9JLR1|S61A2_MOUSE	Protein transport protein Sec61 subunit alpha isoform 2 OS=Mus musculus OX=10090 GN=Sec61a2 PE=2 SV=3	MOUSE	2
3108	1.28	1.4	14.42	4.81	tr|Q3TPX5|Q3TPX5_MOUSE	Uncharacterized protein OS=Mus musculus OX=10090 GN=Mras PE=1 SV=1	MOUSE	1
3109	1.27	1.38	4.51	0.94	tr|Q3UR91|Q3UR91_MOUSE	Pumilio homolog 2 OS=Mus musculus OX=10090 GN=Pum2 PE=1 SV=1	MOUSE	1
3110	1.27	1.38	7.75	2.91	tr|Q8CAQ9|Q8CAQ9_MOUSE	Exocyst complex component Sec8 OS=Mus musculus OX=10090 GN=Exoc4 PE=2 SV=1	MOUSE	1
3111	1.27	1.35	36.54	15.38	tr|Q6VEU5|Q6VEU5_MOUSE	H+ transporting F1 ATP synthase epsilon subunit OS=Mus musculus OX=10090 GN=Atp5e PE=2 SV=1	MOUSE	1
3112	1.27	1.35	9.85	4.93	tr|Q3UEK9|Q3UEK9_MOUSE	Alpha-2-HS-glycoprotein OS=Mus musculus OX=10090 GN=Ahsg PE=1 SV=1	MOUSE	1

3113	1.27	1.35	26.97	26.97	sp|O54962|BAF_MOUSE	Barrier-to-autointegration factor OS=Mus musculus OX=10090 GN=Banf1 PE=1 SV=1	MOUSE	1
3114	1.26	2.04	11.71	7.28	tr|Q8K0B7|Q8K0B7_MOUSE	Solute carrier family 25, member 32 OS=Mus musculus OX=10090 GN=Slc25a32 PE=2 SV=1	MOUSE	2
3115	1.26	1.66	1.93	1.29	sp|Q62415|ASPP1_MOUSE	Apoptosis-stimulating of p53 protein 1 OS=Mus musculus OX=10090 GN=Ppp1r13b PE=1 SV=2	MOUSE	1
3116	1.25	1.36	7.30	1.41	sp|P55012|S12A2_MOUSE	Solute carrier family 12 member 2 OS=Mus musculus OX=10090 GN=Slc12a2 PE=1 SV=2	MOUSE	1
3117	1.25	1.34	12.77	3.74	tr|G3X9A7|G3X9A7_MOUSE	PI-PLC X domain-containing protein 3 OS=Mus musculus OX=10090 GN=Plcxd3 PE=1 SV=1	MOUSE	1
3118	1.25	1.33	15.00	2.50	tr|Q3TZ63|Q3TZ63_MOUSE	Uncharacterized protein OS=Mus musculus OX=10090 GN=Tbc1d13 PE=1 SV=1	MOUSE	1
3119	1.25	1.33	1.92	1.92	tr|A0JLY3|A0JLY3_MOUSE	Christmas factor (Fragment) OS=Mus musculus OX=10090 GN=F9 PE=2 SV=1	MOUSE	1
3120	1.24	1.41	9.86	1.59	tr|A0A2R8VK76|A0A2R8VK76_MOUSE	Cleavage and polyadenylation-specificity factor subunit 1 OS=Mus musculus OX=10090 GN=Cpsf1 PE=1 SV=1	MOUSE	1
3121	1.24	1.38	11.67	2.38	sp|Q8BHS3|RBM22_MOUSE	Pre-mRNA-splicing factor RBM22 OS=Mus musculus OX=10090 GN=Rbm22 PE=1 SV=1	MOUSE	1
3122	1.23	1.36	7.91	1.78	tr|E9PX94|E9PX94_MOUSE	Coiled-coil and C2 domain-containing protein 1A OS=Mus musculus OX=10090 GN=Cc2d1a PE=1 SV=2	MOUSE	1
3123	1.23	1.35	8.00	2.00	sp|Q8BVU5|NUDT9_MOUSE	ADP-ribose pyrophosphatase, mitochondrial OS=Mus musculus OX=10090 GN=Nudt9 PE=1 SV=1	MOUSE	1
3124	1.23	1.33	7.92	2.58	tr|Q9DCC1|Q9DCC1_MOUSE	mRNA-capping enzyme OS=Mus musculus OX=10090 GN=Rngtt PE=1 SV=1	MOUSE	1
3125	1.23	1.33	21.88	6.25	tr|H3BK03|H3BK03_MOUSE	Paraoxonase (Fragment) OS=Mus musculus OX=10090 GN=Pon1 PE=1 SV=9	MOUSE	2
3126	1.23	1.33	10.90	2.93	sp|Q61048|WBP4_MOUSE	WW domain-binding protein 4 OS=Mus musculus OX=10090 GN=Wbp4 PE=1 SV=4	MOUSE	1
3127	1.22	2.88	6.36	2.59	tr|Q99KA8|Q99KA8_MOUSE	Receptor protein-tyrosine kinase OS=Mus musculus OX=10090 GN=Epha4 PE=2 SV=1	MOUSE	2
3128	1.22	1.35	5.96	0.88	tr|A0A0R4J2D3|A0A0R4J2D3_MOUSE	Transport and Golgi organization protein 1 homolog OS=Mus musculus OX=10090 GN=Mia3 PE=1 SV=3	MOUSE	1
3129	1.21	1.33	9.28	1.38	tr|H3BKN0|H3BKN0_MOUSE	NOL1/NOP2/Sun domain family member 2 OS=Mus musculus OX=10090 GN=Nsun2 PE=1 SV=1	MOUSE	1

3130	1.21	1.3	23.44	4.79	tr|Q9CXU4|Q9CXU4_MOUSE	Mitochondrial import inner membrane translocase subunit TIM23 OS=Mus musculus OX=10090 GN=Timm23 PE=1 SV=1	MOUSE	1
3131	1.21	1.29	20.00	11.00	tr|Q3U1A4|Q3U1A4_MOUSE	Uncharacterized protein (Fragment) OS=Mus musculus OX=10090 GN=Rabif PE=2 SV=1	MOUSE	1
3132	1.2	1.54	6.51	2.26	tr|Q3U3S0|Q3U3S0_MOUSE	WD_REPEATS_REGION domain-containing protein OS=Mus musculus OX=10090 GN=Strn4 PE=2 SV=1	MOUSE	1
3133	1.2	1.29	6.69	2.02	sp|Q8CH02|SUGP1_MOUSE	SURP and G-patch domain-containing protein 1 OS=Mus musculus OX=10090 GN=Sugp1 PE=1 SV=1	MOUSE	1
3134	1.2	1.28	7.04	4.22	tr|D3Z1F7|D3Z1F7_MOUSE	Putative monooxygenase p33MONOX OS=Mus musculus OX=10090 GN=4833439L19Rik PE=1 SV=1	MOUSE	1
3135	1.2	1.28	4.04	1.49	RRRRRtr|A2BFG8|A2BFG8_MOUSE	REVERSED Zinc finger protein 971 OS=Mus musculus OX=10090 GN=Zfp971 PE=1 SV=1	MOUSE	1
3136	1.19	1.29	20.87	11.30	tr|Z4YKM2|Z4YKM2_MOUSE	CDGSH iron-sulfur domain-containing protein 3, mitochondrial OS=Mus musculus OX=10090 GN=Cisd3 PE=1 SV=1	MOUSE	1
3137	1.19	1.28	10.34	10.34	tr|D3Z198|D3Z198_MOUSE	28S ribosomal protein S17, mitochondrial (Fragment) OS=Mus musculus OX=10090 GN=Mrps17 PE=1 SV=1	MOUSE	1
3138	1.19	1.27	9.61	5.65	tr|Q3THH3|Q3THH3_MOUSE	MRP-S23 domain-containing protein OS=Mus musculus OX=10090 GN=Mrps23 PE=2 SV=1	MOUSE	1
3139	1.18	1.29	23.53	19.61	tr|Q505A8|Q505A8_MOUSE	Ribosomal protein L39 OS=Mus musculus OX=10090 GN=Rpl39 PE=1 SV=1	MOUSE	1
3140	1.18	1.27	1.87	0.71	tr|Q0VF51|Q0VF51_MOUSE	Regulating synaptic membrane exocytosis protein 2 OS=Mus musculus OX=10090 GN=Rims2 PE=1 SV=1	MOUSE	1
3141	1.17	1.26	7.67	2.36	tr|D3YVU0|D3YVU0_MOUSE	Ubiquitin carboxyl-terminal hydrolase 46 OS=Mus musculus OX=10090 GN=Usp46 PE=1 SV=1	MOUSE	1
3142	1.17	1.25	11.27	8.45	tr|F6ZXW2|F6ZXW2_MOUSE	Family with sequence similarity 98, member C (Fragment) OS=Mus musculus OX=10090 GN=Fam98c PE=1 SV=1	MOUSE	1
3143	1.17	1.25	14.04	14.04	tr|H7BWZ1|H7BWZ1_MOUSE	Ubiquitin-fold modifier 1 OS=Mus musculus OX=10090 GN=Ufm1 PE=1 SV=1	MOUSE	1
3144	1.17	1.24	4.11	4.11	tr|Q8VI86|Q8VI86_MOUSE	COMM domain-containing protein 1 OS=Mus musculus OX=10090 GN=Commd1 PE=1 SV=1	MOUSE	1

3145	1.16	4.65	9.79	8.01	tr|Q6DFY2|Q6DFY2_MOUSE	Opioid-binding protein/cell adhesion molecule-like OS=Mus musculus OX=10090 GN=Opcml PE=1 SV=1	MOUSE	3
3146	1.16	1.28	5.95	2.73	tr|Q3UV20|Q3UV20_MOUSE	MAM domain-containing glycosylphosphatidylinositol anchor protein 2 OS=Mus musculus OX=10090 GN=Mdga2 PE=1 SV=1	MOUSE	2
3147	1.16	1.25	17.24	9.85	tr|Q2NL54|Q2NL54_MOUSE	Uncharacterized protein OS=Mus musculus OX=10090 GN=Mrgbp PE=2 SV=1	MOUSE	2
3148	1.16	1.25	11.29	2.82	tr|F8WI12|F8WI12_MOUSE	Upstream stimulatory factor 2 OS=Mus musculus OX=10090 GN=Usf2 PE=1 SV=1	MOUSE	1
3149	1.16	1.25	5.00	2.61	tr|A0A1W2P7K6|A0A1W2P7K6_MOUSE	Rab-3A-interacting protein OS=Mus musculus OX=10090 GN=Rab3ip PE=1 SV=1	MOUSE	1
3150	1.15	1.27	7.52	7.52	tr|Q0VAW6|Q0VAW6_MOUSE	RIKEN cDNA 8030462N17 gene OS=Mus musculus OX=10090 GN=8030462N17Rik PE=1 SV=1	MOUSE	2
3151	1.15	1.23	1.65	0.87	tr|Q8C4G3|Q8C4G3_MOUSE	Phospholipid-transporting ATPase OS=Mus musculus OX=10090 GN=Atp9a PE=1 SV=1	MOUSE	1
3152	1.14	3.5	26.25	17.08	tr|Q3TYL7|Q3TYL7_MOUSE	DnaJ homolog subfamily B member 1 OS=Mus musculus OX=10090 GN=Dnajb1 PE=1 SV=1	MOUSE	4
3153	1.14	1.35	8.05	3.17	sp|Q9CW79|GOGA1_MOUSE	Golgin subfamily A member 1 OS=Mus musculus OX=10090 GN=Golga1 PE=1 SV=2	MOUSE	2
3154	1.14	1.23	10.21	2.70	tr|Q3TMC4|Q3TMC4_MOUSE	Solute carrier family 25 member 44 OS=Mus musculus OX=10090 GN=Slc25a44 PE=1 SV=1	MOUSE	1
3155	1.13	1.3	17.43	1.95	tr|G3UXM5|G3UXM5_MOUSE	Serine/threonine-protein kinase BRSK2 (Fragment) OS=Mus musculus OX=10090 GN=Brsk2 PE=1 SV=1	MOUSE	1
3156	1.13	1.27	4.85	0.87	tr|F8VQJ3|F8VQJ3_MOUSE	Laminin subunit gamma-1 OS=Mus musculus OX=10090 GN=Lamc1 PE=1 SV=1	MOUSE	1
3157	1.13	1.27	38.18	30.00	tr|E0CY88|E0CY88_MOUSE	Cytochrome b5 OS=Mus musculus OX=10090 GN=Cyb5a PE=1 SV=1	MOUSE	2
3158	1.13	1.23	4.85	1.25	tr|F6T836|F6T836_MOUSE	Rho GTPase-activating protein 26 OS=Mus musculus OX=10090 GN=Arhgap26 PE=1 SV=1	MOUSE	1
3159	1.13	1.21	6.22	3.53	sp|Q921Q3|ALG1_MOUSE	Chitobiosyldiphosphodolichol beta-mannosyltransferase OS=Mus musculus OX=10090 GN=Alg1 PE=1 SV=3	MOUSE	1
3160	1.12	1.24	12.46	6.67	tr|Q9D7A3|Q9D7A3_MOUSE	Uncharacterized protein OS=Mus musculus OX=10090 GN=Rnf113a2 PE=2 SV=1	MOUSE	2

3161	1.12	1.23	7.62	1.45	tr|A0A0A6YXM5|A0A0A6YXM5_MOUSE	REST corepressor 3 OS=Mus musculus OX=10090 GN=Rcor3 PE=1 SV=1	MOUSE	1
3162	1.12	1.23	9.72	4.17	sp|Q99KR3|LACB2_MOUSE	Endoribonuclease LACTB2 OS=Mus musculus OX=10090 GN=Lactb2 PE=1 SV=1	MOUSE	1
3163	1.12	1.2	36.14	12.05	sp|P48771|CX7A2_MOUSE	Cytochrome c oxidase subunit 7A2, mitochondrial OS=Mus musculus OX=10090 GN=Cox7a2 PE=1 SV=2	MOUSE	1
3164	1.12	1.2	3.18	3.18	sp|Q9D6J3|YJU2_MOUSE	Splicing factor YJU2 OS=Mus musculus OX=10090 GN=Yju2 PE=1 SV=1	MOUSE	1
3165	1.11	3.28	19.73	19.73	tr|Q3UFQ4|Q3UFQ4_MOUSE	UBC core domain-containing protein OS=Mus musculus OX=10090 GN=Ube2d1 PE=2 SV=1	MOUSE	5
3166	1.11	1.22	22.25	6.32	tr|Q8K2D8|Q8K2D8_MOUSE	Acidic fibroblast growth factor intracellular-binding protein OS=Mus musculus OX=10090 GN=Fibp PE=1 SV=1	MOUSE	1
3167	1.11	1.2	16.50	4.85	sp|Q8BVG8|NAT14_MOUSE	N-acetyltransferase 14 OS=Mus musculus OX=10090 GN=Nat14 PE=1 SV=1	MOUSE	1
3168	1.11	1.19	18.31	7.75	sp|P83877|TXN4A_MOUSE	Thioredoxin-like protein 4A OS=Mus musculus OX=10090 GN=Txnl4a PE=1 SV=1	MOUSE	1
3169	1.1	1.29	12.19	1.82	tr|Q80V79|Q80V79_MOUSE	DNA mismatch repair protein OS=Mus musculus OX=10090 GN=Msh2 PE=2 SV=1	MOUSE	2
3170	1.1	1.19	15.16	3.55	sp|B1AVZ0|UPP_MOUSE	Uracil phosphoribosyltransferase homolog OS=Mus musculus OX=10090 GN=Uprt PE=1 SV=1	MOUSE	1
3171	1.1	1.18	6.06	6.06	sp|Q9CPS6|HINT3_MOUSE	Histidine triad nucleotide-binding protein 3 OS=Mus musculus OX=10090 GN=Hint3 PE=1 SV=1	MOUSE	1
3172	1.1	1.17	5.53	5.53	sp|P70425|RIT2_MOUSE	GTP-binding protein Rit2 OS=Mus musculus OX=10090 GN=Rit2 PE=1 SV=1	MOUSE	1
3173	1.09	1.19	1.74	0.45	sp|Q9Z2U2-2|ZN292_MOUSE	Isoform 2 of Zinc finger protein 292 OS=Mus musculus OX=10090 GN=Zfp292	MOUSE	1
3174	1.09	1.18	1.49	0.75	sp|Q6P1E1|ZMIZ1_MOUSE	Zinc finger MIZ domain-containing protein 1 OS=Mus musculus OX=10090 GN=Zmiz1 PE=1 SV=1	MOUSE	1
3175	1.09	1.17	15.76	4.30	sp|Q60866|PTER_MOUSE	Phosphotriesterase-related protein OS=Mus musculus OX=10090 GN=Pter PE=1 SV=1	MOUSE	1
3176	1.09	1.17	3.15	1.49	tr|F7B0S9|F7B0S9_MOUSE	Sodium/potassium/calcium exchanger 4 (Fragment) OS=Mus musculus OX=10090 GN=Slc24a4 PE=1 SV=1	MOUSE	1

3177	1.09	1.17	5.38	2.02	tr|E9QKU9|E9QKU9_MOUSE	Tetratricopeptide repeat protein 23 OS=Mus musculus OX=10090 GN=Ttc23 PE=4 SV=1	MOUSE	1
3178	1.09	1.17	8.56	4.96	tr|E9Q2S9|E9Q2S9_MOUSE	Coiled-coil domain-containing protein 43 OS=Mus musculus OX=10090 GN=Ccdc43 PE=1 SV=1	MOUSE	1
3179	1.09	1.17	4.72	4.72	tr|A0A087WRY3|A0A087WRY3_MOUSE	Nuclear ubiquitous casein and cyclin-dependent kinase substrate 1 OS=Mus musculus OX=10090 GN=Nucks1 PE=1 SV=1	MOUSE	1
3180	1.08	1.2	13.48	2.14	sp|Q8R1F1|NIBA2_MOUSE	Protein Niban 2 OS=Mus musculus OX=10090 GN=Niban2 PE=1 SV=2	MOUSE	1
3181	1.07	4.88	7.93	4.06	tr|Q6NXV2|Q6NXV2_MOUSE	Solute carrier family 12, member 4 OS=Mus musculus OX=10090 GN=Slc12a4 PE=2 SV=1	MOUSE	5
3182	1.07	2.44	8.99	4.27	tr|A1KR23|A1KR23_MOUSE	Glycine receptor beta subunit OS=Mus musculus OX=10090 GN=Glrb PE=1 SV=1	MOUSE	2
3183	1.07	1.35	10.83	4.72	tr|Q91YQ8|Q91YQ8_MOUSE	Amacr protein OS=Mus musculus OX=10090 GN=Amacr PE=2 SV=1	MOUSE	1
3184	1.07	1.2	3.10	1.13	tr|E9QPX1|E9QPX1_MOUSE	Collagen alpha-1(XVIII) chain OS=Mus musculus OX=10090 GN=Col18a1 PE=1 SV=1	MOUSE	2
3185	1.07	1.15	16.20	4.22	tr|F6SQH7|F6SQH7_MOUSE	Polymerase delta-interacting protein 2 (Fragment) OS=Mus musculus OX=10090 GN=Poldip2 PE=1 SV=1	MOUSE	1
3186	1.07	1.14	25.00	25.00	tr|C3VDI2|C3VDI2_MOUSE	Etoposide induced 2.4 protein (Fragment) OS=Mus musculus OX=10090 GN=Ei24 PE=4 SV=1	MOUSE	1
3187	1.06	1.14	3.50	1.65	tr|A0A1S6GWK5|A0A1S6GWK5_MOUSE	GCFC domain-containing protein OS=Mus musculus OX=10090 GN=Paxbp1 PE=2 SV=1	MOUSE	1
3188	1.05	1.15	17.99	4.50	sp|Q9JK23|PSMG1_MOUSE	Proteasome assembly chaperone 1 OS=Mus musculus OX=10090 GN=Psmg1 PE=1 SV=1	MOUSE	1
3189	1.05	1.14	6.18	2.13	sp|D3YZP9|CCDC6_MOUSE	Coiled-coil domain-containing protein 6 OS=Mus musculus OX=10090 GN=Ccdc6 PE=1 SV=1	MOUSE	1
3190	1.05	1.13	21.98	8.62	sp|Q922M7|ASHWN_MOUSE	Ashwin OS=Mus musculus OX=10090 PE=1 SV=1	MOUSE	1
3191	1.04	1.15	11.81	2.24	sp|Q9JM13|RABX5_MOUSE	Rab5 GDP/GTP exchange factor OS=Mus musculus OX=10090 GN=Rabgef1 PE=1 SV=1	MOUSE	1
3192	1.04	1.13	13.20	9.14	sp|Q9JJV1|CRBA2_MOUSE	Beta-crystallin A2 OS=Mus musculus OX=10090 GN=Cryba2 PE=2 SV=3	MOUSE	1

3193	1.04	1.12	21.74	13.04	sp|Q9DAM7|TM263_MOUSE	Transmembrane protein 263 OS=Mus musculus OX=10090 GN=Tmem263 PE=1 SV=1	MOUSE	1
3194	1.03	21.36	69.86	69.52	sp|Q9JKB3-2|YBOX3_MOUSE	Isoform 2 of Y-box-binding protein 3 OS=Mus musculus OX=10090 GN=Ybx3	MOUSE	12
3195	1.03	1.22	10.11	5.32	tr|Q9QWX2|Q9QWX2_MOUSE	Apoptosis regulator Bcl-X (Fragment) OS=Mus musculus OX=10090 GN=Bcl2l1 PE=1 SV=1	MOUSE	1
3196	1.03	1.19	7.46	2.62	tr|Q3UGC8|Q3UGC8_MOUSE	Propanoyl-CoA:carbon dioxide ligase subunit alpha OS=Mus musculus OX=10090 GN=Pcca PE=1 SV=1	MOUSE	2
3197	1.03	1.12	8.57	1.32	sp|Q8R2M2|TDIF2_MOUSE	Deoxynucleotidyltransferase terminal-interacting protein 2 OS=Mus musculus OX=10090 GN=Dnttip2 PE=1 SV=1	MOUSE	1
3198	1.03	1.11	8.09	2.76	tr|A2AEM2|A2AEM2_MOUSE	Chloride channel CLIC-like protein 1 OS=Mus musculus OX=10090 GN=Clcc1 PE=1 SV=1	MOUSE	1
3199	1.03	1.11	7.23	5.70	sp|O70494-3|SP3_MOUSE	Isoform 3 of Transcription factor Sp3 OS=Mus musculus OX=10090 GN=Sp3	MOUSE	2
3200	1.03	1.1	2.99	2.99	tr|Q0H9Q2|Q0H9Q2_MOUSE	Transcription factor Pur-1 (Fragment) OS=Mus musculus OX=10090 GN=Maz PE=2 SV=1	MOUSE	1
3201	1.02	1.14	5.78	2.20	tr|Q3UFC2|Q3UFC2_MOUSE	Anaphase-promoting complex subunit 5 OS=Mus musculus OX=10090 GN=Anapc5 PE=1 SV=1	MOUSE	1
3202	1.02	1.12	6.73	4.17	sp|Q9EQC4|ELOV4_MOUSE	Elongation of very long chain fatty acids protein 4 OS=Mus musculus OX=10090 GN=Elovl4 PE=1 SV=2	MOUSE	2
3203	1.02	1.1	2.78	1.46	tr|Q8C0W5|Q8C0W5_MOUSE	RNB domain-containing protein OS=Mus musculus OX=10090 GN=Dis3l2 PE=2 SV=1	MOUSE	1
3204	1.02	1.1	1.95	1.95	sp|Q5XJE5|LEO1_MOUSE	RNA polymerase-associated protein LEO1 OS=Mus musculus OX=10090 GN=Leo1 PE=1 SV=2	MOUSE	2
3205	1.01	1.15	12.30	4.85	tr|S4R2D5|S4R2D5_MOUSE	Ubiquitin-like modifier-activating enzyme ATG7 OS=Mus musculus OX=10090 GN=Atg7 PE=1 SV=1	MOUSE	2
3206	1.01	1.14	3.80	2.17	tr|A0A2X0SZ45|A0A2X0SZ45_MOUSE	SRGAP2 (Fragment) OS=Mus musculus OX=10090 GN=SRGAP2 PE=4 SV=1	MOUSE	2
3207	1.01	1.14	6.50	2.07	tr|D3YY38|D3YY38_MOUSE	Probable cationic amino acid transporter OS=Mus musculus OX=10090 GN=Slc7a14 PE=1 SV=1	MOUSE	1
3208	1.01	1.12	4.04	0.82	tr|F6U6U5|F6U6U5_MOUSE	Serine/threonine-protein kinase SIK3 (Fragment) OS=Mus musculus OX=10090 GN=Sik3 PE=1 SV=1	MOUSE	1
3209	1.01	1.12	6.61	2.77	tr|D3YUP1|D3YUP1_MOUSE	Coactivator-associated arginine methyltransferase 1 OS=Mus musculus OX=10090 GN=Carm1 PE=1 SV=1	MOUSE	1

3210	1.01	1.11	2.12	0.45	tr|A0A140LHX5|A0A140LHX5_MOUSE	Protein unc-13 homolog B OS=Mus musculus OX=10090 GN=Unc13b PE=1 SV=1	MOUSE	1
3211	1.01	1.09	8.17	4.28	sp|Q9CZX7|PP4P2_MOUSE	Type 2 phosphatidylinositol 4,5-bisphosphate 4-phosphatase OS=Mus musculus OX=10090 GN=Pip4p2 PE=1 SV=1	MOUSE	1
3212	1.01	1.09	2.55	2.55	tr|Q3UCZ5|Q3UCZ5_MOUSE	Tyrosine-protein phosphatase non-receptor type OS=Mus musculus OX=10090 GN=Ptpn1 PE=2 SV=1	MOUSE	1
3213	1.01	1.08	24.24	13.13	tr|D3Z7G3|D3Z7G3_MOUSE	Protein FAM241B (Fragment) OS=Mus musculus OX=10090 GN=Fam241b PE=1 SV=1	MOUSE	1
3214	1	1.12	8.68	8.68	sp|Q99K23|UFSP2_MOUSE	Ufm1-specific protease 2 OS=Mus musculus OX=10090 GN=Ufsp2 PE=1 SV=1	MOUSE	2
3215	1	1.09	3.09	1.85	sp|Q80WG5|LRC8A_MOUSE	Volume-regulated anion channel subunit LRRC8A OS=Mus musculus OX=10090 GN=Lrrc8a PE=1 SV=1	MOUSE	1
3216	1	1.08	2.89	2.89	tr|A0A3B2WDB0|A0A3B2WDB0_MOUSE	Vomeronasal 2, receptor 82 OS=Mus musculus OX=10090 GN=Vmn2r82 PE=4 SV=1	MOUSE	1
3217	1	1.08	7.50	7.50	sp|P28667|MRP_MOUSE	MARCKS-related protein OS=Mus musculus OX=10090 GN=Marcksl1 PE=1 SV=2	MOUSE	1
3218	0.99	1.29	10.91	2.00	tr|Q3TUQ7|Q3TUQ7_MOUSE	Acetyl-CoA carboxylase kinase OS=Mus musculus OX=10090 GN=Prkaa1 PE=1 SV=1	MOUSE	1
3219	0.99	1.14	6.60	0.75	sp|Q5SFM8|RBM27_MOUSE	RNA-binding protein 27 OS=Mus musculus OX=10090 GN=Rbm27 PE=1 SV=3	MOUSE	1
3220	0.99	1.1	10.90	2.32	tr|Q8BPN4|Q8BPN4_MOUSE	AP-2 transcription factor OS=Mus musculus OX=10090 GN=Tfap2a PE=1 SV=1	MOUSE	1
3221	0.99	1.07	10.22	2.68	tr|Q08EA5|Q08EA5_MOUSE	Serine (Or cysteine) peptidase inhibitor, clade A, member 1F OS=Mus musculus OX=10090 GN=Serpina1f PE=2 SV=1	MOUSE	1
3222	0.99	1.07	20.16	8.07	tr|A0A1W2P7P4|A0A1W2P7P4_MOUSE	Protein max OS=Mus musculus OX=10090 GN=Max PE=1 SV=1	MOUSE	1
3223	0.99	1.07	4.42	4.42	tr|Q8BT06|Q8BT06_MOUSE	Tetraspanin OS=Mus musculus OX=10090 GN=Cd63 PE=2 SV=1	MOUSE	1
3224	0.98	1.11	13.94	5.45	tr|Q3TWR3|Q3TWR3_MOUSE	5'-AMP-activated protein kinase subunit gamma-1 OS=Mus musculus OX=10090 GN=Prkag1 PE=1 SV=1	MOUSE	1

3225	0.98	1.07	6.84	2.04	tr|Q059U9|Q059U9_MOUSE	A kinase (PRKA) anchor protein 8 OS=Mus musculus OX=10090 GN=Akap8 PE=1 SV=1	MOUSE	1
3226	0.98	1.07	3.85	1.10	sp|Q80ZI6|LRSM1_MOUSE	E3 ubiquitin-protein ligase LRSAM1 OS=Mus musculus OX=10090 GN=Lrsam1 PE=1 SV=1	MOUSE	1
3227	0.98	1.06	4.36	2.82	tr|A6YR19|A6YR19_MOUSE	Phosphodiesterase OS=Mus musculus OX=10090 GN=Pde8b PE=2 SV=1	MOUSE	4
3228	0.97	1.05	7.66	4.79	tr|A0A338P7L8|A0A338P7L8_MOUSE	Charged multivesicular body protein 2b OS=Mus musculus OX=10090 GN=Chmp2b PE=1 SV=1	MOUSE	1
3229	0.97	1.05	26.60	26.60	sp|Q9D164|FXYD6_MOUSE	FXYD domain-containing ion transport regulator 6 OS=Mus musculus OX=10090 GN=Fxyd6 PE=1 SV=2	MOUSE	1
3230	0.97	1.05	5.98	5.98	sp|Q9D0J4|ARL2_MOUSE	ADP-ribosylation factor-like protein 2 OS=Mus musculus OX=10090 GN=Arl2 PE=1 SV=1	MOUSE	1
3231	0.96	1.09	7.74	2.15	tr|Q3UI47|Q3UI47_MOUSE	Kinesin-like protein OS=Mus musculus OX=10090 GN=Kif3a PE=1 SV=1	MOUSE	1
3232	0.96	1.06	4.32	1.12	sp|Q80UF4|SDCG8_MOUSE	Serologically defined colon cancer antigen 8 homolog OS=Mus musculus OX=10090 GN=Sdccag8 PE=1 SV=1	MOUSE	1
3233	0.96	1.05	10.06	4.09	sp|Q9EPL4|METL9_MOUSE	Protein-L-histidine N-pros-methyltransferase OS=Mus musculus OX=10090 GN=Mettl9 PE=1 SV=1	MOUSE	1
3234	0.96	1.05	5.82	3.70	tr|Q8C4C9|Q8C4C9_MOUSE	DnaJ homolog subfamily B member 12 OS=Mus musculus OX=10090 GN=Dnajb12 PE=1 SV=1	MOUSE	1
3235	0.96	1.04	7.20	2.73	tr|A2RSW6|A2RSW6_MOUSE	Aminomethyltransferase OS=Mus musculus OX=10090 GN=Amt PE=1 SV=1	MOUSE	1
3236	0.95	1.04	7.13	2.20	sp|Q9QYE6|GOGA5_MOUSE	Golgin subfamily A member 5 OS=Mus musculus OX=10090 GN=Golga5 PE=1 SV=2	MOUSE	1
3237	0.95	1.04	17.78	5.56	sp|Q8VE99|CC115_MOUSE	Coiled-coil domain-containing protein 115 OS=Mus musculus OX=10090 GN=Ccdc115 PE=1 SV=1	MOUSE	1
3238	0.95	1.04	4.78	3.34	sp|O35668|HAP1_MOUSE	Huntingtin-associated protein 1 OS=Mus musculus OX=10090 GN=Hap1 PE=1 SV=1	MOUSE	1
3239	0.95	1.02	18.33	18.33	tr|Q80VN1|Q80VN1_MOUSE	1110007C09Rik protein OS=Mus musculus OX=10090 GN=Card19 PE=2 SV=1	MOUSE	1
3240	0.94	3.12	4.50	1.57	tr|Q3UH70|Q3UH70_MOUSE	Bromodomain-containing protein 4 OS=Mus musculus OX=10090 GN=Brd4 PE=1 SV=1	MOUSE	2
3241	0.94	1.83	7.82	2.56	tr|E9QNX9|E9QNX9_MOUSE	Tyrosine-protein kinase receptor OS=Mus musculus OX=10090 GN=Igf1r PE=1 SV=1	MOUSE	3

3242	0.94	1.28	72.00	40.00	sp|P63216|GBG3_MOUSE	Guanine nucleotide-binding protein G(I)/G(S)/G(O) subunit gamma-3 OS=Mus musculus OX=10090 GN=Gng3 PE=1 SV=1	MOUSE	1
3243	0.94	1.07	8.09	1.51	tr|Q3TMH3|Q3TMH3_MOUSE	WD_REPEATS_REGION domain-containing protein OS=Mus musculus OX=10090 GN=Wdr6 PE=2 SV=1	MOUSE	2
3244	0.94	1.06	9.38	1.91	tr|D3YZC2|D3YZC2_MOUSE	Menin OS=Mus musculus OX=10090 GN=Men1 PE=1 SV=1	MOUSE	1
3245	0.94	1.02	5.28	1.96	sp|B2RQA1|KCA10_MOUSE	Potassium voltage-gated channel subfamily A member 10 OS=Mus musculus OX=10090 GN=Kcna10 PE=2 SV=1	MOUSE	1
3246	0.94	1.02	6.31	3.30	tr|Q3TV62|Q3TV62_MOUSE	Torsin OS=Mus musculus OX=10090 GN=Tor1a PE=1 SV=1	MOUSE	1
3247	0.94	1.02	22.22	11.97	sp|Q9D7J4|COX20_MOUSE	Cytochrome c oxidase assembly protein COX20, mitochondrial OS=Mus musculus OX=10090 GN=Cox20 PE=1 SV=1	MOUSE	1
3248	0.94	1.02	5.18	5.18	tr|H3BLH2|H3BLH2_MOUSE	Sulfide:quinone oxidoreductase, mitochondrial (Fragment) OS=Mus musculus OX=10090 GN=Sqor PE=1 SV=1	MOUSE	1
3249	0.93	1.06	4.76	1.68	sp|Q9ESJ4|SPN90_MOUSE	NCK-interacting protein with SH3 domain OS=Mus musculus OX=10090 GN=Nckipsd PE=1 SV=2	MOUSE	1
3250	0.93	1.03	9.97	4.98	sp|Q8BGU5|CCNY_MOUSE	Cyclin-Y OS=Mus musculus OX=10090 GN=Ccny PE=1 SV=1	MOUSE	1
3251	0.93	1.01	5.82	3.27	tr|B9EJ14|B9EJ14_MOUSE	Voltage-dependent calcium channel gamma-7 subunit OS=Mus musculus OX=10090 GN=Cacng7 PE=1 SV=1	MOUSE	1
3252	0.92	1.04	6.45	0.63	sp|Q9JJ28|FLII_MOUSE	Protein flightless-1 homolog OS=Mus musculus OX=10090 GN=Flii PE=1 SV=1	MOUSE	2
3253	0.92	1.01	12.14	8.09	tr|B9EKL6|B9EKL6_MOUSE	Protein tyrosine phosphatase 4a1 OS=Mus musculus OX=10090 GN=Ptp4a1 PE=1 SV=1	MOUSE	1
3254	0.92	1	5.13	5.13	sp|Q9D531|NXNL2_MOUSE	Nucleoredoxin-like protein 2 OS=Mus musculus OX=10090 GN=Nxnl2 PE=1 SV=1	MOUSE	1
3255	0.92	1	4.27	4.27	sp|P70460|VASP_MOUSE	Vasodilator-stimulated phosphoprotein OS=Mus musculus OX=10090 GN=Vasp PE=1 SV=4	MOUSE	1
3256	0.91	2.5	4.28	0.88	tr|Q5SWZ5|Q5SWZ5_MOUSE	Myosin phosphatase Rho-interacting protein OS=Mus musculus OX=10090 GN=Mprip PE=1 SV=1	MOUSE	2

3257	0.91	1.41	11.34	5.09	tr|Q8R2U8|Q8R2U8_MOUSE	Protein-serine/threonine kinase OS=Mus musculus OX=10090 GN=Pdk1 PE=2 SV=1	MOUSE	2
3258	0.91	1.03	3.61	2.29	sp|Q6NVE8|WDR44_MOUSE	WD repeat-containing protein 44 OS=Mus musculus OX=10090 GN=Wdr44 PE=1 SV=1	MOUSE	2
3259	0.91	1	7.98	1.95	sp|Q4VBD2|TAPT1_MOUSE	Transmembrane anterior posterior transformation protein 1 OS=Mus musculus OX=10090 GN=Tapt1 PE=1 SV=2	MOUSE	1
3260	0.91	0.99	4.60	1.59	tr|A0A5H1ZRL3|A0A5H1ZRL3_MOUSE	Negative elongation factor B OS=Mus musculus OX=10090 GN=Nelfb PE=1 SV=1	MOUSE	1
3261	0.91	0.99	15.17	9.66	sp|Q8VHC3|SELM_MOUSE	Selenoprotein M OS=Mus musculus OX=10090 GN=Selenom PE=1 SV=3	MOUSE	1
3262	0.91	0.99	2.59	1.38	RRRRRsp|P98083|SHC1_MOUSE	REVERSED SHC-transforming protein 1 OS=Mus musculus OX=10090 GN=Shc1 PE=1 SV=3	MOUSE	1
3263	0.91	0.98	6.90	6.90	tr|Q05BF8|Q05BF8_MOUSE	Pggt1b protein OS=Mus musculus OX=10090 GN=Pggt1b PE=2 SV=1	MOUSE	1
3264	0.91	0.98	0.55	0.55	RRRRRsp|Q5SWW4|MED13_MOUSE	REVERSED Mediator of RNA polymerase II transcription subunit 13 OS=Mus musculus OX=10090 GN=Med13 PE=1 SV=1	MOUSE	1
3265	0.9	42.28	49.83	49.48	tr|G5E8G0|G5E8G0_MOUSE	Heterogeneous nuclear ribonucleoprotein D0 OS=Mus musculus OX=10090 GN=Hnrnpd PE=1 SV=1	MOUSE	22
3266	0.9	1.07	7.42	1.85	tr|Q5SW15|Q5SW15_MOUSE	U6 small nuclear RNA (adenine-(43)-N(6))-methyltransferase OS=Mus musculus OX=10090 GN=Mettl16 PE=1 SV=1	MOUSE	1
3267	0.9	0.99	22.15	8.30	sp|Q3TQI7|TLS1_MOUSE	Telomere length and silencing protein 1 homolog OS=Mus musculus OX=10090 PE=1 SV=2	MOUSE	2
3268	0.9	0.99	5.13	2.26	tr|Q6A0E5|Q6A0E5_MOUSE	L-serine-phosphatidylethanolamine phosphatidyltransferase (Fragment) OS=Mus musculus OX=10090 GN=mKIAA0024 PE=3 SV=1	MOUSE	1
3269	0.9	0.98	8.75	2.14	tr|Q571M3|Q571M3_MOUSE	MKIAA4018 protein (Fragment) OS=Mus musculus OX=10090 GN=Lrrc1 PE=2 SV=1	MOUSE	1
3270	0.9	0.98	2.42	1.08	sp|Q8VHK1|CSKI2_MOUSE	Caskin-2 OS=Mus musculus OX=10090 GN=Caskin2 PE=1 SV=3	MOUSE	1
3271	0.9	0.98	6.19	6.19	tr|A0A0U1RPM3|A0A0U1RPM3_MOUSE	PRA1 family protein OS=Mus musculus OX=10090 GN=Rabac1 PE=1 SV=1	MOUSE	1

3272	0.88	0.99	9.31	2.00	tr|Q6F4J1|Q6F4J1_MOUSE	Tubulin gamma chain OS=Mus musculus OX=10090 GN=Tubg1 PE=1 SV=1	MOUSE	1
3273	0.87	3.15	11.49	5.17	tr|J3QMQ8|J3QMQ8_MOUSE	Thioredoxin-disulfide reductase OS=Mus musculus OX=10090 GN=Txnrd2 PE=1 SV=1	MOUSE	3
3274	0.87	1.04	10.58	5.04	sp|Q8CHS8|VP37A_MOUSE	Vacuolar protein sorting-associated protein 37A OS=Mus musculus OX=10090 GN=Vps37a PE=1 SV=1	MOUSE	1
3275	0.87	0.99	12.42	2.78	sp|Q6GQS1|SCMC3_MOUSE	Calcium-binding mitochondrial carrier protein SCaMC-3 OS=Mus musculus OX=10090 GN=Slc25a23 PE=1 SV=1	MOUSE	1
3276	0.87	0.98	5.31	1.82	sp|Q8CJ53|CIP4_MOUSE	Cdc42-interacting protein 4 OS=Mus musculus OX=10090 GN=Trip10 PE=1 SV=2	MOUSE	1
3277	0.87	0.95	5.60	2.66	tr|Q9CZI7|Q9CZI7_MOUSE	Annexin OS=Mus musculus OX=10090 GN=Anxa2 PE=2 SV=1	MOUSE	1
3278	0.87	0.95	11.76	6.72	sp|Q9CQY2|RAMAC_MOUSE	RNA guanine-N7 methyltransferase activating subunit OS=Mus musculus OX=10090 GN=Ramac PE=3 SV=1	MOUSE	1
3279	0.87	0.94	1.77	1.77	tr|A2AI62|A2AI62_MOUSE	Hephaestin OS=Mus musculus OX=10090 GN=Heph PE=1 SV=1	MOUSE	1
3280	0.86	1.44	41.94	35.48	tr|Q61782|Q61782_MOUSE	Type I epidermal keratin mRNA, 3'end (Fragment) OS=Mus musculus OX=10090 PE=2 SV=1	MOUSE	2
3281	0.86	1.08	18.97	5.17	tr|E0CYS1|E0CYS1_MOUSE	Glutamate receptor-interacting protein 2 OS=Mus musculus OX=10090 GN=Grip2 PE=1 SV=1	MOUSE	1
3282	0.86	0.97	6.72	1.37	tr|Q3TAC0|Q3TAC0_MOUSE	BCAS3 domain-containing protein (Fragment) OS=Mus musculus OX=10090 GN=Bcas3 PE=2 SV=1	MOUSE	1
3283	0.85	1.09	5.00	5.00	tr|F6ZE40|F6ZE40_MOUSE	Ubiquinone biosynthesis monooxygenase COQ6, mitochondrial (Fragment) OS=Mus musculus OX=10090 GN=Coq6 PE=1 SV=1	MOUSE	1
3284	0.85	0.97	4.79	1.18	tr|A1L340|A1L340_MOUSE	Retinitis pigmentosa 1 homolog (Human)-like 1 OS=Mus musculus OX=10090 GN=Rp1l1 PE=2 SV=1	MOUSE	1
3285	0.85	0.94	11.47	1.89	tr|Q8K2V2|Q8K2V2_MOUSE	Tmcc1 protein (Fragment) OS=Mus musculus OX=10090 GN=Tmcc1 PE=2 SV=1	MOUSE	1
3286	0.84	0.98	14.26	2.06	tr|F6TVP2|F6TVP2_MOUSE	Deoxynucleoside triphosphate triphosphohydrolase SAMHD1 (Fragment) OS=Mus musculus OX=10090 GN=Samhd1 PE=1 SV=1	MOUSE	1
3287	0.84	0.98	11.35	1.89	tr|A0A1D5RLL3|A0A1D5RLL3_MOUSE	Protein EFR3 homolog A OS=Mus musculus OX=10090 GN=Efr3a PE=1 SV=1	MOUSE	1

3288	0.84	0.97	4.41	1.14	tr|A0A140LHU9|A0A140LHU9_MOUSE	Patatin-like phospholipase domain-containing protein 6 OS=Mus musculus OX=10090 GN=Pnpla6 PE=1 SV=1	MOUSE	1
3289	0.84	0.96	15.86	3.23	sp|Q8K0C9|GMDS_MOUSE	GDP-mannose 4,6 dehydratase OS=Mus musculus OX=10090 GN=Gmds PE=1 SV=1	MOUSE	1
3290	0.84	0.94	1.54	0.45	sp|Q6ZQH8|NU188_MOUSE	Nucleoporin NUP188 OS=Mus musculus OX=10090 GN=Nup188 PE=1 SV=2	MOUSE	1
3291	0.84	0.93	6.49	2.64	tr|E9PYH3|E9PYH3_MOUSE	Ethanolamine-phosphate phospho-lyase OS=Mus musculus OX=10090 GN=Etnppl PE=1 SV=1	MOUSE	1
3292	0.84	0.92	8.28	2.61	tr|Q921V3|Q921V3_MOUSE	Cysteine desulfurase, mitochondrial OS=Mus musculus OX=10090 GN=Nfs1 PE=2 SV=1	MOUSE	1
3293	0.83	0.96	9.50	1.61	tr|Q149D1|Q149D1_MOUSE	Threonine synthase-like 1 (Bacterial) OS=Mus musculus OX=10090 GN=Thnsl1 PE=1 SV=1	MOUSE	1
3294	0.83	0.93	8.22	2.35	sp|Q3TIV5|ZC3HF_MOUSE	Zinc finger CCCH domain-containing protein 15 OS=Mus musculus OX=10090 GN=Zc3h15 PE=1 SV=2	MOUSE	1
3295	0.83	0.91	11.22	2.20	sp|Q91V36|NRBP2_MOUSE	Nuclear receptor-binding protein 2 OS=Mus musculus OX=10090 GN=Nrbp2 PE=1 SV=2	MOUSE	1
3296	0.82	2.99	8.01	2.71	tr|A2AS47|A2AS47_MOUSE	Plakophilin-4 OS=Mus musculus OX=10090 GN=Pkp4 PE=1 SV=1	MOUSE	2
3297	0.82	2.99	22.07	15.86	sp|Q9D6K5|SYJ2B_MOUSE	Synaptojanin-2-binding protein OS=Mus musculus OX=10090 GN=Synj2bp PE=1 SV=1	MOUSE	2
3298	0.82	0.95	6.89	1.89	sp|Q6NVF0|OCRL_MOUSE	Inositol polyphosphate 5-phosphatase OCRL OS=Mus musculus OX=10090 GN=Ocrl PE=1 SV=1	MOUSE	1
3299	0.82	0.92	20.27	2.66	sp|Q9D6T0|NOSIP_MOUSE	Nitric oxide synthase-interacting protein OS=Mus musculus OX=10090 GN=Nosip PE=1 SV=1	MOUSE	1
3300	0.82	0.9	10.00	10.00	tr|Q5SXR2|Q5SXR2_MOUSE	Vacuole membrane protein 1 (Fragment) OS=Mus musculus OX=10090 GN=Vmp1 PE=1 SV=1	MOUSE	1
3301	0.81	0.99	4.59	1.08	RRRRRtr|B2RXR1|B2RXR1_MOUSE	REVERSED Coiled-coil domain containing 88B OS=Mus musculus OX=10090 GN=Ccdc88b PE=2 SV=1	MOUSE	1
3302	0.81	0.94	17.34	10.33	tr|V9GX21|V9GX21_MOUSE	ATP synthase mitochondrial F1 complex assembly factor 1 (Fragment) OS=Mus musculus OX=10090 GN=Atpaf1 PE=1 SV=1	MOUSE	3
3303	0.8	5	4.92	4.15	tr|Q3UIM5|Q3UIM5_MOUSE	Carnitine O-palmitoyltransferase OS=Mus musculus OX=10090 GN=Cpt1b PE=2 SV=1	MOUSE	3

3304	0.8	0.93	15.22	10.87	sp|P70245|EBP_MOUSE	3-beta-hydroxysteroid-Delta(8),Delta(7)-isomerase OS=Mus musculus OX=10090 GN=Ebp PE=1 SV=3	MOUSE	2
3305	0.8	0.88	3.06	3.06	RRRRRsp|Q9D5T7|HORM1_MOUSE	REVERSED HORMA domain-containing protein 1 OS=Mus musculus OX=10090 GN=Hormad1 PE=1 SV=1	MOUSE	1
3306	0.79	9.96	26.15	23.73	sp|Q00897|A1AT4_MOUSE	Alpha-1-antitrypsin 1-4 OS=Mus musculus OX=10090 GN=Serpina1d PE=1 SV=1	MOUSE	6
3307	0.79	0.98	2.68	0.67	RRRRRtr|Q921Y1|Q921Y1_MOUSE	REVERSED RNA helicase OS=Mus musculus OX=10090 GN=Dhx16 PE=2 SV=1	MOUSE	1
3308	0.79	0.91	3.56	0.97	tr|E9PUC4|E9PUC4_MOUSE	A-kinase anchor protein SPHKAP OS=Mus musculus OX=10090 GN=Sphkap PE=1 SV=1	MOUSE	1
3309	0.79	0.89	14.67	6.67	tr|Q8C6B5|Q8C6B5_MOUSE	Uncharacterized protein OS=Mus musculus OX=10090 GN=Yy1 PE=2 SV=1	MOUSE	1
3310	0.78	0.91	2.72	0.35	tr|A1ILG8|A1ILG8_MOUSE	Chorein OS=Mus musculus OX=10090 GN=Vps13c PE=2 SV=1	MOUSE	1
3311	0.77	0.88	9.08	1.59	tr|Q3UVI9|Q3UVI9_MOUSE	Uncharacterized protein OS=Mus musculus OX=10090 GN=Abcf2 PE=2 SV=1	MOUSE	1
3312	0.77	0.85	9.23	2.79	sp|Q5RJ54|ZSC26_MOUSE	Zinc finger and SCAN domain-containing protein 26 OS=Mus musculus OX=10090 GN=Zscan26 PE=2 SV=1	MOUSE	1
3313	0.77	0.85	1.42	1.33	tr|A2AMY5|A2AMY5_MOUSE	Ubiquitin-associated protein 2 OS=Mus musculus OX=10090 GN=Ubap2 PE=1 SV=1	MOUSE	1
3314	0.77	0.85	1.62	1.62	tr|O88325|O88325_MOUSE	Alpha-N-acetylglucosaminidase OS=Mus musculus OX=10090 GN=Naglu PE=1 SV=1	MOUSE	1
3315	0.76	0.91	11.32	0.00	tr|B2RQV1|B2RQV1_MOUSE	Uncharacterized protein OS=Mus musculus OX=10090 GN=Naa25 PE=2 SV=1	MOUSE	0
3316	0.76	0.88	7.82	2.61	sp|Q8CI33|C19L1_MOUSE	CWF19-like protein 1 OS=Mus musculus OX=10090 GN=Cwf19l1 PE=1 SV=2	MOUSE	1
3317	0.76	0.88	9.31	6.85	sp|P97792|CXAR_MOUSE	Coxsackievirus and adenovirus receptor homolog OS=Mus musculus OX=10090 GN=Cxadr PE=1 SV=1	MOUSE	2
3318	0.76	0.86	5.28	1.32	tr|G5E8A0|G5E8A0_MOUSE	Oxysterol-binding protein OS=Mus musculus OX=10090 GN=Osbpl11 PE=1 SV=1	MOUSE	1
3319	0.76	0.86	36.67	13.33	tr|G3UZ30|G3UZ30_MOUSE	E3 ubiquitin-protein ligase PPP1R11 (Fragment) OS=Mus musculus OX=10090 GN=Ppp1r11 PE=1 SV=2	MOUSE	1
3320	0.76	0.86	7.47	3.90	sp|Q02614|S30BP_MOUSE	SAP30-binding protein OS=Mus musculus OX=10090 GN=Sap30bp PE=1 SV=2	MOUSE	1

3321	0.76	0.84	6.75	1.33	tr|D6QSS8|D6QSS8_MOUSE	A disintegrin and metallopeptidase domain 22 isoform OS=Mus musculus OX=10090 GN=Adam22 PE=2 SV=1	MOUSE	1
3322	0.76	0.84	4.25	1.65	tr|Q69ZZ7|Q69ZZ7_MOUSE	F-spondin (Fragment) OS=Mus musculus OX=10090 GN=mKIAA0762 PE=4 SV=1	MOUSE	1
3323	0.76	0.84	1.36	1.36	tr|Q7TMQ0|Q7TMQ0_MOUSE	Sodium/nucleoside cotransporter OS=Mus musculus OX=10090 GN=Slc28a2 PE=2 SV=1	MOUSE	1
3324	0.76	0.84	3.79	3.79	RRRRRtr|A0A1D5RLR3|A0A1D5RLR3_MOUSE	REVERSED RNA-binding protein 34 OS=Mus musculus OX=10090 GN=Rbm34 PE=1 SV=1	MOUSE	1
3325	0.76	0.83	3.91	3.91	tr|Q9ES94|Q9ES94_MOUSE	Cathepsin X OS=Mus musculus OX=10090 GN=Ctsz PE=2 SV=1	MOUSE	1
3326	0.75	2.6	17.51	6.09	sp|Q6PAK3|ANM8_MOUSE	Protein arginine N-methyltransferase 8 OS=Mus musculus OX=10090 GN=Prmt8 PE=1 SV=2	MOUSE	2
3327	0.75	0.83	5.56	1.68	tr|A0A0J9YVF0|A0A0J9YVF0_MOUSE	Metabotropic glutamate receptor 2 OS=Mus musculus OX=10090 GN=Grm2 PE=1 SV=1	MOUSE	1
3328	0.75	0.83	4.44	1.03	tr|B7XG49|B7XG49_MOUSE	Family with sequence similarity 71, member A OS=Mus musculus OX=10090 GN=Fam71a PE=1 SV=1	MOUSE	1
3329	0.75	0.82	4.32	2.78	sp|Q9EPR4|S23A2_MOUSE	Solute carrier family 23 member 2 OS=Mus musculus OX=10090 GN=Slc23a2 PE=1 SV=2	MOUSE	1
3330	0.74	2.56	9.94	3.63	sp|Q8K0D0|CDK17_MOUSE	Cyclin-dependent kinase 17 OS=Mus musculus OX=10090 GN=Cdk17 PE=1 SV=2	MOUSE	2
3331	0.74	0.84	9.02	2.65	tr|Q541Z2|Q541Z2_MOUSE	Uncharacterized protein OS=Mus musculus OX=10090 GN=Fnta PE=1 SV=1	MOUSE	1
3332	0.74	0.82	2.39	0.95	tr|Q5DTJ4|Q5DTJ4_MOUSE	MKIAA4178 protein (Fragment) OS=Mus musculus OX=10090 GN=Prrc2a PE=2 SV=1	MOUSE	1
3333	0.74	0.82	34.78	34.78	tr|D3YWR8|D3YWR8_MOUSE	RIKEN cDNA 1110059G10 gene OS=Mus musculus OX=10090 GN=1110059G10Rik PE=1 SV=1	MOUSE	1
3334	0.73	0.99	11.40	2.39	sp|Q8BFR4|GNS_MOUSE	N-acetylglucosamine-6-sulfatase OS=Mus musculus OX=10090 GN=Gns PE=1 SV=1	MOUSE	1
3335	0.73	0.93	4.33	0.59	tr|B9EKJ3|B9EKJ3_MOUSE	Protein MON2 homolog OS=Mus musculus OX=10090 GN=Mon2 PE=1 SV=1	MOUSE	1
3336	0.73	0.83	8.46	1.38	tr|Q9QZA3|Q9QZA3_MOUSE	Synapsin IIIa OS=Mus musculus OX=10090 GN=Syn3 PE=2 SV=1	MOUSE	1

3337	0.73	0.83	7.58	2.40	sp|Q6NS82|RETR2_MOUSE	Reticulophagy regulator 2 OS=Mus musculus OX=10090 GN=Retreg2 PE=1 SV=2	MOUSE	1
3338	0.72	102.62	28.62	18.54	sp|Q8C8R3-4|ANK2_MOUSE	Isoform 4 of Ankyrin-2 OS=Mus musculus OX=10090 GN=Ank2	MOUSE	48
3339	0.72	1.01	4.22	0.53	tr|F8VQD1|F8VQD1_MOUSE	Protein polybromo-1 OS=Mus musculus OX=10090 GN=Pbrm1 PE=1 SV=1	MOUSE	1
3340	0.72	0.83	16.97	5.96	sp|Q9JKX6|NUDT5_MOUSE	ADP-sugar pyrophosphatase OS=Mus musculus OX=10090 GN=Nudt5 PE=1 SV=1	MOUSE	1
3341	0.72	0.82	6.84	1.40	tr|A0A0R4J102|A0A0R4J102_MOUSE	Cytoplasmic polyadenylation element-binding protein 3 OS=Mus musculus OX=10090 GN=Cpeb3 PE=1 SV=1	MOUSE	1
3342	0.72	0.8	4.13	1.65	sp|D3YXJ0|DGKH_MOUSE	Diacylglycerol kinase eta OS=Mus musculus OX=10090 GN=Dgkh PE=1 SV=2	MOUSE	1
3343	0.72	0.79	1.17	1.17	RRRRRsp|Q6PFH3|DCA15_MOUSE	REVERSED DDB1- and CUL4-associated factor 15 OS=Mus musculus OX=10090 GN=Dcaf15 PE=2 SV=1	MOUSE	1
3344	0.71	0.8	44.58	16.87	tr|A0A0N4SW28|A0A0N4SW28_MOUSE	Guanine nucleotide-binding protein subunit gamma OS=Mus musculus OX=10090 GN=Gng12 PE=1 SV=1	MOUSE	1
3345	0.71	0.79	6.71	1.07	sp|Q8VD75|HIP1_MOUSE	Huntingtin-interacting protein 1 OS=Mus musculus OX=10090 GN=Hip1 PE=1 SV=2	MOUSE	1
3346	0.71	0.79	3.08	1.85	tr|Q8BIY0|Q8BIY0_MOUSE	Uncharacterized protein OS=Mus musculus OX=10090 GN=Rnasel PE=2 SV=1	MOUSE	1
3347	0.71	0.79	7.46	3.73	tr|A0A0X1KG67|A0A0X1KG67_MOUSE	ER membrane protein complex subunit 10 OS=Mus musculus OX=10090 GN=Emc10 PE=1 SV=1	MOUSE	1
3348	0.7	0.8	2.19	0.51	tr|E9Q6A7|E9Q6A7_MOUSE	Bromodomain PHD finger transcription factor OS=Mus musculus OX=10090 GN=Bptf PE=1 SV=1	MOUSE	1
3349	0.7	0.8	16.73	8.37	sp|Q2TPA8|HSDL2_MOUSE	Hydroxysteroid dehydrogenase-like protein 2 OS=Mus musculus OX=10090 GN=Hsdl2 PE=1 SV=1	MOUSE	1
3350	0.69	0.8	18.97	0.00	sp|Q8K0D7|GET1_MOUSE	Guided entry of tail-anchored proteins factor 1 OS=Mus musculus OX=10090 GN=Get1 PE=1 SV=1	MOUSE	0
3351	0.69	0.79	7.72	2.36	tr|A0A0R4J0B5|A0A0R4J0B5_MOUSE	RUS family member 1 OS=Mus musculus OX=10090 GN=Rusf1 PE=1 SV=1	MOUSE	1
3352	0.69	0.77	3.31	0.71	tr|K3W4M8|K3W4M8_MOUSE	Adhesion G protein-coupled receptor L3 OS=Mus musculus OX=10090 GN=Adgrl3 PE=1 SV=1	MOUSE	1
3353	0.69	0.77	25.89	8.93	tr|Q925A2|Q925A2_MOUSE	Fibroblast growth factor OS=Mus musculus OX=10090 GN=Fgf2 PE=1 SV=1	MOUSE	1

3354	0.69	0.76	7.77	2.95	tr|Q80U76|Q80U76_MOUSE	Ribosome biogenesis regulatory protein (Fragment) OS=Mus musculus OX=10090 GN=Rrs1 PE=2 SV=2	MOUSE	1
3355	0.69	0.76	4.87	2.02	tr|Q9EPU8|Q9EPU8_MOUSE	Sodium-calcium exchanger 3 (Fragment) OS=Mus musculus OX=10090 GN=Slc8a3 PE=2 SV=2	MOUSE	1
3356	0.69	0.76	7.88	2.96	tr|Q8C1Y9|Q8C1Y9_MOUSE	Uncharacterized protein OS=Mus musculus OX=10090 GN=Arl6ip1 PE=2 SV=1	MOUSE	1
3357	0.69	0.76	1.25	1.25	tr|V9GX81|V9GX81_MOUSE	Maestro heat-like repeat family member 6 OS=Mus musculus OX=10090 GN=Mroh6 PE=1 SV=1	MOUSE	1
3358	0.69	0.76	3.16	3.16	tr|Q3UEW2|Q3UEW2_MOUSE	HP domain-containing protein OS=Mus musculus OX=10090 GN=Dmtn PE=2 SV=1	MOUSE	1
3359	0.69	0.76	10.87	10.87	sp|Q9D1Q4|DPM3_MOUSE	Dolichol-phosphate mannosyltransferase subunit 3 OS=Mus musculus OX=10090 GN=Dpm3 PE=1 SV=1	MOUSE	1
3360	0.68	0.79	13.19	3.83	tr|Q3UXF7|Q3UXF7_MOUSE	Uncharacterized protein (Fragment) OS=Mus musculus OX=10090 GN=Scfd2 PE=2 SV=1	MOUSE	1
3361	0.68	0.76	3.20	0.85	tr|Q6KAM6|Q6KAM6_MOUSE	MFLJ00369 protein (Fragment) OS=Mus musculus OX=10090 GN=Arhgef1 PE=2 SV=1	MOUSE	1
3362	0.68	0.75	16.08	10.49	sp|Q8C6B9|AROS_MOUSE	Active regulator of SIRT1 OS=Mus musculus OX=10090 GN=Rps19bp1 PE=1 SV=1	MOUSE	1
3363	0.67	0.74	3.81	3.81	tr|Q0VGS1|Q0VGS1_MOUSE	Vasohibin 1 OS=Mus musculus OX=10090 GN=Vash1 PE=2 SV=1	MOUSE	1
3364	0.67	0.74	2.94	2.94	RRRRRtr|A0A0J9YV03|A0A0J9YV03_MOUSE	REVERSED Protein FAM187B OS=Mus musculus OX=10090 GN=Fam187b PE=3 SV=1	MOUSE	1
3365	0.66	0.91	7.23	3.03	sp|Q5SUA5|MYO1G_MOUSE	Unconventional myosin-Ig OS=Mus musculus OX=10090 GN=Myo1g PE=1 SV=1	MOUSE	2
3366	0.66	0.74	15.90	5.64	sp|Q6P3D0|NUD16_MOUSE	U8 snoRNA-decapping enzyme OS=Mus musculus OX=10090 GN=Nudt16 PE=1 SV=1	MOUSE	1
3367	0.66	0.73	3.76	0.98	sp|Q9EPQ2|RPGR1_MOUSE	X-linked retinitis pigmentosa GTPase regulator-interacting protein 1 OS=Mus musculus OX=10090 GN=Rpgrip1 PE=1 SV=1	MOUSE	1
3368	0.66	0.73	2.60	2.60	tr|Q9DCV1|Q9DCV1_MOUSE	Cathepsin C OS=Mus musculus OX=10090 GN=Ctsc PE=2 SV=1	MOUSE	1

3369	0.65	0.75	7.41	2.53	tr|B9EKS7|B9EKS7_MOUSE	CDP-diacylglycerol--glycerol-3-phosphate 3-phosphatidyltransferase OS=Mus musculus OX=10090 GN=Pgs1 PE=1 SV=1	MOUSE	1
3370	0.65	0.75	11.67	4.10	sp|Q91Z49|UIF_MOUSE	UAP56-interacting factor OS=Mus musculus OX=10090 GN=Fyttd1 PE=1 SV=1	MOUSE	1
3371	0.65	0.72	3.97	1.67	tr|B2RPY3|B2RPY3_MOUSE	F-box and leucine-rich repeat protein 16 OS=Mus musculus OX=10090 GN=Fbxl16 PE=1 SV=1	MOUSE	1
3372	0.65	0.72	4.38	4.38	tr|Q3UGB8|Q3UGB8_MOUSE	Derlin OS=Mus musculus OX=10090 GN=Derl1 PE=2 SV=1	MOUSE	1
3373	0.65	0.72	4.60	4.60	tr|A0A3Q4L2U5|A0A3Q4L2U5_MOUSE	Solute carrier family 25 member 46 OS=Mus musculus OX=10090 GN=Slc25a46 PE=1 SV=1	MOUSE	1
3374	0.64	19.49	12.00	6.68	tr|A0A0R4J170|A0A0R4J170_MOUSE	Transcription activator BRG1 OS=Mus musculus OX=10090 GN=Smarca4 PE=1 SV=1	MOUSE	11
3375	0.64	0.74	10.11	5.32	sp|Q9CPR1|RWDD4_MOUSE	RWD domain-containing protein 4 OS=Mus musculus OX=10090 GN=Rwdd4 PE=2 SV=1	MOUSE	1
3376	0.64	0.72	4.95	1.85	tr|Q8K131|Q8K131_MOUSE	Beta-galactosidase OS=Mus musculus OX=10090 GN=Glb1 PE=2 SV=1	MOUSE	1
3377	0.63	0.82	3.64	1.25	tr|Q3UHC9|Q3UHC9_MOUSE	Potassium voltage-gated channel subfamily H member 1 OS=Mus musculus OX=10090 GN=Kcnh1 PE=1 SV=1	MOUSE	2
3378	0.63	0.71	20.90	12.69	tr|F8WJI3|F8WJI3_MOUSE	Coiled-coil domain-containing protein 58 OS=Mus musculus OX=10090 GN=Ccdc58 PE=1 SV=1	MOUSE	1
3379	0.63	0.71	6.03	3.45	RRRRRtr|Q6RJ37|Q6RJ37_MOUSE	REVERSED MHC class I heavy chain maturation peptide H-2K(D) (Fragment) OS=Mus musculus OX=10090 GN=H2-K1 PE=2 SV=1	MOUSE	1
3380	0.63	0.69	1.15	0.46	RRRRRtr|B7ZN28|B7ZN28_MOUSE	REVERSED Papilin OS=Mus musculus OX=10090 GN=Papln PE=1 SV=1	MOUSE	1
3381	0.63	0.69	5.62	5.62	tr|Q9CSA3|Q9CSA3_MOUSE	RING-type E3 ubiquitin transferase (Fragment) OS=Mus musculus OX=10090 GN=Rnf138 PE=2 SV=1	MOUSE	1
3382	0.63	0.69	2.07	2.07	tr|Q3TYJ1|Q3TYJ1_MOUSE	Solute carrier family 18 (Vesicular monoamine), member 3 OS=Mus musculus OX=10090 GN=Slc18a3 PE=1 SV=1	MOUSE	1
3383	0.63	0.68	1.54	0.70	RRRRRsp|P70298|CUX2_MOUSE	REVERSED Homeobox protein cut-like 2 OS=Mus musculus OX=10090 GN=Cux2 PE=2 SV=2	MOUSE	1
3384	0.63	0.68	1.54	0.87	tr|Q8CFX3|Q8CFX3_MOUSE	Protocadherin 1 OS=Mus musculus OX=10090 GN=Pcdh1 PE=1 SV=1	MOUSE	1

3385	0.62	0.69	1.67	0.68	tr|A0A5H1ZRL7|A0A5H1ZRL7_MOUSE	Transformation-related protein 53-binding protein 1 OS=Mus musculus OX=10090 GN=Trp53bp1 PE=1 SV=1	MOUSE	1
3386	0.62	0.68	8.08	3.85	tr|E9QN77|E9QN77_MOUSE	Transmembrane and ubiquitin-like domain-containing protein 1 OS=Mus musculus OX=10090 GN=Tmub1 PE=1 SV=1	MOUSE	1
3387	0.62	0.67	18.10	18.10	tr|Q45TP8|Q45TP8_MOUSE	CKLF-like MARVEL transmembrane domain-containing protein 5 OS=Mus musculus OX=10090 GN=Cmtm5 PE=1 SV=1	MOUSE	1
3388	0.61	0.68	4.68	2.27	tr|Q91WW6|Q91WW6_MOUSE	TATA-binding protein associated factor TAFII135 (Fragment) OS=Mus musculus OX=10090 GN=Taf4 PE=2 SV=1	MOUSE	1
3389	0.61	0.67	6.31	3.40	tr|Q8R0L6|Q8R0L6_MOUSE	Coro2a protein (Fragment) OS=Mus musculus OX=10090 GN=Coro2a PE=2 SV=1	MOUSE	1
3390	0.61	0.67	3.69	1.73	tr|F8WJ51|F8WJ51_MOUSE	Netrin-G1 OS=Mus musculus OX=10090 GN=Ntng1 PE=1 SV=2	MOUSE	1
3391	0.61	0.67	9.66	9.66	tr|F6TGV1|F6TGV1_MOUSE	Ribosomal RNA-processing protein 40 (Fragment) OS=Mus musculus OX=10090 GN=Exosc3 PE=1 SV=1	MOUSE	1
3392	0.61	0.66	4.13	4.13	tr|Q3UII2|Q3UII2_MOUSE	Tetraspanin OS=Mus musculus OX=10090 GN=Cd82 PE=1 SV=1	MOUSE	1
3393	0.6	0.68	2.75	0.99	tr|F8VQ93|F8VQ93_MOUSE	PH-interacting protein OS=Mus musculus OX=10090 GN=Phip PE=1 SV=1	MOUSE	1
3394	0.6	0.67	22.22	6.51	sp|Q99KI3|EMC3_MOUSE	ER membrane protein complex subunit 3 OS=Mus musculus OX=10090 GN=Emc3 PE=1 SV=3	MOUSE	1
3395	0.6	0.67	4.11	1.34	RRRRRtr|Q6A011|Q6A011_MOUSE	REVERSED MKIAA0706 protein (Fragment) OS=Mus musculus OX=10090 GN=Kif1c PE=2 SV=1	MOUSE	1
3396	0.6	0.66	2.65	0.69	RRRRRtr|F7C528|F7C528_MOUSE	REVERSED DNA helicase (Fragment) OS=Mus musculus OX=10090 GN=Chd3 PE=1 SV=1	MOUSE	1
3397	0.6	0.65	14.97	5.88	sp|Q8CIT0|CRF_MOUSE	Corticoliberin OS=Mus musculus OX=10090 GN=Crh PE=2 SV=1	MOUSE	1
