# Supplementary material for: Retinal Proteomic Analysis in a Mouse Model of Endotoxin-Induced Uveitis Using Data-Independent Acquisition-Based Mass Spectrometry
Source: Int J Mol Sci. 2022 Jun 9;23(12):6464. doi: 10.3390/ijms23126464 (PMC9223489; doi:10.3390/ijms23126464)
Supplement: Supplementary file 1 [file ijms-23-06464-s001.zip › supplementary pdf/Table S4.pdf]

Table S4. Canonical pathways identified by IPA bioinformatic software with p-value less than 0.05 (p-value was calculated with the Fischer's exact test. Z-score represents activation or inhibition of the corresponding pathways. NaN represents z-score unpredicted.)

No.	Ingenuity Canonical Pathways	p-value	Ratio	z-score	Molecules
1	Synaptogenesis Signaling Pathway	0.000	0.0442	0.277	Ap2s1, Calm3, Cplx4, Mapk3, Nlgn2, Prkaca, Ralb, Rap1a, Rras2, Sgta, Snap25, Sncb, Stxbp1, Vamp2
2	Acute Phase Response Signaling	0.000	0.0595	2.111	Fga, Fgg, Ftl, Hp, Hpx, Mapk3, Ralb, Rap1a, Rras2, Serpina3, Ttr
3	SNARE Signaling Pathway	0.000	0.0662	-1	Calm3, Cplx4, Myl6, Prkaca, Sgta, Snap25, Sncb, Stxbp1, Vamp2
4	Opioid Signaling Pathway	0.000	0.0429	0.905	Ap2s1, Calm3, Clta, Cltb, Gnai1, Gnat1, Mapk3, Prkaca, Ralb, Rap1a, Rgs6, Rras2
5	Iron homeostasis signaling pathway	0.000	0.0647	NaN	Atp6ap1, Atp6v0d1, Atp6v1f, Atp6v1g1, Ftl, Hp, Hpx, Mapk3, Skp1
6	α-Adrenergic Signaling	0.000	0.0741	0	Calm3, Gnai1, Gnat1, Mapk3, Prkaca, Ralb, Rap1a, Rras2
7	Role of MAPK Signaling in Promoting the Pathogenesis of Influenza	0.000	0.0708	0	Atp6ap1, Atp6v0d1, Atp6v1f, Atp6v1g1, Mapk3, Ralb, Rap1a, Rras2
8	Phagosome Maturation	0.000	0.0566	NaN	Atp6ap1, Atp6v0d1, Atp6v1f, Atp6v1g1, Ctsa, Dynlrb1, Lamp2, Snap25, Vamp2
9	EIF2 Signaling	0.000	0.0446	1.134	Eif3k, Mapk3, Ralb, Rap1a, Rpl15, Rpl38, Rplp2, Rps24, Rps29, Rras2
10	Virus Entry via Endocytic Pathways	0.000	0.0673	NaN	Ap2s1, Clta, Cltb, Dnm1, Ralb, Rap1a, Rras2
11	Oncostatin M Signaling	0.000	0.116	1.342	Mapk3, Ralb, Rap1a, Rras2, Stat1

12	Chemokine Signaling	0.000	0.075	0	Calm3, Gnai1, Mapk3, Ralb, Rap1a, Rras2
13	CLEAR Signaling Pathway	0.000	0.0351	0.632	Atp6ap1, Atp6v0d1, Atp6v1f, Atp6v1g1, Ctsa, Mapk3, Ralb, Rap1a, Rras2, Ywhae
14	G Beta Gamma Signaling	0.000	0.0543	0.378	Gnai1, Gnat1, Mapk3, Prkaca, Ralb, Rap1a, Rras2
15	fMLP Signaling in Neutrophils	0.000	0.0534	0	Calm3, Gnai1, Gnat1, Mapk3, Ralb, Rap1a, Rras2
16	CCR3 Signaling in Eosinophils	0.000	0.0519	1	Calm3, Gnai1, Gnat1, Mapk3, Ralb, Rap1a, Rras2
17	CNTF Signaling	0.000	0.0877	1.342	Mapk3, Ralb, Rap1a, Rras2, Stat1
18	Gαi Signaling	0.000	0.05	0.816	Gnai1, Gnat1, Mapk3, Prkaca, Ralb, Rap1a, Rras2
19	Apelin Endothelial Signaling Pathway	0.000	0.0496	0	Calm3, Gnai1, Gnat1, Mapk3, Ralb, Rap1a, Rras2
20	Cardiac Hypertrophy Signaling	0.000	0.0346	0.707	Calm3, Gnai1, Gnat1, Mapk3, Myl6, Prkaca, Ralb, Rap1a, Rras2
21	Thrombopoietin Signaling	0.000	0.0794	1.342	Mapk3, Ralb, Rap1a, Rras2, Stat1
22	IGF-1 Signaling	0.000	0.0577	1.342	Mapk3, Prkaca, Ralb, Rap1a, Rras2, Ywhae
23	Colorectal Cancer Metastasis Signaling	0.000	0.0332	1.633	Gnai1, Gnat1, Mapk3, Msh6, Prkaca, Ralb, Rap1a, Rras2, Stat1
24	Estrogen Receptor Signaling	0.000	0.027	-0.333	Atp5f1d, Gnai1, Gnat1, Mapk3, Myl6, Ndufb5, Ndufs8, Prkaca, Ralb, Rap1a, Rras2
25	Role of JAK1 and JAK3 in γc Cytokine Signaling	0.000	0.0725	NaN	Mapk3, Ralb, Rap1a, Rras2, Stat1
26	GM-CSF Signaling	0.000	0.0714	1.342	Mapk3, Ralb, Rap1a, Rras2, Stat1
27	Role of Tissue Factor in Cancer	0.000	0.0517	NaN	Fga, Fgg, Mapk3, Ralb, Rap1a, Rras2
28	Glucocorticoid Receptor Signaling	0.000	0.0224	NaN	Actl6b, Atp5f1d, Fgg, Fkbp5, Hp, Mapk3, Ndufb5, Ndufs8, Prkaca, Ralb, Rap1a, Rras2, Stat1

29	CXCR4 Signaling	0.000	0.0414	0	Gnai1, Gnat1, Mapk3, Myl6, Ralb, Rap1a, Rras2
30	Renin-Angiotensin Signaling	0.000	0.0492	1.342	Mapk3, Prkaca, Ralb, Rap1a, Rras2, Stat1
31	IL-3 Signaling	0.000	0.0633	1.342	Mapk3, Ralb, Rap1a, Rras2, Stat1
32	Renal Cell Carcinoma Signaling	0.000	0.0625	1	Eloc, Mapk3, Ralb, Rap1a, Rras2
33	Regulation of eIF4 and p70S6K Signaling	0.000	0.0391	1	Eif3k, Mapk3, Ralb, Rap1a, Rps24, Rps29, Rras2
34	Endocannabinoid Developing Neuron Pathway	0.000	0.0472	0.447	Gnai1, Mapk3, Prkaca, Ralb, Rap1a, Rras2
35	FLT3 Signaling in Hematopoietic Progenitor Cells	0.000	0.061	1.342	Mapk3, Ralb, Rap1a, Rras2, Stat1
36	JAK/STAT Signaling	0.000	0.061	1.342	Mapk3, Ralb, Rap1a, Rras2, Stat1
37	Synaptic Long Term Potentiation	0.000	0.0458	0.816	Calm3, Mapk3, Prkaca, Ralb, Rap1a, Rras2
38	p70S6K Signaling	0.000	0.0455	0.447	Gnai1, Mapk3, Ralb, Rap1a, Rras2, Ywhae
39	Ferroptosis Signaling Pathway	0.000	0.0455	0	Ftl, H2ax, Mapk3, Ralb, Rap1a, Rras2
40	P2Y Purigenic Receptor Signaling Pathway	0.000	0.0451	1.633	Gnai1, Mapk3, Prkaca, Ralb, Rap1a, Rras2
41	Prolactin Signaling	0.000	0.0581	1.342	Mapk3, Ralb, Rap1a, Rras2, Stat1
42	PDGF Signaling	0.000	0.0581	1.342	Mapk3, Ralb, Rap1a, Rras2, Stat1
43	BMP signaling pathway	0.000	0.0575	1.342	Mapk3, Prkaca, Ralb, Rap1a, Rras2
44	GNRH Signaling	0.000	0.0365	0.816	Calm3, Gnai1, Mapk3, Prkaca, Ralb, Rap1a, Rras2

45	Regulation of Cellular Mechanics by Calpain Protease	0.000	0.0562	1	Ezr, Mapk3, Ralb, Rap1a, Rras2
46	Melanoma Signaling	0.000	0.08	1	Mapk3, Ralb, Rap1a, Rras2
47	Insulin Receptor Signaling	0.000	0.0429	0.816	Mapk3, Prkaca, Ralb, Rap1a, Rras2, Vamp2
48	UVC-Induced MAPK Signaling	0.000	0.0784	1	Mapk3, Ralb, Rap1a, Rras2
49	Hereditary Breast Cancer Signaling	0.000	0.0423	NaN	Actl6b, H2ax, Msh6, Ralb, Rap1a, Rras2
50	Xenobiotic Metabolism General Signaling Pathway	0.000	0.042	0.816	Ftl, Mapk3, Mgst3, Ralb, Rap1a, Rras2
51	Sertoli Cell-Sertoli Cell Junction Signaling	0.000	0.034	NaN	Epn2, Mapk3, Prkaca, Ralb, Rap1a, Rras2, Ybx3
52	Phototransduction Pathway	0.000	0.0741	NaN	Gnat1, Gngt1, Guca1a, Prkaca
53	Melanocyte Development and Pigmentation Signaling	0.000	0.051	1.342	Mapk3, Prkaca, Ralb, Rap1a, Rras2
54	UVA-Induced MAPK Signaling	0.000	0.051	1.342	Mapk3, Ralb, Rap1a, Rras2, Stat1
55	VEGF Signaling	0.000	0.0505	1	Mapk3, Ralb, Rap1a, Rras2, Ywhae
56	mTOR Signaling	0.001	0.033	1	Eif3k, Mapk3, Ralb, Rap1a, Rps24, Rps29, Rras2
57	Oxytocin Signaling Pathway	0.001	0.0285	0	Calm3, Gnai1, Mapk3, Myl6, Prkaca, Ralb, Rap1a, Rras2
58	Cancer Drug Resistance By Drug Efflux	0.001	0.069	NaN	Mapk3, Ralb, Rap1a, Rras2
59	Huntington's Disease Signaling	0.001	0.0283	NaN	Atp5f1d, Clta, Cltb, Dnm1, Gls, Mapk3, Snap25, Vamp2
60	Ovarian Cancer Signaling	0.001	0.038	1.342	Mapk3, Msh6, Prkaca, Ralb, Rap1a, Rras2

61	Endometrial Cancer Signaling	0.001	0.0667	1	Mapk3, Ralb, Rap1a, Rras2
62	Role of NFAT in Cardiac Hypertrophy	0.001	0.0315	1.134	Calm3, Gnai1, Mapk3, Prkaca, Ralb, Rap1a, Rras2
63	IL-2 Signaling	0.001	0.0656	1	Mapk3, Ralb, Rap1a, Rras2
64	Thrombin Signaling	0.001	0.0311	0.447	Gnai1, Gnat1, Mapk3, Myl6, Ralb, Rap1a, Rras2
65	ERB2-ERBB3 Signaling	0.001	0.0615	1	Mapk3, Ralb, Rap1a, Rras2
66	CDK5 Signaling	0.001	0.0439	0.447	Mapk3, Prkaca, Ralb, Rap1a, Rras2
67	NRF2-mediated Oxidative Stress Response	0.001	0.0295	0.447	Fkbp5, Ftl, Mapk3, Mgst3, Ralb, Rap1a, Rras2
68	ERBB4 Signaling	0.001	0.0588	1	Mapk3, Ralb, Rap1a, Rras2
69	Remodeling of Epithelial Adherens Junctions	0.001	0.0588	NaN	Clip1, Dnm1, Hgs, Nme1
70	PAK Signaling	0.001	0.0424	0.447	Mapk3, Myl6, Ralb, Rap1a, Rras2
71	SPINK1 General Cancer Pathway	0.001	0.058	1	Mapk3, Ralb, Rap1a, Rras2
72	MSP-RON Signaling In Macrophages Pathway	0.001	0.042	0.447	Mapk3, Ralb, Rap1a, Rras2, Stat1
73	Tight Junction Signaling	0.001	0.0337	NaN	Myl6, Prkaca, Safb, Snap25, Vamp2, Ybx3
74	Agrin Interactions at Neuromuscular Junction	0.001	0.0571	1	Mapk3, Ralb, Rap1a, Rras2
75	Melatonin Signaling	0.001	0.0556	0	Calm3, Gnai1, Mapk3, Prkaca
76	Glioma Invasiveness Signaling	0.001	0.0548	1	Mapk3, Ralb, Rap1a, Rras2
77	Glioma Signaling	0.001	0.0403	0.447	Calm3, Mapk3, Ralb, Rap1a, Rras2
78	Gαs Signaling	0.001	0.04	NaN	Gnai1, Gnat1, Mapk3, Prkaca, Rap1a

79	ERK5 Signaling	0.001	0.0541	0	Ralb, Rap1a, Rras2, Ywhae
80	14-3-3-mediated Signaling	0.002	0.0394	0.447	Mapk3, Ralb, Rap1a, Rras2, Ywhae
81	GDNF Family Ligand-Receptor Interactions	0.002	0.0526	1	Mapk3, Ralb, Rap1a, Rras2
82	Antiproliferative Role of Somatostatin Receptor 2	0.002	0.0519	1	Mapk3, Ralb, Rap1a, Rras2
83	Endothelin-1 Signaling	0.002	0.0312	0	Gnai1, Gnat1, Mapk3, Ralb, Rap1a, Rras2
84	NF-κB Activation by Viruses	0.002	0.0513	1	Mapk3, Ralb, Rap1a, Rras2
85	Neurotrophin/TRK Signaling	0.002	0.0513	1	Mapk3, Ralb, Rap1a, Rras2
86	Thyroid Cancer Signaling	0.002	0.0506	1	Mapk3, Ralb, Rap1a, Rras2
87	Synaptic Long Term Depression	0.002	0.0308	0	Gnai1, Gnat1, Mapk3, Ralb, Rap1a, Rras2
88	Gα12/13 Signaling	0.002	0.0376	0.447	Mapk3, Myl6, Ralb, Rap1a, Rras2
89	GABA Receptor Signaling	0.002	0.0376	NaN	Ap2s1, Dnm1, Gabrg2, Gnai1, Gnat1
90	Hepatic Fibrosis Signaling Pathway	0.002	0.0214	-1	Calm3, Ftl, Gnai1, Mapk3, Myl6, Prkaca, Ralb, Rap1a, Rras2
91	Oxytocin In Brain Signaling Pathway	0.002	0.0305	0	Calm3, Gnai1, Mapk3, Ralb, Rap1a, Rras2
92	Signaling by Rho Family GTPases	0.002	0.0261	1	Clip1, Ezr, Gnai1, Gnat1, Mapk3, Myl6, Septin6
93	Gap Junction Signaling	0.002	0.0303	NaN	Gnai1, Mapk3, Prkaca, Ralb, Rap1a, Rras2
94	Estrogen-Dependent Breast Cancer Signaling	0.002	0.0494	1	Mapk3, Ralb, Rap1a, Rras2
95	PI3K/AKT Signaling	0.002	0.0302	0	Cdc37, Mapk3, Ralb, Rap1a, Rras2, Ywhae
96	Adrenomedullin signaling pathway	0.002	0.0302	0.816	Calm3, Mapk3, Prkaca, Ralb, Rap1a, Rras2

97	Role of MAPK Signaling in the Pathogenesis of Influenza	0.002	0.0488	NaN	Mapk3, Ralb, Rap1a, Rras2
98	Ephrin Receptor Signaling	0.002	0.0296	0	Gnai1, Gnat1, Mapk3, Ralb, Rap1a, Rras2
99	VEGF Family Ligand-Receptor Interactions	0.002	0.0476	1	Mapk3, Ralb, Rap1a, Rras2
100	PEDF Signaling	0.002	0.0476	1	Mapk3, Ralb, Rap1a, Rras2
101	MSP-RON Signaling In Cancer Cells Pathway	0.002	0.0357	0.447	Mapk3, Ralb, Rap1a, Rras2, Ywhae
102	LPS-stimulated MAPK Signaling	0.002	0.0471	1	Mapk3, Ralb, Rap1a, Rras2
103	PI3K Signaling in B Lymphocytes	0.003	0.035	0.447	Calm3, Mapk3, Ralb, Rap1a, Rras2
104	IL-8 Signaling	0.003	0.0286	0.447	Gnai1, Gnat1, Mapk3, Ralb, Rap1a, Rras2
105	Ceramide Signaling	0.003	0.0444	1	Mapk3, Ralb, Rap1a, Rras2
106	Autophagy	0.003	0.0279	0.447	Calm3, Gorasp2, Lamp2, Mapk3, Prkaca, Ralb
107	Acute Myeloid Leukemia Signaling	0.003	0.044	1	Mapk3, Ralb, Rap1a, Rras2
108	Apelin Adipocyte Signaling Pathway	0.003	0.044	0	Gnai1, Mapk3, Mgst3, Prkaca
109	ERK/MAPK Signaling	0.003	0.0278	1.633	Mapk3, Prkaca, Ralb, Rap1a, Rras2, Stat1
110	Corticotropin Releasing Hormone Signaling	0.003	0.0331	0	Calm3, Gnai1, Mapk3, Prkaca, Rap1a
111	Non-Small Cell Lung Cancer Signaling	0.003	0.0426	1	Mapk3, Ralb, Rap1a, Rras2
112	ERBB Signaling	0.003	0.0426	1	Mapk3, Ralb, Rap1a, Rras2
113	Relaxin Signaling	0.004	0.0323	NaN	Gnai1, Gnat1, Mapk3, Prkaca, Rap1a

114	TGF-β Signaling	0.004	0.0417	1	Mapk3, Ralb, Rap1a, Rras2
115	Extrinsic Prothrombin Activation Pathway	0.005	0.125	NaN	Fga, Fgg
116	Mouse Embryonic Stem Cell Pluripotency	0.005	0.0385	1	Mapk3, Ralb, Rap1a, Rras2
117	Apoptosis Signaling	0.005	0.0385	-1	Mapk3, Ralb, Rap1a, Rras2
118	Neuroinflammation Signaling Pathway	0.005	0.0221	0.447	Calb1, Calb2, Gabrg2, Gls, Glul, Mapk3, Stat1
119	Androgen Signaling	0.005	0.0296	NaN	Calm3, Gnai1, Gnat1, Mapk3, Prkaca
120	Chronic Myeloid Leukemia Signaling	0.005	0.0374	NaN	Mapk3, Ralb, Rap1a, Rras2
121	Telomerase Signaling	0.005	0.0374	1	Mapk3, Ralb, Rap1a, Rras2
122	PPAR Signaling	0.005	0.0374	-1	Mapk3, Ralb, Rap1a, Rras2
123	Germ Cell-Sertoli Cell Junction Signaling	0.005	0.0292	NaN	Epn2, Mapk3, Ralb, Rap1a, Rras2
124	Actin Cytoskeleton Signaling	0.006	0.0245	0.816	Ezr, Mapk3, Myl6, Ralb, Rap1a, Rras2
125	Axonal Guidance Signaling	0.006	0.0177	NaN	Gnai1, Gnat1, Mapk3, Myl6, Plxnb2, Prkaca, Ralb, Rap1a, Rras2
126	Glutamine Biosynthesis I	0.007	1	NaN	Glul
127	Prostate Cancer Signaling	0.007	0.0351	NaN	Mapk3, Ralb, Rap1a, Rras2
128	Bladder Cancer Signaling	0.007	0.0345	1	Mapk3, Ralb, Rap1a, Rras2
129	Neuregulin Signaling	0.007	0.0342	1	Mapk3, Ralb, Rap1a, Rras2
130	Fc Epsilon RI Signaling	0.008	0.0339	1	Mapk3, Ralb, Rap1a, Rras2
131	Cholecystokinin/Gastrin-mediated Signaling	0.008	0.0336	1	Mapk3, Ralb, Rap1a, Rras2

132	Role of NANOG in Mammalian Embryonic Stem Cell Pluripotency	0.008	0.0333	1	Mapk3, Ralb, Rap1a, Rras2
133	NGF Signaling	0.008	0.0333	1	Mapk3, Ralb, Rap1a, Rras2
134	Circadian Rhythm Signaling	0.009	0.0224	NaN	Gnai1, Mapk3, Prkaca, Ralb, Rap1a, Rras2
135	Regulation Of The Epithelial Mesenchymal Transition By Growth Factors Pathway	0.009	0.026	1.342	Mapk3, Mest, Ralb, Rap1a, Rras2
136	Clathrin-mediated Endocytosis Signaling	0.009	0.0259	NaN	Ap2s1, Clta, Cltb, Dnm1, Hgs
137	Insulin Secretion Signaling Pathway	0.009	0.0221	0.816	Mapk3, Prkaca, Rap1a, Snap25, Stat1, Vamp2
138	Glutamate Receptor Signaling	0.009	0.0455	NaN	Calm3, Gls, Glul
139	PPARα/RXRα Activation	0.009	0.0256	-0.447	Mapk3, Prkaca, Ralb, Rap1a, Rras2
140	IL-6 Signaling	0.010	0.0312	1	Mapk3, Ralb, Rap1a, Rras2
141	ID1 Signaling Pathway	0.010	0.025	1.342	Mapk3, Plxnb2, Ralb, Rap1a, Rras2
142	IL-22 Signaling	0.011	0.0833	NaN	Mapk3, Stat1
143	Coronavirus Pathogenesis Pathway	0.011	0.0246	-0.447	Atp6ap1, Mapk3, Rps24, Rps29, Stat1
144	HGF Signaling	0.011	0.0303	1	Mapk3, Ralb, Rap1a, Rras2
145	Role of JAK family kinases in IL-6-type Cytokine Signaling	0.011	0.08	NaN	Mapk3, Stat1
146	Ephrin B Signaling	0.012	0.0417	NaN	Gnai1, Gnat1, Mapk3
147	HIF1α Signaling	0.012	0.024	1.342	Eloc, Mapk3, Ralb, Rap1a, Rras2

148	STAT3 Pathway	0.012	0.0296	1	Mapk3, Ralb, Rap1a, Rras2
149	Xenobiotic Metabolism Signaling	0.012	0.0207	NaN	Ftl, Mapk3, Mgst3, Ralb, Rap1a, Rras2
150	Sirtuin Signaling Pathway	0.013	0.0205	-2	Atp5f1d, Gls, Mapk3, Ndufb5, Ndufs8, Timm13
151	RAC Signaling	0.013	0.029	1	Mapk3, Ralb, Rap1a, Rras2
152	Glutamine Degradation I	0.013	0.5	NaN	Gls
153	Macropinocytosis Signaling	0.014	0.0395	NaN	Ralb, Rap1a, Rras2
154	Angiopoietin Signaling	0.014	0.039	NaN	Ralb, Rap1a, Rras2
155	Calcium Signaling	0.014	0.0229	0	Calm3, Mapk3, Myl6, Prkaca, Rap1a
156	Role of BRCA1 in DNA Damage Response	0.016	0.0375	NaN	Actl6b, Msh6, Stat1
157	Dilated Cardiomyopathy Signaling Pathway	0.017	0.027	1	Dmd, Mapk3, Myl6, Prkaca
158	HER-2 Signaling in Breast Cancer	0.017	0.022	1	Mapk3, Nup160, Ralb, Rap1a, Rras2
159	PTEN Signaling	0.017	0.0267	-1	Mapk3, Ralb, Rap1a, Rras2
160	FcγRIIB Signaling in B Lymphocytes	0.019	0.0353	NaN	Ralb, Rap1a, Rras2
161	Protein Kinase A Signaling	0.019	0.0171	0	Calm3, Gnai1, Mapk3, Myl6, Prkaca, Rap1a, Ywhae
162	Hypusine Biosynthesis	0.019	0.333	NaN	Eif5a
163	cAMP-mediated signaling	0.019	0.0213	-0.447	Calm3, Gnai1, Mapk3, Prkaca, Rap1a
164	Epithelial Adherens Junction Signaling	0.020	0.0255	-2	Ralb, Rap1a, Rras2, Ywhae

165	Role of Macrophages, Fibroblasts and Endothelial Cells in Rheumatoid Arthritis	0.021	0.0183	NaN	Calm3, Mapk3, Mif, Ralb, Rap1a, Rras2
166	Coagulation System	0.022	0.0571	NaN	Fga, Fgg
167	MIF-mediated Glucocorticoid Regulation	0.023	0.0556	NaN	Mapk3, Mif
168	Actin Nucleation by ARP-WASP Complex	0.023	0.0323	NaN	Ralb, Rap1a, Rras2
169	IL-4 Signaling	0.023	0.0323	NaN	Ralb, Rap1a, Rras2
170	HMGB1 Signaling	0.025	0.024	1	Mapk3, Ralb, Rap1a, Rras2
171	IL-1 Signaling	0.025	0.0312	NaN	Gnai1, Gnat1, Prkaca
172	Wound Healing Signaling Pathway	0.026	0.0198	1.342	Mapk3, Ralb, Rap1a, Rras2, Stat1
173	Gαq Signaling	0.026	0.0235	NaN	Calm3, Gnai1, Gnat1, Mapk3
174	Branched-chain α-keto acid Dehydrogenase Complex	0.026	0.25	NaN	Dbt
175	Eumelanin Biosynthesis	0.026	0.25	NaN	Mif
176	Mitochondrial Dysfunction	0.026	0.0234	NaN	Atp5f1d, Ndufb5, Ndufs8, Txn2
177	Glioblastoma Multiforme Signaling	0.026	0.0234	1	Mapk3, Ralb, Rap1a, Rras2
178	Apelin Cardiomyocyte Signaling Pathway	0.028	0.0303	NaN	Gnai1, Mapk3, Myl6
179	Molecular Mechanisms of Cancer	0.028	0.0157	NaN	Gnai1, Gnat1, Mapk3, Prkaca, Ralb, Rap1a, Rras2
180	Erythropoietin Signaling Pathway	0.030	0.0226	1	Mapk3, Ralb, Rap1a, Rras2

181	Tumor Microenvironment Pathway	0.030	0.0223	1	Mapk3, Ralb, Rap1a, Rras2
182	Intrinsic Prothrombin Activation Pathway	0.031	0.0476	NaN	Fga, Fgg
183	Pentose Phosphate Pathway (Oxidative Branch)	0.032	0.2	NaN	Pgls
184	MIF Regulation of Innate Immunity	0.034	0.0455	NaN	Mapk3, Mif
185	G Protein Signaling Mediated by Tubby	0.034	0.0455	NaN	Gnai1, Gnat1
186	Paxillin Signaling	0.034	0.0278	NaN	Ralb, Rap1a, Rras2
187	IL-17 Signaling	0.035	0.0214	1	Mapk3, Ralb, Rap1a, Rras2
188	Oxidative Phosphorylation	0.036	0.027	NaN	Atp5f1d, Ndufb5, Ndufs8
189	iNOS Signaling	0.038	0.0426	NaN	Calm3, Stat1
190	Leukocyte Extravasation Signaling	0.039	0.0207	-1	Ezr, Gnai1, Myl6, Rap1a
191	Glycine Cleavage Complex	0.039	0.167	NaN	Gcsh
192	nNOS Signaling in Skeletal Muscle Cells	0.040	0.0417	NaN	Calm3, Dmd
193	Apelin Muscle Signaling Pathway	0.040	0.0417	NaN	Gnai1, Gnat1
194	PFKFB4 Signaling Pathway	0.040	0.0417	NaN	Mapk3, Prkaca
195	Regulation of the Epithelial-Mesenchymal Transition Pathway	0.040	0.0205	NaN	Mapk3, Ralb, Rap1a, Rras2
196	Pulmonary Healing Signaling Pathway	0.043	0.0201	1	Mapk3, Ralb, Rap1a, Rras2

197	Natural Killer Cell Signaling	0.043	0.0201	1	Mapk3, Ralb, Rap1a, Rras2
198	G-Protein Coupled Receptor Signaling	0.043	0.0128	-0.333	Calm3, Gnai1, Gnat1, Mapk3, Myl6, Prkaca, Ralb, Rap1a, Rras2
199	Cell Cycle: G2/M DNA Damage Checkpoint Regulation	0.043	0.04	NaN	Skp1, Ywhae
200	Nitric Oxide Signaling in the Cardiovascular System	0.044	0.0252	NaN	Calm3, Mapk3, Prkaca
201	Amyloid Processing	0.045	0.0392	NaN	Mapk3, Prkaca
202	Neuroprotective Role of THOP1 in Alzheimer's Disease	0.045	0.025	NaN	Prkaca, Serpina3, Ywhae
203	Acetyl-CoA Biosynthesis I (Pyruvate Dehydrogenase Complex)	0.045	0.143	NaN	Dbt
204	Senescence Pathway	0.047	0.0168	0.447	Calm3, Mapk3, Ralb, Rap1a, Rras2
205	CREB Signaling in Neurons	0.047	0.0132	1.134	Calm3, Gnai1, Gnat1, Mapk3, Prkaca, Ralb, Rap1a, Rras2
206	LXR/RXR Activation	0.047	0.0244	NaN	Fga, Hpx, Ttr
207	RHOA Signaling	0.048	0.0242	NaN	Ezr, Myl6, Septin6
