# Supplementary material for: Retinal Proteomic Analysis in a Mouse Model of Endotoxin-Induced Uveitis Using Data-Independent Acquisition-Based Mass Spectrometry
Source: Int J Mol Sci. 2022 Jun 9;23(12):6464. doi: 10.3390/ijms23126464 (PMC9223489; doi:10.3390/ijms23126464)
Supplement: Supplementary file 1 [file ijms-23-06464-s001.zip › supplementary pdf/Table S5.pdf]

Table S5. Primer sequence for qRT-PCR analyses

Gene	Forward Primer (5′-3′)	Reverse Primer (5′-3′)
GAPDH	GTAGAGGGCATGGTGATTTCCT	CACAGTCTTCTGAGTGGCAGTGAT
IL-1β	CACCCTGCAGCTGGAGAGT	GACAAACCGCTTTTCCATCTTC
IL-6	TAGTCCTTCCTACCCCAATTTCC	TTGGTCCTTAGCCACTCCTTC
iNOS	TGACGGCAAACATGACTTCAG	GCCATCGGGCATCTGGTA
TLR4	ATGGCATGGCTTACACCACC	GAGGCCAATTTTGTCTCCACA
HP	GCACTTGGTTCGCTATCGCT	GCTTCTCGTCGTTTAAGGTGTA
HPX	AGCAGTGGCGCTAAATATCCT	CAACTCTCCCGTTGGCAGTA
FGG	ACCAGAGATAACTGTTGCATCCT	CCACGTCGGTTTGGTAAGAAG
