# Supplementary material for: Retinal Proteomic Analysis in a Mouse Model of Endotoxin-Induced Uveitis Using Data-Independent Acquisition-Based Mass Spectrometry
Source: Int J Mol Sci. 2022 Jun 9;23(12):6464. doi: 10.3390/ijms23126464 (PMC9223489; doi:10.3390/ijms23126464)

Figure S2. FDR analysis of combined IDA library of retina at protein level generated by Protein Pilot software

Protein-Level FDR Analysis

Proteins Identified at Critical False Discovery Rates

	Number of Proteins Detected		
**Critical FDR**	**Local FDR**	*Global FDR*	**Global FDR from Fit**
**1.0%**	*2950*	*3380*	**3397**
**5.0%**	**3166**	*3751*	*3754*
**10.0%**	**3267**	*4300*	*4089*

* *It is recommended you use numbers in bold and avoid using numbers in italics.*

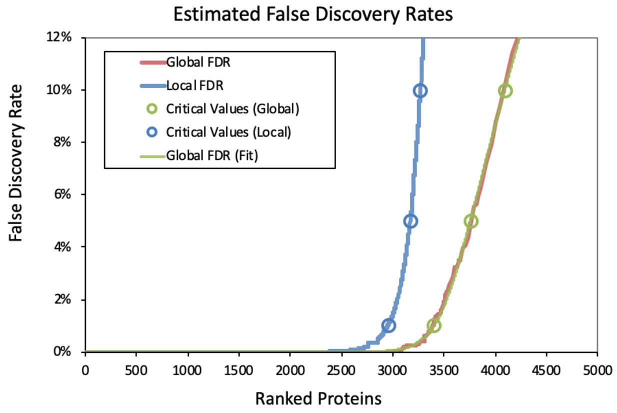

Correspondence between FDR Levels and ProteinPilot Reported Confidences

	Corresponding ProteinPilot Confidence		
**Critical FDR**	**Local FDR**	*Global FDR*	**Global FDR from Fit**
**1.0%**	*98.1%*	*77.1%*	**74.9%**
**5.0%**	**92.4%**	*39.7%*	*38.3%*
**10.0%**	**87.7%**	*22.4%*	*20.6%*

	Corresponding Unused ProtScore		
**Critical FDR**	**Local FDR**	*Global FDR*	**Global FDR from Fit**
**1.0%**	*1.730*	*0.640*	**0.600**
**5.0%**	**1.120**	*0.220*	*0.210*
**10.0%**	**0.910**	*0.110*	*0.100*

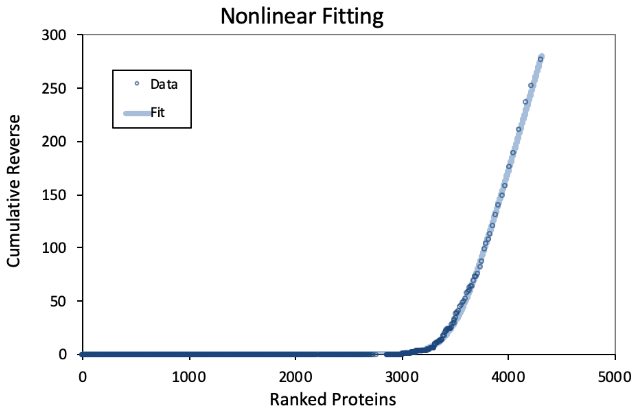

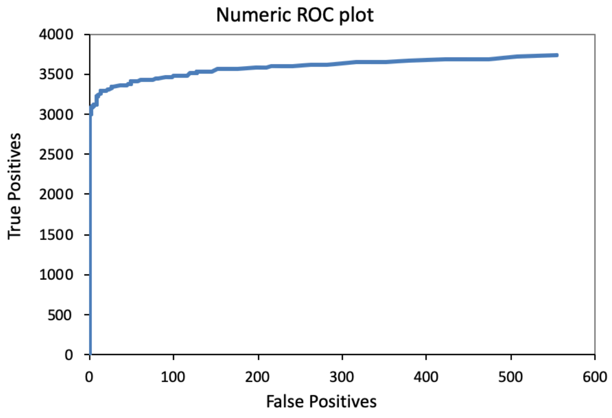

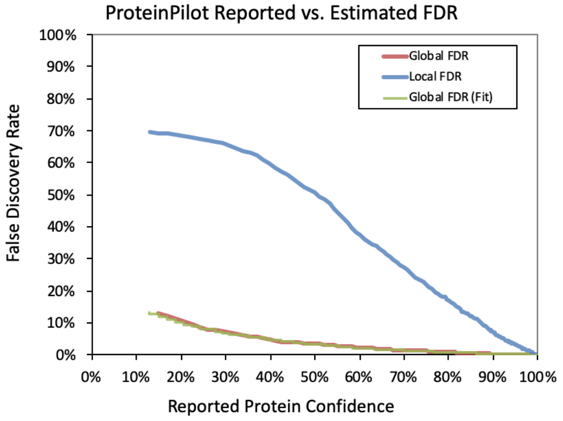

Supplement: Supplementary file 1 [file ijms-23-06464-s001.zip › supplementary pdf/Figure S2.pdf]
