# Supplementary material for: Retinal Proteomic Analysis in a Mouse Model of Endotoxin-Induced Uveitis Using Data-Independent Acquisition-Based Mass Spectrometry
Source: Int J Mol Sci. 2022 Jun 9;23(12):6464. doi: 10.3390/ijms23126464 (PMC9223489; doi:10.3390/ijms23126464)
Supplement: Supplementary file 1 [file ijms-23-06464-s001.zip › supplementary pdf/Figure S3.pdf]

Figure S3. FDR analysis of combined IDA library of retina at peptide level generated by Protein Pilot software

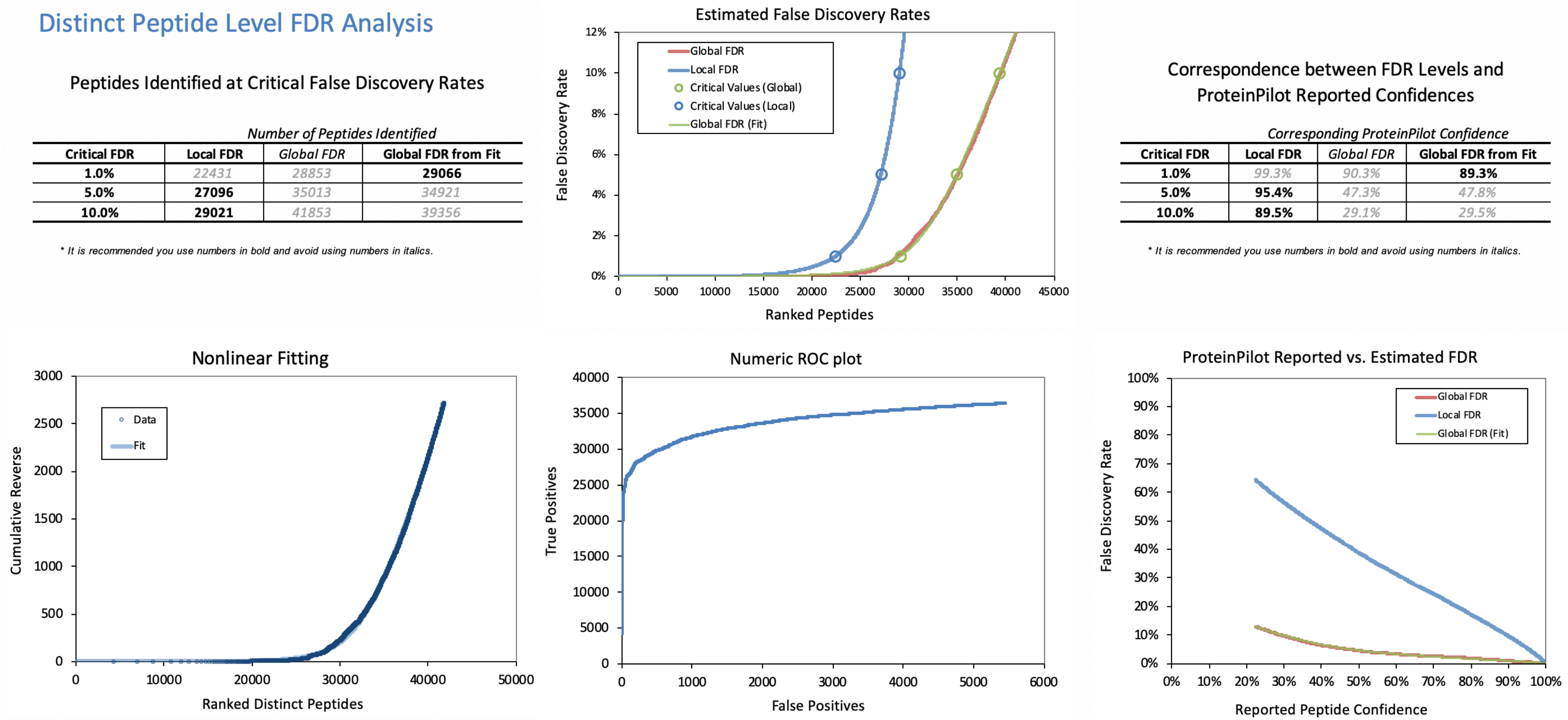

Peptides Identified at Critical False Discovery Rates

	Number of Peptides Identified		
**Critical FDR**	**Local FDR**	*Global FDR*	**Global FDR from Fit**
**1.0%**	*22431*	*28853*	**29066**
**5.0%**	**27096**	*35013*	*34921*
**10.0%**	**29021**	*41853*	*39356*

** It is recommended you use numbers in bold and avoid using numbers in italics.*

	Corresponding ProteinPilot Confidence		
**Critical FDR**	**Local FDR**	*Global FDR*	**Global FDR from Fit**
**1.0%**	*99.3%*	*90.3%*	**89.3%**
**5.0%**	**95.4%**	*47.3%*	*47.8%*
**10.0%**	**89.5%**	*29.1%*	*29.5%*

** It is recommended you use numbers in bold and avoid using numbers in italics.*
