# Supplementary material for: Retinal Proteomic Analysis in a Mouse Model of Endotoxin-Induced Uveitis Using Data-Independent Acquisition-Based Mass Spectrometry
Source: Int J Mol Sci. 2022 Jun 9;23(12):6464. doi: 10.3390/ijms23126464 (PMC9223489; doi:10.3390/ijms23126464)
Supplement: Supplementary file 1 [file ijms-23-06464-s001.zip › supplementary pdf/Figure S4.pdf]

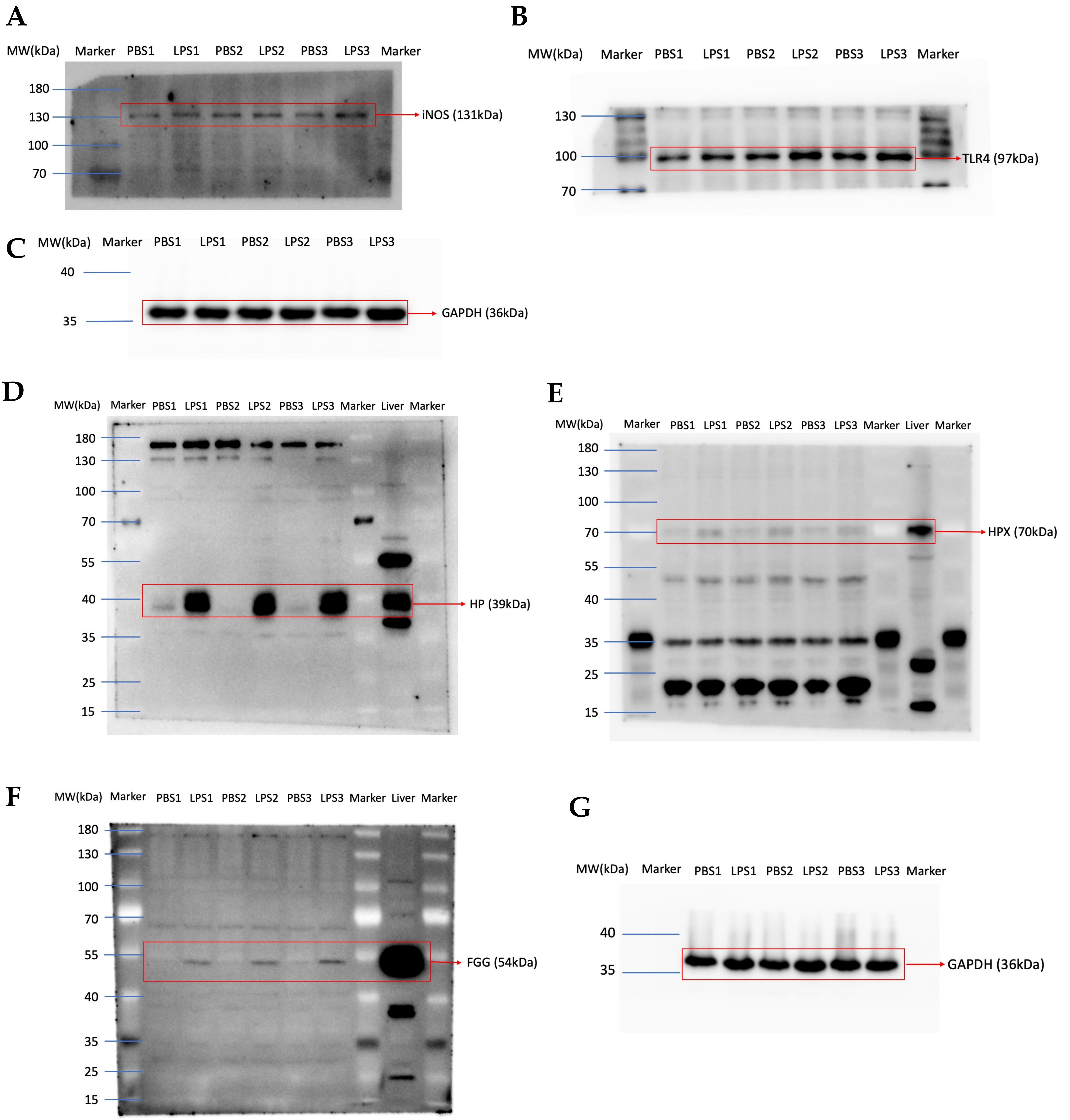

Figure S4. Original western blot images of iNOS (**A**), TLR4 (**B**), GAPDH (**C**) presented in Figure 1E, and HP (**D**), HPX (**E**) , FGG(**F**), and GAPDH (**G**) presented in Figure 6B. The molecular weight markers were labeled at the left side. The red arrowheads and rectangles represent the immunoreactive bands that are used for the quantitative analysis respectively. PBS1, PBS2, PBS3 represent three independent samples derived from C57BL/6J+PBS group. LPS1, LPS2, PLS3 represent three independent samples derived from C57BL/6J+LPS group. The liver tissue of C57BL/6J mice were used as positive control to determine the specific immunoreactive bands in HP, HPX, and FGG. HP, HPX, and FGG protein levels are normalized to GAPDH protein levels using ImageJ.
